# Discovery of Some Heterocyclic Molecules as Bone Morphogenetic Protein 2 (BMP-2)-Inducible Kinase Inhibitors: Virtual Screening, ADME Properties, and Molecular Docking Simulations

**DOI:** 10.3390/molecules27175571

**Published:** 2022-08-30

**Authors:** Amany Belal, Hazem Elkady, Ahmed A. Al-Karmalawy, Ali H. Amin, Mohammed M. Ghoneim, Mohamed El-Sherbiny, Rasha Hamed Al-Serwi, Mohamed Attia Abdou, Mona H. Ibrahim, Ahmed B. M. Mehany

**Affiliations:** 1 Department of Pharmaceutical Chemistry, College of Pharmacy, Taif University, P.O. Box 11099, Taif 21944, Saudi Arabia; 2 Pharmaceutical Medicinal Chemistry & Drug Design Department, Faculty of Pharmacy (Boys), Al-Azhar University, Cairo 11884, Egypt; 3 Department of Pharmaceutical Medicinal Chemistry, Faculty of Pharmacy, Horus University-Egypt, New Damietta 34518, Egypt; 4 Deanship of Scientific Research, Umm Al-Qura University, Makkah 21955, Saudi Arabia; 5 Zoology Department, Faculty of Science, Mansoura University, Mansoura 35516, Egypt; 6 Pharmacognosy and Medicinal Plants Department, Faculty of Pharmacy (Boys), Al-Azhar University, Cairo 11884, Egypt; 7 Department of Pharmacy Practice, College of Pharmacy, AlMaarefa University, Ad Diriyah, Riyadh 13713, Saudi Arabia; 8 Department of Basic Medical Sciences, College of Medicine, AlMaarefa University, P.O. Box 71666, Riyadh 11597, Saudi Arabia; 9 Department of Basic Dental Sciences, College of Dentistry, Princess Nourah bint Abdulrahman University, P.O. Box 84428, Riyadh 11671, Saudi Arabia; 10 Orthopedic Surgery Department, Al Iman General Hospital, Riyadh 11671, Saudi Arabia; 11 Department of Pharmaceutical Medicinal Chemistry and Drug Design, Faculty of Pharmacy (Girls), Al-Azhar University, Cairo 11884, Egypt; 12 Zoology Department, Faculty of Science (Boys), Al-Azhar University, Cairo 11884, Egypt

**Keywords:** benzothiophene, benzofuran, BMP-2 inducible kinase, ADMET, docking

## Abstract

Bone morphogenetic proteins (BMPs) are growth factors that have a vital role in the production of bone, cartilage, ligaments, and tendons. Tumors’ upregulation of bone morphogenetic proteins (BMPs) and their receptors are key features of cancer progression. Regulation of the BMP kinase system is a new promising strategy for the development of anti-cancer drugs. In this work, based on a careful literature study, a library of benzothiophene and benzofuran derivatives was subjected to different computational techniques to study the effect of chemical structure changes on the ability of these two scaffolds to target BMP-2 inducible kinase, and to reach promising candidates with proposed activity against BMP-2 inducible kinase. The results of screening against Lipinski’s and Veber’s Rules produced twenty-one outside eighty-four compounds having drug-like molecular nature. Computational ADMET studies favored ten compounds (**11, 26, 27, 29, 30, 31, 34, 35, 65**, and **72**) with good pharmacokinetic profile. Computational toxicity studies excluded compound **34** to elect nine compounds for molecular docking studies which displayed eight compounds (**26, 27, 29, 30, 31, 35, 65**, and **72**) as promising BMP-2 inducible kinase inhibitors. The nine fascinating compounds will be subjected to extensive screening against serine/threonine kinases to explore their potential against these critical proteins. These promising candidates based on benzothiophene and benzofuran scaffolds deserve further clinical investigation as BMP-2 kinase inhibitors for the treatment of cancer.

## 1. Introduction

Bone morphogenetic proteins (BMPs) are a type of transforming growth factor-β (TGF-β) superfamily that has multiple functions. More than a quarter of a million BMP members have been identified. Cell proliferation, survival, differentiation, and apoptosis are all regulated by BMPs. It is important to produce bone, cartilage, ligaments, and tendons [1].

When BMPs attach to their cell surface receptors on mesenchymal cells, the BMP signaling cascade is initiated, and signals are transmitted to the cell nucleus via particular proteins. The mesenchymal cell becomes a chondrocyte or an osteoblast as a result of the expression of genes that lead to the production of macromolecules involved in cartilage and bone development [2]. Following the implantation of this protein component of the bone matrix, a complex series of cellular events occurred, including mesenchymal cell infiltration, cartilage formation, vascularization, bone formation, and finally remodeling of new bone tissue, as well as population by hematopoietic bone marrow elements [3]. BMPs have been found to directly differentiate cells into the osteoblast phenotype, in addition to chondrocyte lineage differentiation [4]. Bone morphogenetic protein-2 (BMP-2) is the only osteoinductive growth factor approved by the FDA for use as a bone graft alternative.

BMP signaling is associated with cancer-related cellular phenotypes. BMPs can increase cell migration and invasiveness by arresting the cell cycle of many different cell types in the early G1 phase [5]. BMPs have been shown to play a function in a wide range of cancers and other malignancies. BMPs can either suppress or promote tumorigenesis, with the majority of cases favoring metastasis [6]. Intestinal tumorigenesis can be triggered by disruption of the tightly controlled homeostatic BMP signaling gradients [7]. The most important factor of hereditary risk and predisposition for sporadic colorectal cancer (CRC) susceptibility, according to recent research, is variation in the BMP pathway [8]. As rhBMP-2 has been observed to be associated with a higher risk of developing new cancer than vertebral bone graft, its use in spine surgery has been the subject of significant discussion [9]. BMP-2-inducible kinase belongs to the Numb-associated kinase (NAK) family of serine/threonine kinases [10]. BMP-2 is crucial to the occurrence and progression of colon cancer, prostatic carcinoma, and lung cancer [11,12]. Furthermore, BMP-2 is essential for prenatal and postnatal mammary gland development, but it has also been identified in breast cancer cells [13]. Additionally, BMP-2 increases gastric cancer cell motility and invasion by activating PI-3 kinase/Akt; blocking this pathway may limit BMP-2-mediated metastasis [14]. Various heterocyclic molecules are found in a variety of drugs and have become a key research foundation in medicinal chemistry. This is mostly owing to the adaptability and specific physicochemical properties of heterocyclic molecules. Benzofuran is one of the identified heterocyclic compounds [15]. Benzothiophene and Benzofuran scaffold is one of the privileged frameworks in drug development, as this core displays diverse biological activities allowing them to function as anti-convulsant, anti-cancer, anti-diabetic, anti-tubercular, anti-oxidant, anti-inflammatory, anti-microbial, and many other agents [16,17].

**Figure 1:**
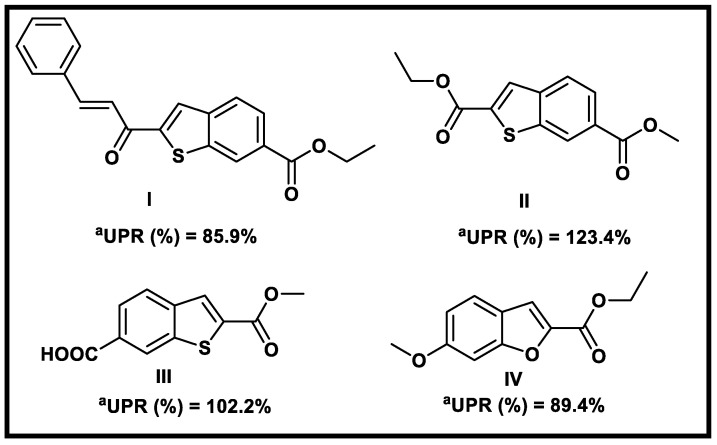
Reported benzothiophene and benzofuran derivatives as BMP-2 up-regulators. ^a^ UPR: Up-regulatory percent Activities on BMP-2 expression.

**Figure 2:**
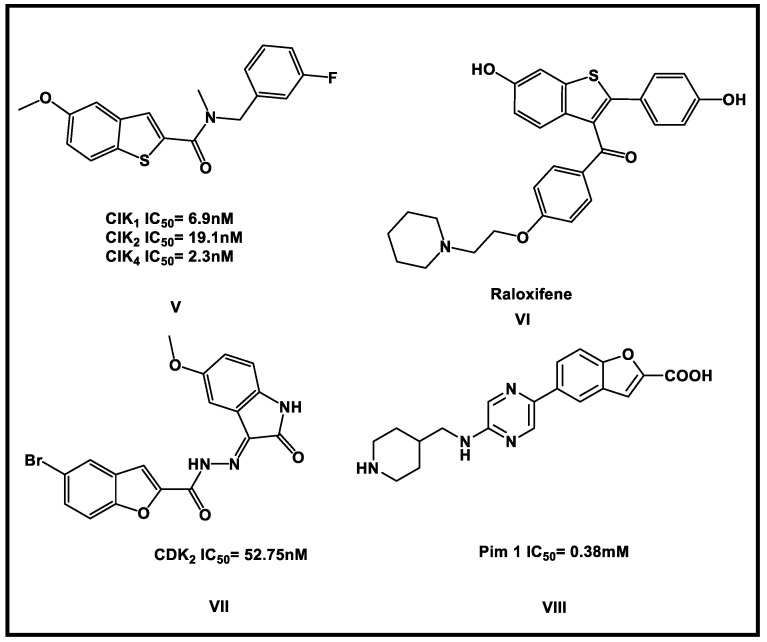
Reported benzothiophenes as CIK1,2,4 inhibitors (V), raloxifene (VI): anti-breast cancer, benzofuran derivatives VII and VIII as CDK2 and Pim1 inhibitors, respectively.

Many literature studies revealed that substituted benzothiophene and benzofuran derivatives were a new class of small molecules that act as potential anabolic agents targeting BMP-2 [18,19]. In particular, compounds **I**, **II III**, and **IV** (Figure 1) enhanced BMP-2 expression in vitro and stimulated bone formation and trabecular connectivity restoration in vivo [18]. In contrast, benzothiophene and benzofuran derivatives can inhibit several protein kinases and act as anticancer agents such as compounds **V**–**VIII** (Figure 2) [16,20,21,22].

In this work, we will try to find new inhibitors for BMP-2 that may be useful in the treatment of several types of cancers. We decided to investigate some reported benzothiophene and benzofuran derivatives [23,24] for their potential as BMP-2 inducible kinase inhibitors. The selected library (Figure S1 Supplementary data) was subjected to different computational techniques such as ADMET, toxicity, and docking studies to reach promising candidates with proposed activity against BMP-2-inducible kinase. Finally, the promising compounds will undergo additional screening against kinases related to serine/threonine kinases (CDK2, Pim1, cell division protein kinase 2, casein kinase II, and eukaryotic translation initiation factor 2-alpha kinase 3).

## 2. Results and Discussion

### 2.1. Virtual Screening

In the current study, an in silico computational study was conducted to determine the number of rotatable bonds, topology polar surface area (TPSA), and other physicochemical properties for the tested candidates according to the directions of Veber’s and Lipinski’s Rules of five [25].

Lipinski proposed that the absorption of an orally administered compound is more likely to be better if the molecule satisfies at least three out of four of the following rules: (1) hydrogen bond donors (HBD) ≤ 5; (2) hydrogen bond acceptors (HBA) ≤ 10; (3) molecular weight < 500; (4) a coefficient of partition between octanol and water logP < 5. Compounds contravening more than one of these rules could not have good bioavailability. Moreover, reduced molecular flexibility, as measured by the number of rotatable bonds, and low polar surface area are found to be important predictors of good oral bioavailability [26]. In this regard, Veber’s Rule says that a compound with 10 or fewer rotatable bonds (RTB) and a polar surface area (TPSA) no greater than 140 ′Å2 should present good oral bioavailability [26].

The results presented in Table 1 showed that twenty-one outside eighty-four tested compounds (**11, 26–35, 40, 61–63, 65**, and **68–72**) showed no contravention of Lipinski’s and Veber’s Rules, respectively, and hence display a drug-like molecular nature.

At first, with respect to the number of hydrogen bond donors (HBD), compounds **11, 26–35, 40, 61–63, 65**, and **68–72** have an HBD range of 0–4. These values are less than 5 and obey the first parameter of Lipinski’s Rule. Moreover, regarding the number of hydrogen bond acceptors (HBA), all the mentioned 21 compounds have HBA ranging from 3–8 (i.e., less than 10), which meets the second parameter Lipinski’s Rule. Concerning the molecular weight parameter of compounds **11, 26–35, 40, 61–63, 65**, and **68–72**, it was found that these compounds have a molecular weight ranging from 208.234 to 499.604. Such values are less than 500, which confirms the third parameter of Lipinski’s Rule. In addition, compounds **11, 26–34, 65, 68**, and **72** demonstrated a logP value range from 1.45 to 4.994. The obtained values match with the fourth parameter of Lipinski’s Rule, which suggests that good drug candidates should have logP values less than 5. According to Veber’s Rule, number of rotatable bonds was calculated, and this is an important parameter to measure the molecular flexibility and oral bioavailability of the drug candidates. The results revealed that compounds **11, 26–35, 40, 61–63, 65**, and **68–72** displayed an acceptable number of rotatable bonds ranging from 2–10 that meet the criteria of Veber’s Rule. Moreover, the number of TPSA (a physicochemical property describing the polarity of molecules) of such compounds is within the acceptable values (ranging from 74.77 to 123.91 Å^2^) of less than 140 Å². On the other hand, the rest of the tested compounds did not meet the criteria of Lipinski’s and Veber’s Rules, respectively. A closer look at the data presented in Table S1 (Supplementary Data) demonstrated that compounds **1–10, 12–25, 36–39, 41–60, 64, 66, 67**, and **73–84** have a molecular weight of more than 500 and logP values of more than 5, which does not meet the criteria of Lipinski’s Rule. Additionally, most of them do not obey Veber’s Rule, as shown in Table S1 (Supplementary Data).

### 2.2. Computational ADMET Analysis

Twenty-one compounds, **11, 26–35, 40, 61–65, 68–72**, met the criteria of Lipinski’s and Veber’s Rules and were further investigated for their pharmacokinetic properties (ADMET studies) according to the reported procedures [27,28,29,30,31,32]. *N*-[6-(3-[[(cyclopropylmethyl)sulfonyl]amino]phenyl)-1*H*-indazol-3-yl]cyclopropanecarboxamide (**IDK**), an indazole inhibitor, is used as a reference molecule in this computational study. Blood–brain barrier (BBB) penetration, aqueous solubility, intestinal absorption, CYP2D6 binding, and plasma protein binding properties of the elected candidates were calculated using Discovery studio 4.0 (Discovery Studio 2016 Vélizy-Villacoublay, France).

**Figure 3:**
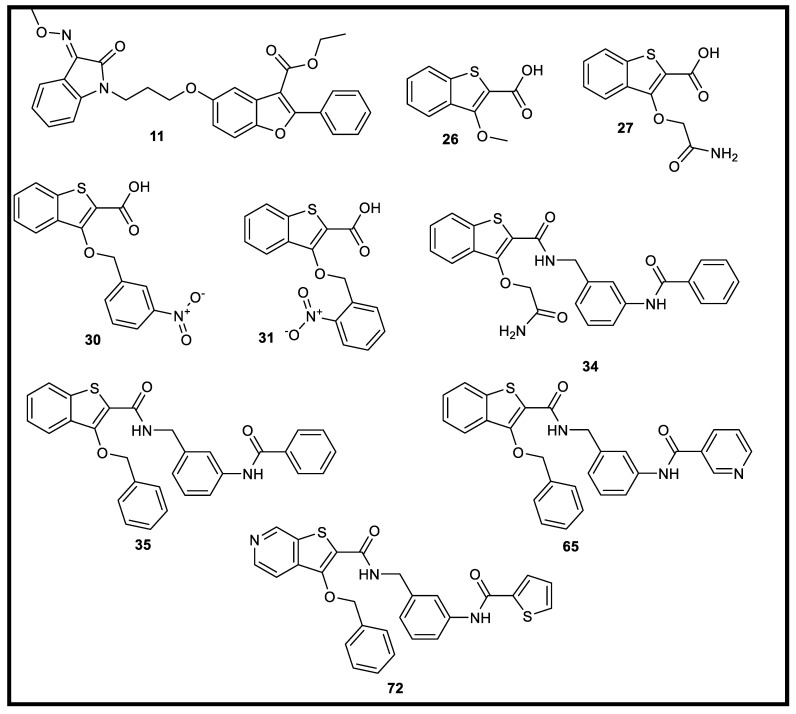
Compounds **11, 26, 27, 30, 31, 34, 35, 65**, and **72** exhibited medium to very low BBB penetration levels.

The results revealed that ten out of twenty-one compounds showed good ADMET profiles and drug-likeness properties. In detail, compounds **11, 26, 27, 29, 30, 31, 34, 35, 65**, and **72** exhibited medium to very low BBB penetration levels (Figure 3); therefore, such compounds were anticipated to be safe for the CNS. In addition, it was found that all of the ten compounds had moderate to good absorption behavior. Moreover, the solubility level of the ten compounds is expected to be better than or even similar to that of the reference drug that showed a low solubility level. For cytochrome P450 2D6 (CYP2D6) inhibition, all the examined members were predicted as non-inhibitors. Finally, all compounds were expected to bind to plasma protein by more than 90% (Table 2).

### 2.3. Computational Toxicity Studies

Based on the previous findings, computational toxicity profiles of the favored ten compounds (**11, 26, 27, 29, 30, 31, 34, 35, 65**, and **72**) were examined following the reported procedures [27,28,29,30,31,32]. Six toxicity models were used through Discovery studio software version 4.0 [33,34]. 

All the tested compounds showed TD_50_ values better than **IDK**, and the values ranged from 6.036 to 431.948 g/kg body. Furthermore, compounds **27, 29, 30**, and **31** showed rat maximum tolerated dose values higher than that of **IDK**, and the values range is from 0.190 to 0.210 g/kg body weight. Conversely, the rest of the compounds showed maximum tolerated doses ranging from 0.035 to 0.115 g/kg that were lower than that of **IDK**. Compounds **34, 35**, and **65** revealed rat oral LD_50_ values of 2.364, 5.598, and 5.308, respectively, which were higher than that of **IDK** (2.626 g/kg body weight). On the other hand, the rest of the compounds showed oral LD_50_ values ranging from 0.230 to 2.223 g/kg body weight, which were lower than that of **IDK**. Additionally, all compounds were estimated to be non-toxic against the developmental toxicity potential model except compound **34**. Moreover, compounds **26, 27, 34**, and **72** showed LOAEL values ranging from 0.053 to 0.088 g/kg body weight whereas **IDK** exhibited 0.049 g/kg body weight. Finally, all the elected compounds showed no irritancy in the skin irritancy model as shown in Table S2 (Supplementary Data).

### 2.4. Molecular Docking Studies

Molecular docking was performed using Molecular Operating Environment (MOE) [35] to recognize the binding modes and interactions with the crucial amino acids [36,37,38,39,40,41]. Nine compounds (**11**, **26**, **27**, **29**, **30**, **31**, **35**, **65**, and **72**) displayed good computational ADMET and toxicity profiles. They were subjected to further docking studies. Such studies were carried out for the tested candidates to inspect their binding free energies (∆G) and binding modes against BMP-2-inducible kinase (PDB ID: 5I3R) using IDK as a reference (Table 3).

At first, the docking procedure was validated and the RMSD value was **1.05**, which indicated the validity of the docking process (Figure S2) (See Supplementary data).

**Figure 4:**
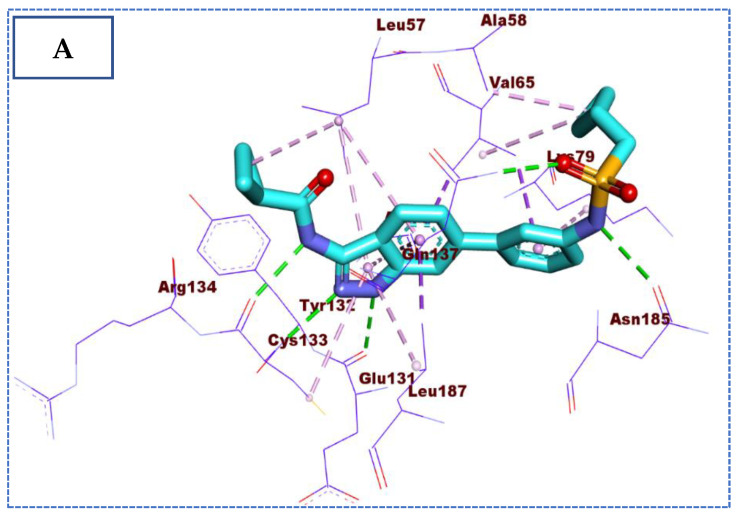

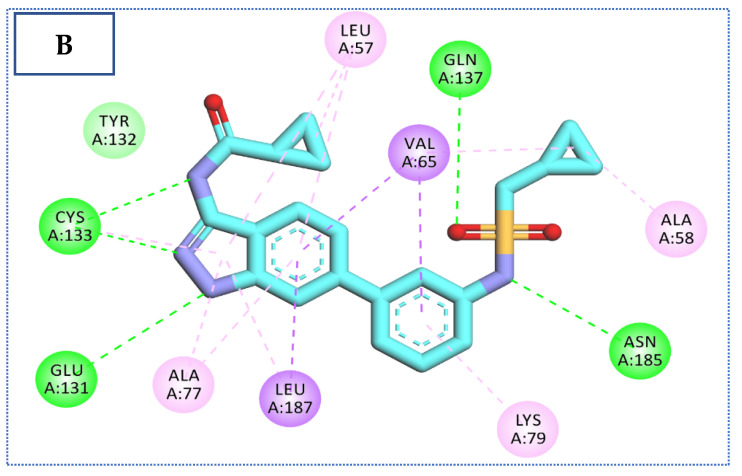
(**A**) The 3D and (**B**) 2D binding of co-crystallized ligand in the active site of BMP-2-inducible kinase (hydrogen bonds = green dashed lines, pi-pi interactions = purple dashed lines, and pi-alkyl interactions = light pink dashed lines).

**IDK** orientation with the amino acids of the pocket has been studied and displayed in Figure 4. The proposed binding pattern revealed an affinity value of −22.72 Kcal/mol. The interaction between **IDK** and BMP-2-inducible kinase is stabilized through five hydrogen bonds and a series of hydrophobic interactions. The N-(1H-indazol-3-yl)cyclopropanecarboxamide moiety forms three hydrogen bonds with the two crucial amino acids, Cys133 and Glu131, and nine hydrophobic interactions with Cys133, Leu57, Leu187, Val65, and Ala77. Moreover, 1-cyclopropyl-N-phenylmethanesulfonamide moiety was buried in the active pocket of BMP-2-inducible kinase through the formation of two hydrogen bonds with Gln137 and Asn185 and four hydrophobic interactions with Val65, Ala58, and Lys79 (Figure 4).

**Figure 5:**
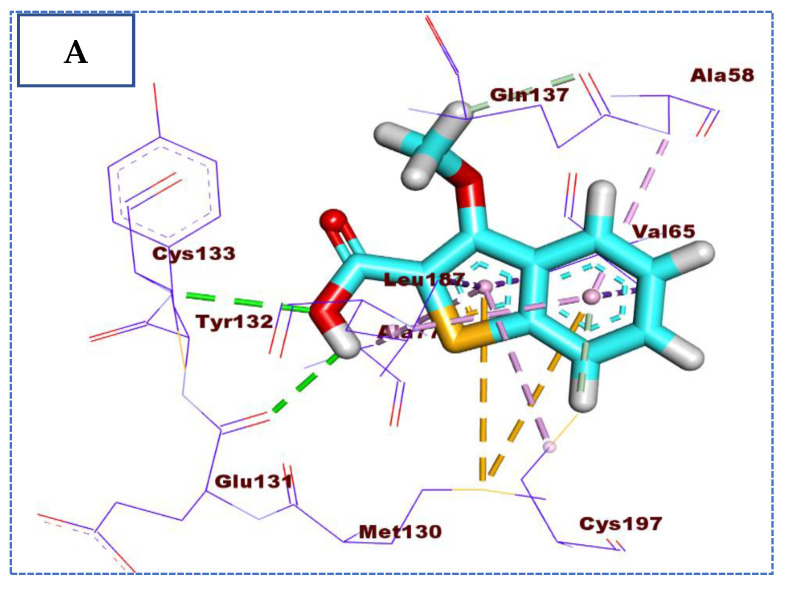

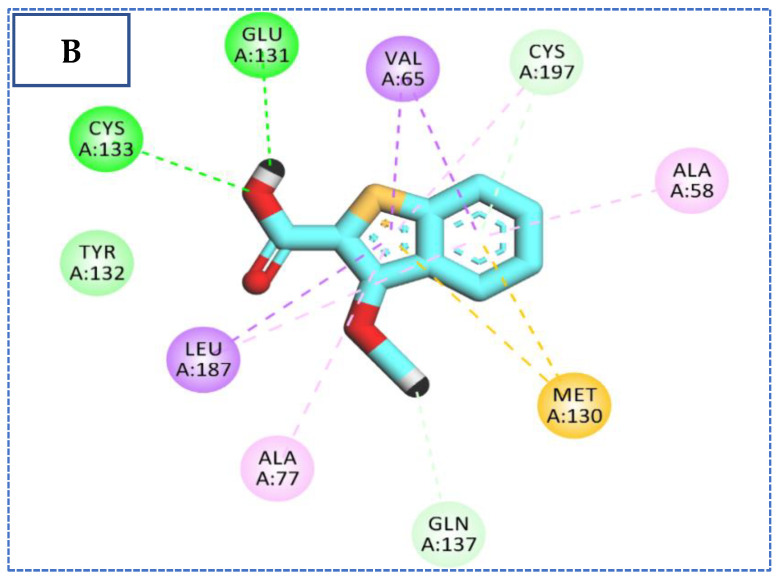
(**A**) The 3D and (**B**) 2D binding of **26** in the active site of BMP-2-inducible kinase (hydrogen bonds = green dashed lines, electrostatic interactions = orange dashed lines, pi-pi interactions = purple dashed lines, and pi-alkyl interactions = light pink dashed lines).

Compound **26** (affinity value of −22.90 Kcal/mol) combined with the receptor protein in a manner similar to that of IDK. The 3-methoxybenzo[*b*]thiophene moiety formed two hydrogen bonds with Cys197 and Gln137, in addition to seven hydrophobic interactions with Val65, Cys197, Ala58, Ala77, and Leu187, and two pi-sulfur interactions with Met130. Furthermore, the 2-carboxylic acid moiety formed two hydrogen bonds with the two essential amino acids, Cys133 and Glu131 (Figure 5).

**Figure 6:**
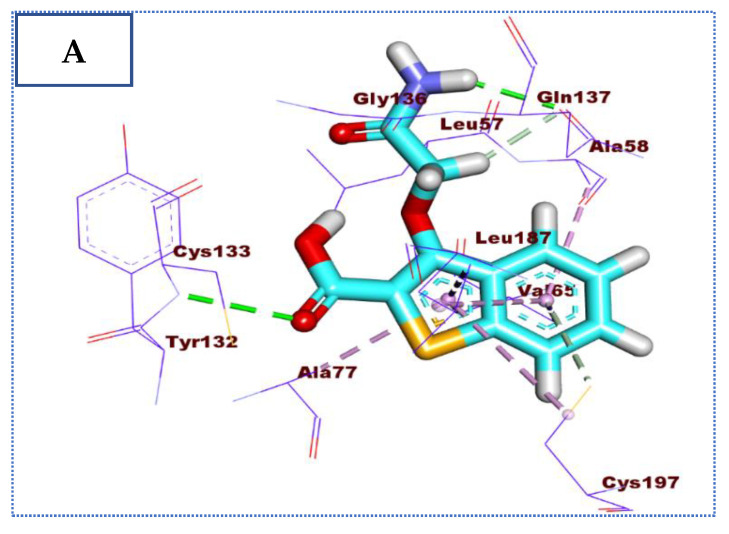

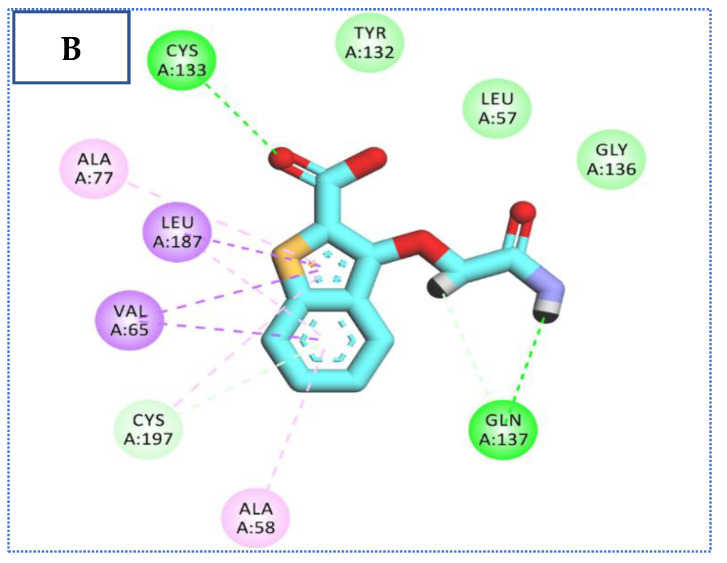
(**A**) The 3D and (**B**) 2D binding of **27** in the active site of BMP-2-inducible kinase (hydrogen bonds = green dashed lines, pi-pi interactions = purple dashed lines, and pi-alkyl interactions = light pink dashed lines).

Docking simulation of compound **27** revealed that it has a good fitting into the enzyme active site with a docking score of −23.34 Kcal/mol. The benzo[b]thiophene-2-carboxylic acid moiety formed two hydrogen bonds with Cys133 and Cys197. In addition, it formed seven pi-alkyl and pi-sigma interactions with Ala77, Val65, Cys197, Ala58, and Leu187. Furthermore, the 2-amino-2-oxoethoxy moiety formed two hydrogen bonds with Gln137 (Figure 6).

**Figure 7:**
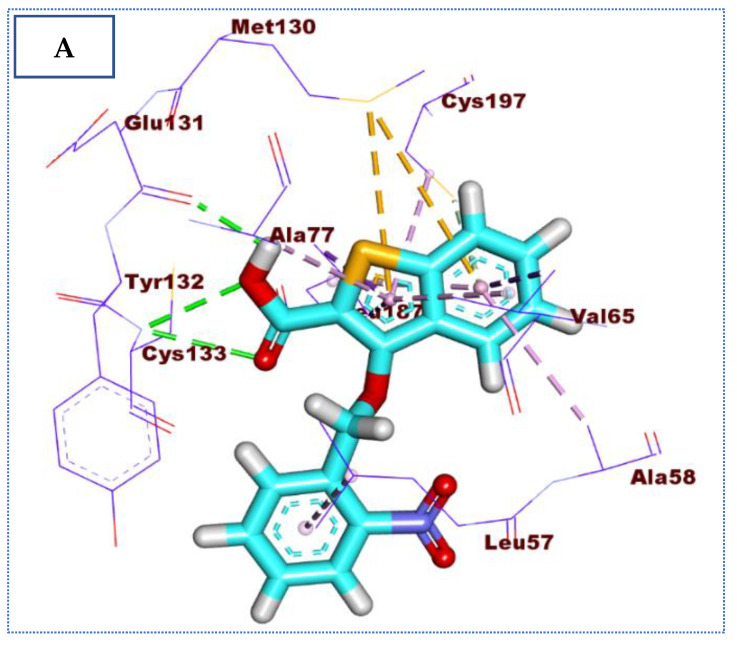

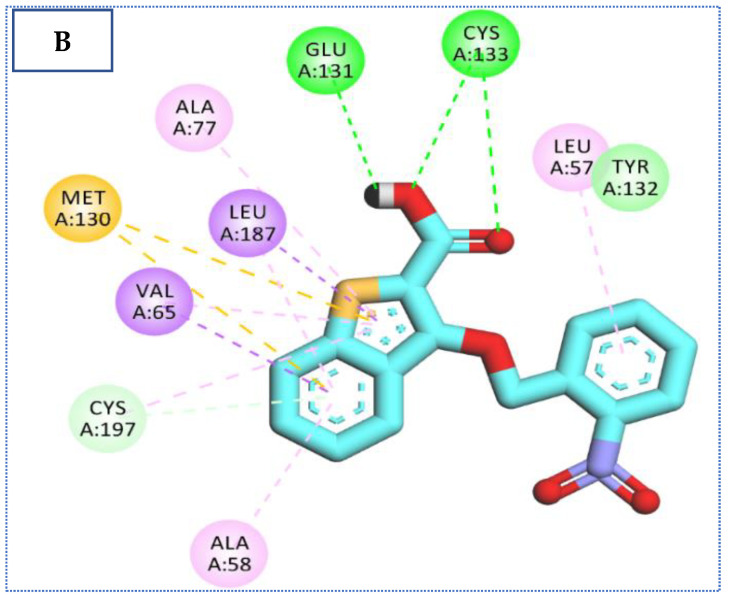
(**A**) The 3D and (**B**) 2D binding of **31** in the active site of BMP-2-inducible kinase (hydrogen bonds = green dashed lines, electrostatic interactions = orange dashed lines, pi-pi interactions = purple dashed lines, and pi-alkyl interactions = light pink dashed lines).

Interestingly, the docking result of compound **31** (affinity value of −22.22 Kcal/mol) is almost like that of IDK. The 3-ethoxybenzo[*b*]thiophene-2-carboxylic acid moiety formed three hydrogen bonds with Cys133 and Glu131. In addition, it is involved in binding with the receptor through the formation of seven hydrophobic interactions with Ala77, Ala58, Cys197, Leu187, and Val65, in addition to two pi-sulfur interactions with Met130. On the other hand, the 2-nitrobenzene moiety formed one pi-pi interaction with Leu57 (Figure 7).

**Figure 8:**
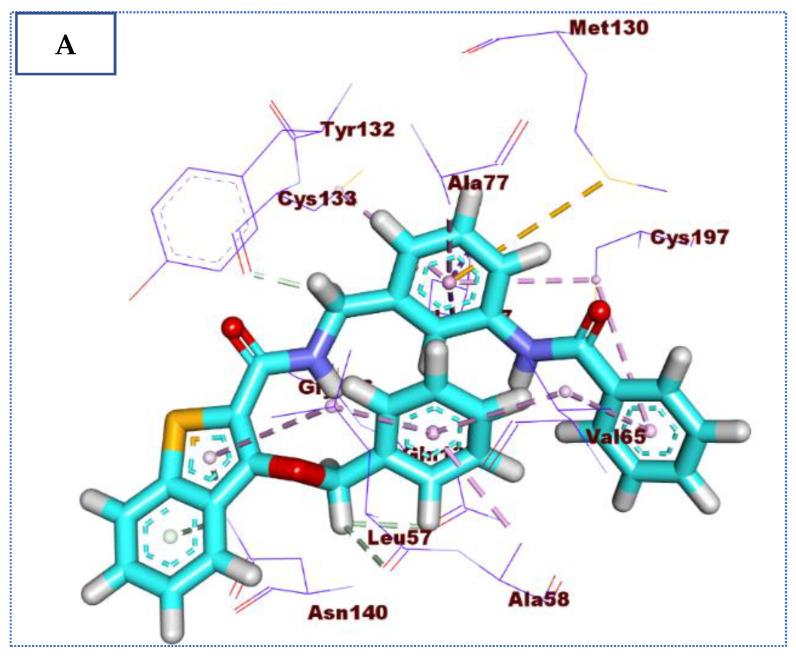

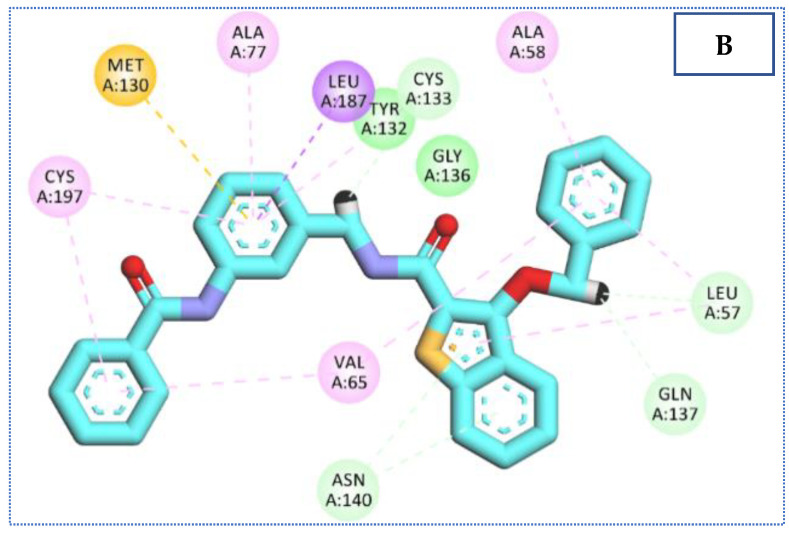
(**A**) The 3D and (**B**) 2D binding of **35** in the active site of BMP-2-inducible kinase (hydrogen bonds = green dashed lines, electrostatic interactions = orange dashed lines, pi-pi interactions = purple dashed lines, and pi-alkyl interactions = light pink dashed lines).

Molecular docking results of compound **35** revealed an affinity value of −24.25 Kcal/mol. The results showed formation of five hydrogen-bonding interactions, ten hydrophobic interactions, in addition to one electrostatic interaction, as observed in Figure 8.

**Figure 9:**
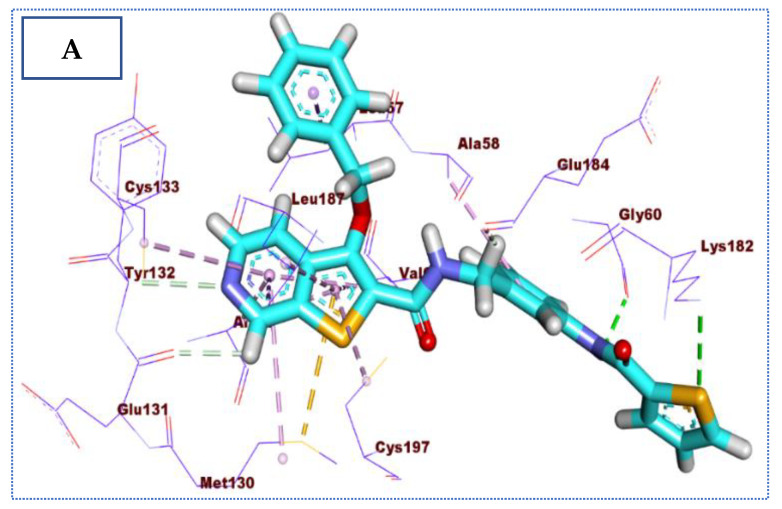

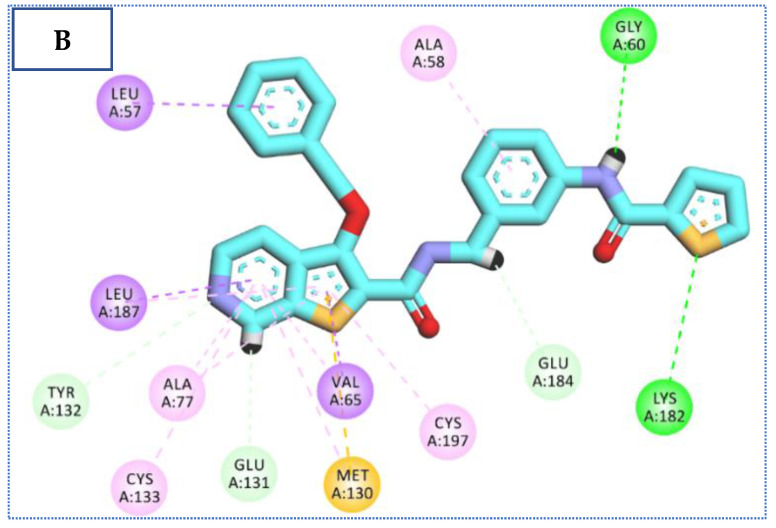
(**A**) The 3D and (**B**) 2D binding of **72** in the active site of BMP-2-inducible kinase (hydrogen bonds = green dashed lines, electrostatic interactions = orange dashed lines, pi-pi interactions = purple dashed lines, and pi-alkyl interactions = light pink dashed lines).

The proposed binding mode of compound **72** revealed an affinity value of −24.22 Kcal/mol. The 3-(benzyloxy) thieno[2,3-*c*]pyridine-2-carboxamide moiety formed ten hydrophobic interactions with Cys133, Ala77, Leu57, Leu187, and Val65. Likewise, it formed two hydrogen bonds with Glu131 and Tyr132, in addition to one pi-sulfur interaction with Met130. Moreover, the *N*-(3-(thiophene-2-carboxamido)benzyl) moiety formed three extra hydrogen bonds with Glu184, Gly60, and Lys182 and one pi-pi interaction with Ala58 (Figure 9).

### 2.5. Further Investigation against Protein Kinases

**Figure 10:**
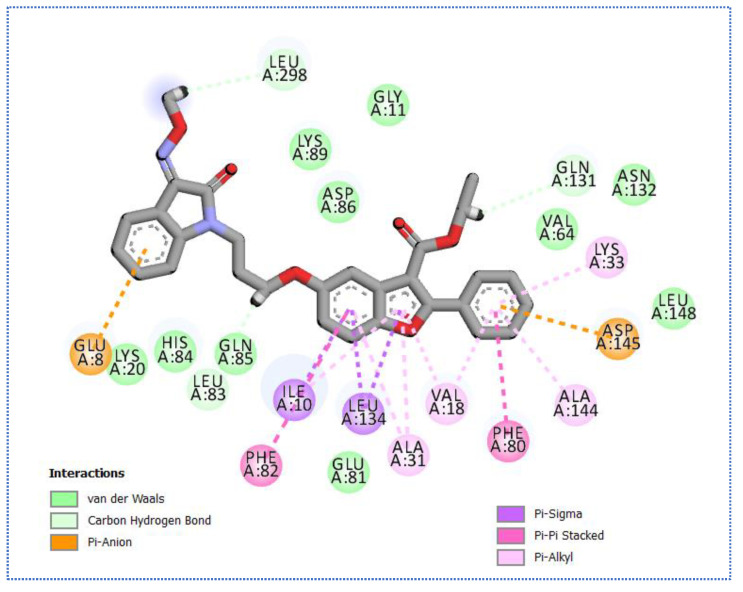
The 2D binding of compound **11** with Cell division protein kinase 2 (PDB ID: 3lfn).

**Figure 11:**
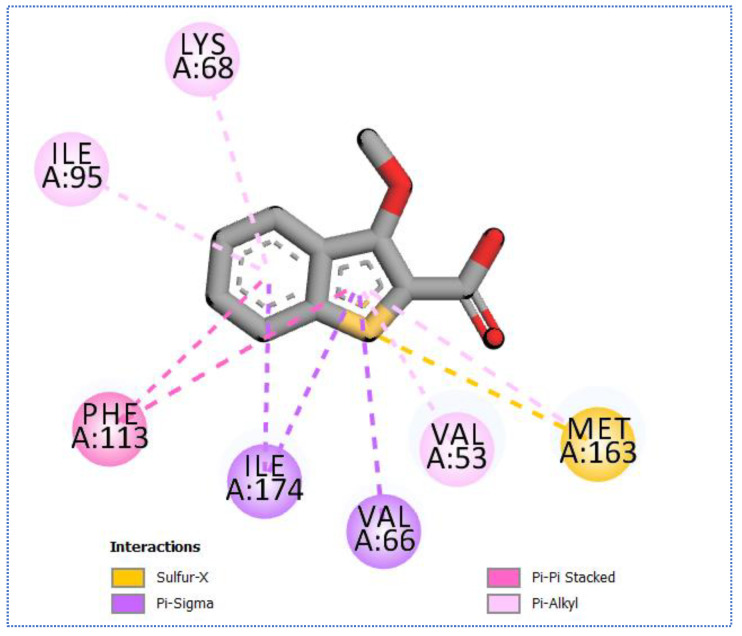
The 2D binding of compound **26** with Casein kinase II subunit alpha (PDB ID: 5csp).

**Figure 12:**
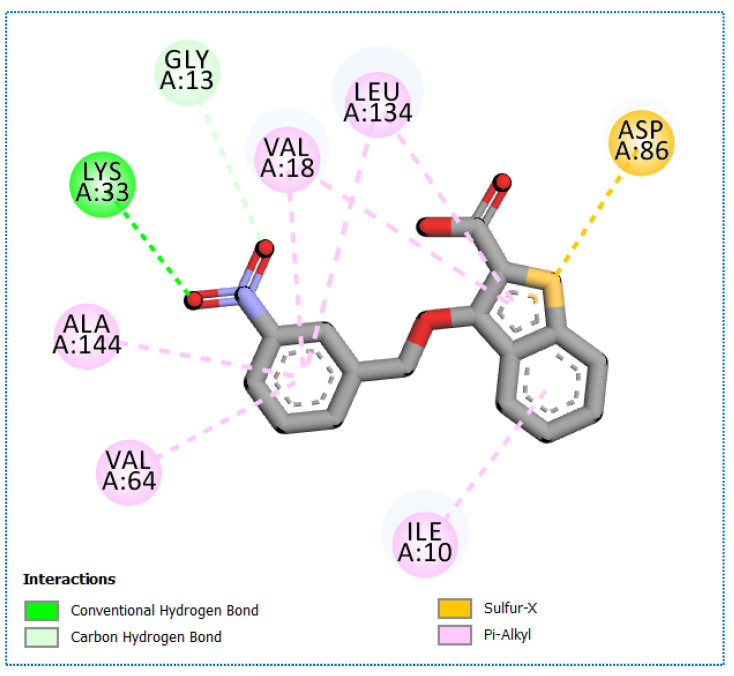
The 2D binding of compound **30** with cell division protein kinase 2 (PDB ID: 2wev).

The promising nine compounds will be subjected to further screening against kinases in general to explore their potential against these crucial proteins. Compound **11** was revealed to target 48 types of kinases (Supplementary Data) with a binding affinity ranging from −4.694 to −10.033 Kcal/mol. Compound **11** showed a ligand similarity score of 0.428 with Cell division protein kinase 2 ligand (A27). Moreover, it showed a binding similarity score of more than 66% with a docking score equal to −9.463 Kcal/mol. Figure 10 illustrates the mode and interactions of compound **11** with Cell division protein kinase 2. Compound **26** (Figure 11) revealed good potential to target three types of kinases, Casein kinase II subunit alpha, Proto-oncogene serine/threonine-protein kinase pim-1, and Serine/threonine-protein kinase pim-1 with a ligand similarity score of more than 40% and a binding affinity range from −6.4 to −7.00 Kcal/mol. Compound **27** showed the ability to target seven types of kinases with a ligand similarity score of more than 40% and a binding affinity range from −6.4 to −6.7 Kcal/mol. The best binding affinity was assigned for Serine/threonine-protein kinase pim-1. Furthermore, compound **27** revealed a good potential to target seven kinases with ligand similarity of more than 40% and a binding affinity range from −6.7 to 8.7 Kcal/mol, and it has shown a ligand and binding similarity mode of more than 40% and a docking score energy equal to −8.7 Kcal/mol with Cyclin-dependent kinase 2. Compound **30** showed potential against nine types of protein kinases, and the best proposed potential was assigned for cell division protein kinase 2 with a binding affinity of −8.7 Kcal/mol. Figure 12 exhibits the binding mode and interactions of compound **30** with cell division protein kinase 2.

**Figure 13:**
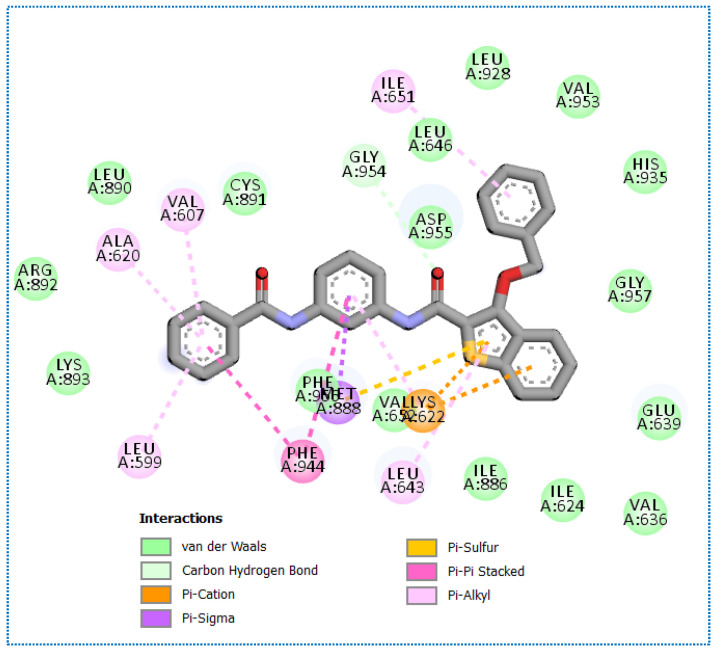
The 2D binding of compound **35** with Eukaryotic translation initiation factor 2-alpha kinase 3 (PDB ID: 4 × 7 h).

Compound **31** showed binding affinity equal to −8.53 against Cyclin-dependent kinase 2 and compound **35** showed more binding affinity towards protein kinase named Eukaryotic translation initiation factor 2-alpha kinase, and its binding affinity score is −11.812 Kcal/mol. Figure 13 show 2D binding mode of compound **35** with Eukaryotic translation initiation factor 2-alpha kinase-3. Compounds **65** and **72** revealed no potential to target kinases through the ligTMap tool.

## 3. Conclusions

Eighty-four benzothiophene and benzofuran derivatives were selected for in silico screening to reach promising BMP-2 inducible kinase inhibitors. Twenty-one compounds showed agreement with both Lipinski’s and Veber’s Rules. In silico ADMET studies revealed ten compounds (**11, 26, 27, 29, 30, 31, 34, 35, 65**, and **72**) with good pharmacokinetic profile. Additionally, in silico toxicity studies revealed nine compounds (**11, 26, 27, 29, 30, 31, 35, 65**, and **72**) with an acceptable toxicity profile. In addition, docking studies were carried out on BMP-2 and other kinases using the nine promising compounds. Molecular docking against BMP-2 inducible kinases demonstrated that eight compounds (**26**, **27**, **29**, **30**, **31**, **35**, **65**, and **72**) with significant binding affinity to the target displayed significant BMP-2 inducible kinase inhibitor properties. Finally, we can say that these organic molecules deserve further pre-clinical and clinical investigations as they may serve as anti-BMP-2 inducible kinases.

## 4. Experimental Section

### 4.1. Screening against Lipinski’s and Veber’s Rules

Lipinski and Veber descriptors were determined using Discovery studio 4.0. At first, the CHARMM force field was applied then the tested compounds were prepared and minimized according to the preparation of small molecule protocol. Then, the Lipinski and Veber descriptors protocol was applied to carry out these studies.

### 4.2. Computational ADMET Studies

ADMET descriptors (absorption, distribution, metabolism, excretion and toxicity) of the compounds were determined using Discovery studio 4.0. At first, the CHARMM force field was applied then the tested compounds were prepared and minimized according to the preparation of small molecule protocol. Then, ADMET descriptors protocol was applied to carry out these studies [42,43,44,45,46,47].

### 4.3. Computational Toxicity Studies

The toxicity parameters of the synthesized compounds were calculated using Discovery studio 4.0. IDK was used as a reference drug. At first, the CHARMM force field was applied then the compounds were prepared and minimized according to the preparation of small molecule protocol. Then, different parameters were calculated from the toxicity prediction (extensible) protocol [29,48].

### 4.4. Docking Studies

Crystallographic structure of tubulin [PDB ID: 5I3R, resolution: 2.40 Å] was retrieved from Protein Data Bank (http://www.pdb.org) and considered a target for docking simulation. The docking analysis was performed using MOE2014 software (Montreal, QC, Canada) to evaluate the free energy and binding modes of the synthesized compounds against BMP-2-inducible kinase. At first, the crystal structure of the target was prepared by removing water molecules and retaining the essential chain and the co-crystallized ligand (IDK). Then, the protein structure was protonated, and the hydrogen atoms were hidden. Next, the energy was minimized, and the binding pocket of the protein was defined.

The 2D structures of the tested compounds and reference ligand (IDK) were sketched using ChemBioDraw Ultra 14.0 and saved in MDL-SD format. Then, the saved files were opened using MOE and 3D structures were protonated. Next, energy minimization was applied. Before the docking process, validation of the docking protocol was carried out by running the simulation only using the co-crystallized ligand (IDK), which showed a low RMSD value. The molecular docking of the tested compounds was performed using a default protocol against the target receptor. In each case, 30 docked structures were generated using genetic algorithm searches, ASE was used for scoring, and forcefield (MMFF94X) for refinement. The ASE scoring function estimates the free energy of binding of the ligand from a given pose. The functional form is a sum of terms:

$$\Delta G=\;c+E_{flex}+\underset{h-bonds}{\sum }c_{HB\;}f_{HB}+\underset{m-lig}{\sum }c_{M\;}f_{M}+\underset{atoms\;i}{\sum }\Delta D_{i}$$

where *C* represents the average gain/loss of rotational and translational entropy; E*_flex_* is the energy due to the loss of flexibility of the ligand (calculated from ligand topology only); *F_HB_* measures geometric imperfections of hydrogen bonds and takes a value in [0,1]; *C_HB_* is the energy of an ideal hydrogen bond; *F_M_* measures geometric imperfections of metal ligations and takes a value in [0,1]; *C_M_* is the energy of an ideal metal ligation, and *D_i_* is the desolvation energy of atom *i*.

The output from MOE was further analyzed and visualized using Discovery Studio 4.0 software [49,50,51,52,53,54,55].

## Figures and Tables

**Table 1:** Physicochemical properties of the tested compounds passed Lipinski’s and Veber’s Rules.

Comp.	Lipinski’s Rule				Veber’s Rule	
	Num HD	Num HA	M Wt	AlogP	Num Rotatable Bonds	TPSA
**11**	0	8	498.527	4.994	10	90.57
**26**	1	3	208.234	2.596	2	74.77
**27**	3	5	251.258	1.45	4	117.86
**28**	1	3	284.33	4.18	4	74.77
**29**	1	6	329.327	4.074	5	120.59
**30**	1	6	329.327	4.074	5	120.59
**31**	1	6	329.327	4.074	5	120.59
**32**	1	3	298.356	4.666	4	74.77
**33**	2	5	416.492	4.543	6	95.67
**34**	4	7	459.517	3.396	8	138.76
**35**	2	5	492.588	6.126	8	95.67
**40**	3	4	388.482	4.595	6	92.59
**61**	3	6	481.566	5.511	8	111.46
**62**	2	6	482.55	5.521	8	108.81
**63**	2	5	498.616	6.08	8	123.91
**65**	2	6	493.576	4.976	8	108.56
**68**	2	5	430.519	4.462	7	95.67
**69**	2	5	444.545	5.129	8	95.67
**70**	2	5	458.572	5.585	9	95.67
**71**	2	5	458.572	5.591	8	95.67
**72**	2	6	499.604	4.929	8	136.8

**Table 2:** Predicted computational ADMET for the tested candidate.

Comp.	BBB Level ^a^	Solubility Level ^b^	Absorption Level ^c^	CYP2D6 Prediction ^d^	PPB Prediction ^e^
**11**	4	2	1	false	True
**26**	2	3	0	false	True
**27**	3	3	0	false	True
**28**	1	2	0	false	True
**29**	2	2	0	false	True
**30**	2	2	0	false	true
**31**	2	2	0	false	True
**32**	1	2	0	false	True
**33**	1	2	0	false	True
**34**	4	2	0	false	True
**35**	4	1	1	false	True
**40**	1	2	0	false	True
**61**	4	1	1	false	True
**62**	4	1	1	false	True
**63**	4	1	1	false	True
**65**	4	2	0	false	True
**68**	1	2	0	false	True
**69**	1	2	0	false	True
**70**	1	1	0	false	True
**71**	1	1	1	false	True
**72**	2	2	0	false	True
**IDK**	4	2	0	false	True

^a^ BBB level, blood–brain barrier level, 0 = very high, 1 = high, 2 = medium, 3 = low, 4 = very low. ^b^ Solubility level, 1 = very low, 2 = low, 3 = good, 4 = optimal. ^c^ Absorption level, 0 = good, 1 = moderate, 2 = poor, 3 = very poor. ^d^ CYP2D6, cytochrome P2D6, TRUE = inhibitor, FALSE = non inhibitor. ^e^ PBB, plasma protein binding, FALSE means less than 90%, TRUE means more than 90%.

**Table 3:** Calculated ∆G, amino acid interactions, and distances for the best five candidates and co-crystallized ligand (IDK) against BMP-2-inducible kinase (∆G in Kcal/mol).

Comp	∆G Kcal/mol	No. of H Bonds	Distance (Å)	Amino Acid Involved
**26**	−22.90	4	2.59	Glu131
			2.15	Cys133
			2.92	Cys197
			2.78	Gln137
**27**	−23.34	4	2.92	Cys133
			2.39	Cys197
			2.35, 2.44	Gln137
**31**	−22.22	4	2.24	Glu131
			3.08, 3.18	Cys133
			2.52	Cys197
**35**	−24.25	5	2.80, 2.72	Asn140
			2.33	Cys 133
			2.85	Gln 137
			2.86	Leu 57
**72**	−24.22	5	2.72	Glu131
			2.75	Glu184
			2.68	Lys 182
			2.76	Tyr 132
			2.65	Gly 60
**Co-crystallized ligand (IDK)**	−22.72	5	3.22	Glu131
			2.79, 2.83	Cys133
			3.36	Asn185
			2.89	Gln137

## Data Availability

Data are supplied in supplementary information files.

## Supplementary Materials

The following supporting information can be downloaded at: https://www.mdpi.com/article/10.3390/molecules27175571/s1. Figure S1: The chemical structures of the tested compounds; Table S2: Physicochemical properties of the tested compounds not passed Lipinski and Veber Rules; Figure S2: Validation of the docking process showed alignment of the co-crystallized pose and the re-docked pose of the same ligand inside BMP-2-inducible kinase; Supplementary data of toxicity study.
