# Supplementary material for: Discovery of Some Heterocyclic Molecules as Bone Morphogenetic Protein 2 (BMP-2)-Inducible Kinase Inhibitors: Virtual Screening, ADME Properties, and Molecular Docking Simulations
Source: Molecules. 2022 Aug 30;27(17):5571. doi: 10.3390/molecules27175571 (PMC9457949; doi:10.3390/molecules27175571)
Supplement: Supplementary file 1 [file molecules-27-05571-s001.zip › molecules-1842330-supplementary.pdf]

<u>Content</u>

1	**Figure SI-1: The chemical structures of the tested compounds.**
2	**Table S2**: Physicochemical properties of the tested compounds not passed Lipinski and Veber Rules
3	**Figure SI-2:** Validation of the docking process showed alignment of the co-crystallized pose and the re-docked pose of the same ligand inside BMP-2-inducible kinase.
4	**Supplementary data of toxicity study**

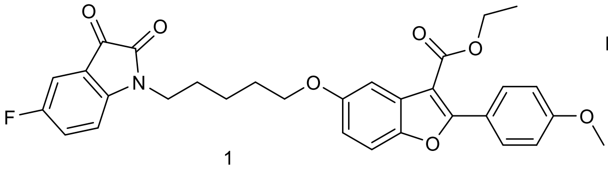

1

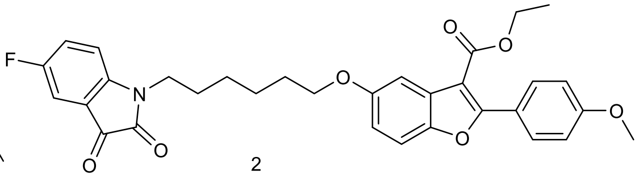

2

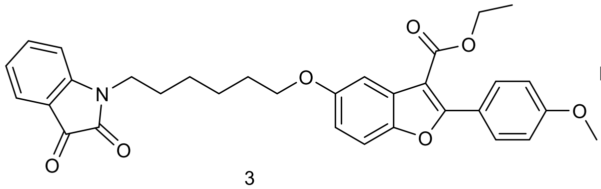

3

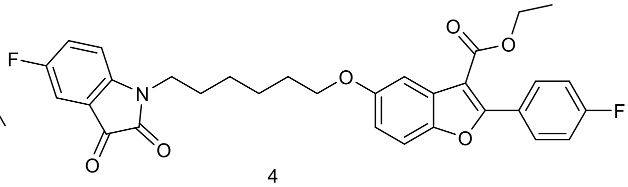

4

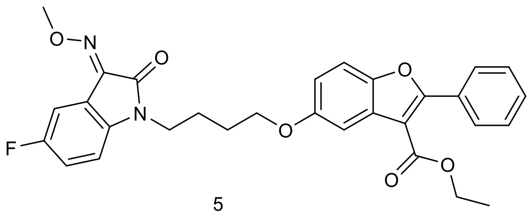

5

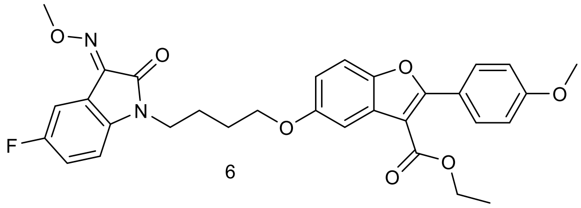

6

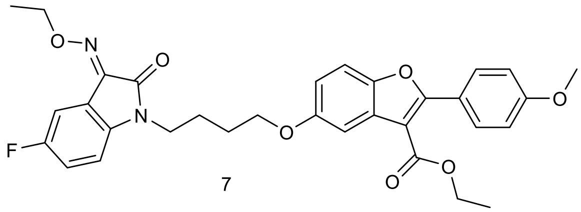

7

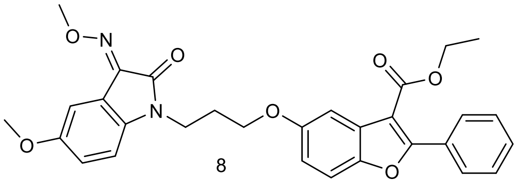

8

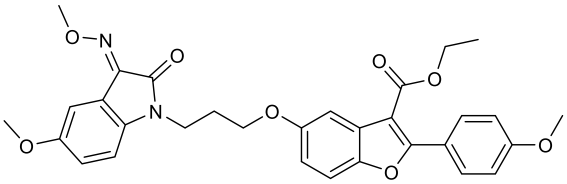

9

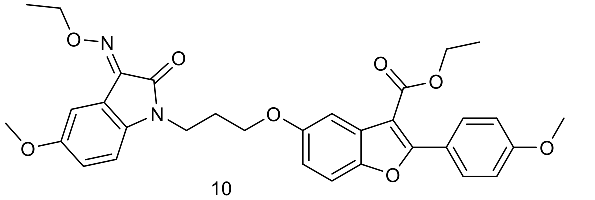

10

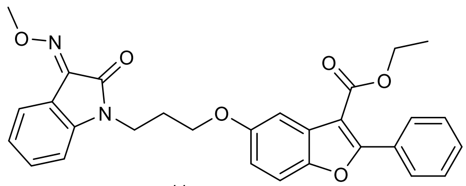

11

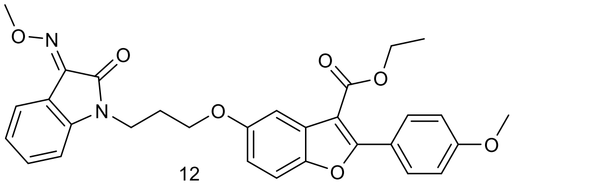

12

Figure SI-1: The chemical structures of the tested compounds.

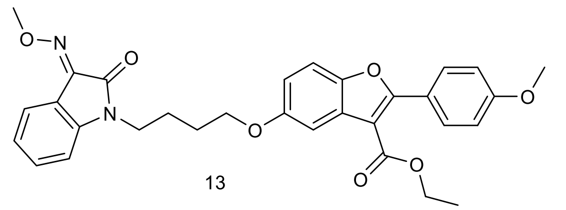

13

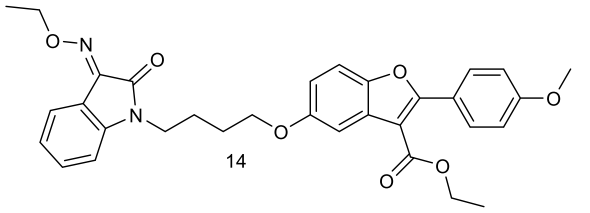

14

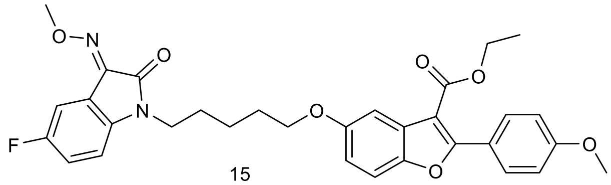

15

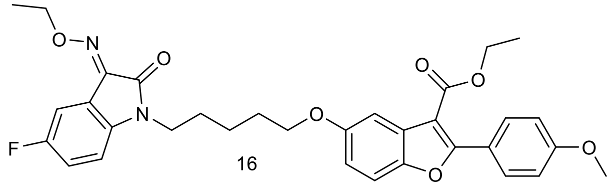

16

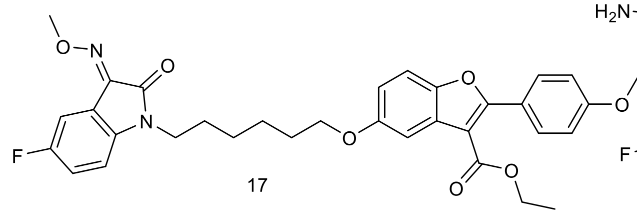

17

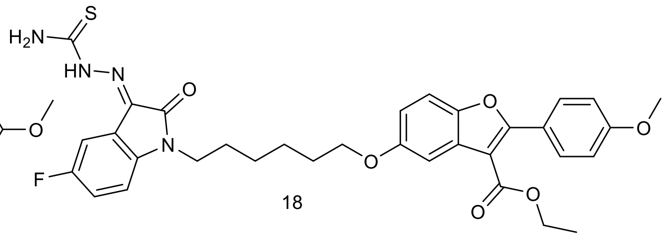

18

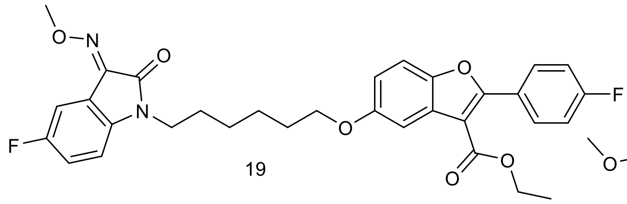

19

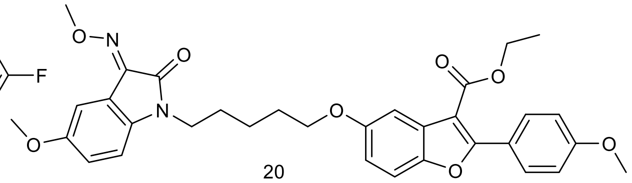

20

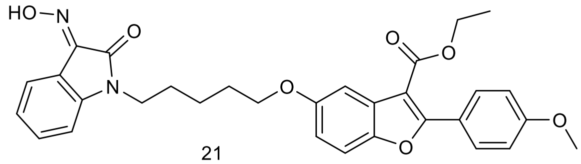

21

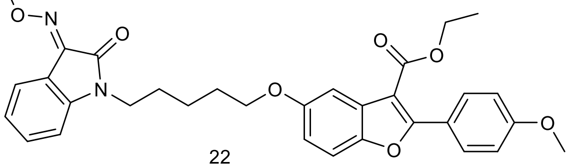

22

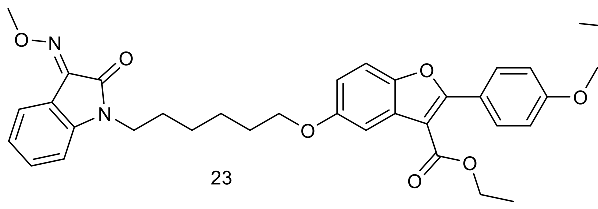

23

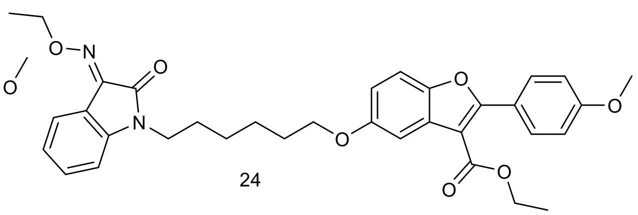

24

Figure SI-1: (continued) The chemical structures of the tested compounds.

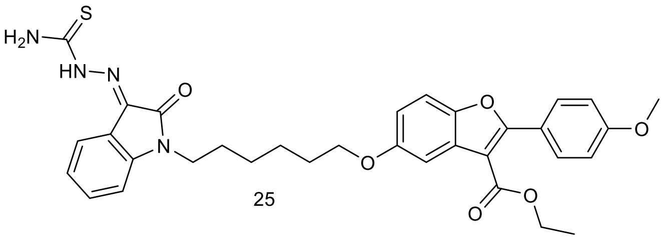

25

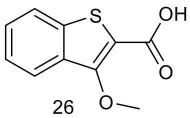

26

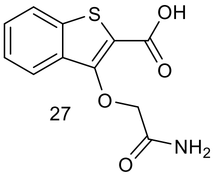

27

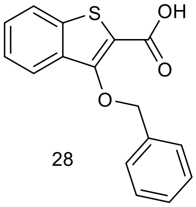

28

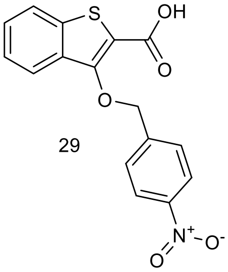

29

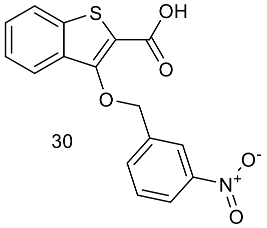

30

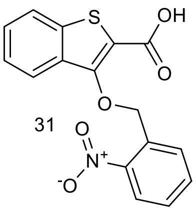

31

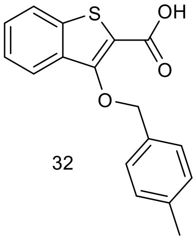

32

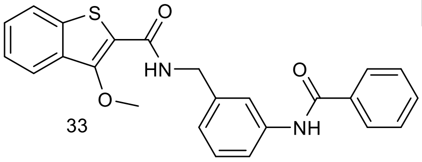

33

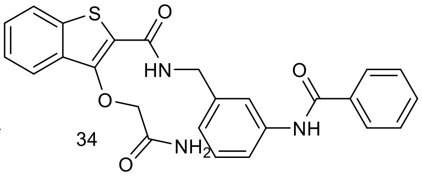

34

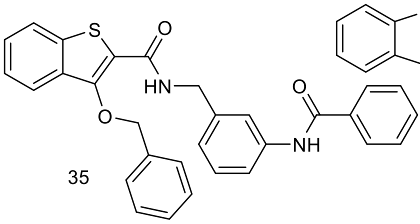

35

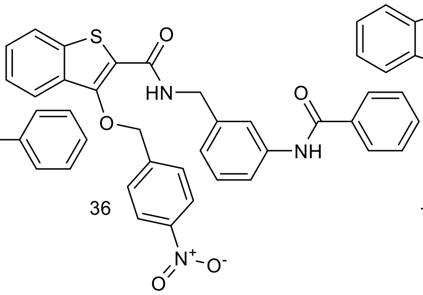

36

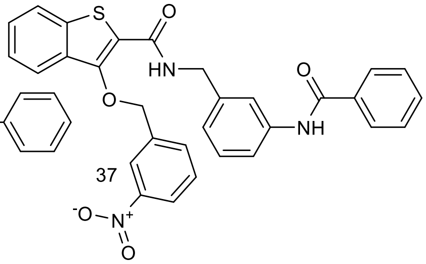

37

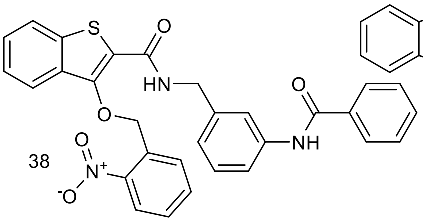

38

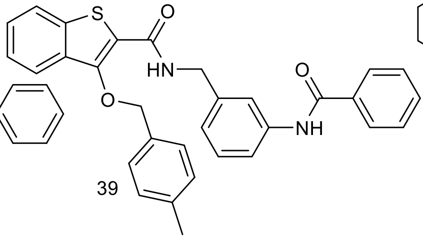

39

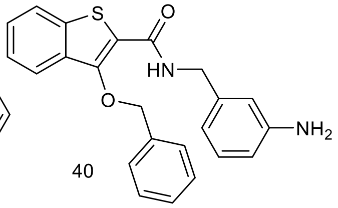

40

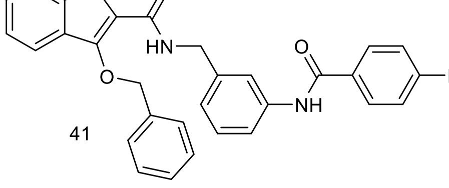

41

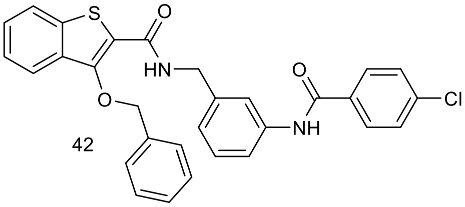

42

Figure SI-1: (continued) The chemical structures of the tested compounds.

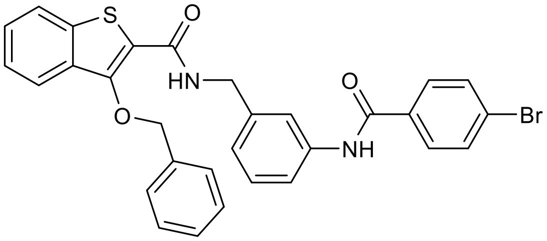

43

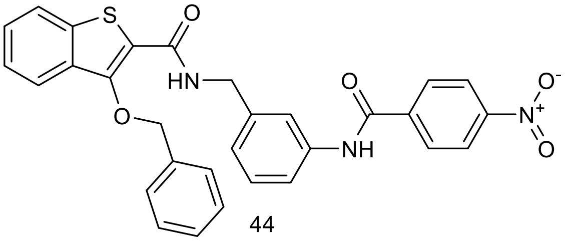

44

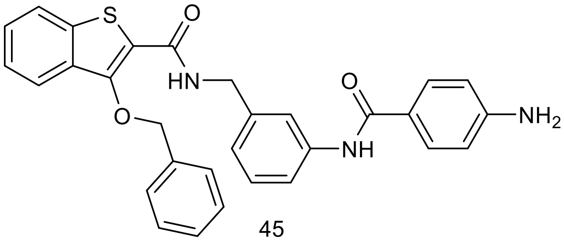

45

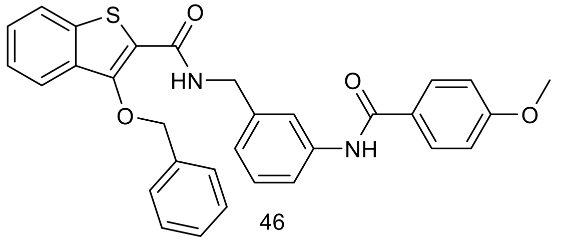

46

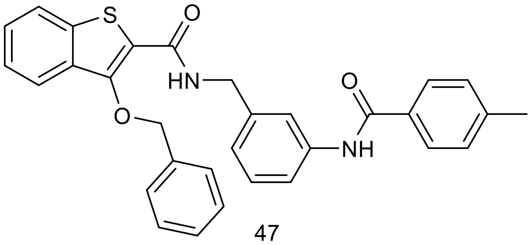

47

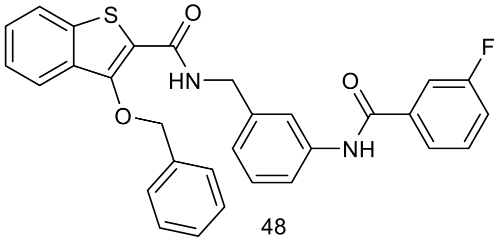

48

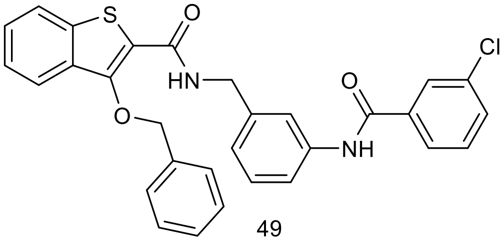

49

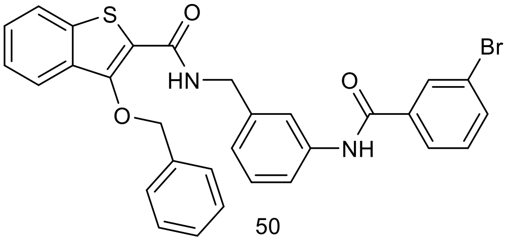

50

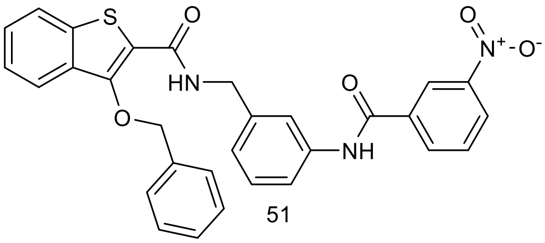

51

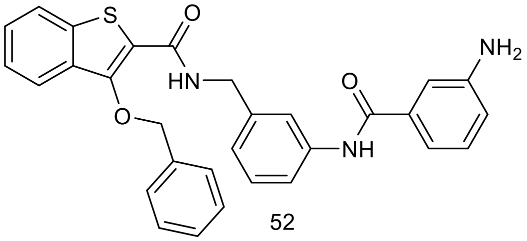

52

Figure SI-1: (continued) The chemical structures of the tested compounds.

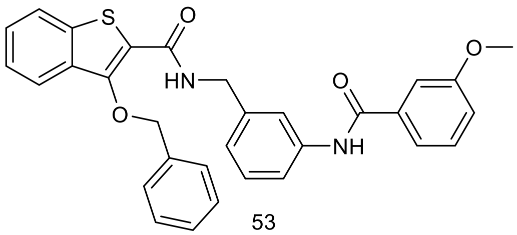

53

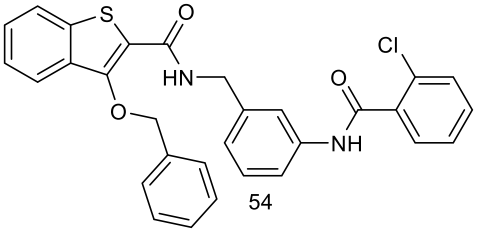

54

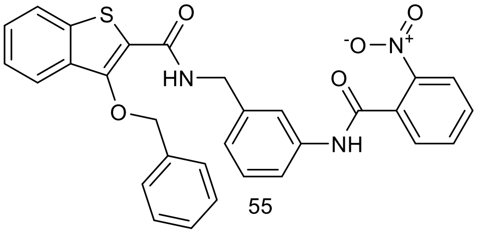

55

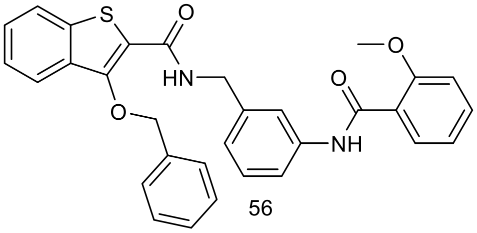

56

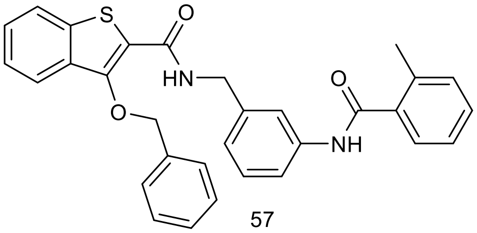

57

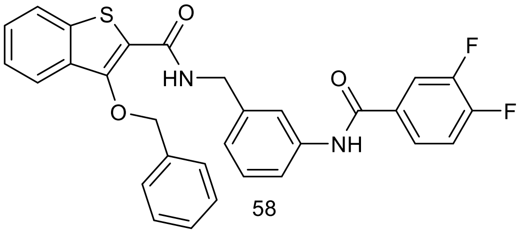

58

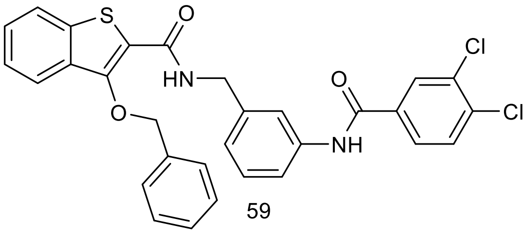

59

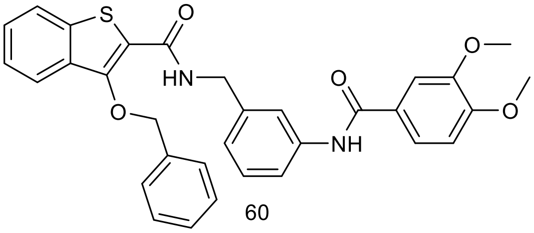

60

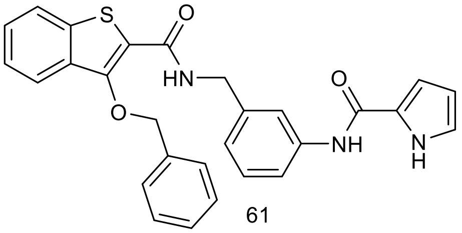

61

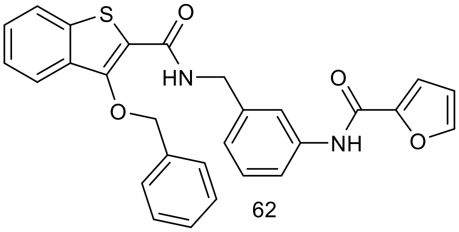

62

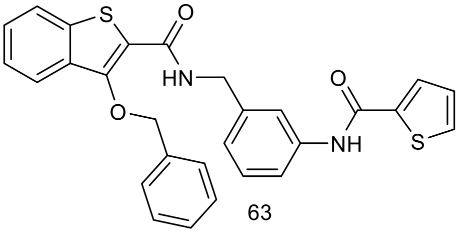

63

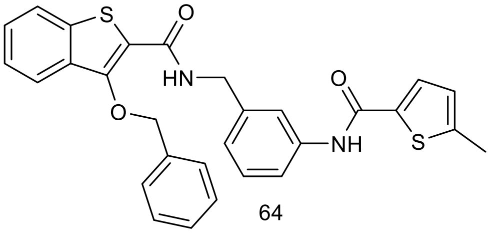

64

Figure SI-1: (continued) The chemical structures of the tested compounds.

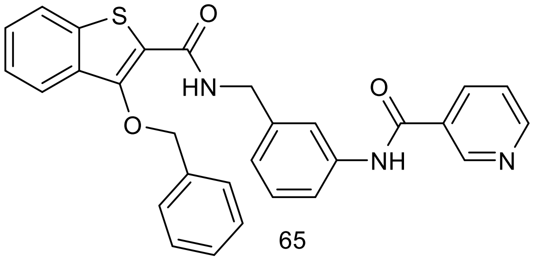

65

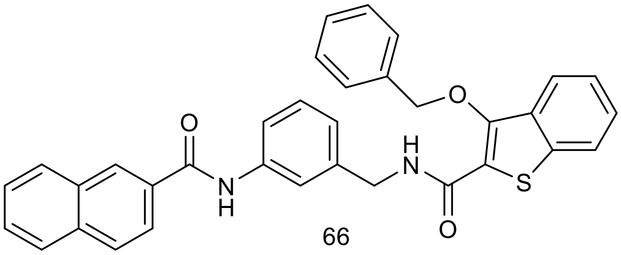

66

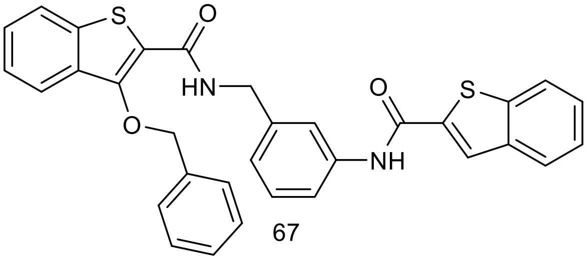

67

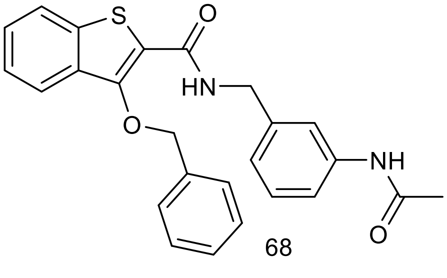

68

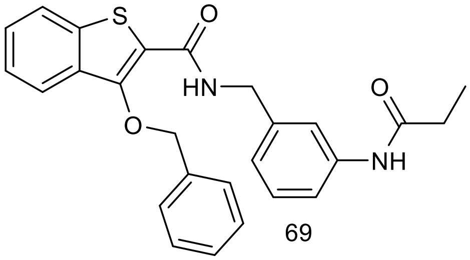

69

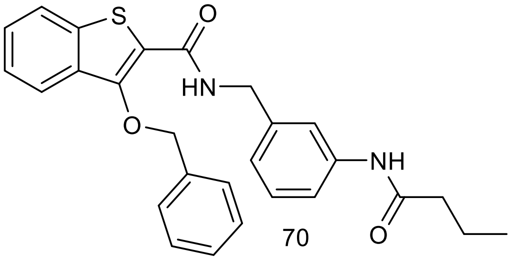

70

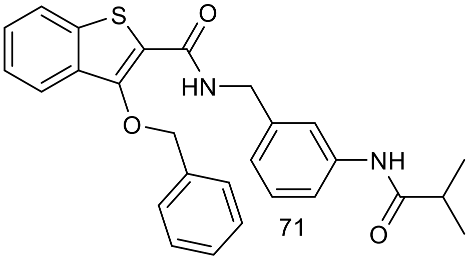

71

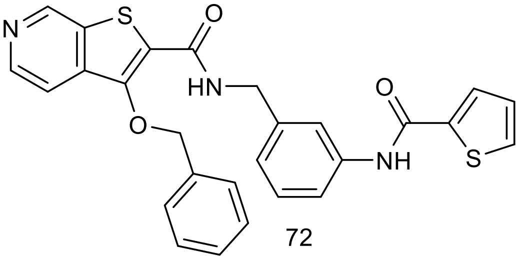

72

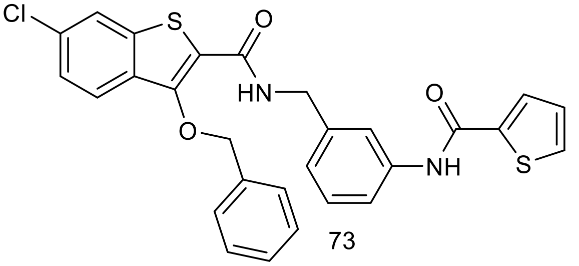

73

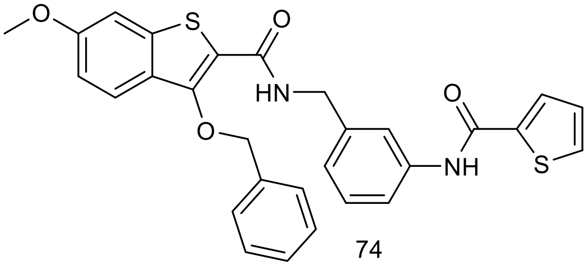

74

Figure SI-1: (continued) The chemical structures of the tested compounds.

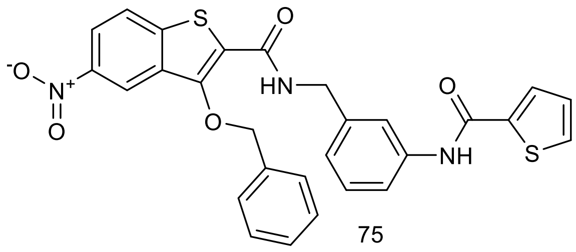

75

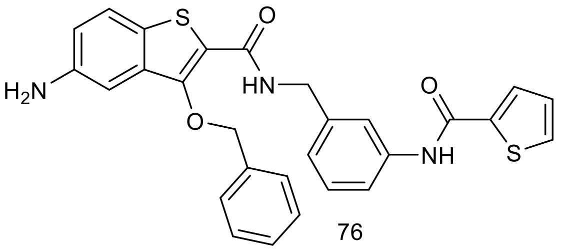

76

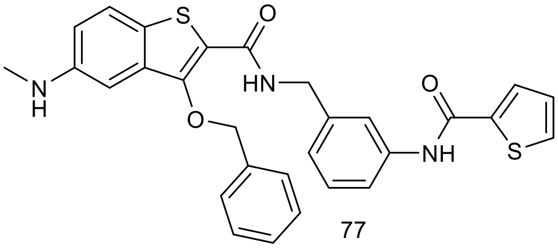

77

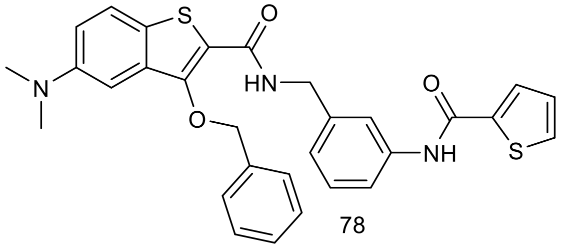

78

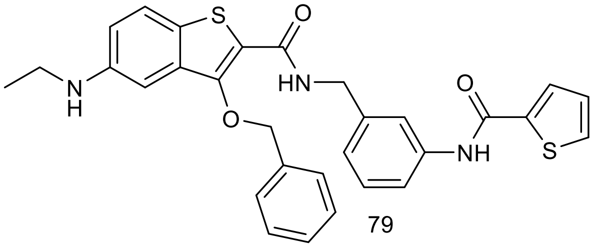

79

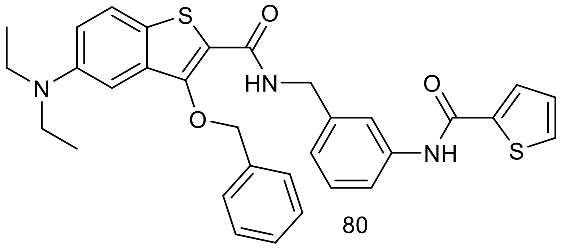

80

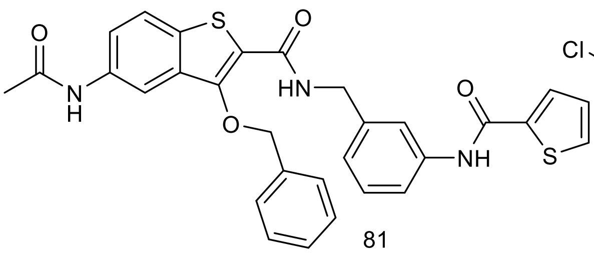

81

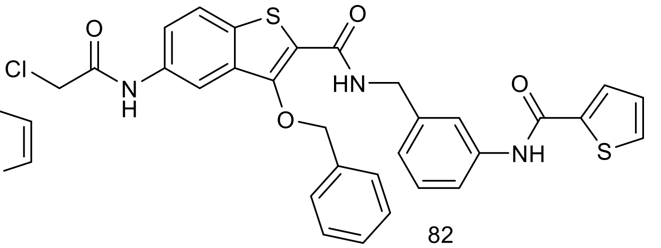

82

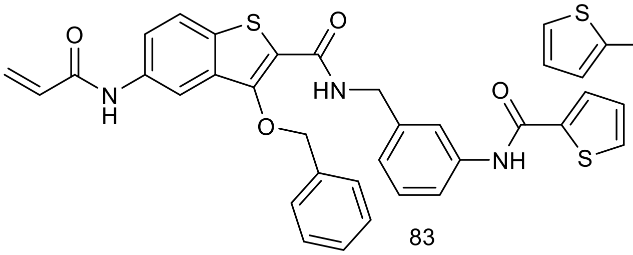

83

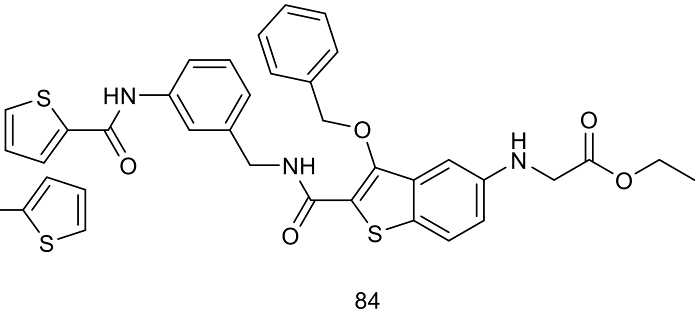

84

Figure SI-1: (continued) The chemical structures of the tested compounds.

Table S2: Physicochemical properties of the tested compounds not passed Lipinski and Veber Rules

	Lipinski Rules				Veber Rules	
comp.	Num HD	Num HA	M Wt	AlogP	Num Rotatable Bonds	MPSA
1	0	8	545.555	6.113	12	95.28
2	0	8	559.582	6.57	13	95.28
3	0	8	541.591	6.364	13	95.28
4	0	7	547.546	6.792	12	86.05
5	0	8	530.544	5.78	11	90.57
6	0	9	560.57	5.764	12	99.8
7	0	9	574.596	6.112	13	99.8
8	0	9	528.553	4.978	11	99.8
9	0	10	558.578	4.962	12	109.03
10	0	10	572.605	5.311	13	109.03
12	0	9	528.553	4.978	11	99.8
13	0	9	542.579	5.558	12	99.8
14	0	9	556.606	5.907	13	99.8
15	0	9	574.596	6.22	13	99.8
16	0	9	588.623	6.569	14	99.8
17	0	9	588.623	6.676	14	99.8
18	3	10	632.702	6.78	15	160.71
19	0	8	576.587	6.898	13	90.57
20	0	10	586.632	5.998	14	109.03
21	1	9	542.579	5.975	12	110.8
22	0	9	556.606	6.014	13	99.8
23	0	9	570.632	6.471	14	99.8
24	0	9	584.659	6.819	15	99.8
25	3	10	614.711	6.575	15	160.71
36	2	8	537.586	6.021	9	141.48
37	2	8	537.586	6.021	9	141.48
38	2	8	537.586	6.021	9	141.48
39	2	5	506.615	6.612	8	95.67
41	2	5	510.579	6.332	8	95.67
42	2	5	527.033	6.791	8	95.67
43	2	5	571.484	6.875	8	95.67
44	2	8	537.586	6.021	9	141.49
45	4	6	507.603	5.38	8	121.69
46	2	6	522.614	6.11	9	104.9
47	2	5	506.615	6.612	8	95.67

48	2	5	510.579	6.332	8	95.67
49	2	5	527.033	6.791	8	95.67
50	2	5	571.484	6.875	8	95.67
51	2	8	537.586	6.021	9	141.49
52	4	6	507.603	5.38	8	121.69
53	2	6	522.614	6.11	9	104.9
54	2	5	527.033	6.791	8	95.67
55	2	8	537.586	6.021	9	141.49
56	2	6	522.614	6.11	9	104.9
57	2	5	506.615	6.612	8	95.67
58	2	5	528.569	6.537	8	95.67
59	2	5	561.478	7.455	8	95.67
60	2	7	552.64	6.093	10	114.13
64	2	5	512.642	6.225	8	123.91
66	2	5	542.647	7.035	8	95.67
67	2	5	548.675	7.279	8	123.91
73	2	5	533.061	6.744	8	123.91
74	2	6	528.642	6.063	9	133.14
75	2	8	543.613	5.974	9	169.73
76	4	6	513.631	5.333	8	149.93
77	3	6	527.657	5.888	9	135.94
78	2	6	541.684	6.242	9	127.15
79	3	6	541.684	6.237	10	135.94
80	2	6	569.737	6.939	11	127.15
81	3	7	555.667	5.2	9	153.01
82	3	7	590.112	5.732	10	153.01
83	3	7	567.678	5.897	10	153.01
84	3	8	599.72	5.944	13	162.23

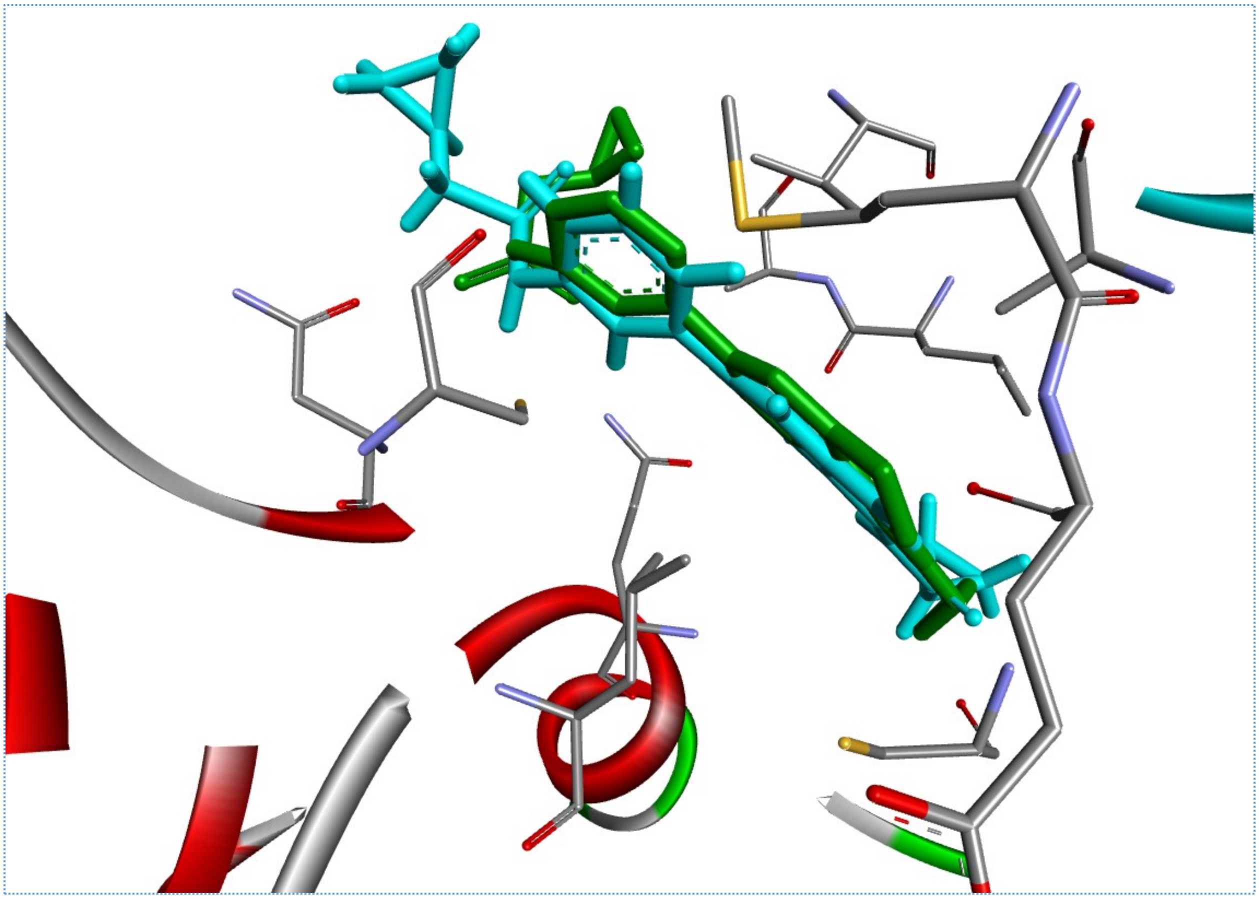

Figure S1-2 Validation of the docking process showed alignment of the co-crystallized pose (turquoise) and the re-docked pose (green) of the same ligand inside BMP-2-inducible kinase, PDB: 5I3R.

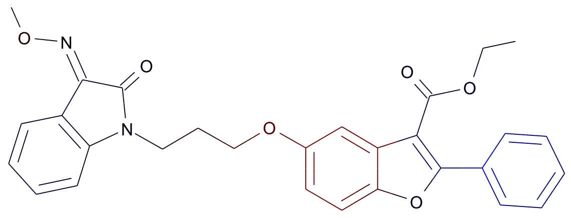

$C_{29}H_{26}N_2O_6$

Molecular Weight: 498.52653

ALogP: 4.994

Rotatable Bonds: 10

Acceptors: 6

Donors: 0

Model Prediction

Prediction: Non-Toxic

Probability: 0.490

Enrichment: 0.932

Bayesian Score: -1.580

Mahalanobis Distance: 12.016

Mahalanobis Distance p-value: 2.72e-005

Prediction: Positive if the Bayesian score is above the estimated best cutoff value from minimizing the false positive and false negative rate.
Probability: The esimated probability that the sample is in the positive category. This assumes that the Bayesian score follows a normal distribution and is different from the prediction using a cutoff.
Enrichment: An estimate of enrichment, that is, the increased likelihood (versus random) of this sample being in the category.
Bayesian Score: The standard Laplacian-modified Bayesian score.
Mahalanobis Distance: The Mahalanobis distance (MD) is the distance to the center of the training data. The larger the MD, the less trustworthy the prediction.
Mahalanobis Distance p-value: The p-value gives the fraction of training data with an MD greater than or equal to the one for the given sample, assuming normally distributed data. The smaller the p-value, the less trustworthy the prediciton. For highly non-normal X properties (e.g., fingerprints), the MD p-value is wildly inaccurate.

Structural Similar Compounds

Name	Nicardipine	Suxibuzone	Soforalcone
Structure	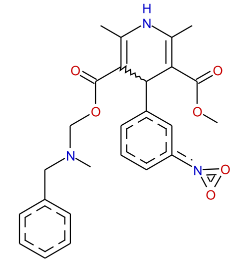	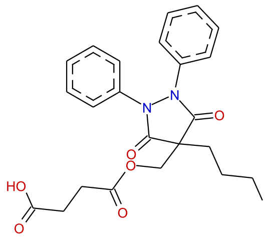	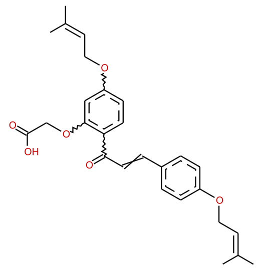
Actual Endpoint	Non-Toxic	Toxic	Toxic
Predicted Endpoint	Non-Toxic	Toxic	Toxic
Distance	0.649	0.649	0.658
Reference	Kiso to Rinsho 13:1149-1159; 1979	Oyo Yakuri 20:377-386; 1980	Oyo Yakuri 19:525-535; 1980

Model Applicability

Unknown features are fingerprint features in the query molecule, but not found in the training set.

1. OPS PC13 out of range. Value: -3.5488. Training min, max, SD, explained variance: -3.5435, 3.7718, 1.291, 0.0244.
2. OPS PC22 out of range. Value: -2.9499. Training min, max, SD, explained variance: -2.4788, 2.7503, 0.9196, 0.0124.

Feature Contribution

Top features for positive contribution

Fingerprint	Bit/Smiles	Feature Structure	Score	Toxic in training set
SCFP_6	1237755852	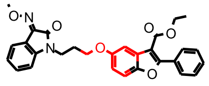 [*]CO[c]1:[cH]:[cH]:[*]:[c](:[*]):[cH]:1	0.453	8 out of 9

SCFP_6	-910601882	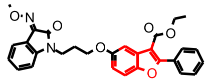 [*][c]1:o:[c]2:[cH]:[cH]:[*]:[cH]:[c]:2:[c]:1[*]	0.271	1 out of 1
SCFP_6	-1380805791	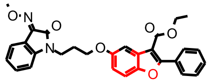 [*][c]1:[*]:[c]2:[*]:[*]:o:[c]:2:[cH]:[cH]:1	0.271	1 out of 1

Top Features for negative contribution

Fingerprint	Bit/Smiles	Feature Structure	Score	Toxic in training set
SCFP_6	1655112089	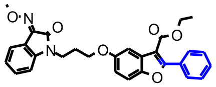 [*]:[c](:[*])[c]1:[cH]:[cH]:[cH]:[cH]:[cH]:1	-0.438	1 out of 4
SCFP_6	1683883894	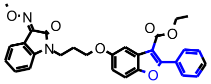 [*][c]1:[*]:[*]:o:[c]:1[c]2:[cH]:[cH]:[*]:[cH]:[cH]:2	-0.422	0 out of 1
SCFP_6	-1798255434	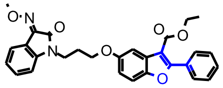 [*][c]1:[*]:[*]:o:[c]:1[c](:[*]):[*]	-0.422	0 out of 1

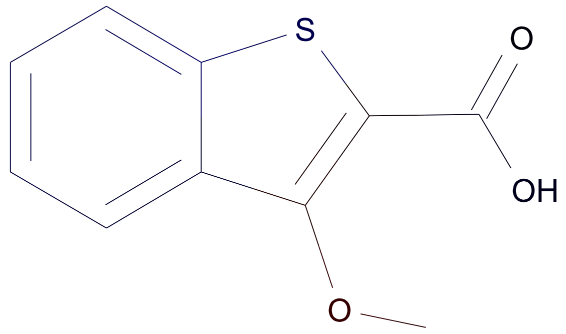

$C_{10}H_8O_3S$
Molecular Weight: 208.23372
ALogP: 2.596
Rotatable Bonds: 2
Acceptors: 3
Donors: 1

Model Prediction

Prediction: Non-Toxic
Probability: 0.491
Enrichment: 0.934
Bayesian Score: -1.548
Mahalanobis Distance: 9.632
Mahalanobis Distance p-value: 0.0509

Prediction: Positive if the Bayesian score is above the estimated best cutoff value from minimizing the false positive and false negative rate.
Probability: The esimated probability that the sample is in the positive category. This assumes that the Bayesian score follows a normal distribution and is different from the prediction using a cutoff.
Enrichment: An estimate of enrichment, that is, the increased likelihood (versus random) of this sample being in the category.
Bayesian Score: The standard Laplacian-modified Bayesian score.
Mahalanobis Distance: The Mahalanobis distance (MD) is the distance to the center of the training data. The larger the MD, the less trustworthy the prediction.
Mahalanobis Distance p-value: The p-value gives the fraction of training data with an MD greater than or equal to the one for the given sample, assuming normally distributed data. The smaller the p-value, the less trustworthy the prediciton. For highly non-normal X properties (e.g., fingerprints), the MD p-value is wildly inaccurate.

Structural Similar Compounds

Name	3;6-Dichloropicolinic Acid	Suprofen	Triclopyr
Structure	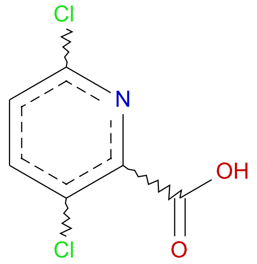	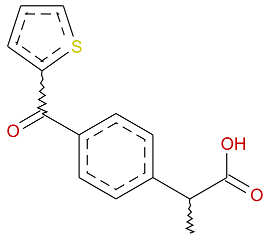	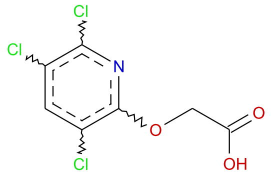
Actual Endpoint	Non-Toxic	Non-Toxic	Non-Toxic
Predicted Endpoint	Non-Toxic	Non-Toxic	Non-Toxic
Distance	0.547	0.578	0.602
Reference	Fundam Appl Toxicol 4:91-97; 1984	Oyo Yakuri 26:449-459; 1983	Fundam Appl Toxicol 4:872-882; 1984

Model Applicability

Unknown features are fingerprint features in the query molecule, but not found in the training set.

1. All properties and OPS components are within expected ranges.

Feature Contribution

Top features for positive contribution

Fingerprint	Bit/Smiles	Feature Structure	Score	Toxic in training set
SCFP_6	591469355	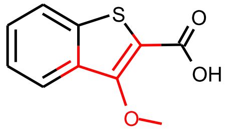 [*][c]1:[*]:[*]:[c](:[*]):[c]:1OC	0.411	10 out of 12

SCFP_6	136239834	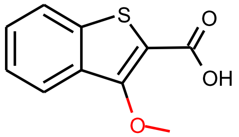<br>[*]OC	0.242	23 out of 34
SCFP_6	276283342	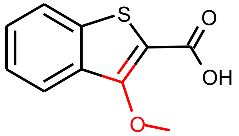<br>[*]CO[c](:[*]):[*]	0.196	20 out of 31

Top Features for negative contribution

Fingerprint	Bit/Smiles	Feature Structure	Score	Toxic in training set
SCFP_6	1653687455	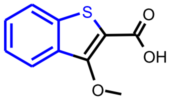<br>[*]1:[*]:[c]2:[cH]:[cH]:[cH]:[cH]:[c]:2:s:1	-0.422	0 out of 1
SCFP_6	2083824450	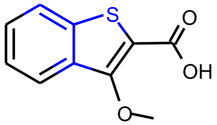<br>[*]:[cH]:[c]1:s:[*]:[*]:[c]:1:[*]	-0.422	0 out of 1
SCFP_6	-1376127683	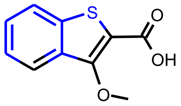<br>[*]1:[*]:[c]2:[*]:[cH]:[cH]:[cH]:[c]:2:s:1	-0.422	0 out of 1

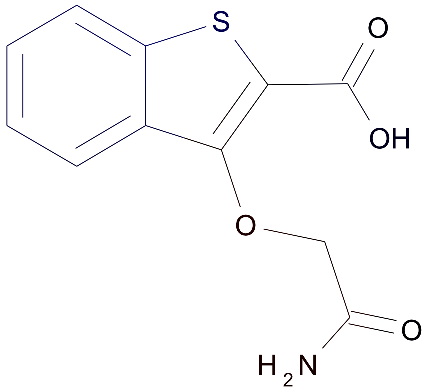

$C_{11}H_9NO_4S$
Molecular Weight: 251.25846
ALogP: 1.45
Rotatable Bonds: 4
Acceptors: 4
Donors: 2

Model Prediction

Prediction: Non-Toxic
Probability: 0.494
Enrichment: 0.939
Bayesian Score: -1.463
Mahalanobis Distance: 10.528
Mahalanobis Distance p-value: 0.00485

Prediction: Positive if the Bayesian score is above the estimated best cutoff value from minimizing the false positive and false negative rate.
Probability: The esimated probability that the sample is in the positive category. This assumes that the Bayesian score follows a normal distribution and is different from the prediction using a cutoff.
Enrichment: An estimate of enrichment, that is, the increased likelihood (versus random) of this sample being in the category.
Bayesian Score: The standard Laplacian-modified Bayesian score.
Mahalanobis Distance: The Mahalanobis distance (MD) is the distance to the center of the training data. The larger the MD, the less trustworthy the prediction.
Mahalanobis Distance p-value: The p-value gives the fraction of training data with an MD greater than or equal to the one for the given sample, assuming normally distributed data. The smaller the p-value, the less trustworthy the prediciton. For highly non-normal X properties (e.g., fingerprints), the MD p-value is wildly inaccurate.

Structural Similar Compounds

Name	Caffeic Acid	6-Chloro-4-nitro-2-aminophenol	Dinoseb
Structure	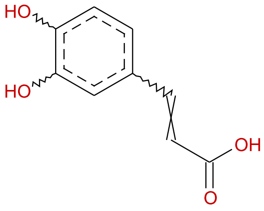	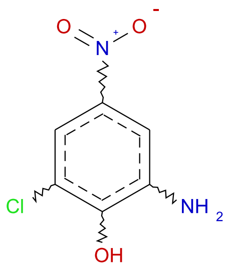	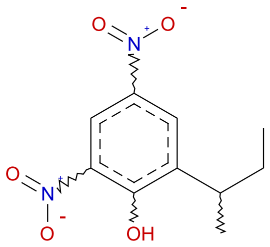
Actual Endpoint	Toxic	Non-Toxic	Toxic
Predicted Endpoint	Toxic	Non-Toxic	Toxic
Distance	0.650	0.657	0.672
Reference	Toxicol Appl Pharmacol 36(2):227-37; 1976	Food Chem Toxicol 22(2):147-149; 1984	Arch Environ Contam Toxicol 15(4):377-84; 1986

Model Applicability

Unknown features are fingerprint features in the query molecule, but not found in the training set.

1. All properties and OPS components are within expected ranges.

Feature Contribution

Top features for positive contribution

Fingerprint	Bit/Smiles	Feature Structure	Score	Toxic in training set
SCFP_6	-1357949052	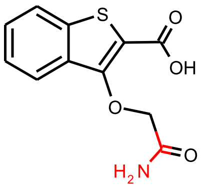 [*]C(=[*])N	0.453	8 out of 9

SCFP_6	276283342	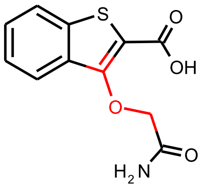 [*]CO[c](:[*]):[*]	0.196	20 out of 31
SCFP_6	560173167	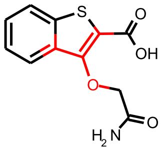 [*]O[c](:[cH]:[*]):[cH]:[*]	0.182	43 out of 68
**Top Features for negative contribution**				
**Fingerprint**	**Bit/Smiles**	**Feature Structure**	**Score**	**Toxic in training set**
SCFP_6	2083824450	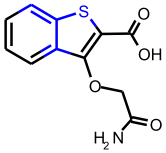 [*]:[cH]:[c]1:s:[*]:[*]:[c]:1:[*]	-0.422	0 out of 1
SCFP_6	1653687455	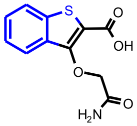 [*]1:[*]:[c]2:[cH]:[cH]:[cH]:[cH]:[c]:2:s:1	-0.422	0 out of 1
SCFP_6	-1376127683	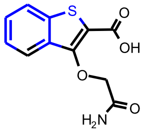 [*]1:[*]:[c]2:[*]:[cH]:[cH]:[cH]:[c]:2:s:1	-0.422	0 out of 1

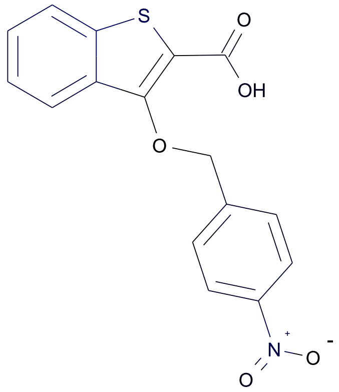

$C_{16}H_{11}NO_5S$
Molecular Weight: 329.32724
ALogP: 4.074
Rotatable Bonds: 5
Acceptors: 5
Donors: 1

Model Prediction

Prediction: Non-Toxic
Probability: 0.403
Enrichment: 0.766
Bayesian Score: -4.307
Mahalanobis Distance: 10.962
Mahalanobis Distance p-value: 0.00124

Prediction: Positive if the Bayesian score is above the estimated best cutoff value from minimizing the false positive and false negative rate.
Probability: The esimated probability that the sample is in the positive category. This assumes that the Bayesian score follows a normal distribution and is different from the prediction using a cutoff.
Enrichment: An estimate of enrichment, that is, the increased likelihood (versus random) of this sample being in the category.
Bayesian Score: The standard Laplacian-modified Bayesian score.
Mahalanobis Distance: The Mahalanobis distance (MD) is the distance to the center of the training data. The larger the MD, the less trustworthy the prediction.
Mahalanobis Distance p-value: The p-value gives the fraction of training data with an MD greater than or equal to the one for the given sample, assuming normally distributed data. The smaller the p-value, the less trustworthy the prediciton. For highly non-normal X properties (e.g., fingerprints), the MD p-value is wildly inaccurate.

Structural Similar Compounds

Name	Dinoseb	Suprofen	Guthion
Structure	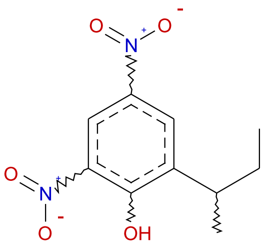	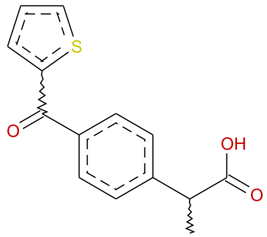	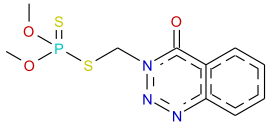
Actual Endpoint	Toxic	Non-Toxic	Non-Toxic
Predicted Endpoint	Toxic	Non-Toxic	Non-Toxic
Distance	0.608	0.626	0.672
Reference	Arch Environ Contam Toxicol 15(4):377-84; 1986	Oyo Yakuri 26:449-459; 1983	Arch Toxicol 43:177-186; 1980

Model Applicability

Unknown features are fingerprint features in the query molecule, but not found in the training set.

1. All properties and OPS components are within expected ranges.

Feature Contribution

Top features for positive contribution				
**Fingerprint**	**Bit/Smiles**	**Feature Structure**	**Score**	**Toxic in training set**
SCFP_6	276283342	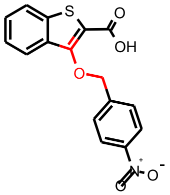 [*]CO[c](:[*]):[*]	0.196	20 out of 31

SCFP_6	560173167	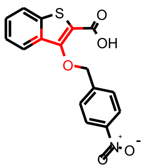 [*]O[c](:[cH]:[*]):[cH]:[*]	0.182	43 out of 68
**Top Features for negative contribution**				
**Fingerprint**	**Bit/Smiles**	**Feature Structure**	**Score**	**Toxic in training set**
SCFP_6	-1380909229	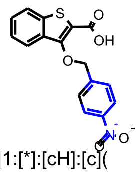 [*][c]1:[*]:[cH]:[c](:[cH]:[cH]:1)[N+](=[*])[*]	-0.449	6 out of 19
SCFP_6	1311339974	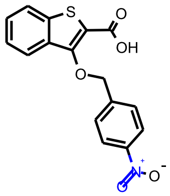 [*][N+](=O)[*]	-0.446	3 out of 10
SCFP_6	2083824450	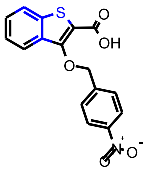 [*]:[cH]:[c]1:s:[*]:[*]:[c]:1:[*]	-0.422	0 out of 1

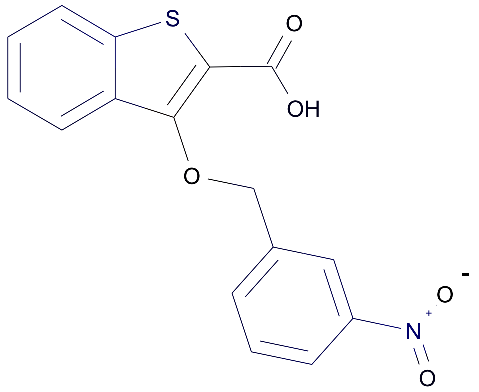

$C_{16}H_{11}NO_5S$

Molecular Weight: 329.32724

ALogP: 4.074

Rotatable Bonds: 5

Acceptors: 5

Donors: 1

Structural Similar Compounds

Name	Dinoseb	Suprofen	Guthion
Structure	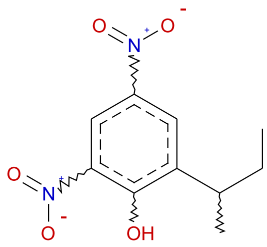	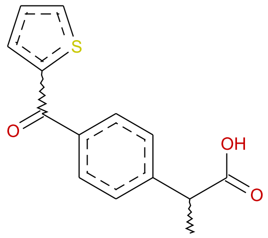	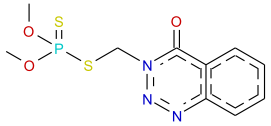
Actual Endpoint	Toxic	Non-Toxic	Non-Toxic
Predicted Endpoint	Toxic	Non-Toxic	Non-Toxic
Distance	0.603	0.629	0.673
Reference	Arch Environ Contam Toxicol 15(4):377-84; 1986	Oyo Yakuri 26:449-459; 1983	Arch Toxicol 43:177-186; 1980

Model Prediction

Prediction: Non-Toxic

Probability: 0.383

Enrichment: 0.728

Bayesian Score: -5.025

Mahalanobis Distance: 10.962

Mahalanobis Distance p-value: 0.00124

Prediction: Positive if the Bayesian score is above the estimated best cutoff value from minimizing the false positive and false negative rate.
Probability: The esimated probability that the sample is in the positive category. This assumes that the Bayesian score follows a normal distribution and is different from the prediction using a cutoff.
Enrichment: An estimate of enrichment, that is, the increased likelihood (versus random) of this sample being in the category.
Bayesian Score: The standard Laplacian-modified Bayesian score.
Mahalanobis Distance: The Mahalanobis distance (MD) is the distance to the center of the training data. The larger the MD, the less trustworthy the prediction.
Mahalanobis Distance p-value: The p-value gives the fraction of training data with an MD greater than or equal to the one for the given sample, assuming normally distributed data. The smaller the p-value, the less trustworthy the prediciton. For highly non-normal X properties (e.g., fingerprints), the MD p-value is wildly inaccurate.

Model Applicability

Unknown features are fingerprint features in the query molecule, but not found in the training set.

1. All properties and OPS components are within expected ranges.

Feature Contribution

Top features for positive contribution

Fingerprint	Bit/Smiles	Feature Structure	Score	Toxic in training set
SCFP_6	276283342	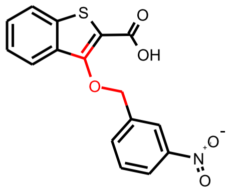 [*]CO[c](:[*]):[*]	0.196	20 out of 31

SCFP_6	560173167	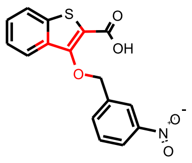 [*]O[c](:[cH]:[*]):[cH]:[*]	0.182	43 out of 68
**Top Features for negative contribution**				
**Fingerprint**	**Bit/Smiles**	**Feature Structure**	**Score**	**Toxic in training set**
SCFP_6	-1630708879	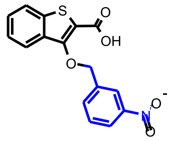 [*]C[c]1:[cH]:[cH]:[cH]:[c](:[cH]:1)[N+](=[*])[*]	-0.718	0 out of 2
SCFP_6	-1380909229	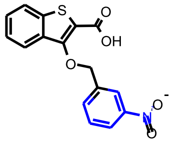 [*][c]1:[*]:[cH]:[c](:[cH]:[cH]:1)[N+](=[*])[*]	-0.449	6 out of 19
SCFP_6	1311339974	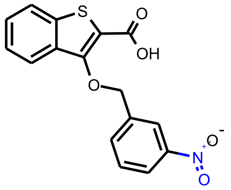 [*][N+](=O)[*]	-0.446	3 out of 10

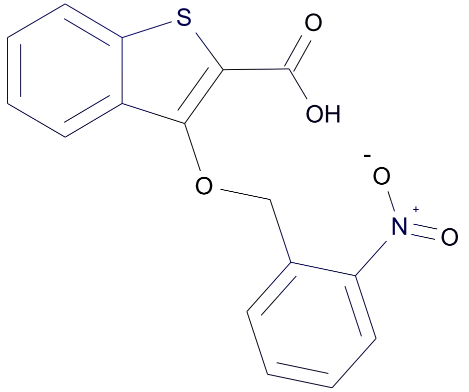

$C_{16}H_{11}NO_5S$
Molecular Weight: 329.32724
ALogP: 4.074
Rotatable Bonds: 5
Acceptors: 5
Donors: 1

Model Prediction

Prediction: Non-Toxic
Probability: 0.396
Enrichment: 0.753
Bayesian Score: -4.560
Mahalanobis Distance: 11.885
Mahalanobis Distance p-value: 4.53e-005

Prediction: Positive if the Bayesian score is above the estimated best cutoff value from minimizing the false positive and false negative rate.
Probability: The esimated probability that the sample is in the positive category. This assumes that the Bayesian score follows a normal distribution and is different from the prediction using a cutoff.
Enrichment: An estimate of enrichment, that is, the increased likelihood (versus random) of this sample being in the category.
Bayesian Score: The standard Laplacian-modified Bayesian score.
Mahalanobis Distance: The Mahalanobis distance (MD) is the distance to the center of the training data. The larger the MD, the less trustworthy the prediction.
Mahalanobis Distance p-value: The p-value gives the fraction of training data with an MD greater than or equal to the one for the given sample, assuming normally distributed data. The smaller the p-value, the less trustworthy the prediciton. For highly non-normal X properties (e.g., fingerprints), the MD p-value is wildly inaccurate.

Structural Similar Compounds

Name	Dinoseb	Suprofen	Guthion
Structure	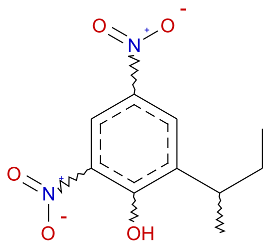	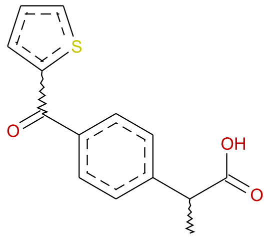	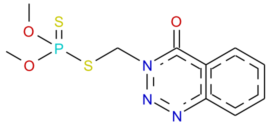
Actual Endpoint	Toxic	Non-Toxic	Non-Toxic
Predicted Endpoint	Toxic	Non-Toxic	Non-Toxic
Distance	0.609	0.628	0.673
Reference	Arch Environ Contam Toxicol 15(4):377-84; 1986	Oyo Yakuri 26:449-459; 1983	Arch Toxicol 43:177-186; 1980

Model Applicability

Unknown features are fingerprint features in the query molecule, but not found in the training set.

1. All properties and OPS components are within expected ranges.

Feature Contribution

Top features for positive contribution

Fingerprint	Bit/Smiles	Feature Structure	Score	Toxic in training set
SCFP_6	276283342	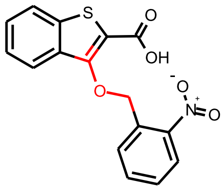 [*]CO[c](:[*]):[*]	0.196	20 out of 31

SCFP_6	560173167	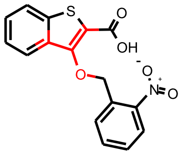 [*]O[c](:[cH]:[*]):[cH]:[*]	0.182	43 out of 68

Top Features for negative contribution

Fingerprint	Bit/Smiles	Feature Structure	Score	Toxic in training set
SCFP_6	-1380909229	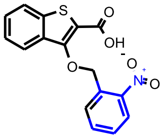 [*][c]1:[*]:[cH]:[c](:[cH]:[cH]:1)[N+](=[*])[*]	-0.449	6 out of 19
SCFP_6	1311339974	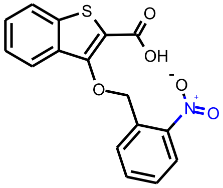 [*][N+](=O)[*]	-0.446	3 out of 10
SCFP_6	-1376127683	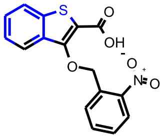 [*]1:[*]:[c]2:[*]:[cH]:[cH]:[cH]:[c]:2:s:1	-0.422	0 out of 1

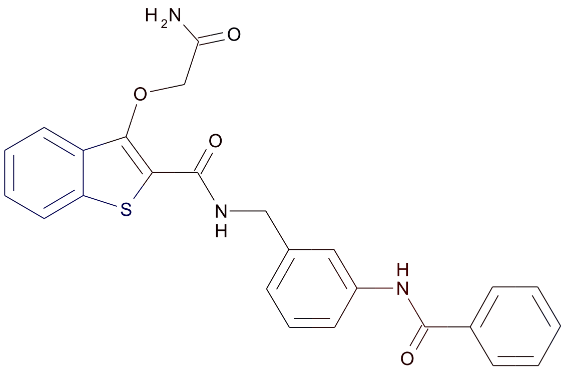

$C_{25}H_{21}N_3O_4S$
Molecular Weight: 459.51694
ALogP: 3.396
Rotatable Bonds: 8
Acceptors: 4
Donors: 3

Model Prediction

Prediction: Toxic
Probability: 0.542
Enrichment: 1.031
Bayesian Score: -0.142
Mahalanobis Distance: 12.243
Mahalanobis Distance p-value: 1.1e-005

Prediction: Positive if the Bayesian score is above the estimated best cutoff value from minimizing the false positive and false negative rate.
Probability: The esimated probability that the sample is in the positive category. This assumes that the Bayesian score follows a normal distribution and is different from the prediction using a cutoff.
Enrichment: An estimate of enrichment, that is, the increased likelihood (versus random) of this sample being in the category.
Bayesian Score: The standard Laplacian-modified Bayesian score.
Mahalanobis Distance: The Mahalanobis distance (MD) is the distance to the center of the training data. The larger the MD, the less trustworthy the prediction.
Mahalanobis Distance p-value: The p-value gives the fraction of training data with an MD greater than or equal to the one for the given sample, assuming normally distributed data. The smaller the p-value, the less trustworthy the prediciton. For highly non-normal X properties (e.g., fingerprints), the MD p-value is wildly inaccurate.

Structural Similar Compounds

Name	Ochratoxin a	Amsacrine	Etofenamate
Structure	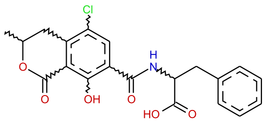	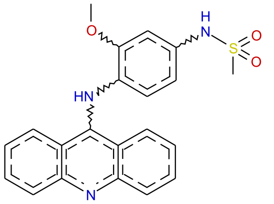	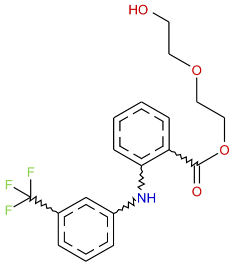
Actual Endpoint	Toxic	Toxic	Non-Toxic
Predicted Endpoint	Toxic	Toxic	Non-Toxic
Distance	0.664	0.704	0.719
Reference	Toxicol Appl Pharmacol 37(2):331-8; 1976	Fundam Appl Toxicol 7(2):214-20; 1986	Iyakuhin Kenkyu 13(4):896-909; 1982

Model Applicability

Unknown features are fingerprint features in the query molecule, but not found in the training set.

1. All properties and OPS components are within expected ranges.

Feature Contribution

Top features for positive contribution				
**Fingerprint**	**Bit/Smiles**	**Feature Structure**	**Score**	**Toxic in training set**
SCFP_6	-1357949052	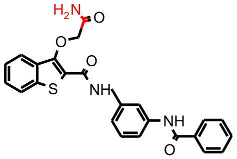 [*]C(=[*])N	0.453	8 out of 9

SCFP_6	282594097	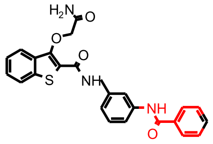 [*]NC(=O)[c]1:[cH]:[cH]:[*]:[cH]:[cH]:1	0.441	3 out of 3
SCFP_6	-347281112	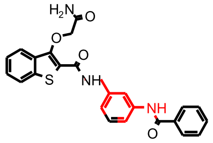 [*]C[c]1:[cH]:[*]:[cH]:[c](N[*]):[cH]:1	0.381	2 out of 2

Top Features for negative contribution

Fingerprint	Bit/Smiles	Feature Structure	Score	Toxic in training set
SCFP_6	2083824450	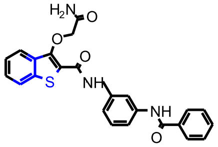 [*]:[cH]:[c]1:s:[*]:[*]:[c]:1:[*]	-0.422	0 out of 1
SCFP_6	-1376127683	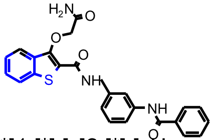 [*]1:[*]:[c]2:[*]:[cH]:[cH]:[cH]:[c]:2:s:1	-0.422	0 out of 1
SCFP_6	1653687455	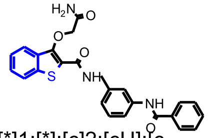 [*]1:[*]:[c]2:[cH]:[cH]:[cH]:[cH]:[c]:2:s:1	-0.422	0 out of 1

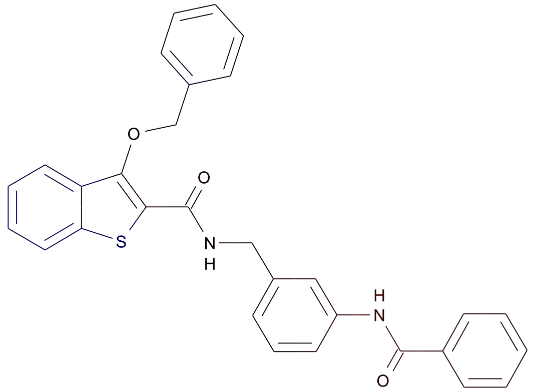

$C_{30}H_{24}N_2O_3S$
Molecular Weight: 492.58816
ALogP: 6.126
Rotatable Bonds: 8
Acceptors: 3
Donors: 2

Model Prediction

Prediction: Non-Toxic
Probability: 0.523
Enrichment: 0.995
Bayesian Score: -0.657
Mahalanobis Distance: 11.638
Mahalanobis Distance p-value: 0.000116

Prediction: Positive if the Bayesian score is above the estimated best cutoff value from minimizing the false positive and false negative rate.
Probability: The esimated probability that the sample is in the positive category. This assumes that the Bayesian score follows a normal distribution and is different from the prediction using a cutoff.
Enrichment: An estimate of enrichment, that is, the increased likelihood (versus random) of this sample being in the category.
Bayesian Score: The standard Laplacian-modified Bayesian score.
Mahalanobis Distance: The Mahalanobis distance (MD) is the distance to the center of the training data. The larger the MD, the less trustworthy the prediction.
Mahalanobis Distance p-value: The p-value gives the fraction of training data with an MD greater than or equal to the one for the given sample, assuming normally distributed data. The smaller the p-value, the less trustworthy the prediciton. For highly non-normal X properties (e.g., fingerprints), the MD p-value is wildly inaccurate.

Structural Similar Compounds

Name	Brovanexine .HCl (Free base form)	Estramustine Phosphate Disodium (Free acid form)	Pimozide
Structure	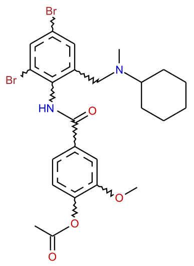	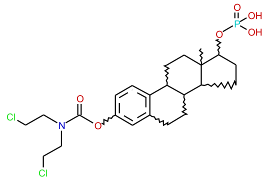	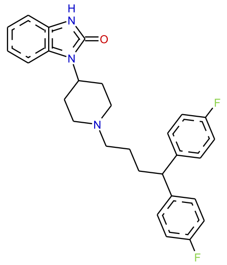
Actual Endpoint	Toxic	Non-Toxic	Non-Toxic
Predicted Endpoint	Toxic	Non-Toxic	Non-Toxic
Distance	0.648	0.678	0.681
Reference	Kiso to Rinsho 16(13):7179-7195; 1982	Oyo Yakuri 20(6):1219-1236; 1980	Kiso to Rinsho 14:2163-2170; 1980

Model Applicability

Unknown features are fingerprint features in the query molecule, but not found in the training set.

1. All properties and OPS components are within expected ranges.

Feature Contribution

Top features for positive contribution				
Fingerprint	Bit/Smiles	Feature Structure	Score	Toxic in training set
SCFP_6	282594097	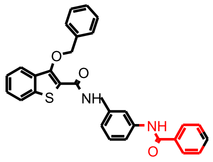 [*]NC(=O)[c]1:[cH]:[cH]:[*]:[cH]:[cH]:1	0.441	3 out of 3

SCFP_6	-347281112	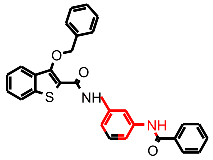 [*]C[c]1:[cH]:[*]:[cH]:[c](N[*]):[cH]:1	0.381	2 out of 2
SCFP_6	1257084377	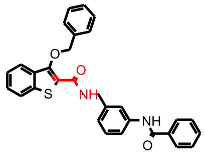 [*]NC(=O)[c](:[*]):[*]	0.362	14 out of 18

Top Features for negative contribution

Fingerprint	Bit/Smiles	Feature Structure	Score	Toxic in training set
SCFP_6	-1376127683	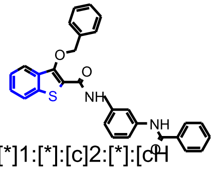 [*]1:[*]:[c]2:[*]:[cH]:[cH]:[cH]:[c]:2:s: 1	-0.422	0 out of 1
SCFP_6	1653687455	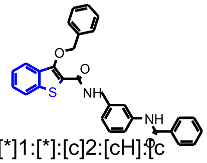 [*]1:[*]:[c]2:[cH]:[c H]:[cH]:[cH]:[c]:2:s :1	-0.422	0 out of 1
SCFP_6	-711596826	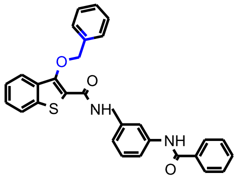 [*]OC[c](:[*]):[*]	-0.422	0 out of 1

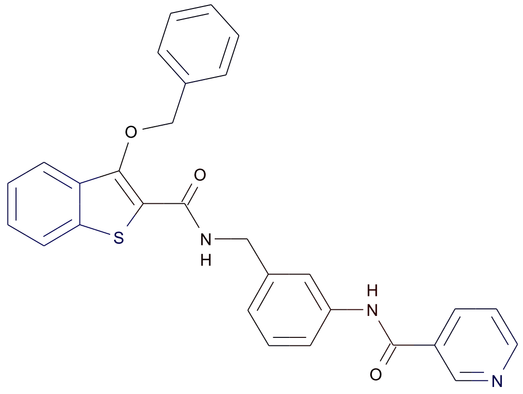

$C_{29}H_{23}N_3O_3S$
Molecular Weight: 493.57622
ALogP: 4.976
Rotatable Bonds: 8
Acceptors: 4
Donors: 2

Model Prediction

Prediction: Non-Toxic
Probability: 0.461
Enrichment: 0.877
Bayesian Score: -2.429
Mahalanobis Distance: 11.593
Mahalanobis Distance p-value: 0.000137

Prediction: Positive if the Bayesian score is above the estimated best cutoff value from minimizing the false positive and false negative rate.
Probability: The esimated probability that the sample is in the positive category. This assumes that the Bayesian score follows a normal distribution and is different from the prediction using a cutoff.
Enrichment: An estimate of enrichment, that is, the increased likelihood (versus random) of this sample being in the category.
Bayesian Score: The standard Laplacian-modified Bayesian score.
Mahalanobis Distance: The Mahalanobis distance (MD) is the distance to the center of the training data. The larger the MD, the less trustworthy the prediction.
Mahalanobis Distance p-value: The p-value gives the fraction of training data with an MD greater than or equal to the one for the given sample, assuming normally distributed data. The smaller the p-value, the less trustworthy the prediciton. For highly non-normal X properties (e.g., fingerprints), the MD p-value is wildly inaccurate.

Structural Similar Compounds

Name	Estramustine Phosphate Disodium (Free acid form)	Brovanexine .HCl (Free base form)	Acemetacin
Structure	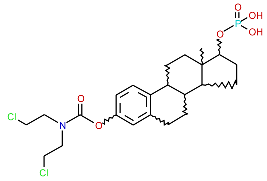	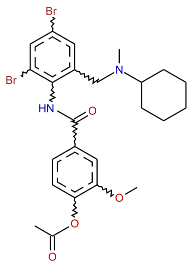	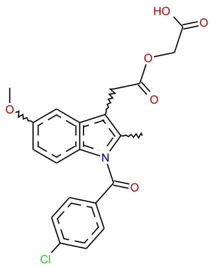
Actual Endpoint	Non-Toxic	Toxic	Non-Toxic
Predicted Endpoint	Non-Toxic	Toxic	Non-Toxic
Distance	0.604	0.657	0.686
Reference	Oyo Yakuri 20(6):1219-1236; 1980	Kiso to Rinsho 16(13):7179-7195; 1982	Oyo Yakuri 22(6):777-786; 1981

Model Applicability

Unknown features are fingerprint features in the query molecule, but not found in the training set.

1. All properties and OPS components are within expected ranges.

Feature Contribution

Top features for positive contribution

Fingerprint	Bit/Smiles	Feature Structure	Score	Toxic in training set
SCFP_6	-347281112	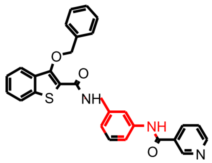 [*]C[c]1:[cH]:[*]:[cH]:[c](N[*]):[cH]:1	0.381	2 out of 2

SCFP_6	1257084377	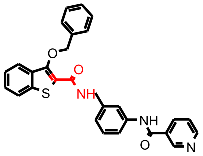 [*]NC(=O)[c](:[*]):[*]	0.362	14 out of 18
SCFP_6	903335088	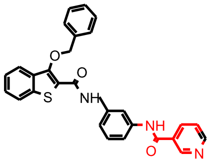 [*]NC(=O)[c]1:[cH]:[cH]:[*]:n:[cH]:1	0.271	1 out of 1

Top Features for negative contribution

Fingerprint	Bit/Smiles	Feature Structure	Score	Toxic in training set
SCFP_6	-758850909	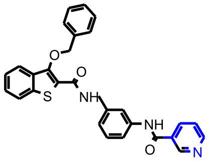 [*][c]1:[*]:n:[cH]:[cH]:[cH]:1	-0.646	2 out of 9
SCFP_6	-1376127683	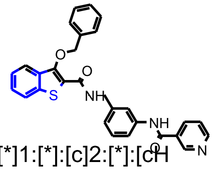 [*]1:[*]:[c]2:[*]:[cH]:[cH]:[cH]:[c]:2:s:1	-0.422	0 out of 1
SCFP_6	1653687455	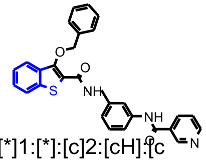 [*]1:[*]:[c]2:[cH]:[cH]:[cH]:[cH]:[c]:2:s:1	-0.422	0 out of 1

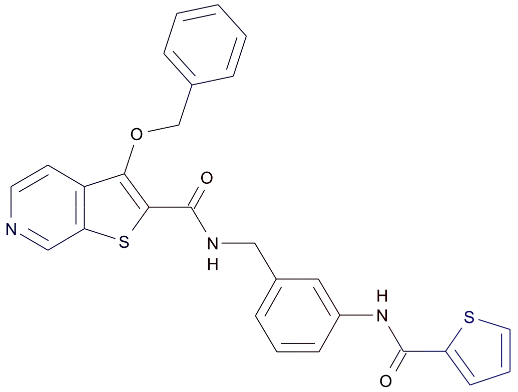

$C_{27}H_{21}N_3O_3S_2$
Molecular Weight: 499.60394
ALogP: 4.929
Rotatable Bonds: 8
Acceptors: 4
Donors: 2

Model Prediction

Prediction: Non-Toxic
Probability: 0.448
Enrichment: 0.852
Bayesian Score: -2.830
Mahalanobis Distance: 11.282
Mahalanobis Distance p-value: 0.000418

Prediction: Positive if the Bayesian score is above the estimated best cutoff value from minimizing the false positive and false negative rate.
Probability: The esimated probability that the sample is in the positive category. This assumes that the Bayesian score follows a normal distribution and is different from the prediction using a cutoff.
Enrichment: An estimate of enrichment, that is, the increased likelihood (versus random) of this sample being in the category.
Bayesian Score: The standard Laplacian-modified Bayesian score.
Mahalanobis Distance: The Mahalanobis distance (MD) is the distance to the center of the training data. The larger the MD, the less trustworthy the prediction.
Mahalanobis Distance p-value: The p-value gives the fraction of training data with an MD greater than or equal to the one for the given sample, assuming normally distributed data. The smaller the p-value, the less trustworthy the prediciton. For highly non-normal X properties (e.g., fingerprints), the MD p-value is wildly inaccurate.

Structural Similar Compounds

Name	Estramustine Phosphate Disodium (Free acid form)	Acemetacin	Amsacrine
Structure	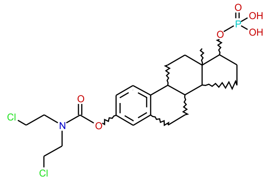	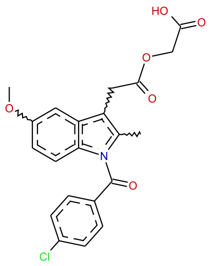	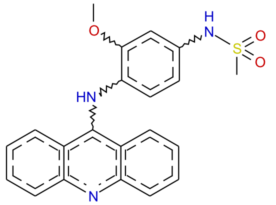
Actual Endpoint	Non-Toxic	Non-Toxic	Toxic
Predicted Endpoint	Non-Toxic	Non-Toxic	Toxic
Distance	0.629	0.706	0.707
Reference	Oyo Yakuri 20(6):1219-1236; 1980	Oyo Yakuri 22(6):777-786; 1981	Fundam Appl Toxicol 7(2):214-20; 1986

Model Applicability

Unknown features are fingerprint features in the query molecule, but not found in the training set.

1. All properties and OPS components are within expected ranges.

Feature Contribution

Top features for positive contribution

Fingerprint	Bit/Smiles	Feature Structure	Score	Toxic in training set
SCFP_6	-347281112	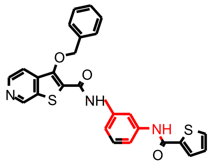 [*]C[c]1:[cH]:[*]:[cH]:[c](N[*]):[cH]:1	0.381	2 out of 2

SCFP_6	1257084377	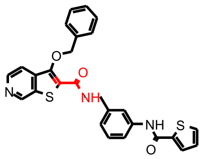 [*]NC(=O)[c](:[*]):[*]	0.362	14 out of 18
SCFP_6	1631845520	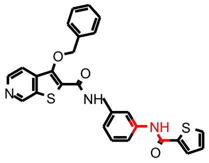 [*]C(=[*])N[c](:[*]): [*]	0.210	8 out of 12

Top Features for negative contribution

**Fingerprint**	**Bit/Smiles**	**Feature Structure**	**Score**	**Toxic in training set**
SCFP_6	1912773504	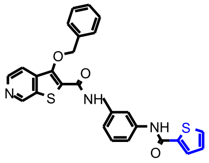 [*][c]1:[cH]:[cH]:[cH]:s:1	-0.718	0 out of 2
SCFP_6	2083824450	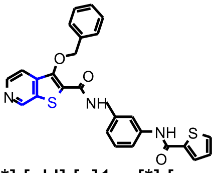 [*]:[cH]:[c]1:s:[*]:[*]:[c]:1:[*]	-0.422	0 out of 1
SCFP_6	152804597	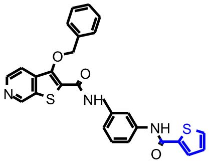 [*]C(=[*])[c]1:[cH]:[cH]:[cH]:s:1	-0.422	0 out of 1

Co-crystallized ligand

TOPKAT_Developmental_Toxicity_Potential

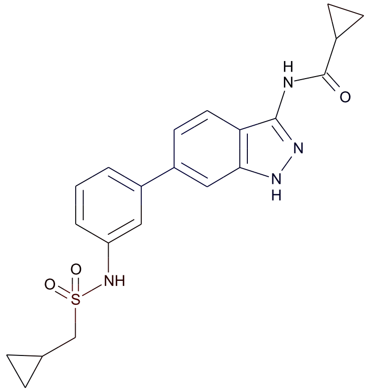

$C_{21}H_{22}N_4O_3S$
Molecular Weight: 410.48938
ALogP: 3.14
Rotatable Bonds: 7
Acceptors: 4
Donors: 3

Model Prediction

Prediction: Non-Toxic
Probability: 0.514
Enrichment: 0.977
Bayesian Score: -0.913
Mahalanobis Distance: 8.474
Mahalanobis Distance p-value: 0.379

Prediction: Positive if the Bayesian score is above the estimated best cutoff value from minimizing the false positive and false negative rate.
Probability: The esimated probability that the sample is in the positive category. This assumes that the Bayesian score follows a normal distribution and is different from the prediction using a cutoff.
Enrichment: An estimate of enrichment, that is, the increased likelihood (versus random) of this sample being in the category.
Bayesian Score: The standard Laplacian-modified Bayesian score.
Mahalanobis Distance: The Mahalanobis distance (MD) is the distance to the center of the training data. The larger the MD, the less trustworthy the prediction.
Mahalanobis Distance p-value: The p-value gives the fraction of training data with an MD greater than or equal to the one for the given sample, assuming normally distributed data. The smaller the p-value, the less trustworthy the prediciton. For highly non-normal X properties (e.g., fingerprints), the MD p-value is wildly inaccurate.

Structural Similar Compounds

Name	Amsacrine	Ochratoxin a	Benomyl
Structure	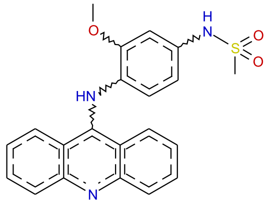	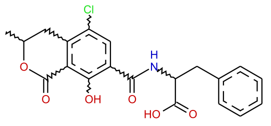	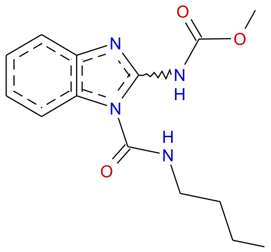
Actual Endpoint	Toxic	Toxic	Toxic
Predicted Endpoint	Toxic	Toxic	Toxic
Distance	0.631	0.644	0.681
Reference	Fundam Appl Toxicol 7(2):214-20; 1986	Toxicol Appl Pharmacol 37(2):331-8; 1976	J Toxicol Environ Health 17:405-417; 1986

Model Applicability

Unknown features are fingerprint features in the query molecule, but not found in the training set.

1. All properties and OPS components are within expected ranges.

Feature Contribution

Top features for positive contribution

Fingerprint	Bit/Smiles	Feature Structure	Score	Toxic in training set
SCFP_6	-26456656	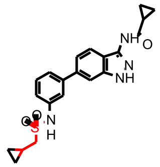 [*]S(=[*])(=[*])CC1[*][*]1	0.381	2 out of 2

SCFP_6	1382093202	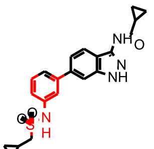 [*][c]1:[*]:[cH]:[cH]:[c](NS(=[*])(=[*])[*]):[cH]:1	0.271	1 out of 1
SCFP_6	149168366	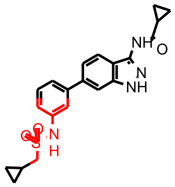 [*]CS(=O)(=O)N[c](:[cH]:[*]):[cH]:[*]	0.271	1 out of 1

Top Features for negative contribution

Fingerprint	Bit/Smiles	Feature Structure	Score	Toxic in training set
SCFP_6	149212520	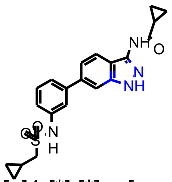 [*]:[c]1:[*]:[*]:n:[nH]:1	-0.448	5 out of 16
SCFP_6	-1800047921	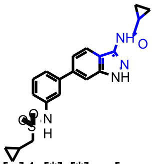 [*]:[c]1:[*]:[*]:n:[c]:1NC(=O)C2[*][*]2	-0.422	0 out of 1
SCFP_6	-1798553344	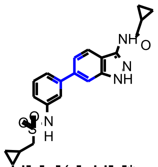 [*]:[cH]:[c](:[cH]:[*])[c](:[*]):[*]	-0.358	3 out of 9

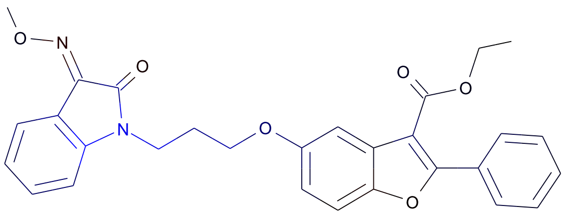

$C_{29}H_{26}N_2O_6$
Molecular Weight: 498.52653
ALogP: 4.994
Rotatable Bonds: 10
Acceptors: 6
Donors: 0

Model Prediction

Prediction: Non-Carcinogen
Probability: 0.219
Enrichment: 0.684
Bayesian Score: -9.584
Mahalanobis Distance: 17.328
Mahalanobis Distance p-value: 4.75e-012

Prediction: Positive if the Bayesian score is above the estimated best cutoff value from minimizing the false positive and false negative rate.
Probability: The esimated probability that the sample is in the positive category. This assumes that the Bayesian score follows a normal distribution and is different from the prediction using a cutoff.
Enrichment: An estimate of enrichment, that is, the increased likelihood (versus random) of this sample being in the category.
Bayesian Score: The standard Laplacian-modified Bayesian score.
Mahalanobis Distance: The Mahalanobis distance (MD) is the distance to the center of the training data. The larger the MD, the less trustworthy the prediction.
Mahalanobis Distance p-value: The p-value gives the fraction of training data with an MD greater than or equal to the one for the given sample, assuming normally distributed data. The smaller the p-value, the less trustworthy the prediciton. For highly non-normal X properties (e.g., fingerprints), the MD p-value is wildly inaccurate.

Structural Similar Compounds

Name	Nefazodone	Verapamil	Ketoconazole
Structure	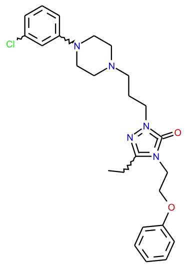	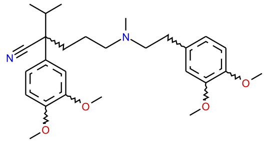	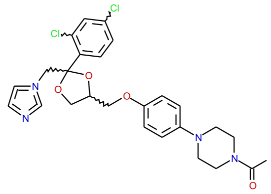
Actual Endpoint	Non-Carcinogen	Non-Carcinogen	Non-Carcinogen
Predicted Endpoint	Non-Carcinogen	Non-Carcinogen	Non-Carcinogen
Distance	0.578	0.691	0.696
Reference	US FDA (Centre for Drug Eval.& Res./Off. Testing & Res.) Sept. 1997	US FDA (Centre for Drug Eval.& Res./Off. Testing & Res.) Sept. 1997	US FDA (Centre for Drug Eval.& Res./Off. Testing & Res.) Sept. 1997

Model Applicability

Unknown features are fingerprint features in the query molecule, but not found in the training set.

1. All properties and OPS components are within expected ranges.
2. Unknown ECFP_2 feature: -1658273810: [*]C(=[*])[c]1:[c]([*]):[*]:[*]:[c]:1:[*]
3. Unknown ECFP_2 feature: 1716966732: [*]\N=C/1\C(=[*])[*][*]:[c]1:[*]
4. Unknown ECFP_2 feature: -820505146: [*]ON=C([*])[*]
5. Unknown ECFP_2 feature: -408704017: [*]=NOC

Feature Contribution

Top features for positive contribution				
Fingerprint	Bit/Smiles	Feature Structure	Score	Carcinogen in training set
ECFP_6	-1087070950	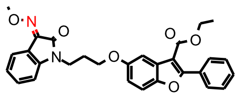 [*]N=[*]	0.724	10 out of 14

ECFP_6	1334014211	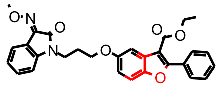 [*]:[cH]:[c]1:o:[*]:[*]:[c]:1:[*]	0.424	1 out of 1
ECFP_6	1203316083	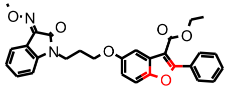 [*][c]1:[*]:[*]:[c](: [*]):o:1	0.337	3 out of 6

Top Features for negative contribution

Fingerprint	Bit/Smiles	Feature Structure	Score	Carcinogen in training set
ECFP_6	-661097313	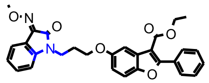 [*]CN1C(=[*])[*][*]:[c]1:[*]	-1.550	0 out of 12
ECFP_6	-2125181541	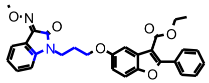 [*]CCCN1C(=[*])[*][*] :[c]1:[*]	-0.805	0 out of 4
ECFP_6	-1998067937	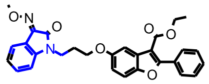 [*]CN1C(=[*])C(=[*])[c]2:[cH]:[*]:[cH]:[c H]:[c]1:2	-0.657	0 out of 3

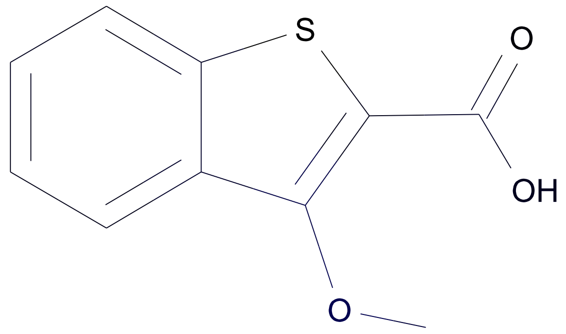

$C_{10}H_8O_3S$
Molecular Weight: 208.23372
ALogP: 2.596
Rotatable Bonds: 2
Acceptors: 3
Donors: 1

Model Prediction

Prediction: Non-Carcinogen
Probability: 0.213
Enrichment: 0.665
Bayesian Score: -3.828
Mahalanobis Distance: 10.844
Mahalanobis Distance p-value: 0.138

Prediction: Positive if the Bayesian score is above the estimated best cutoff value from minimizing the false positive and false negative rate.
Probability: The esimated probability that the sample is in the positive category. This assumes that the Bayesian score follows a normal distribution and is different from the prediction using a cutoff.
Enrichment: An estimate of enrichment, that is, the increased likelihood (versus random) of this sample being in the category.
Bayesian Score: The standard Laplacian-modified Bayesian score.
Mahalanobis Distance: The Mahalanobis distance (MD) is the distance to the center of the training data. The larger the MD, the less trustworthy the prediction.
Mahalanobis Distance p-value: The p-value gives the fraction of training data with an MD greater than or equal to the one for the given sample, assuming normally distributed data. The smaller the p-value, the less trustworthy the prediciton. For highly non-normal X properties (e.g., fingerprints), the MD p-value is wildly inaccurate.

Structural Similar Compounds

Name	Aspirin	Thiabendazole	Suprofen
Structure	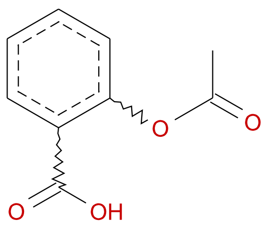	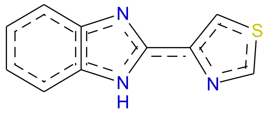	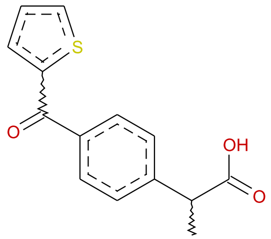
Actual Endpoint	Non-Carcinogen	Non-Carcinogen	Carcinogen
Predicted Endpoint	Non-Carcinogen	Non-Carcinogen	Carcinogen
Distance	0.561	0.572	0.599
Reference	US FDA (Centre for Drug Eval.& Res./Off. Testing & Res.) Sept. 1997	US FDA (Centre for Drug Eval.& Res./Off. Testing & Res.) Sept. 1997	US FDA (Centre for Drug Eval.& Res./Off. Testing & Res.) Sept. 1997

Model Applicability

Unknown features are fingerprint features in the query molecule, but not found in the training set.

1. All properties and OPS components are within expected ranges.
2. Unknown ECFP_2 feature: -1670580914: [*]C(=[*])[c]1:s:[*]:[*]:[c]:1[*]
3. Unknown ECFP_2 feature: 1895035276: [*]:[cH]:[c]1:s:[*]:[*]:[c]:1:[*]

Feature Contribution

Top features for positive contribution

Fingerprint	Bit/Smiles	Feature Structure	Score	Carcinogen in training set
ECFP_6	914325265	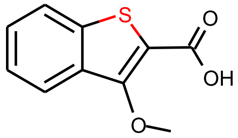 [*]:s:[*]	0.127	4 out of 11

Top Features for negative contribution

Fingerprint	Bit/Smiles	Feature Structure	Score	Carcinogen in training set
ECFP_6	1307307440	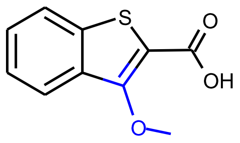 [*]:[c](:[*])OC	-0.558	4 out of 25
ECFP_6	2025485523	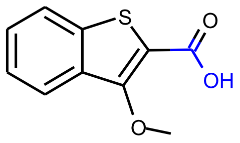 [*]C(=[*])O	-0.506	8 out of 45
ECFP_6	864909220	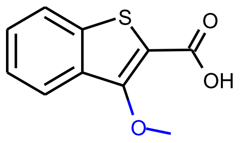 [*]OC	-0.466	7 out of 38

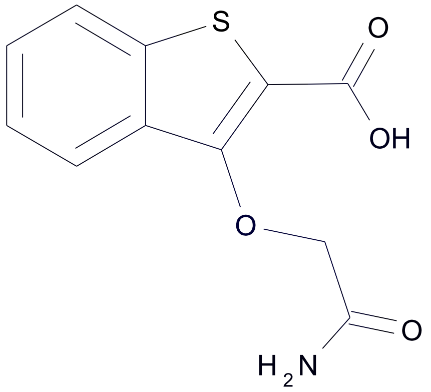

$C_{11}H_9NO_4S$
Molecular Weight: 251.25846
ALogP: 1.45
Rotatable Bonds: 4
Acceptors: 4
Donors: 2

Model Prediction

Prediction: Non-Carcinogen
Probability: 0.221
Enrichment: 0.689
Bayesian Score: -2.878
Mahalanobis Distance: 13.671
Mahalanobis Distance p-value: 5.12e-005

Prediction: Positive if the Bayesian score is above the estimated best cutoff value from minimizing the false positive and false negative rate.
Probability: The esimated probability that the sample is in the positive category. This assumes that the Bayesian score follows a normal distribution and is different from the prediction using a cutoff.
Enrichment: An estimate of enrichment, that is, the increased likelihood (versus random) of this sample being in the category.
Bayesian Score: The standard Laplacian-modified Bayesian score.
Mahalanobis Distance: The Mahalanobis distance (MD) is the distance to the center of the training data. The larger the MD, the less trustworthy the prediction.
Mahalanobis Distance p-value: The p-value gives the fraction of training data with an MD greater than or equal to the one for the given sample, assuming normally distributed data. The smaller the p-value, the less trustworthy the prediciton. For highly non-normal X properties (e.g., fingerprints), the MD p-value is wildly inaccurate.

Structural Similar Compounds

Name	Nithiazide	Sulfisoxazole	Captopril
Structure	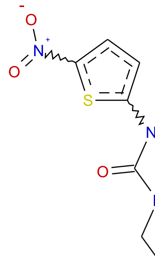	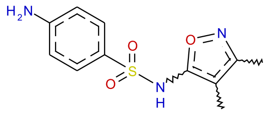	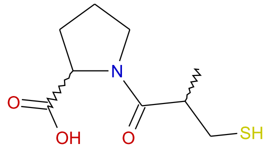
Actual Endpoint	Non-Carcinogen	Non-Carcinogen	Non-Carcinogen
Predicted Endpoint	Non-Carcinogen	Non-Carcinogen	Non-Carcinogen
Distance	0.572	0.607	0.612
Reference	US FDA (Centre for Drug Eval.& Res./Off. Testing & Res.) Sept. 1997	US FDA (Centre for Drug Eval.& Res./Off. Testing & Res.) Sept. 1997	US FDA (Centre for Drug Eval.& Res./Off. Testing & Res.) Sept. 1997

Model Applicability

Unknown features are fingerprint features in the query molecule, but not found in the training set.

1. All properties and OPS components are within expected ranges.
2. Unknown ECFP_2 feature: -1670580914: [*]C(=[*])[c]1:s:[*]:[*]:[c]:1[*]
3. Unknown ECFP_2 feature: 1895035276: [*]:[cH]:[c]1:s:[*]:[*]:[c]:1:[*]

Feature Contribution

Top features for positive contribution

Fingerprint	Bit/Smiles	Feature Structure	Score	Carcinogen in training set
ECFP_6	914325265	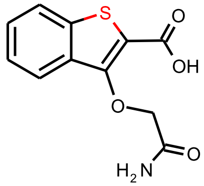 [*]:s:[*]	0.127	4 out of 11

Top Features for negative contribution

Fingerprint	Bit/Smiles	Feature Structure	Score	Carcinogen in training set
ECFP_6	2025485523	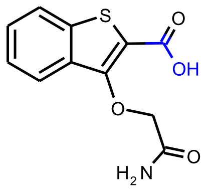 [*]C(=[*])O	-0.506	8 out of 45
ECFP_6	-1686813061	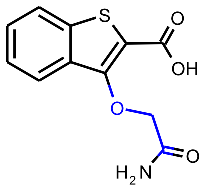 [*]OCC(=[*])[*]	-0.482	0 out of 2
ECFP_6	-1708545601	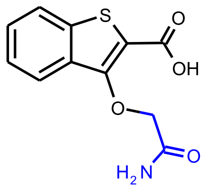 [*]CC(=O)N	-0.270	0 out of 1

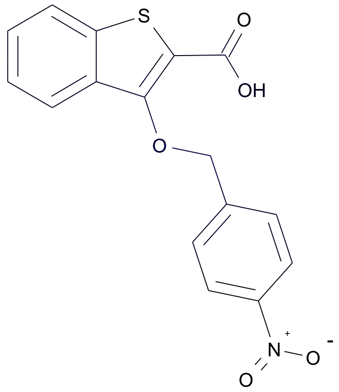

$C_{16}H_{11}NO_5S$
Molecular Weight: 329.32724
ALogP: 4.074
Rotatable Bonds: 5
Acceptors: 5
Donors: 1

Model Prediction

Prediction: Non-Carcinogen
Probability: 0.216
Enrichment: 0.675
Bayesian Score: -3.403
Mahalanobis Distance: 14.631
Mahalanobis Distance p-value: 1.18e-006

Prediction: Positive if the Bayesian score is above the estimated best cutoff value from minimizing the false positive and false negative rate.
Probability: The esimated probability that the sample is in the positive category. This assumes that the Bayesian score follows a normal distribution and is different from the prediction using a cutoff.
Enrichment: An estimate of enrichment, that is, the increased likelihood (versus random) of this sample being in the category.
Bayesian Score: The standard Laplacian-modified Bayesian score.
Mahalanobis Distance: The Mahalanobis distance (MD) is the distance to the center of the training data. The larger the MD, the less trustworthy the prediction.
Mahalanobis Distance p-value: The p-value gives the fraction of training data with an MD greater than or equal to the one for the given sample, assuming normally distributed data. The smaller the p-value, the less trustworthy the prediciton. For highly non-normal X properties (e.g., fingerprints), the MD p-value is wildly inaccurate.

Structural Similar Compounds

Name	Omeprazole	Suprofen	Lansoprazole
Structure	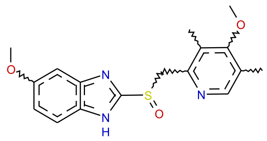	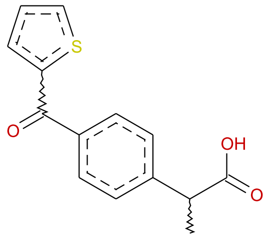	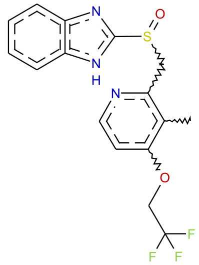
Actual Endpoint	Non-Carcinogen	Carcinogen	Carcinogen
Predicted Endpoint	Non-Carcinogen	Carcinogen	Carcinogen
Distance	0.666	0.688	0.690
Reference	US FDA (Centre for Drug Eval.& Res./Off. Testing & Res.) Sept. 1997	US FDA (Centre for Drug Eval.& Res./Off. Testing & Res.) Sept. 1997	US FDA (Centre for Drug Eval.& Res./Off. Testing & Res.) Sept. 1997

Model Applicability

Unknown features are fingerprint features in the query molecule, but not found in the training set.

1. All properties and OPS components are within expected ranges.
2. Unknown ECFP_2 feature: 1043790491: [*][N+](=[*])[*]
3. Unknown ECFP_2 feature: 781519895: [*][O-]
4. Unknown ECFP_2 feature: -1670580914: [*]C(=[*])[c]1:s:[*]:[*]:[c]:1[*]
5. Unknown ECFP_2 feature: 1895035276: [*]:[cH]:[c]1:s:[*]:[*]:[c]:1:[*]
6. Unknown ECFP_2 feature: -179073144: [*][N+](=[*])[c](:[cH]:[*]):[cH]:[*]
7. Unknown ECFP_2 feature: -215026467: [*]:[c](:[*])[N+](=O)[O-]
8. Unknown ECFP_2 feature: 2104376220: [*][N+](=O)[*]
9. Unknown ECFP_2 feature: -659271057: [*][N+](=[*])[O-]

Feature Contribution

Top features for positive contribution

Fingerprint	Bit/Smiles	Feature Structure	Score	Carcinogen in training set

ECFP_6	914325265	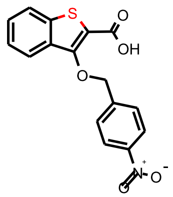 [*]:s:[*]	0.127	4 out of 11
**Top Features for negative contribution**				
**Fingerprint**	**Bit/Smiles**	**Feature Structure**	**Score**	**Carcinogen in training set**
ECFP_6	2025485523	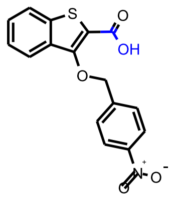 [*]C(=[*])O	-0.506	8 out of 45
ECFP_6	-1897063316	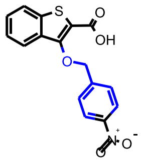 [*]OC[c]1:[cH]:[cH]:[*]:[cH]:[cH]:1	-0.270	0 out of 1
ECFP_6	770547857	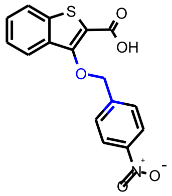 [*]OC[c](:[*]):[*]	-0.242	1 out of 5

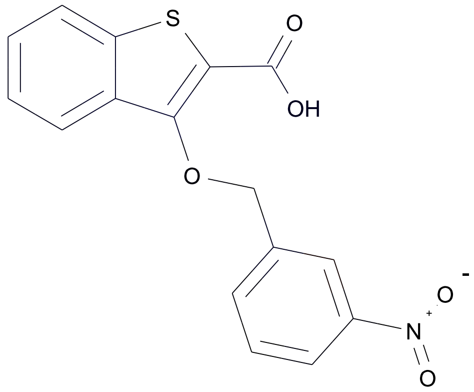

$C_{16}H_{11}NO_5S$
Molecular Weight: 329.32724
ALogP: 4.074
Rotatable Bonds: 5
Acceptors: 5
Donors: 1

Model Prediction

Prediction: Non-Carcinogen
Probability: 0.219
Enrichment: 0.683
Bayesian Score: -3.088
Mahalanobis Distance: 15.999
Mahalanobis Distance p-value: 2.85e-009

Prediction: Positive if the Bayesian score is above the estimated best cutoff value from minimizing the false positive and false negative rate.
Probability: The esimated probability that the sample is in the positive category. This assumes that the Bayesian score follows a normal distribution and is different from the prediction using a cutoff.
Enrichment: An estimate of enrichment, that is, the increased likelihood (versus random) of this sample being in the category.
Bayesian Score: The standard Laplacian-modified Bayesian score.
Mahalanobis Distance: The Mahalanobis distance (MD) is the distance to the center of the training data. The larger the MD, the less trustworthy the prediction.
Mahalanobis Distance p-value: The p-value gives the fraction of training data with an MD greater than or equal to the one for the given sample, assuming normally distributed data. The smaller the p-value, the less trustworthy the prediciton. For highly non-normal X properties (e.g., fingerprints), the MD p-value is wildly inaccurate.

Structural Similar Compounds

Name	Omeprazole	Niclosamide	Suprofen
Structure	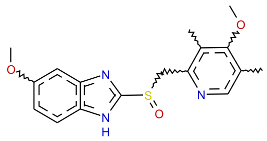	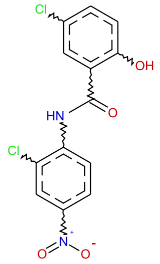	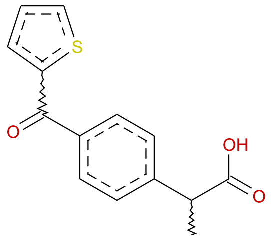
Actual Endpoint	Non-Carcinogen	Non-Carcinogen	Carcinogen
Predicted Endpoint	Non-Carcinogen	Non-Carcinogen	Carcinogen
Distance	0.663	0.688	0.691
Reference	US FDA (Centre for Drug Eval.& Res./Off. Testing & Res.) Sept. 1997	US FDA (Centre for Drug Eval.& Res./Off. Testing & Res.) Sept. 1997	US FDA (Centre for Drug Eval.& Res./Off. Testing & Res.) Sept. 1997

Model Applicability

Unknown features are fingerprint features in the query molecule, but not found in the training set.

1. All properties and OPS components are within expected ranges.
2. Unknown ECFP_2 feature: 1043790491: [*][N+](=[*])[*]
3. Unknown ECFP_2 feature: 781519895: [*][O-]
4. Unknown ECFP_2 feature: -1670580914: [*]C(=[*])[c]1:s:[*]:[*]:[c]:1[*]
5. Unknown ECFP_2 feature: 1895035276: [*]:[cH]:[c]1:s:[*]:[*]:[c]:1:[*]
6. Unknown ECFP_2 feature: -179073144: [*][N+](=[*])[c](:[cH]:[*]):[cH]:[*]
7. Unknown ECFP_2 feature: -215026467: [*]:[c](:[*])[N+](=O)[O-]
8. Unknown ECFP_2 feature: 2104376220: [*][N+](=O)[*]
9. Unknown ECFP_2 feature: -659271057: [*][N+](=[*])[O-]

Feature Contribution

Top features for positive contribution

Fingerprint	Bit/Smiles	Feature Structure	Score	Carcinogen in training set

ECFP_6	-1846894271	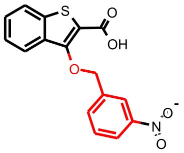 [*]OC[c]1:[cH]:[cH]:[cH]:[c]([*]):[cH]:1	0.424	1 out of 1
ECFP_6	-1845486197	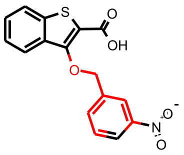 [*]OC[c]1:[cH]:[cH]:[*]:[c]([*]):[cH]:1	0.212	1 out of 2
ECFP_6	914325265	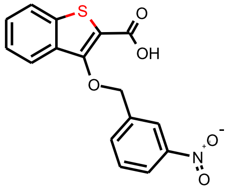 [*]:s:[*]	0.127	4 out of 11

Top Features for negative contribution

Fingerprint	Bit/Smiles	Feature Structure	Score	Carcinogen in training set
ECFP_6	2007300961	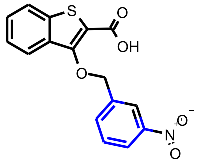 [*][c]1:[*]:[c]([*]):[cH]:[cH]:[cH]:1	-0.652	5 out of 34
ECFP_6	2025485523	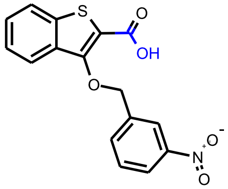 [*]C(=[*])O	-0.506	8 out of 45

ECFP_6	770547857	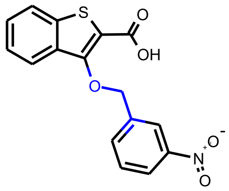<br>[*]OC[c](:[*]):[*]	-0.242	1 out of 5

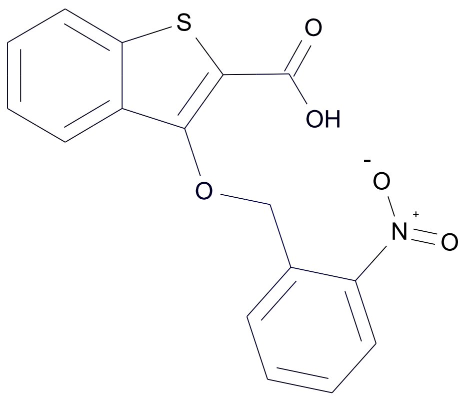

$C_{16}H_{11}NO_5S$
Molecular Weight: 329.32724
ALogP: 4.074
Rotatable Bonds: 5
Acceptors: 5
Donors: 1

Model Prediction

Prediction: Non-Carcinogen
Probability: 0.215
Enrichment: 0.670
Bayesian Score: -3.593
Mahalanobis Distance: 12.614
Mahalanobis Distance p-value: 0.00185

Prediction: Positive if the Bayesian score is above the estimated best cutoff value from minimizing the false positive and false negative rate.
Probability: The esimated probability that the sample is in the positive category. This assumes that the Bayesian score follows a normal distribution and is different from the prediction using a cutoff.
Enrichment: An estimate of enrichment, that is, the increased likelihood (versus random) of this sample being in the category.
Bayesian Score: The standard Laplacian-modified Bayesian score.
Mahalanobis Distance: The Mahalanobis distance (MD) is the distance to the center of the training data. The larger the MD, the less trustworthy the prediction.
Mahalanobis Distance p-value: The p-value gives the fraction of training data with an MD greater than or equal to the one for the given sample, assuming normally distributed data. The smaller the p-value, the less trustworthy the prediciton. For highly non-normal X properties (e.g., fingerprints), the MD p-value is wildly inaccurate.

Structural Similar Compounds

Name	Omeprazole	Suprofen	Lansoprazole
Structure	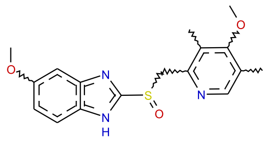	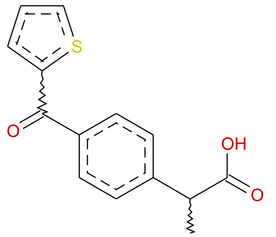	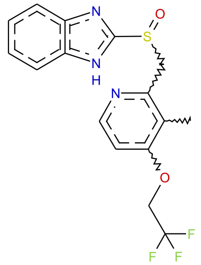
Actual Endpoint	Non-Carcinogen	Carcinogen	Carcinogen
Predicted Endpoint	Non-Carcinogen	Carcinogen	Carcinogen
Distance	0.666	0.689	0.691
Reference	US FDA (Centre for Drug Eval.& Res./Off. Testing & Res.) Sept. 1997	US FDA (Centre for Drug Eval.& Res./Off. Testing & Res.) Sept. 1997	US FDA (Centre for Drug Eval.& Res./Off. Testing & Res.) Sept. 1997

Model Applicability

Unknown features are fingerprint features in the query molecule, but not found in the training set.

1. All properties and OPS components are within expected ranges.
2. Unknown ECFP_2 feature: 1043790491: [*][N+](=[*])[*]
3. Unknown ECFP_2 feature: 781519895: [*][O-]
4. Unknown ECFP_2 feature: -1670580914: [*]C(=[*])[c]1:s:[*]:[*]:[c]:1[*]
5. Unknown ECFP_2 feature: 1895035276: [*]:[cH]:[c]1:s:[*]:[*]:[c]:1:[*]
6. Unknown ECFP_2 feature: -1956535100: [*][c](:[*]):[c](:[cH]:[*])[N+](=[*])[*]
7. Unknown ECFP_2 feature: -215026467: [*]:[c](:[*])[N+](=O)[O-]
8. Unknown ECFP_2 feature: 2104376220: [*][N+](=O)[*]
9. Unknown ECFP_2 feature: -659271057: [*][N+](=[*])[O-]

Feature Contribution

Top features for positive contribution				
**Fingerprint**	**Bit/Smiles**	**Feature Structure**	**Score**	**Carcinogen in training set**

ECFP_6	914325265	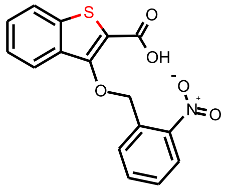 [*]:s:[*]	0.127	4 out of 11

Top Features for negative contribution

Fingerprint	Bit/Smiles	Feature Structure	Score	Carcinogen in training set
ECFP_6	2025485523	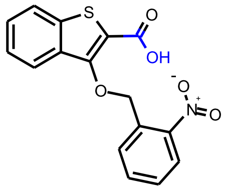 [*]C(=[*])O	-0.506	8 out of 45
ECFP_6	-358687393	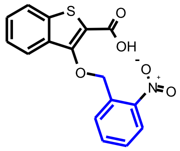 [*]C[c]1:[cH]:[cH]:[cH]:[cH]:[c]:1[*]	-0.482	0 out of 2
ECFP_6	770547857	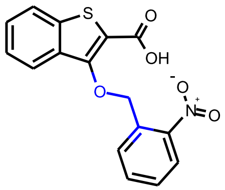 [*]OC[c](:[*]):[*]	-0.242	1 out of 5

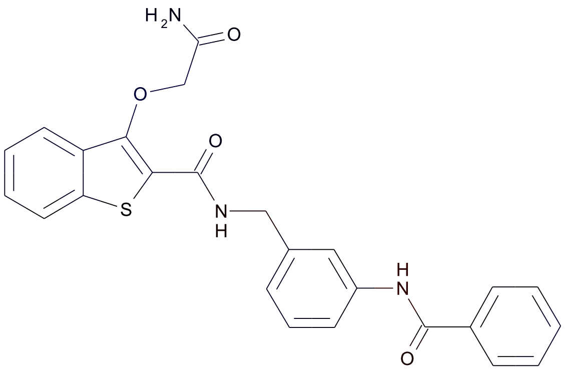

$C_{25}H_{21}N_3O_4S$
Molecular Weight: 459.51694
ALogP: 3.396
Rotatable Bonds: 8
Acceptors: 4
Donors: 3

Model Prediction

Prediction: Non-Carcinogen
Probability: 0.214
Enrichment: 0.668
Bayesian Score: -3.683
Mahalanobis Distance: 18.874
Mahalanobis Distance p-value: 1.77e-015

Prediction: Positive if the Bayesian score is above the estimated best cutoff value from minimizing the false positive and false negative rate.
Probability: The esimated probability that the sample is in the positive category. This assumes that the Bayesian score follows a normal distribution and is different from the prediction using a cutoff.
Enrichment: An estimate of enrichment, that is, the increased likelihood (versus random) of this sample being in the category.
Bayesian Score: The standard Laplacian-modified Bayesian score.
Mahalanobis Distance: The Mahalanobis distance (MD) is the distance to the center of the training data. The larger the MD, the less trustworthy the prediction.
Mahalanobis Distance p-value: The p-value gives the fraction of training data with an MD greater than or equal to the one for the given sample, assuming normally distributed data. The smaller the p-value, the less trustworthy the prediciton. For highly non-normal X properties (e.g., fingerprints), the MD p-value is wildly inaccurate.

Structural Similar Compounds

Name	Glyburide	Glimepride	Fluvastatin
Structure	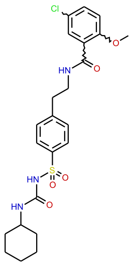	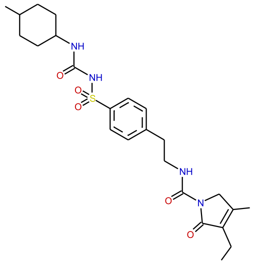	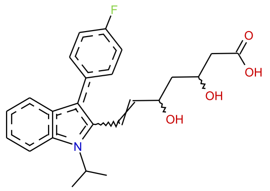
Actual Endpoint	Non-Carcinogen	Carcinogen	Non-Carcinogen
Predicted Endpoint	Non-Carcinogen	Carcinogen	Non-Carcinogen
Distance	0.597	0.601	0.644
Reference	US FDA (Centre for Drug Eval.& Res./Off. Testing & Res.) Sept. 1997	US FDA (Centre for Drug Eval.& Res./Off. Testing & Res.) Sept. 1997	US FDA (Centre for Drug Eval.& Res./Off. Testing & Res.) Sept. 1997

Model Applicability

Unknown features are fingerprint features in the query molecule, but not found in the training set.

1. All properties and OPS components are within expected ranges.
2. Unknown ECFP_2 feature: -1670580914: [*]C(=[*])[c]1:s:[*]:[*]:[c]:1[*]
3. Unknown ECFP_2 feature: 1895035276: [*]:[cH]:[c]1:s:[*]:[*]:[c]:1:[*]

Feature Contribution

Top features for positive contribution				
**Fingerprint**	**Bit/Smiles**	**Feature Structure**	**Score**	**Carcinogen in training set**
ECFP_6	769925792	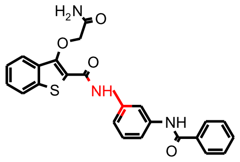 [*]NC[c](:[*]):[*]	0.617	2 out of 2

ECFP_6	-223149939	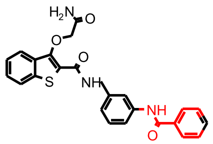 [*]NC(=O)[c]1:[cH]:[cH]:[*]:[cH]:[cH]:1	0.442	2 out of 3
ECFP_6	-177077903	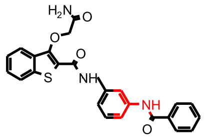 [*]N[c](:[cH]:[*]):[cH]:[*]	0.279	4 out of 9

Top Features for negative contribution

**Fingerprint**	**Bit/Smiles**	**Feature Structure**	**Score**	**Carcinogen in training set**
ECFP_6	2007300961	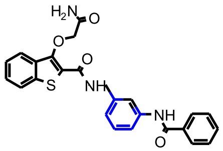 [*][c]1:[*]:[c]([*]):[cH]:[cH]:[cH]:1	-0.652	5 out of 34
ECFP_6	-1686813061	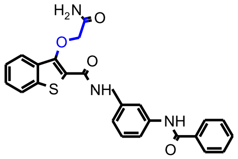 [*]OCC(=[*])[*]	-0.482	0 out of 2
ECFP_6	1451403962	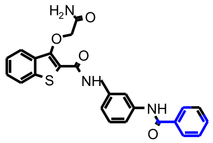 [*]C(=[*])[c]1:[cH]:[*]:[cH]:[cH]:[cH]:1	-0.459	1 out of 7

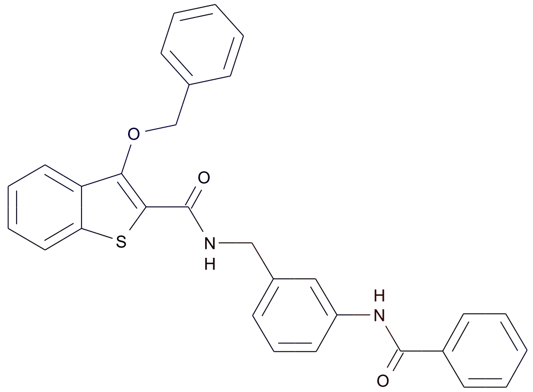

$C_{30}H_{24}N_2O_3S$
Molecular Weight: 492.58816
ALogP: 6.126
Rotatable Bonds: 8
Acceptors: 3
Donors: 2

Model Prediction

Prediction: Non-Carcinogen
Probability: 0.217
Enrichment: 0.678
Bayesian Score: -3.277
Mahalanobis Distance: 18.402
Mahalanobis Distance p-value: 2.05e-014

Prediction: Positive if the Bayesian score is above the estimated best cutoff value from minimizing the false positive and false negative rate.
Probability: The esimated probability that the sample is in the positive category. This assumes that the Bayesian score follows a normal distribution and is different from the prediction using a cutoff.
Enrichment: An estimate of enrichment, that is, the increased likelihood (versus random) of this sample being in the category.
Bayesian Score: The standard Laplacian-modified Bayesian score.
Mahalanobis Distance: The Mahalanobis distance (MD) is the distance to the center of the training data. The larger the MD, the less trustworthy the prediction.
Mahalanobis Distance p-value: The p-value gives the fraction of training data with an MD greater than or equal to the one for the given sample, assuming normally distributed data. The smaller the p-value, the less trustworthy the prediciton. For highly non-normal X properties (e.g., fingerprints), the MD p-value is wildly inaccurate.

Structural Similar Compounds

Name	Terfenadine	Astemizole	Pimozide
Structure	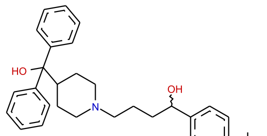	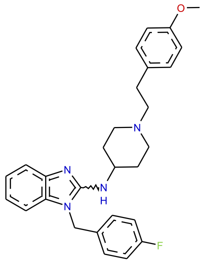	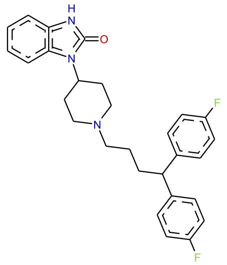
Actual Endpoint	Non-Carcinogen	Non-Carcinogen	Carcinogen
Predicted Endpoint	Non-Carcinogen	Non-Carcinogen	Carcinogen
Distance	0.639	0.698	0.722
Reference	US FDA (Centre for Drug Eval.& Res./Off. Testing & Res.) Sept. 1997	US FDA (Centre for Drug Eval.& Res./Off. Testing & Res.) Sept. 1997	US FDA (Centre for Drug Eval.& Res./Off. Testing & Res.) Sept. 1997

Model Applicability

Unknown features are fingerprint features in the query molecule, but not found in the training set.

1. All properties and OPS components are within expected ranges.
2. Unknown ECFP_2 feature: -1670580914: [*]C(=[*])[c]1:s:[*]:[*]:[c]:1[*]
3. Unknown ECFP_2 feature: 1895035276: [*]:[cH]:[c]1:s:[*]:[*]:[c]:1:[*]

Feature Contribution

Top features for positive contribution

Fingerprint	Bit/Smiles	Feature Structure	Score	Carcinogen in training set
ECFP_6	769925792	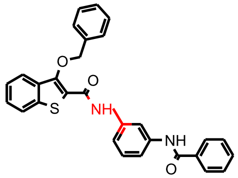 [*]NC[c](:[*]):[*]	0.617	2 out of 2

ECFP_6	-223149939	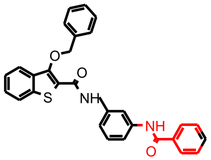 [*]NC(=O)[c]1:[cH]:[cH]:[*]:[cH]:[cH]:1	0.442	2 out of 3
ECFP_6	-177077903	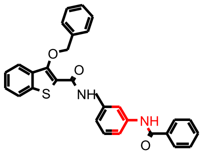 [*]N[c](:[cH]:[*]):[cH]:[*]	0.279	4 out of 9

Top Features for negative contribution

Fingerprint	Bit/Smiles	Feature Structure	Score	Carcinogen in training set
ECFP_6	2007300961	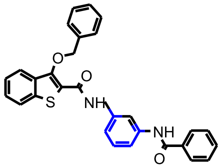 [*][c]1:[*]:[c]([*]):[cH]:[cH]:[cH]:1	-0.652	5 out of 34
ECFP_6	1451403962	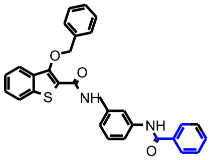 [*]C(=[*])[c]1:[cH]:[*]:[cH]:[cH]:[cH]:1	-0.459	1 out of 7
ECFP_6	1430169877	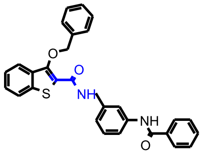 [*]NC(=O)[c](:[*]):[*]	-0.287	3 out of 14

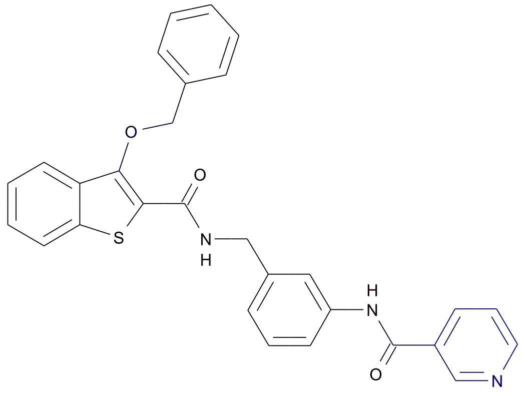

$C_{29}H_{23}N_3O_3S$
Molecular Weight: 493.57622
ALogP: 4.976
Rotatable Bonds: 8
Acceptors: 4
Donors: 2

Model Prediction

Prediction: Non-Carcinogen
Probability: 0.205
Enrichment: 0.639
Bayesian Score: -6.039
Mahalanobis Distance: 17.960
Mahalanobis Distance p-value: 1.98e-013

Prediction: Positive if the Bayesian score is above the estimated best cutoff value from minimizing the false positive and false negative rate.
Probability: The esimated probability that the sample is in the positive category. This assumes that the Bayesian score follows a normal distribution and is different from the prediction using a cutoff.
Enrichment: An estimate of enrichment, that is, the increased likelihood (versus random) of this sample being in the category.
Bayesian Score: The standard Laplacian-modified Bayesian score.
Mahalanobis Distance: The Mahalanobis distance (MD) is the distance to the center of the training data. The larger the MD, the less trustworthy the prediction.
Mahalanobis Distance p-value: The p-value gives the fraction of training data with an MD greater than or equal to the one for the given sample, assuming normally distributed data. The smaller the p-value, the less trustworthy the prediciton. For highly non-normal X properties (e.g., fingerprints), the MD p-value is wildly inaccurate.

Structural Similar Compounds

Name	Glyburide	Fluvastatin	Flecainide
Structure	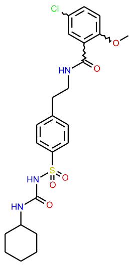	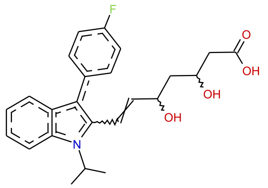	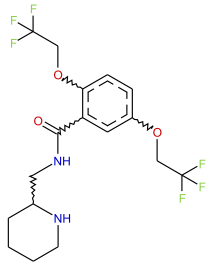
Actual Endpoint	Non-Carcinogen	Non-Carcinogen	Non-Carcinogen
Predicted Endpoint	Non-Carcinogen	Non-Carcinogen	Non-Carcinogen
Distance	0.643	0.674	0.674
Reference	US FDA (Centre for Drug Eval.& Res./Off. Testing & Res.) Sept. 1997	US FDA (Centre for Drug Eval.& Res./Off. Testing & Res.) Sept. 1997	US FDA (Centre for Drug Eval.& Res./Off. Testing & Res.) Sept. 1997

Model Applicability

Unknown features are fingerprint features in the query molecule, but not found in the training set.

1. All properties and OPS components are within expected ranges.
2. Unknown ECFP_2 feature: -1670580914: [*]C(=[*])[c]1:s:[*]:[*]:[c]:1[*]
3. Unknown ECFP_2 feature: 1895035276: [*]:[cH]:[c]1:s:[*]:[*]:[c]:1:[*]

Feature Contribution

Top features for positive contribution

Fingerprint	Bit/Smiles	Feature Structure	Score	Carcinogen in training set
ECFP_6	769925792	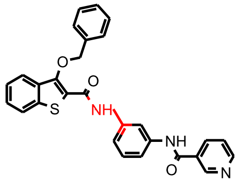 [*]NC[c](:[*]):[*]	0.617	2 out of 2

ECFP_6	-177077903	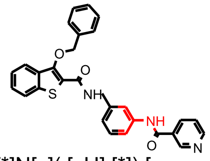<br>[*]N[c](:[cH]:[*]):[c<br>H]:[*]	0.279	4 out of 9
ECFP_6	894876384	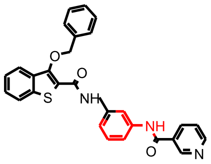<br>[*]N[c]1:[cH]:[*]:[cH<br>]:[cH]:[cH]:1	0.212	1 out of 2

Top Features for negative contribution

Fingerprint	Bit/Smiles	Feature Structure	Score	Carcinogen in training set
ECFP_6	2013347047	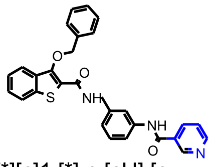<br>[*][c]1:[*]:n:[cH]:[c<br>H]:[cH]:1	-0.805	0 out of 4
ECFP_6	2007300961	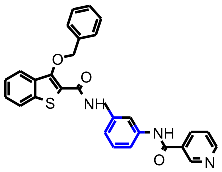<br>[*][c]1:[*]:[c]([*]):<br>[cH]:[cH]:[cH]:1	-0.652	5 out of 34
ECFP_6	764951226	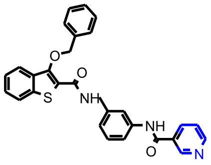<br>[*]1:[cH]:[cH]:[cH]:n<br>:[cH]:1	-0.482	0 out of 2

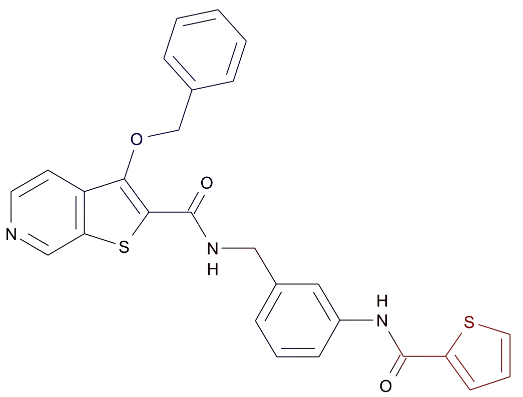

$C_{27}H_{21}N_3O_3S_2$
Molecular Weight: 499.60394
ALogP: 4.929
Rotatable Bonds: 8
Acceptors: 4
Donors: 2

Model Prediction

Prediction: Carcinogen
Probability: 0.249
Enrichment: 0.778
Bayesian Score: -0.738
Mahalanobis Distance: 16.656
Mahalanobis Distance p-value: 1.28e-010

Prediction: Positive if the Bayesian score is above the estimated best cutoff value from minimizing the false positive and false negative rate.
Probability: The esimated probability that the sample is in the positive category. This assumes that the Bayesian score follows a normal distribution and is different from the prediction using a cutoff.
Enrichment: An estimate of enrichment, that is, the increased likelihood (versus random) of this sample being in the category.
Bayesian Score: The standard Laplacian-modified Bayesian score.
Mahalanobis Distance: The Mahalanobis distance (MD) is the distance to the center of the training data. The larger the MD, the less trustworthy the prediction.
Mahalanobis Distance p-value: The p-value gives the fraction of training data with an MD greater than or equal to the one for the given sample, assuming normally distributed data. The smaller the p-value, the less trustworthy the prediciton. For highly non-normal X properties (e.g., fingerprints), the MD p-value is wildly inaccurate.

Structural Similar Compounds

Name	Glyburide	Glimepride	Fluvastatin
Structure	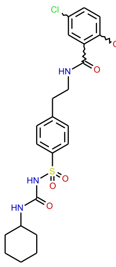	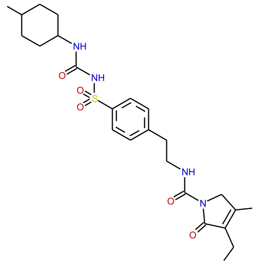	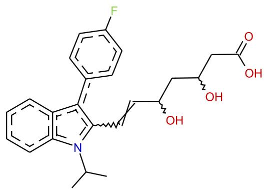
Actual Endpoint	Non-Carcinogen	Carcinogen	Non-Carcinogen
Predicted Endpoint	Non-Carcinogen	Carcinogen	Non-Carcinogen
Distance	0.642	0.675	0.713
Reference	US FDA (Centre for Drug Eval.& Res./Off. Testing & Res.) Sept. 1997	US FDA (Centre for Drug Eval.& Res./Off. Testing & Res.) Sept. 1997	US FDA (Centre for Drug Eval.& Res./Off. Testing & Res.) Sept. 1997

Model Applicability

Unknown features are fingerprint features in the query molecule, but not found in the training set.

1. All properties and OPS components are within expected ranges.
2. Unknown ECFP_2 feature: 1895035276: [*]:[cH]:[c]1:s:[*]:[*]:[c]:1:[*]
3. Unknown ECFP_2 feature: -1670580914: [*]C(=[*])[c]1:s:[*]:[*]:[c]:1[*]

Feature Contribution

Top features for positive contribution

Fingerprint	Bit/Smiles	Feature Structure	Score	Carcinogen in training set
ECFP_6	769925792	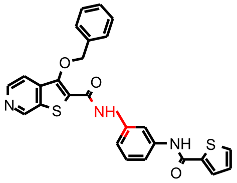 [*]NC[c](:[*]):[*]	0.617	2 out of 2

ECFP_6	706235072	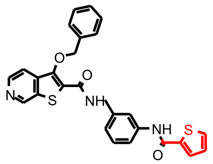 [*]C(=[*])[c]1:[cH]:[cH]:[cH]:s:1	0.424	1 out of 1
ECFP_6	173951371	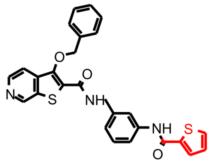 [*]C(=[*])[c]1:[cH]:[cH]:[cH]:s:1	0.424	1 out of 1
**Top Features for negative contribution**				
**Fingerprint**	**Bit/Smiles**	**Feature Structure**	**Score**	**Carcinogen in training set**
ECFP_6	2007300961	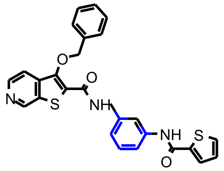 [*][c]1:[*]:[c]([*]): [cH]:[cH]:[cH]:1	-0.652	5 out of 34
ECFP_6	1430169877	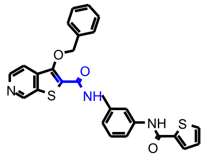 [*]NC(=O)[c](:[*]):[*]	-0.287	3 out of 14
ECFP_6	-1897063316	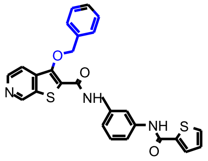 [*]OC[c]1:[cH]:[cH]:[*]:[cH]:[cH]:1	-0.270	0 out of 1

Co-crystallized ligand

TOPKAT_Mouse_Female_FDA_None_vs_Carcinogen

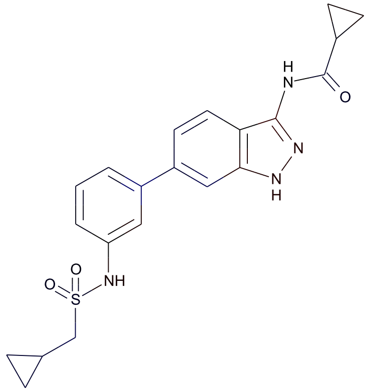

$C_{21}H_{22}N_4O_3S$

Molecular Weight: 410.48938

ALogP: 3.14

Rotatable Bonds: 7

Acceptors: 4

Donors: 3

Model Prediction

Prediction: Non-Carcinogen

Probability: 0.222

Enrichment: 0.691

Bayesian Score: -2.809

Mahalanobis Distance: 12.697

Mahalanobis Distance p-value: 0.00143

Prediction: Positive if the Bayesian score is above the estimated best cutoff value from minimizing the false positive and false negative rate.
Probability: The esimated probability that the sample is in the positive category. This assumes that the Bayesian score follows a normal distribution and is different from the prediction using a cutoff.
Enrichment: An estimate of enrichment, that is, the increased likelihood (versus random) of this sample being in the category.
Bayesian Score: The standard Laplacian-modified Bayesian score.
Mahalanobis Distance: The Mahalanobis distance (MD) is the distance to the center of the training data. The larger the MD, the less trustworthy the prediction.
Mahalanobis Distance p-value: The p-value gives the fraction of training data with an MD greater than or equal to the one for the given sample, assuming normally distributed data. The smaller the p-value, the less trustworthy the prediciton. For highly non-normal X properties (e.g., fingerprints), the MD p-value is wildly inaccurate.

Structural Similar Compounds

Name	Torsemide	Bicalutamide	Glimepride
Structure	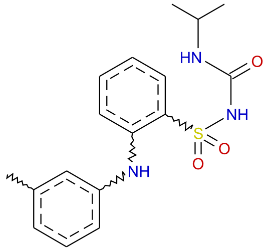	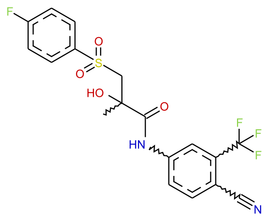	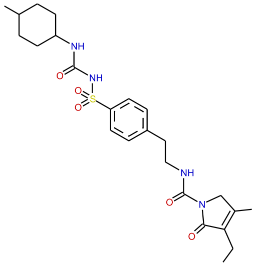
Actual Endpoint	Non-Carcinogen	Non-Carcinogen	Carcinogen
Predicted Endpoint	Non-Carcinogen	Non-Carcinogen	Carcinogen
Distance	0.611	0.630	0.633
Reference	US FDA (Centre for Drug Eval.& Res./Off. Testing & Res.) Sept. 1997	US FDA (Centre for Drug Eval.& Res./Off. Testing & Res.) Sept. 1997	US FDA (Centre for Drug Eval.& Res./Off. Testing & Res.) Sept. 1997

Model Applicability

Unknown features are fingerprint features in the query molecule, but not found in the training set.

1. All properties and OPS components are within expected ranges.
2. Unknown ECFP_2 feature: -857146788: [*]C(=[*])C1CC1
3. Unknown ECFP_2 feature: 600440273: [*][c]1:[*]:[*]:[nH]:n:1
4. Unknown ECFP_2 feature: -1341194584: [*]S(=[*])(=[*])CC1[*][*]1

Feature Contribution

Top features for positive contribution				
**Fingerprint**	**Bit/Smiles**	**Feature Structure**	**Score**	**Carcinogen in training set**
ECFP_6	2082767335	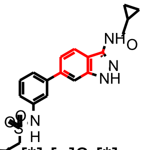 [*][c]1:[*]:[*]:[c]2:[*]:[*]:[c]([*]):[c]:2:[cH]:[cH]:1	0.617	2 out of 2

ECFP_6	1049768340	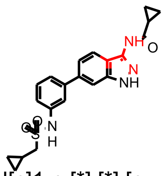 [*]N[c]1:n:[*]:[*]:[c]:1:[*]	0.424	1 out of 1
ECFP_6	-177077903	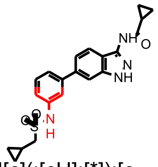 [*]N[c](:[cH]:[*]):[cH]:[*]	0.279	4 out of 9

Top Features for negative contribution

Fingerprint	Bit/Smiles	Feature Structure	Score	Carcinogen in training set
ECFP_6	-219423964	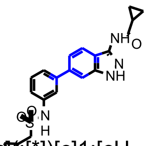 [*]:[c](:[*])[c]1:[cH]:[*]:[c](:[*]):[cH]:[cH]:1	-0.935	0 out of 5
ECFP_6	-2090328339	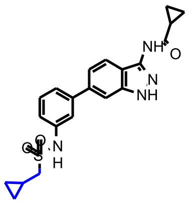 [*]CC1CC1	-0.657	0 out of 3
ECFP_6	2007300961	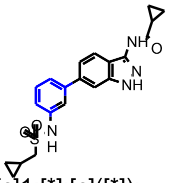 [*][c]1:[*]:[c]([*]):[cH]:[cH]:[cH]:1	-0.652	5 out of 34

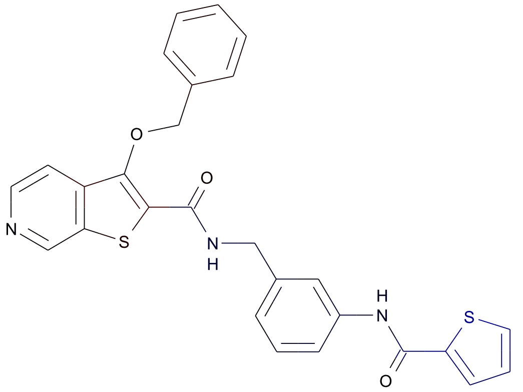

$C_{27}H_{21}N_3O_3S_2$
Molecular Weight: 499.60394
ALogP: 4.929
Rotatable Bonds: 8
Acceptors: 4
Donors: 2

Model Prediction

Prediction: Single-Carcinogen
Probability: 0.280
Enrichment: 0.683
Bayesian Score: -4.046
Mahalanobis Distance: 12.931
Mahalanobis Distance p-value: 6.28e-005

Prediction: Positive if the Bayesian score is above the estimated best cutoff value from minimizing the false positive and false negative rate.
Probability: The esimated probability that the sample is in the positive category. This assumes that the Bayesian score follows a normal distribution and is different from the prediction using a cutoff.
Enrichment: An estimate of enrichment, that is, the increased likelihood (versus random) of this sample being in the category.
Bayesian Score: The standard Laplacian-modified Bayesian score.
Mahalanobis Distance: The Mahalanobis distance (MD) is the distance to the center of the training data. The larger the MD, the less trustworthy the prediction.
Mahalanobis Distance p-value: The p-value gives the fraction of training data with an MD greater than or equal to the one for the given sample, assuming normally distributed data. The smaller the p-value, the less trustworthy the prediciton. For highly non-normal X properties (e.g., fingerprints), the MD p-value is wildly inaccurate.

Structural Similar Compounds

Name	Glimepride	Simvastatin	Lovastatin
Structure	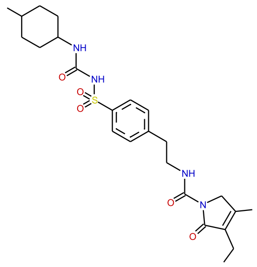	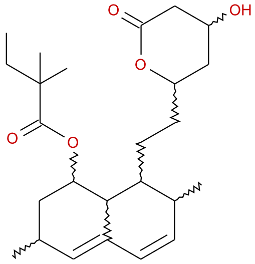	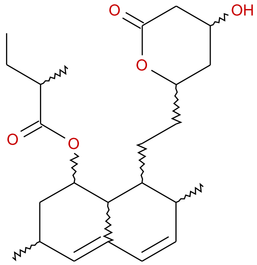
Actual Endpoint	Single-Carcinogen	Multiple-Carcinogen	Multiple-Carcinogen
Predicted Endpoint	Single-Carcinogen	Multiple-Carcinogen	Multiple-Carcinogen
Distance	0.689	0.769	0.778
Reference	US FDA (Centre for Drug Eval.& Res./Off. Testing & Res.) Sept. 1997	US FDA (Centre for Drug Eval.& Res./Off. Testing & Res.) Sept. 1997	US FDA (Centre for Drug Eval.& Res./Off. Testing & Res.) Sept. 1997

Model Applicability

Unknown features are fingerprint features in the query molecule, but not found in the training set.

1. All properties and OPS components are within expected ranges.
2. Unknown ECFP_2 feature: 1895035276: [*]:[cH]:[c]1:s:[*]:[*]:[c]:1:[*]
3. Unknown ECFP_2 feature: -1670580914: [*]C(=[*])[c]1:s:[*]:[*]:[c]:1[*]

Feature Contribution

Top features for positive contribution

Fingerprint	Bit/Smiles	Feature Structure	Score	Multiple-Carcinogen in training set
ECFP_4	-1531301414	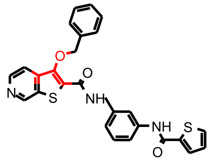 [*]O[c]1:[c]([*]):[*]:[*]:[c]:1:[*]	0.351	1 out of 1

ECFP_4	85262808	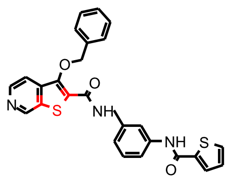 [*][c]1:[*]:[*]:[c](:[*]):s:1	0.351	1 out of 1
ECFP_4	894876384	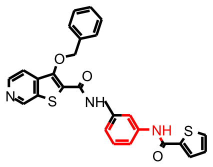 [*]N[c]1:[cH]:[*]:[cH]:[cH]:[cH]:1	0.351	1 out of 1
**Top Features for negative contribution**				
**Fingerprint**	**Bit/Smiles**	**Feature Structure**	**Score**	**Multiple-Carcinogen in training set**
ECFP_4	497523368	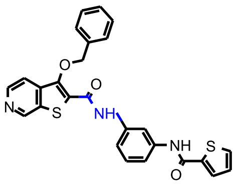 [*]CNC(=[*])[*]	-0.968	0 out of 4
ECFP_4	-1255706725	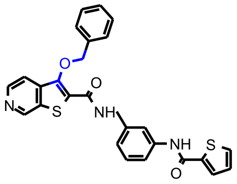 [*]CO[c](:[*]):[*]	-0.657	1 out of 7
ECFP_4	-1426923364	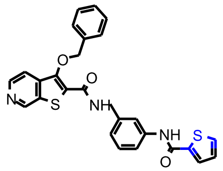 [*][c]1:[*]:[*]:[cH]:s:1	-0.597	0 out of 2

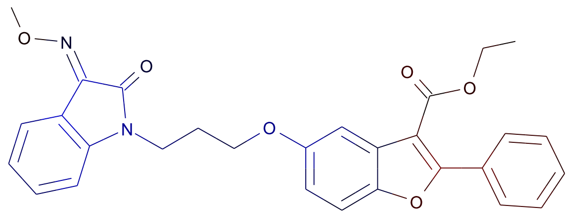

$C_{29}H_{26}N_2O_6$
Molecular Weight: 498.52653
ALogP: 4.994
Rotatable Bonds: 10
Acceptors: 6
Donors: 0

Model Prediction

Prediction: Non-Carcinogen
Probability: 0.205
Enrichment: 0.696
Bayesian Score: -4.681
Mahalanobis Distance: 15.420
Mahalanobis Distance p-value: 1.38e-008

Prediction: Positive if the Bayesian score is above the estimated best cutoff value from minimizing the false positive and false negative rate.
Probability: The esimated probability that the sample is in the positive category. This assumes that the Bayesian score follows a normal distribution and is different from the prediction using a cutoff.
Enrichment: An estimate of enrichment, that is, the increased likelihood (versus random) of this sample being in the category.
Bayesian Score: The standard Laplacian-modified Bayesian score.
Mahalanobis Distance: The Mahalanobis distance (MD) is the distance to the center of the training data. The larger the MD, the less trustworthy the prediction.
Mahalanobis Distance p-value: The p-value gives the fraction of training data with an MD greater than or equal to the one for the given sample, assuming normally distributed data. The smaller the p-value, the less trustworthy the prediciton. For highly non-normal X properties (e.g., fingerprints), the MD p-value is wildly inaccurate.

Structural Similar Compounds

Name	Nefazodone	Ketoconazole	Verapamil
Structure	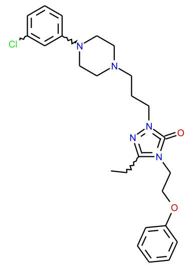	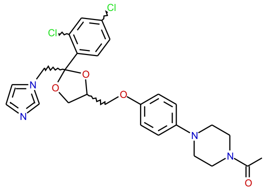	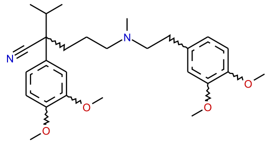
Actual Endpoint	Non-Carcinogen	Non-Carcinogen	Non-Carcinogen
Predicted Endpoint	Non-Carcinogen	Non-Carcinogen	Non-Carcinogen
Distance	0.576	0.670	0.671
Reference	US FDA (Centre for Drug Eval.& Res./Off. Testing & Res.) Sept. 1997	US FDA (Centre for Drug Eval.& Res./Off. Testing & Res.) Sept. 1997	US FDA (Centre for Drug Eval.& Res./Off. Testing & Res.) Sept. 1997

Model Applicability

Unknown features are fingerprint features in the query molecule, but not found in the training set.

1. All properties and OPS components are within expected ranges.

Feature Contribution

Top features for positive contribution

Fingerprint	Bit/Smiles	Feature Structure	Score	Carcinogen in training set
FCFP_6	757274319	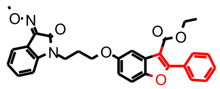 [*][c]1:[*]:[*]:o:[c]:1[c]2:[cH]:[cH]:[cH]:[cH]:[cH]:2	0.676	2 out of 2

FCFP_6	391786003	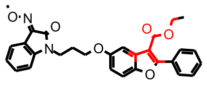<br>[*]COC(=O)[c]1:[c]([*<br>]):[*]:[*]:[c]:1:[*]	0.668	3 out of 4
FCFP_6	-822674211	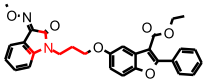<br>[*]CCCN1C(=[*])[*][*]<br>:[c]1:[*]	0.517	2 out of 3

Top Features for negative contribution

Fingerprint	Bit/Smiles	Feature Structure	Score	Carcinogen in training set
FCFP_6	-70433048	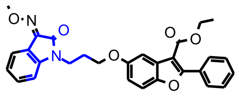<br>[*]CCN1C(=O)C(=[*])[c<br>](:[*]):[c]1:[cH]:[*<br>]	-0.839	0 out of 5
FCFP_6	1674955425	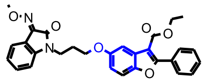<br>[*]O[c]1:[cH]:[*]:[c]<br>2:[*]:[*]:[c]([*]):[<br>c]:2:[cH]:1	-0.719	0 out of 4
FCFP_6	564869443	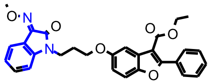<br>[*]\N=C/1\C(=[*])N([*<br>])[c]2:[cH]:[*]:[cH]<br>:[cH]:[c]1:2	-0.719	0 out of 4

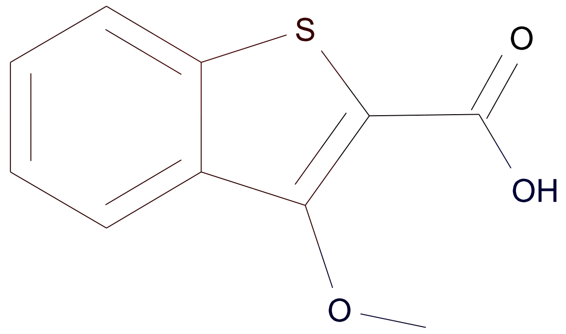

$C_{10}H_8O_3S$
Molecular Weight: 208.23372
ALogP: 2.596
Rotatable Bonds: 2
Acceptors: 3
Donors: 1

Structural Similar Compounds

Name	Thiabendazole	Aspirin	Ethionamide
Structure	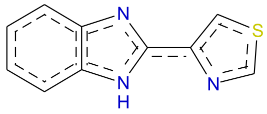	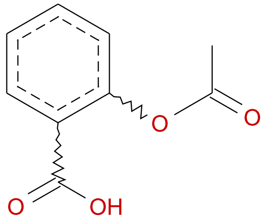	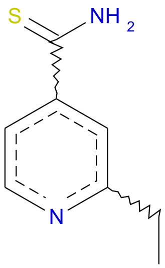
Actual Endpoint	Non-Carcinogen	Non-Carcinogen	Carcinogen
Predicted Endpoint	Non-Carcinogen	Non-Carcinogen	Carcinogen
Distance	0.549	0.568	0.579
Reference	US FDA (Centre for Drug Eval.& Res./Off. Testing & Res.) Sept. 1997	US FDA (Centre for Drug Eval.& Res./Off. Testing & Res.) Sept. 1997	US FDA (Centre for Drug Eval.& Res./Off. Testing & Res.) Sept. 1997

Model Applicability

Unknown features are fingerprint features in the query molecule, but not found in the training set.

1. All properties and OPS components are within expected ranges.

Feature Contribution

Top features for positive contribution

Fingerprint	Bit/Smiles	Feature Structure	Score	Carcinogen in training set
FCFP_6	-387072142	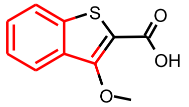 [*][c]1:[*]:[*]:[c]2:[cH]:[cH]:[cH]:[cH]:[c]:1:2	0.477	4 out of 8

Model Prediction

Prediction: Carcinogen
Probability: 0.320
Enrichment: 1.089
Bayesian Score: 0.523
Mahalanobis Distance: 13.977
Mahalanobis Distance p-value: 7.12e-006

Prediction: Positive if the Bayesian score is above the estimated best cutoff value from minimizing the false positive and false negative rate.
Probability: The esimated probability that the sample is in the positive category. This assumes that the Bayesian score follows a normal distribution and is different from the prediction using a cutoff.
Enrichment: An estimate of enrichment, that is, the increased likelihood (versus random) of this sample being in the category.
Bayesian Score: The standard Laplacian-modified Bayesian score.
Mahalanobis Distance: The Mahalanobis distance (MD) is the distance to the center of the training data. The larger the MD, the less trustworthy the prediction.
Mahalanobis Distance p-value: The p-value gives the fraction of training data with an MD greater than or equal to the one for the given sample, assuming normally distributed data. The smaller the p-value, the less trustworthy the prediciton. For highly non-normal X properties (e.g., fingerprints), the MD p-value is wildly inaccurate.

FCFP_6	-1320007763	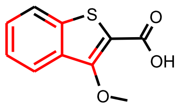<br>[*][c]1:[*]:[*]:[c]2:<br>[*]:[cH]:[cH]:[cH]:[<br>c]:1:2	0.348	6 out of 15
FCFP_6	-1539132615	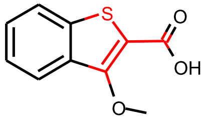<br>[*]C(=[*])[c]1:s:[*]:<br>[*]:[c]:1[*]	0.328	19 out of 51
**Top Features for negative contribution**				
**Fingerprint**	**Bit/Smiles**	**Feature Structure**	**Score**	**Carcinogen in training set**
FCFP_6	-548632217	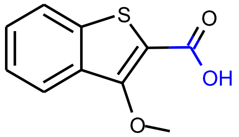<br>[*]C(=[*])O	-0.383	9 out of 52
FCFP_6	-1549222613	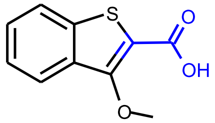<br>[*]:[c](:[*])C(=O)O	-0.351	1 out of 7
FCFP_6	7	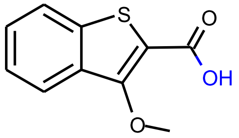<br>[*]O	-0.308	15 out of 79

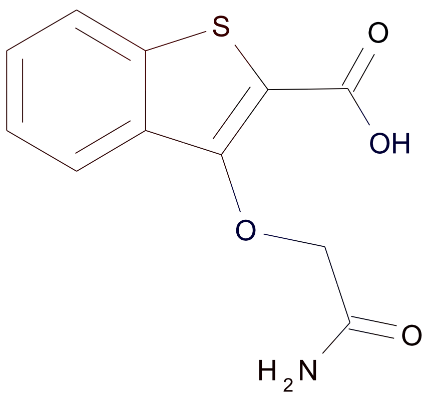

$C_{11}H_9NO_4S$
Molecular Weight: 251.25846
ALogP: 1.45
Rotatable Bonds: 4
Acceptors: 4
Donors: 2

Model Prediction

Prediction: Carcinogen
Probability: 0.321
Enrichment: 1.091
Bayesian Score: 0.549
Mahalanobis Distance: 14.730
Mahalanobis Distance p-value: 3.05e-007

Prediction: Positive if the Bayesian score is above the estimated best cutoff value from minimizing the false positive and false negative rate.
Probability: The esimated probability that the sample is in the positive category. This assumes that the Bayesian score follows a normal distribution and is different from the prediction using a cutoff.
Enrichment: An estimate of enrichment, that is, the increased likelihood (versus random) of this sample being in the category.
Bayesian Score: The standard Laplacian-modified Bayesian score.
Mahalanobis Distance: The Mahalanobis distance (MD) is the distance to the center of the training data. The larger the MD, the less trustworthy the prediction.
Mahalanobis Distance p-value: The p-value gives the fraction of training data with an MD greater than or equal to the one for the given sample, assuming normally distributed data. The smaller the p-value, the less trustworthy the prediciton. For highly non-normal X properties (e.g., fingerprints), the MD p-value is wildly inaccurate.

Structural Similar Compounds

Name	Nithiazide	Sulfisoxazole	Furothiazole
Structure	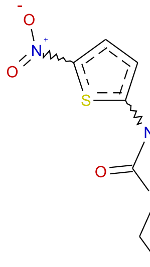	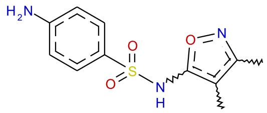	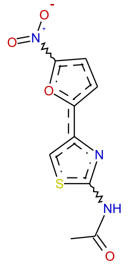
Actual Endpoint	Carcinogen	Non-Carcinogen	Carcinogen
Predicted Endpoint	Carcinogen	Carcinogen	Carcinogen
Distance	0.564	0.580	0.590
Reference	US FDA (Centre for Drug Eval.& Res./Off. Testing & Res.) Sept. 1997	US FDA (Centre for Drug Eval.& Res./Off. Testing & Res.) Sept. 1997	US FDA (Centre for Drug Eval.& Res./Off. Testing & Res.) Sept. 1997

Model Applicability

Unknown features are fingerprint features in the query molecule, but not found in the training set.

1. All properties and OPS components are within expected ranges.

Feature Contribution

Top features for positive contribution

Fingerprint	Bit/Smiles	Feature Structure	Score	Carcinogen in training set
FCFP_6	-387072142	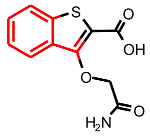 [*][c]1:[*]:[*]:[c]2:[cH]:[cH]:[cH]:[cH]:[c]:1:2	0.477	4 out of 8

FCFP_6	566058135	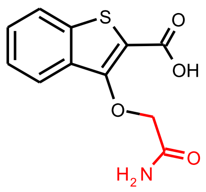 [*]CC(=O)N	0.447	17 out of 40
FCFP_6	-1320007763	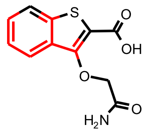 [*][c]1:[*]:[*]:[c]2:[*]:[cH]:[cH]:[cH]:[c]:1:2	0.348	6 out of 15
**Top Features for negative contribution**				
**Fingerprint**	**Bit/Smiles**	**Feature Structure**	**Score**	**Carcinogen in training set**
FCFP_6	-548632217	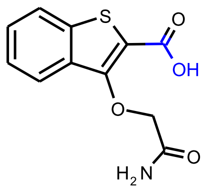 [*]C(=[*])O	-0.383	9 out of 52
FCFP_6	-1549222613	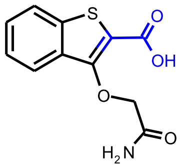 [*]:[c](:[*])C(=O)O	-0.351	1 out of 7
FCFP_6	7	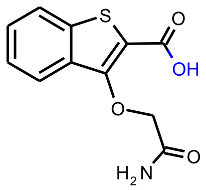 [*]O	-0.308	15 out of 79

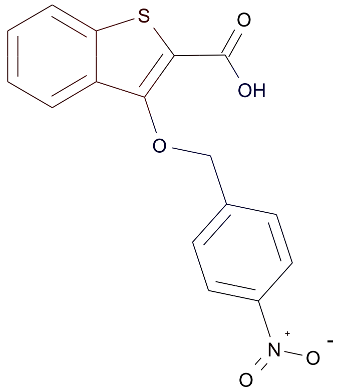

$C_{16}H_{11}NO_5S$
Molecular Weight: 329.32724
ALogP: 4.074
Rotatable Bonds: 5
Acceptors: 5
Donors: 1

Model Prediction

Prediction: Carcinogen

Probability: 0.280

Enrichment: 0.950

Bayesian Score: -1.007

Mahalanobis Distance: 15.871

Mahalanobis Distance p-value: 1.67e-009

Prediction: Positive if the Bayesian score is above the estimated best cutoff value from minimizing the false positive and false negative rate.
Probability: The esimated probability that the sample is in the positive category. This assumes that the Bayesian score follows a normal distribution and is different from the prediction using a cutoff.
Enrichment: An estimate of enrichment, that is, the increased likelihood (versus random) of this sample being in the category.
Bayesian Score: The standard Laplacian-modified Bayesian score.
Mahalanobis Distance: The Mahalanobis distance (MD) is the distance to the center of the training data. The larger the MD, the less trustworthy the prediction.
Mahalanobis Distance p-value: The p-value gives the fraction of training data with an MD greater than or equal to the one for the given sample, assuming normally distributed data. The smaller the p-value, the less trustworthy the prediciton. For highly non-normal X properties (e.g., fingerprints), the MD p-value is wildly inaccurate.

Structural Similar Compounds

Name	Omeprazole	Lansoprazole	Suprofen
Structure	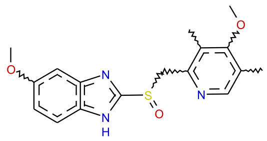	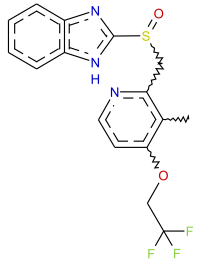	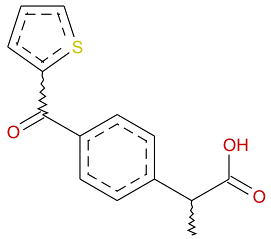
Actual Endpoint	Non-Carcinogen	Carcinogen	Carcinogen
Predicted Endpoint	Non-Carcinogen	Carcinogen	Carcinogen
Distance	0.635	0.654	0.667
Reference	US FDA (Centre for Drug Eval.& Res./Off. Testing & Res.) Sept. 1997	US FDA (Centre for Drug Eval.& Res./Off. Testing & Res.) Sept. 1997	US FDA (Centre for Drug Eval.& Res./Off. Testing & Res.) Sept. 1997

Model Applicability

Unknown features are fingerprint features in the query molecule, but not found in the training set.

1. All properties and OPS components are within expected ranges.
2. Unknown FCFP_2 feature: 5: [*][O-]
3. Unknown FCFP_2 feature: -828984032: [*][N+](=[*])[c](:[cH]:[*]):[cH]:[*]
4. Unknown FCFP_2 feature: -1338588315: [*]:[c](:[*])[N+](=O)[O-]
5. Unknown FCFP_2 feature: 1872392852: [*][N+](=O)[*]
6. Unknown FCFP_2 feature: 260476081: [*][N+](=[*])[O-]

Feature Contribution

Top features for positive contribution

Fingerprint	Bit/Smiles	Feature Structure	Score	Carcinogen in training set
FCFP_6	-387072142	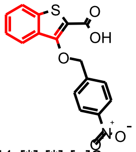 [*][c]1:[*]:[*]:[c]2:[cH]:[cH]:[cH]:[cH]:[c]:1:2	0.477	4 out of 8

FCFP_6	-1320007763	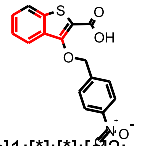<br>[*][c]1:[*]:[*]:[c]2:[*]:[cH]:[cH]:[cH]:[c]:1:2	0.348	6 out of 15
FCFP_6	-1539132615	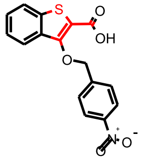<br>[*]C(=[*])[c]1:s:[*]:[*]:[c]:1[*]	0.328	19 out of 51

Top Features for negative contribution

**Fingerprint**	**Bit/Smiles**	**Feature Structure**	**Score**	**Carcinogen in training set**
FCFP_6	-548632217	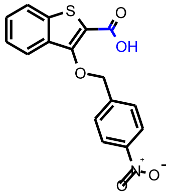<br>[*]C(=[*])O	-0.383	9 out of 52
FCFP_6	-1549222613	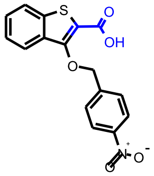<br>[*]:[c](:[*])C(=O)O	-0.351	1 out of 7
FCFP_6	907036844	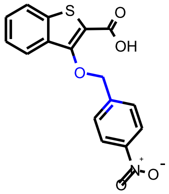<br>[*]OC[c](:[*]):[*]	-0.313	3 out of 17

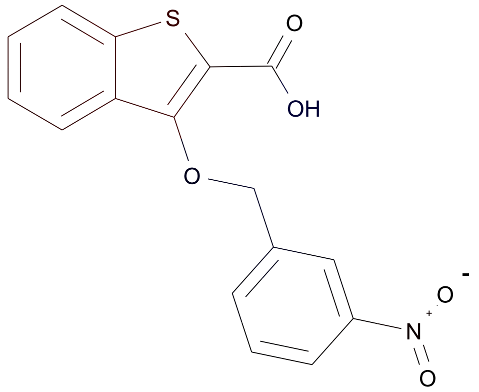

$C_{16}H_{11}NO_5S$
Molecular Weight: 329.32724
ALogP: 4.074
Rotatable Bonds: 5
Acceptors: 5
Donors: 1

Model Prediction

Prediction: Carcinogen
Probability: 0.286
Enrichment: 0.973
Bayesian Score: -0.737
Mahalanobis Distance: 15.871
Mahalanobis Distance p-value: 1.67e-009

Prediction: Positive if the Bayesian score is above the estimated best cutoff value from minimizing the false positive and false negative rate.
Probability: The esimated probability that the sample is in the positive category. This assumes that the Bayesian score follows a normal distribution and is different from the prediction using a cutoff.
Enrichment: An estimate of enrichment, that is, the increased likelihood (versus random) of this sample being in the category.
Bayesian Score: The standard Laplacian-modified Bayesian score.
Mahalanobis Distance: The Mahalanobis distance (MD) is the distance to the center of the training data. The larger the MD, the less trustworthy the prediction.
Mahalanobis Distance p-value: The p-value gives the fraction of training data with an MD greater than or equal to the one for the given sample, assuming normally distributed data. The smaller the p-value, the less trustworthy the prediciton. For highly non-normal X properties (e.g., fingerprints), the MD p-value is wildly inaccurate.

Structural Similar Compounds

Name	Omeprazole	Lansoprazole	Suprofen
Structure	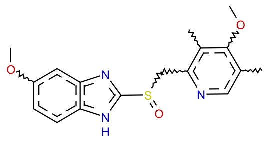	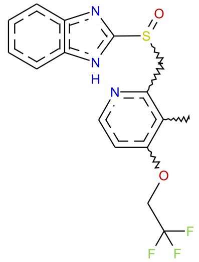	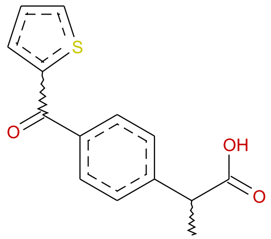
Actual Endpoint	Non-Carcinogen	Carcinogen	Carcinogen
Predicted Endpoint	Non-Carcinogen	Carcinogen	Carcinogen
Distance	0.637	0.656	0.670
Reference	US FDA (Centre for Drug Eval.& Res./Off. Testing & Res.) Sept. 1997	US FDA (Centre for Drug Eval.& Res./Off. Testing & Res.) Sept. 1997	US FDA (Centre for Drug Eval.& Res./Off. Testing & Res.) Sept. 1997

Model Applicability

Unknown features are fingerprint features in the query molecule, but not found in the training set.

1. All properties and OPS components are within expected ranges.
2. Unknown FCFP_2 feature: 5: [*][O-]
3. Unknown FCFP_2 feature: -828984032: [*][N+](=[*])[c](:[cH]:[*]):[cH]:[*]
4. Unknown FCFP_2 feature: -1338588315: [*]:[c](:[*])[N+](=O)[O-]
5. Unknown FCFP_2 feature: 1872392852: [*][N+](=O)[*]
6. Unknown FCFP_2 feature: 260476081: [*][N+](=[*])[O-]

Feature Contribution

Top features for positive contribution

Fingerprint	Bit/Smiles	Feature Structure	Score	Carcinogen in training set
FCFP_6	-387072142	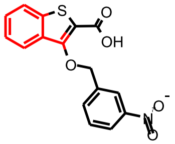 [*][c]1:[*]:[*]:[c]2:[cH]:[cH]:[cH]:[cH]:[c]:1:2	0.477	4 out of 8

FCFP_6	-1320007763	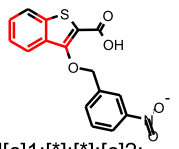 [*][c]1:[*]:[*]:[c]2:[*]:[cH]:[cH]:[cH]:[c]:1:2	0.348	6 out of 15
FCFP_6	-1539132615	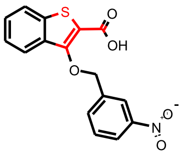 [*]C(=[*])[c]1:s:[*]:[*]:[c]:1[*]	0.328	19 out of 51
**Top Features for negative contribution**				
**Fingerprint**	**Bit/Smiles**	**Feature Structure**	**Score**	**Carcinogen in training set**
FCFP_6	-548632217	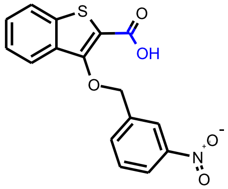 [*]C(=[*])O	-0.383	9 out of 52
FCFP_6	-1549222613	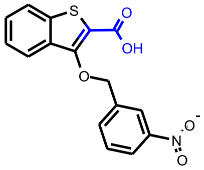 [*]:[c](:[*])C(=O)O	-0.351	1 out of 7
FCFP_6	907036844	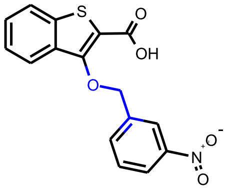 [*]OC[c](:[*]):[*]	-0.313	3 out of 17

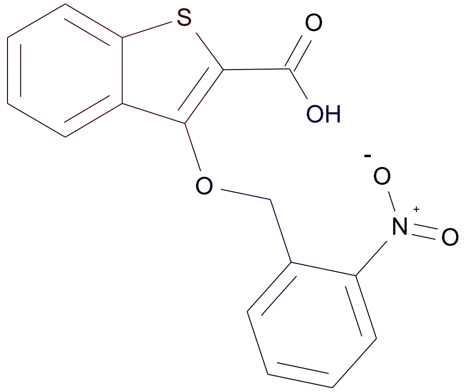

$C_{16}H_{11}NO_5S$
Molecular Weight: 329.32724
ALogP: 4.074
Rotatable Bonds: 5
Acceptors: 5
Donors: 1

Model Prediction

Prediction: Carcinogen
Probability: 0.278
Enrichment: 0.944
Bayesian Score: -1.081
Mahalanobis Distance: 16.120
Mahalanobis Distance p-value: 5.06e-010

Prediction: Positive if the Bayesian score is above the estimated best cutoff value from minimizing the false positive and false negative rate.
Probability: The esimated probability that the sample is in the positive category. This assumes that the Bayesian score follows a normal distribution and is different from the prediction using a cutoff.
Enrichment: An estimate of enrichment, that is, the increased likelihood (versus random) of this sample being in the category.
Bayesian Score: The standard Laplacian-modified Bayesian score.
Mahalanobis Distance: The Mahalanobis distance (MD) is the distance to the center of the training data. The larger the MD, the less trustworthy the prediction.
Mahalanobis Distance p-value: The p-value gives the fraction of training data with an MD greater than or equal to the one for the given sample, assuming normally distributed data. The smaller the p-value, the less trustworthy the prediciton. For highly non-normal X properties (e.g., fingerprints), the MD p-value is wildly inaccurate.

Structural Similar Compounds

Name	Omeprazole	Lansoprazole	Suprofen
Structure	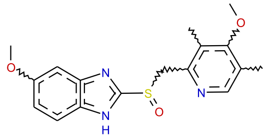	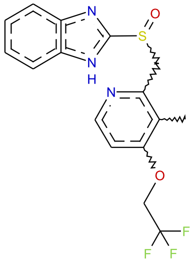	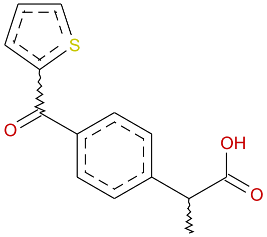
Actual Endpoint	Non-Carcinogen	Carcinogen	Carcinogen
Predicted Endpoint	Non-Carcinogen	Carcinogen	Carcinogen
Distance	0.636	0.656	0.669
Reference	US FDA (Centre for Drug Eval.& Res./Off. Testing & Res.) Sept. 1997	US FDA (Centre for Drug Eval.& Res./Off. Testing & Res.) Sept. 1997	US FDA (Centre for Drug Eval.& Res./Off. Testing & Res.) Sept. 1997

Model Applicability

Unknown features are fingerprint features in the query molecule, but not found in the training set.

1. All properties and OPS components are within expected ranges.
2. Unknown FCFP_2 feature: 5: [*][O-]
3. Unknown FCFP_2 feature: -828984032: [*][N+](=[*])[c](:[cH]:[*]):[cH]:[*]
4. Unknown FCFP_2 feature: -1338588315: [*]:[c](:[*])[N+](=O)[O-]
5. Unknown FCFP_2 feature: 1872392852: [*][N+](=O)[*]
6. Unknown FCFP_2 feature: 260476081: [*][N+](=[*])[O-]

Feature Contribution

Top features for positive contribution

Fingerprint	Bit/Smiles	Feature Structure	Score	Carcinogen in training set
FCFP_6	-387072142	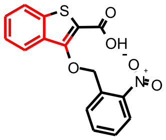 [*][c]1:[*]:[*]:[c]2:[cH]:[cH]:[cH]:[cH]:[c]:1:2	0.477	4 out of 8

FCFP_6	-1320007763	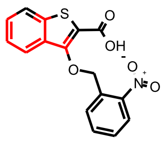 [*][c]1:[*]:[*]:[c]2:[*]:[cH]:[cH]:[cH]:[c]:1:2	0.348	6 out of 15
FCFP_6	-1539132615	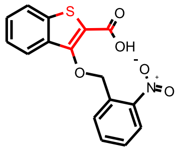 [*]C(=[*])[c]1:s:[*]:[*]:[c]:1[*]	0.328	19 out of 51

Top Features for negative contribution

**Fingerprint**	**Bit/Smiles**	**Feature Structure**	**Score**	**Carcinogen in training set**
FCFP_6	-548632217	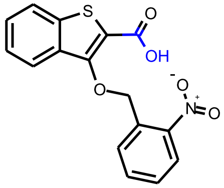 [*]C(=[*])O	-0.383	9 out of 52
FCFP_6	-1549222613	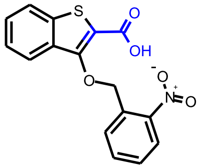 [*]:[c](:[*])C(=O)O	-0.351	1 out of 7
FCFP_6	907036844	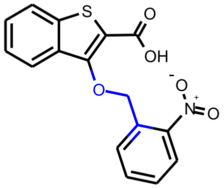 [*]OC[c](:[*]):[*]	-0.313	3 out of 17

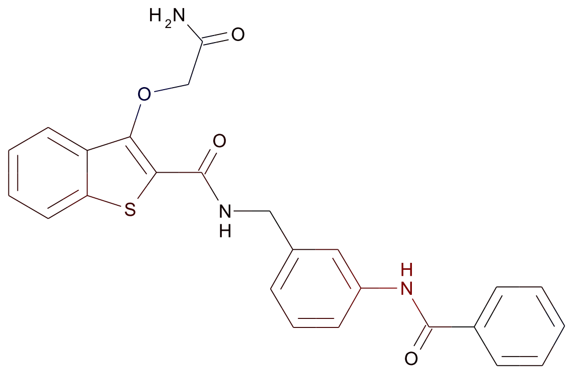

$C_{25}H_{21}N_3O_4S$
Molecular Weight: 459.51694
ALogP: 3.396
Rotatable Bonds: 8
Acceptors: 4
Donors: 3

Model Prediction

Prediction: Carcinogen
Probability: 0.362
Enrichment: 1.230
Bayesian Score: 1.881
Mahalanobis Distance: 17.436
Mahalanobis Distance p-value: 6.98e-013

Prediction: Positive if the Bayesian score is above the estimated best cutoff value from minimizing the false positive and false negative rate.
Probability: The esimated probability that the sample is in the positive category. This assumes that the Bayesian score follows a normal distribution and is different from the prediction using a cutoff.
Enrichment: An estimate of enrichment, that is, the increased likelihood (versus random) of this sample being in the category.
Bayesian Score: The standard Laplacian-modified Bayesian score.
Mahalanobis Distance: The Mahalanobis distance (MD) is the distance to the center of the training data. The larger the MD, the less trustworthy the prediction.
Mahalanobis Distance p-value: The p-value gives the fraction of training data with an MD greater than or equal to the one for the given sample, assuming normally distributed data. The smaller the p-value, the less trustworthy the prediciton. For highly non-normal X properties (e.g., fingerprints), the MD p-value is wildly inaccurate.

Structural Similar Compounds

Name	Glyburide	Glimepride	Fluvastatin
Structure	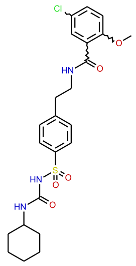	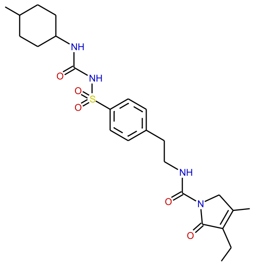	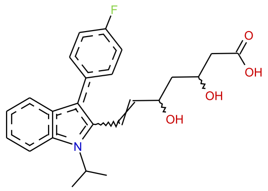
Actual Endpoint	Non-Carcinogen	Carcinogen	Non-Carcinogen
Predicted Endpoint	Non-Carcinogen	Carcinogen	Non-Carcinogen
Distance	0.593	0.594	0.620
Reference	US FDA (Centre for Drug Eval.& Res./Off. Testing & Res.) Sept. 1997	US FDA (Centre for Drug Eval.& Res./Off. Testing & Res.) Sept. 1997	US FDA (Centre for Drug Eval.& Res./Off. Testing & Res.) Sept. 1997

Model Applicability

Unknown features are fingerprint features in the query molecule, but not found in the training set.

1. All properties and OPS components are within expected ranges.

Feature Contribution

Top features for positive contribution

Fingerprint	Bit/Smiles	Feature Structure	Score	Carcinogen in training set
FCFP_6	-581879738	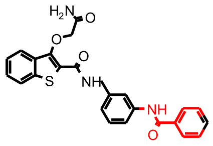 [*]NC(=O)[c]1:[cH]:[cH]:[*]:[cH]:[cH]:1	0.770	4 out of 5

FCFP_6	-1838187238	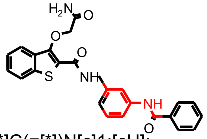 [*]C(=[*])N[c]1:[cH]:[cH]:[*]:[c]([*]):[cH]:1	0.565	4 out of 7
FCFP_6	-387072142	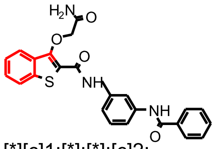 [*][c]1:[*]:[*]:[c]2:[cH]:[cH]:[cH]:[cH]:[c]:1:2	0.477	4 out of 8
**Top Features for negative contribution**				
**Fingerprint**	**Bit/Smiles**	**Feature Structure**	**Score**	**Carcinogen in training set**
FCFP_6	-1977359400	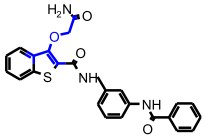 [*]CCO[c](:[cH]:[*]):[cH]:[*]	-0.300	5 out of 27
FCFP_6	907096426	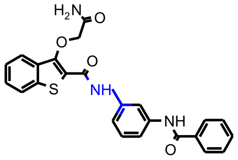 [*]NC[c](:[*]):[*]	-0.254	1 out of 6
FCFP_6	-1925475824	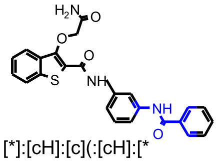 [*]:[cH]:[c](:[cH]:[*])C(=O)N[c](:[*]):[*]	-0.233	0 out of 1

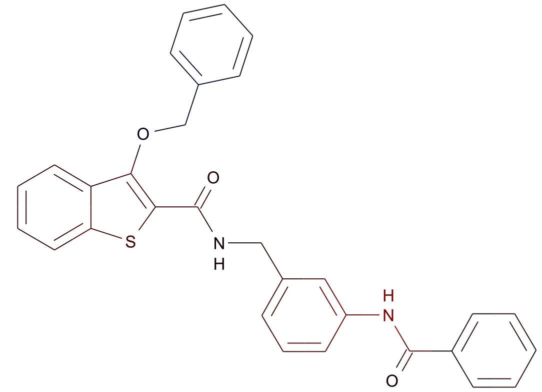

$C_{30}H_{24}N_2O_3S$
Molecular Weight: 492.58816
ALogP: 6.126
Rotatable Bonds: 8
Acceptors: 3
Donors: 2

Model Prediction

Prediction: Carcinogen
Probability: 0.398
Enrichment: 1.353
Bayesian Score: 2.957
Mahalanobis Distance: 17.954
Mahalanobis Distance p-value: 4.71e-014

Prediction: Positive if the Bayesian score is above the estimated best cutoff value from minimizing the false positive and false negative rate.
Probability: The esimated probability that the sample is in the positive category. This assumes that the Bayesian score follows a normal distribution and is different from the prediction using a cutoff.
Enrichment: An estimate of enrichment, that is, the increased likelihood (versus random) of this sample being in the category.
Bayesian Score: The standard Laplacian-modified Bayesian score.
Mahalanobis Distance: The Mahalanobis distance (MD) is the distance to the center of the training data. The larger the MD, the less trustworthy the prediction.
Mahalanobis Distance p-value: The p-value gives the fraction of training data with an MD greater than or equal to the one for the given sample, assuming normally distributed data. The smaller the p-value, the less trustworthy the prediciton. For highly non-normal X properties (e.g., fingerprints), the MD p-value is wildly inaccurate.

Structural Similar Compounds

Name	Terfenadine	Astemizole	Pimozide
Structure	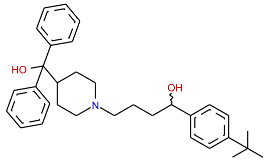	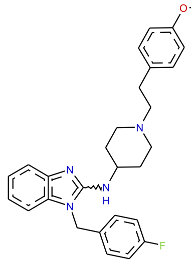	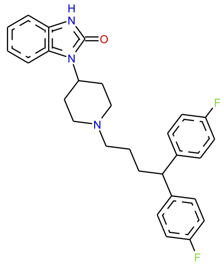
Actual Endpoint	Non-Carcinogen	Non-Carcinogen	Non-Carcinogen
Predicted Endpoint	Non-Carcinogen	Non-Carcinogen	Non-Carcinogen
Distance	0.627	0.665	0.692
Reference	US FDA (Centre for Drug Eval.& Res./Off. Testing & Res.) Sept. 1997	US FDA (Centre for Drug Eval.& Res./Off. Testing & Res.) Sept. 1997	US FDA (Centre for Drug Eval.& Res./Off. Testing & Res.) Sept. 1997

Model Applicability

Unknown features are fingerprint features in the query molecule, but not found in the training set.

1. All properties and OPS components are within expected ranges.

Feature Contribution

Top features for positive contribution				
**Fingerprint**	**Bit/Smiles**	**Feature Structure**	**Score**	**Carcinogen in training set**
FCFP_6	-581879738	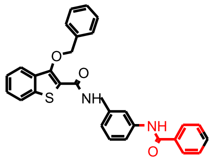 [*]NC(=O)[c]1:[cH]:[cH]:[*]:[cH]:[cH]:1	0.770	4 out of 5

FCFP_6	-1838187238	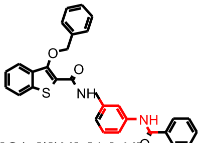 [*]C(=[*])N[c]1:[cH]:[cH]:[*]:[c]([*]):[cH]:1	0.565	4 out of 7
FCFP_6	-387072142	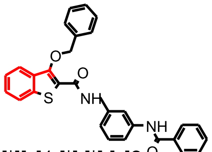 [*][c]1:[*]:[*]:[c]2:[cH]:[cH]:[cH]:[cH]:[c]:1:2	0.477	4 out of 8

Top Features for negative contribution

**Fingerprint**	**Bit/Smiles**	**Feature Structure**	**Score**	**Carcinogen in training set**
FCFP_6	907036844	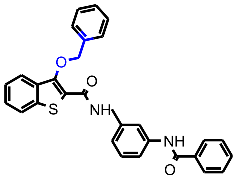 [*]OC[c](:[*]):[*]	-0.313	3 out of 17
FCFP_6	907096426	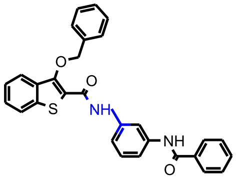 [*]NC[c](:[*]):[*]	-0.254	1 out of 6
FCFP_6	-1925475824	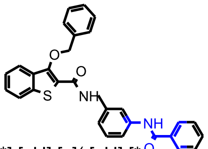 [*]:[cH]:[c](:[cH]:[*])C(=O)N[c](:[*]):[*]	-0.233	0 out of 1

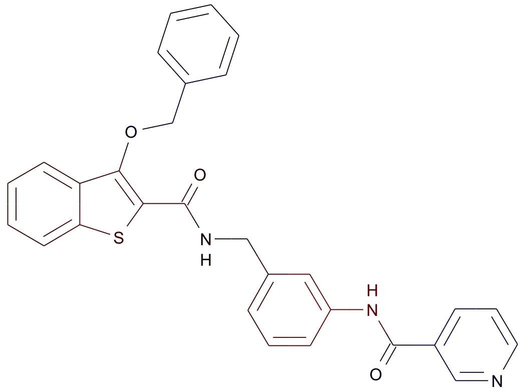

$C_{29}H_{23}N_3O_3S$
Molecular Weight: 493.57622
ALogP: 4.976
Rotatable Bonds: 8
Acceptors: 4
Donors: 2

Model Prediction

Prediction: Carcinogen
Probability: 0.336
Enrichment: 1.141
Bayesian Score: 1.045
Mahalanobis Distance: 17.597
Mahalanobis Distance p-value: 3.03e-013

Prediction: Positive if the Bayesian score is above the estimated best cutoff value from minimizing the false positive and false negative rate.
Probability: The esimated probability that the sample is in the positive category. This assumes that the Bayesian score follows a normal distribution and is different from the prediction using a cutoff.
Enrichment: An estimate of enrichment, that is, the increased likelihood (versus random) of this sample being in the category.
Bayesian Score: The standard Laplacian-modified Bayesian score.
Mahalanobis Distance: The Mahalanobis distance (MD) is the distance to the center of the training data. The larger the MD, the less trustworthy the prediction.
Mahalanobis Distance p-value: The p-value gives the fraction of training data with an MD greater than or equal to the one for the given sample, assuming normally distributed data. The smaller the p-value, the less trustworthy the prediciton. For highly non-normal X properties (e.g., fingerprints), the MD p-value is wildly inaccurate.

Structural Similar Compounds

Name	Glyburide	Fluvastatin	Flecainide
Structure	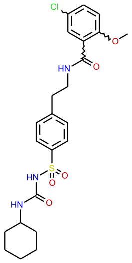	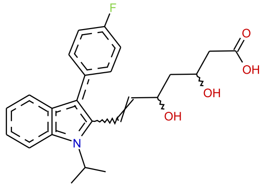	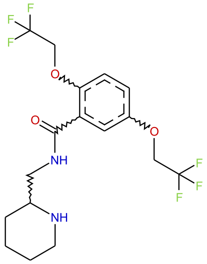
Actual Endpoint	Non-Carcinogen	Non-Carcinogen	Non-Carcinogen
Predicted Endpoint	Non-Carcinogen	Non-Carcinogen	Non-Carcinogen
Distance	0.639	0.655	0.673
Reference	US FDA (Centre for Drug Eval.& Res./Off. Testing & Res.) Sept. 1997	US FDA (Centre for Drug Eval.& Res./Off. Testing & Res.) Sept. 1997	US FDA (Centre for Drug Eval.& Res./Off. Testing & Res.) Sept. 1997

Model Applicability

Unknown features are fingerprint features in the query molecule, but not found in the training set.

1. All properties and OPS components are within expected ranges.

Feature Contribution

Top features for positive contribution

Fingerprint	Bit/Smiles	Feature Structure	Score	Carcinogen in training set
FCFP_6	-1838187238	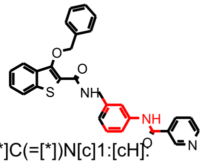 [*]C(=[*])N[c]1:[cH]:[cH]:[*]:[c]([*]):[cH]:1	0.565	4 out of 7

FCFP_6	-387072142	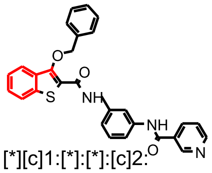<br>[*][c]1:[*]:[*]:[c]2:<br>[cH]:[cH]:[cH]:[cH]:<br>[c]:1:2	0.477	4 out of 8
FCFP_6	1294255210	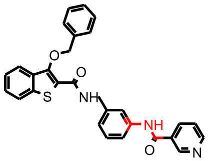<br>[*]C(=[*])N[c](:[*]):<br>[*]	0.441	12 out of 28
**Top Features for negative contribution**				
**Fingerprint**	**Bit/Smiles**	**Feature Structure**	**Score**	**Carcinogen in training set**
FCFP_6	1153798395	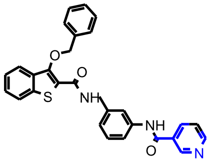<br>[*]C(=[*])[c]1:[cH]:[<br>*]:[cH]:n:[cH]:1	-0.582	0 out of 3
FCFP_6	1888947587	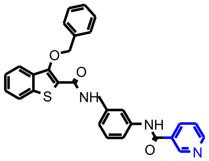<br>[*][c]1:[cH]:[cH]:[cH<br>]:n:[cH]:1	-0.423	0 out of 2
FCFP_6	907036844	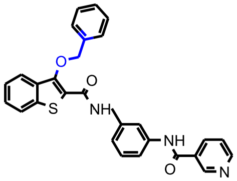<br>[*]OC[c](:[*]):[*]	-0.313	3 out of 17

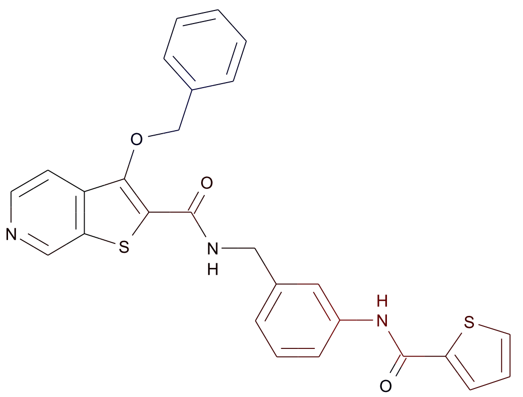

$C_{27}H_{21}N_3O_3S_2$
Molecular Weight: 499.60394
ALogP: 4.929
Rotatable Bonds: 8
Acceptors: 4
Donors: 2

Model Prediction

Prediction: Carcinogen
Probability: 0.365
Enrichment: 1.242
Bayesian Score: 1.989
Mahalanobis Distance: 19.066
Mahalanobis Distance p-value: 1.24e-016

Prediction: Positive if the Bayesian score is above the estimated best cutoff value from minimizing the false positive and false negative rate.
Probability: The esimated probability that the sample is in the positive category. This assumes that the Bayesian score follows a normal distribution and is different from the prediction using a cutoff.
Enrichment: An estimate of enrichment, that is, the increased likelihood (versus random) of this sample being in the category.
Bayesian Score: The standard Laplacian-modified Bayesian score.
Mahalanobis Distance: The Mahalanobis distance (MD) is the distance to the center of the training data. The larger the MD, the less trustworthy the prediction.
Mahalanobis Distance p-value: The p-value gives the fraction of training data with an MD greater than or equal to the one for the given sample, assuming normally distributed data. The smaller the p-value, the less trustworthy the prediciton. For highly non-normal X properties (e.g., fingerprints), the MD p-value is wildly inaccurate.

Structural Similar Compounds

Name	Glyburide	Glimepride	Fluvastatin
Structure	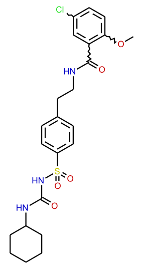	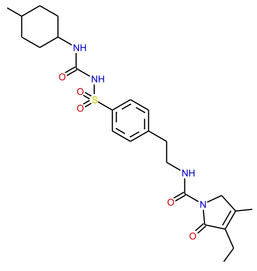	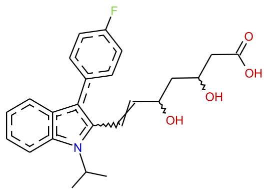
Actual Endpoint	Non-Carcinogen	Carcinogen	Non-Carcinogen
Predicted Endpoint	Non-Carcinogen	Carcinogen	Non-Carcinogen
Distance	0.637	0.672	0.704
Reference	US FDA (Centre for Drug Eval.& Res./Off. Testing & Res.) Sept. 1997	US FDA (Centre for Drug Eval.& Res./Off. Testing & Res.) Sept. 1997	US FDA (Centre for Drug Eval.& Res./Off. Testing & Res.) Sept. 1997

Model Applicability

Unknown features are fingerprint features in the query molecule, but not found in the training set.

1. OPS PC23 out of range. Value: 3.6488. Training min, max, SD, explained variance: -3.8399, 3.416, 1.099, 0.0127.

Feature Contribution

Top features for positive contribution

Fingerprint	Bit/Smiles	Feature Structure	Score	Carcinogen in training set
FCFP_6	-1838187238	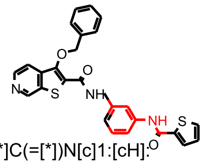 [*]C(=[*])N[c]1:[cH]:[cH]:[*]:[c]([*]):[cH]:1	0.565	4 out of 7

FCFP_6	1294255210	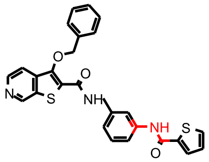 [*]C(=[*])N[c](:[*]):[*]	0.441	12 out of 28
FCFP_6	137138064	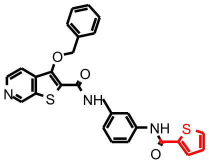 [*]C(=[*])[c]1:[cH]:[cH]:[cH]:s:1	0.439	3 out of 6
**Top Features for negative contribution**				
**Fingerprint**	**Bit/Smiles**	**Feature Structure**	**Score**	**Carcinogen in training set**
FCFP_6	907036844	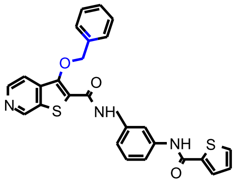 [*]OC[c](:[*]):[*]	-0.313	3 out of 17
FCFP_6	547884906	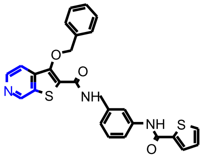 [*][c]1:[*]:[cH]:[cH]:n:[cH]:1	-0.254	1 out of 6
FCFP_6	907096426	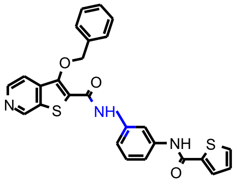 [*]NC[c](:[*]):[*]	-0.254	1 out of 6

Co-crystallized ligand

TOPKAT_Mouse_Male_FDA_None_vs_Carcinogen

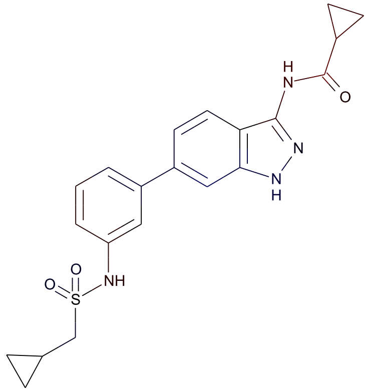

$C_{21}H_{22}N_4O_3S$
Molecular Weight: 410.48938
ALogP: 3.14
Rotatable Bonds: 7
Acceptors: 4
Donors: 3

Structural Similar Compounds

Name	Fluvastatin	Torsemide	Bicalutamide
Structure	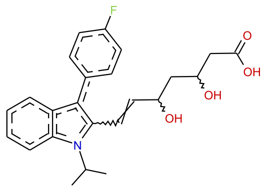	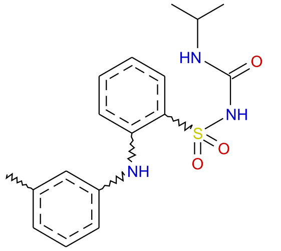	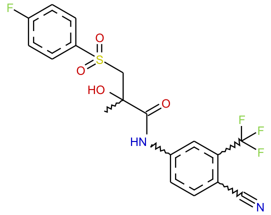
Actual Endpoint	Non-Carcinogen	Non-Carcinogen	Carcinogen
Predicted Endpoint	Non-Carcinogen	Non-Carcinogen	Carcinogen
Distance	0.623	0.624	0.624
Reference	US FDA (Centre for Drug Eval.& Res./Off. Testing & Res.) Sept. 1997	US FDA (Centre for Drug Eval.& Res./Off. Testing & Res.) Sept. 1997	US FDA (Centre for Drug Eval.& Res./Off. Testing & Res.) Sept. 1997

Model Prediction

Prediction: Carcinogen
Probability: 0.303
Enrichment: 1.029
Bayesian Score: -0.115
Mahalanobis Distance: 14.190
Mahalanobis Distance p-value: 3e-006

Prediction: Positive if the Bayesian score is above the estimated best cutoff value from minimizing the false positive and false negative rate.
Probability: The esimated probability that the sample is in the positive category. This assumes that the Bayesian score follows a normal distribution and is different from the prediction using a cutoff.
Enrichment: An estimate of enrichment, that is, the increased likelihood (versus random) of this sample being in the category.
Bayesian Score: The standard Laplacian-modified Bayesian score.
Mahalanobis Distance: The Mahalanobis distance (MD) is the distance to the center of the training data. The larger the MD, the less trustworthy the prediction.
Mahalanobis Distance p-value: The p-value gives the fraction of training data with an MD greater than or equal to the one for the given sample, assuming normally distributed data. The smaller the p-value, the less trustworthy the prediciton. For highly non-normal X properties (e.g., fingerprints), the MD p-value is wildly inaccurate.

Model Applicability

Unknown features are fingerprint features in the query molecule, but not found in the training set.

1. All properties and OPS components are within expected ranges.

Feature Contribution

Top features for positive contribution

Fingerprint	Bit/Smiles	Feature Structure	Score	Carcinogen in training set
FCFP_6	-55265897	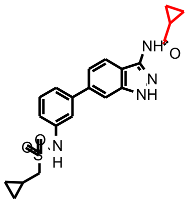 [*]C(=[*])C1CC1	0.594	17 out of 34

FCFP_6	-1838187238	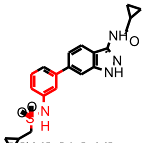 [*]C(=[*])N[c]1:[cH]:[cH]:[*]:[c]([*]):[cH]:1	0.565	4 out of 7
FCFP_6	566058135	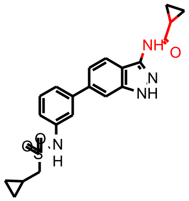 [*]CC(=O)N	0.447	17 out of 40

Top Features for negative contribution

Fingerprint	Bit/Smiles	Feature Structure	Score	Carcinogen in training set
FCFP_6	1764617907	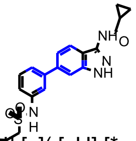 [*]:[cH]:[c](:[cH]:[*])[c]1:[cH]:[cH]:[*]:[c](:[*]):[cH]:1	-0.719	0 out of 4
FCFP_6	1018942292	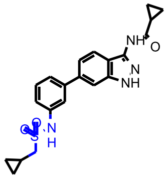 [*]CS(=O)(=O)N[*]	-0.423	0 out of 2
FCFP_6	262592487	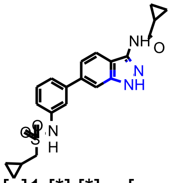 [*]:[c]1:[*]:[*]:n:[nH]:1	-0.423	0 out of 2

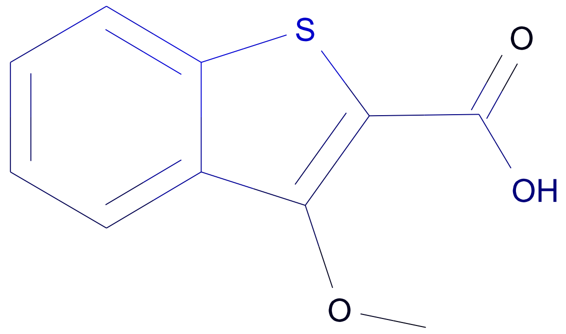

$C_{10}H_8O_3S$
Molecular Weight: 208.23372
ALogP: 2.596
Rotatable Bonds: 2
Acceptors: 3
Donors: 1

Model Prediction

Prediction: Single-Carcinogen
Probability: 0.146
Enrichment: 0.486
Bayesian Score: -10.221
Mahalanobis Distance: 12.909
Mahalanobis Distance p-value: 0.000116

Prediction: Positive if the Bayesian score is above the estimated best cutoff value from minimizing the false positive and false negative rate.
Probability: The esimated probability that the sample is in the positive category. This assumes that the Bayesian score follows a normal distribution and is different from the prediction using a cutoff.
Enrichment: An estimate of enrichment, that is, the increased likelihood (versus random) of this sample being in the category.
Bayesian Score: The standard Laplacian-modified Bayesian score.
Mahalanobis Distance: The Mahalanobis distance (MD) is the distance to the center of the training data. The larger the MD, the less trustworthy the prediction.
Mahalanobis Distance p-value: The p-value gives the fraction of training data with an MD greater than or equal to the one for the given sample, assuming normally distributed data. The smaller the p-value, the less trustworthy the prediciton. For highly non-normal X properties (e.g., fingerprints), the MD p-value is wildly inaccurate.

Structural Similar Compounds

Name	Ethionamide	Suprofen	Nitroacetophenetide
Structure	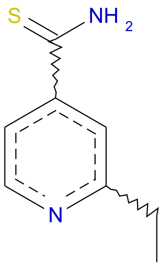	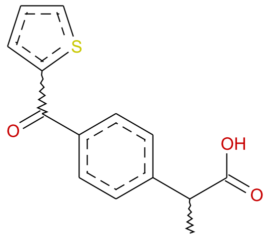	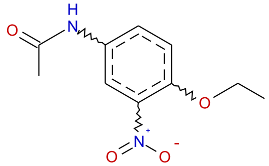
Actual Endpoint	Single-Carcinogen	Single-Carcinogen	Single-Carcinogen
Predicted Endpoint	Single-Carcinogen	Single-Carcinogen	Single-Carcinogen
Distance	0.588	0.616	0.659
Reference	US FDA (Centre for Drug Eval.& Res./Off. Testing & Res.) Sept. 1997	US FDA (Centre for Drug Eval.& Res./Off. Testing & Res.) Sept. 1997	US FDA (Centre for Drug Eval.& Res./Off. Testing & Res.) Sept. 1997

Model Applicability

Unknown features are fingerprint features in the query molecule, but not found in the training set.

1. All properties and OPS components are within expected ranges.

Feature Contribution

Top features for positive contribution

Fingerprint	Bit/Smiles	Feature Structure	Score	Multiple-Carcinogen in training set
FCFP_12	-1977641857	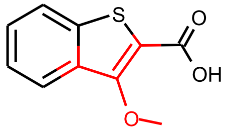 [*][c]1:[*]:[*]:[c](:[*]):[c]:1OC	0.105	2 out of 5

Top Features for negative contribution

Fingerprint	Bit/Smiles	Feature Structure	Score	Multiple-Carcinogen in training set

FCFP_12	-548632217	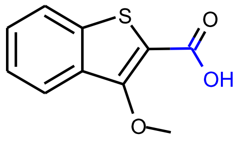 [*]C(=[*])O	-1.402	0 out of 9
FCFP_12	178336375	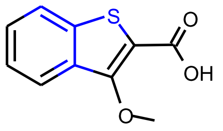 [*]:[cH]:[c]1:s:[*]:[*]:[c]:1:[*]	-0.994	0 out of 5
FCFP_12	7	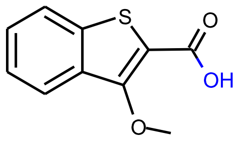 [*]O	-0.710	2 out of 15

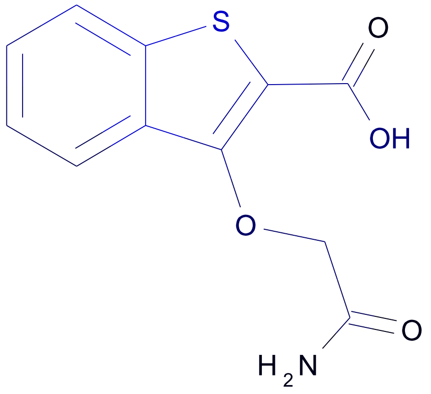

$C_{11}H_9NO_4S$
Molecular Weight: 251.25846
ALogP: 1.45
Rotatable Bonds: 4
Acceptors: 4
Donors: 2

Model Prediction

Prediction: Single-Carcinogen
Probability: 0.157
Enrichment: 0.520
Bayesian Score: -12.776
Mahalanobis Distance: 14.265
Mahalanobis Distance p-value: 9.67e-006

Prediction: Positive if the Bayesian score is above the estimated best cutoff value from minimizing the false positive and false negative rate.
Probability: The esimated probability that the sample is in the positive category. This assumes that the Bayesian score follows a normal distribution and is different from the prediction using a cutoff.
Enrichment: An estimate of enrichment, that is, the increased likelihood (versus random) of this sample being in the category.
Bayesian Score: The standard Laplacian-modified Bayesian score.
Mahalanobis Distance: The Mahalanobis distance (MD) is the distance to the center of the training data. The larger the MD, the less trustworthy the prediction.
Mahalanobis Distance p-value: The p-value gives the fraction of training data with an MD greater than or equal to the one for the given sample, assuming normally distributed data. The smaller the p-value, the less trustworthy the prediciton. For highly non-normal X properties (e.g., fingerprints), the MD p-value is wildly inaccurate.

Structural Similar Compounds

Name	Nithiazide	Sulfamethazine	Furothiazole
Structure	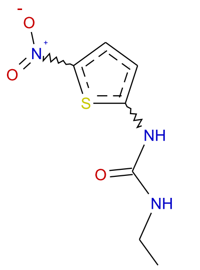	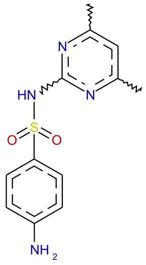	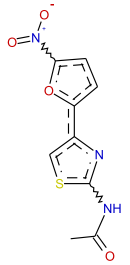
Actual Endpoint	Single-Carcinogen	Single-Carcinogen	Single-Carcinogen
Predicted Endpoint	Single-Carcinogen	Single-Carcinogen	Single-Carcinogen
Distance	0.587	0.641	0.644
Reference	US FDA (Centre for Drug Eval.& Res./Off. Testing & Res.) Sept. 1997	US FDA (Centre for Drug Eval.& Res./Off. Testing & Res.) Sept. 1997	US FDA (Centre for Drug Eval.& Res./Off. Testing & Res.) Sept. 1997

Model Applicability

Unknown features are fingerprint features in the query molecule, but not found in the training set.

1. All properties and OPS components are within expected ranges.

Feature Contribution

Top features for positive contribution

Fingerprint	Bit/Smiles	Feature Structure	Score	Multiple-Carcinogen in training set
FCFP_12	1070061035	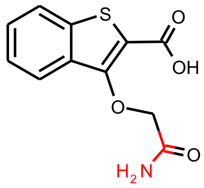 [*]C(=[*])N	0.135	13 out of 33

Top Features for negative contribution

Fingerprint	Bit/Smiles	Feature Structure	Score	Multiple-Carcinogen in training set

FCFP_12	-548632217	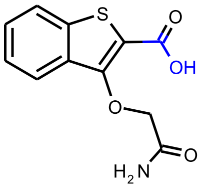 [*]C(=[*])O	-1.402	0 out of 9
FCFP_12	-1977359400	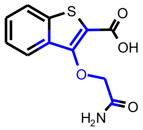 [*]C(=[*])CO[c]1:[c]([*]):[*]:[*]:[c]:1:[*]	-0.994	0 out of 5
FCFP_12	178336375	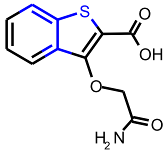 [*]:[cH]:[c]1:s:[*]:[*]:[c]:1:[*]	-0.994	0 out of 5

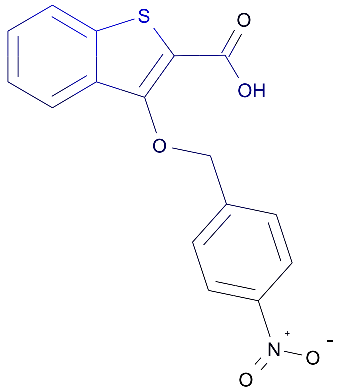

$C_{16}H_{11}NO_5S$

Molecular Weight: 329.32724

ALogP: 4.074

Rotatable Bonds: 5

Acceptors: 5

Donors: 1

Model Prediction

Prediction: Single-Carcinogen

Probability: 0.148

Enrichment: 0.490

Bayesian Score: -10.828

Mahalanobis Distance: 15.545

Mahalanobis Distance p-value: 9.35e-007

Prediction: Positive if the Bayesian score is above the estimated best cutoff value from minimizing the false positive and false negative rate.
Probability: The esimated probability that the sample is in the positive category. This assumes that the Bayesian score follows a normal distribution and is different from the prediction using a cutoff.
Enrichment: An estimate of enrichment, that is, the increased likelihood (versus random) of this sample being in the category.
Bayesian Score: The standard Laplacian-modified Bayesian score.
Mahalanobis Distance: The Mahalanobis distance (MD) is the distance to the center of the training data. The larger the MD, the less trustworthy the prediction.
Mahalanobis Distance p-value: The p-value gives the fraction of training data with an MD greater than or equal to the one for the given sample, assuming normally distributed data. The smaller the p-value, the less trustworthy the prediciton. For highly non-normal X properties (e.g., fingerprints), the MD p-value is wildly inaccurate.

Structural Similar Compounds

Name	Lansoprazole	Suprofen	Oxaprocin
Structure	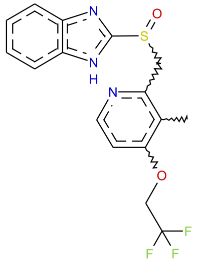	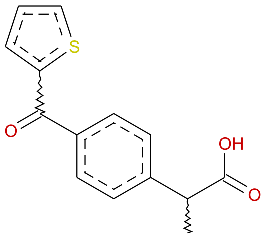	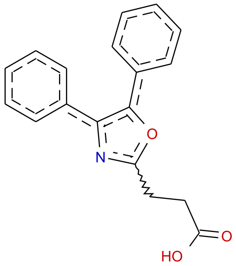
Actual Endpoint	Single-Carcinogen	Single-Carcinogen	Single-Carcinogen
Predicted Endpoint	Single-Carcinogen	Single-Carcinogen	Single-Carcinogen
Distance	0.676	0.713	0.756
Reference	US FDA (Centre for Drug Eval.& Res./Off. Testing & Res.) Sept. 1997	US FDA (Centre for Drug Eval.& Res./Off. Testing & Res.) Sept. 1997	US FDA (Centre for Drug Eval.& Res./Off. Testing & Res.) Sept. 1997

Model Applicability

Unknown features are fingerprint features in the query molecule, but not found in the training set.

1. All properties and OPS components are within expected ranges.
2. Unknown FCFP_2 feature: 5: [*][O-]
3. Unknown FCFP_2 feature: -828984032: [*][N+](=[*])[c](:[cH]:[*]):[cH]:[*]
4. Unknown FCFP_2 feature: -1338588315: [*]:[c](:[*])[N+](=O)[O-]
5. Unknown FCFP_2 feature: 1872392852: [*][N+](=O)[*]
6. Unknown FCFP_2 feature: 260476081: [*][N+](=[*])[O-]

Feature Contribution

Top Features for negative contribution

Fingerprint	Bit/Smiles	Feature Structure	Score	Multiple-Carcinogen in training set

FCFP_12	-548632217	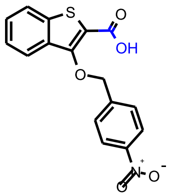 [*]C(=[*])O	-1.402	0 out of 9
FCFP_12	178336375	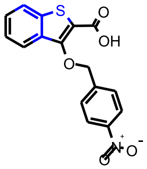 [*]:[cH]:[c]1:s:[*]:[*]:[c]:1:[*]	-0.994	0 out of 5
FCFP_12	7	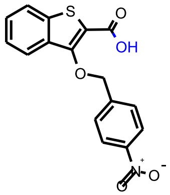 [*]O	-0.710	2 out of 15

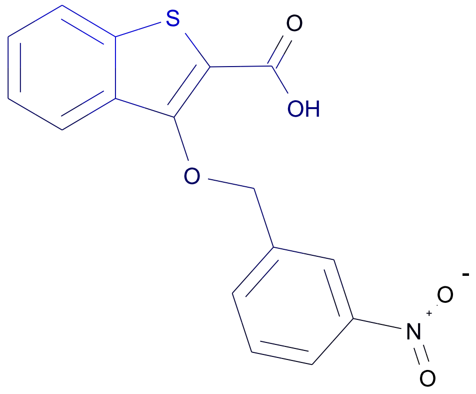

$C_{16}H_{11}NO_5S$

Molecular Weight: 329.32724

ALogP: 4.074

Rotatable Bonds: 5

Acceptors: 5

Donors: 1

Model Prediction

Prediction: Single-Carcinogen

Probability: 0.148

Enrichment: 0.493

Bayesian Score: -11.121

Mahalanobis Distance: 15.545

Mahalanobis Distance p-value: 9.35e-007

Prediction: Positive if the Bayesian score is above the estimated best cutoff value from minimizing the false positive and false negative rate.
Probability: The esimated probability that the sample is in the positive category. This assumes that the Bayesian score follows a normal distribution and is different from the prediction using a cutoff.
Enrichment: An estimate of enrichment, that is, the increased likelihood (versus random) of this sample being in the category.
Bayesian Score: The standard Laplacian-modified Bayesian score.
Mahalanobis Distance: The Mahalanobis distance (MD) is the distance to the center of the training data. The larger the MD, the less trustworthy the prediction.
Mahalanobis Distance p-value: The p-value gives the fraction of training data with an MD greater than or equal to the one for the given sample, assuming normally distributed data. The smaller the p-value, the less trustworthy the prediciton. For highly non-normal X properties (e.g., fingerprints), the MD p-value is wildly inaccurate.

Structural Similar Compounds

Name	Lansoprazole	Suprofen	Oxaprocin
Structure	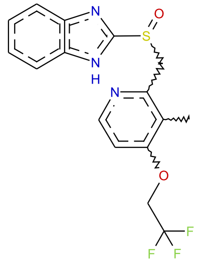	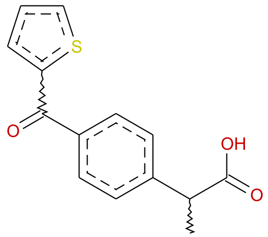	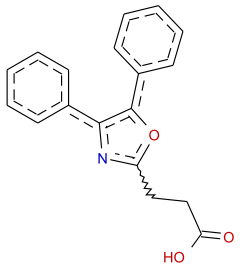
Actual Endpoint	Single-Carcinogen	Single-Carcinogen	Single-Carcinogen
Predicted Endpoint	Single-Carcinogen	Single-Carcinogen	Single-Carcinogen
Distance	0.675	0.713	0.756
Reference	US FDA (Centre for Drug Eval.& Res./Off. Testing & Res.) Sept. 1997	US FDA (Centre for Drug Eval.& Res./Off. Testing & Res.) Sept. 1997	US FDA (Centre for Drug Eval.& Res./Off. Testing & Res.) Sept. 1997

Model Applicability

Unknown features are fingerprint features in the query molecule, but not found in the training set.

1. All properties and OPS components are within expected ranges.
2. Unknown FCFP_2 feature: 5: [*][O-]
3. Unknown FCFP_2 feature: -828984032: [*][N+](=[*])[c](:[cH]:[*]):[cH]:[*]
4. Unknown FCFP_2 feature: -1338588315: [*]:[c](:[*])[N+](=O)[O-]
5. Unknown FCFP_2 feature: 1872392852: [*][N+](=O)[*]
6. Unknown FCFP_2 feature: 260476081: [*][N+](=[*])[O-]

Feature Contribution

Top Features for negative contribution

Fingerprint	Bit/Smiles	Feature Structure	Score	Multiple-Carcinogen in training set

FCFP_12	-548632217	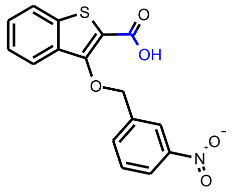 [*]C(=[*])O	-1.402	0 out of 9
FCFP_12	178336375	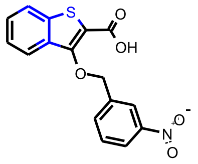 [*]:[cH]:[c]1:s:[*]:[*]:[c]:1:[*]	-0.994	0 out of 5
FCFP_12	7	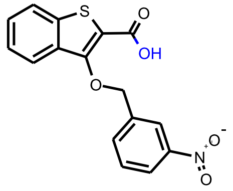 [*]O	-0.710	2 out of 15

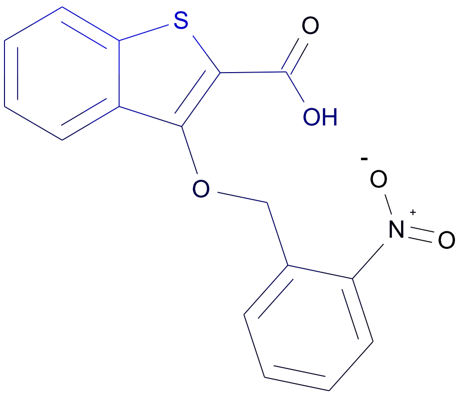

$C_{16}H_{11}NO_5S$
Molecular Weight: 329.32724
ALogP: 4.074
Rotatable Bonds: 5
Acceptors: 5
Donors: 1

Model Prediction

Prediction: Single-Carcinogen
Probability: 0.148
Enrichment: 0.491
Bayesian Score: -10.958
Mahalanobis Distance: 15.706
Mahalanobis Distance p-value: 6.99e-007

Prediction: Positive if the Bayesian score is above the estimated best cutoff value from minimizing the false positive and false negative rate.
Probability: The esimated probability that the sample is in the positive category. This assumes that the Bayesian score follows a normal distribution and is different from the prediction using a cutoff.
Enrichment: An estimate of enrichment, that is, the increased likelihood (versus random) of this sample being in the category.
Bayesian Score: The standard Laplacian-modified Bayesian score.
Mahalanobis Distance: The Mahalanobis distance (MD) is the distance to the center of the training data. The larger the MD, the less trustworthy the prediction.
Mahalanobis Distance p-value: The p-value gives the fraction of training data with an MD greater than or equal to the one for the given sample, assuming normally distributed data. The smaller the p-value, the less trustworthy the prediciton. For highly non-normal X properties (e.g., fingerprints), the MD p-value is wildly inaccurate.

Structural Similar Compounds

Name	Lansoprazole	Suprofen	Oxaprocin
Structure	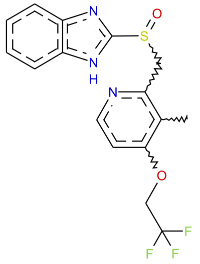	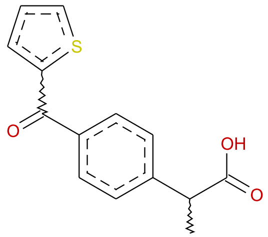	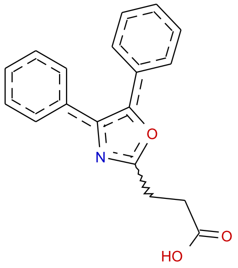
Actual Endpoint	Single-Carcinogen	Single-Carcinogen	Single-Carcinogen
Predicted Endpoint	Single-Carcinogen	Single-Carcinogen	Single-Carcinogen
Distance	0.674	0.710	0.754
Reference	US FDA (Centre for Drug Eval.& Res./Off. Testing & Res.) Sept. 1997	US FDA (Centre for Drug Eval.& Res./Off. Testing & Res.) Sept. 1997	US FDA (Centre for Drug Eval.& Res./Off. Testing & Res.) Sept. 1997

Model Applicability

Unknown features are fingerprint features in the query molecule, but not found in the training set.

1. All properties and OPS components are within expected ranges.
2. Unknown FCFP_2 feature: 5: [*][O-]
3. Unknown FCFP_2 feature: -828984032: [*][N+](=[*])[c](:[cH]:[*]):[cH]:[*]
4. Unknown FCFP_2 feature: -1338588315: [*]:[c](:[*])[N+](=O)[O-]
5. Unknown FCFP_2 feature: 1872392852: [*][N+](=O)[*]
6. Unknown FCFP_2 feature: 260476081: [*][N+](=[*])[O-]

Feature Contribution

Top Features for negative contribution

Fingerprint	Bit/Smiles	Feature Structure	Score	Multiple-Carcinogen in training set

FCFP_12	-548632217	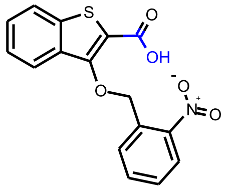 [*]C(=[*])O	-1.402	0 out of 9
FCFP_12	178336375	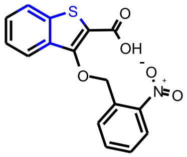 [*]:[cH]:[c]1:s:[*]:[*]:[c]:1:[*]	-0.994	0 out of 5
FCFP_12	7	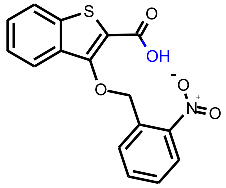 [*]O	-0.710	2 out of 15

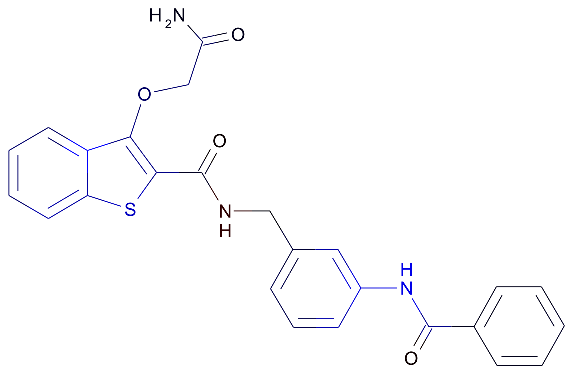

$C_{25}H_{21}N_3O_4S$

Molecular Weight: 459.51694

ALogP: 3.396

Rotatable Bonds: 8

Acceptors: 4

Donors: 3

Structural Similar Compounds

Name	Glimepride	Bicalutamide	Primidolol
Structure	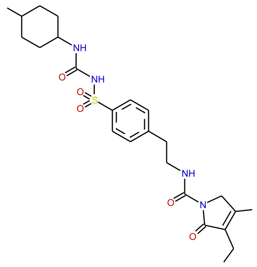	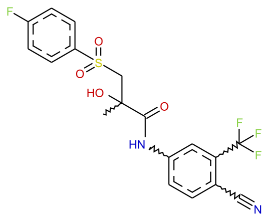	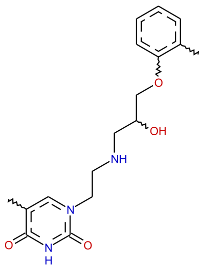
Actual Endpoint	Single-Carcinogen	Single-Carcinogen	Single-Carcinogen
Predicted Endpoint	Single-Carcinogen	Single-Carcinogen	Single-Carcinogen
Distance	0.622	0.717	0.785
Reference	US FDA (Centre for Drug Eval.& Res./Off. Testing & Res.) Sept. 1997	US FDA (Centre for Drug Eval.& Res./Off. Testing & Res.) Sept. 1997	US FDA (Centre for Drug Eval.& Res./Off. Testing & Res.) Sept. 1997

Model Prediction

Prediction: Single-Carcinogen

Probability: 0.144

Enrichment: 0.477

Bayesian Score: -14.177

Mahalanobis Distance: 19.587

Mahalanobis Distance p-value: 8.33e-010

Prediction: Positive if the Bayesian score is above the estimated best cutoff value from minimizing the false positive and false negative rate.
Probability: The esimated probability that the sample is in the positive category. This assumes that the Bayesian score follows a normal distribution and is different from the prediction using a cutoff.
Enrichment: An estimate of enrichment, that is, the increased likelihood (versus random) of this sample being in the category.
Bayesian Score: The standard Laplacian-modified Bayesian score.
Mahalanobis Distance: The Mahalanobis distance (MD) is the distance to the center of the training data. The larger the MD, the less trustworthy the prediction.
Mahalanobis Distance p-value: The p-value gives the fraction of training data with an MD greater than or equal to the one for the given sample, assuming normally distributed data. The smaller the p-value, the less trustworthy the prediciton. For highly non-normal X properties (e.g., fingerprints), the MD p-value is wildly inaccurate.

Model Applicability

Unknown features are fingerprint features in the query molecule, but not found in the training set.

1. OPS PC2 out of range. Value: 5.1119. Training min, max, SD, explained variance: -5.2888, 4.2744, 2.566, 0.1229.

Feature Contribution

Top features for positive contribution

Fingerprint	Bit/Smiles	Feature Structure	Score	Multiple-Carcinogen in training set
FCFP_12	427906732	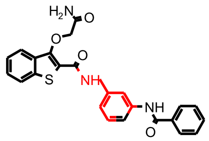 [*]NC[c]1:[cH]:[cH]:[*]:[c]([*]):[cH]:1	0.400	1 out of 1

FCFP_12	907096426	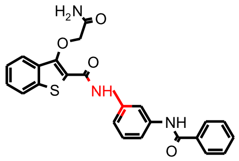 [*]NC[c](:[*]):[*]	0.400	1 out of 1
FCFP_12	-581879738	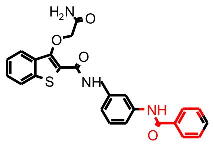 [*]NC(=O)[c]1:[cH]:[cH]:[*]:[cH]:[cH]:1	0.239	2 out of 4

Top Features for negative contribution

**Fingerprint**	**Bit/Smiles**	**Feature Structure**	**Score**	**Multiple-Carcinogen in training set**
FCFP_12	1294255210	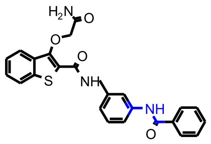 [*]C(=[*])N[c](:[*]):[*]	-1.626	0 out of 12
FCFP_12	590925877	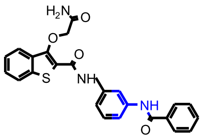 [*]N[c](:[cH]:[*]):[cH]:[*]	-0.998	1 out of 13
FCFP_12	-1977359400	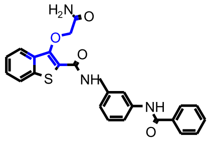 [*]C(=[*])CO[c]1:[c]([*]):[*]:[*]:[c]:1:[*]	-0.994	0 out of 5

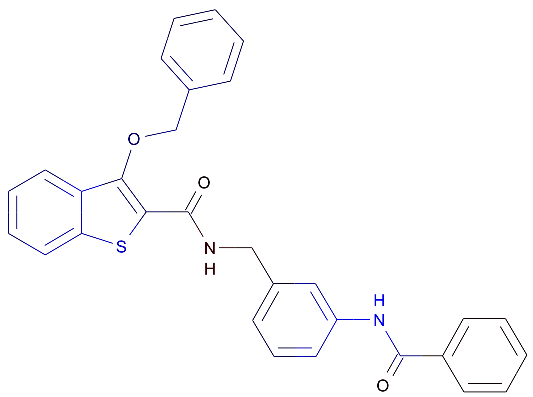

$C_{30}H_{24}N_2O_3S$
Molecular Weight: 492.58816
ALogP: 6.126
Rotatable Bonds: 8
Acceptors: 3
Donors: 2

Model Prediction

Prediction: Single-Carcinogen
Probability: 0.155
Enrichment: 0.516
Bayesian Score: -12.518
Mahalanobis Distance: 20.642
Mahalanobis Distance p-value: 1.49e-010

Prediction: Positive if the Bayesian score is above the estimated best cutoff value from minimizing the false positive and false negative rate.
Probability: The esimated probability that the sample is in the positive category. This assumes that the Bayesian score follows a normal distribution and is different from the prediction using a cutoff.
Enrichment: An estimate of enrichment, that is, the increased likelihood (versus random) of this sample being in the category.
Bayesian Score: The standard Laplacian-modified Bayesian score.
Mahalanobis Distance: The Mahalanobis distance (MD) is the distance to the center of the training data. The larger the MD, the less trustworthy the prediction.
Mahalanobis Distance p-value: The p-value gives the fraction of training data with an MD greater than or equal to the one for the given sample, assuming normally distributed data. The smaller the p-value, the less trustworthy the prediciton. For highly non-normal X properties (e.g., fingerprints), the MD p-value is wildly inaccurate.

Structural Similar Compounds

Name	Simvastatin	Glimepride	Lovastatin
Structure	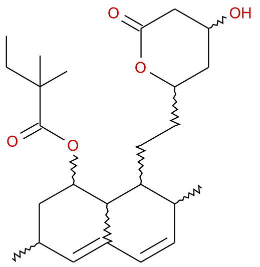	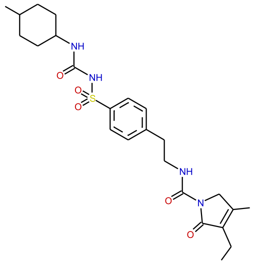	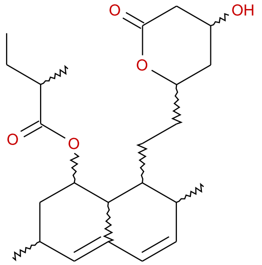
Actual Endpoint	Multiple-Carcinogen	Single-Carcinogen	Multiple-Carcinogen
Predicted Endpoint	Multiple-Carcinogen	Single-Carcinogen	Multiple-Carcinogen
Distance	0.831	0.833	0.854
Reference	US FDA (Centre for Drug Eval.& Res./Off. Testing & Res.) Sept. 1997	US FDA (Centre for Drug Eval.& Res./Off. Testing & Res.) Sept. 1997	US FDA (Centre for Drug Eval.& Res./Off. Testing & Res.) Sept. 1997

Model Applicability

Unknown features are fingerprint features in the query molecule, but not found in the training set.

1. OPS PC2 out of range. Value: 5.0183. Training min, max, SD, explained variance: -5.2888, 4.2744, 2.566, 0.1229.

Feature Contribution

Top features for positive contribution

Fingerprint	Bit/Smiles	Feature Structure	Score	Multiple-Carcinogen in training set
FCFP_12	427906732	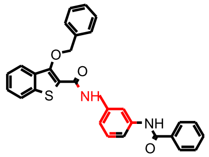 [*]NC[c]1:[cH]:[cH]:[*]:[c]([*]):[cH]:1	0.400	1 out of 1

FCFP_12	907096426	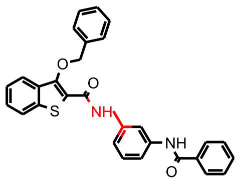<br>[*]NC[c](:[*]):[*]	0.400	1 out of 1
FCFP_12	-581879738	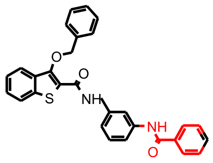<br>[*]NC(=O)[c]1:[cH]:[c<br>H]:[*]:[cH]:[cH]:1	0.239	2 out of 4

Top Features for negative contribution

**Fingerprint**	**Bit/Smiles**	**Feature Structure**	**Score**	**Multiple-Carcinogen in training set**
FCFP_12	1294255210	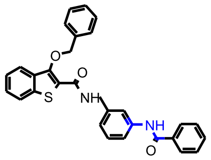<br>[*]C(=[*])N[c](:[*]):<br>[*]	-1.626	0 out of 12
FCFP_12	590925877	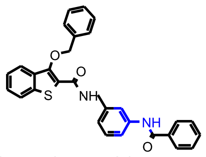<br>[*]N[c](:[cH]:[*]):[c<br>H]:[*]	-0.998	1 out of 13
FCFP_12	178336375	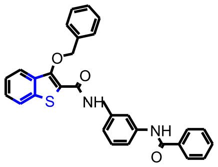<br>[*]:[cH]:[c]1:s:[*]:[<br>*]:[c]:1:[*]	-0.994	0 out of 5

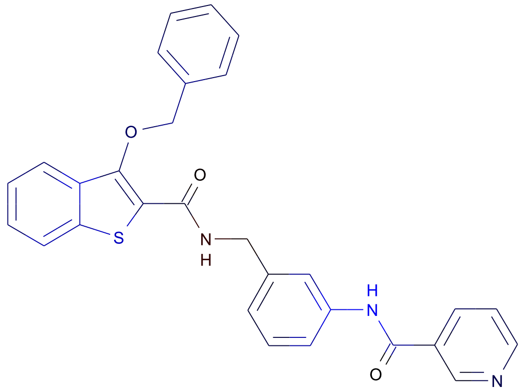

$C_{29}H_{23}N_3O_3S$
Molecular Weight: 493.57622
ALogP: 4.976
Rotatable Bonds: 8
Acceptors: 4
Donors: 2

Model Prediction

Prediction: Single-Carcinogen
Probability: 0.146
Enrichment: 0.484
Bayesian Score: -13.940
Mahalanobis Distance: 20.711
Mahalanobis Distance p-value: 1.34e-010

Prediction: Positive if the Bayesian score is above the estimated best cutoff value from minimizing the false positive and false negative rate.
Probability: The esimated probability that the sample is in the positive category. This assumes that the Bayesian score follows a normal distribution and is different from the prediction using a cutoff.
Enrichment: An estimate of enrichment, that is, the increased likelihood (versus random) of this sample being in the category.
Bayesian Score: The standard Laplacian-modified Bayesian score.
Mahalanobis Distance: The Mahalanobis distance (MD) is the distance to the center of the training data. The larger the MD, the less trustworthy the prediction.
Mahalanobis Distance p-value: The p-value gives the fraction of training data with an MD greater than or equal to the one for the given sample, assuming normally distributed data. The smaller the p-value, the less trustworthy the prediciton. For highly non-normal X properties (e.g., fingerprints), the MD p-value is wildly inaccurate.

Structural Similar Compounds

Name	Glimepride	Bicalutamide	Simvastatin
Structure	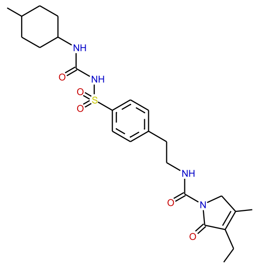	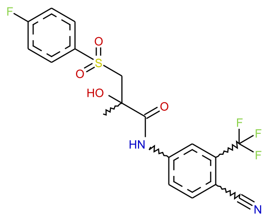	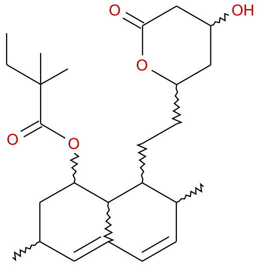
Actual Endpoint	Single-Carcinogen	Single-Carcinogen	Multiple-Carcinogen
Predicted Endpoint	Single-Carcinogen	Single-Carcinogen	Multiple-Carcinogen
Distance	0.729	0.740	0.764
Reference	US FDA (Centre for Drug Eval.& Res./Off. Testing & Res.) Sept. 1997	US FDA (Centre for Drug Eval.& Res./Off. Testing & Res.) Sept. 1997	US FDA (Centre for Drug Eval.& Res./Off. Testing & Res.) Sept. 1997

Model Applicability

Unknown features are fingerprint features in the query molecule, but not found in the training set.

1. OPS PC2 out of range. Value: 5.4108. Training min, max, SD, explained variance: -5.2888, 4.2744, 2.566, 0.1229.

Feature Contribution

Top features for positive contribution

Fingerprint	Bit/Smiles	Feature Structure	Score	Multiple-Carcinogen in training set
FCFP_12	427906732	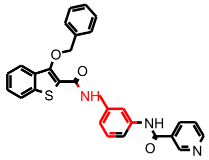 [*]NC[c]1:[cH]:[cH]:[*]:[c]([*]):[cH]:1	0.400	1 out of 1

FCFP_12	547884906	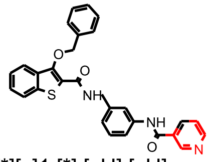 [*][c]1:[*]:[cH]:[cH]:n:[cH]:1	0.400	1 out of 1
FCFP_12	907096426	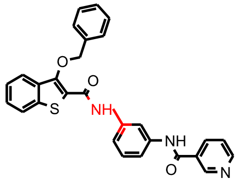 [*]NC[c](:[*]):[*]	0.400	1 out of 1

Top Features for negative contribution

Fingerprint	Bit/Smiles	Feature Structure	Score	Multiple-Carcinogen in training set
FCFP_12	1294255210	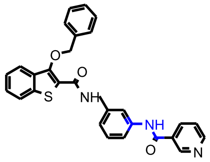 [*]C(=[*])N[c](:[*]):[*]	-1.626	0 out of 12
FCFP_12	590925877	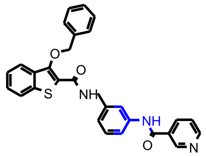 [*]N[c](:[cH]:[*]):[cH]:[*]	-0.998	1 out of 13
FCFP_12	178336375	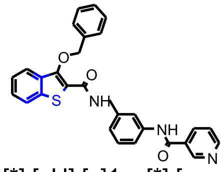 [*]:[cH]:[c]1:s:[*]:[*]:[c]:1:[*]	-0.994	0 out of 5

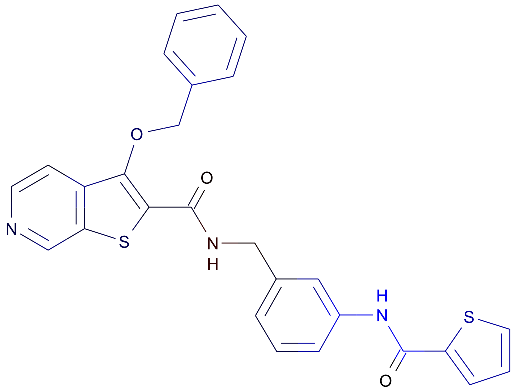

$C_{27}H_{21}N_3O_3S_2$

Molecular Weight: 499.60394

ALogP: 4.929

Rotatable Bonds: 8

Acceptors: 4

Donors: 2

Model Prediction

Prediction: Single-Carcinogen

Probability: 0.138

Enrichment: 0.460

Bayesian Score: -14.779

Mahalanobis Distance: 20.892

Mahalanobis Distance p-value: 1e-010

Prediction: Positive if the Bayesian score is above the estimated best cutoff value from minimizing the false positive and false negative rate.
Probability: The esimated probability that the sample is in the positive category. This assumes that the Bayesian score follows a normal distribution and is different from the prediction using a cutoff.
Enrichment: An estimate of enrichment, that is, the increased likelihood (versus random) of this sample being in the category.
Bayesian Score: The standard Laplacian-modified Bayesian score.
Mahalanobis Distance: The Mahalanobis distance (MD) is the distance to the center of the training data. The larger the MD, the less trustworthy the prediction.
Mahalanobis Distance p-value: The p-value gives the fraction of training data with an MD greater than or equal to the one for the given sample, assuming normally distributed data. The smaller the p-value, the less trustworthy the prediciton. For highly non-normal X properties (e.g., fingerprints), the MD p-value is wildly inaccurate.

Structural Similar Compounds

Name	Glimepride	Bicalutamide	Simvastatin
Structure	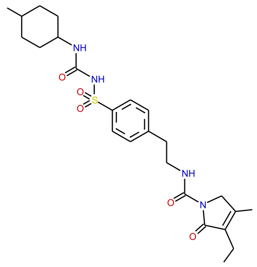	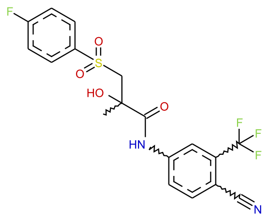	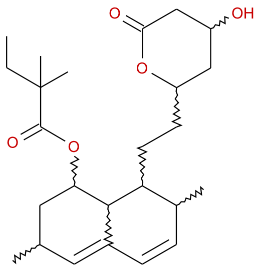
Actual Endpoint	Single-Carcinogen	Single-Carcinogen	Multiple-Carcinogen
Predicted Endpoint	Single-Carcinogen	Single-Carcinogen	Multiple-Carcinogen
Distance	0.724	0.733	0.798
Reference	US FDA (Centre for Drug Eval.& Res./Off. Testing & Res.) Sept. 1997	US FDA (Centre for Drug Eval.& Res./Off. Testing & Res.) Sept. 1997	US FDA (Centre for Drug Eval.& Res./Off. Testing & Res.) Sept. 1997

Model Applicability

Unknown features are fingerprint features in the query molecule, but not found in the training set.

1. OPS PC2 out of range. Value: 5.424. Training min, max, SD, explained variance: -5.2888, 4.2744, 2.566, 0.1229.

Feature Contribution

Top features for positive contribution

Fingerprint	Bit/Smiles	Feature Structure	Score	Multiple-Carcinogen in training set
FCFP_12	427906732	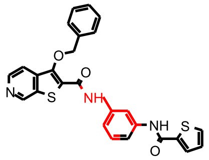 [*]NC[c]1:[cH]:[cH]:[*]:[c]([*]):[cH]:1	0.400	1 out of 1

FCFP_12	907096426	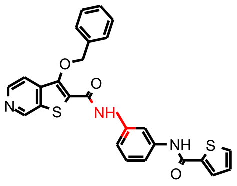 [*]NC[c](:[*]):[*]	0.400	1 out of 1
FCFP_12	547884906	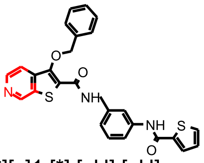 [*][c]1:[*]:[cH]:[cH]:n:[cH]:1	0.400	1 out of 1
**Top Features for negative contribution**				
**Fingerprint**	**Bit/Smiles**	**Feature Structure**	**Score**	**Multiple-Carcinogen in training set**
FCFP_12	1294255210	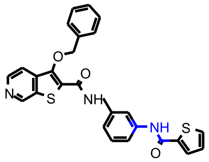 [*]C(=[*])N[c](:[*]):[*]	-1.626	0 out of 12
FCFP_12	590925877	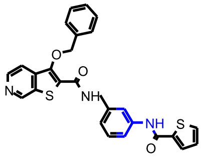 [*]N[c](:[cH]:[*]):[cH]:[*]	-0.998	1 out of 13
FCFP_12	-1462709112	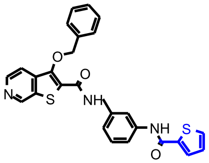 [*]C(=[*])[c]1:[cH]:[cH]:[cH]:s:1	-0.994	0 out of 5

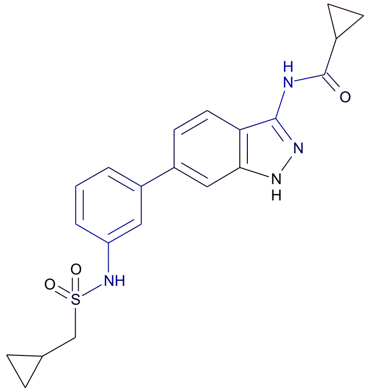

$C_{21}H_{22}N_4O_3S$
Molecular Weight: 410.48938
ALogP: 3.14
Rotatable Bonds: 7
Acceptors: 4
Donors: 3

Model Prediction

Prediction: Single-Carcinogen
Probability: 0.146
Enrichment: 0.484
Bayesian Score: -9.555
Mahalanobis Distance: 14.646
Mahalanobis Distance p-value: 4.8e-006

Prediction: Positive if the Bayesian score is above the estimated best cutoff value from minimizing the false positive and false negative rate.
Probability: The esimated probability that the sample is in the positive category. This assumes that the Bayesian score follows a normal distribution and is different from the prediction using a cutoff.
Enrichment: An estimate of enrichment, that is, the increased likelihood (versus random) of this sample being in the category.
Bayesian Score: The standard Laplacian-modified Bayesian score.
Mahalanobis Distance: The Mahalanobis distance (MD) is the distance to the center of the training data. The larger the MD, the less trustworthy the prediction.
Mahalanobis Distance p-value: The p-value gives the fraction of training data with an MD greater than or equal to the one for the given sample, assuming normally distributed data. The smaller the p-value, the less trustworthy the prediciton. For highly non-normal X properties (e.g., fingerprints), the MD p-value is wildly inaccurate.

Structural Similar Compounds

Name	Glimepride	Bicalutamide	Primidolol
Structure	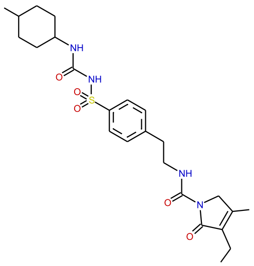	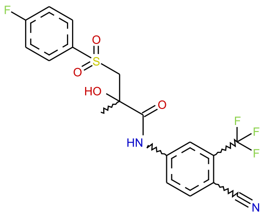	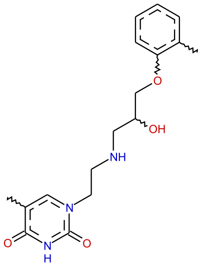
Actual Endpoint	Single-Carcinogen	Single-Carcinogen	Single-Carcinogen
Predicted Endpoint	Single-Carcinogen	Single-Carcinogen	Single-Carcinogen
Distance	0.667	0.692	0.751
Reference	US FDA (Centre for Drug Eval.& Res./Off. Testing & Res.) Sept. 1997	US FDA (Centre for Drug Eval.& Res./Off. Testing & Res.) Sept. 1997	US FDA (Centre for Drug Eval.& Res./Off. Testing & Res.) Sept. 1997

Model Applicability

Unknown features are fingerprint features in the query molecule, but not found in the training set.

1. All properties and OPS components are within expected ranges.
2. Unknown FCFP_2 feature: 262592487: [*]:[c]1:[*]:[*]:n:[nH]:1
3. Unknown FCFP_2 feature: 1747267175: [*][c]1:[*]:[*]:[nH]:n:1
4. Unknown FCFP_2 feature: 1018942292: [*]CS(=O)(=O)N[*]

Feature Contribution

Top features for positive contribution

Fingerprint	Bit/Smiles	Feature Structure	Score	Multiple-Carcinogen in training set
FCFP_12	-1043339860	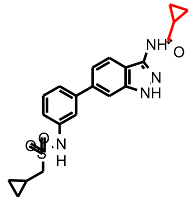 [*]C(=[*])C1CC1	0.349	12 out of 24

FCFP_12	-55265897	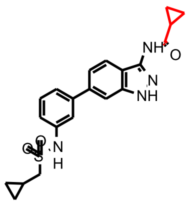 [*]C(=[*])C1CC1	0.282	8 out of 17
FCFP_12	307448885	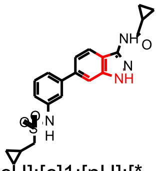 [*]:[cH]:[c]1:[nH]:[*]:[*]:[c]:1:[*]	0.174	1 out of 2

Top Features for negative contribution

Fingerprint	Bit/Smiles	Feature Structure	Score	Multiple-Carcinogen in training set
FCFP_12	1294255210	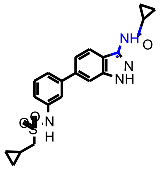 [*]C(=[*])N[c](:[*]):[*]	-1.626	0 out of 12
FCFP_12	-1151884458	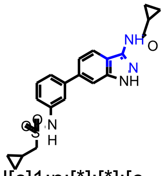 [*]N[c]1:n:[*]:[*]:[c]:1:[*]	-1.112	0 out of 6
FCFP_12	590925877	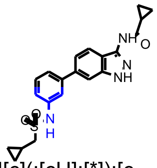 [*]N[c](:[cH]:[*]):[cH]:[*]	-0.998	1 out of 13

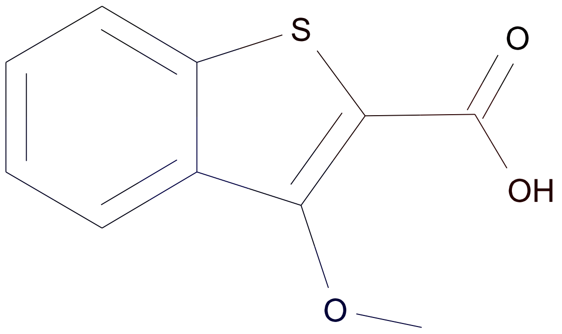

$C_{10}H_8O_3S$
Molecular Weight: 208.23372
ALogP: 2.596
Rotatable Bonds: 2
Acceptors: 3
Donors: 1

Model Prediction

Prediction: Mild
Probability: 0.802
Enrichment: 1.164
Bayesian Score: -0.938
Mahalanobis Distance: 9.900
Mahalanobis Distance p-value: 0.119

Prediction: Positive if the Bayesian score is above the estimated best cutoff value from minimizing the false positive and false negative rate.
Probability: The esimated probability that the sample is in the positive category. This assumes that the Bayesian score follows a normal distribution and is different from the prediction using a cutoff.
Enrichment: An estimate of enrichment, that is, the increased likelihood (versus random) of this sample being in the category.
Bayesian Score: The standard Laplacian-modified Bayesian score.
Mahalanobis Distance: The Mahalanobis distance (MD) is the distance to the center of the training data. The larger the MD, the less trustworthy the prediction.
Mahalanobis Distance p-value: The p-value gives the fraction of training data with an MD greater than or equal to the one for the given sample, assuming normally distributed data. The smaller the p-value, the less trustworthy the prediciton. For highly non-normal X properties (e.g., fingerprints), the MD p-value is wildly inaccurate.

Structural Similar Compounds

Name	PHENOL;2;4-DICHLORO-6-NITRO	Furil; monooxime	BENZOIC ACID;2-CHLORO-4-NITRO-
Structure	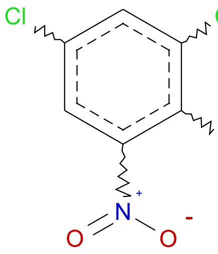	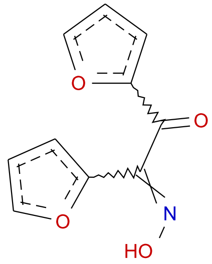	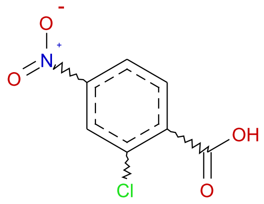
Actual Endpoint	Moderate_Severe	Moderate_Severe	Moderate_Severe
Predicted Endpoint	Moderate_Severe	Moderate_Severe	Moderate_Severe
Distance	0.485	0.495	0.580
Reference	28ZPAK-;80;72	Prehled Prumyslove Toxikologie; Organicke Latky; Marhold; J. -;793;86	28ZPAK-;91;72

Model Applicability

Unknown features are fingerprint features in the query molecule, but not found in the training set.

1. All properties and OPS components are within expected ranges.

Feature Contribution

Top features for positive contribution

Fingerprint	Bit/Smiles	Feature Structure	Score	Moderate_Severe in training set
FCFP_10	-548632217	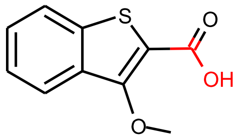 [*]C(=[*])O	0.319	54 out of 59

FCFP_10	2047994594	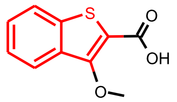 [*][c]1:s:[c]2:[cH]:[cH]:[cH]:[cH]:[c]:2:[c]:1[*]	0.294	3 out of 3
FCFP_10	-1716224640	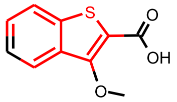 [*][c]1:s:[c]2:[cH]:[cH]:[*]:[cH]:[c]:2:[c]:1[*]	0.294	3 out of 3

Top Features for negative contribution

Fingerprint	Bit/Smiles	Feature Structure	Score	Moderate_Severe in training set
FCFP_10	-1977641857	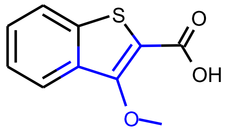 [*][c]1:[*]:[*]:[c](:[*]):[c]:1OC	-0.780	4 out of 15
FCFP_10	-1320007763	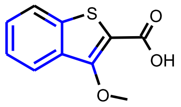 [*][c]1:[*]:[*]:[c]2:[*]:[cH]:[cH]:[cH]:[c]:1:2	-0.316	19 out of 40
FCFP_10	136627117	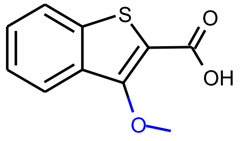 [*]OC	-0.316	46 out of 96

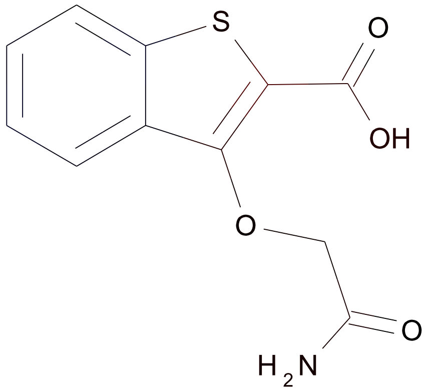

$C_{11}H_9NO_4S$
Molecular Weight: 251.25846
ALogP: 1.45
Rotatable Bonds: 4
Acceptors: 4
Donors: 2

Model Prediction

Prediction: Moderate_Severe
Probability: 0.833
Enrichment: 1.208
Bayesian Score: 0.702
Mahalanobis Distance: 10.798
Mahalanobis Distance p-value: 0.00969

Prediction: Positive if the Bayesian score is above the estimated best cutoff value from minimizing the false positive and false negative rate.
Probability: The esimated probability that the sample is in the positive category. This assumes that the Bayesian score follows a normal distribution and is different from the prediction using a cutoff.
Enrichment: An estimate of enrichment, that is, the increased likelihood (versus random) of this sample being in the category.
Bayesian Score: The standard Laplacian-modified Bayesian score.
Mahalanobis Distance: The Mahalanobis distance (MD) is the distance to the center of the training data. The larger the MD, the less trustworthy the prediction.
Mahalanobis Distance p-value: The p-value gives the fraction of training data with an MD greater than or equal to the one for the given sample, assuming normally distributed data. The smaller the p-value, the less trustworthy the prediciton. For highly non-normal X properties (e.g., fingerprints), the MD p-value is wildly inaccurate.

Structural Similar Compounds

Name	1-NAPHTHALENESULFONIC ACID; 2-AMINO-	2-NAPHTHALENE SULFONIC ACID;5-AMINO-	NAPHTHENESULFONIC ACID;2-HYDROXY-
Structure	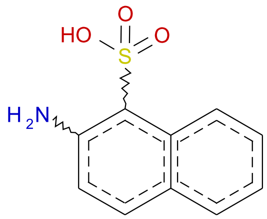	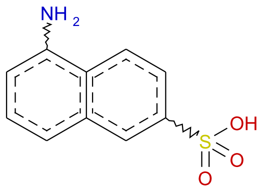	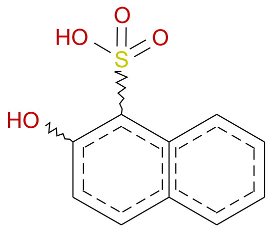
Actual Endpoint	Mild	Mild	Moderate_Severe
Predicted Endpoint	Mild	Mild	Moderate_Severe
Distance	0.543	0.556	0.557
Reference	28ZPAK-;187;72	28ZPAK-;187;72	28ZPAK-;186;72

Model Applicability

Unknown features are fingerprint features in the query molecule, but not found in the training set.

1. All properties and OPS components are within expected ranges.

Feature Contribution

Top features for positive contribution

Fingerprint	Bit/Smiles	Feature Structure	Score	Moderate_Severe in training set
FCFP_10	-548632217	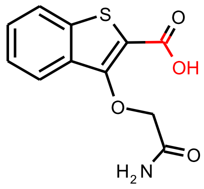 [*]C(=[*])O	0.319	54 out of 59

FCFP_10	2047994594	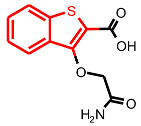<br>[*][c]1:s:[c]2:[cH]:[cH]:[cH]:[cH]:[c]:2:[c]:1[*]	0.294	3 out of 3
FCFP_10	-1716224640	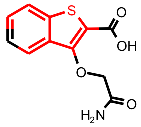<br>[*][c]1:s:[c]2:[cH]:[cH]:[*]:[cH]:[c]:2:[c]:1[*]	0.294	3 out of 3

Top Features for negative contribution

Fingerprint	Bit/Smiles	Feature Structure	Score	Moderate_Severe in training set
FCFP_10	-1320007763	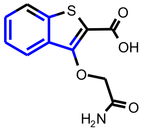<br>[*][c]1:[*]:[*]:[c]2:[*]:[cH]:[cH]:[cH]:[c]:1:2	-0.316	19 out of 40
FCFP_10	713358128	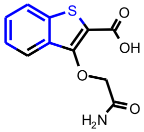<br>[*]1:[*]:[c]2:[*]:[cH]:[cH]:[cH]:[c]:2:s:1	-0.307	8 out of 17
FCFP_10	307419094	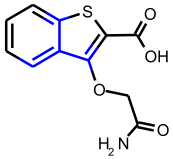<br>[*][c]1:[*]:[*]:[c](:[*]):[c]:1:[cH]:[*]	-0.290	21 out of 43

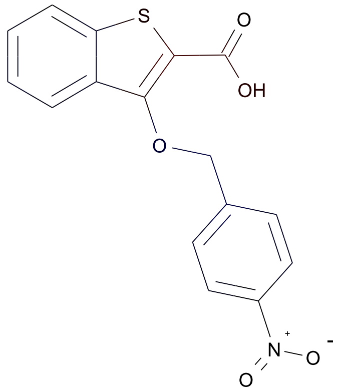

$C_{16}H_{11}NO_5S$
Molecular Weight: 329.32724
ALogP: 4.074
Rotatable Bonds: 5
Acceptors: 5
Donors: 1

Structural Similar Compounds

Name	BENZOIC ACID; 5-(CHLOROSULFONYL)-2;4-DICHLORO-	PHENOL;2-SEC-BUTYL-4;6-DINITRO-	ETHER; (p-NITROPHENYL) (alpha;alpha;alpha-TRIFLUORO-2-NITRO-p-TOLYL)
Structure	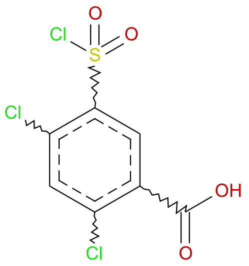	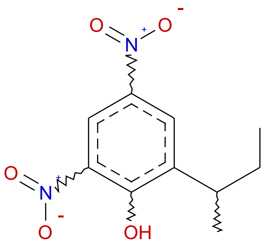	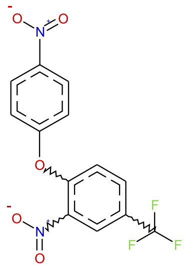
Actual Endpoint	Moderate_Severe	Moderate_Severe	Moderate_Severe
Predicted Endpoint	Moderate_Severe	Moderate_Severe	Moderate_Severe
Distance	0.660	0.664	0.703
Reference	FCTOD7 20;573;82	28ZPAK-;108;72	CIGET* -;-;77

Model Prediction

Prediction: Mild
Probability: 0.798
Enrichment: 1.158
Bayesian Score: -1.079
Mahalanobis Distance: 9.407
Mahalanobis Distance p-value: 0.301

Prediction: Positive if the Bayesian score is above the estimated best cutoff value from minimizing the false positive and false negative rate.
Probability: The esimated probability that the sample is in the positive category. This assumes that the Bayesian score follows a normal distribution and is different from the prediction using a cutoff.
Enrichment: An estimate of enrichment, that is, the increased likelihood (versus random) of this sample being in the category.
Bayesian Score: The standard Laplacian-modified Bayesian score.
Mahalanobis Distance: The Mahalanobis distance (MD) is the distance to the center of the training data. The larger the MD, the less trustworthy the prediction.
Mahalanobis Distance p-value: The p-value gives the fraction of training data with an MD greater than or equal to the one for the given sample, assuming normally distributed data. The smaller the p-value, the less trustworthy the prediciton. For highly non-normal X properties (e.g., fingerprints), the MD p-value is wildly inaccurate.

Model Applicability

Unknown features are fingerprint features in the query molecule, but not found in the training set.

1. All properties and OPS components are within expected ranges.
2. Unknown FCFP_2 feature: -828984032: [*][N+](=[*])[c](:[cH]:[*]):[cH]:[*]
3. Unknown FCFP_2 feature: -1338588315: [*]:[c](:[*])[N+](=O)[O-]
4. Unknown FCFP_2 feature: 1872392852: [*][N+](=O)[*]
5. Unknown FCFP_2 feature: 260476081: [*][N+](=[*])[O-]

Feature Contribution

Top features for positive contribution				
**Fingerprint**	**Bit/Smiles**	**Feature Structure**	**Score**	**Moderate_Severe in training set**
FCFP_10	-548632217	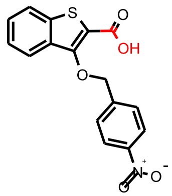 [*]C(=[*])O	0.319	54 out of 59

FCFP_10	2047994594	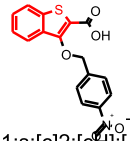 [*][c]1:s:[c]2:[cH]:[cH]:[cH]:[cH]:[c]:2:[c]:1[*]	0.294	3 out of 3
FCFP_10	-1716224640	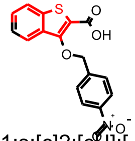 [*][c]1:s:[c]2:[cH]:[cH]:[*]:[cH]:[c]:2:[c]:1[*]	0.294	3 out of 3

Top Features for negative contribution

Fingerprint	Bit/Smiles	Feature Structure	Score	Moderate_Severe in training set
FCFP_10	907036844	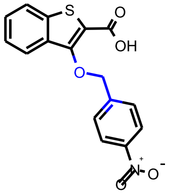 [*]OC[c](:[*]):[*]	-0.600	1 out of 4
FCFP_10	2106393770	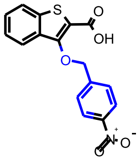 [*]OC[c]1:[cH]:[cH]:[*]:[cH]:[cH]:1	-0.400	1 out of 3
FCFP_10	-1320007763	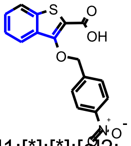 [*][c]1:[*]:[*]:[c]2:[*]:[cH]:[cH]:[cH]:[c]:1:2	-0.316	19 out of 40

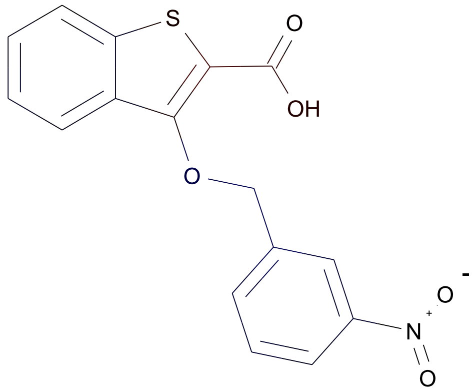

$C_{16}H_{11}NO_5S$

Molecular Weight: 329.32724

ALogP: 4.074

Rotatable Bonds: 5

Acceptors: 5

Donors: 1

Structural Similar Compounds

Name	BENZOIC ACID; 5-(CHLOROSULFONYL)-2;4-DICHLORO-	PHENOL;2-SEC-BUTYL-4;6-DINITRO-	ETHER; (p-NITROPHENYL) (alpha;alpha;alpha-TRIFLUORO-2-NITRO-p-TOLYL)
Structure	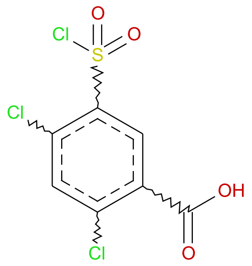	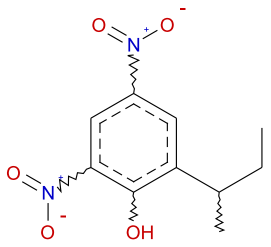	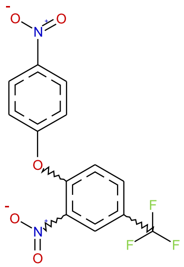
Actual Endpoint	Moderate_Severe	Moderate_Severe	Moderate_Severe
Predicted Endpoint	Moderate_Severe	Moderate_Severe	Moderate_Severe
Distance	0.660	0.665	0.704
Reference	FCTOD7 20;573;82	28ZPAK-;108;72	CIGET* -;-;77

Model Prediction

Prediction: Mild

Probability: 0.787

Enrichment: 1.142

Bayesian Score: -1.478

Mahalanobis Distance: 9.407

Mahalanobis Distance p-value: 0.301

Prediction: Positive if the Bayesian score is above the estimated best cutoff value from minimizing the false positive and false negative rate.
Probability: The esimated probability that the sample is in the positive category. This assumes that the Bayesian score follows a normal distribution and is different from the prediction using a cutoff.
Enrichment: An estimate of enrichment, that is, the increased likelihood (versus random) of this sample being in the category.
Bayesian Score: The standard Laplacian-modified Bayesian score.
Mahalanobis Distance: The Mahalanobis distance (MD) is the distance to the center of the training data. The larger the MD, the less trustworthy the prediction.
Mahalanobis Distance p-value: The p-value gives the fraction of training data with an MD greater than or equal to the one for the given sample, assuming normally distributed data. The smaller the p-value, the less trustworthy the prediciton. For highly non-normal X properties (e.g., fingerprints), the MD p-value is wildly inaccurate.

Model Applicability

Unknown features are fingerprint features in the query molecule, but not found in the training set.

1. All properties and OPS components are within expected ranges.
2. Unknown FCFP_2 feature: -828984032: [*][N+](=[*])[c](:[cH]:[*]):[cH]:[*]
3. Unknown FCFP_2 feature: -1338588315: [*]:[c](:[*])[N+](=O)[O-]
4. Unknown FCFP_2 feature: 1872392852: [*][N+](=O)[*]
5. Unknown FCFP_2 feature: 260476081: [*][N+](=[*])[O-]

Feature Contribution

Top features for positive contribution

Fingerprint	Bit/Smiles	Feature Structure	Score	Moderate_Severe in training set
FCFP_10	-548632217	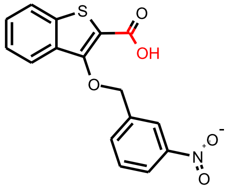 [*]C(=[*])O	0.319	54 out of 59

FCFP_10	-1716224640	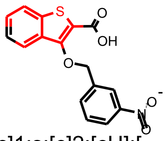 [*][c]1:s:[c]2:[cH]:[cH]:[*]:[cH]:[c]:2:[c]:1[*]	0.294	3 out of 3
FCFP_10	2047994594	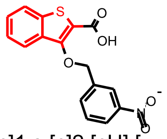 [*][c]1:s:[c]2:[cH]:[cH]:[cH]:[cH]:[c]:2:[c]:1[*]	0.294	3 out of 3

Top Features for negative contribution

Fingerprint	Bit/Smiles	Feature Structure	Score	Moderate_Severe in training set
FCFP_10	907036844	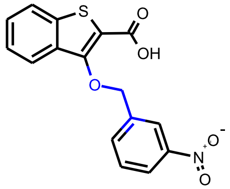 [*]OC[c](:[*]):[*]	-0.600	1 out of 4
FCFP_10	1390842262	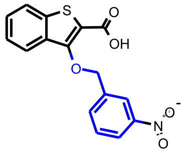 [*]OC[c]1:[cH]:[cH]:[cH]:[c]([*]):[cH]:1	-0.400	1 out of 3
FCFP_10	2106393770	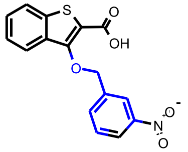 [*]OC[c]1:[cH]:[cH]:[*]:[cH]:[cH]:1	-0.400	1 out of 3

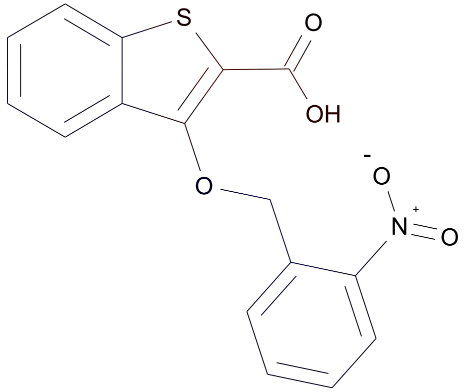

$C_{16}H_{11}NO_5S$
Molecular Weight: 329.32724
ALogP: 4.074
Rotatable Bonds: 5
Acceptors: 5
Donors: 1

Structural Similar Compounds

Name	BENZOIC ACID; 5-(CHLOROSULFONYL)-2;4-DICHLORO-	PHENOL;2-SEC-BUTYL-4;6-DINITRO-	ETHER; (p-NITROPHENYL) (alpha;alpha;alpha-TRIFLUORO-2-NITRO-p-TOLYL)
Structure	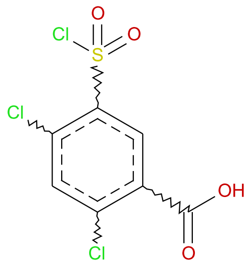	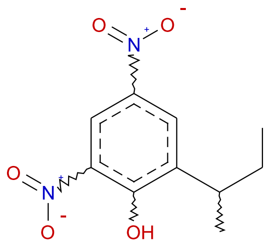	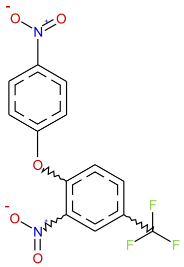
Actual Endpoint	Moderate_Severe	Moderate_Severe	Moderate_Severe
Predicted Endpoint	Moderate_Severe	Moderate_Severe	Moderate_Severe
Distance	0.659	0.664	0.703
Reference	FCTOD7 20;573;82	28ZPAK-;108;72	CIGET* -;-;77

Model Prediction

Prediction: Mild
Probability: 0.801
Enrichment: 1.163
Bayesian Score: -0.963
Mahalanobis Distance: 9.407
Mahalanobis Distance p-value: 0.301

Prediction: Positive if the Bayesian score is above the estimated best cutoff value from minimizing the false positive and false negative rate.
Probability: The esimated probability that the sample is in the positive category. This assumes that the Bayesian score follows a normal distribution and is different from the prediction using a cutoff.
Enrichment: An estimate of enrichment, that is, the increased likelihood (versus random) of this sample being in the category.
Bayesian Score: The standard Laplacian-modified Bayesian score.
Mahalanobis Distance: The Mahalanobis distance (MD) is the distance to the center of the training data. The larger the MD, the less trustworthy the prediction.
Mahalanobis Distance p-value: The p-value gives the fraction of training data with an MD greater than or equal to the one for the given sample, assuming normally distributed data. The smaller the p-value, the less trustworthy the prediciton. For highly non-normal X properties (e.g., fingerprints), the MD p-value is wildly inaccurate.

Model Applicability

Unknown features are fingerprint features in the query molecule, but not found in the training set.

1. All properties and OPS components are within expected ranges.
2. Unknown FCFP_2 feature: -828984032: [*][N+](=[*])[c](:[cH]:[*]):[cH]:[*]
3. Unknown FCFP_2 feature: -1338588315: [*]:[c](:[*])[N+](=O)[O-]
4. Unknown FCFP_2 feature: 1872392852: [*][N+](=O)[*]
5. Unknown FCFP_2 feature: 260476081: [*][N+](=[*])[O-]

Feature Contribution

Top features for positive contribution				
**Fingerprint**	**Bit/Smiles**	**Feature Structure**	**Score**	**Moderate_Severe in training set**
FCFP_10	-548632217	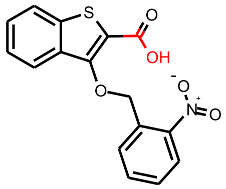 [*]C(=[*])O	0.319	54 out of 59

FCFP_10	-1716224640	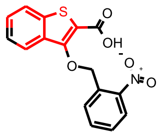 [*][c]1:s:[c]2:[cH]:[cH]:[*]:[cH]:[c]:2:[c]:1[*]	0.294	3 out of 3
FCFP_10	2047994594	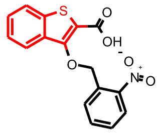 [*][c]1:s:[c]2:[cH]:[cH]:[cH]:[cH]:[c]:2: [c]:1[*]	0.294	3 out of 3

Top Features for negative contribution

**Fingerprint**	**Bit/Smiles**	**Feature Structure**	**Score**	**Moderate_Severe in training set**
FCFP_10	907036844	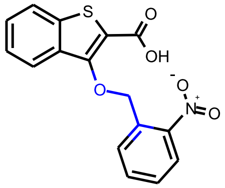 [*]OC[c](:[*]):[*]	-0.600	1 out of 4
FCFP_10	-1320007763	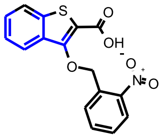 [*][c]1:[*]:[*]:[c]2: [*]:[cH]:[cH]:[cH]:[c]:1:2	-0.316	19 out of 40
FCFP_10	713358128	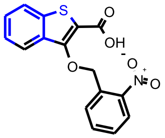 [*]1:[*]:[c]2:[*]:[cH]:[cH]:[cH]:[c]:2:s: 1	-0.307	8 out of 17

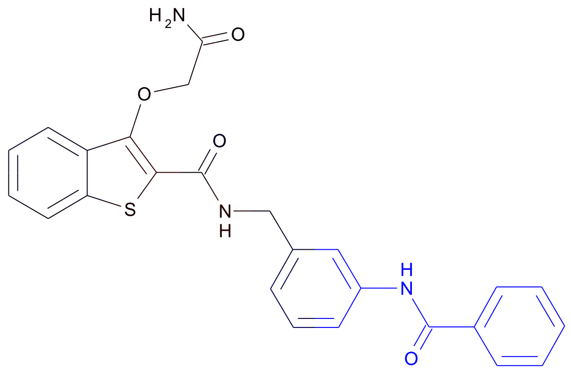

$C_{25}H_{21}N_3O_4S$
Molecular Weight: 459.51694
ALogP: 3.396
Rotatable Bonds: 8
Acceptors: 4
Donors: 3

Model Prediction

Prediction: Mild
Probability: 0.077
Enrichment: 0.112
Bayesian Score: -11.500
Mahalanobis Distance: 10.633
Mahalanobis Distance p-value: 0.0166

Prediction: Positive if the Bayesian score is above the estimated best cutoff value from minimizing the false positive and false negative rate.
Probability: The esimated probability that the sample is in the positive category. This assumes that the Bayesian score follows a normal distribution and is different from the prediction using a cutoff.
Enrichment: An estimate of enrichment, that is, the increased likelihood (versus random) of this sample being in the category.
Bayesian Score: The standard Laplacian-modified Bayesian score.
Mahalanobis Distance: The Mahalanobis distance (MD) is the distance to the center of the training data. The larger the MD, the less trustworthy the prediction.
Mahalanobis Distance p-value: The p-value gives the fraction of training data with an MD greater than or equal to the one for the given sample, assuming normally distributed data. The smaller the p-value, the less trustworthy the prediciton. For highly non-normal X properties (e.g., fingerprints), the MD p-value is wildly inaccurate.

Structural Similar Compounds

Name	1-AMINO-4-BENZOYLAMINO-ANTHRAQUINONE	4;4'-DIAMINO-1;1'-DIANTHRIMIDE	ANTHRAQUINONE; 1-((2-HYDROXYETHYL)AMINO)-4-(METHYLAMINO)-
Structure	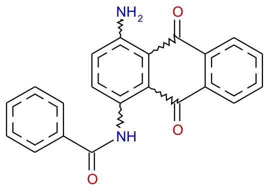	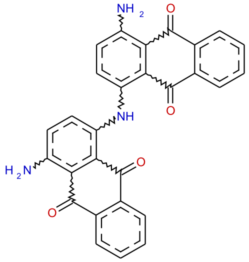	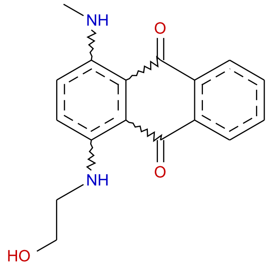
Actual Endpoint	Mild	Mild	Mild
Predicted Endpoint	Mild	Mild	Mild
Distance	0.774	0.785	0.805
Reference	28ZPAK-;124;72	28ZPAK-;125;72	28ZPAK 245;72

Model Applicability

Unknown features are fingerprint features in the query molecule, but not found in the training set.

1. All properties and OPS components are within expected ranges.

Feature Contribution

Top features for positive contribution				
**Fingerprint**	**Bit/Smiles**	**Feature Structure**	**Score**	**Moderate_Severe in training set**
FCFP_10	907096426	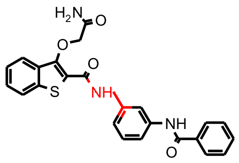 [*]NC[c](:[*]):[*]	0.332	5 out of 5

FCFP_10	427906732	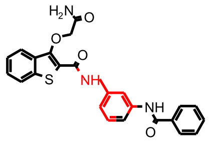 [*]NC[c]1:[cH]:[cH]:[*]:[c]([*]):[cH]:1	0.294	3 out of 3
FCFP_10	2047994594	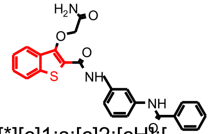 [*][c]1:s:[c]2:[cH]:[cH]:[cH]:[cH]:[c]:2: [c]:1[*]	0.294	3 out of 3
**Top Features for negative contribution**				
**Fingerprint**	**Bit/Smiles**	**Feature Structure**	**Score**	**Moderate_Severe in training set**
FCFP_10	-1112960996	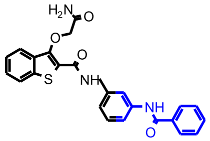 [*]:[cH]:[c](NC(=O)[c]1:[cH]:[cH]:[cH]:[c H]:[cH]:1):[cH]:[*]	-1.293	0 out of 4
FCFP_10	-1205069278	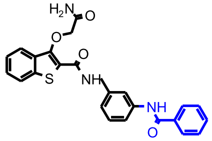 [*]NC(=O)[c]1:[cH]:[c H]:[cH]:[cH]:[cH]:1	-1.293	0 out of 4
FCFP_10	384221478	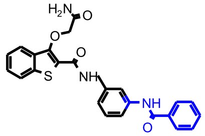 [*]:[c](:[*])NC(=O)[c]1:[cH]:[cH]:[cH]:[c H]:[cH]:1	-1.293	0 out of 4

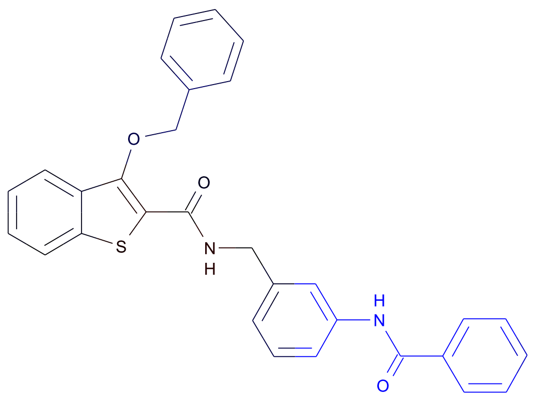

$C_{30}H_{24}N_2O_3S$
Molecular Weight: 492.58816
ALogP: 6.126
Rotatable Bonds: 8
Acceptors: 3
Donors: 2

Structural Similar Compounds

Name	ANTHRAQUINONE; 1;4-BIS(p-TOLYLAMINO)-	DINAPHTHO(1;2;3-CD:3';2';1'-IM)PERYLENE-5;10-DIONE;16;17-DIHYDROXY	2-(1'-ANTHRAQUINONYL)-AMINOBENZANTHRONE
Structure	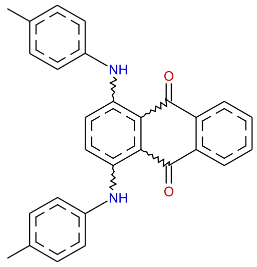	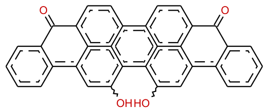	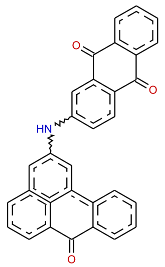
Actual Endpoint	Moderate_Severe	Mild	Mild
Predicted Endpoint	Mild	Mild	Mild
Distance	0.681	0.719	0.774
Reference	28ZPAK -;124;72	28ZPAK-;104;72	28ZPAK-;126;72

Model Prediction

Prediction: Mild
Probability: 0.024
Enrichment: 0.035
Bayesian Score: -13.233
Mahalanobis Distance: 8.377
Mahalanobis Distance p-value: 0.814

Prediction: Positive if the Bayesian score is above the estimated best cutoff value from minimizing the false positive and false negative rate.
Probability: The esimated probability that the sample is in the positive category. This assumes that the Bayesian score follows a normal distribution and is different from the prediction using a cutoff.
Enrichment: An estimate of enrichment, that is, the increased likelihood (versus random) of this sample being in the category.
Bayesian Score: The standard Laplacian-modified Bayesian score.
Mahalanobis Distance: The Mahalanobis distance (MD) is the distance to the center of the training data. The larger the MD, the less trustworthy the prediction.
Mahalanobis Distance p-value: The p-value gives the fraction of training data with an MD greater than or equal to the one for the given sample, assuming normally distributed data. The smaller the p-value, the less trustworthy the prediciton. For highly non-normal X properties (e.g., fingerprints), the MD p-value is wildly inaccurate.

Model Applicability

Unknown features are fingerprint features in the query molecule, but not found in the training set.

1. All properties and OPS components are within expected ranges.

Feature Contribution

Top features for positive contribution

Fingerprint	Bit/Smiles	Feature Structure	Score	Moderate_Severe in training set
FCFP_10	907096426	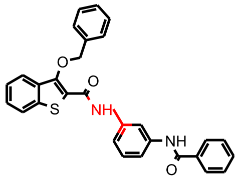 [*]NC[c](:[*]):[*]	0.332	5 out of 5

FCFP_10	427906732	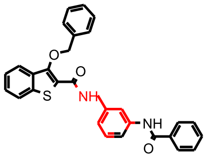<br>[*]NC[c]1:[cH]:[cH]:[<br>*]:[c]([*]):[cH]:1	0.294	3 out of 3
FCFP_10	-1716224640	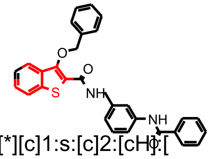<br>[*][c]1:s:[c]2:[cH]:[<br>cH]:[*]:[cH]:[c]:2:[<br>c]:1[*]	0.294	3 out of 3
**Top Features for negative contribution**				
**Fingerprint**	**Bit/Smiles**	**Feature Structure**	**Score**	**Moderate_Severe in training set**
FCFP_10	241406177	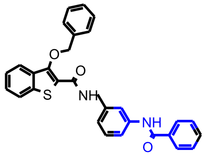<br>[*]:[cH]:[c](NC(=O)[c<br>]1:[cH]:[cH]:[*]:[cH<br>]:[cH]:1):[cH]:[*]	-1.293	0 out of 4
FCFP_10	384221478	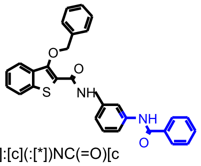<br>[*]:[c](:[*])NC(=O)[c<br>]1:[cH]:[cH]:[cH]:[c<br>H]:[cH]:1	-1.293	0 out of 4
FCFP_10	-1205069278	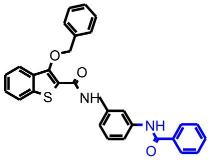<br>[*]NC(=O)[c]1:[cH]:[c<br>H]:[cH]:[cH]:[cH]:1	-1.293	0 out of 4

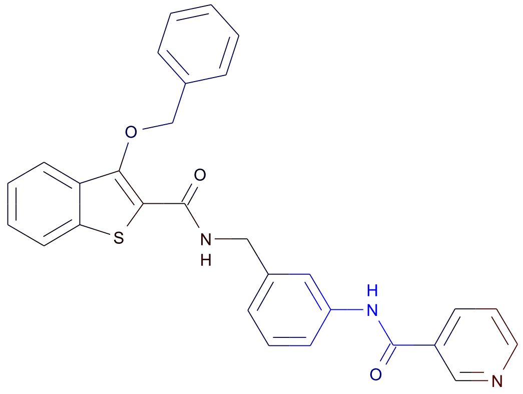

$C_{29}H_{23}N_3O_3S$
Molecular Weight: 493.57622
ALogP: 4.976
Rotatable Bonds: 8
Acceptors: 4
Donors: 2

Structural Similar Compounds

Name	ANTHRAQUINONE; 1;4-BIS(p-TOLYLAMINO)-	DINAPHTHO(1;2;3-CD:3';2';1'-IM)PERYLENE-5;10-DIONE;16;17-DIHYDROXY	1-BENZOYLAMINO-4-METHOXY-5-CHLORANTHRAQUINONE
Structure	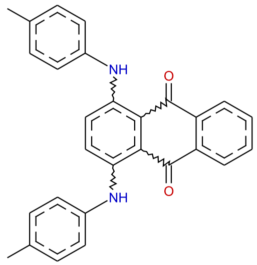	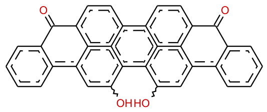	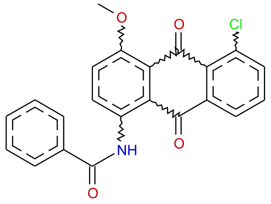
Actual Endpoint	Moderate_Severe	Mild	Mild
Predicted Endpoint	Mild	Mild	Mild
Distance	0.743	0.754	0.775
Reference	28ZPAK -;124;72	28ZPAK-;104;72	28ZPAK-;90;72

Model Prediction

Prediction: Mild
Probability: 0.558
Enrichment: 0.810
Bayesian Score: -5.522
Mahalanobis Distance: 7.848
Mahalanobis Distance p-value: 0.951

Prediction: Positive if the Bayesian score is above the estimated best cutoff value from minimizing the false positive and false negative rate.
Probability: The esimated probability that the sample is in the positive category. This assumes that the Bayesian score follows a normal distribution and is different from the prediction using a cutoff.
Enrichment: An estimate of enrichment, that is, the increased likelihood (versus random) of this sample being in the category.
Bayesian Score: The standard Laplacian-modified Bayesian score.
Mahalanobis Distance: The Mahalanobis distance (MD) is the distance to the center of the training data. The larger the MD, the less trustworthy the prediction.
Mahalanobis Distance p-value: The p-value gives the fraction of training data with an MD greater than or equal to the one for the given sample, assuming normally distributed data. The smaller the p-value, the less trustworthy the prediciton. For highly non-normal X properties (e.g., fingerprints), the MD p-value is wildly inaccurate.

Model Applicability

Unknown features are fingerprint features in the query molecule, but not found in the training set.

1. All properties and OPS components are within expected ranges.

Feature Contribution

Top features for positive contribution

Fingerprint	Bit/Smiles	Feature Structure	Score	Moderate_Severe in training set
FCFP_10	907096426	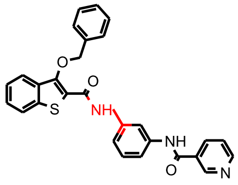 [*]NC[c](:[*]):[*]	0.332	5 out of 5

FCFP_10	547884906	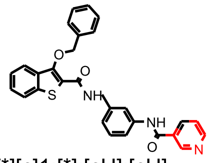 [*][c]1:[*]:[cH]:[cH]:n:[cH]:1	0.317	4 out of 4
FCFP_10	-1716224640	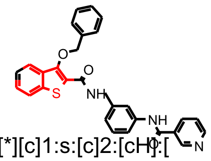 [*][c]1:s:[c]2:[cH]:[cH]:[*]:[cH]:[c]:2:[c]:1[*]	0.294	3 out of 3

Top Features for negative contribution

Fingerprint	Bit/Smiles	Feature Structure	Score	Moderate_Severe in training set
FCFP_10	-1925475824	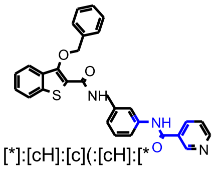 [*]:[cH]:[c](:[cH]:[*])C(=O)N[c](:[*]):[*]	-1.293	0 out of 4
FCFP_10	1175232969	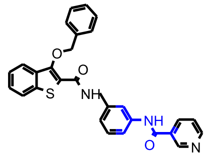 [*]:[cH]:[c](NC(=O)[c](:[*]):[*]):[cH]:[*]	-1.293	0 out of 4
FCFP_10	-1699003333	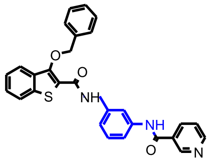 [*]C[c]1:[cH]:[cH]:[cH]:[c](N[*]):[cH]:1	-1.091	2 out of 12

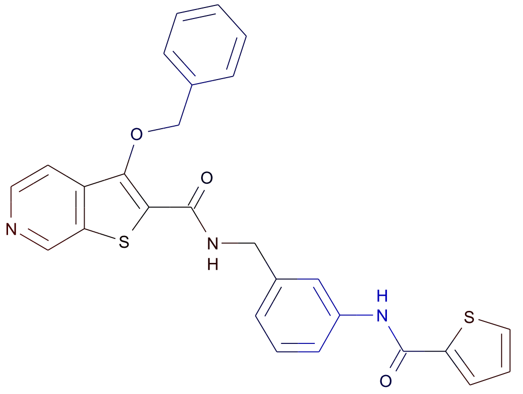

$C_{27}H_{21}N_3O_3S_2$

Molecular Weight: 499.60394

ALogP: 4.929

Rotatable Bonds: 8

Acceptors: 4

Donors: 2

Structural Similar Compounds

Name	DINAPHTHO(1;2;3-CD:3';2';1'-IM)PERYLENE-5;10-DIONE;16;17-DIHYDROXY	ANTHRAQUINONE; 1;4-BIS(p-TOLYLAMINO)-	ANTHRAQUINONE; 1;1'-IMINODI-
Structure	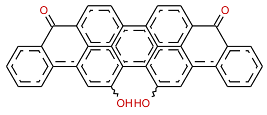	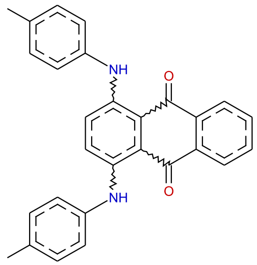	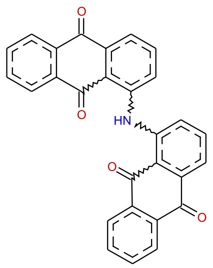
Actual Endpoint	Mild	Moderate_Severe	Mild
Predicted Endpoint	Mild	Mild	Mild
Distance	0.789	0.792	0.797
Reference	28ZPAK-;104;72	28ZPAK -;124;72	28ZPAK-;125;72

Model Prediction

Prediction: Mild

Probability: 0.708

Enrichment: 1.028

Bayesian Score: -3.371

Mahalanobis Distance: 8.130

Mahalanobis Distance p-value: 0.894

Prediction: Positive if the Bayesian score is above the estimated best cutoff value from minimizing the false positive and false negative rate.
Probability: The esimated probability that the sample is in the positive category. This assumes that the Bayesian score follows a normal distribution and is different from the prediction using a cutoff.
Enrichment: An estimate of enrichment, that is, the increased likelihood (versus random) of this sample being in the category.
Bayesian Score: The standard Laplacian-modified Bayesian score.
Mahalanobis Distance: The Mahalanobis distance (MD) is the distance to the center of the training data. The larger the MD, the less trustworthy the prediction.
Mahalanobis Distance p-value: The p-value gives the fraction of training data with an MD greater than or equal to the one for the given sample, assuming normally distributed data. The smaller the p-value, the less trustworthy the prediciton. For highly non-normal X properties (e.g., fingerprints), the MD p-value is wildly inaccurate.

Model Applicability

Unknown features are fingerprint features in the query molecule, but not found in the training set.

1. All properties and OPS components are within expected ranges.

Feature Contribution

Top features for positive contribution

Fingerprint	Bit/Smiles	Feature Structure	Score	Moderate_Severe in training set
FCFP_10	907096426	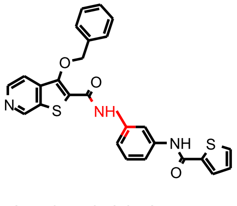 [*]NC[c](:[*]):[*]	0.332	5 out of 5

FCFP_10	547884906	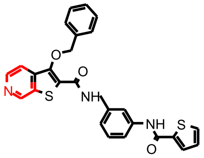 [*][c]1:[*]:[cH]:[cH]:n:[cH]:1	0.317	4 out of 4
FCFP_10	427906732	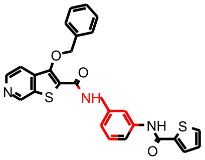 [*]NC[c]1:[cH]:[cH]:[*]:[c]([*]):[cH]:1	0.294	3 out of 3

Top Features for negative contribution

Fingerprint	Bit/Smiles	Feature Structure	Score	Moderate_Severe in training set
FCFP_10	1175232969	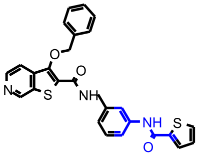 [*]:[cH]:[c](NC(=O)[c](:[*]):[*]):[cH]:[*]	-1.293	0 out of 4
FCFP_10	-1699003333	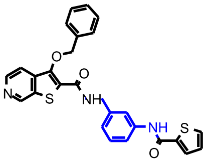 [*]C[c]1:[cH]:[cH]:[cH]:[c](N[*]):[cH]:1	-1.091	2 out of 12
FCFP_10	907036844	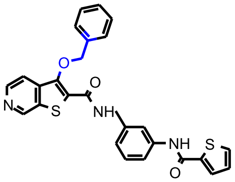 [*]OC[c](:[*]):[*]	-0.600	1 out of 4

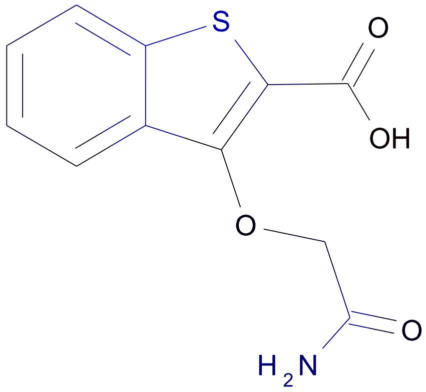

$C_{11}H_9NO_4S$
Molecular Weight: 251.25846
ALogP: 1.45
Rotatable Bonds: 4
Acceptors: 4
Donors: 2

Structural Similar Compounds

Name	NAPHTHENESULFONIC ACID;2-HYDROXY-	FURIL;DIOXIME	O-TOLUENESULFONAMIDE; N-(2-HYDROXYETHYL)-4-NITRO-
Structure	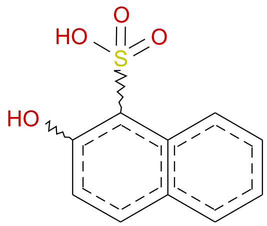	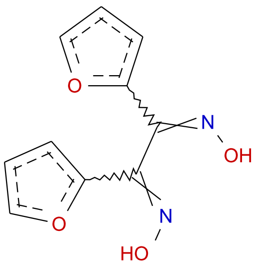	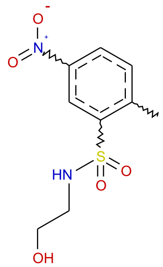
Actual Endpoint	Moderate	Moderate	Moderate
Predicted Endpoint	Moderate	Moderate	Moderate
Distance	0.581	0.597	0.603
Reference	28ZPAK-;186;72	28ZPAK	28ZPAK-;200;72

Model Prediction

Prediction: Moderate
Probability: 0.382
Enrichment: 0.616
Bayesian Score: -5.988
Mahalanobis Distance: 11.595
Mahalanobis Distance p-value: 0.00012

Prediction: Positive if the Bayesian score is above the estimated best cutoff value from minimizing the false positive and false negative rate.
Probability: The esimated probability that the sample is in the positive category. This assumes that the Bayesian score follows a normal distribution and is different from the prediction using a cutoff.
Enrichment: An estimate of enrichment, that is, the increased likelihood (versus random) of this sample being in the category.
Bayesian Score: The standard Laplacian-modified Bayesian score.
Mahalanobis Distance: The Mahalanobis distance (MD) is the distance to the center of the training data. The larger the MD, the less trustworthy the prediction.
Mahalanobis Distance p-value: The p-value gives the fraction of training data with an MD greater than or equal to the one for the given sample, assuming normally distributed data. The smaller the p-value, the less trustworthy the prediciton. For highly non-normal X properties (e.g., fingerprints), the MD p-value is wildly inaccurate.

Model Applicability

Unknown features are fingerprint features in the query molecule, but not found in the training set.

1. OPS PC11 out of range. Value: -3.9999. Training min, max, SD, explained variance: -3.5736, 4.4401, 1.244, 0.0204.

Feature Contribution

Top features for positive contribution

Fingerprint	Bit/Smiles	Feature Structure	Score	Severe in training set
SCFP_12	-424485343	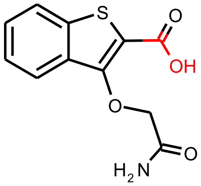 [*]C(=[*])O	0.263	43 out of 54

SCFP_12	1651620003	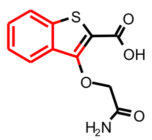 [*][c]1:[*]:[*]:[c]2:[cH]:[cH]:[cH]:[cH]:[c]:1:2	0.158	8 out of 11
SCFP_12	-1379673609	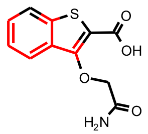 [*][c]1:[*]:[*]:[c]2:[*]:[cH]:[cH]:[cH]:[c]:1:2	0.109	13 out of 19
**Top Features for negative contribution**				
**Fingerprint**	**Bit/Smiles**	**Feature Structure**	**Score**	**Severe in training set**
SCFP_12	-1357949052	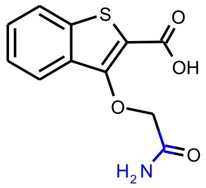 [*]C(=[*])N	-1.396	0 out of 5
SCFP_12	1310748454	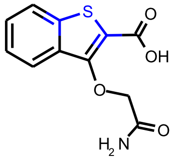 [*][c]1:[*]:[*]:[c](:[*]):s:1	-1.038	0 out of 3
SCFP_12	2083824450	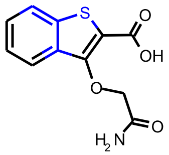 [*]:[cH]:[c]1:s:[*]:[*]:[c]:1:[*]	-1.038	0 out of 3

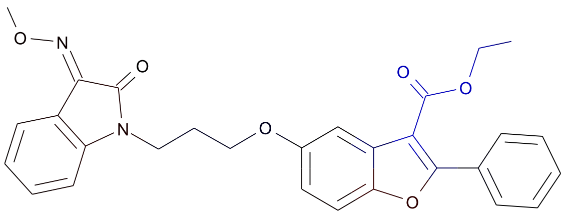

$C_{29}H_{26}N_2O_6$
Molecular Weight: 498.52653
ALogP: 4.994
Rotatable Bonds: 10
Acceptors: 6
Donors: 0

Structural Similar Compounds

Name	Benzoic acid; p-(N-butyl-2-(piperidino)acetamido)-; butyl ester;	PROPANE 2;2-BIS(3;5-DICHLORO-4-(2;3-EPOXYPROPOXY)PHENYL)-	4;5-EPOXYCYCLO HEXANE-1;2-DICARBOXYLIC ACID; DI-(2-ETHYL HEXYL)ESTER
Structure	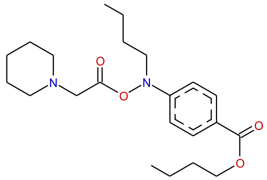	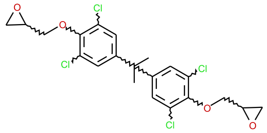	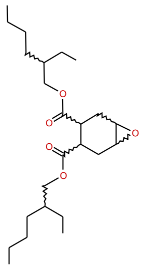
Actual Endpoint	Irritant	Irritant	Irritant
Predicted Endpoint	Non-Irritant	Irritant	Non-Irritant
Distance	0.658	0.718	0.735
Reference	Arzneimittel-Forschung 8;609;58	28ZPAK-;137;72	AIHAAP 23;95;62

Model Prediction

Prediction: Non-Irritant
Probability: 0.921
Enrichment: 1.083
Bayesian Score: -3.435
Mahalanobis Distance: 9.747
Mahalanobis Distance p-value: 0.163

Prediction: Positive if the Bayesian score is above the estimated best cutoff value from minimizing the false positive and false negative rate.
Probability: The esimated probability that the sample is in the positive category. This assumes that the Bayesian score follows a normal distribution and is different from the prediction using a cutoff.
Enrichment: An estimate of enrichment, that is, the increased likelihood (versus random) of this sample being in the category.
Bayesian Score: The standard Laplacian-modified Bayesian score.
Mahalanobis Distance: The Mahalanobis distance (MD) is the distance to the center of the training data. The larger the MD, the less trustworthy the prediction.
Mahalanobis Distance p-value: The p-value gives the fraction of training data with an MD greater than or equal to the one for the given sample, assuming normally distributed data. The smaller the p-value, the less trustworthy the prediciton. For highly non-normal X properties (e.g., fingerprints), the MD p-value is wildly inaccurate.

Model Applicability

Unknown features are fingerprint features in the query molecule, but not found in the training set.

1. All properties and OPS components are within expected ranges.

Feature Contribution

Top features for positive contribution

Fingerprint	Bit/Smiles	Feature Structure	Score	Irritant in training set
FCFP_12	1747237384	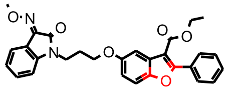 [*][c]1:[*]:[*]:[c](:[*]):o:1	0.208	44 out of 44

FCFP_12	178336375	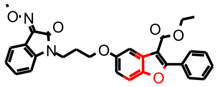 [*]:[cH]:[c]1:o:[*]:[*]:[c]:1:[*]	0.202	19 out of 19
FCFP_12	713358128	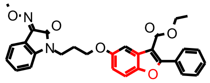 [*][c]1:[*]:[c]2:[*]: [*]:o:[c]:2:[cH]:[cH]:1	0.200	17 out of 17

Top Features for negative contribution

Fingerprint	Bit/Smiles	Feature Structure	Score	Irritant in training set
FCFP_12	-157382193	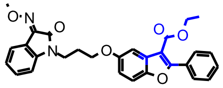 [*][c]1:[*]:[*]:[c](: [*]):[c]:1C(=O)OCC	-1.231	0 out of 3
FCFP_12	-798628285	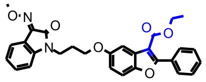 [*]:[c](:[*])C(=O)OCC	-1.096	5 out of 21
FCFP_12	391786003	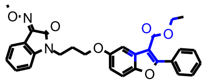 [*]COC(=O)[c]1:[c]([*]):[*]:[*]:[c]:1:[*]	-0.710	13 out of 34

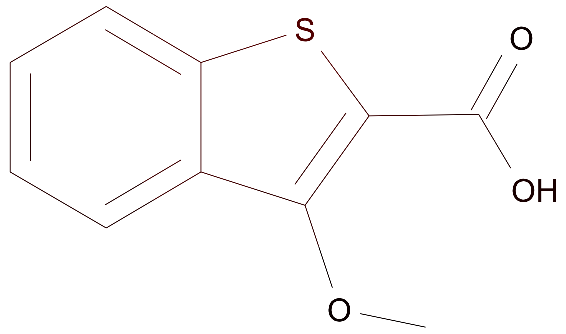

$C_{10}H_8O_3S$
Molecular Weight: 208.23372
ALogP: 2.596
Rotatable Bonds: 2
Acceptors: 3
Donors: 1

Model Prediction

Prediction: Irritant
Probability: 1.000
Enrichment: 1.176
Bayesian Score: 2.644
Mahalanobis Distance: 4.853
Mahalanobis Distance p-value: 1

Prediction: Positive if the Bayesian score is above the estimated best cutoff value from minimizing the false positive and false negative rate.
Probability: The esimated probability that the sample is in the positive category. This assumes that the Bayesian score follows a normal distribution and is different from the prediction using a cutoff.
Enrichment: An estimate of enrichment, that is, the increased likelihood (versus random) of this sample being in the category.
Bayesian Score: The standard Laplacian-modified Bayesian score.
Mahalanobis Distance: The Mahalanobis distance (MD) is the distance to the center of the training data. The larger the MD, the less trustworthy the prediction.
Mahalanobis Distance p-value: The p-value gives the fraction of training data with an MD greater than or equal to the one for the given sample, assuming normally distributed data. The smaller the p-value, the less trustworthy the prediciton. For highly non-normal X properties (e.g., fingerprints), the MD p-value is wildly inaccurate.

Structural Similar Compounds

Name	PHENOL;2;4-DICHLORO-6-NITRO	Furil; monooxime	BENZOIC ACID;2-CHLORO-4-NITRO-
Structure	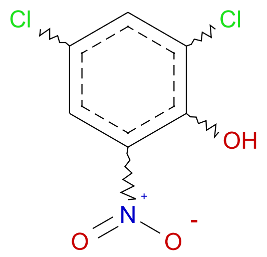	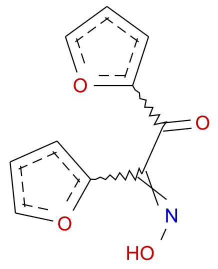	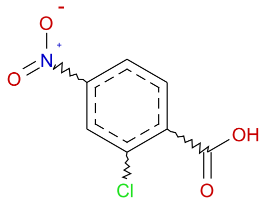
Actual Endpoint	Irritant	Irritant	Irritant
Predicted Endpoint	Irritant	Irritant	Irritant
Distance	0.481	0.487	0.576
Reference	28ZPAK-;80;72	Prehled Prumyslove Toxikologie; Organicke Latky; Marhold; J. -;793;86	28ZPAK-;91;72

Model Applicability

Unknown features are fingerprint features in the query molecule, but not found in the training set.

1. All properties and OPS components are within expected ranges.

Feature Contribution

Top features for positive contribution

Fingerprint	Bit/Smiles	Feature Structure	Score	Irritant in training set
FCFP_12	1747237384	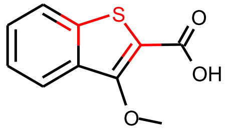 [*][c]1:[*]:[*]:[c](:[*]):o:1	0.208	44 out of 44

FCFP_12	178336375	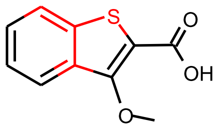<br>[*]:[cH]:[c]1:o:[*]:[*]:[c]:1:[*]	0.202	19 out of 19
FCFP_12	713358128	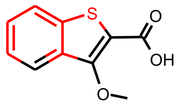<br>[*][c]1:[*]:[c]2:[*]: [*]:o:[c]:2:[cH]:[cH]:1	0.200	17 out of 17
**Top Features for negative contribution**				
**Fingerprint**	**Bit/Smiles**	**Feature Structure**	**Score**	**Irritant in training set**
FCFP_12	0	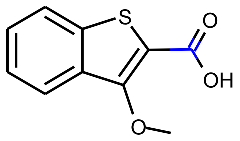<br>[*]C(=[*])[*]	0.000	1184 out of 1397
FCFP_12	1	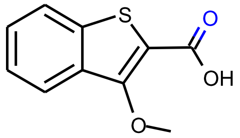<br>[*]=O	0.000	872 out of 1051
FCFP_12	307419094	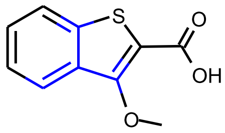<br>[*][c]1:[*]:[*]:[c](: [*]):[c]:1:[cH]:[*]	0.000	43 out of 52

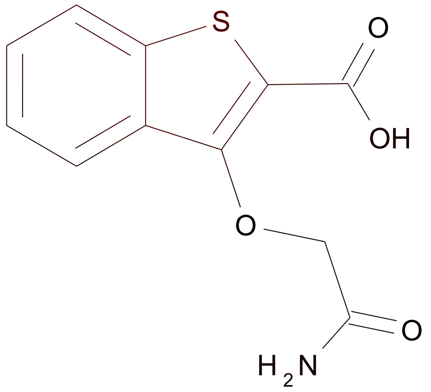

$C_{11}H_9NO_4S$
Molecular Weight: 251.25846
ALogP: 1.45
Rotatable Bonds: 4
Acceptors: 4
Donors: 2

Model Prediction

Prediction: Irritant
Probability: 1.000
Enrichment: 1.176
Bayesian Score: 2.841
Mahalanobis Distance: 6.089
Mahalanobis Distance p-value: 1

Prediction: Positive if the Bayesian score is above the estimated best cutoff value from minimizing the false positive and false negative rate.
Probability: The esimated probability that the sample is in the positive category. This assumes that the Bayesian score follows a normal distribution and is different from the prediction using a cutoff.
Enrichment: An estimate of enrichment, that is, the increased likelihood (versus random) of this sample being in the category.
Bayesian Score: The standard Laplacian-modified Bayesian score.
Mahalanobis Distance: The Mahalanobis distance (MD) is the distance to the center of the training data. The larger the MD, the less trustworthy the prediction.
Mahalanobis Distance p-value: The p-value gives the fraction of training data with an MD greater than or equal to the one for the given sample, assuming normally distributed data. The smaller the p-value, the less trustworthy the prediciton. For highly non-normal X properties (e.g., fingerprints), the MD p-value is wildly inaccurate.

Structural Similar Compounds

Name	1-NAPHTHALENESULFONIC ACID; 2-AMINO-	2-NAPHTHALENE SULFONIC ACID;5-AMINO-	NAPHTHENESULFONIC ACID;2-HYDROXY-
Structure	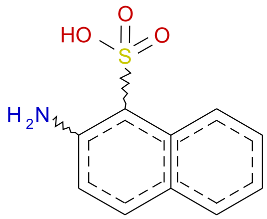	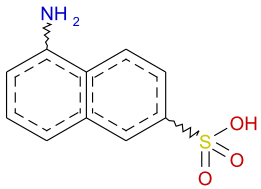	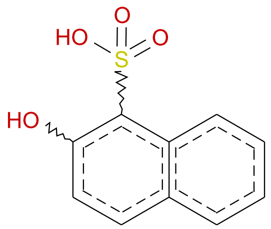
Actual Endpoint	Irritant	Irritant	Irritant
Predicted Endpoint	Irritant	Irritant	Irritant
Distance	0.537	0.549	0.551
Reference	28ZPAK-;187;72	28ZPAK-;187;72	28ZPAK-;186;72

Model Applicability

Unknown features are fingerprint features in the query molecule, but not found in the training set.

1. All properties and OPS components are within expected ranges.

Feature Contribution

Top features for positive contribution

Fingerprint	Bit/Smiles	Feature Structure	Score	Irritant in training set
FCFP_12	1747237384	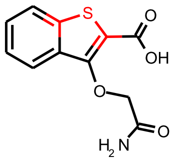 [*][c]1:[*]:[*]:[c](:[*]):o:1	0.208	44 out of 44

FCFP_12	178336375	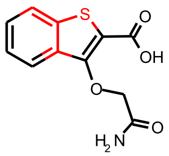 [*]:[cH]:[c]1:o:[*]:[*]:[c]:1:[*]	0.202	19 out of 19
FCFP_12	713358128	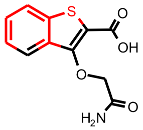 [*][c]1:[*]:[c]2:[*]: [*]:o:[c]:2:[cH]:[cH]:1	0.200	17 out of 17

Top Features for negative contribution

**Fingerprint**	**Bit/Smiles**	**Feature Structure**	**Score**	**Irritant in training set**
FCFP_12	307419094	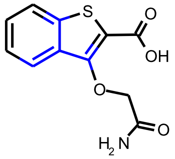 [*][c]1:[*]:[*]:[c](: [*]):[c]:1:[cH]:[*]	0.000	43 out of 52
FCFP_12	-1272768868	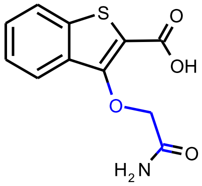 [*]CCO[*]	0.000	396 out of 514
FCFP_12	1872154524	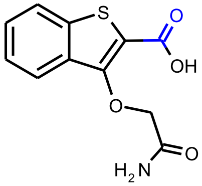 [*]C(=O)[*]	0.000	563 out of 690

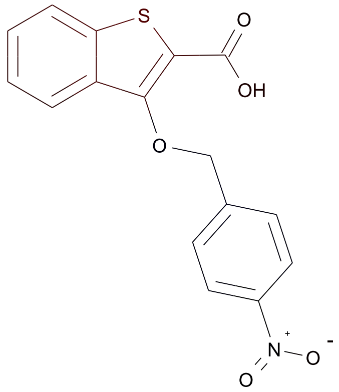

$C_{16}H_{11}NO_5S$
Molecular Weight: 329.32724
ALogP: 4.074
Rotatable Bonds: 5
Acceptors: 5
Donors: 1

Structural Similar Compounds

Name	BENZOIC ACID; 5-(CHLOROSULFONYL)-2;4-DICHLORO-	PHENOL;2-SEC-BUTYL-4;6-DINITRO-	2;5-DICHLORO-4(3'-METHYL-5' PYRAZOLON-1'-YL)BENZENE SULFONIC ACID
Structure	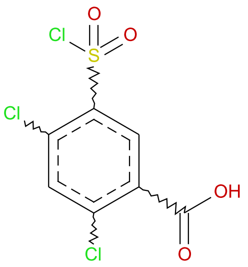	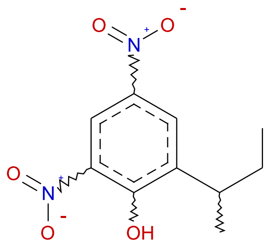	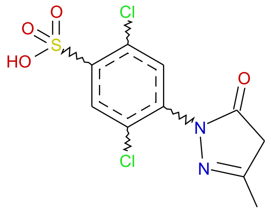
Actual Endpoint	Irritant	Irritant	Irritant
Predicted Endpoint	Irritant	Irritant	Irritant
Distance	0.644	0.652	0.695
Reference	FCTOD7 20;573;82	28ZPAK-;108;72	28ZPAK-;186;72

Model Prediction

Prediction: Irritant
Probability: 1.000
Enrichment: 1.176
Bayesian Score: 1.946
Mahalanobis Distance: 3.253
Mahalanobis Distance p-value: 1

Prediction: Positive if the Bayesian score is above the estimated best cutoff value from minimizing the false positive and false negative rate.
Probability: The esimated probability that the sample is in the positive category. This assumes that the Bayesian score follows a normal distribution and is different from the prediction using a cutoff.
Enrichment: An estimate of enrichment, that is, the increased likelihood (versus random) of this sample being in the category.
Bayesian Score: The standard Laplacian-modified Bayesian score.
Mahalanobis Distance: The Mahalanobis distance (MD) is the distance to the center of the training data. The larger the MD, the less trustworthy the prediction.
Mahalanobis Distance p-value: The p-value gives the fraction of training data with an MD greater than or equal to the one for the given sample, assuming normally distributed data. The smaller the p-value, the less trustworthy the prediciton. For highly non-normal X properties (e.g., fingerprints), the MD p-value is wildly inaccurate.

Model Applicability

Unknown features are fingerprint features in the query molecule, but not found in the training set.

1. All properties and OPS components are within expected ranges.
2. Unknown FCFP_2 feature: -828984032: [*][N+](=[*])[c](:[cH]:[*]):[cH]:[*]
3. Unknown FCFP_2 feature: -1338588315: [*]:[c](:[*])[N+](=O)[O-]
4. Unknown FCFP_2 feature: 1872392852: [*][N+](=O)[*]
5. Unknown FCFP_2 feature: 260476081: [*][N+](=[*])[O-]

Feature Contribution

Top features for positive contribution				
**Fingerprint**	**Bit/Smiles**	**Feature Structure**	**Score**	**Irritant in training set**
FCFP_12	1747237384	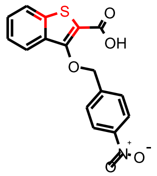 [*][c]1:[*]:[*]:[c](:[*]):o:1	0.208	44 out of 44

FCFP_12	178336375	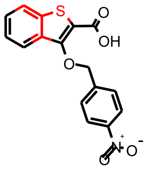<br>[*]:[cH]:[c]1:o:[*]:[*]:[c]:1:[*]	0.202	19 out of 19
FCFP_12	713358128	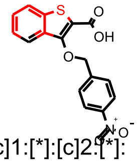<br>[*][c]1:[*]:[c]2:[*]:[*]:o:[c]:2:[cH]:[cH]:1	0.200	17 out of 17
**Top Features for negative contribution**				
**Fingerprint**	**Bit/Smiles**	**Feature Structure**	**Score**	**Irritant in training set**
FCFP_12	2106393770	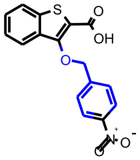<br>[*]OC[c]1:[cH]:[cH]:[*]:[cH]:[cH]:1	-0.231	3 out of 5
FCFP_12	907036844	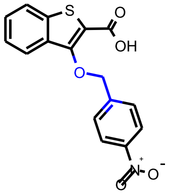<br>[*]OC[c](:[*]):[*]	-0.156	4 out of 6
FCFP_12	8	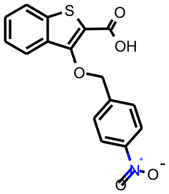<br>[*][N+](=[*])[*]	-0.056	3 out of 4

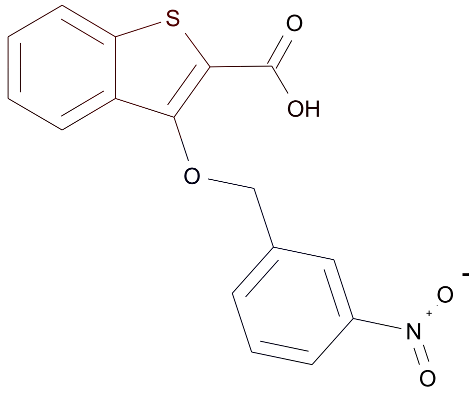

$C_{16}H_{11}NO_5S$
Molecular Weight: 329.32724
ALogP: 4.074
Rotatable Bonds: 5
Acceptors: 5
Donors: 1

Model Prediction

Prediction: Irritant
Probability: 1.000
Enrichment: 1.176
Bayesian Score: 1.715
Mahalanobis Distance: 3.253
Mahalanobis Distance p-value: 1

Prediction: Positive if the Bayesian score is above the estimated best cutoff value from minimizing the false positive and false negative rate.
Probability: The esimated probability that the sample is in the positive category. This assumes that the Bayesian score follows a normal distribution and is different from the prediction using a cutoff.
Enrichment: An estimate of enrichment, that is, the increased likelihood (versus random) of this sample being in the category.
Bayesian Score: The standard Laplacian-modified Bayesian score.
Mahalanobis Distance: The Mahalanobis distance (MD) is the distance to the center of the training data. The larger the MD, the less trustworthy the prediction.
Mahalanobis Distance p-value: The p-value gives the fraction of training data with an MD greater than or equal to the one for the given sample, assuming normally distributed data. The smaller the p-value, the less trustworthy the prediciton. For highly non-normal X properties (e.g., fingerprints), the MD p-value is wildly inaccurate.

Structural Similar Compounds

Name	BENZOIC ACID; 5-(CHLOROSULFONYL)-2;4-DICHLORO-	PHENOL;2-SEC-BUTYL-4;6-DINITRO-	2;5-DICHLORO-4(3'-METHYL-5' PYRAZOLON-1'-YL)BENZENE SULFONIC ACID
Structure	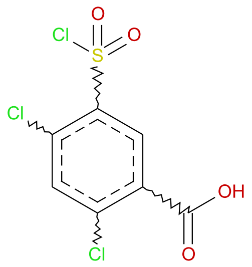	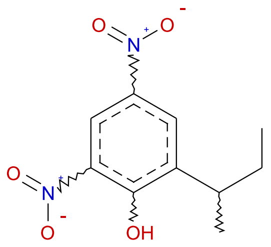	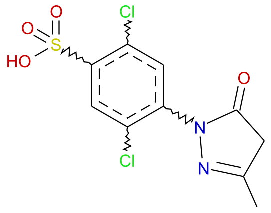
Actual Endpoint	Irritant	Irritant	Irritant
Predicted Endpoint	Irritant	Irritant	Irritant
Distance	0.643	0.652	0.695
Reference	FCTOD7 20;573;82	28ZPAK-;108;72	28ZPAK-;186;72

Model Applicability

Unknown features are fingerprint features in the query molecule, but not found in the training set.

1. All properties and OPS components are within expected ranges.
2. Unknown FCFP_2 feature: -828984032: [*][N+](=[*])[c](:[cH]:[*]):[cH]:[*]
3. Unknown FCFP_2 feature: -1338588315: [*]:[c](:[*])[N+](=O)[O-]
4. Unknown FCFP_2 feature: 1872392852: [*][N+](=O)[*]
5. Unknown FCFP_2 feature: 260476081: [*][N+](=[*])[O-]

Feature Contribution

Top features for positive contribution

Fingerprint	Bit/Smiles	Feature Structure	Score	Irritant in training set
FCFP_12	1747237384	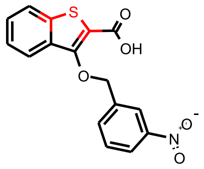 [*][c]1:[*]:[*]:[c](:[*]):o:1	0.208	44 out of 44

FCFP_12	178336375	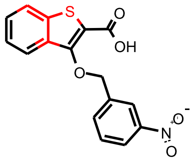 [*]:[cH]:[c]1:o:[*]:[*]:[c]:1:[*]	0.202	19 out of 19
FCFP_12	713358128	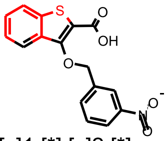 [*][c]1:[*]:[c]2:[*]:[*]:o:[c]:2:[cH]:[cH]:1	0.200	17 out of 17
**Top Features for negative contribution**				
**Fingerprint**	**Bit/Smiles**	**Feature Structure**	**Score**	**Irritant in training set**
FCFP_12	2106393770	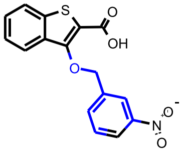 [*]OC[c]1:[cH]:[cH]:[*]:[cH]:[cH]:1	-0.231	3 out of 5
FCFP_12	1390842262	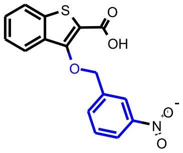 [*]OC[c]1:[cH]:[cH]:[cH]:[c]([*]):[cH]:1	-0.231	3 out of 5
FCFP_12	907036844	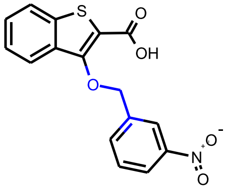 [*]OC[c](:[*]):[*]	-0.156	4 out of 6

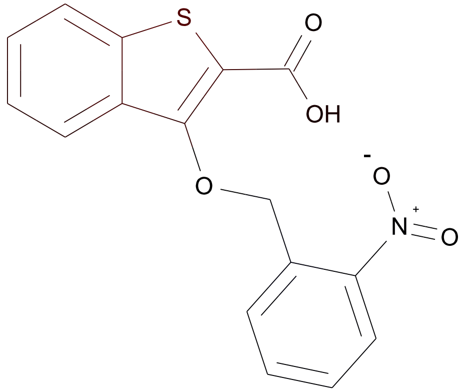

$C_{16}H_{11}NO_5S$

Molecular Weight: 329.32724

ALogP: 4.074

Rotatable Bonds: 5

Acceptors: 5

Donors: 1

Structural Similar Compounds

Name	BENZOIC ACID; 5-(CHLOROSULFONYL)-2;4-DICHLORO-	PHENOL;2-SEC-BUTYL-4;6-DINITRO-	2;5-DICHLORO-4(3'-METHYL-5' PYRAZOLON-1'-YL)BENZENE SULFONIC ACID
Structure	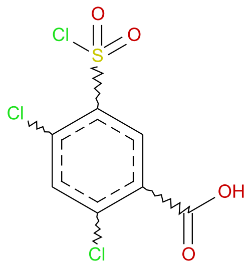	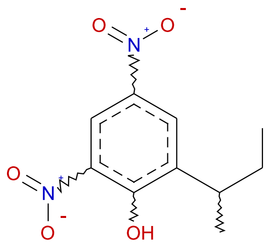	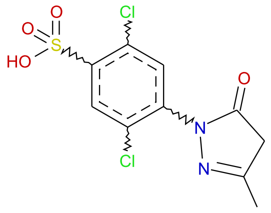
Actual Endpoint	Irritant	Irritant	Irritant
Predicted Endpoint	Irritant	Irritant	Irritant
Distance	0.641	0.650	0.693
Reference	FCTOD7 20;573;82	28ZPAK-;108;72	28ZPAK-;186;72

Model Prediction

Prediction: Irritant

Probability: 1.000

Enrichment: 1.176

Bayesian Score: 2.081

Mahalanobis Distance: 3.253

Mahalanobis Distance p-value: 1

Prediction: Positive if the Bayesian score is above the estimated best cutoff value from minimizing the false positive and false negative rate.
Probability: The esimated probability that the sample is in the positive category. This assumes that the Bayesian score follows a normal distribution and is different from the prediction using a cutoff.
Enrichment: An estimate of enrichment, that is, the increased likelihood (versus random) of this sample being in the category.
Bayesian Score: The standard Laplacian-modified Bayesian score.
Mahalanobis Distance: The Mahalanobis distance (MD) is the distance to the center of the training data. The larger the MD, the less trustworthy the prediction.
Mahalanobis Distance p-value: The p-value gives the fraction of training data with an MD greater than or equal to the one for the given sample, assuming normally distributed data. The smaller the p-value, the less trustworthy the prediciton. For highly non-normal X properties (e.g., fingerprints), the MD p-value is wildly inaccurate.

Model Applicability

Unknown features are fingerprint features in the query molecule, but not found in the training set.

1. All properties and OPS components are within expected ranges.
2. Unknown FCFP_2 feature: -828984032: [*][N+](=[*])[c](:[cH]:[*]):[cH]:[*]
3. Unknown FCFP_2 feature: -1338588315: [*]:[c](:[*])[N+](=O)[O-]
4. Unknown FCFP_2 feature: 1872392852: [*][N+](=O)[*]
5. Unknown FCFP_2 feature: 260476081: [*][N+](=[*])[O-]

Feature Contribution

Top features for positive contribution				
Fingerprint	Bit/Smiles	Feature Structure	Score	Irritant in training set
FCFP_12	1747237384	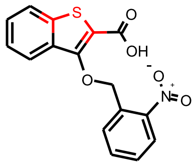 [*][c]1:[*]:[*]:[c](:[*]):o:1	0.208	44 out of 44

FCFP_12	178336375	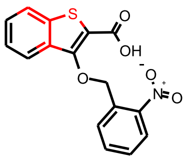 [*]:[cH]:[c]1:o:[*]:[*]:[c]:1:[*]	0.202	19 out of 19
FCFP_12	713358128	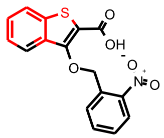 [*][c]1:[*]:[c]2:[*]:[*]:o:[c]:2:[cH]:[cH]:1	0.200	17 out of 17

Top Features for negative contribution

Fingerprint	Bit/Smiles	Feature Structure	Score	Irritant in training set
FCFP_12	907036844	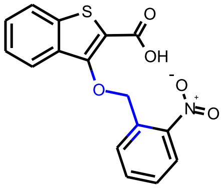 [*]OC[c](:[*]):[*]	-0.156	4 out of 6
FCFP_12	-1698724694	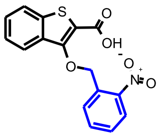 [*]=C1[*][*][c]2:[cH]:[cH]:[cH]:[cH]:[c]1:2	-0.096	107 out of 146
FCFP_12	8	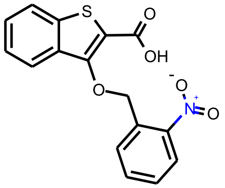 [*][N+](=[*])[*]	-0.056	3 out of 4

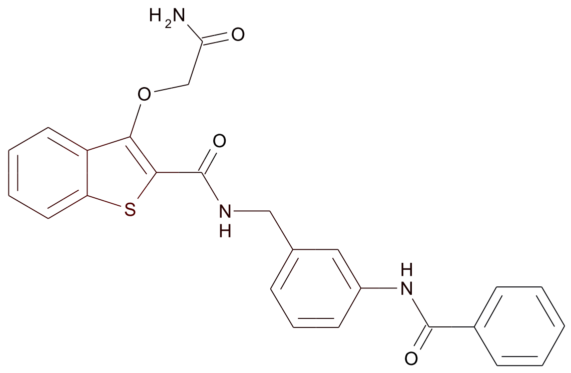

$C_{25}H_{21}N_3O_4S$
Molecular Weight: 459.51694
ALogP: 3.396
Rotatable Bonds: 8
Acceptors: 4
Donors: 3

Model Prediction

Prediction: Irritant
Probability: 1.000
Enrichment: 1.176
Bayesian Score: 2.995
Mahalanobis Distance: 8.048
Mahalanobis Distance p-value: 0.914

Prediction: Positive if the Bayesian score is above the estimated best cutoff value from minimizing the false positive and false negative rate.
Probability: The esimated probability that the sample is in the positive category. This assumes that the Bayesian score follows a normal distribution and is different from the prediction using a cutoff.
Enrichment: An estimate of enrichment, that is, the increased likelihood (versus random) of this sample being in the category.
Bayesian Score: The standard Laplacian-modified Bayesian score.
Mahalanobis Distance: The Mahalanobis distance (MD) is the distance to the center of the training data. The larger the MD, the less trustworthy the prediction.
Mahalanobis Distance p-value: The p-value gives the fraction of training data with an MD greater than or equal to the one for the given sample, assuming normally distributed data. The smaller the p-value, the less trustworthy the prediciton. For highly non-normal X properties (e.g., fingerprints), the MD p-value is wildly inaccurate.

Structural Similar Compounds

Name	BENZANILIDE;2';2'''-DITHIOBIS-	1-AMINO-4-BENZOYLAMINO-ANTHRAQUINONE	4;4'-DIAMINO-1;1'-DIANTHRIMIDE
Structure	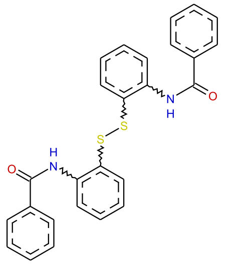	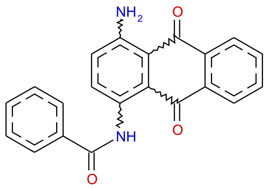	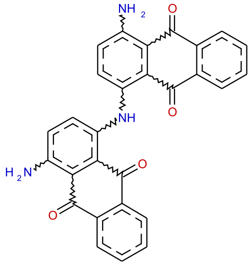
Actual Endpoint	Non-Irritant	Irritant	Irritant
Predicted Endpoint	Non-Irritant	Irritant	Irritant
Distance	0.757	0.766	0.771
Reference	28ZPAK-;173;72	28ZPAK-;124;72	28ZPAK-;125;72

Model Applicability

Unknown features are fingerprint features in the query molecule, but not found in the training set.

1. All properties and OPS components are within expected ranges.

Feature Contribution

Top features for positive contribution				
Fingerprint	Bit/Smiles	Feature Structure	Score	Irritant in training set
FCFP_12	1747237384	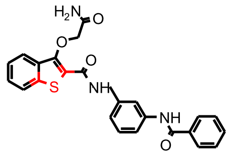 [*][c]1:[*]:[*]:[c](:[*]):o:1	0.208	44 out of 44

FCFP_12	178336375	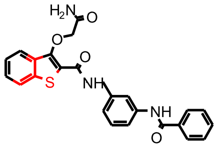<br>[*]:[cH]:[c]1:o:[*]:[<br>*]:[c]:1:[*]	0.202	19 out of 19
FCFP_12	713358128	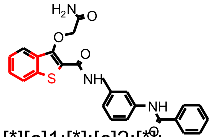<br>[*][c]1:[*]:[c]2:[*]:<br>[*]:o:[c]:2:[cH]:[cH<br>]:1	0.200	17 out of 17
**Top Features for negative contribution**				
**Fingerprint**	**Bit/Smiles**	**Feature Structure**	**Score**	**Irritant in training set**
FCFP_12	-1698724694	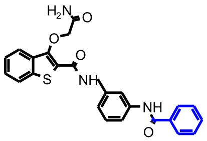<br>[*]=C1[*][*][c]2:[cH]<br>:[cH]:[cH]:[cH]:[c]1<br>:2	-0.096	107 out of 146
FCFP_12	-581879738	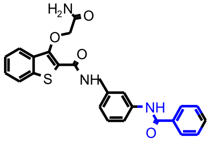<br>[*]NC(=O)[c]1:[cH]:[c<br>H]:[*]:[cH]:[cH]:1	0.000	4 out of 5
FCFP_12	1175232969	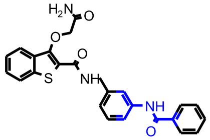<br>[*]:[cH]:[c](NC(=O)[c<br>](:[*]):[*]):[cH]:[*<br>]	0.000	4 out of 5

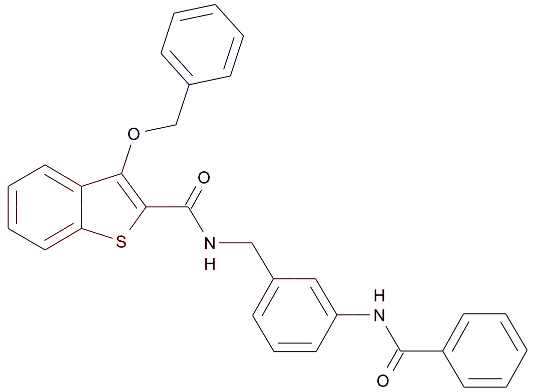

$C_{30}H_{24}N_2O_3S$
Molecular Weight: 492.58816
ALogP: 6.126
Rotatable Bonds: 8
Acceptors: 3
Donors: 2

Model Prediction

Prediction: Irritant
Probability: 1.000
Enrichment: 1.176
Bayesian Score: 1.807
Mahalanobis Distance: 6.356
Mahalanobis Distance p-value: 1

Prediction: Positive if the Bayesian score is above the estimated best cutoff value from minimizing the false positive and false negative rate.
Probability: The esimated probability that the sample is in the positive category. This assumes that the Bayesian score follows a normal distribution and is different from the prediction using a cutoff.
Enrichment: An estimate of enrichment, that is, the increased likelihood (versus random) of this sample being in the category.
Bayesian Score: The standard Laplacian-modified Bayesian score.
Mahalanobis Distance: The Mahalanobis distance (MD) is the distance to the center of the training data. The larger the MD, the less trustworthy the prediction.
Mahalanobis Distance p-value: The p-value gives the fraction of training data with an MD greater than or equal to the one for the given sample, assuming normally distributed data. The smaller the p-value, the less trustworthy the prediciton. For highly non-normal X properties (e.g., fingerprints), the MD p-value is wildly inaccurate.

Structural Similar Compounds

Name	BENZANILIDE;2';2'''-DITHIOBIS-	ANTHRAQUINONE; 1;4-BIS(p-TOLYLAMINO)-	DINAPHTHO(1;2;3-CD:3';2';1'-IM)PERYLENE-5;10-DIONE;16;17-DIHYDROXY
Structure	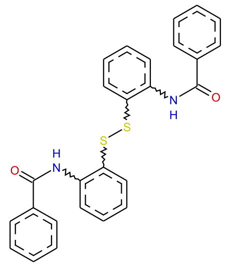	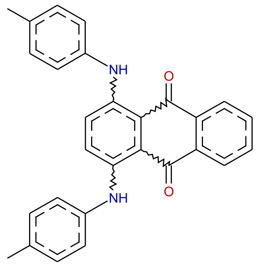	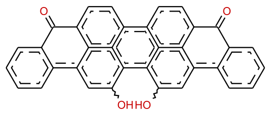
Actual Endpoint	Non-Irritant	Irritant	Irritant
Predicted Endpoint	Non-Irritant	Non-Irritant	Irritant
Distance	0.546	0.671	0.699
Reference	28ZPAK-;173;72	28ZPAK -;124;72	28ZPAK-;104;72

Model Applicability

Unknown features are fingerprint features in the query molecule, but not found in the training set.

1. All properties and OPS components are within expected ranges.

Feature Contribution

Top features for positive contribution

Fingerprint	Bit/Smiles	Feature Structure	Score	Irritant in training set
FCFP_12	1747237384	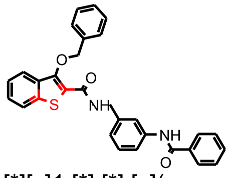 [*][c]1:[*]:[*]:[c](:[*]):o:1	0.208	44 out of 44

FCFP_12	178336375	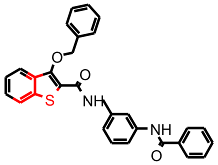<br>[*]:[cH]:[c]1:o:[*]:[<br>*]:[c]:1:[*]	0.202	19 out of 19
FCFP_12	713358128	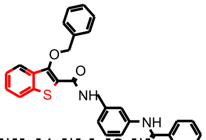<br>[*][c]1:[*]:[c]2:[*]:o:<br>[*]:o:[c]:2:[cH]:[cH<br>]:1	0.200	17 out of 17
**Top Features for negative contribution**				
**Fingerprint**	**Bit/Smiles**	**Feature Structure**	**Score**	**Irritant in training set**
FCFP_12	2106393770	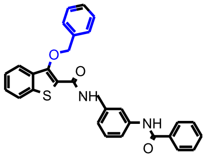<br>[*]OC[c]1:[cH]:[cH]:[<br>*]:[cH]:[cH]:1	-0.231	3 out of 5
FCFP_12	1390842262	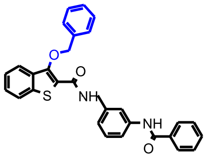<br>[*]OC[c]1:[cH]:[cH]:[<br>cH]:[c]([*]):[cH]:1	-0.231	3 out of 5
FCFP_12	907036844	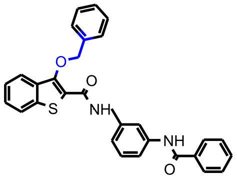<br>[*]OC[c](:[*]):[*]	-0.156	4 out of 6

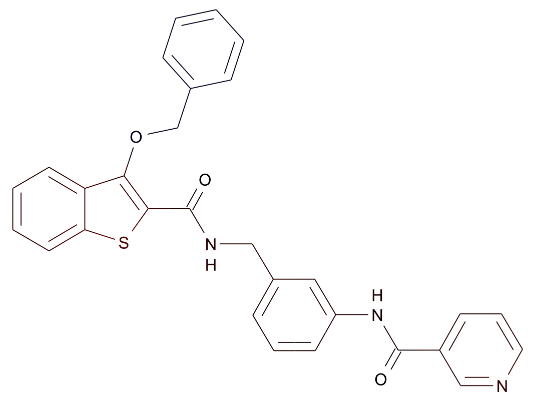

$C_{29}H_{23}N_3O_3S$
Molecular Weight: 493.57622
ALogP: 4.976
Rotatable Bonds: 8
Acceptors: 4
Donors: 2

Structural Similar Compounds

Name	BENZANILIDE;2';2'''-DITHIOBIS-	ANTHRAQUINONE; 1;4-BIS(p-TOLYLAMINO)-	DINAPHTHO(1;2;3-CD:3';2';1'-IM)PERYLENE-5;10-DIONE;16;17-DIHYDROXY
Structure	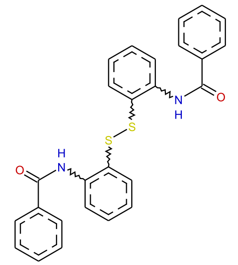	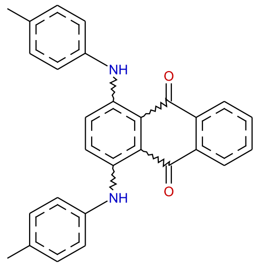	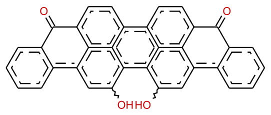
Actual Endpoint	Non-Irritant	Irritant	Irritant
Predicted Endpoint	Non-Irritant	Non-Irritant	Irritant
Distance	0.565	0.723	0.728
Reference	28ZPAK-;173;72	28ZPAK -;124;72	28ZPAK-;104;72

Model Prediction

Prediction: Irritant
Probability: 1.000
Enrichment: 1.176
Bayesian Score: 2.601
Mahalanobis Distance: 5.838
Mahalanobis Distance p-value: 1

Prediction: Positive if the Bayesian score is above the estimated best cutoff value from minimizing the false positive and false negative rate.
Probability: The esimated probability that the sample is in the positive category. This assumes that the Bayesian score follows a normal distribution and is different from the prediction using a cutoff.
Enrichment: An estimate of enrichment, that is, the increased likelihood (versus random) of this sample being in the category.
Bayesian Score: The standard Laplacian-modified Bayesian score.
Mahalanobis Distance: The Mahalanobis distance (MD) is the distance to the center of the training data. The larger the MD, the less trustworthy the prediction.
Mahalanobis Distance p-value: The p-value gives the fraction of training data with an MD greater than or equal to the one for the given sample, assuming normally distributed data. The smaller the p-value, the less trustworthy the prediciton. For highly non-normal X properties (e.g., fingerprints), the MD p-value is wildly inaccurate.

Model Applicability

Unknown features are fingerprint features in the query molecule, but not found in the training set.

1. All properties and OPS components are within expected ranges.

Feature Contribution

Top features for positive contribution

Fingerprint	Bit/Smiles	Feature Structure	Score	Irritant in training set
FCFP_12	1747237384	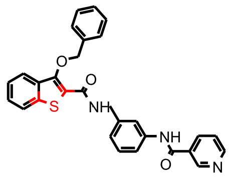 [*][c]1:[*]:[*]:[c](:[*]):o:1	0.208	44 out of 44

FCFP_12	178336375	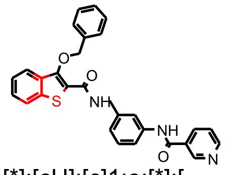 [*]:[cH]:[c]1:o:[*]:[*]:[c]:1:[*]	0.202	19 out of 19
FCFP_12	713358128	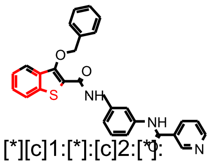 [*][c]1:[*]:[c]2:[*]: [*]:o:[c]:2:[cH]:[cH]:1	0.200	17 out of 17

Top Features for negative contribution

Fingerprint	Bit/Smiles	Feature Structure	Score	Irritant in training set
FCFP_12	1390842262	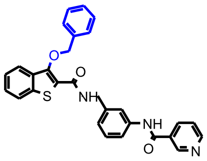 [*]OC[c]1:[cH]:[cH]:[cH]:[c]([*]):[cH]:1	-0.231	3 out of 5
FCFP_12	2106393770	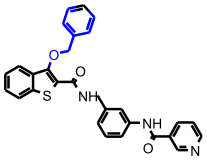 [*]OC[c]1:[cH]:[cH]:[*]:[cH]:[cH]:1	-0.231	3 out of 5
FCFP_12	907036844	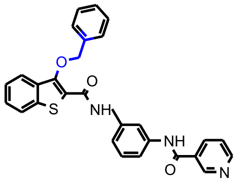 [*]OC[c](:[*]):[*]	-0.156	4 out of 6

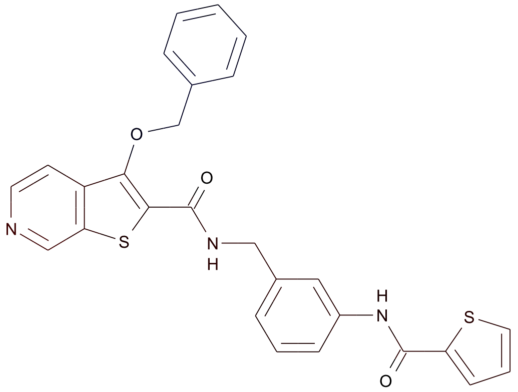

$C_{27}H_{21}N_3O_3S_2$
Molecular Weight: 499.60394
ALogP: 4.929
Rotatable Bonds: 8
Acceptors: 4
Donors: 2

Structural Similar Compounds

Name	BENZANILIDE;2';2'''-DITHIOBIS-	DINAPHTHO(1;2;3-CD:3';2';1'-IM)PERYLENE-5;10-DIONE;16;17-DIHYDROXY	ANTHRAQUINONE; 1;4-BIS(p-TOLYLAMINO)-
Structure	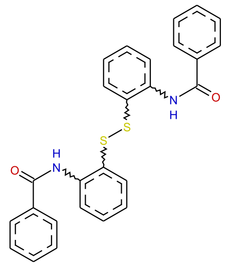	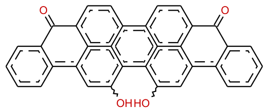	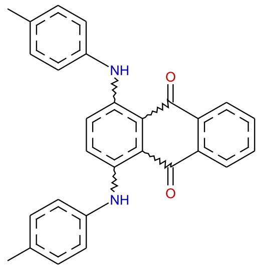
Actual Endpoint	Non-Irritant	Irritant	Irritant
Predicted Endpoint	Non-Irritant	Irritant	Non-Irritant
Distance	0.583	0.765	0.776
Reference	28ZPAK-;173;72	28ZPAK-;104;72	28ZPAK -;124;72

Model Prediction

Prediction: Irritant
Probability: 1.000
Enrichment: 1.176
Bayesian Score: 2.679
Mahalanobis Distance: 6.204
Mahalanobis Distance p-value: 1

Prediction: Positive if the Bayesian score is above the estimated best cutoff value from minimizing the false positive and false negative rate.
Probability: The esimated probability that the sample is in the positive category. This assumes that the Bayesian score follows a normal distribution and is different from the prediction using a cutoff.
Enrichment: An estimate of enrichment, that is, the increased likelihood (versus random) of this sample being in the category.
Bayesian Score: The standard Laplacian-modified Bayesian score.
Mahalanobis Distance: The Mahalanobis distance (MD) is the distance to the center of the training data. The larger the MD, the less trustworthy the prediction.
Mahalanobis Distance p-value: The p-value gives the fraction of training data with an MD greater than or equal to the one for the given sample, assuming normally distributed data. The smaller the p-value, the less trustworthy the prediciton. For highly non-normal X properties (e.g., fingerprints), the MD p-value is wildly inaccurate.

Model Applicability

Unknown features are fingerprint features in the query molecule, but not found in the training set.

1. All properties and OPS components are within expected ranges.

Feature Contribution

Top features for positive contribution				
Fingerprint	Bit/Smiles	Feature Structure	Score	Irritant in training set
FCFP_12	1747237384	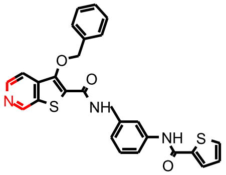 [*][c]1:[*]:[*]:[c](:[*]):o:1	0.208	44 out of 44

FCFP_12	178336375	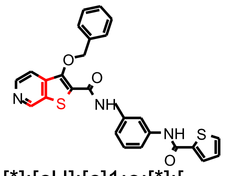 [*]:[cH]:[c]1:o:[*]:[*]:[c]:1:[*]	0.202	19 out of 19
FCFP_12	-124655670	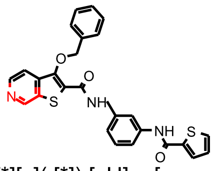 [*][c](:[*]):[cH]:n:[*]	0.200	16 out of 16
**Top Features for negative contribution**				
**Fingerprint**	**Bit/Smiles**	**Feature Structure**	**Score**	**Irritant in training set**
FCFP_12	2106393770	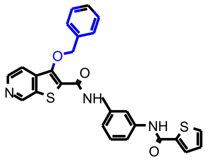 [*]OC[c]1:[cH]:[cH]:[*]:[cH]:[cH]:1	-0.231	3 out of 5
FCFP_12	1390842262	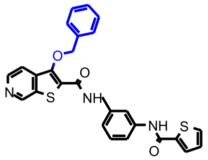 [*]OC[c]1:[cH]:[cH]:[cH]:[c]([*]):[cH]:1	-0.231	3 out of 5
FCFP_12	907036844	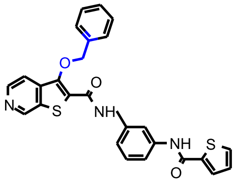 [*]OC[c](:[*]):[*]	-0.156	4 out of 6

Co-crystallized ligand

TOPKAT_Ocular_Irritancy_None_vs_Irritant

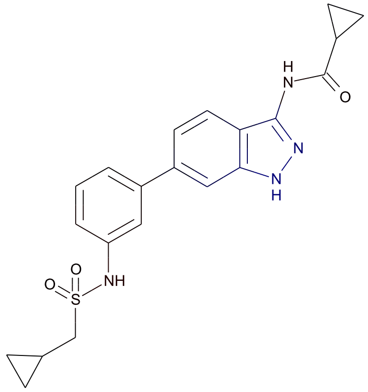

$C_{21}H_{22}N_4O_3S$
Molecular Weight: 410.48938
ALogP: 3.14
Rotatable Bonds: 7
Acceptors: 4
Donors: 3

Structural Similar Compounds

Name	ANTHRAQUINONE; 1-((2-HYDROXYETHYL)AMINO)-4-(METHYLAMINO)-	METHANE;TRIS(4-AMINOPHENYL)-	Disperse Black 9
Structure	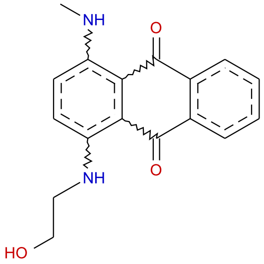	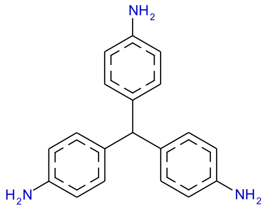	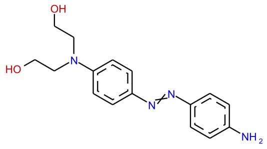
Actual Endpoint	Irritant	Irritant	Non-Irritant
Predicted Endpoint	Irritant	Irritant	Non-Irritant
Distance	0.718	0.726	0.737
Reference	28ZPAK 245;72	28ZPAK-;73;72	J. Am. Coll. Toxicol. 5(3):205;1986

Model Prediction

Prediction: Non-Irritant
Probability: 0.963
Enrichment: 1.133
Bayesian Score: -1.806
Mahalanobis Distance: 8.133
Mahalanobis Distance p-value: 0.894

Prediction: Positive if the Bayesian score is above the estimated best cutoff value from minimizing the false positive and false negative rate.
Probability: The esimated probability that the sample is in the positive category. This assumes that the Bayesian score follows a normal distribution and is different from the prediction using a cutoff.
Enrichment: An estimate of enrichment, that is, the increased likelihood (versus random) of this sample being in the category.
Bayesian Score: The standard Laplacian-modified Bayesian score.
Mahalanobis Distance: The Mahalanobis distance (MD) is the distance to the center of the training data. The larger the MD, the less trustworthy the prediction.
Mahalanobis Distance p-value: The p-value gives the fraction of training data with an MD greater than or equal to the one for the given sample, assuming normally distributed data. The smaller the p-value, the less trustworthy the prediciton. For highly non-normal X properties (e.g., fingerprints), the MD p-value is wildly inaccurate.

Model Applicability

Unknown features are fingerprint features in the query molecule, but not found in the training set.

1. All properties and OPS components are within expected ranges.
2. Unknown FCFP_2 feature: -1151884458: [*]N[c]1:n:[*]:[*]:[c]:1:[*]

Feature Contribution

Top features for positive contribution				
**Fingerprint**	**Bit/Smiles**	**Feature Structure**	**Score**	**Irritant in training set**
FCFP_12	17	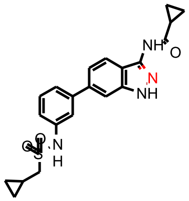 [*]:o:[*]	0.189	48 out of 49

FCFP_12	566058135	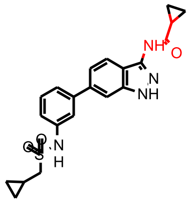 [*]CC(=O)N	0.163	23 out of 24
FCFP_12	-616878326	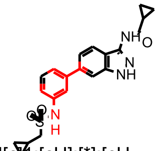 [*]N[c]1:[cH]:[*]:[cH]:[c](:[cH]:1)[c](:[*]):[*]	0.156	3 out of 3

Top Features for negative contribution

Fingerprint	Bit/Smiles	Feature Structure	Score	Irritant in training set
FCFP_12	450853127	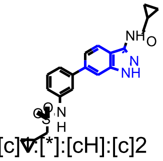 [*][c]1:[*]:[cH]:[c]2:[c]([*]):n:[nH]:[c]:2:[cH]:1	-0.592	0 out of 1
FCFP_12	-1369564483	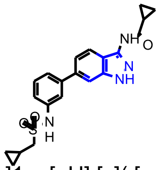 [*][c]1:n:[nH]:[c](:[cH]:[*]):[c]:1:[*]	-0.592	0 out of 1
FCFP_12	1747267175	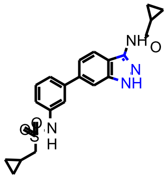 [*][c]1:[*]:[*]:[nH]:n:1	-0.592	0 out of 1

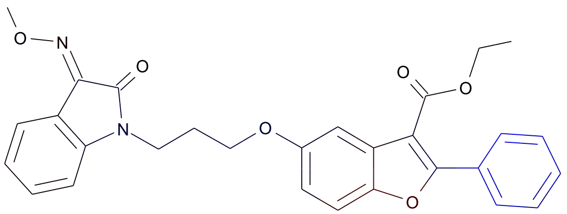

$C_{29}H_{26}N_2O_6$
Molecular Weight: 498.52653
ALogP: 4.994
Rotatable Bonds: 10
Acceptors: 6
Donors: 0

Structural Similar Compounds

Name	Nefazodone	Verapamil	Ketoconazole
Structure	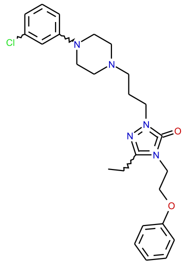	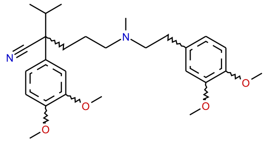	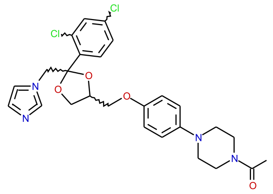
Actual Endpoint	Non-Carcinogen	Non-Carcinogen	Non-Carcinogen
Predicted Endpoint	Non-Carcinogen	Non-Carcinogen	Non-Carcinogen
Distance	0.607	0.702	0.712
Reference	US FDA (Centre for Drug Eval.& Res./Off. Testing & Res.) Sept. 1997	US FDA (Centre for Drug Eval.& Res./Off. Testing & Res.) Sept. 1997	US FDA (Centre for Drug Eval.& Res./Off. Testing & Res.) Sept. 1997

Model Applicability

Unknown features are fingerprint features in the query molecule, but not found in the training set.

1. All properties and OPS components are within expected ranges.
2. Unknown ECFP_2 feature: 1716966732: [*]\N=C/1\C(=[*])[*][*]:[c]1:[*]
3. Unknown ECFP_2 feature: -820505146: [*]ON=C([*])[*]
4. Unknown ECFP_2 feature: -408704017: [*]=NOC

Model Prediction

Prediction: Non-Carcinogen
Probability: 0.229
Enrichment: 0.712
Bayesian Score: -4.311
Mahalanobis Distance: 17.382
Mahalanobis Distance p-value: 3.78e-014

Prediction: Positive if the Bayesian score is above the estimated best cutoff value from minimizing the false positive and false negative rate.
Probability: The esimated probability that the sample is in the positive category. This assumes that the Bayesian score follows a normal distribution and is different from the prediction using a cutoff.
Enrichment: An estimate of enrichment, that is, the increased likelihood (versus random) of this sample being in the category.
Bayesian Score: The standard Laplacian-modified Bayesian score.
Mahalanobis Distance: The Mahalanobis distance (MD) is the distance to the center of the training data. The larger the MD, the less trustworthy the prediction.
Mahalanobis Distance p-value: The p-value gives the fraction of training data with an MD greater than or equal to the one for the given sample, assuming normally distributed data. The smaller the p-value, the less trustworthy the prediciton. For highly non-normal X properties (e.g., fingerprints), the MD p-value is wildly inaccurate.

Feature Contribution

Top features for positive contribution				
**Fingerprint**	**Bit/Smiles**	**Feature Structure**	**Score**	**Carcinogen in training set**
ECFP_12	1203316083	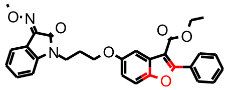 [*][c]1:[*]:[*]:[c](:[*]):o:1	0.681	9 out of 13

ECFP_12	1334014211	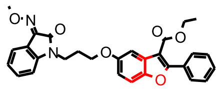 [*]:[cH]:[c]1:o:[*]:[*]:[c]:1:[*]	0.613	2 out of 2
ECFP_12	-289253356	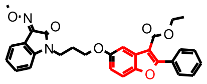 [*][c]1:o:[c]2:[cH]:[cH]:[*]:[cH]:[c]:2:[c]:1[*]	0.421	1 out of 1
**Top Features for negative contribution**				
**Fingerprint**	**Bit/Smiles**	**Feature Structure**	**Score**	**Carcinogen in training set**
ECFP_12	-840989128	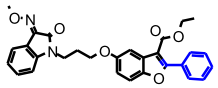 [*]:[c](:[*])[c]1:[cH]:[cH]:[cH]:[cH]:[cH]:1	-0.797	1 out of 11
ECFP_12	-212601949	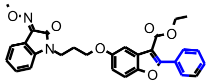 [*]:[c](:[*])[c]1:[cH]:[*]:[cH]:[cH]:[cH] :1	-0.797	1 out of 11
ECFP_12	-281505363	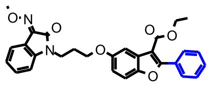 [*][c]1:[cH]:[cH]:[cH]:[cH]:[cH]:1	-0.560	11 out of 64

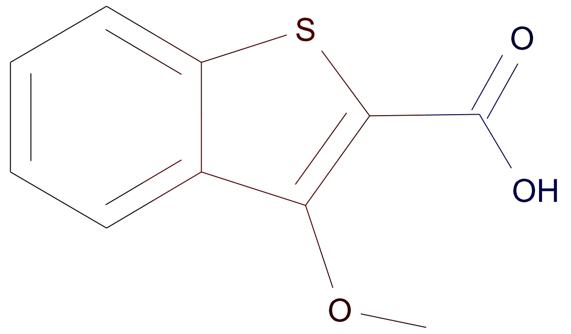

$C_{10}H_8O_3S$
Molecular Weight: 208.23372
ALogP: 2.596
Rotatable Bonds: 2
Acceptors: 3
Donors: 1

Model Prediction

Prediction: Carcinogen
Probability: 0.301
Enrichment: 0.934
Bayesian Score: 0.105
Mahalanobis Distance: 10.422
Mahalanobis Distance p-value: 0.201

Prediction: Positive if the Bayesian score is above the estimated best cutoff value from minimizing the false positive and false negative rate.
Probability: The esimated probability that the sample is in the positive category. This assumes that the Bayesian score follows a normal distribution and is different from the prediction using a cutoff.
Enrichment: An estimate of enrichment, that is, the increased likelihood (versus random) of this sample being in the category.
Bayesian Score: The standard Laplacian-modified Bayesian score.
Mahalanobis Distance: The Mahalanobis distance (MD) is the distance to the center of the training data. The larger the MD, the less trustworthy the prediction.
Mahalanobis Distance p-value: The p-value gives the fraction of training data with an MD greater than or equal to the one for the given sample, assuming normally distributed data. The smaller the p-value, the less trustworthy the prediciton. For highly non-normal X properties (e.g., fingerprints), the MD p-value is wildly inaccurate.

Structural Similar Compounds

Name	Aspirin	Thiabendazole	Suprofen
Structure	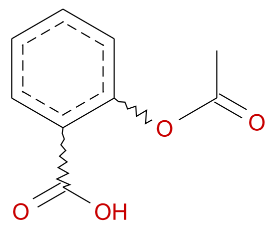	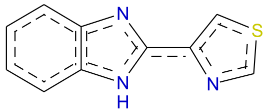	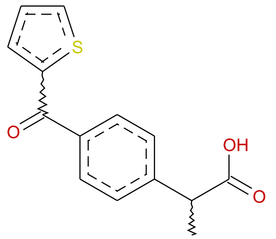
Actual Endpoint	Non-Carcinogen	Non-Carcinogen	Non-Carcinogen
Predicted Endpoint	Non-Carcinogen	Non-Carcinogen	Non-Carcinogen
Distance	0.570	0.579	0.613
Reference	US FDA (Centre for Drug Eval.& Res./Off. Testing & Res.) Sept. 1997	US FDA (Centre for Drug Eval.& Res./Off. Testing & Res.) Sept. 1997	US FDA (Centre for Drug Eval.& Res./Off. Testing & Res.) Sept. 1997

Model Applicability

Unknown features are fingerprint features in the query molecule, but not found in the training set.

1. All properties and OPS components are within expected ranges.
2. Unknown ECFP_2 feature: -1670580914: [*]C(=[*])[c]1:s:[*]:[*]:[c]:1[*]
3. Unknown ECFP_2 feature: 1895035276: [*]:[cH]:[c]1:s:[*]:[*]:[c]:1:[*]

Feature Contribution

Top features for positive contribution

Fingerprint	Bit/Smiles	Feature Structure	Score	Carcinogen in training set
ECFP_12	85262808	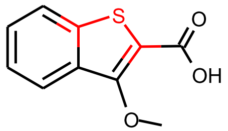 [*][c]1:[*]:[*]:[c](:[*]):s:1	0.851	5 out of 5

ECFP_12	2052151141	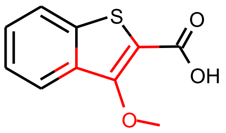 [*][c]1:[*]:[*]:[c](:[*]):[c]:1OC	0.668	4 out of 5
ECFP_12	914325265	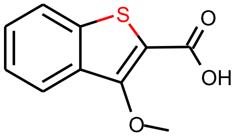 [*]:s:[*]	0.516	8 out of 14
**Top Features for negative contribution**				
**Fingerprint**	**Bit/Smiles**	**Feature Structure**	**Score**	**Carcinogen in training set**
ECFP_12	1429461619	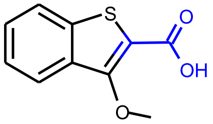 [*]:[c](:[*])C(=O)O	-0.989	1 out of 14
ECFP_12	2025485523	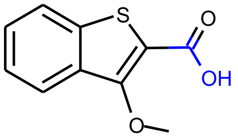 [*]C(=[*])O	-0.366	14 out of 66
ECFP_12	1997021792	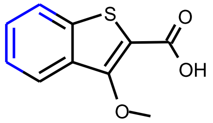 [*]:[cH]:[cH]:[cH]:[*]	-0.296	36 out of 156

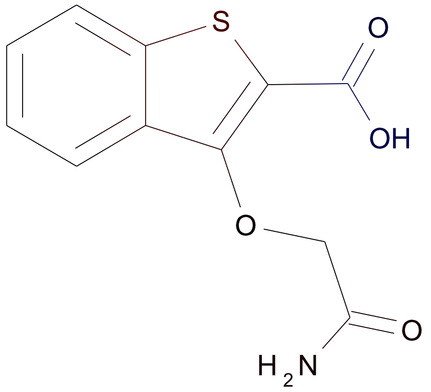

$C_{11}H_9NO_4S$
Molecular Weight: 251.25846
ALogP: 1.45
Rotatable Bonds: 4
Acceptors: 4
Donors: 2

Model Prediction

Prediction: Carcinogen
Probability: 0.298
Enrichment: 0.926
Bayesian Score: -0.020
Mahalanobis Distance: 13.514
Mahalanobis Distance p-value: 1.87e-005

Prediction: Positive if the Bayesian score is above the estimated best cutoff value from minimizing the false positive and false negative rate.
Probability: The esimated probability that the sample is in the positive category. This assumes that the Bayesian score follows a normal distribution and is different from the prediction using a cutoff.
Enrichment: An estimate of enrichment, that is, the increased likelihood (versus random) of this sample being in the category.
Bayesian Score: The standard Laplacian-modified Bayesian score.
Mahalanobis Distance: The Mahalanobis distance (MD) is the distance to the center of the training data. The larger the MD, the less trustworthy the prediction.
Mahalanobis Distance p-value: The p-value gives the fraction of training data with an MD greater than or equal to the one for the given sample, assuming normally distributed data. The smaller the p-value, the less trustworthy the prediciton. For highly non-normal X properties (e.g., fingerprints), the MD p-value is wildly inaccurate.

Structural Similar Compounds

Name	Nithiazide	Sulfamethoxazole	Sulfisoxazole
Structure	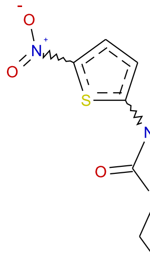	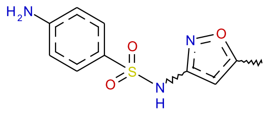	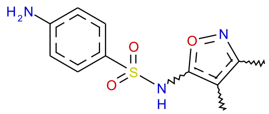
Actual Endpoint	Carcinogen	Carcinogen	Non-Carcinogen
Predicted Endpoint	Carcinogen	Carcinogen	Carcinogen
Distance	0.583	0.586	0.611
Reference	US FDA (Centre for Drug Eval.& Res./Off. Testing & Res.) Sept. 1997	US FDA (Centre for Drug Eval.& Res./Off. Testing & Res.) Sept. 1997	US FDA (Centre for Drug Eval.& Res./Off. Testing & Res.) Sept. 1997

Model Applicability

Unknown features are fingerprint features in the query molecule, but not found in the training set.

1. All properties and OPS components are within expected ranges.
2. Unknown ECFP_2 feature: -1670580914: [*]C(=[*])[c]1:s:[*]:[*]:[c]:1[*]
3. Unknown ECFP_2 feature: 1895035276: [*]:[cH]:[c]1:s:[*]:[*]:[c]:1:[*]

Feature Contribution

Top features for positive contribution				
**Fingerprint**	**Bit/Smiles**	**Feature Structure**	**Score**	**Carcinogen in training set**
ECFP_12	85262808	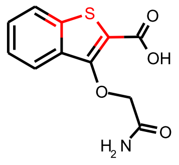 [*][c]1:[*]:[*]:[c](:[*]):s:1	0.851	5 out of 5

ECFP_12	914325265	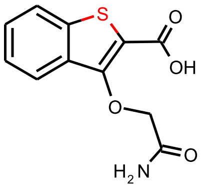 [*]:s:[*]	0.516	8 out of 14
ECFP_12	-1531301414	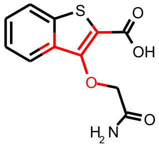 [*]O[c]1:[c]([*]):[*]:[*]:[c]:1:[*]	0.454	5 out of 9
**Top Features for negative contribution**				
**Fingerprint**	**Bit/Smiles**	**Feature Structure**	**Score**	**Carcinogen in training set**
ECFP_12	1429461619	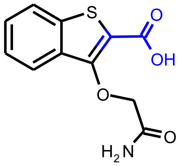 [*]:[c](:[*])C(=O)O	-0.989	1 out of 14
ECFP_12	2025485523	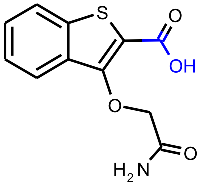 [*]C(=[*])O	-0.366	14 out of 66
ECFP_12	1997021792	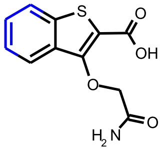 [*]:[cH]:[cH]:[cH]:[*]	-0.296	36 out of 156

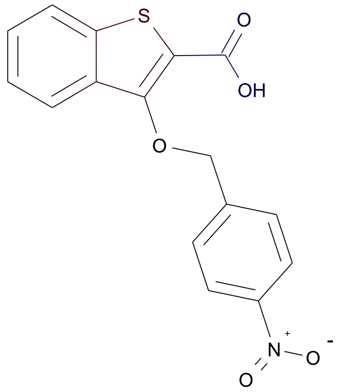

$C_{16}H_{11}NO_5S$

Molecular Weight: 329.32724

ALogP: 4.074

Rotatable Bonds: 5

Acceptors: 5

Donors: 1

Model Prediction

Prediction: Carcinogen

Probability: 0.280

Enrichment: 0.869

Bayesian Score: -0.975

Mahalanobis Distance: 14.340

Mahalanobis Distance p-value: 4.74e-007

Prediction: Positive if the Bayesian score is above the estimated best cutoff value from minimizing the false positive and false negative rate.
Probability: The esimated probability that the sample is in the positive category. This assumes that the Bayesian score follows a normal distribution and is different from the prediction using a cutoff.
Enrichment: An estimate of enrichment, that is, the increased likelihood (versus random) of this sample being in the category.
Bayesian Score: The standard Laplacian-modified Bayesian score.
Mahalanobis Distance: The Mahalanobis distance (MD) is the distance to the center of the training data. The larger the MD, the less trustworthy the prediction.
Mahalanobis Distance p-value: The p-value gives the fraction of training data with an MD greater than or equal to the one for the given sample, assuming normally distributed data. The smaller the p-value, the less trustworthy the prediciton. For highly non-normal X properties (e.g., fingerprints), the MD p-value is wildly inaccurate.

Structural Similar Compounds

Name	Omeprazole	Niclosamide	Lansoprazole
Structure	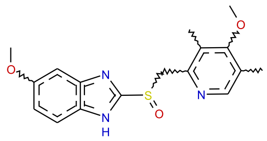	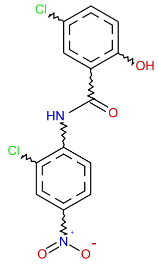	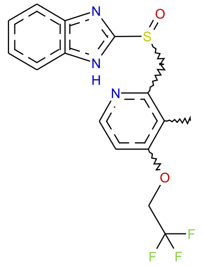
Actual Endpoint	Carcinogen	Non-Carcinogen	Carcinogen
Predicted Endpoint	Carcinogen	Non-Carcinogen	Carcinogen
Distance	0.675	0.701	0.702
Reference	US FDA (Centre for Drug Eval.& Res./Off. Testing & Res.) Sept. 1997	US FDA (Centre for Drug Eval.& Res./Off. Testing & Res.) Sept. 1997	US FDA (Centre for Drug Eval.& Res./Off. Testing & Res.) Sept. 1997

Model Applicability

Unknown features are fingerprint features in the query molecule, but not found in the training set.

1. All properties and OPS components are within expected ranges.
2. Unknown ECFP_2 feature: 1043790491: [*][N+](=[*])[*]
3. Unknown ECFP_2 feature: 781519895: [*][O-]
4. Unknown ECFP_2 feature: -1670580914: [*]C(=[*])[c]1:s:[*]:[*]:[c]:1[*]
5. Unknown ECFP_2 feature: 1895035276: [*]:[cH]:[c]1:s:[*]:[*]:[c]:1:[*]
6. Unknown ECFP_2 feature: -179073144: [*][N+](=[*])[c](:[cH]:[*]):[cH]:[*]
7. Unknown ECFP_2 feature: -215026467: [*]:[c](:[*])[N+](=O)[O-]
8. Unknown ECFP_2 feature: 2104376220: [*][N+](=O)[*]
9. Unknown ECFP_2 feature: -659271057: [*][N+](=[*])[O-]

Feature Contribution

Top features for positive contribution				
Fingerprint	Bit/Smiles	Feature Structure	Score	Carcinogen in training set

ECFP_12	85262808	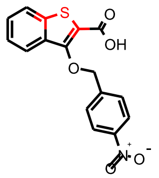 [*][c]1:[*]:[*]:[c](:[*]):s:1	0.851	5 out of 5
ECFP_12	914325265	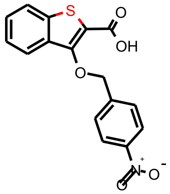 [*]:s:[*]	0.516	8 out of 14
ECFP_12	-1531301414	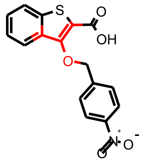 [*]O[c]1:[c]([*]):[*]:[*]:[c]:1:[*]	0.454	5 out of 9

Top Features for negative contribution

Fingerprint	Bit/Smiles	Feature Structure	Score	Carcinogen in training set
ECFP_12	1429461619	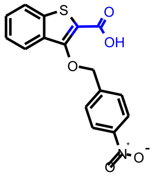 [*]:[c](:[*])C(=O)O	-0.989	1 out of 14
ECFP_12	2025485523	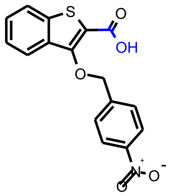 [*]C(=[*])O	-0.366	14 out of 66

ECFP_12	1997021792	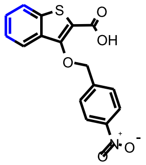 [*]:[cH]:[cH]:[cH]:[*]	-0.296	36 out of 156

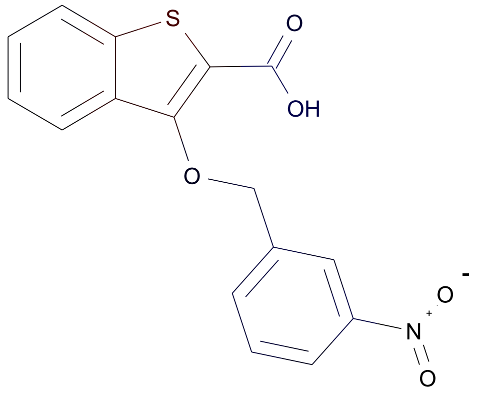

$C_{16}H_{11}NO_5S$
Molecular Weight: 329.32724
ALogP: 4.074
Rotatable Bonds: 5
Acceptors: 5
Donors: 1

Model Prediction

Prediction: Carcinogen
Probability: 0.267
Enrichment: 0.830
Bayesian Score: -1.697
Mahalanobis Distance: 15.431
Mahalanobis Distance p-value: 2.1e-009

Prediction: Positive if the Bayesian score is above the estimated best cutoff value from minimizing the false positive and false negative rate.
Probability: The esimated probability that the sample is in the positive category. This assumes that the Bayesian score follows a normal distribution and is different from the prediction using a cutoff.
Enrichment: An estimate of enrichment, that is, the increased likelihood (versus random) of this sample being in the category.
Bayesian Score: The standard Laplacian-modified Bayesian score.
Mahalanobis Distance: The Mahalanobis distance (MD) is the distance to the center of the training data. The larger the MD, the less trustworthy the prediction.
Mahalanobis Distance p-value: The p-value gives the fraction of training data with an MD greater than or equal to the one for the given sample, assuming normally distributed data. The smaller the p-value, the less trustworthy the prediciton. For highly non-normal X properties (e.g., fingerprints), the MD p-value is wildly inaccurate.

Structural Similar Compounds

Name	Omeprazole	Niclosamide	Lansoprazole
Structure	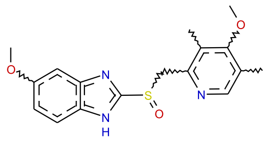	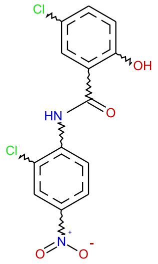	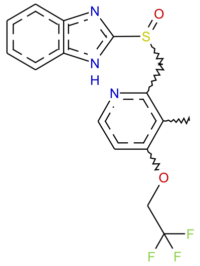
Actual Endpoint	Carcinogen	Non-Carcinogen	Carcinogen
Predicted Endpoint	Carcinogen	Non-Carcinogen	Carcinogen
Distance	0.671	0.697	0.702
Reference	US FDA (Centre for Drug Eval.& Res./Off. Testing & Res.) Sept. 1997	US FDA (Centre for Drug Eval.& Res./Off. Testing & Res.) Sept. 1997	US FDA (Centre for Drug Eval.& Res./Off. Testing & Res.) Sept. 1997

Model Applicability

Unknown features are fingerprint features in the query molecule, but not found in the training set.

1. All properties and OPS components are within expected ranges.
2. Unknown ECFP_2 feature: 1043790491: [*][N+](=[*])[*]
3. Unknown ECFP_2 feature: 781519895: [*][O-]
4. Unknown ECFP_2 feature: -1670580914: [*]C(=[*])[c]1:s:[*]:[*]:[c]:1[*]
5. Unknown ECFP_2 feature: 1895035276: [*]:[cH]:[c]1:s:[*]:[*]:[c]:1:[*]
6. Unknown ECFP_2 feature: -179073144: [*][N+](=[*])[c](:[cH]:[*]):[cH]:[*]
7. Unknown ECFP_2 feature: -215026467: [*]:[c](:[*])[N+](=O)[O-]
8. Unknown ECFP_2 feature: 2104376220: [*][N+](=O)[*]
9. Unknown ECFP_2 feature: -659271057: [*][N+](=[*])[O-]

Feature Contribution

Top features for positive contribution				
Fingerprint	Bit/Smiles	Feature Structure	Score	Carcinogen in training set

ECFP_12	85262808	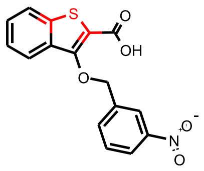 [*][c]1:[*]:[*]:[c](:[*]):s:1	0.851	5 out of 5
ECFP_12	914325265	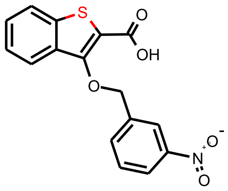 [*]:s:[*]	0.516	8 out of 14
ECFP_12	-1531301414	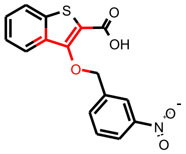 [*]O[c]1:[c]([*]):[*]:[*]:[c]:1:[*]	0.454	5 out of 9

Top Features for negative contribution

Fingerprint	Bit/Smiles	Feature Structure	Score	Carcinogen in training set
ECFP_12	1429461619	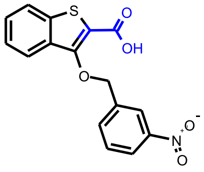 [*]:[c](:[*])C(=O)O	-0.989	1 out of 14
ECFP_12	-1845486197	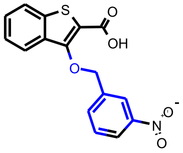 [*]OC[c]1:[cH]:[cH]:[*]:[c]([*]):[cH]:1	-0.485	0 out of 2

ECFP_12	2007300961	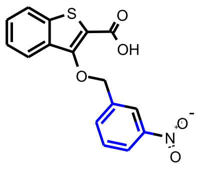 [*][c]1:[*]:[c]([*]):[cH]:[cH]:[cH]:1	-0.426	7 out of 36

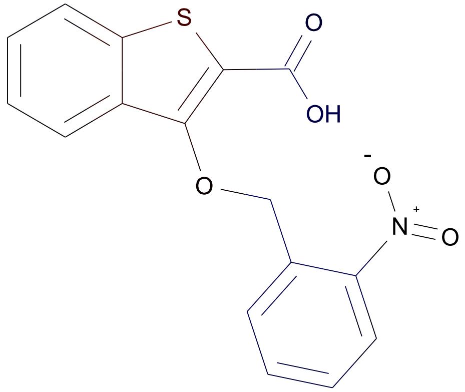

$C_{16}H_{11}NO_5S$
Molecular Weight: 329.32724
ALogP: 4.074
Rotatable Bonds: 5
Acceptors: 5
Donors: 1

Model Prediction

Prediction: Non-Carcinogen
Probability: 0.260
Enrichment: 0.808
Bayesian Score: -2.121
Mahalanobis Distance: 12.120
Mahalanobis Distance p-value: 0.00327

Prediction: Positive if the Bayesian score is above the estimated best cutoff value from minimizing the false positive and false negative rate.
Probability: The esimated probability that the sample is in the positive category. This assumes that the Bayesian score follows a normal distribution and is different from the prediction using a cutoff.
Enrichment: An estimate of enrichment, that is, the increased likelihood (versus random) of this sample being in the category.
Bayesian Score: The standard Laplacian-modified Bayesian score.
Mahalanobis Distance: The Mahalanobis distance (MD) is the distance to the center of the training data. The larger the MD, the less trustworthy the prediction.
Mahalanobis Distance p-value: The p-value gives the fraction of training data with an MD greater than or equal to the one for the given sample, assuming normally distributed data. The smaller the p-value, the less trustworthy the prediciton. For highly non-normal X properties (e.g., fingerprints), the MD p-value is wildly inaccurate.

Structural Similar Compounds

Name	Omeprazole	Niclosamide	Lansoprazole
Structure	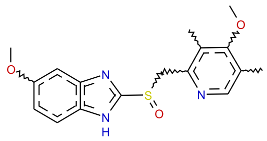	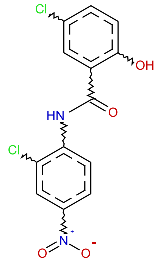	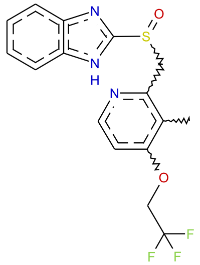
Actual Endpoint	Carcinogen	Non-Carcinogen	Carcinogen
Predicted Endpoint	Carcinogen	Non-Carcinogen	Carcinogen
Distance	0.674	0.700	0.701
Reference	US FDA (Centre for Drug Eval.& Res./Off. Testing & Res.) Sept. 1997	US FDA (Centre for Drug Eval.& Res./Off. Testing & Res.) Sept. 1997	US FDA (Centre for Drug Eval.& Res./Off. Testing & Res.) Sept. 1997

Model Applicability

Unknown features are fingerprint features in the query molecule, but not found in the training set.

1. All properties and OPS components are within expected ranges.
2. Unknown ECFP_2 feature: 1043790491: [*][N+](=[*])[*]
3. Unknown ECFP_2 feature: 781519895: [*][O-]
4. Unknown ECFP_2 feature: -1670580914: [*]C(=[*])[c]1:s:[*]:[*]:[c]:1[*]
5. Unknown ECFP_2 feature: 1895035276: [*]:[cH]:[c]1:s:[*]:[*]:[c]:1:[*]
6. Unknown ECFP_2 feature: -1956535100: [*][c](:[*]):[c](:[cH]:[*])[N+](=[*])[*]
7. Unknown ECFP_2 feature: -215026467: [*]:[c](:[*])[N+](=O)[O-]
8. Unknown ECFP_2 feature: 2104376220: [*][N+](=O)[*]
9. Unknown ECFP_2 feature: -659271057: [*][N+](=[*])[O-]

Feature Contribution

Top features for positive contribution				
Fingerprint	Bit/Smiles	Feature Structure	Score	Carcinogen in training set

ECFP_12	85262808	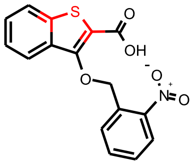 [*][c]1:[*]:[*]:[c](:[*]):s:1	0.851	5 out of 5
ECFP_12	914325265	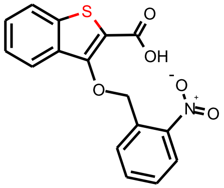 [*]:s:[*]	0.516	8 out of 14
ECFP_12	-1531301414	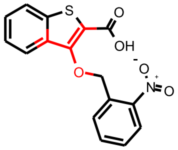 [*]O[c]1:[c]([*]):[*]:[*]:[c]:1:[*]	0.454	5 out of 9

Top Features for negative contribution

Fingerprint	Bit/Smiles	Feature Structure	Score	Carcinogen in training set
ECFP_12	1429461619	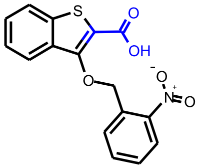 [*]:[c](:[*])C(=O)O	-0.989	1 out of 14
ECFP_12	1584051730	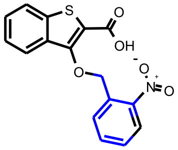 [*]C[c]1:[cH]:[cH]:[cH]:[*]:[c]:1[*]	-0.811	0 out of 4

ECFP_12	-358687393	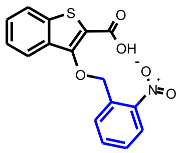 [*]C[c]1:[cH]:[cH]:[cH]:[cH]:[c]:1[*]	-0.485	0 out of 2

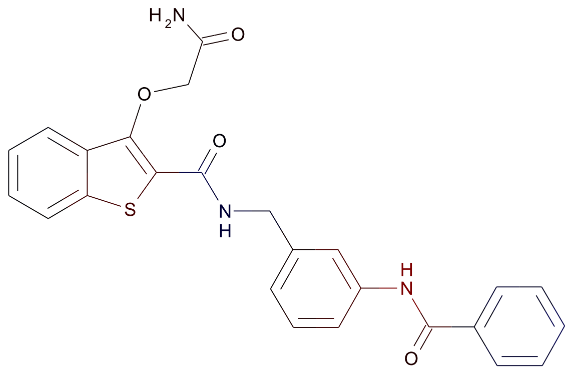

$C_{25}H_{21}N_3O_4S$
Molecular Weight: 459.51694
ALogP: 3.396
Rotatable Bonds: 8
Acceptors: 4
Donors: 3

Structural Similar Compounds

Name	Glimepride	Glyburide	Fluvastatin
Structure	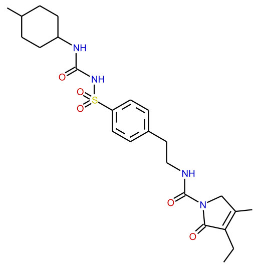	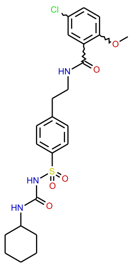	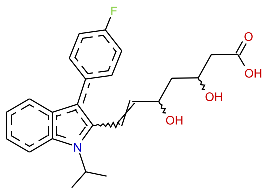
Actual Endpoint	Non-Carcinogen	Non-Carcinogen	Non-Carcinogen
Predicted Endpoint	Non-Carcinogen	Non-Carcinogen	Non-Carcinogen
Distance	0.616	0.616	0.658
Reference	US FDA (Centre for Drug Eval.& Res./Off. Testing & Res.) Sept. 1997	US FDA (Centre for Drug Eval.& Res./Off. Testing & Res.) Sept. 1997	US FDA (Centre for Drug Eval.& Res./Off. Testing & Res.) Sept. 1997

Model Prediction

Prediction: Carcinogen
Probability: 0.297
Enrichment: 0.922
Bayesian Score: -0.082
Mahalanobis Distance: 17.924
Mahalanobis Distance p-value: 1.47e-015

Prediction: Positive if the Bayesian score is above the estimated best cutoff value from minimizing the false positive and false negative rate.
Probability: The esimated probability that the sample is in the positive category. This assumes that the Bayesian score follows a normal distribution and is different from the prediction using a cutoff.
Enrichment: An estimate of enrichment, that is, the increased likelihood (versus random) of this sample being in the category.
Bayesian Score: The standard Laplacian-modified Bayesian score.
Mahalanobis Distance: The Mahalanobis distance (MD) is the distance to the center of the training data. The larger the MD, the less trustworthy the prediction.
Mahalanobis Distance p-value: The p-value gives the fraction of training data with an MD greater than or equal to the one for the given sample, assuming normally distributed data. The smaller the p-value, the less trustworthy the prediciton. For highly non-normal X properties (e.g., fingerprints), the MD p-value is wildly inaccurate.

Model Applicability

Unknown features are fingerprint features in the query molecule, but not found in the training set.

1. All properties and OPS components are within expected ranges.
2. Unknown ECFP_2 feature: -1670580914: [*]C(=[*])[c]1:s:[*]:[*]:[c]:1[*]
3. Unknown ECFP_2 feature: 1895035276: [*]:[cH]:[c]1:s:[*]:[*]:[c]:1:[*]

Feature Contribution

Top features for positive contribution

Fingerprint	Bit/Smiles	Feature Structure	Score	Carcinogen in training set
ECFP_12	85262808	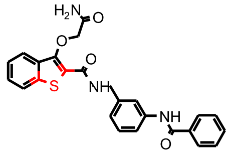 [*][c]1:[*]:[*]:[c](:[*]):s:1	0.851	5 out of 5

ECFP_12	-223149939	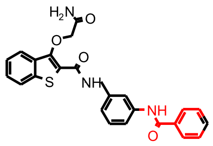 [*]NC(=O)[c]1:[cH]:[cH]:[*]:[cH]:[cH]:1	0.613	2 out of 2
ECFP_12	-177077903	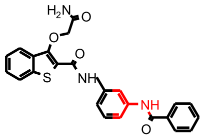 [*]N[c](:[cH]:[*]):[cH]:[*]	0.529	6 out of 10
**Top Features for negative contribution**				
**Fingerprint**	**Bit/Smiles**	**Feature Structure**	**Score**	**Carcinogen in training set**
ECFP_12	497523368	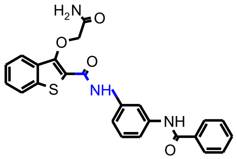 [*]CNC(=[*])[*]	-0.989	1 out of 14
ECFP_12	-281505363	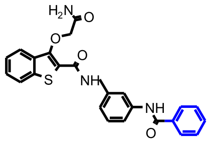 [*][c]1:[cH]:[cH]:[cH]:[cH]:[cH]:1	-0.560	11 out of 64
ECFP_12	1571214559	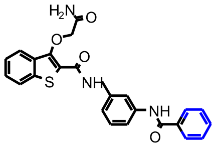 [*]1:[cH]:[cH]:[cH]:[cH]:[cH]:1	-0.560	11 out of 64

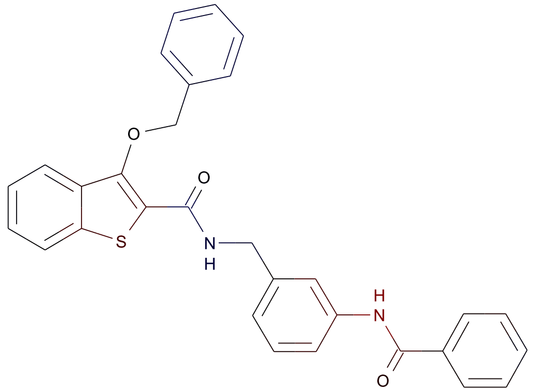

$C_{30}H_{24}N_2O_3S$
Molecular Weight: 492.58816
ALogP: 6.126
Rotatable Bonds: 8
Acceptors: 3
Donors: 2

Model Prediction

Prediction: Carcinogen
Probability: 0.308
Enrichment: 0.958
Bayesian Score: 0.473
Mahalanobis Distance: 17.376
Mahalanobis Distance p-value: 3.93e-014

Prediction: Positive if the Bayesian score is above the estimated best cutoff value from minimizing the false positive and false negative rate.
Probability: The esimated probability that the sample is in the positive category. This assumes that the Bayesian score follows a normal distribution and is different from the prediction using a cutoff.
Enrichment: An estimate of enrichment, that is, the increased likelihood (versus random) of this sample being in the category.
Bayesian Score: The standard Laplacian-modified Bayesian score.
Mahalanobis Distance: The Mahalanobis distance (MD) is the distance to the center of the training data. The larger the MD, the less trustworthy the prediction.
Mahalanobis Distance p-value: The p-value gives the fraction of training data with an MD greater than or equal to the one for the given sample, assuming normally distributed data. The smaller the p-value, the less trustworthy the prediciton. For highly non-normal X properties (e.g., fingerprints), the MD p-value is wildly inaccurate.

Structural Similar Compounds

Name	Terfenadine	Pimozide	Astemizole
Structure	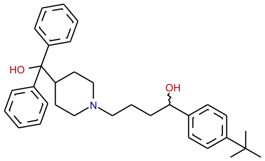	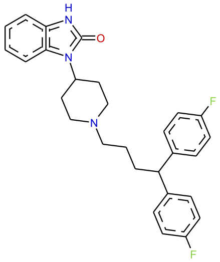	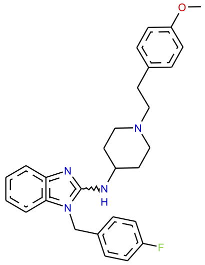
Actual Endpoint	Non-Carcinogen	Non-Carcinogen	Non-Carcinogen
Predicted Endpoint	Non-Carcinogen	Non-Carcinogen	Non-Carcinogen
Distance	0.650	0.705	0.712
Reference	US FDA (Centre for Drug Eval.& Res./Off. Testing & Res.) Sept. 1997	US FDA (Centre for Drug Eval.& Res./Off. Testing & Res.) Sept. 1997	US FDA (Centre for Drug Eval.& Res./Off. Testing & Res.) Sept. 1997

Model Applicability

Unknown features are fingerprint features in the query molecule, but not found in the training set.

1. All properties and OPS components are within expected ranges.
2. Unknown ECFP_2 feature: -1670580914: [*]C(=[*])[c]1:s:[*]:[*]:[c]:1[*]
3. Unknown ECFP_2 feature: 1895035276: [*]:[cH]:[c]1:s:[*]:[*]:[c]:1:[*]

Feature Contribution

Top features for positive contribution

Fingerprint	Bit/Smiles	Feature Structure	Score	Carcinogen in training set
ECFP_12	85262808	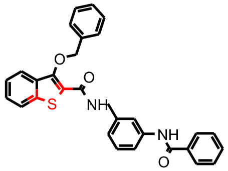 [*][c]1:[*]:[*]:[c](:[*]):s:1	0.851	5 out of 5

ECFP_12	-223149939	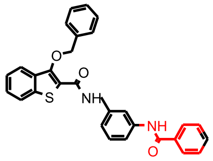 [*]NC(=O)[c]1:[cH]:[cH]:[*]:[cH]:[cH]:1	0.613	2 out of 2
ECFP_12	-177077903	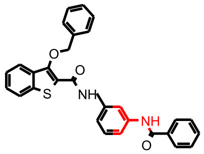 [*]N[c](:[cH]:[*]):[cH]:[*]	0.529	6 out of 10
**Top Features for negative contribution**				
**Fingerprint**	**Bit/Smiles**	**Feature Structure**	**Score**	**Carcinogen in training set**
ECFP_12	497523368	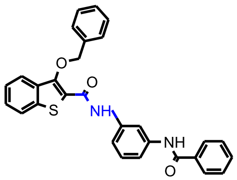 [*]CNC(=[*])[*]	-0.989	1 out of 14
ECFP_12	-281505363	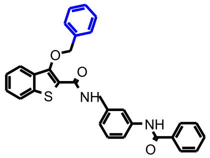 [*][c]1:[cH]:[cH]:[cH]:[cH]:[cH]:1	-0.560	11 out of 64
ECFP_12	1571214559	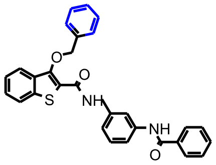 [*]1:[cH]:[cH]:[cH]:[cH]:[cH]:1	-0.560	11 out of 64

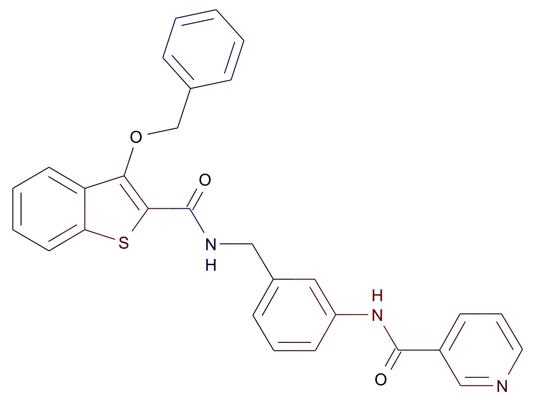

$C_{29}H_{23}N_3O_3S$
Molecular Weight: 493.57622
ALogP: 4.976
Rotatable Bonds: 8
Acceptors: 4
Donors: 2

Model Prediction

Prediction: Carcinogen
Probability: 0.327
Enrichment: 1.016
Bayesian Score: 1.318
Mahalanobis Distance: 18.125
Mahalanobis Distance p-value: 4.32e-016

Prediction: Positive if the Bayesian score is above the estimated best cutoff value from minimizing the false positive and false negative rate.
Probability: The esimated probability that the sample is in the positive category. This assumes that the Bayesian score follows a normal distribution and is different from the prediction using a cutoff.
Enrichment: An estimate of enrichment, that is, the increased likelihood (versus random) of this sample being in the category.
Bayesian Score: The standard Laplacian-modified Bayesian score.
Mahalanobis Distance: The Mahalanobis distance (MD) is the distance to the center of the training data. The larger the MD, the less trustworthy the prediction.
Mahalanobis Distance p-value: The p-value gives the fraction of training data with an MD greater than or equal to the one for the given sample, assuming normally distributed data. The smaller the p-value, the less trustworthy the prediciton. For highly non-normal X properties (e.g., fingerprints), the MD p-value is wildly inaccurate.

Structural Similar Compounds

Name	Glyburide	Fluvastatin	Flecainide
Structure	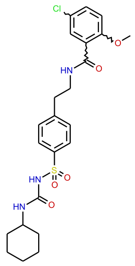	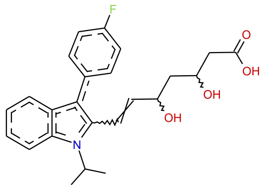	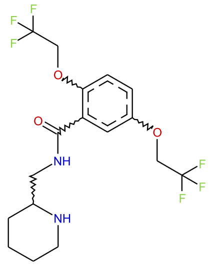
Actual Endpoint	Non-Carcinogen	Non-Carcinogen	Non-Carcinogen
Predicted Endpoint	Non-Carcinogen	Non-Carcinogen	Non-Carcinogen
Distance	0.661	0.690	0.693
Reference	US FDA (Centre for Drug Eval.& Res./Off. Testing & Res.) Sept. 1997	US FDA (Centre for Drug Eval.& Res./Off. Testing & Res.) Sept. 1997	US FDA (Centre for Drug Eval.& Res./Off. Testing & Res.) Sept. 1997

Model Applicability

Unknown features are fingerprint features in the query molecule, but not found in the training set.

1. All properties and OPS components are within expected ranges.
2. Unknown ECFP_2 feature: -1670580914: [*]C(=[*])[c]1:s:[*]:[*]:[c]:1[*]
3. Unknown ECFP_2 feature: 1895035276: [*]:[cH]:[c]1:s:[*]:[*]:[c]:1:[*]

Feature Contribution

Top features for positive contribution

Fingerprint	Bit/Smiles	Feature Structure	Score	Carcinogen in training set
ECFP_12	85262808	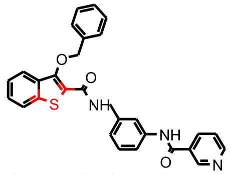 [*][c]1:[*]:[*]:[c](:[*]):s:1	0.851	5 out of 5

ECFP_12	-177077903	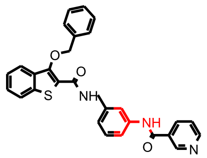 [*]N[c](:[cH]:[*]):[c H]:[*]	0.529	6 out of 10
ECFP_12	914325265	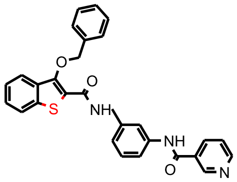 [*]:s:[*]	0.516	8 out of 14

Top Features for negative contribution

Fingerprint	Bit/Smiles	Feature Structure	Score	Carcinogen in training set
ECFP_12	497523368	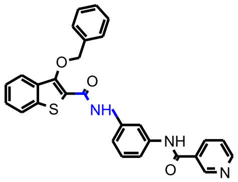 [*]CNC(=[*])[*]	-0.989	1 out of 14
ECFP_12	-281505363	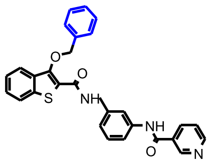 [*][c]1:[cH]:[cH]:[cH]:[cH]:[cH]:1	-0.560	11 out of 64
ECFP_12	1571214559	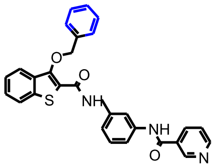 [*]1:[cH]:[cH]:[cH]:[cH]:[cH]:1	-0.560	11 out of 64

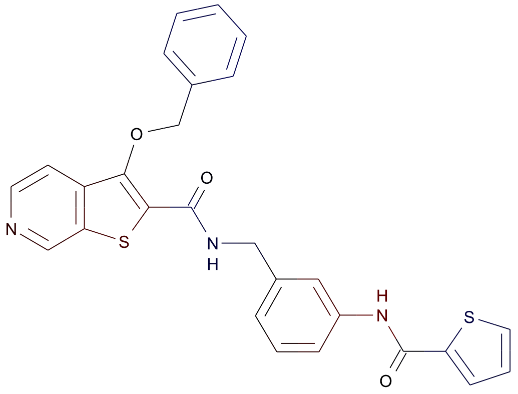

$C_{27}H_{21}N_3O_3S_2$
Molecular Weight: 499.60394
ALogP: 4.929
Rotatable Bonds: 8
Acceptors: 4
Donors: 2

Model Prediction

Prediction: Carcinogen
Probability: 0.283
Enrichment: 0.880
Bayesian Score: -0.780
Mahalanobis Distance: 16.881
Mahalanobis Distance p-value: 7.07e-013

Prediction: Positive if the Bayesian score is above the estimated best cutoff value from minimizing the false positive and false negative rate.
Probability: The esimated probability that the sample is in the positive category. This assumes that the Bayesian score follows a normal distribution and is different from the prediction using a cutoff.
Enrichment: An estimate of enrichment, that is, the increased likelihood (versus random) of this sample being in the category.
Bayesian Score: The standard Laplacian-modified Bayesian score.
Mahalanobis Distance: The Mahalanobis distance (MD) is the distance to the center of the training data. The larger the MD, the less trustworthy the prediction.
Mahalanobis Distance p-value: The p-value gives the fraction of training data with an MD greater than or equal to the one for the given sample, assuming normally distributed data. The smaller the p-value, the less trustworthy the prediciton. For highly non-normal X properties (e.g., fingerprints), the MD p-value is wildly inaccurate.

Structural Similar Compounds

Name	Glyburide	Glimepride	Fluvastatin
Structure	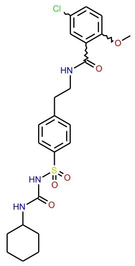	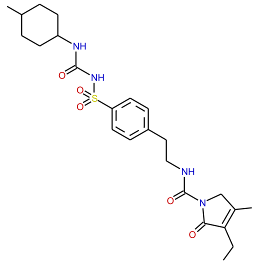	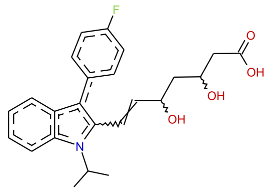
Actual Endpoint	Non-Carcinogen	Non-Carcinogen	Non-Carcinogen
Predicted Endpoint	Non-Carcinogen	Non-Carcinogen	Non-Carcinogen
Distance	0.660	0.690	0.726
Reference	US FDA (Centre for Drug Eval.& Res./Off. Testing & Res.) Sept. 1997	US FDA (Centre for Drug Eval.& Res./Off. Testing & Res.) Sept. 1997	US FDA (Centre for Drug Eval.& Res./Off. Testing & Res.) Sept. 1997

Model Applicability

Unknown features are fingerprint features in the query molecule, but not found in the training set.

1. All properties and OPS components are within expected ranges.
2. Unknown ECFP_2 feature: 1895035276: [*]:[cH]:[c]1:s:[*]:[*]:[c]:1:[*]
3. Unknown ECFP_2 feature: -1670580914: [*]C(=[*])[c]1:s:[*]:[*]:[c]:1[*]

Feature Contribution

Top features for positive contribution

Fingerprint	Bit/Smiles	Feature Structure	Score	Carcinogen in training set
ECFP_12	85262808	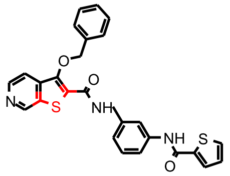 [*][c]1:[*]:[*]:[c](:[*]):s:1	0.851	5 out of 5

ECFP_12	-177077903	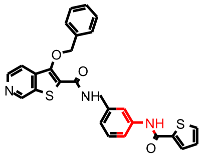 [*]N[c](:[cH]:[*]):[c H]:[*]	0.529	6 out of 10
ECFP_12	914325265	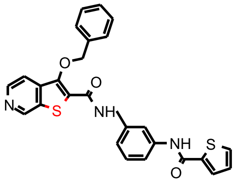 [*]:s:[*]	0.516	8 out of 14

Top Features for negative contribution

Fingerprint	Bit/Smiles	Feature Structure	Score	Carcinogen in training set
ECFP_12	497523368	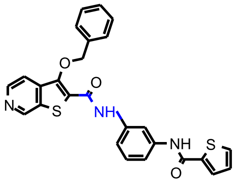 [*]CNC(=[*])[*]	-0.989	1 out of 14
ECFP_12	1571214559	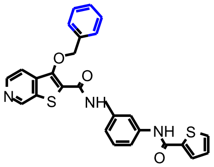 [*]1:[cH]:[cH]:[cH]:[cH]:[cH]:1	-0.560	11 out of 64
ECFP_12	-281505363	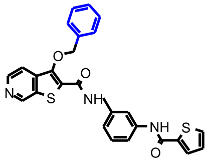 [*][c]1:[cH]:[cH]:[cH]:[cH]:[cH]:1	-0.560	11 out of 64

Co-crystallized ligand

TOPKAT_Rat_Female_FDA_None_vs_Carcinogen

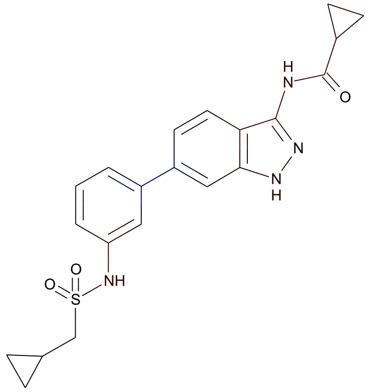

$C_{21}H_{22}N_4O_3S$

Molecular Weight: 410.48938

ALogP: 3.14

Rotatable Bonds: 7

Acceptors: 4

Donors: 3

Structural Similar Compounds

Name	Torsemide	Bicalutamide	Glimepride
Structure	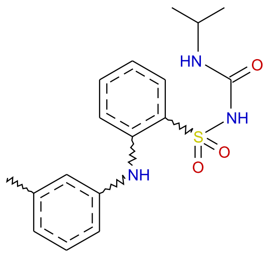	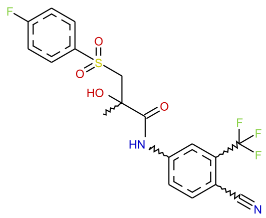	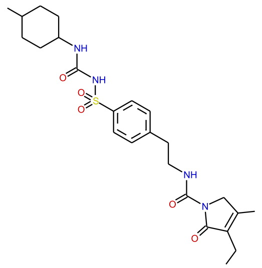
Actual Endpoint	Carcinogen	Carcinogen	Non-Carcinogen
Predicted Endpoint	Carcinogen	Carcinogen	Non-Carcinogen
Distance	0.630	0.650	0.654
Reference	US FDA (Centre for Drug Eval.& Res./Off. Testing & Res.) Sept. 1997	US FDA (Centre for Drug Eval.& Res./Off. Testing & Res.) Sept. 1997	US FDA (Centre for Drug Eval.& Res./Off. Testing & Res.) Sept. 1997

Model Prediction

Prediction: Carcinogen

Probability: 0.286

Enrichment: 0.888

Bayesian Score: -0.639

Mahalanobis Distance: 10.897

Mahalanobis Distance p-value: 0.0833

Prediction: Positive if the Bayesian score is above the estimated best cutoff value from minimizing the false positive and false negative rate.
Probability: The esimated probability that the sample is in the positive category. This assumes that the Bayesian score follows a normal distribution and is different from the prediction using a cutoff.
Enrichment: An estimate of enrichment, that is, the increased likelihood (versus random) of this sample being in the category.
Bayesian Score: The standard Laplacian-modified Bayesian score.
Mahalanobis Distance: The Mahalanobis distance (MD) is the distance to the center of the training data. The larger the MD, the less trustworthy the prediction.
Mahalanobis Distance p-value: The p-value gives the fraction of training data with an MD greater than or equal to the one for the given sample, assuming normally distributed data. The smaller the p-value, the less trustworthy the prediciton. For highly non-normal X properties (e.g., fingerprints), the MD p-value is wildly inaccurate.

Model Applicability

Unknown features are fingerprint features in the query molecule, but not found in the training set.

1. All properties and OPS components are within expected ranges.
2. Unknown ECFP_2 feature: 600440273: [*][c]1:[*]:[*]:[nH]:n:1
3. Unknown ECFP_2 feature: -1341194584: [*]S(=[*])(=[*])CC1[*][*]1

Feature Contribution

Top features for positive contribution

Fingerprint	Bit/Smiles	Feature Structure	Score	Carcinogen in training set
ECFP_12	-177077903	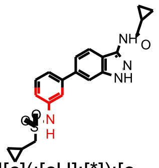 [*]N[c](:[cH]:[*]):[cH]:[*]	0.529	6 out of 10

ECFP_12	-1236483485	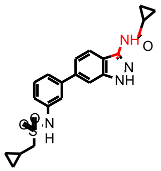 [*]C(=[*])N[c](:[*]):[*]	0.460	9 out of 17
ECFP_12	1099224616	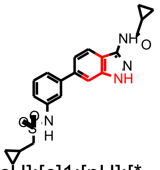 [*]:[cH]:[c]1:[nH]:[*]:[*]:[c]:1:[*]	0.456	6 out of 11
**Top Features for negative contribution**				
**Fingerprint**	**Bit/Smiles**	**Feature Structure**	**Score**	**Carcinogen in training set**
ECFP_12	-212601949	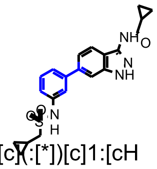 [*]:[c](:[*])[c]1:[cH]:[*]:[cH]:[cH]:[cH]:1	-0.797	1 out of 11
ECFP_12	-181568884	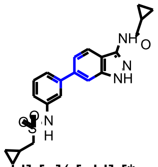 [*]:[cH]:[c](:[cH]:[*])[c](:[*]):[*]	-0.505	3 out of 18
ECFP_12	2007300961	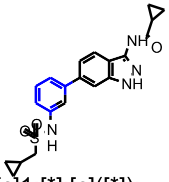 [*][c]1:[*]:[c]([*]):[cH]:[cH]:[cH]:1	-0.426	7 out of 36

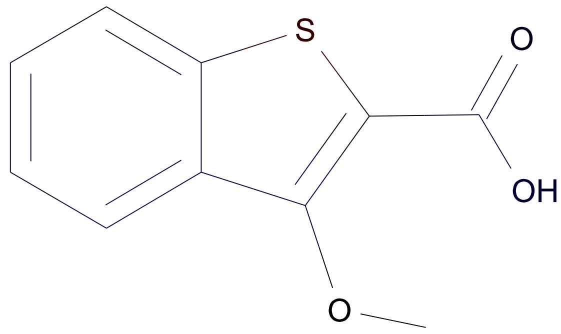

$C_{10}H_8O_3S$
Molecular Weight: 208.23372
ALogP: 2.596
Rotatable Bonds: 2
Acceptors: 3
Donors: 1

Model Prediction

Prediction: Single-Carcinogen
Probability: 0.473
Enrichment: 1.266
Bayesian Score: -1.311
Mahalanobis Distance: 12.486
Mahalanobis Distance p-value: 0.000659

Prediction: Positive if the Bayesian score is above the estimated best cutoff value from minimizing the false positive and false negative rate.
Probability: The esimated probability that the sample is in the positive category. This assumes that the Bayesian score follows a normal distribution and is different from the prediction using a cutoff.
Enrichment: An estimate of enrichment, that is, the increased likelihood (versus random) of this sample being in the category.
Bayesian Score: The standard Laplacian-modified Bayesian score.
Mahalanobis Distance: The Mahalanobis distance (MD) is the distance to the center of the training data. The larger the MD, the less trustworthy the prediction.
Mahalanobis Distance p-value: The p-value gives the fraction of training data with an MD greater than or equal to the one for the given sample, assuming normally distributed data. The smaller the p-value, the less trustworthy the prediciton. For highly non-normal X properties (e.g., fingerprints), the MD p-value is wildly inaccurate.

Structural Similar Compounds

Name	Cytembena	Methoxsalen; 8-	Milrinone
Structure	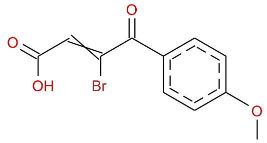	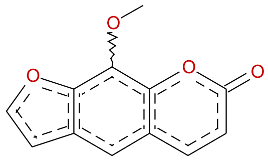	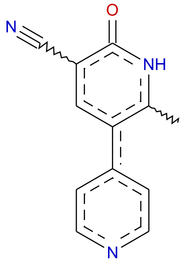
Actual Endpoint	Multiple-Carcinogen	Single-Carcinogen	Single-Carcinogen
Predicted Endpoint	Multiple-Carcinogen	Single-Carcinogen	Single-Carcinogen
Distance	0.602	0.624	0.632
Reference	US FDA (Centre for Drug Eval.& Res./Off. Testing & Res.) Sept. 1997	US FDA (Centre for Drug Eval.& Res./Off. Testing & Res.) Sept. 1997	US FDA (Centre for Drug Eval.& Res./Off. Testing & Res.) Sept. 1997

Model Applicability

Unknown features are fingerprint features in the query molecule, but not found in the training set.

1. All properties and OPS components are within expected ranges.

Feature Contribution

Top features for positive contribution

Fingerprint	Bit/Smiles	Feature Structure	Score	Multiple-Carcinogen in training set
SCFP_4	17	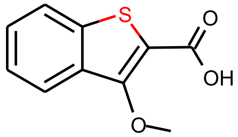 [*]:s:[*]	0.548	10 out of 17

SCFP_4	1310748454	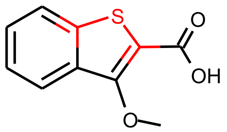<br>[*][c]1:[*]:[*]:[c](:[*]):s:1	0.444	4 out of 7
SCFP_4	3	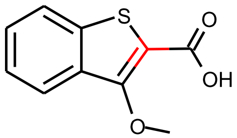<br>[*][c](:[*]):[*]	0.199	36 out of 93
**Top Features for negative contribution**				
**Fingerprint**	**Bit/Smiles**	**Feature Structure**	**Score**	**Multiple-Carcinogen in training set**
SCFP_4	112346096	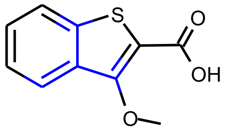<br>[*][c]1:[*]:[*]:[c](:[*]):[c]:1:[cH]:[*]	-0.730	1 out of 10
SCFP_4	1132967294	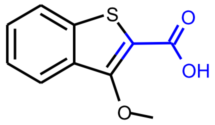<br>[*]:[c](:[*])C(=O)O	-0.666	0 out of 3
SCFP_4	-1379673609	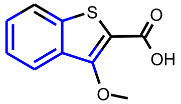<br>[*][c]1:[*]:[*]:[c]2:[*]:[cH]:[cH]:[cH]:[c]:1:2	-0.472	1 out of 7

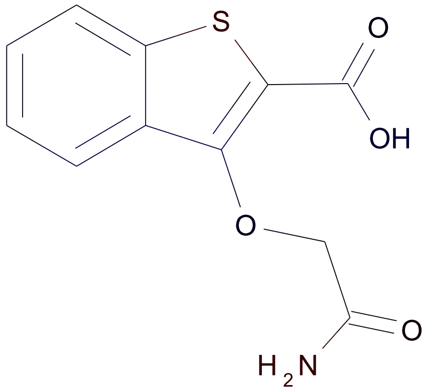

$C_{11}H_9NO_4S$
Molecular Weight: 251.25846
ALogP: 1.45
Rotatable Bonds: 4
Acceptors: 4
Donors: 2

Structural Similar Compounds

Name	Nithiazide	Sulfamethoxazole	Furothiazole
Structure	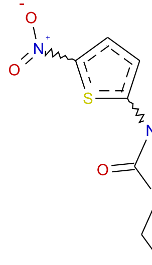	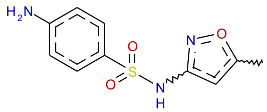	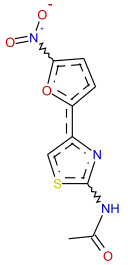
Actual Endpoint	Multiple-Carcinogen	Single-Carcinogen	Single-Carcinogen
Predicted Endpoint	Multiple-Carcinogen	Single-Carcinogen	Multiple-Carcinogen
Distance	0.555	0.563	0.607
Reference	US FDA (Centre for Drug Eval.& Res./Off. Testing & Res.) Sept. 1997	US FDA (Centre for Drug Eval.& Res./Off. Testing & Res.) Sept. 1997	US FDA (Centre for Drug Eval.& Res./Off. Testing & Res.) Sept. 1997

Model Prediction

Prediction: Single-Carcinogen
Probability: 0.509
Enrichment: 1.362
Bayesian Score: 0.006
Mahalanobis Distance: 14.626
Mahalanobis Distance p-value: 6.81e-006

Prediction: Positive if the Bayesian score is above the estimated best cutoff value from minimizing the false positive and false negative rate.
Probability: The esimated probability that the sample is in the positive category. This assumes that the Bayesian score follows a normal distribution and is different from the prediction using a cutoff.
Enrichment: An estimate of enrichment, that is, the increased likelihood (versus random) of this sample being in the category.
Bayesian Score: The standard Laplacian-modified Bayesian score.
Mahalanobis Distance: The Mahalanobis distance (MD) is the distance to the center of the training data. The larger the MD, the less trustworthy the prediction.
Mahalanobis Distance p-value: The p-value gives the fraction of training data with an MD greater than or equal to the one for the given sample, assuming normally distributed data. The smaller the p-value, the less trustworthy the prediciton. For highly non-normal X properties (e.g., fingerprints), the MD p-value is wildly inaccurate.

Model Applicability

Unknown features are fingerprint features in the query molecule, but not found in the training set.

1. All properties and OPS components are within expected ranges.

Feature Contribution

Top features for positive contribution

Fingerprint	Bit/Smiles	Feature Structure	Score	Multiple-Carcinogen in training set
SCFP_4	17	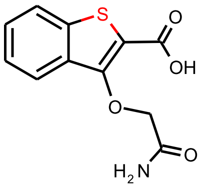 [*]:s:[*]	0.548	10 out of 17

SCFP_4	1310748454	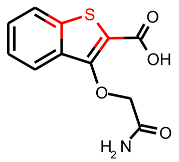 [*][c]1:[*]:[*]:[c](:[*]):s:1	0.444	4 out of 7
SCFP_4	-1357949052	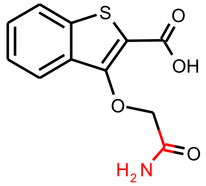 [*]C(=[*])N	0.444	4 out of 7

Top Features for negative contribution

Fingerprint	Bit/Smiles	Feature Structure	Score	Multiple-Carcinogen in training set
SCFP_4	112346096	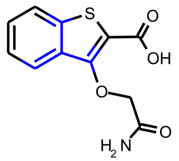 [*][c]1:[*]:[*]:[c](:[*]):[c]:1:[cH]:[*]	-0.730	1 out of 10
SCFP_4	1132967294	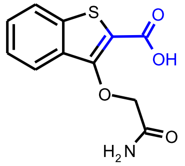 [*]:[c](:[*])C(=O)O	-0.666	0 out of 3
SCFP_4	-711656408	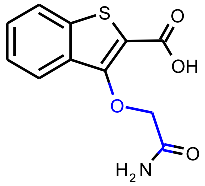 [*]OCC(=[*])[*]	-0.472	1 out of 7

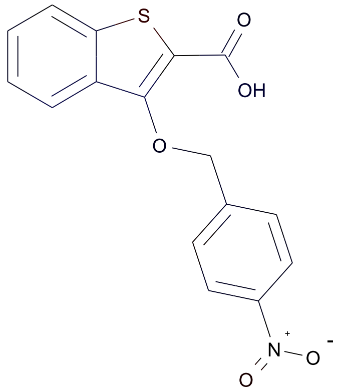

$C_{16}H_{11}NO_5S$
Molecular Weight: 329.32724
ALogP: 4.074
Rotatable Bonds: 5
Acceptors: 5
Donors: 1

Model Prediction

Prediction: Single-Carcinogen
Probability: 0.469
Enrichment: 1.255
Bayesian Score: -1.435
Mahalanobis Distance: 21.981
Mahalanobis Distance p-value: 9.24e-013

Prediction: Positive if the Bayesian score is above the estimated best cutoff value from minimizing the false positive and false negative rate.
Probability: The esimated probability that the sample is in the positive category. This assumes that the Bayesian score follows a normal distribution and is different from the prediction using a cutoff.
Enrichment: An estimate of enrichment, that is, the increased likelihood (versus random) of this sample being in the category.
Bayesian Score: The standard Laplacian-modified Bayesian score.
Mahalanobis Distance: The Mahalanobis distance (MD) is the distance to the center of the training data. The larger the MD, the less trustworthy the prediction.
Mahalanobis Distance p-value: The p-value gives the fraction of training data with an MD greater than or equal to the one for the given sample, assuming normally distributed data. The smaller the p-value, the less trustworthy the prediciton. For highly non-normal X properties (e.g., fingerprints), the MD p-value is wildly inaccurate.

Structural Similar Compounds

Name	Omeprazole	Dantrolene	Estrogens; conjug.
Structure	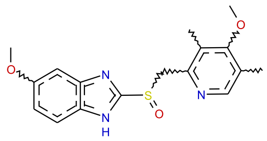	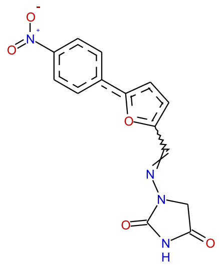	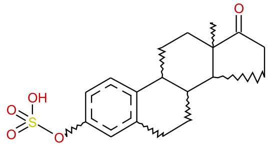
Actual Endpoint	Multiple-Carcinogen	Multiple-Carcinogen	Single-Carcinogen
Predicted Endpoint	Multiple-Carcinogen	Multiple-Carcinogen	Single-Carcinogen
Distance	0.627	0.645	0.659
Reference	US FDA (Centre for Drug Eval.& Res./Off. Testing & Res.) Sept. 1997	US FDA (Centre for Drug Eval.& Res./Off. Testing & Res.) Sept. 1997	US FDA (Centre for Drug Eval.& Res./Off. Testing & Res.) Sept. 1997

Model Applicability

Unknown features are fingerprint features in the query molecule, but not found in the training set.

1. All properties and OPS components are within expected ranges.

Feature Contribution

Top features for positive contribution

Fingerprint	Bit/Smiles	Feature Structure	Score	Multiple-Carcinogen in training set
SCFP_4	17	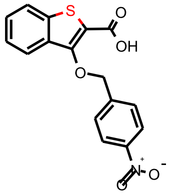 [*]:s:[*]	0.548	10 out of 17

SCFP_4	1311339974	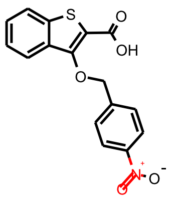 [*][N+](=O)[*]	0.504	9 out of 16
SCFP_4	1310748454	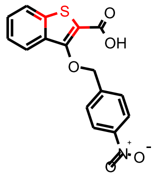 [*][c]1:[*]:[*]:[c](:[*]):s:1	0.444	4 out of 7

Top Features for negative contribution

Fingerprint	Bit/Smiles	Feature Structure	Score	Multiple-Carcinogen in training set
SCFP_4	112346096	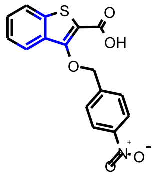 [*][c]1:[*]:[*]:[c](:[*]):[c]:1:[cH]:[*]	-0.730	1 out of 10
SCFP_4	1132967294	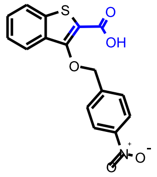 [*]:[c](:[*])C(=O)O	-0.666	0 out of 3
SCFP_4	-711596826	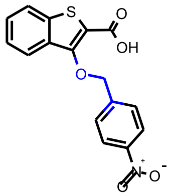 [*]OC[c](:[*]):[*]	-0.489	0 out of 2

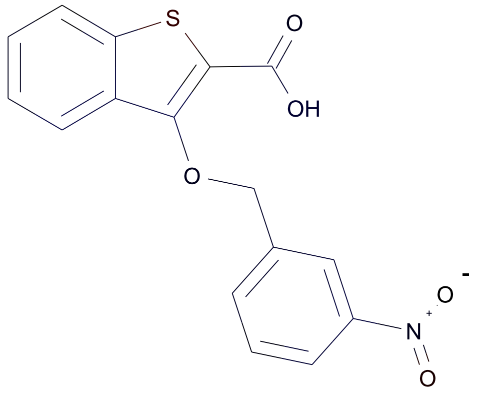

$C_{16}H_{11}NO_5S$
Molecular Weight: 329.32724
ALogP: 4.074
Rotatable Bonds: 5
Acceptors: 5
Donors: 1

Model Prediction

Prediction: Single-Carcinogen
Probability: 0.452
Enrichment: 1.208
Bayesian Score: -1.924
Mahalanobis Distance: 21.981
Mahalanobis Distance p-value: 9.24e-013

Prediction: Positive if the Bayesian score is above the estimated best cutoff value from minimizing the false positive and false negative rate.
Probability: The esimated probability that the sample is in the positive category. This assumes that the Bayesian score follows a normal distribution and is different from the prediction using a cutoff.
Enrichment: An estimate of enrichment, that is, the increased likelihood (versus random) of this sample being in the category.
Bayesian Score: The standard Laplacian-modified Bayesian score.
Mahalanobis Distance: The Mahalanobis distance (MD) is the distance to the center of the training data. The larger the MD, the less trustworthy the prediction.
Mahalanobis Distance p-value: The p-value gives the fraction of training data with an MD greater than or equal to the one for the given sample, assuming normally distributed data. The smaller the p-value, the less trustworthy the prediciton. For highly non-normal X properties (e.g., fingerprints), the MD p-value is wildly inaccurate.

Structural Similar Compounds

Name	Omeprazole	Dantrolene	Estrogens; conjug.
Structure	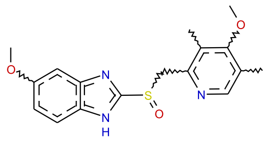	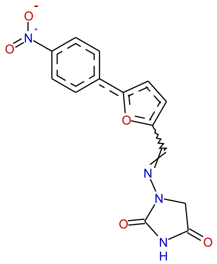	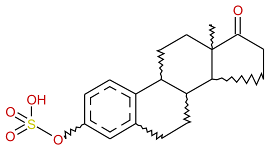
Actual Endpoint	Multiple-Carcinogen	Multiple-Carcinogen	Single-Carcinogen
Predicted Endpoint	Multiple-Carcinogen	Multiple-Carcinogen	Single-Carcinogen
Distance	0.628	0.646	0.660
Reference	US FDA (Centre for Drug Eval.& Res./Off. Testing & Res.) Sept. 1997	US FDA (Centre for Drug Eval.& Res./Off. Testing & Res.) Sept. 1997	US FDA (Centre for Drug Eval.& Res./Off. Testing & Res.) Sept. 1997

Model Applicability

Unknown features are fingerprint features in the query molecule, but not found in the training set.

1. All properties and OPS components are within expected ranges.

Feature Contribution

Top features for positive contribution

Fingerprint	Bit/Smiles	Feature Structure	Score	Multiple-Carcinogen in training set
SCFP_4	17	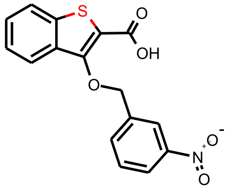 [*]:s:[*]	0.548	10 out of 17

SCFP_4	1311339974	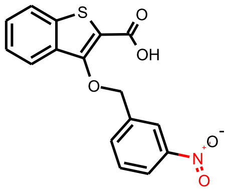 [*][N+](=O)[*]	0.504	9 out of 16
SCFP_4	1310748454	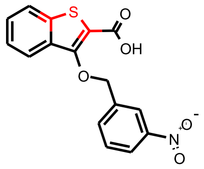 [*][c]1:[*]:[*]:[c](:[*]):s:1	0.444	4 out of 7
**Top Features for negative contribution**				
**Fingerprint**	**Bit/Smiles**	**Feature Structure**	**Score**	**Multiple-Carcinogen in training set**
SCFP_4	112346096	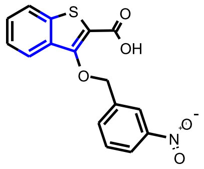 [*][c]1:[*]:[*]:[c](:[*]):[c]:1:[cH]:[*]	-0.730	1 out of 10
SCFP_4	1132967294	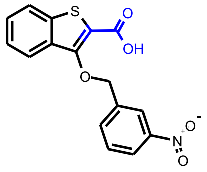 [*]:[c](:[*])C(=O)O	-0.666	0 out of 3
SCFP_4	-352263424	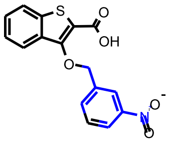 [*]C[c]1:[cH]:[*]:[cH]:[c](:[cH]:1)[N+](=[*])[*]	-0.489	0 out of 2

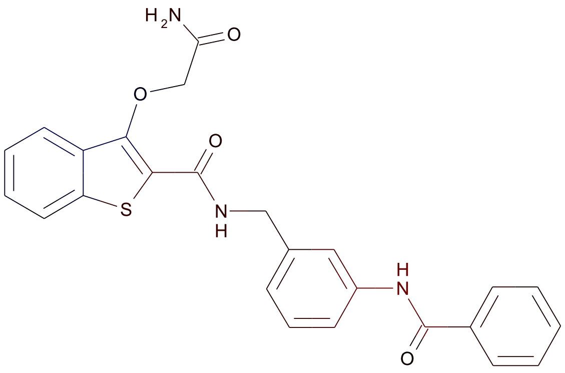

$C_{25}H_{21}N_{3}O_{4}S$
Molecular Weight: 459.51694
ALogP: 3.396
Rotatable Bonds: 8
Acceptors: 4
Donors: 3

Model Prediction

Prediction: Multiple-Carcinogen
Probability: 0.632
Enrichment: 1.690
Bayesian Score: 3.919
Mahalanobis Distance: 18.230
Mahalanobis Distance p-value: 2.44e-009

Prediction: Positive if the Bayesian score is above the estimated best cutoff value from minimizing the false positive and false negative rate.
Probability: The esimated probability that the sample is in the positive category. This assumes that the Bayesian score follows a normal distribution and is different from the prediction using a cutoff.
Enrichment: An estimate of enrichment, that is, the increased likelihood (versus random) of this sample being in the category.
Bayesian Score: The standard Laplacian-modified Bayesian score.
Mahalanobis Distance: The Mahalanobis distance (MD) is the distance to the center of the training data. The larger the MD, the less trustworthy the prediction.
Mahalanobis Distance p-value: The p-value gives the fraction of training data with an MD greater than or equal to the one for the given sample, assuming normally distributed data. The smaller the p-value, the less trustworthy the prediciton. For highly non-normal X properties (e.g., fingerprints), the MD p-value is wildly inaccurate.

Structural Similar Compounds

Name	Bicalutamide	Torsemide	Mesuprine
Structure	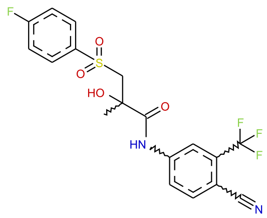	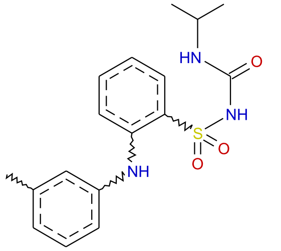	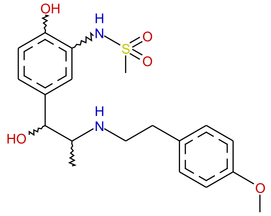
Actual Endpoint	Multiple-Carcinogen	Single-Carcinogen	Single-Carcinogen
Predicted Endpoint	Multiple-Carcinogen	Single-Carcinogen	Single-Carcinogen
Distance	0.654	0.692	0.754
Reference	US FDA (Centre for Drug Eval.& Res./Off. Testing & Res.) Sept. 1997	US FDA (Centre for Drug Eval.& Res./Off. Testing & Res.) Sept. 1997	US FDA (Centre for Drug Eval.& Res./Off. Testing & Res.) Sept. 1997

Model Applicability

Unknown features are fingerprint features in the query molecule, but not found in the training set.

1. All properties and OPS components are within expected ranges.

Feature Contribution

Top features for positive contribution

Fingerprint	Bit/Smiles	Feature Structure	Score	Multiple-Carcinogen in training set
SCFP_4	1631845520	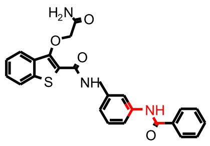 [*]C(=[*])N[c](:[*]):[*]	0.601	6 out of 9

SCFP_4	17	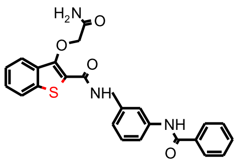 [*]:s:[*]	0.548	10 out of 17
SCFP_4	-1375926917	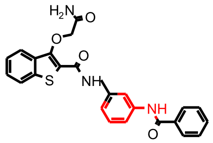 [*]N[c]1:[cH]:[*]:[cH]:[cH]:[cH]:1	0.522	6 out of 10
**Top Features for negative contribution**				
**Fingerprint**	**Bit/Smiles**	**Feature Structure**	**Score**	**Multiple-Carcinogen in training set**
SCFP_4	112346096	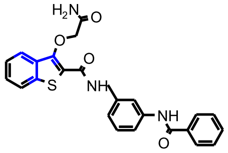 [*][c]1:[*]:[*]:[c](:[*]):[c]:1:[cH]:[*]	-0.730	1 out of 10
SCFP_4	-1379673609	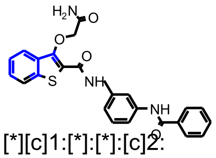 [*][c]1:[*]:[*]:[c]2:[*]:[cH]:[cH]:[cH]:[c]:1:2	-0.472	1 out of 7
SCFP_4	-711656408	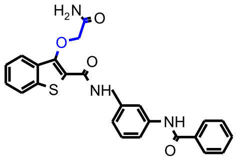 [*]OCC(=[*])[*]	-0.472	1 out of 7

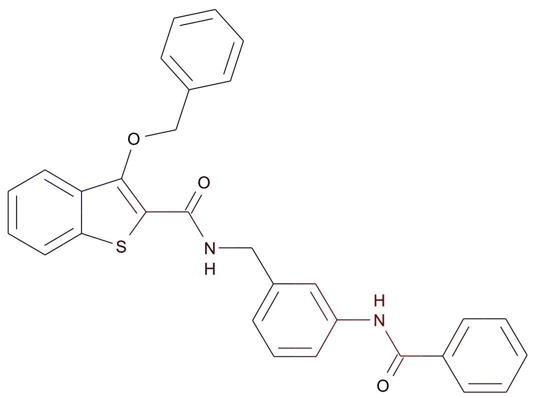

$C_{30}H_{24}N_2O_3S$
Molecular Weight: 492.58816
ALogP: 6.126
Rotatable Bonds: 8
Acceptors: 3
Donors: 2

Model Prediction

Prediction: Multiple-Carcinogen
Probability: 0.557
Enrichment: 1.490
Bayesian Score: 2.316
Mahalanobis Distance: 18.143
Mahalanobis Distance p-value: 2.95e-009

Prediction: Positive if the Bayesian score is above the estimated best cutoff value from minimizing the false positive and false negative rate.
Probability: The esimated probability that the sample is in the positive category. This assumes that the Bayesian score follows a normal distribution and is different from the prediction using a cutoff.
Enrichment: An estimate of enrichment, that is, the increased likelihood (versus random) of this sample being in the category.
Bayesian Score: The standard Laplacian-modified Bayesian score.
Mahalanobis Distance: The Mahalanobis distance (MD) is the distance to the center of the training data. The larger the MD, the less trustworthy the prediction.
Mahalanobis Distance p-value: The p-value gives the fraction of training data with an MD greater than or equal to the one for the given sample, assuming normally distributed data. The smaller the p-value, the less trustworthy the prediciton. For highly non-normal X properties (e.g., fingerprints), the MD p-value is wildly inaccurate.

Structural Similar Compounds

Name	Simvastatin	Bicalutamide	Lansoprazole
Structure	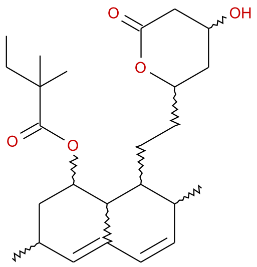	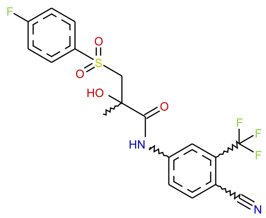	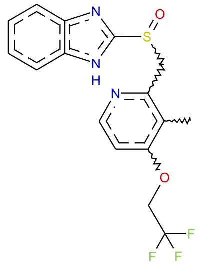
Actual Endpoint	Multiple-Carcinogen	Multiple-Carcinogen	Single-Carcinogen
Predicted Endpoint	Multiple-Carcinogen	Multiple-Carcinogen	Single-Carcinogen
Distance	0.804	0.811	0.845
Reference	US FDA (Centre for Drug Eval.& Res./Off. Testing & Res.) Sept. 1997	US FDA (Centre for Drug Eval.& Res./Off. Testing & Res.) Sept. 1997	US FDA (Centre for Drug Eval.& Res./Off. Testing & Res.) Sept. 1997

Model Applicability

Unknown features are fingerprint features in the query molecule, but not found in the training set.

1. All properties and OPS components are within expected ranges.

Feature Contribution

Top features for positive contribution

Fingerprint	Bit/Smiles	Feature Structure	Score	Multiple-Carcinogen in training set
SCFP_4	1631845520	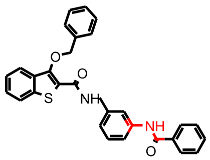 [*]C(=[*])N[c](:[*]):[*]	0.601	6 out of 9

SCFP_4	17	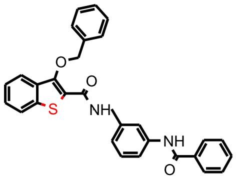 [*]:s:[*]	0.548	10 out of 17
SCFP_4	-1375926917	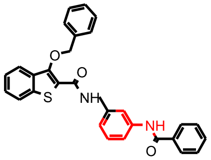 [*]N[c]1:[cH]:[*]:[cH]:[cH]:[cH]:1	0.522	6 out of 10
**Top Features for negative contribution**				
**Fingerprint**	**Bit/Smiles**	**Feature Structure**	**Score**	**Multiple-Carcinogen in training set**
SCFP_4	112346096	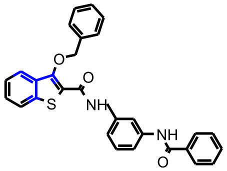 [*][c]1:[*]:[*]:[c](:[*]):[c]:1:[cH]:[*]	-0.730	1 out of 10
SCFP_4	-711596826	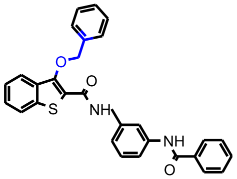 [*]OC[c](:[*]):[*]	-0.489	0 out of 2
SCFP_4	-1379673609	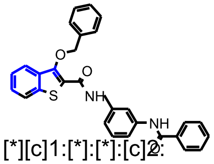 [*][c]1:[*]:[*]:[c]2: [*]:[cH]:[cH]:[cH]:[c]:1:2	-0.472	1 out of 7

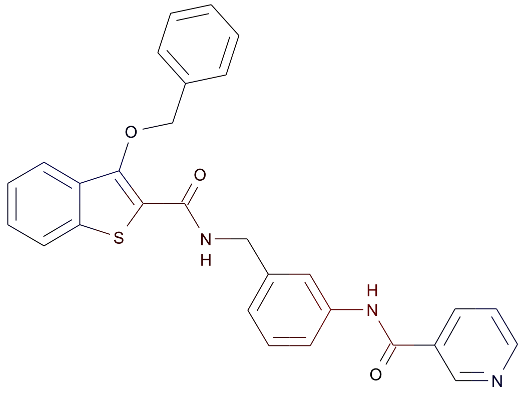

$C_{29}H_{23}N_3O_3S$
Molecular Weight: 493.57622
ALogP: 4.976
Rotatable Bonds: 8
Acceptors: 4
Donors: 2

Model Prediction

Prediction: Multiple-Carcinogen
Probability: 0.559
Enrichment: 1.494
Bayesian Score: 2.348
Mahalanobis Distance: 20.190
Mahalanobis Distance p-value: 3.7e-011

Prediction: Positive if the Bayesian score is above the estimated best cutoff value from minimizing the false positive and false negative rate.
Probability: The esimated probability that the sample is in the positive category. This assumes that the Bayesian score follows a normal distribution and is different from the prediction using a cutoff.
Enrichment: An estimate of enrichment, that is, the increased likelihood (versus random) of this sample being in the category.
Bayesian Score: The standard Laplacian-modified Bayesian score.
Mahalanobis Distance: The Mahalanobis distance (MD) is the distance to the center of the training data. The larger the MD, the less trustworthy the prediction.
Mahalanobis Distance p-value: The p-value gives the fraction of training data with an MD greater than or equal to the one for the given sample, assuming normally distributed data. The smaller the p-value, the less trustworthy the prediciton. For highly non-normal X properties (e.g., fingerprints), the MD p-value is wildly inaccurate.

Structural Similar Compounds

Name	Bicalutamide	Simvastatin	Lansoprazole
Structure	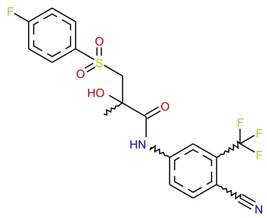	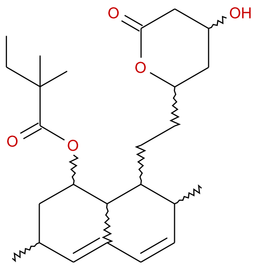	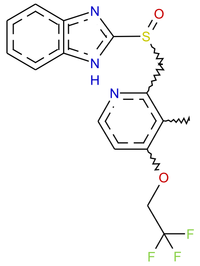
Actual Endpoint	Multiple-Carcinogen	Multiple-Carcinogen	Single-Carcinogen
Predicted Endpoint	Multiple-Carcinogen	Multiple-Carcinogen	Single-Carcinogen
Distance	0.693	0.735	0.752
Reference	US FDA (Centre for Drug Eval.& Res./Off. Testing & Res.) Sept. 1997	US FDA (Centre for Drug Eval.& Res./Off. Testing & Res.) Sept. 1997	US FDA (Centre for Drug Eval.& Res./Off. Testing & Res.) Sept. 1997

Model Applicability

Unknown features are fingerprint features in the query molecule, but not found in the training set.

1. All properties and OPS components are within expected ranges.

Feature Contribution

Top features for positive contribution

Fingerprint	Bit/Smiles	Feature Structure	Score	Multiple-Carcinogen in training set
SCFP_4	1631845520	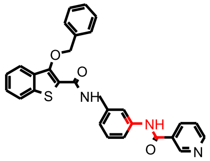 [*]C(=[*])N[c](:[*]):[*]	0.601	6 out of 9

SCFP_4	17	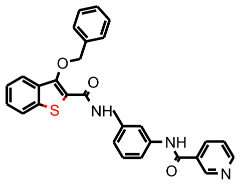 [*]:s:[*]	0.548	10 out of 17
SCFP_4	-1375926917	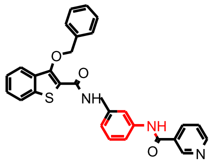 [*]N[c]1:[cH]:[*]:[cH<br>]:[cH]:[cH]:1	0.522	6 out of 10

Top Features for negative contribution

Fingerprint	Bit/Smiles	Feature Structure	Score	Multiple-Carcinogen in training set
SCFP_4	112346096	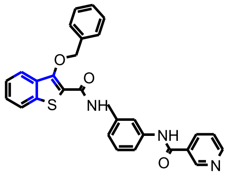 [*][c]1:[*]:[*]:[c](:<br>[*]):[c]:1:[cH]:[*]	-0.730	1 out of 10
SCFP_4	1188429584	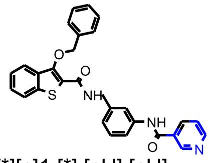 [*][c]1:[*]:[cH]:[cH]<br>:n:[cH]:1	-0.666	0 out of 3
SCFP_4	-758850909	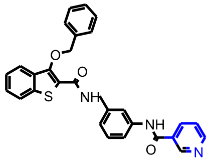 [*][c]1:[*]:n:[cH]:[c<br>H]:[cH]:1	-0.489	0 out of 2

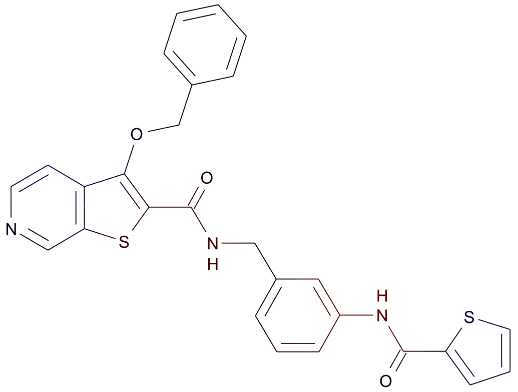

$C_{27}H_{21}N_3O_3S_2$
Molecular Weight: 499.60394
ALogP: 4.929
Rotatable Bonds: 8
Acceptors: 4
Donors: 2

Model Prediction

Prediction: Multiple-Carcinogen
Probability: 0.539
Enrichment: 1.442
Bayesian Score: 1.938
Mahalanobis Distance: 18.704
Mahalanobis Distance p-value: 8.76e-010

Prediction: Positive if the Bayesian score is above the estimated best cutoff value from minimizing the false positive and false negative rate.
Probability: The esimated probability that the sample is in the positive category. This assumes that the Bayesian score follows a normal distribution and is different from the prediction using a cutoff.
Enrichment: An estimate of enrichment, that is, the increased likelihood (versus random) of this sample being in the category.
Bayesian Score: The standard Laplacian-modified Bayesian score.
Mahalanobis Distance: The Mahalanobis distance (MD) is the distance to the center of the training data. The larger the MD, the less trustworthy the prediction.
Mahalanobis Distance p-value: The p-value gives the fraction of training data with an MD greater than or equal to the one for the given sample, assuming normally distributed data. The smaller the p-value, the less trustworthy the prediciton. For highly non-normal X properties (e.g., fingerprints), the MD p-value is wildly inaccurate.

Structural Similar Compounds

Name	Bicalutamide	Lansoprazole	Simvastatin
Structure	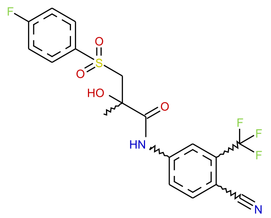	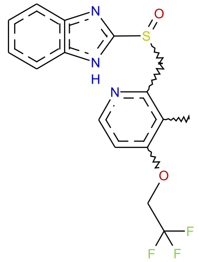	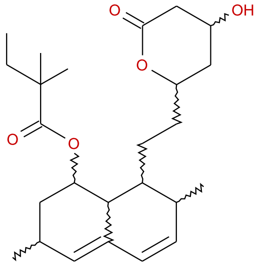
Actual Endpoint	Multiple-Carcinogen	Single-Carcinogen	Multiple-Carcinogen
Predicted Endpoint	Multiple-Carcinogen	Single-Carcinogen	Multiple-Carcinogen
Distance	0.687	0.761	0.770
Reference	US FDA (Centre for Drug Eval.& Res./Off. Testing & Res.) Sept. 1997	US FDA (Centre for Drug Eval.& Res./Off. Testing & Res.) Sept. 1997	US FDA (Centre for Drug Eval.& Res./Off. Testing & Res.) Sept. 1997

Model Applicability

Unknown features are fingerprint features in the query molecule, but not found in the training set.

1. All properties and OPS components are within expected ranges.

Feature Contribution

Top features for positive contribution

Fingerprint	Bit/Smiles	Feature Structure	Score	Multiple-Carcinogen in training set
SCFP_4	1631845520	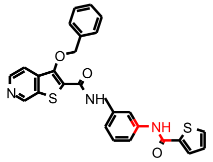 [*]C(=[*])N[c](:[*]):[*]	0.601	6 out of 9

SCFP_4	17	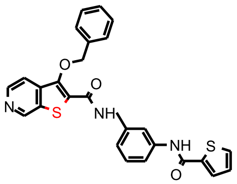 [*]:s:[*]	0.548	10 out of 17
SCFP_4	-1375926917	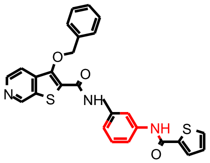 [*]N[c]1:[cH]:[*]:[cH]:[cH]:[cH]:1	0.522	6 out of 10
**Top Features for negative contribution**				
**Fingerprint**	**Bit/Smiles**	**Feature Structure**	**Score**	**Multiple-Carcinogen in training set**
SCFP_4	112346096	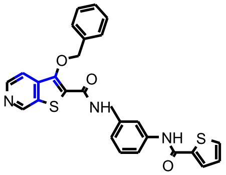 [*][c]1:[*]:[*]:[c](: [*]):[c]:1:[cH]:[*]	-0.730	1 out of 10
SCFP_4	1188429584	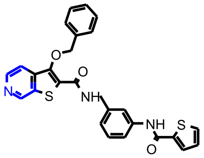 [*][c]1:[*]:[cH]:[cH] :n:[cH]:1	-0.666	0 out of 3
SCFP_4	-711596826	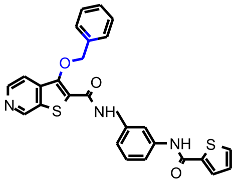 [*]OC[c](:[*]):[*]	-0.489	0 out of 2

Co-crystallized ligand

TOPKAT_Rat_Female_FDA_Single_vs_Multiple

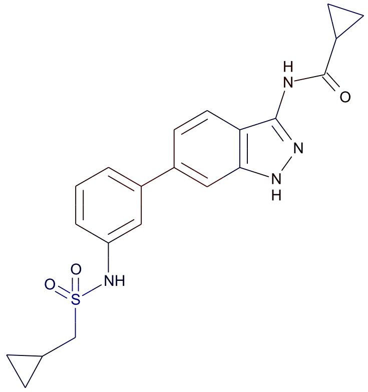

$C_{21}H_{22}N_4O_3S$
Molecular Weight: 410.48938
ALogP: 3.14
Rotatable Bonds: 7
Acceptors: 4
Donors: 3

Structural Similar Compounds

Name	Torsemide	Bicalutamide	Ursodiol
Structure	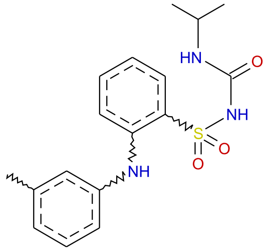	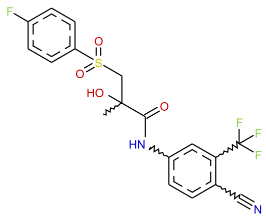	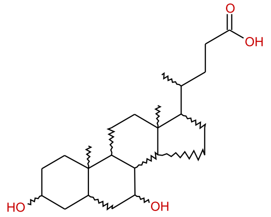
Actual Endpoint	Single-Carcinogen	Multiple-Carcinogen	Single-Carcinogen
Predicted Endpoint	Single-Carcinogen	Multiple-Carcinogen	Single-Carcinogen
Distance	0.597	0.630	0.690
Reference	US FDA (Centre for Drug Eval.& Res./Off. Testing & Res.) Sept. 1997	US FDA (Centre for Drug Eval.& Res./Off. Testing & Res.) Sept. 1997	US FDA (Centre for Drug Eval.& Res./Off. Testing & Res.) Sept. 1997

Model Prediction

Prediction: Single-Carcinogen
Probability: 0.425
Enrichment: 1.135
Bayesian Score: -2.602
Mahalanobis Distance: 13.183
Mahalanobis Distance p-value: 0.000154

Prediction: Positive if the Bayesian score is above the estimated best cutoff value from minimizing the false positive and false negative rate.
Probability: The esimated probability that the sample is in the positive category. This assumes that the Bayesian score follows a normal distribution and is different from the prediction using a cutoff.
Enrichment: An estimate of enrichment, that is, the increased likelihood (versus random) of this sample being in the category.
Bayesian Score: The standard Laplacian-modified Bayesian score.
Mahalanobis Distance: The Mahalanobis distance (MD) is the distance to the center of the training data. The larger the MD, the less trustworthy the prediction.
Mahalanobis Distance p-value: The p-value gives the fraction of training data with an MD greater than or equal to the one for the given sample, assuming normally distributed data. The smaller the p-value, the less trustworthy the prediciton. For highly non-normal X properties (e.g., fingerprints), the MD p-value is wildly inaccurate.

Model Applicability

Unknown features are fingerprint features in the query molecule, but not found in the training set.

1. All properties and OPS components are within expected ranges.

Feature Contribution

Top features for positive contribution

Fingerprint	Bit/Smiles	Feature Structure	Score	Multiple-Carcinogen in training set
SCFP_4	667776369	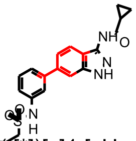 [*]:[c](:[*])[c]1:[cH]:[*]:[c](:[*]):[cH]:[cH]:1	0.610	2 out of 2

SCFP_4	1631845520	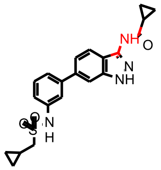 [*]C(=[*])N[c](:[*]): [*]	0.601	6 out of 9
SCFP_4	-1375926917	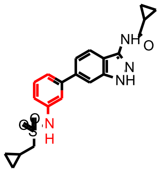 [*]N[c]1:[cH]:[*]:[cH]:[cH]:[cH]:1	0.522	6 out of 10

Top Features for negative contribution

Fingerprint	Bit/Smiles	Feature Structure	Score	Multiple-Carcinogen in training set
SCFP_4	1135377188	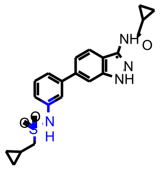 [*]S(=[*])(=[*])N[c](:[*]):[*]	-0.816	0 out of 4
SCFP_4	112346096	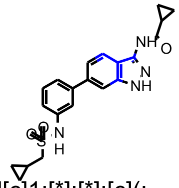 [*][c]1:[*]:[*]:[c](: [*]):[c]:1:[cH]:[*]	-0.730	1 out of 10
SCFP_4	-1043339860	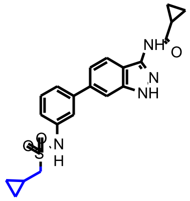 [*]CC1CC1	-0.675	4 out of 28

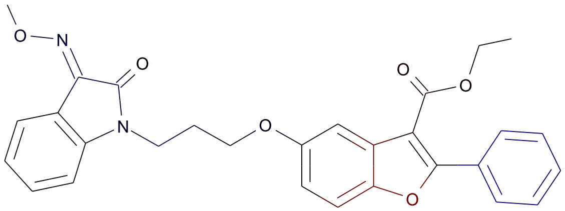

$C_{29}H_{26}N_2O_6$
Molecular Weight: 498.52653
ALogP: 4.994
Rotatable Bonds: 10
Acceptors: 6
Donors: 0

Model Prediction

Prediction: Non-Carcinogen
Probability: 0.280
Enrichment: 0.837
Bayesian Score: -2.985
Mahalanobis Distance: 17.222
Mahalanobis Distance p-value: 8.92e-012

Prediction: Positive if the Bayesian score is above the estimated best cutoff value from minimizing the false positive and false negative rate.
Probability: The esimated probability that the sample is in the positive category. This assumes that the Bayesian score follows a normal distribution and is different from the prediction using a cutoff.
Enrichment: An estimate of enrichment, that is, the increased likelihood (versus random) of this sample being in the category.
Bayesian Score: The standard Laplacian-modified Bayesian score.
Mahalanobis Distance: The Mahalanobis distance (MD) is the distance to the center of the training data. The larger the MD, the less trustworthy the prediction.
Mahalanobis Distance p-value: The p-value gives the fraction of training data with an MD greater than or equal to the one for the given sample, assuming normally distributed data. The smaller the p-value, the less trustworthy the prediciton. For highly non-normal X properties (e.g., fingerprints), the MD p-value is wildly inaccurate.

Structural Similar Compounds

Name	Nefazodone	Verapamil	Ketoconazole
Structure	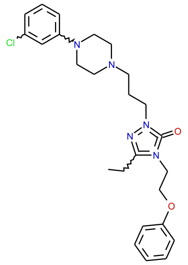	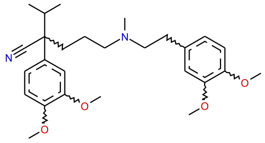	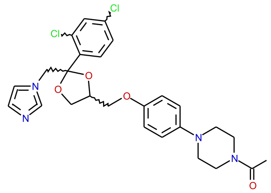
Actual Endpoint	Non-Carcinogen	Non-Carcinogen	Non-Carcinogen
Predicted Endpoint	Non-Carcinogen	Non-Carcinogen	Non-Carcinogen
Distance	0.582	0.682	0.691
Reference	US FDA (Centre for Drug Eval.& Res./Off. Testing & Res.) Sept. 1997	US FDA (Centre for Drug Eval.& Res./Off. Testing & Res.) Sept. 1997	US FDA (Centre for Drug Eval.& Res./Off. Testing & Res.) Sept. 1997

Model Applicability

Unknown features are fingerprint features in the query molecule, but not found in the training set.

1. All properties and OPS components are within expected ranges.

Feature Contribution

Top features for positive contribution

Fingerprint	Bit/Smiles	Feature Structure	Score	Carcinogen in training set
SCFP_6	112644006	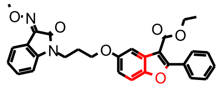 [*]:[cH]:[c]1:o:[*]:[*]:[c]:1:[*]	0.712	3 out of 3

SCFP_6	794417578	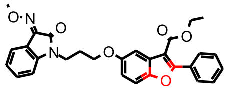 [*][c]1:[*]:[*]:[c](:[*]):o:1	0.600	9 out of 14
SCFP_6	-768812299	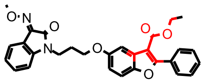 [*]COC(=O)[c]1:[c]([*]):[*]:[*]:[c]:1:[*]	0.429	3 out of 5

Top Features for negative contribution

Fingerprint	Bit/Smiles	Feature Structure	Score	Carcinogen in training set
SCFP_6	1655112089	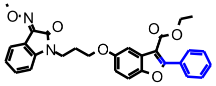 [*]:[c](:[*])[c]1:[cH]:[cH]:[cH]:[cH]:[cH]:1	-0.674	0 out of 3
SCFP_6	1653911926	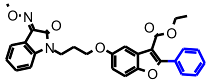 [*][c]1:[cH]:[cH]:[cH]:[cH]:[cH]:1	-0.504	12 out of 64
SCFP_6	1683883894	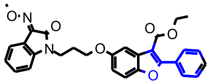 [*][c]1:[*]:[*]:o:[c]:1[c]2:[cH]:[cH]:[*]:[cH]:[cH]:2	-0.496	0 out of 2

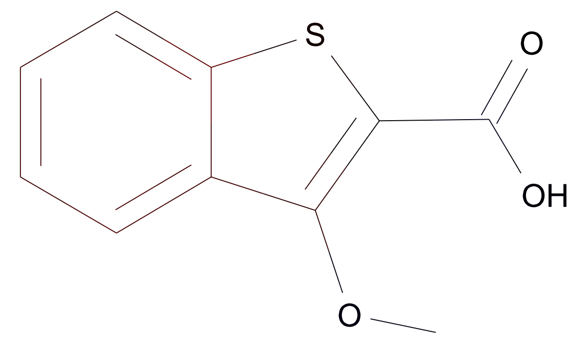

$C_{10}H_8O_3S$
Molecular Weight: 208.23372
ALogP: 2.596
Rotatable Bonds: 2
Acceptors: 3
Donors: 1

Model Prediction

Prediction: Carcinogen
Probability: 0.387
Enrichment: 1.157
Bayesian Score: 1.019
Mahalanobis Distance: 12.954
Mahalanobis Distance p-value: 0.00186

Prediction: Positive if the Bayesian score is above the estimated best cutoff value from minimizing the false positive and false negative rate.
Probability: The esimated probability that the sample is in the positive category. This assumes that the Bayesian score follows a normal distribution and is different from the prediction using a cutoff.
Enrichment: An estimate of enrichment, that is, the increased likelihood (versus random) of this sample being in the category.
Bayesian Score: The standard Laplacian-modified Bayesian score.
Mahalanobis Distance: The Mahalanobis distance (MD) is the distance to the center of the training data. The larger the MD, the less trustworthy the prediction.
Mahalanobis Distance p-value: The p-value gives the fraction of training data with an MD greater than or equal to the one for the given sample, assuming normally distributed data. The smaller the p-value, the less trustworthy the prediciton. For highly non-normal X properties (e.g., fingerprints), the MD p-value is wildly inaccurate.

Structural Similar Compounds

Name	Thiabendazole	Aspirin	Suprofen
Structure	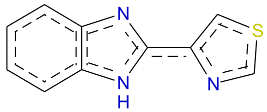	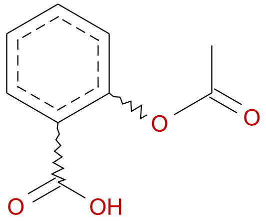	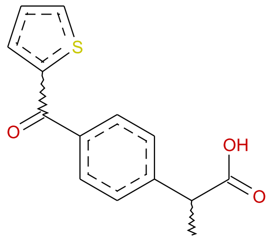
Actual Endpoint	Carcinogen	Non-Carcinogen	Non-Carcinogen
Predicted Endpoint	Carcinogen	Non-Carcinogen	Non-Carcinogen
Distance	0.574	0.575	0.593
Reference	US FDA (Centre for Drug Eval.& Res./Off. Testing & Res.) Sept. 1997	US FDA (Centre for Drug Eval.& Res./Off. Testing & Res.) Sept. 1997	US FDA (Centre for Drug Eval.& Res./Off. Testing & Res.) Sept. 1997

Model Applicability

Unknown features are fingerprint features in the query molecule, but not found in the training set.

1. All properties and OPS components are within expected ranges.

Feature Contribution

Top features for positive contribution

Fingerprint	Bit/Smiles	Feature Structure	Score	Carcinogen in training set
SCFP_6	1651620003	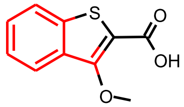 [*][c]1:[*]:[*]:[c]2:[cH]:[cH]:[cH]:[cH]:[c]:1:2	0.643	7 out of 10

SCFP_6	-1379673609	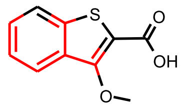 [*][c]1:[*]:[*]:[c]2:[*]:[cH]:[cH]:[cH]:[c]:1:2	0.526	11 out of 19
SCFP_6	1310748454	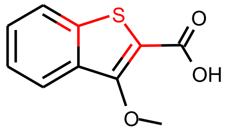 [*][c]1:[*]:[*]:[c](:[*]):s:1	0.437	7 out of 13
**Top Features for negative contribution**				
**Fingerprint**	**Bit/Smiles**	**Feature Structure**	**Score**	**Carcinogen in training set**
SCFP_6	932433556	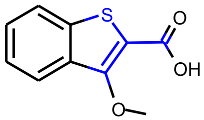 [*]C(=[*])[c]1:s:[*]:[*]:[c]:1[*]	-0.278	0 out of 1
SCFP_6	-424485343	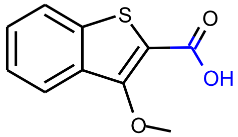 [*]C(=[*])O	-0.208	17 out of 66
SCFP_6	591469355	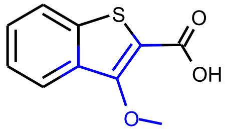 [*][c]1:[*]:[*]:[c](:[*]):[c]:1OC	-0.135	8 out of 29

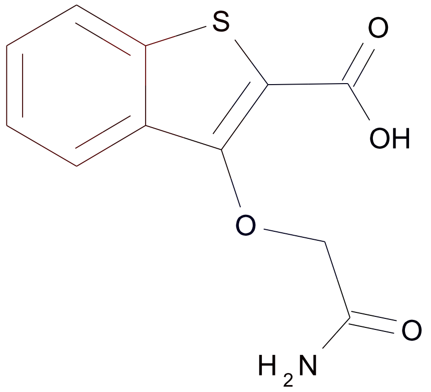

$C_{11}H_9NO_4S$
Molecular Weight: 251.25846
ALogP: 1.45
Rotatable Bonds: 4
Acceptors: 4
Donors: 2

Model Prediction

Prediction: Carcinogen
Probability: 0.406
Enrichment: 1.215
Bayesian Score: 1.626
Mahalanobis Distance: 14.661
Mahalanobis Distance p-value: 2.77e-006

Prediction: Positive if the Bayesian score is above the estimated best cutoff value from minimizing the false positive and false negative rate.
Probability: The esimated probability that the sample is in the positive category. This assumes that the Bayesian score follows a normal distribution and is different from the prediction using a cutoff.
Enrichment: An estimate of enrichment, that is, the increased likelihood (versus random) of this sample being in the category.
Bayesian Score: The standard Laplacian-modified Bayesian score.
Mahalanobis Distance: The Mahalanobis distance (MD) is the distance to the center of the training data. The larger the MD, the less trustworthy the prediction.
Mahalanobis Distance p-value: The p-value gives the fraction of training data with an MD greater than or equal to the one for the given sample, assuming normally distributed data. The smaller the p-value, the less trustworthy the prediciton. For highly non-normal X properties (e.g., fingerprints), the MD p-value is wildly inaccurate.

Structural Similar Compounds

Name	Nithiazide	Sulfamethoxazole	Captopril
Structure	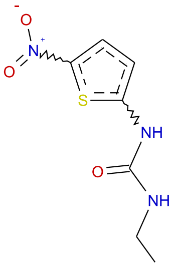	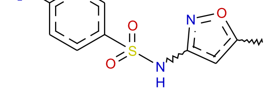	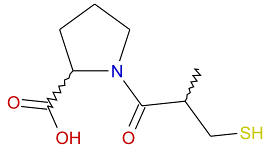
Actual Endpoint	Non-Carcinogen	Carcinogen	Non-Carcinogen
Predicted Endpoint	Non-Carcinogen	Carcinogen	Non-Carcinogen
Distance	0.571	0.579	0.585
Reference	US FDA (Centre for Drug Eval.& Res./Off. Testing & Res.) Sept. 1997	US FDA (Centre for Drug Eval.& Res./Off. Testing & Res.) Sept. 1997	US FDA (Centre for Drug Eval.& Res./Off. Testing & Res.) Sept. 1997

Model Applicability

Unknown features are fingerprint features in the query molecule, but not found in the training set.

1. All properties and OPS components are within expected ranges.

Feature Contribution

Top features for positive contribution

Fingerprint	Bit/Smiles	Feature Structure	Score	Carcinogen in training set
SCFP_6	1651620003	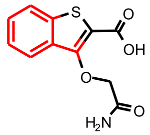 [*][c]1:[*]:[*]:[c]2:[cH]:[cH]:[cH]:[cH]:[c]:1:2	0.643	7 out of 10

SCFP_6	-1379673609	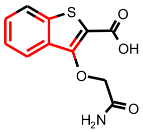<br>[*][c]1:[*]:[*]:[c]2:<br>[*]:[cH]:[cH]:[cH]:[<br>c]:1:2	0.526	11 out of 19
SCFP_6	-1357949052	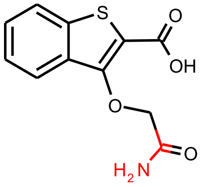<br>[*]C(=[*])N	0.494	8 out of 14

Top Features for negative contribution

Fingerprint	Bit/Smiles	Feature Structure	Score	Carcinogen in training set
SCFP_6	1983013828	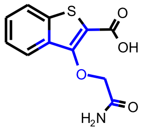<br>[*]C(=[*])CO[c]1:[c](<br>[*]):[*]:[*]:[c]:1:[<br>*]	-0.496	0 out of 2
SCFP_6	932433556	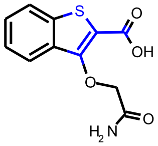<br>[*]C(=[*])[c]1:s:[*]:<br>[*]:[c]:1[*]	-0.278	0 out of 1
SCFP_6	-100452175	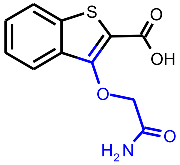<br>[*]:[c](:[*])OCC(=O)N	-0.278	0 out of 1

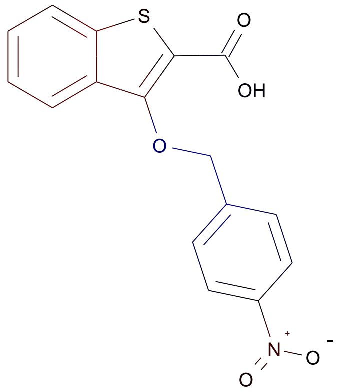

$C_{16}H_{11}NO_5S$
Molecular Weight: 329.32724
ALogP: 4.074
Rotatable Bonds: 5
Acceptors: 5
Donors: 1

Model Prediction

Prediction: Non-Carcinogen
Probability: 0.339
Enrichment: 1.016
Bayesian Score: -0.600
Mahalanobis Distance: 18.107
Mahalanobis Distance p-value: 6.5e-014

Prediction: Positive if the Bayesian score is above the estimated best cutoff value from minimizing the false positive and false negative rate.
Probability: The esimated probability that the sample is in the positive category. This assumes that the Bayesian score follows a normal distribution and is different from the prediction using a cutoff.
Enrichment: An estimate of enrichment, that is, the increased likelihood (versus random) of this sample being in the category.
Bayesian Score: The standard Laplacian-modified Bayesian score.
Mahalanobis Distance: The Mahalanobis distance (MD) is the distance to the center of the training data. The larger the MD, the less trustworthy the prediction.
Mahalanobis Distance p-value: The p-value gives the fraction of training data with an MD greater than or equal to the one for the given sample, assuming normally distributed data. The smaller the p-value, the less trustworthy the prediciton. For highly non-normal X properties (e.g., fingerprints), the MD p-value is wildly inaccurate.

Structural Similar Compounds

Name	Omeprazole	Niclosamide	Suprofen
Structure	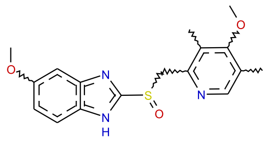	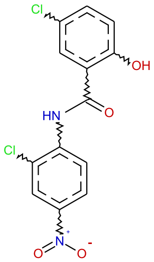	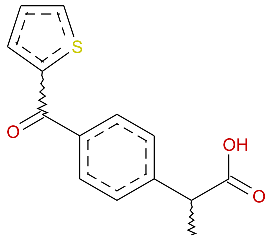
Actual Endpoint	Carcinogen	Non-Carcinogen	Non-Carcinogen
Predicted Endpoint	Carcinogen	Non-Carcinogen	Non-Carcinogen
Distance	0.655	0.666	0.669
Reference	US FDA (Centre for Drug Eval.& Res./Off. Testing & Res.) Sept. 1997	US FDA (Centre for Drug Eval.& Res./Off. Testing & Res.) Sept. 1997	US FDA (Centre for Drug Eval.& Res./Off. Testing & Res.) Sept. 1997

Model Applicability

Unknown features are fingerprint features in the query molecule, but not found in the training set.

1. All properties and OPS components are within expected ranges.

Feature Contribution

Top features for positive contribution

Fingerprint	Bit/Smiles	Feature Structure	Score	Carcinogen in training set
SCFP_6	1651620003	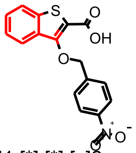 [*][c]1:[*]:[*]:[c]2:[cH]:[cH]:[cH]:[cH]:[c]:1:2	0.643	7 out of 10

SCFP_6	-1379673609	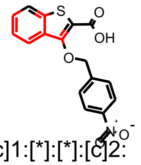 [*][c]1:[*]:[*]:[c]2:[*]:[cH]:[cH]:[cH]:[c]:1:2	0.526	11 out of 19
SCFP_6	1310748454	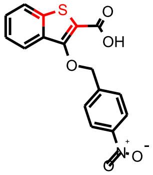 [*][c]1:[*]:[*]:[c](:[*]):s:1	0.437	7 out of 13
**Top Features for negative contribution**				
**Fingerprint**	**Bit/Smiles**	**Feature Structure**	**Score**	**Carcinogen in training set**
SCFP_6	-711596826	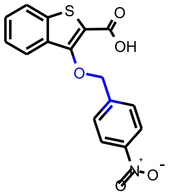 [*]OC[c](:[*]):[*]	-1.177	0 out of 7
SCFP_6	-1216061692	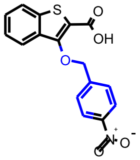 [*]OC[c]1:[cH]:[cH]:[*]:[cH]:[cH]:1	-0.825	0 out of 4
SCFP_6	932433556	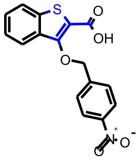 [*]C(=[*])[c]1:s:[*]:[*]:[c]:1[*]	-0.278	0 out of 1

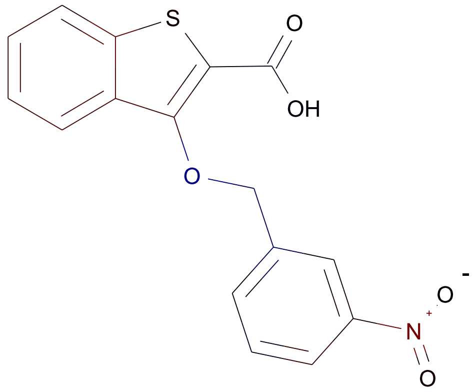

$C_{16}H_{11}NO_5S$
Molecular Weight: 329.32724
ALogP: 4.074
Rotatable Bonds: 5
Acceptors: 5
Donors: 1

Model Prediction

Prediction: Carcinogen
Probability: 0.363
Enrichment: 1.085
Bayesian Score: 0.220
Mahalanobis Distance: 18.107
Mahalanobis Distance p-value: 6.5e-014

Prediction: Positive if the Bayesian score is above the estimated best cutoff value from minimizing the false positive and false negative rate.
Probability: The esimated probability that the sample is in the positive category. This assumes that the Bayesian score follows a normal distribution and is different from the prediction using a cutoff.
Enrichment: An estimate of enrichment, that is, the increased likelihood (versus random) of this sample being in the category.
Bayesian Score: The standard Laplacian-modified Bayesian score.
Mahalanobis Distance: The Mahalanobis distance (MD) is the distance to the center of the training data. The larger the MD, the less trustworthy the prediction.
Mahalanobis Distance p-value: The p-value gives the fraction of training data with an MD greater than or equal to the one for the given sample, assuming normally distributed data. The smaller the p-value, the less trustworthy the prediciton. For highly non-normal X properties (e.g., fingerprints), the MD p-value is wildly inaccurate.

Structural Similar Compounds

Name	Omeprazole	Niclosamide	Suprofen
Structure	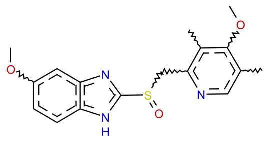	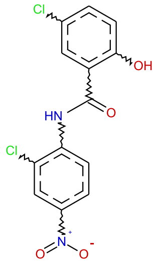	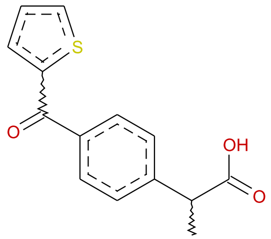
Actual Endpoint	Carcinogen	Non-Carcinogen	Non-Carcinogen
Predicted Endpoint	Carcinogen	Non-Carcinogen	Non-Carcinogen
Distance	0.657	0.667	0.671
Reference	US FDA (Centre for Drug Eval.& Res./Off. Testing & Res.) Sept. 1997	US FDA (Centre for Drug Eval.& Res./Off. Testing & Res.) Sept. 1997	US FDA (Centre for Drug Eval.& Res./Off. Testing & Res.) Sept. 1997

Model Applicability

Unknown features are fingerprint features in the query molecule, but not found in the training set.

1. All properties and OPS components are within expected ranges.

Feature Contribution

Top features for positive contribution				
**Fingerprint**	**Bit/Smiles**	**Feature Structure**	**Score**	**Carcinogen in training set**
SCFP_6	-352263424	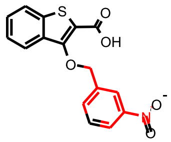 [*]C[c]1:[cH]:[*]:[cH]:[c](:[cH]:1)[N+](=[*])[*]	0.712	3 out of 3

SCFP_6	1651620003	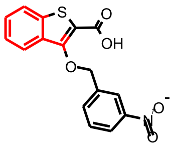 [*][c]1:[*]:[*]:[c]2:[cH]:[cH]:[cH]:[cH]:[c]:1:2	0.643	7 out of 10
SCFP_6	-1630708879	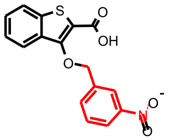 [*]C[c]1:[cH]:[cH]:[cH]:[c](:[cH]:1)[N+](=[*])[*]	0.603	2 out of 2

Top Features for negative contribution

Fingerprint	Bit/Smiles	Feature Structure	Score	Carcinogen in training set
SCFP_6	-711596826	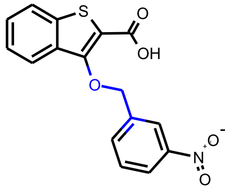 [*]OC[c](:[*]):[*]	-1.177	0 out of 7
SCFP_6	-1216061692	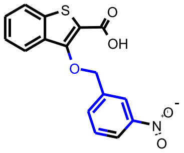 [*]OC[c]1:[cH]:[cH]:[*]:[cH]:[cH]:1	-0.825	0 out of 4
SCFP_6	-365284206	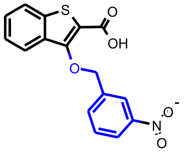 [*]OC[c]1:[cH]:[cH]:[cH]:[c]([*]):[cH]:1	-0.496	0 out of 2

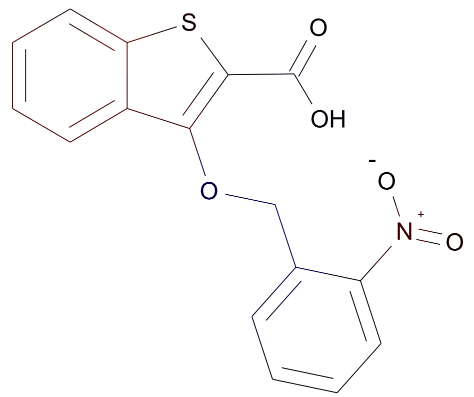

$C_{16}H_{11}NO_5S$
Molecular Weight: 329.32724
ALogP: 4.074
Rotatable Bonds: 5
Acceptors: 5
Donors: 1

Model Prediction

Prediction: Carcinogen
Probability: 0.362
Enrichment: 1.083
Bayesian Score: 0.192
Mahalanobis Distance: 18.494
Mahalanobis Distance p-value: 7.05e-015

Prediction: Positive if the Bayesian score is above the estimated best cutoff value from minimizing the false positive and false negative rate.
Probability: The esimated probability that the sample is in the positive category. This assumes that the Bayesian score follows a normal distribution and is different from the prediction using a cutoff.
Enrichment: An estimate of enrichment, that is, the increased likelihood (versus random) of this sample being in the category.
Bayesian Score: The standard Laplacian-modified Bayesian score.
Mahalanobis Distance: The Mahalanobis distance (MD) is the distance to the center of the training data. The larger the MD, the less trustworthy the prediction.
Mahalanobis Distance p-value: The p-value gives the fraction of training data with an MD greater than or equal to the one for the given sample, assuming normally distributed data. The smaller the p-value, the less trustworthy the prediciton. For highly non-normal X properties (e.g., fingerprints), the MD p-value is wildly inaccurate.

Structural Similar Compounds

Name	Omeprazole	Niclosamide	Suprofen
Structure	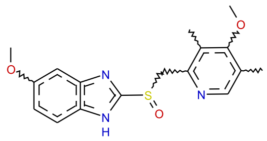	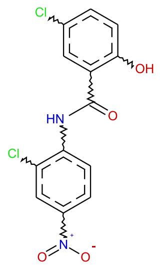	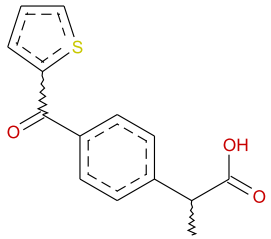
Actual Endpoint	Carcinogen	Non-Carcinogen	Non-Carcinogen
Predicted Endpoint	Carcinogen	Non-Carcinogen	Non-Carcinogen
Distance	0.656	0.667	0.671
Reference	US FDA (Centre for Drug Eval.& Res./Off. Testing & Res.) Sept. 1997	US FDA (Centre for Drug Eval.& Res./Off. Testing & Res.) Sept. 1997	US FDA (Centre for Drug Eval.& Res./Off. Testing & Res.) Sept. 1997

Model Applicability

Unknown features are fingerprint features in the query molecule, but not found in the training set.

1. All properties and OPS components are within expected ranges.

Feature Contribution

Top features for positive contribution				
Fingerprint	Bit/Smiles	Feature Structure	Score	Carcinogen in training set
SCFP_6	1651620003	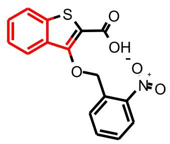 [*][c]1:[*]:[*]:[c]2:[cH]:[cH]:[cH]:[cH]:[c]:1:2	0.643	7 out of 10

SCFP_6	-1379673609	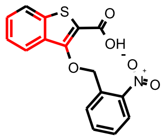 [*][c]1:[*]:[*]:[c]2:[*]:[cH]:[cH]:[cH]:[c]:1:2	0.526	11 out of 19
SCFP_6	1310748454	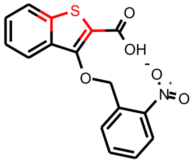 [*][c]1:[*]:[*]:[c](:[*]):s:1	0.437	7 out of 13
**Top Features for negative contribution**				
**Fingerprint**	**Bit/Smiles**	**Feature Structure**	**Score**	**Carcinogen in training set**
SCFP_6	-711596826	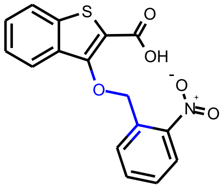 [*]OC[c](:[*]):[*]	-1.177	0 out of 7
SCFP_6	932433556	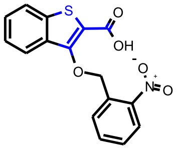 [*]C(=[*])[c]1:s:[*]:[*]:[c]:1[*]	-0.278	0 out of 1
SCFP_6	-1379148975	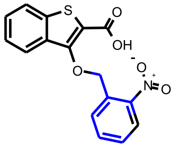 [*]C[c]1:[cH]:[*]:[c]([*]):[cH]:[cH]:1	-0.257	34 out of 138

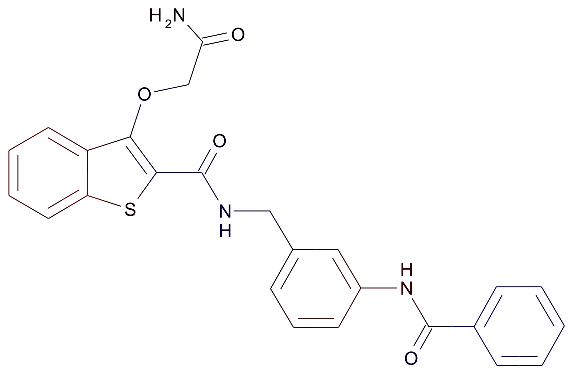

$C_{25}H_{21}N_3O_4S$
Molecular Weight: 459.51694
ALogP: 3.396
Rotatable Bonds: 8
Acceptors: 4
Donors: 3

Structural Similar Compounds

Name	Glyburide	Glimepride	Fluvastatin
Structure	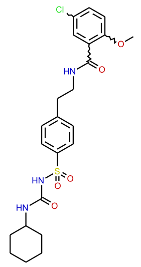	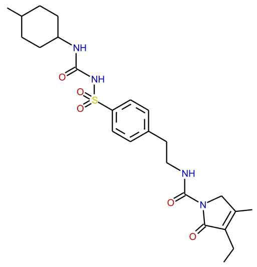	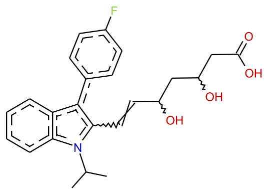
Actual Endpoint	Non-Carcinogen	Non-Carcinogen	Carcinogen
Predicted Endpoint	Non-Carcinogen	Non-Carcinogen	Carcinogen
Distance	0.587	0.600	0.645
Reference	US FDA (Centre for Drug Eval.& Res./Off. Testing & Res.) Sept. 1997	US FDA (Centre for Drug Eval.& Res./Off. Testing & Res.) Sept. 1997	US FDA (Centre for Drug Eval.& Res./Off. Testing & Res.) Sept. 1997

Model Prediction

Prediction: Non-Carcinogen
Probability: 0.339
Enrichment: 1.013
Bayesian Score: -0.631
Mahalanobis Distance: 18.503
Mahalanobis Distance p-value: 6.71e-015

Prediction: Positive if the Bayesian score is above the estimated best cutoff value from minimizing the false positive and false negative rate.
Probability: The esimated probability that the sample is in the positive category. This assumes that the Bayesian score follows a normal distribution and is different from the prediction using a cutoff.
Enrichment: An estimate of enrichment, that is, the increased likelihood (versus random) of this sample being in the category.
Bayesian Score: The standard Laplacian-modified Bayesian score.
Mahalanobis Distance: The Mahalanobis distance (MD) is the distance to the center of the training data. The larger the MD, the less trustworthy the prediction.
Mahalanobis Distance p-value: The p-value gives the fraction of training data with an MD greater than or equal to the one for the given sample, assuming normally distributed data. The smaller the p-value, the less trustworthy the prediciton. For highly non-normal X properties (e.g., fingerprints), the MD p-value is wildly inaccurate.

Model Applicability

Unknown features are fingerprint features in the query molecule, but not found in the training set.

1. All properties and OPS components are within expected ranges.

Feature Contribution

Top features for positive contribution

Fingerprint	Bit/Smiles	Feature Structure	Score	Carcinogen in training set
SCFP_6	1651620003	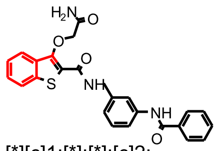 [*][c]1:[*]:[*]:[c]2:[cH]:[cH]:[cH]:[cH]:[c]:1:2	0.643	7 out of 10

SCFP_6	-347048986	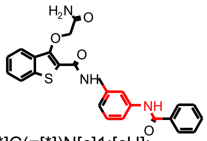 [*]C(=[*])N[c]1:[cH]:[cH]:[*]:[c]([*]):[cH]:1	0.615	5 out of 7
SCFP_6	-1379673609	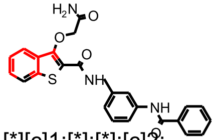 [*][c]1:[*]:[*]:[c]2:[*]:[cH]:[cH]:[cH]:[c]:1:2	0.526	11 out of 19

Top Features for negative contribution

Fingerprint	Bit/Smiles	Feature Structure	Score	Carcinogen in training set
SCFP_6	1653911926	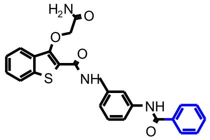 [*][c]1:[cH]:[cH]:[cH]:[cH]:[cH]:1	-0.504	12 out of 64
SCFP_6	1983013828	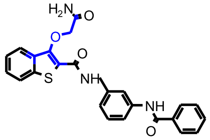 [*]C(=[*])CO[c]1:[c]([*]):[*]:[*]:[c]:1:[*]	-0.496	0 out of 2
SCFP_6	1257084377	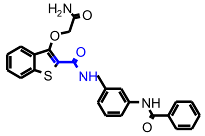 [*]NC(=O)[c](:[*]):[*]	-0.436	4 out of 21

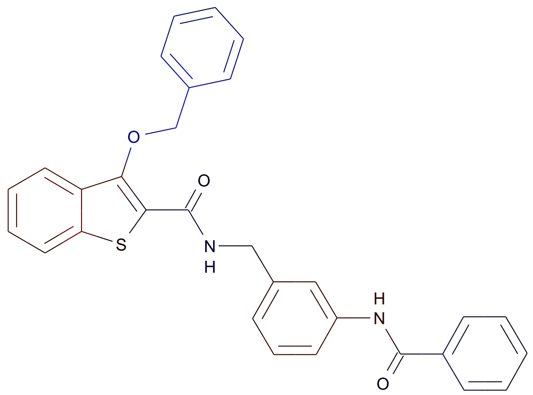

$C_{30}H_{24}N_2O_3S$
Molecular Weight: 492.58816
ALogP: 6.126
Rotatable Bonds: 8
Acceptors: 3
Donors: 2

Model Prediction

Prediction: Non-Carcinogen
Probability: 0.287
Enrichment: 0.860
Bayesian Score: -2.650
Mahalanobis Distance: 17.917
Mahalanobis Distance p-value: 1.91e-013

Prediction: Positive if the Bayesian score is above the estimated best cutoff value from minimizing the false positive and false negative rate.
Probability: The esimated probability that the sample is in the positive category. This assumes that the Bayesian score follows a normal distribution and is different from the prediction using a cutoff.
Enrichment: An estimate of enrichment, that is, the increased likelihood (versus random) of this sample being in the category.
Bayesian Score: The standard Laplacian-modified Bayesian score.
Mahalanobis Distance: The Mahalanobis distance (MD) is the distance to the center of the training data. The larger the MD, the less trustworthy the prediction.
Mahalanobis Distance p-value: The p-value gives the fraction of training data with an MD greater than or equal to the one for the given sample, assuming normally distributed data. The smaller the p-value, the less trustworthy the prediciton. For highly non-normal X properties (e.g., fingerprints), the MD p-value is wildly inaccurate.

Structural Similar Compounds

Name	Terfenadine	Pimozide	Astemizole
Structure	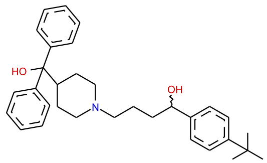	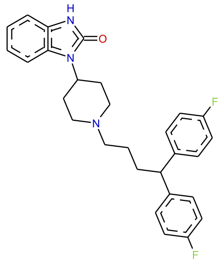	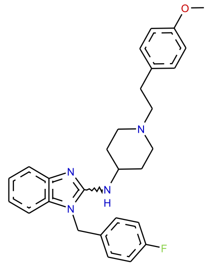
Actual Endpoint	Non-Carcinogen	Non-Carcinogen	Non-Carcinogen
Predicted Endpoint	Non-Carcinogen	Non-Carcinogen	Non-Carcinogen
Distance	0.628	0.693	0.696
Reference	US FDA (Centre for Drug Eval.& Res./Off. Testing & Res.) Sept. 1997	US FDA (Centre for Drug Eval.& Res./Off. Testing & Res.) Sept. 1997	US FDA (Centre for Drug Eval.& Res./Off. Testing & Res.) Sept. 1997

Model Applicability

Unknown features are fingerprint features in the query molecule, but not found in the training set.

1. All properties and OPS components are within expected ranges.

Feature Contribution

Top features for positive contribution

Fingerprint	Bit/Smiles	Feature Structure	Score	Carcinogen in training set
SCFP_6	1651620003	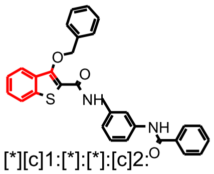 [*][c]1:[*]:[*]:[c]2:[cH]:[cH]:[cH]:[cH]:[c]:1:2	0.643	7 out of 10

SCFP_6	-347048986	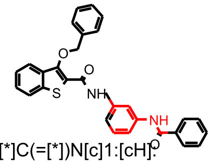 [*]C(=[*])N[c]1:[cH]:[cH]:[*]:[c]([*]):[cH]:1	0.615	5 out of 7
SCFP_6	-1379673609	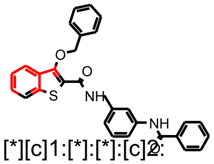 [*][c]1:[*]:[*]:[c]2:[*]:[cH]:[cH]:[cH]:[c]:1:2	0.526	11 out of 19
**Top Features for negative contribution**				
**Fingerprint**	**Bit/Smiles**	**Feature Structure**	**Score**	**Carcinogen in training set**
SCFP_6	-711596826	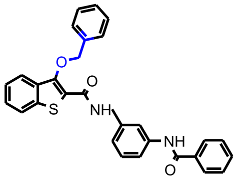 [*]OC[c](:[*]):[*]	-1.177	0 out of 7
SCFP_6	-1216061692	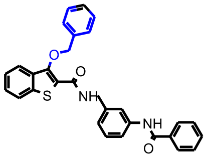 [*]OC[c]1:[cH]:[cH]:[*]:[cH]:[cH]:1	-0.825	0 out of 4
SCFP_6	1653911926	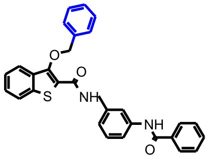 [*][c]1:[cH]:[cH]:[cH]:[cH]:[cH]:1	-0.504	12 out of 64

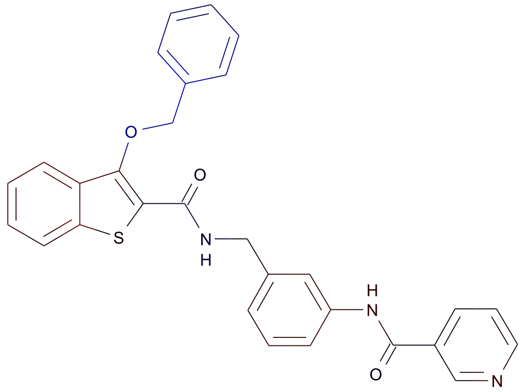

$C_{29}H_{23}N_{3}O_{3}S$
Molecular Weight: 493.57622
ALogP: 4.976
Rotatable Bonds: 8
Acceptors: 4
Donors: 2

Model Prediction

Prediction: Non-Carcinogen
Probability: 0.315
Enrichment: 0.941
Bayesian Score: -1.538
Mahalanobis Distance: 19.380
Mahalanobis Distance p-value: 3.86e-017

Prediction: Positive if the Bayesian score is above the estimated best cutoff value from minimizing the false positive and false negative rate.
Probability: The esimated probability that the sample is in the positive category. This assumes that the Bayesian score follows a normal distribution and is different from the prediction using a cutoff.
Enrichment: An estimate of enrichment, that is, the increased likelihood (versus random) of this sample being in the category.
Bayesian Score: The standard Laplacian-modified Bayesian score.
Mahalanobis Distance: The Mahalanobis distance (MD) is the distance to the center of the training data. The larger the MD, the less trustworthy the prediction.
Mahalanobis Distance p-value: The p-value gives the fraction of training data with an MD greater than or equal to the one for the given sample, assuming normally distributed data. The smaller the p-value, the less trustworthy the prediciton. For highly non-normal X properties (e.g., fingerprints), the MD p-value is wildly inaccurate.

Structural Similar Compounds

Name	Glyburide	Flecainide	Fluvastatin
Structure	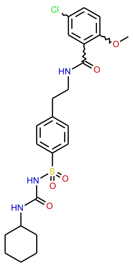	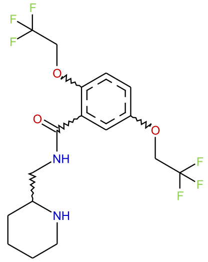	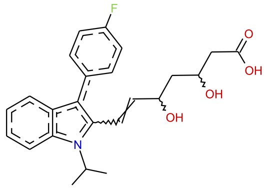
Actual Endpoint	Non-Carcinogen	Non-Carcinogen	Carcinogen
Predicted Endpoint	Non-Carcinogen	Non-Carcinogen	Carcinogen
Distance	0.632	0.672	0.673
Reference	US FDA (Centre for Drug Eval.& Res./Off. Testing & Res.) Sept. 1997	US FDA (Centre for Drug Eval.& Res./Off. Testing & Res.) Sept. 1997	US FDA (Centre for Drug Eval.& Res./Off. Testing & Res.) Sept. 1997

Model Applicability

Unknown features are fingerprint features in the query molecule, but not found in the training set.

1. All properties and OPS components are within expected ranges.

Feature Contribution

Top features for positive contribution

Fingerprint	Bit/Smiles	Feature Structure	Score	Carcinogen in training set
SCFP_6	1651620003	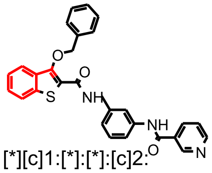 [*][c]1:[*]:[*]:[c]2:[cH]:[cH]:[cH]:[cH]:[c]:1:2	0.643	7 out of 10

SCFP_6	-347048986	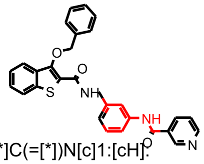<br>[*]C(=[*])N[c]1:[cH]:<br>[cH]:[*]:[c]([*]):[c<br>H]:1	0.615	5 out of 7
SCFP_6	-1379673609	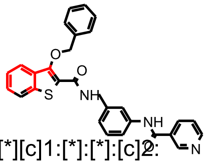<br>[*][c]1:[*]:[*]:[c]2:<br>[*]:[cH]:[cH]:[cH]:[<br>c]:1:2	0.526	11 out of 19
**Top Features for negative contribution**				
**Fingerprint**	**Bit/Smiles**	**Feature Structure**	**Score**	**Carcinogen in training set**
SCFP_6	-711596826	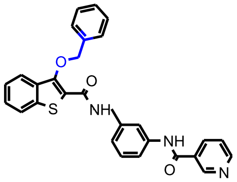<br>[*]OC[c](:[*]):[*]	-1.177	0 out of 7
SCFP_6	-1216061692	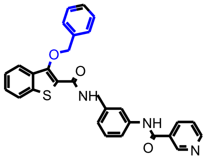<br>[*]OC[c]1:[cH]:[cH]:[<br>*]:[cH]:[cH]:1	-0.825	0 out of 4
SCFP_6	1653911926	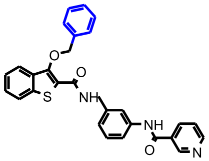<br>[*][c]1:[cH]:[cH]:[cH<br>]:[cH]:[cH]:1	-0.504	12 out of 64

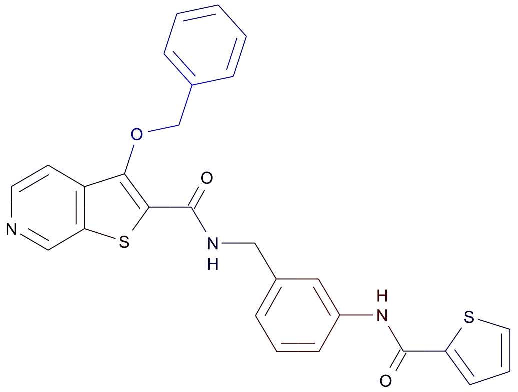

$C_{27}H_{21}N_3O_3S_2$
Molecular Weight: 499.60394
ALogP: 4.929
Rotatable Bonds: 8
Acceptors: 4
Donors: 2

Model Prediction

Prediction: Non-Carcinogen
Probability: 0.252
Enrichment: 0.753
Bayesian Score: -4.325
Mahalanobis Distance: 18.987
Mahalanobis Distance p-value: 3.98e-016

Prediction: Positive if the Bayesian score is above the estimated best cutoff value from minimizing the false positive and false negative rate.
Probability: The esimated probability that the sample is in the positive category. This assumes that the Bayesian score follows a normal distribution and is different from the prediction using a cutoff.
Enrichment: An estimate of enrichment, that is, the increased likelihood (versus random) of this sample being in the category.
Bayesian Score: The standard Laplacian-modified Bayesian score.
Mahalanobis Distance: The Mahalanobis distance (MD) is the distance to the center of the training data. The larger the MD, the less trustworthy the prediction.
Mahalanobis Distance p-value: The p-value gives the fraction of training data with an MD greater than or equal to the one for the given sample, assuming normally distributed data. The smaller the p-value, the less trustworthy the prediciton. For highly non-normal X properties (e.g., fingerprints), the MD p-value is wildly inaccurate.

Structural Similar Compounds

Name	Glyburide	Glimepride	Fluvastatin
Structure	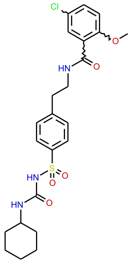	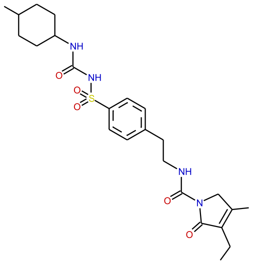	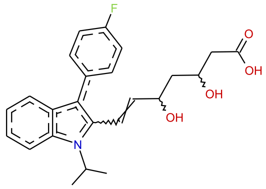
Actual Endpoint	Non-Carcinogen	Non-Carcinogen	Carcinogen
Predicted Endpoint	Non-Carcinogen	Non-Carcinogen	Carcinogen
Distance	0.635	0.673	0.712
Reference	US FDA (Centre for Drug Eval.& Res./Off. Testing & Res.) Sept. 1997	US FDA (Centre for Drug Eval.& Res./Off. Testing & Res.) Sept. 1997	US FDA (Centre for Drug Eval.& Res./Off. Testing & Res.) Sept. 1997

Model Applicability

Unknown features are fingerprint features in the query molecule, but not found in the training set.

1. OPS PC24 out of range. Value: -3.302. Training min, max, SD, explained variance: -3.2733, 3.1662, 1.103, 0.0113.

Feature Contribution

Top features for positive contribution				
**Fingerprint**	**Bit/Smiles**	**Feature Structure**	**Score**	**Carcinogen in training set**
SCFP_6	-347048986	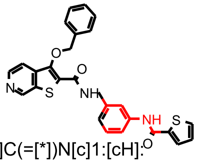 [*]C(=[*])N[c]1:[cH]:[cH]:[*]:[c]([*]):[cH]:1	0.615	5 out of 7

SCFP_6	1310748454	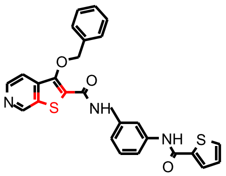 [*][c]1:[*]:[*]:[c](:[*]):s:1	0.437	7 out of 13
SCFP_6	112346096	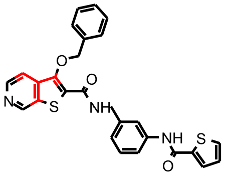 [*][c]1:[*]:[*]:[c](:[*]):[c]:1:[cH]:[*]	0.276	13 out of 30

Top Features for negative contribution

Fingerprint	Bit/Smiles	Feature Structure	Score	Carcinogen in training set
SCFP_6	-711596826	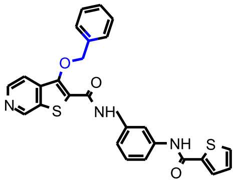 [*]OC[c](:[*]):[*]	-1.177	0 out of 7
SCFP_6	-1216061692	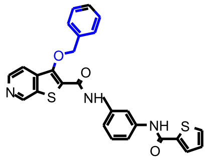 [*]OC[c]1:[cH]:[cH]:[*]:[cH]:[cH]:1	-0.825	0 out of 4
SCFP_6	1653911926	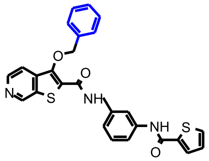 [*][c]1:[cH]:[cH]:[cH]:[cH]:[cH]:1	-0.504	12 out of 64

Co-crystallized ligand

TOPKAT_Rat_Male_FDA_None_vs_Carcinogen

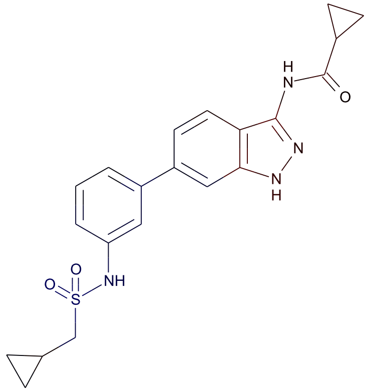

$C_{21}H_{22}N_4O_3S$
Molecular Weight: 410.48938
ALogP: 3.14
Rotatable Bonds: 7
Acceptors: 4
Donors: 3

Structural Similar Compounds

Name	Fluvastatin	Bicalutamide	Torsemide
Structure	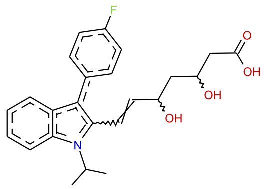	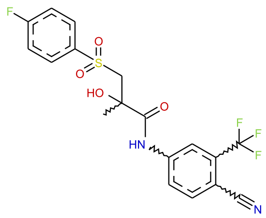	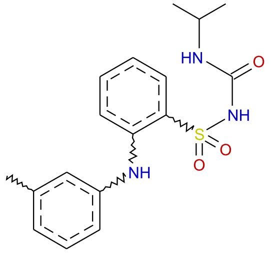
Actual Endpoint	Carcinogen	Carcinogen	Carcinogen
Predicted Endpoint	Carcinogen	Carcinogen	Carcinogen
Distance	0.621	0.621	0.625
Reference	US FDA (Centre for Drug Eval.& Res./Off. Testing & Res.) Sept. 1997	US FDA (Centre for Drug Eval.& Res./Off. Testing & Res.) Sept. 1997	US FDA (Centre for Drug Eval.& Res./Off. Testing & Res.) Sept. 1997

Model Prediction

Prediction: Carcinogen
Probability: 0.350
Enrichment: 1.048
Bayesian Score: -0.211
Mahalanobis Distance: 17.135
Mahalanobis Distance p-value: 1.43e-011

Prediction: Positive if the Bayesian score is above the estimated best cutoff value from minimizing the false positive and false negative rate.
Probability: The esimated probability that the sample is in the positive category. This assumes that the Bayesian score follows a normal distribution and is different from the prediction using a cutoff.
Enrichment: An estimate of enrichment, that is, the increased likelihood (versus random) of this sample being in the category.
Bayesian Score: The standard Laplacian-modified Bayesian score.
Mahalanobis Distance: The Mahalanobis distance (MD) is the distance to the center of the training data. The larger the MD, the less trustworthy the prediction.
Mahalanobis Distance p-value: The p-value gives the fraction of training data with an MD greater than or equal to the one for the given sample, assuming normally distributed data. The smaller the p-value, the less trustworthy the prediciton. For highly non-normal X properties (e.g., fingerprints), the MD p-value is wildly inaccurate.

Model Applicability

Unknown features are fingerprint features in the query molecule, but not found in the training set.

1. All properties and OPS components are within expected ranges.

Feature Contribution

Top features for positive contribution

Fingerprint	Bit/Smiles	Feature Structure	Score	Carcinogen in training set
SCFP_6	149212520	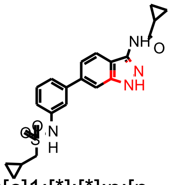 [*]:[c]1:[*]:[*]:n:[nH]:1	0.543	9 out of 15

SCFP_6	-1379673609	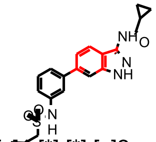<br>[*][c[illegible]:[*]:[*]:[c]2:[*]:[cH]:[cH]:[cH]:[c]:1:2	0.526	11 out of 19
SCFP_6	-26456656	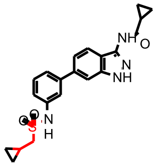<br>[*]S(=[*])(=[*])CC1[*][*]1	0.415	1 out of 1
**Top Features for negative contribution**				
**Fingerprint**	**Bit/Smiles**	**Feature Structure**	**Score**	**Carcinogen in training set**
SCFP_6	182902497	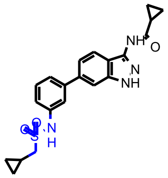<br>[*]CS(=O)(=O)N[*]	-0.825	0 out of 4
SCFP_6	149168366	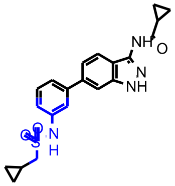<br>[*]CS(=O)(=O)N[c](:[cH]:[*]):[cH]:[*]	-0.674	0 out of 3
SCFP_6	21	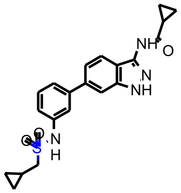<br>[*]S(=[*])(=[*])[*]	-0.283	7 out of 30

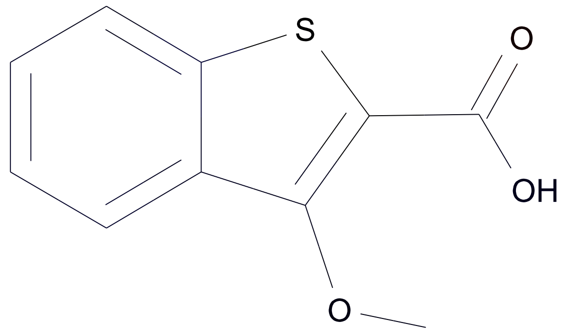

$C_{10}H_8O_3S$
Molecular Weight: 208.23372
ALogP: 2.596
Rotatable Bonds: 2
Acceptors: 3
Donors: 1

Model Prediction

Prediction: Single-Carcinogen
Probability: 0.538
Enrichment: 1.299
Bayesian Score: -1.861
Mahalanobis Distance: 15.909
Mahalanobis Distance p-value: 1.6e-006

Prediction: Positive if the Bayesian score is above the estimated best cutoff value from minimizing the false positive and false negative rate.
Probability: The esimated probability that the sample is in the positive category. This assumes that the Bayesian score follows a normal distribution and is different from the prediction using a cutoff.
Enrichment: An estimate of enrichment, that is, the increased likelihood (versus random) of this sample being in the category.
Bayesian Score: The standard Laplacian-modified Bayesian score.
Mahalanobis Distance: The Mahalanobis distance (MD) is the distance to the center of the training data. The larger the MD, the less trustworthy the prediction.
Mahalanobis Distance p-value: The p-value gives the fraction of training data with an MD greater than or equal to the one for the given sample, assuming normally distributed data. The smaller the p-value, the less trustworthy the prediciton. For highly non-normal X properties (e.g., fingerprints), the MD p-value is wildly inaccurate.

Structural Similar Compounds

Name	Thiabendazole	Cytembena	Flutamide
Structure	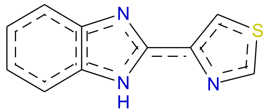	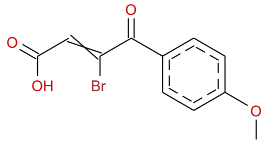	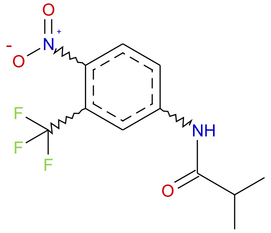
Actual Endpoint	Single-Carcinogen	Single-Carcinogen	Multiple-Carcinogen
Predicted Endpoint	Single-Carcinogen	Single-Carcinogen	Multiple-Carcinogen
Distance	0.586	0.661	0.678
Reference	US FDA (Centre for Drug Eval.& Res./Off. Testing & Res.) Sept. 1997	US FDA (Centre for Drug Eval.& Res./Off. Testing & Res.) Sept. 1997	US FDA (Centre for Drug Eval.& Res./Off. Testing & Res.) Sept. 1997

Model Applicability

Unknown features are fingerprint features in the query molecule, but not found in the training set.

1. All properties and OPS components are within expected ranges.

Feature Contribution

Top features for positive contribution

Fingerprint	Bit/Smiles	Feature Structure	Score	Multiple-Carcinogen in training set
SCFP_8	560173167	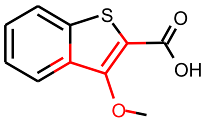 [*]O[c]1:[c]([*]):[*]:[*]:[c]:1:[*]	0.202	14 out of 31

SCFP_8	13	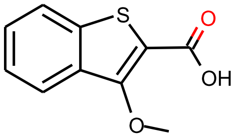<br>[*]=O	0.172	39 out of 90
SCFP_8	1	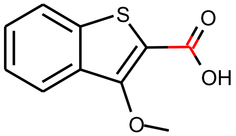<br>[*]C(=[*])[*]	0.146	38 out of 90
**Top Features for negative contribution**				
**Fingerprint**	**Bit/Smiles**	**Feature Structure**	**Score**	**Multiple-Carcinogen in training set**
SCFP_8	-424485343	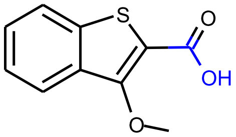<br>[*]C(=[*])O	-0.584	3 out of 17
SCFP_8	136239834	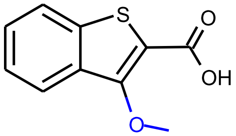<br>[*]OC	-0.358	3 out of 13
SCFP_8	-1379591900	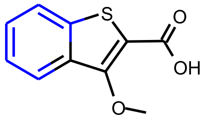<br>[*]:[c]1:[*]:[cH]:[cH]:[cH]:[cH]:1	-0.275	13 out of 48

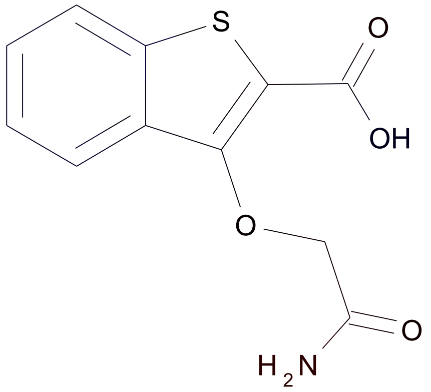

$C_{11}H_9NO_4S$
Molecular Weight: 251.25846
ALogP: 1.45
Rotatable Bonds: 4
Acceptors: 4
Donors: 2

Model Prediction

Prediction: Multiple-Carcinogen
Probability: 0.568
Enrichment: 1.372
Bayesian Score: 0.787
Mahalanobis Distance: 16.291
Mahalanobis Distance p-value: 7.11e-007

Prediction: Positive if the Bayesian score is above the estimated best cutoff value from minimizing the false positive and false negative rate.
Probability: The esimated probability that the sample is in the positive category. This assumes that the Bayesian score follows a normal distribution and is different from the prediction using a cutoff.
Enrichment: An estimate of enrichment, that is, the increased likelihood (versus random) of this sample being in the category.
Bayesian Score: The standard Laplacian-modified Bayesian score.
Mahalanobis Distance: The Mahalanobis distance (MD) is the distance to the center of the training data. The larger the MD, the less trustworthy the prediction.
Mahalanobis Distance p-value: The p-value gives the fraction of training data with an MD greater than or equal to the one for the given sample, assuming normally distributed data. The smaller the p-value, the less trustworthy the prediciton. For highly non-normal X properties (e.g., fingerprints), the MD p-value is wildly inaccurate.

Structural Similar Compounds

Name	Sulfamethoxazole	Furothiazole	Felbamate
Structure	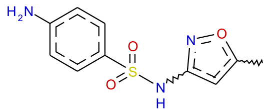	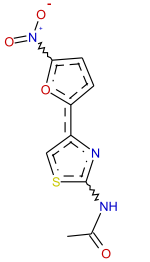	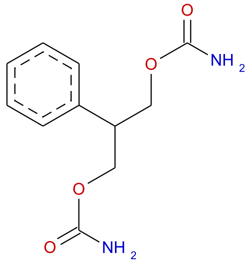
Actual Endpoint	Single-Carcinogen	Single-Carcinogen	Single-Carcinogen
Predicted Endpoint	Single-Carcinogen	Single-Carcinogen	Single-Carcinogen
Distance	0.588	0.644	0.652
Reference	US FDA (Centre for Drug Eval.& Res./Off. Testing & Res.) Sept. 1997	US FDA (Centre for Drug Eval.& Res./Off. Testing & Res.) Sept. 1997	US FDA (Centre for Drug Eval.& Res./Off. Testing & Res.) Sept. 1997

Model Applicability

Unknown features are fingerprint features in the query molecule, but not found in the training set.

1. All properties and OPS components are within expected ranges.

Feature Contribution

Top features for positive contribution

Fingerprint	Bit/Smiles	Feature Structure	Score	Multiple-Carcinogen in training set
SCFP_8	1256995004	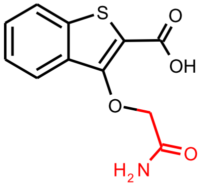 [*]CC(=O)N	0.331	10 out of 19

SCFP_8	9	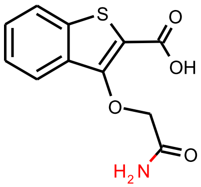 [*]N	0.253	19 out of 40
SCFP_8	-1357949052	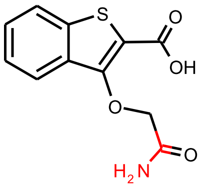 [*]C(=[*])N	0.247	4 out of 8

Top Features for negative contribution

**Fingerprint**	**Bit/Smiles**	**Feature Structure**	**Score**	**Multiple-Carcinogen in training set**
SCFP_8	-424485343	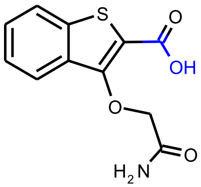 [*]C(=[*])O	-0.584	3 out of 17
SCFP_8	-1379591900	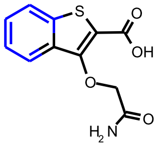 [*]:[c]1:[*]:[cH]:[cH]:[cH]:[cH]:1	-0.275	13 out of 48
SCFP_8	-1379673609	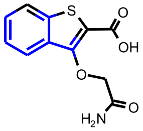 [*][c]1:[*]:[*]:[c]2:[*]:[cH]:[cH]:[cH]:[c]:1:2	-0.222	3 out of 11

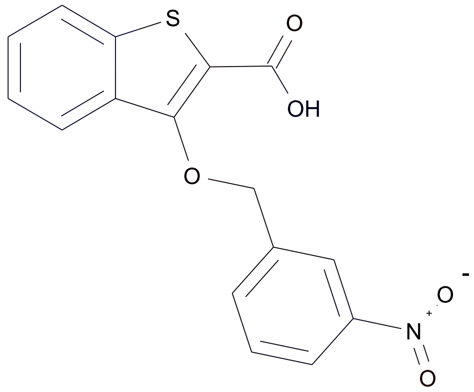

$C_{16}H_{11}NO_5S$
Molecular Weight: 329.32724
ALogP: 4.074
Rotatable Bonds: 5
Acceptors: 5
Donors: 1

Model Prediction

Prediction: Single-Carcinogen
Probability: 0.555
Enrichment: 1.341
Bayesian Score: -0.785
Mahalanobis Distance: 17.198
Mahalanobis Distance p-value: 1.04e-007

Prediction: Positive if the Bayesian score is above the estimated best cutoff value from minimizing the false positive and false negative rate.
Probability: The esimated probability that the sample is in the positive category. This assumes that the Bayesian score follows a normal distribution and is different from the prediction using a cutoff.
Enrichment: An estimate of enrichment, that is, the increased likelihood (versus random) of this sample being in the category.
Bayesian Score: The standard Laplacian-modified Bayesian score.
Mahalanobis Distance: The Mahalanobis distance (MD) is the distance to the center of the training data. The larger the MD, the less trustworthy the prediction.
Mahalanobis Distance p-value: The p-value gives the fraction of training data with an MD greater than or equal to the one for the given sample, assuming normally distributed data. The smaller the p-value, the less trustworthy the prediciton. For highly non-normal X properties (e.g., fingerprints), the MD p-value is wildly inaccurate.

Structural Similar Compounds

Name	Omeprazole	Lansoprazole	Cytembena
Structure	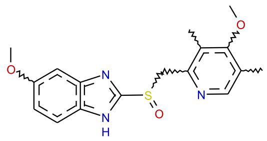	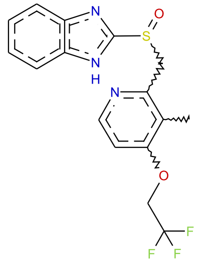	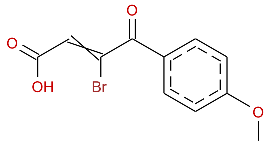
Actual Endpoint	Single-Carcinogen	Multiple-Carcinogen	Single-Carcinogen
Predicted Endpoint	Single-Carcinogen	Multiple-Carcinogen	Single-Carcinogen
Distance	0.663	0.691	0.728
Reference	US FDA (Centre for Drug Eval.& Res./Off. Testing & Res.) Sept. 1997	US FDA (Centre for Drug Eval.& Res./Off. Testing & Res.) Sept. 1997	US FDA (Centre for Drug Eval.& Res./Off. Testing & Res.) Sept. 1997

Model Applicability

Unknown features are fingerprint features in the query molecule, but not found in the training set.

1. All properties and OPS components are within expected ranges.

Feature Contribution

Top features for positive contribution

Fingerprint	Bit/Smiles	Feature Structure	Score	Multiple-Carcinogen in training set
SCFP_8	1311339974	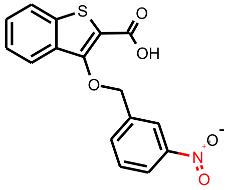 [*][N+](=O)[*]	0.453	8 out of 13

SCFP_8	10	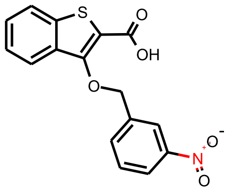 [*][N+](=[*])[*]	0.226	18 out of 39
SCFP_8	2109165795	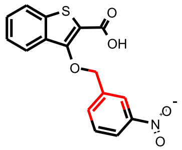 [*]C[c](:[cH]:[*]):[cH]:[*]	0.207	19 out of 42
**Top Features for negative contribution**				
**Fingerprint**	**Bit/Smiles**	**Feature Structure**	**Score**	**Multiple-Carcinogen in training set**
SCFP_8	-424485343	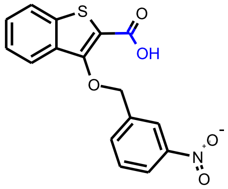 [*]C(=[*])O	-0.584	3 out of 17
SCFP_8	-1630708879	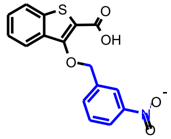 [*]C[c]1:[cH]:[cH]:[cH]:[c](:[cH]:1)[N+](=[*])[*]	-0.546	0 out of 2
SCFP_8	-1379591900	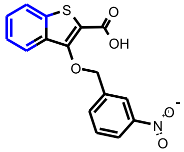 [*]:[c]1:[*]:[cH]:[cH]:[cH]:[cH]:1	-0.275	13 out of 48

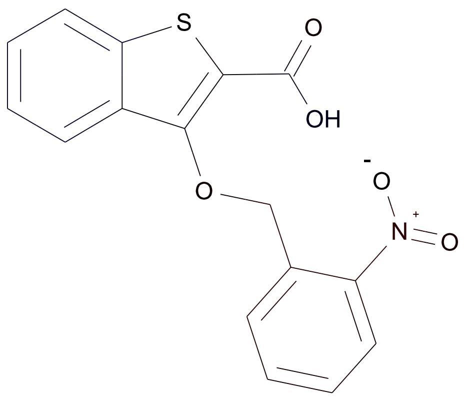

$C_{16}H_{11}NO_5S$
Molecular Weight: 329.32724
ALogP: 4.074
Rotatable Bonds: 5
Acceptors: 5
Donors: 1

Model Prediction

Prediction: Single-Carcinogen
Probability: 0.564
Enrichment: 1.363
Bayesian Score: 0.073
Mahalanobis Distance: 18.312
Mahalanobis Distance p-value: 9.98e-009

Prediction: Positive if the Bayesian score is above the estimated best cutoff value from minimizing the false positive and false negative rate.
Probability: The esimated probability that the sample is in the positive category. This assumes that the Bayesian score follows a normal distribution and is different from the prediction using a cutoff.
Enrichment: An estimate of enrichment, that is, the increased likelihood (versus random) of this sample being in the category.
Bayesian Score: The standard Laplacian-modified Bayesian score.
Mahalanobis Distance: The Mahalanobis distance (MD) is the distance to the center of the training data. The larger the MD, the less trustworthy the prediction.
Mahalanobis Distance p-value: The p-value gives the fraction of training data with an MD greater than or equal to the one for the given sample, assuming normally distributed data. The smaller the p-value, the less trustworthy the prediciton. For highly non-normal X properties (e.g., fingerprints), the MD p-value is wildly inaccurate.

Structural Similar Compounds

Name	Omeprazole	Lansoprazole	Cytembena
Structure	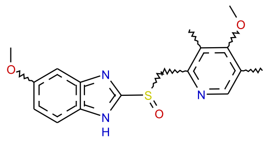	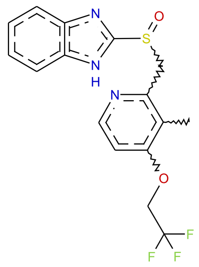	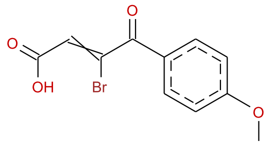
Actual Endpoint	Single-Carcinogen	Multiple-Carcinogen	Single-Carcinogen
Predicted Endpoint	Single-Carcinogen	Multiple-Carcinogen	Single-Carcinogen
Distance	0.662	0.691	0.727
Reference	US FDA (Centre for Drug Eval.& Res./Off. Testing & Res.) Sept. 1997	US FDA (Centre for Drug Eval.& Res./Off. Testing & Res.) Sept. 1997	US FDA (Centre for Drug Eval.& Res./Off. Testing & Res.) Sept. 1997

Model Applicability

Unknown features are fingerprint features in the query molecule, but not found in the training set.

1. All properties and OPS components are within expected ranges.

Feature Contribution

Top features for positive contribution

Fingerprint	Bit/Smiles	Feature Structure	Score	Multiple-Carcinogen in training set
SCFP_8	1311339974	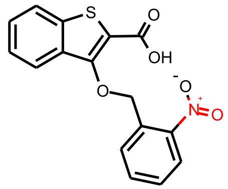 [*][N+](=O)[*]	0.453	8 out of 13

SCFP_8	1654335448	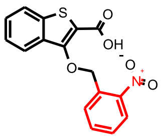 [*][c]1:[cH]:[cH]:[cH]:[cH]:[c]:1[N+](=[*])[*]	0.230	3 out of 6
SCFP_8	10	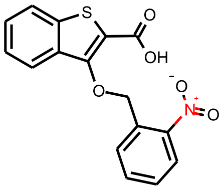 [*][N+](=[*])[*]	0.226	18 out of 39
**Top Features for negative contribution**				
**Fingerprint**	**Bit/Smiles**	**Feature Structure**	**Score**	**Multiple-Carcinogen in training set**
SCFP_8	-424485343	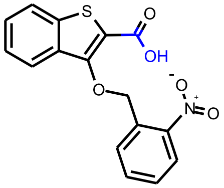 [*]C(=[*])O	-0.584	3 out of 17
SCFP_8	-1379591900	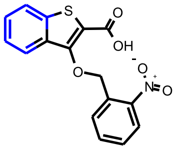 [*]:[c]1:[*]:[cH]:[cH]:[cH]:[cH]:1	-0.275	13 out of 48
SCFP_8	-1379673609	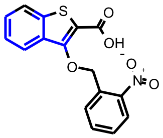 [*][c]1:[*]:[*]:[c]2:[*]:[cH]:[cH]:[cH]:[c]:1:2	-0.222	3 out of 11

Co-crystallized ligand

TOPKAT_Rat_Male_FDA_Single_vs_Multiple

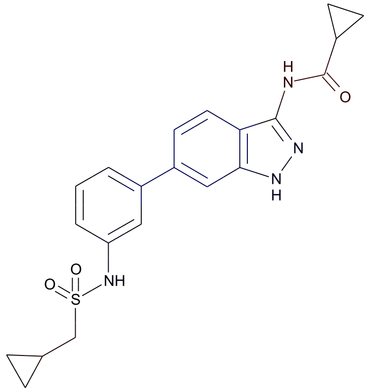

$C_{21}H_{22}N_4O_3S$
Molecular Weight: 410.48938
ALogP: 3.14
Rotatable Bonds: 7
Acceptors: 4
Donors: 3

Model Prediction

Prediction: Single-Carcinogen
Probability: 0.538
Enrichment: 1.300
Bayesian Score: -1.843
Mahalanobis Distance: 14.155
Mahalanobis Distance p-value: 6.33e-005

Prediction: Positive if the Bayesian score is above the estimated best cutoff value from minimizing the false positive and false negative rate.
Probability: The esimated probability that the sample is in the positive category. This assumes that the Bayesian score follows a normal distribution and is different from the prediction using a cutoff.
Enrichment: An estimate of enrichment, that is, the increased likelihood (versus random) of this sample being in the category.
Bayesian Score: The standard Laplacian-modified Bayesian score.
Mahalanobis Distance: The Mahalanobis distance (MD) is the distance to the center of the training data. The larger the MD, the less trustworthy the prediction.
Mahalanobis Distance p-value: The p-value gives the fraction of training data with an MD greater than or equal to the one for the given sample, assuming normally distributed data. The smaller the p-value, the less trustworthy the prediciton. For highly non-normal X properties (e.g., fingerprints), the MD p-value is wildly inaccurate.

Structural Similar Compounds

Name	Fluvastatin	Torsemide	Bicalutamide
Structure	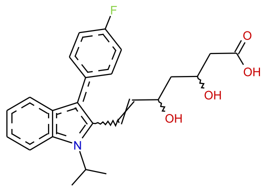	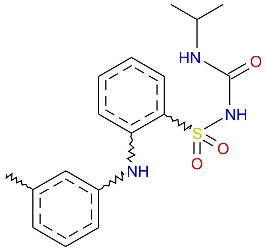	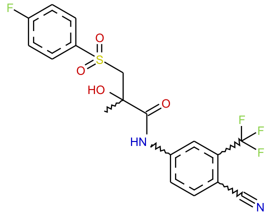
Actual Endpoint	Single-Carcinogen	Multiple-Carcinogen	Multiple-Carcinogen
Predicted Endpoint	Single-Carcinogen	Multiple-Carcinogen	Multiple-Carcinogen
Distance	0.635	0.642	0.672
Reference	US FDA (Centre for Drug Eval.& Res./Off. Testing & Res.) Sept. 1997	US FDA (Centre for Drug Eval.& Res./Off. Testing & Res.) Sept. 1997	US FDA (Centre for Drug Eval.& Res./Off. Testing & Res.) Sept. 1997

Model Applicability

Unknown features are fingerprint features in the query molecule, but not found in the training set.

1. OPS PC8 out of range. Value: 5.0682. Training min, max, SD, explained variance: -4.9055, 5.0367, 1.697, 0.0391.
2. OPS PC9 out of range. Value: -4.6117. Training min, max, SD, explained variance: -2.9055, 6.042, 1.612, 0.0353.

Feature Contribution

Top features for positive contribution

Fingerprint	Bit/Smiles	Feature Structure	Score	Multiple-Carcinogen in training set
SCFP_8	1631845520	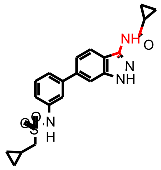 [*]C(=[*])N[c](:[*]):[*]	0.495	6 out of 9

SCFP_8	-26456656	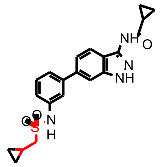 [*]S(=[*])(=[*])CC1[*<br>][*]1	0.383	1 out of 1
SCFP_8	-211646022	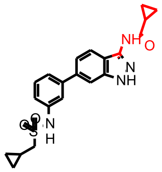 [*]:[c](:[*])NC(=O)C1<br>CC1	0.383	1 out of 1
**Top Features for negative contribution**				
**Fingerprint**	**Bit/Smiles**	**Feature Structure**	**Score**	**Multiple-Carcinogen in training set**
SCFP_8	1135377188	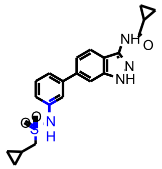 [*]S(=[*])(=[*])N[c](<br>:[*]):[*]	-0.737	0 out of 3
SCFP_8	1205795299	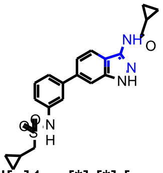 [*]N[c]1:n:[*]:[*]:[c<br>]:1:[*]	-0.546	0 out of 2
SCFP_8	667776369	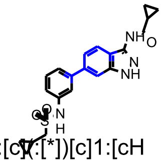 [*]:[c](:[*])[c]1:[cH<br>]:[*]:[c](:[*]):[cH]<br>:[cH]:1	-0.546	0 out of 2

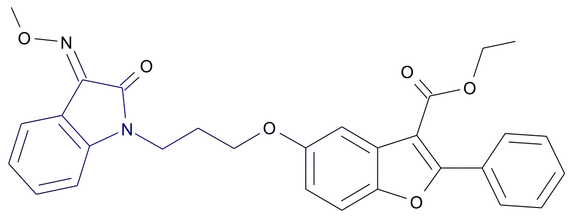

$C_{29}H_{26}N_2O_6$

Molecular Weight: 498.52653

ALogP: 4.994

Rotatable Bonds: 10

Acceptors: 6

Donors: 0

Model Prediction

Prediction: Non-Irritant

Probability: 0.880

Enrichment: 0.956

Bayesian Score: -2.957

Mahalanobis Distance: 10.036

Mahalanobis Distance p-value: 0.0571

Prediction: Positive if the Bayesian score is above the estimated best cutoff value from minimizing the false positive and false negative rate.
Probability: The esimated probability that the sample is in the positive category. This assumes that the Bayesian score follows a normal distribution and is different from the prediction using a cutoff.
Enrichment: An estimate of enrichment, that is, the increased likelihood (versus random) of this sample being in the category.
Bayesian Score: The standard Laplacian-modified Bayesian score.
Mahalanobis Distance: The Mahalanobis distance (MD) is the distance to the center of the training data. The larger the MD, the less trustworthy the prediction.
Mahalanobis Distance p-value: The p-value gives the fraction of training data with an MD greater than or equal to the one for the given sample, assuming normally distributed data. The smaller the p-value, the less trustworthy the prediciton. For highly non-normal X properties (e.g., fingerprints), the MD p-value is wildly inaccurate.

Structural Similar Compounds

Name	Carbamic acid, ((dibutylamino)thio)methyl-, 2,2-dimethyl-2,3-dihydro-7-benzofuranyl ester	Propanoic acid, 2-(4-((5-(trifluoromethyl)-2-pyridinyl)oxy)phenoxy) -, butyl ester	Ethanone, 2-((4-(2,4-dichloro-3-methylbenzoyl)-1,3-dimethyl-1H-pyra zol-5-yl)oxy)- 1-(4-methylphenyl)-
Structure	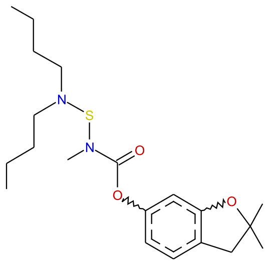	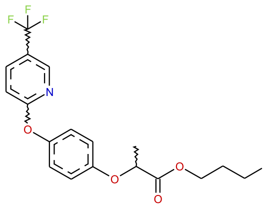	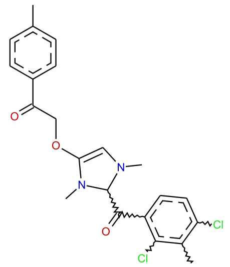
Actual Endpoint	Irritant	Irritant	Irritant
Predicted Endpoint	Irritant	Irritant	Non-Irritant
Distance	0.759	0.771	0.815
Reference	NTIS** National Technical Information Service. (Springfield, VA 22161) Forme rly U.S. Clearinghouse for Scientific & Technical Information. Volume(issue)/pag e/year: OTS0539690	NNGADV Nippon Noyaku Gakkaishi. Journal of the Pesticide Science Society of Japan. (Nippon Noyaku Gakkai, 1-43-11, Komagome, Toshima-ku, Tokyo 170, Japan) V.1- 1976- Volume(issue)/page/year: 15,305,1990	NNGADV Nippon Noyaku Gakkaishi. Journal of the Pesticide Science Society of Japan. (Nippon Noyaku Gakkai, 1-43-11, Komagome, Toshima-ku, Tokyo 170, Japan) V.1- 1976- Volume(issue)/page/year: 15,125,1990

Model Applicability

Unknown features are fingerprint features in the query molecule, but not found in the training set.

1. All properties and OPS components are within expected ranges.

Feature Contribution

Top features for positive contribution				
**Fingerprint**	**Bit/Smiles**	**Feature Structure**	**Score**	**Irritant in training set**

FCFP_12	-798628285	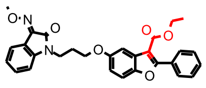 [*]:[c](:[*])C(=O)OCC	0.085	21 out of 21
FCFP_12	-1143686149	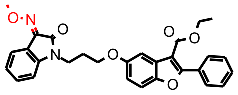 [*]=NOC	0.082	12 out of 12
FCFP_12	-157382193	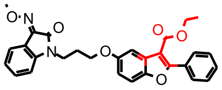 [*][c]1:[*]:[*]:[c](:[*]):[c]:1C(=O)OCC	0.066	3 out of 3
**Top Features for negative contribution**				
**Fingerprint**	**Bit/Smiles**	**Feature Structure**	**Score**	**Irritant in training set**
FCFP_12	-822674211	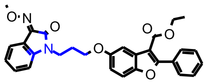 [*]CCCN1C(=[*])[*][*]:[c]1:[*]	-0.650	0 out of 1
FCFP_12	-1432259023	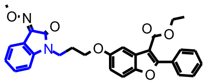 [*]CN1C(=[*])C(=[*])[c]2:[cH]:[cH]:[cH]:[cH]:[c]1:2	-0.627	1 out of 3

FCFP_12	159265197	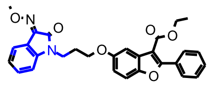 [*]CN1C(=[*])C(=[*])[c]2:[cH]:[*]:[cH]:[cH]:[c]1:2	-0.440	2 out of 4

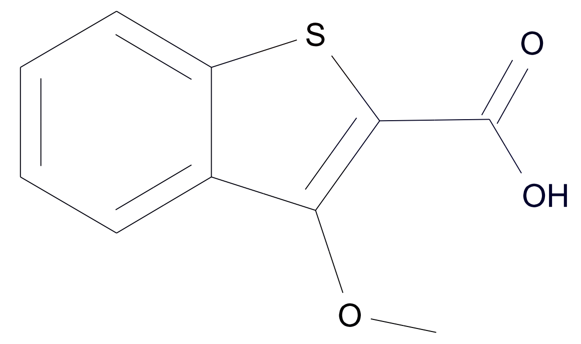

$C_{10}H_8O_3S$
Molecular Weight: 208.23372
ALogP: 2.596
Rotatable Bonds: 2
Acceptors: 3
Donors: 1

Structural Similar Compounds

Name	Phenol, 2,4-dichloro-6-nitro-	Benzoic acid, 2-chloro-4-nitro-	o-Nitrophenethyl alcohol
Structure	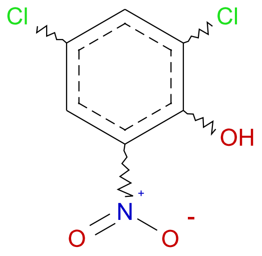	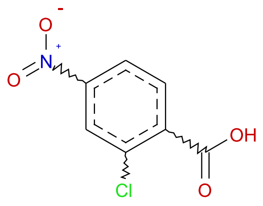	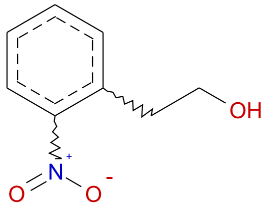
Actual Endpoint	Non-Irritant	Non-Irritant	Non-Irritant
Predicted Endpoint	Non-Irritant	Non-Irritant	Non-Irritant
Distance	0.515	0.584	0.596
Reference	28ZPAK -,80,72	28ZPAK -,91,72	US ARMY

Model Applicability

Unknown features are fingerprint features in the query molecule, but not found in the training set.

1. All properties and OPS components are within expected ranges.

Model Prediction

Prediction: Non-Irritant
Probability: 0.962
Enrichment: 1.045
Bayesian Score: -1.444
Mahalanobis Distance: 11.109
Mahalanobis Distance p-value: 0.00161

Prediction: Positive if the Bayesian score is above the estimated best cutoff value from minimizing the false positive and false negative rate.
Probability: The esimated probability that the sample is in the positive category. This assumes that the Bayesian score follows a normal distribution and is different from the prediction using a cutoff.
Enrichment: An estimate of enrichment, that is, the increased likelihood (versus random) of this sample being in the category.
Bayesian Score: The standard Laplacian-modified Bayesian score.
Mahalanobis Distance: The Mahalanobis distance (MD) is the distance to the center of the training data. The larger the MD, the less trustworthy the prediction.
Mahalanobis Distance p-value: The p-value gives the fraction of training data with an MD greater than or equal to the one for the given sample, assuming normally distributed data. The smaller the p-value, the less trustworthy the prediciton. For highly non-normal X properties (e.g., fingerprints), the MD p-value is wildly inaccurate.

Feature Contribution

Top features for positive contribution

Fingerprint	Bit/Smiles	Feature Structure	Score	Irritant in training set
FCFP_12	-1539132615	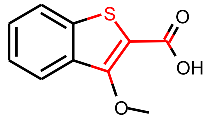 [*]C(=[*])[c]1:s:[*]:[*]:[c]:1[*]	0.079	9 out of 9

FCFP_12	-1716224640	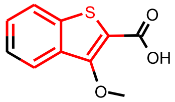 [*][c]1:o:[c]2:[cH]:[cH]:[*]:[cH]:[c]:2:[c]:1[*]	0.058	2 out of 2
**Top Features for negative contribution**				
**Fingerprint**	**Bit/Smiles**	**Feature Structure**	**Score**	**Irritant in training set**
FCFP_12	-1549222613	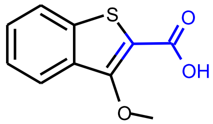 [*]:[c](:[*])C(=O)O	-0.612	5 out of 11
FCFP_12	-548632217	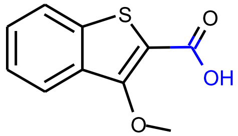 [*]C(=[*])O	-0.128	49 out of 61
FCFP_12	7	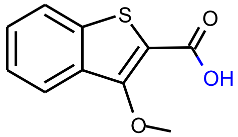 [*]O	-0.118	104 out of 128

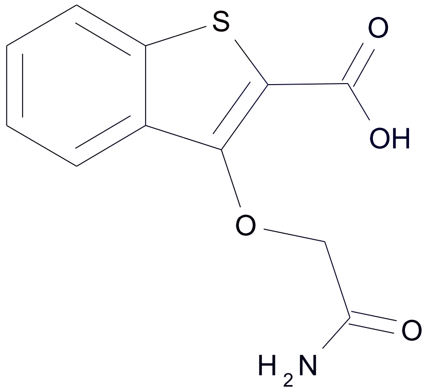

$C_{11}H_9NO_4S$
Molecular Weight: 251.25846
ALogP: 1.45
Rotatable Bonds: 4
Acceptors: 4
Donors: 2

Model Prediction

Prediction: Non-Irritant
Probability: 0.897
Enrichment: 0.974
Bayesian Score: -2.786
Mahalanobis Distance: 12.680
Mahalanobis Distance p-value: 6.06e-007

Prediction: Positive if the Bayesian score is above the estimated best cutoff value from minimizing the false positive and false negative rate.
Probability: The esimated probability that the sample is in the positive category. This assumes that the Bayesian score follows a normal distribution and is different from the prediction using a cutoff.
Enrichment: An estimate of enrichment, that is, the increased likelihood (versus random) of this sample being in the category.
Bayesian Score: The standard Laplacian-modified Bayesian score.
Mahalanobis Distance: The Mahalanobis distance (MD) is the distance to the center of the training data. The larger the MD, the less trustworthy the prediction.
Mahalanobis Distance p-value: The p-value gives the fraction of training data with an MD greater than or equal to the one for the given sample, assuming normally distributed data. The smaller the p-value, the less trustworthy the prediciton. For highly non-normal X properties (e.g., fingerprints), the MD p-value is wildly inaccurate.

Structural Similar Compounds

Name	o-Toluene sulfonamide,N-(2-hydroxyethyl)-4-nitro-	1-Naphthalene sulfonic acid	5-Isoindolinesulfonamide, 6-chloro-1,3-dioxo-
Structure	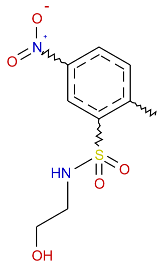	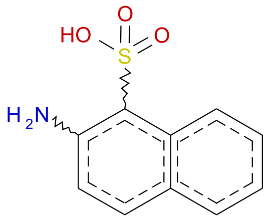	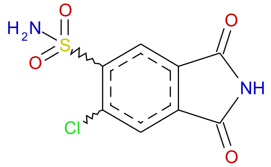
Actual Endpoint	Non-Irritant	Non-Irritant	Irritant
Predicted Endpoint	Non-Irritant	Non-Irritant	Non-Irritant
Distance	0.594	0.642	0.649
Reference	28ZPAK -,200,72	28ZPAK -,187,72	FCTOD7 Food and Chemical Toxicology. (Pergamon Press Inc., Maxwell House, Fa irview Park, Elmsford, NY 10523) V.20- 1982- Volume(issue)/page/year: 20,573 ,1982

Model Applicability

Unknown features are fingerprint features in the query molecule, but not found in the training set.

1. All properties and OPS components are within expected ranges.

Feature Contribution

Top features for positive contribution

Fingerprint	Bit/Smiles	Feature Structure	Score	Irritant in training set
FCFP_12	-1539132615	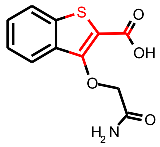 [*]C(=[*])[c]1:s:[*]:[*]:[c]:1[*]	0.079	9 out of 9

FCFP_12	-1716224640	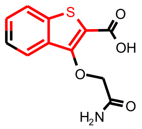<br>[*][c]1:o:[c]2:[cH]:[cH]:[*]:[cH]:[c]:2:[c]:1[*]	0.058	2 out of 2
**Top Features for negative contribution**				
**Fingerprint**	**Bit/Smiles**	**Feature Structure**	**Score**	**Irritant in training set**
FCFP_12	-1549222613	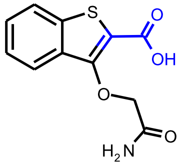<br>[*]:[c](:[*])C(=O)O	-0.612	5 out of 11
FCFP_12	566058135	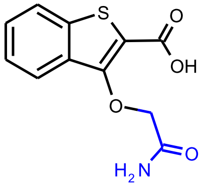<br>[*]CC(=O)N	-0.367	13 out of 21
FCFP_12	-1977359400	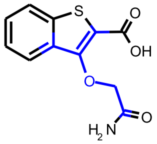<br>[*]CCO[c](:[cH]:[*]): [cH]:[*]	-0.243	17 out of 24

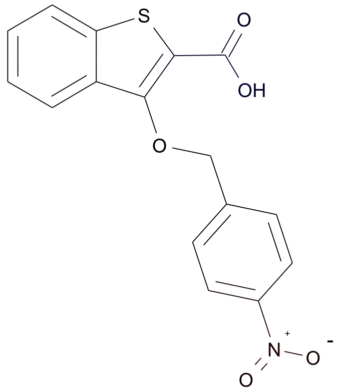

$C_{16}H_{11}NO_5S$
Molecular Weight: 329.32724
ALogP: 4.074
Rotatable Bonds: 5
Acceptors: 5
Donors: 1

Model Prediction

Prediction: Non-Irritant
Probability: 0.972
Enrichment: 1.056
Bayesian Score: -0.833
Mahalanobis Distance: 11.271
Mahalanobis Distance p-value: 0.000823

Prediction: Positive if the Bayesian score is above the estimated best cutoff value from minimizing the false positive and false negative rate.
Probability: The esimated probability that the sample is in the positive category. This assumes that the Bayesian score follows a normal distribution and is different from the prediction using a cutoff.
Enrichment: An estimate of enrichment, that is, the increased likelihood (versus random) of this sample being in the category.
Bayesian Score: The standard Laplacian-modified Bayesian score.
Mahalanobis Distance: The Mahalanobis distance (MD) is the distance to the center of the training data. The larger the MD, the less trustworthy the prediction.
Mahalanobis Distance p-value: The p-value gives the fraction of training data with an MD greater than or equal to the one for the given sample, assuming normally distributed data. The smaller the p-value, the less trustworthy the prediciton. For highly non-normal X properties (e.g., fingerprints), the MD p-value is wildly inaccurate.

Structural Similar Compounds

Name	Acetic acid, 2-(sec-butyl)-4,6-dinitrophenyl ester	Anisole, 6-t-butyl-3-methyl-2,4-dinitro-	Benzenesulfonic acid, 2-anilino-5-nitro-
Structure	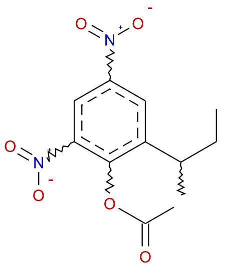	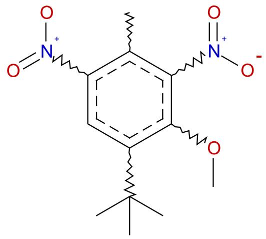	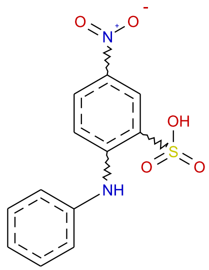
Actual Endpoint	Irritant	Irritant	Irritant
Predicted Endpoint	Non-Irritant	Non-Irritant	Non-Irritant
Distance	0.728	0.742	0.749
Reference	85JCAE "Prehled Prumyslove Toxikologie; Organicke Latky," Marhold, J., Prague , Czechoslovakia, Avicenum, 1986 Volume(issue)/page/year: -,750,1986	FCTXAV Food and Cosmetics Toxicology. (London, UK) V.1-19, 1963-81. For pu blisher information, see FCTOD7. Volume(issue)/page/year: 13,875,1975	85JCAE "Prehled Prumyslove Toxikologie; Organicke Latky," Marhold, J., Prague , Czechoslovakia, Avicenum, 1986 Volume(issue)/page/year: -,1061,1986

Model Applicability

Unknown features are fingerprint features in the query molecule, but not found in the training set.

1. All properties and OPS components are within expected ranges.

Feature Contribution

Top features for positive contribution

Fingerprint	Bit/Smiles	Feature Structure	Score	Irritant in training set
FCFP_12	5	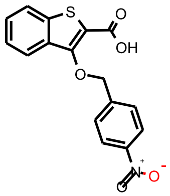 [*][O-]	0.085	27 out of 27

FCFP_12	8	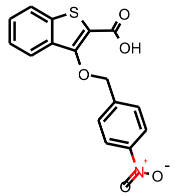 [*][N+](=[*])[*]	0.084	20 out of 20
FCFP_12	-828984032	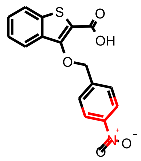 [*][N+](=[*])[c](:[cH]:[*]):[cH]:[*]	0.079	9 out of 9

Top Features for negative contribution

Fingerprint	Bit/Smiles	Feature Structure	Score	Irritant in training set
FCFP_12	-1549222613	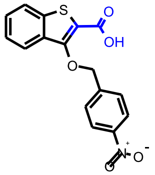 [*]:[c](:[*])C(=O)O	-0.612	5 out of 11
FCFP_12	-548632217	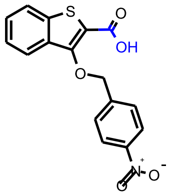 [*]C(=[*])O	-0.128	49 out of 61
FCFP_12	7	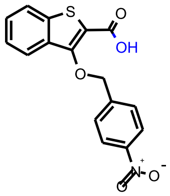 [*]O	-0.118	104 out of 128

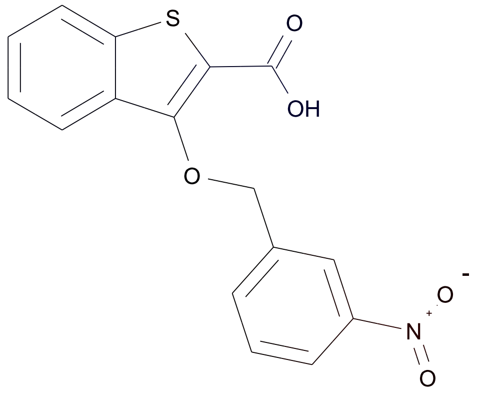

$C_{16}H_{11}NO_5S$
Molecular Weight: 329.32724
ALogP: 4.074
Rotatable Bonds: 5
Acceptors: 5
Donors: 1

Structural Similar Compounds

Name	Acetic acid, 2-(sec-butyl)-4,6-dinitrophenyl ester	Anisole, 6-t-butyl-3-methyl-2,4-dinitro-	Benzenesulfonic acid, 2-anilino-5-nitro-
Structure	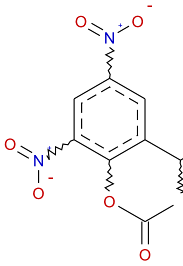	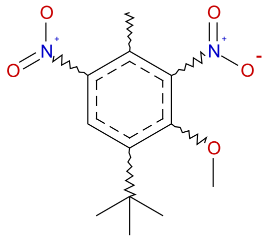	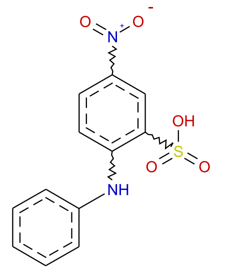
Actual Endpoint	Irritant	Irritant	Irritant
Predicted Endpoint	Non-Irritant	Non-Irritant	Non-Irritant
Distance	0.728	0.742	0.749
Reference	85JCAE "Prehled Prumyslove Toxikologie; Organicke Latky," Marhold, J., Prague , Czechoslovakia, Avicenum, 1986 Volume(issue)/page/year: -,750,1986	FCTXAV Food and Cosmetics Toxicology. (London, UK) V.1-19, 1963-81. For pu blisher information, see FCTOD7. Volume(issue)/page/year: 13,875,1975	85JCAE "Prehled Prumyslove Toxikologie; Organicke Latky," Marhold, J., Prague , Czechoslovakia, Avicenum, 1986 Volume(issue)/page/year: -,1061,1986

Model Prediction

Prediction: Non-Irritant
Probability: 0.973
Enrichment: 1.057
Bayesian Score: -0.763
Mahalanobis Distance: 11.271
Mahalanobis Distance p-value: 0.000823

Prediction: Positive if the Bayesian score is above the estimated best cutoff value from minimizing the false positive and false negative rate.
Probability: The esimated probability that the sample is in the positive category. This assumes that the Bayesian score follows a normal distribution and is different from the prediction using a cutoff.
Enrichment: An estimate of enrichment, that is, the increased likelihood (versus random) of this sample being in the category.
Bayesian Score: The standard Laplacian-modified Bayesian score.
Mahalanobis Distance: The Mahalanobis distance (MD) is the distance to the center of the training data. The larger the MD, the less trustworthy the prediction.
Mahalanobis Distance p-value: The p-value gives the fraction of training data with an MD greater than or equal to the one for the given sample, assuming normally distributed data. The smaller the p-value, the less trustworthy the prediciton. For highly non-normal X properties (e.g., fingerprints), the MD p-value is wildly inaccurate.

Model Applicability

Unknown features are fingerprint features in the query molecule, but not found in the training set.

1. All properties and OPS components are within expected ranges.

Feature Contribution

Top features for positive contribution

Fingerprint	Bit/Smiles	Feature Structure	Score	Irritant in training set
FCFP_12	5	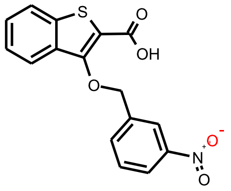 [*][O-]	0.085	27 out of 27

FCFP_12	8	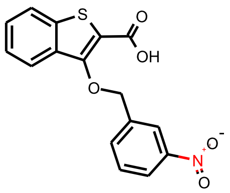 [*][N+](=[*])[*]	0.084	20 out of 20
FCFP_12	-1539132615	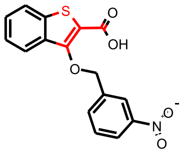 [*]C(=[*])[c]1:s:[*]:[*]:[c]:1[*]	0.079	9 out of 9

Top Features for negative contribution

Fingerprint	Bit/Smiles	Feature Structure	Score	Irritant in training set
FCFP_12	-1549222613	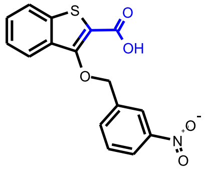 [*]:[c](:[*])C(=O)O	-0.612	5 out of 11
FCFP_12	-548632217	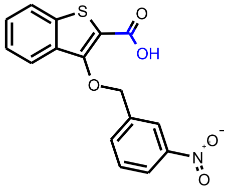 [*]C(=[*])O	-0.128	49 out of 61
FCFP_12	7	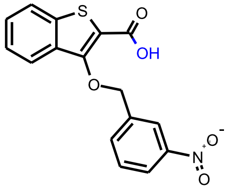 [*]O	-0.118	104 out of 128

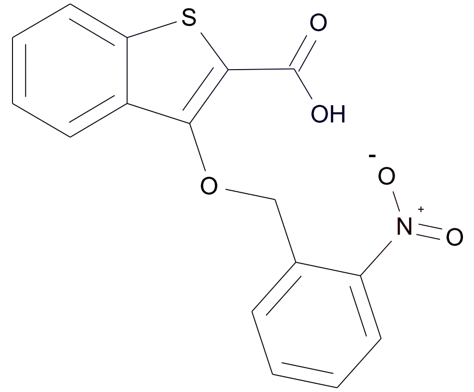

$C_{16}H_{11}NO_5S$
Molecular Weight: 329.32724
ALogP: 4.074
Rotatable Bonds: 5
Acceptors: 5
Donors: 1

Model Prediction

Prediction: Non-Irritant
Probability: 0.971
Enrichment: 1.054
Bayesian Score: -0.965
Mahalanobis Distance: 11.271
Mahalanobis Distance p-value: 0.000823

Prediction: Positive if the Bayesian score is above the estimated best cutoff value from minimizing the false positive and false negative rate.
Probability: The esimated probability that the sample is in the positive category. This assumes that the Bayesian score follows a normal distribution and is different from the prediction using a cutoff.
Enrichment: An estimate of enrichment, that is, the increased likelihood (versus random) of this sample being in the category.
Bayesian Score: The standard Laplacian-modified Bayesian score.
Mahalanobis Distance: The Mahalanobis distance (MD) is the distance to the center of the training data. The larger the MD, the less trustworthy the prediction.
Mahalanobis Distance p-value: The p-value gives the fraction of training data with an MD greater than or equal to the one for the given sample, assuming normally distributed data. The smaller the p-value, the less trustworthy the prediciton. For highly non-normal X properties (e.g., fingerprints), the MD p-value is wildly inaccurate.

Structural Similar Compounds

Name	Acetic acid, 2-(sec-butyl)-4,6-dinitrophenyl ester	Anisole, 6-t-butyl-3-methyl-2,4-dinitro-	Benzenesulfonic acid, 2-anilino-5-nitro-
Structure	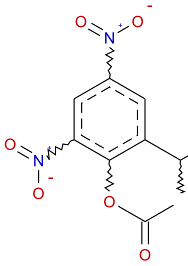	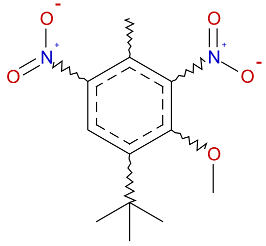	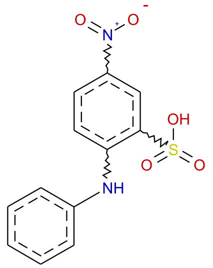
Actual Endpoint	Irritant	Irritant	Irritant
Predicted Endpoint	Non-Irritant	Non-Irritant	Non-Irritant
Distance	0.726	0.740	0.747
Reference	85JCAE "Prehled Prumyslove Toxikologie; Organicke Latky," Marhold, J., Prague , Czechoslovakia, Avicenum, 1986 Volume(issue)/page/year: -,750,1986	FCTXAV Food and Cosmetics Toxicology. (London, UK) V.1-19, 1963-81. For pu blisher information, see FCTOD7. Volume(issue)/page/year: 13,875,1975	85JCAE "Prehled Prumyslove Toxikologie; Organicke Latky," Marhold, J., Prague , Czechoslovakia, Avicenum, 1986 Volume(issue)/page/year: -,1061,1986

Model Applicability

Unknown features are fingerprint features in the query molecule, but not found in the training set.

1. All properties and OPS components are within expected ranges.

Feature Contribution

Top features for positive contribution

Fingerprint	Bit/Smiles	Feature Structure	Score	Irritant in training set
FCFP_12	5	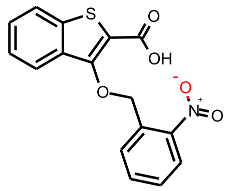 [*][O-]	0.085	27 out of 27

FCFP_12	8	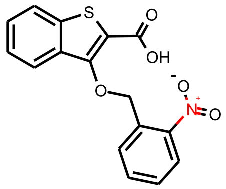 [*][N+](=[*])[*]	0.084	20 out of 20
FCFP_12	-828984032	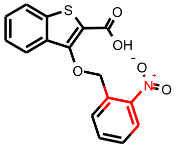 [*][N+](=[*])[c](:[cH]:[*]):[cH]:[*]	0.079	9 out of 9

Top Features for negative contribution

Fingerprint	Bit/Smiles	Feature Structure	Score	Irritant in training set
FCFP_12	-1549222613	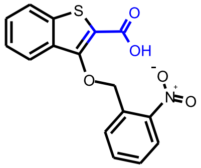 [*]:[c](:[*])C(=O)O	-0.612	5 out of 11
FCFP_12	-548632217	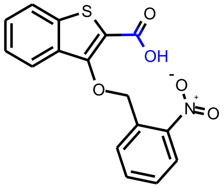 [*]C(=[*])O	-0.128	49 out of 61
FCFP_12	7	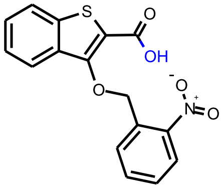 [*]O	-0.118	104 out of 128

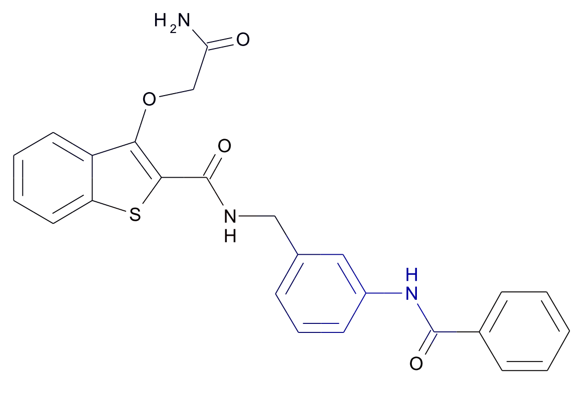

$C_{25}H_{21}N_3O_4S$

Molecular Weight: 459.51694

ALogP: 3.396

Rotatable Bonds: 8

Acceptors: 4

Donors: 3

Model Prediction

Prediction: Non-Irritant

Probability: 0.508

Enrichment: 0.551

Bayesian Score: -4.528

Mahalanobis Distance: 12.168

Mahalanobis Distance p-value: 1.1e-005

Prediction: Positive if the Bayesian score is above the estimated best cutoff value from minimizing the false positive and false negative rate.
Probability: The esimated probability that the sample is in the positive category. This assumes that the Bayesian score follows a normal distribution and is different from the prediction using a cutoff.
Enrichment: An estimate of enrichment, that is, the increased likelihood (versus random) of this sample being in the category.
Bayesian Score: The standard Laplacian-modified Bayesian score.
Mahalanobis Distance: The Mahalanobis distance (MD) is the distance to the center of the training data. The larger the MD, the less trustworthy the prediction.
Mahalanobis Distance p-value: The p-value gives the fraction of training data with an MD greater than or equal to the one for the given sample, assuming normally distributed data. The smaller the p-value, the less trustworthy the prediciton. For highly non-normal X properties (e.g., fingerprints), the MD p-value is wildly inaccurate.

Structural Similar Compounds

Name	Urea, 1,3-bis(2-benzothiazolylthiomethyl)-	Benzenesulfonic acid, 2,2'-(4,4'-biphenylylenedivinylene)di-, disod ium salt	2-Anthracenesulfonic acid, 1-amino-9,10-dihydro-9,10-dioxo-4-(2,4,6 -trimethylanilino)-, monosodium salt
Structure	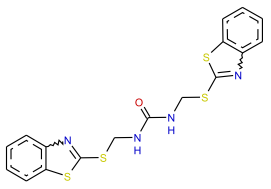	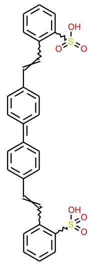	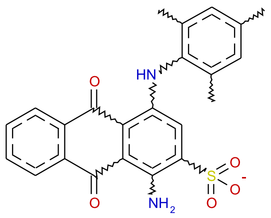
Actual Endpoint	Irritant	Irritant	Irritant
Predicted Endpoint	Irritant	Non-Irritant	Non-Irritant
Distance	0.843	0.865	0.902
Reference	AMIHBC AMA Archives of Industrial Hygiene and Occupational Medicine. (Chicago , IL) V.2-10, 1950-54. For publisher information, see AEHLAU. Volume(issue)/pag e/year: 5,311,1952	MVCRB3 MVC-Report. (Stockholm, Sweden) No.1-2, 1972-73. Discontinued. Volu me(issue)/page/year: 2,193,1973	85JCAE "Prehled Prumyslove Toxikologie; Organicke Latky," Marhold, J., Prague , Czechoslovakia, Avicenum, 1986 Volume(issue)/page/year: -,1327,1986

Model Applicability

Unknown features are fingerprint features in the query molecule, but not found in the training set.

1. All properties and OPS components are within expected ranges.

Feature Contribution

Top features for positive contribution

Fingerprint	Bit/Smiles	Feature Structure	Score	Irritant in training set

FCFP_12	-1539132615	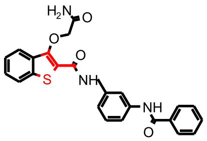 [*]C(=[*])[c]1:s:[*]:[*]:[c]:1[*]	0.079	9 out of 9
FCFP_12	907096426	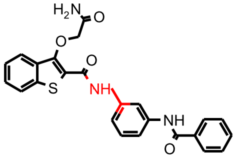 [*]NC[c](:[*]):[*]	0.077	7 out of 7
FCFP_12	427906732	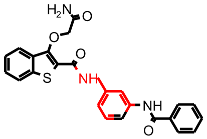 [*]NC[c]1:[cH]:[cH]:[*]:[c]([*]):[cH]:1	0.076	6 out of 6

Top Features for negative contribution

Fingerprint	Bit/Smiles	Feature Structure	Score	Irritant in training set
FCFP_12	-1838187238	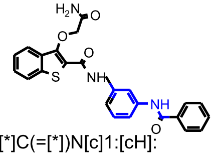 [*]C(=[*])N[c]1:[cH]:[cH]:[*]:[c]([*]):[cH]:1	-0.692	5 out of 12
FCFP_12	-792685140	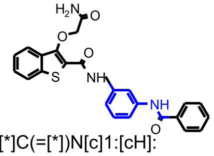 [*]C(=[*])N[c]1:[cH]:[cH]:[cH]:[c]([*]):[cH]:1	-0.650	0 out of 1

FCFP_12	1294255210	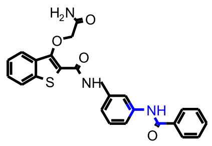 [*]C(=[*])N[c](:[*]):[*]	-0.486	12 out of 22

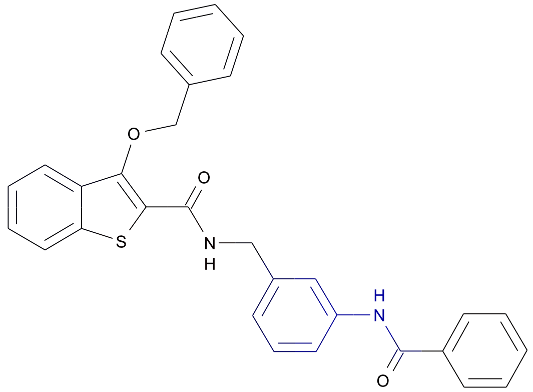

$C_{30}H_{24}N_2O_3S$
Molecular Weight: 492.58816
ALogP: 6.126
Rotatable Bonds: 8
Acceptors: 3
Donors: 2

Model Prediction

Prediction: Non-Irritant
Probability: 0.750
Enrichment: 0.814
Bayesian Score: -3.744
Mahalanobis Distance: 8.646
Mahalanobis Distance p-value: 0.612

Prediction: Positive if the Bayesian score is above the estimated best cutoff value from minimizing the false positive and false negative rate.
Probability: The esimated probability that the sample is in the positive category. This assumes that the Bayesian score follows a normal distribution and is different from the prediction using a cutoff.
Enrichment: An estimate of enrichment, that is, the increased likelihood (versus random) of this sample being in the category.
Bayesian Score: The standard Laplacian-modified Bayesian score.
Mahalanobis Distance: The Mahalanobis distance (MD) is the distance to the center of the training data. The larger the MD, the less trustworthy the prediction.
Mahalanobis Distance p-value: The p-value gives the fraction of training data with an MD greater than or equal to the one for the given sample, assuming normally distributed data. The smaller the p-value, the less trustworthy the prediciton. For highly non-normal X properties (e.g., fingerprints), the MD p-value is wildly inaccurate.

Structural Similar Compounds

Name	Sulfide, bis(4-t-butyl-m-cresyl)-	Benzenesulfonic acid, 2,2'-(4,4'-biphenylylenedivinylene)di-, disod ium salt	N-o-tolylmaleidide
Structure	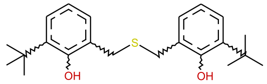	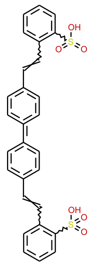	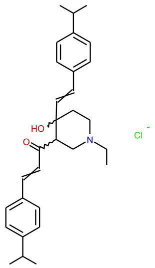
Actual Endpoint	Irritant	Irritant	Irritant
Predicted Endpoint	Irritant	Non-Irritant	Irritant
Distance	0.810	0.817	0.875
Reference	AMIHBC AMA Archives of Industrial Hygiene and Occupational Medicine. (Chicago , IL) V.2-10, 1950-54. For publisher information, see AEHLAU. Volume(issue)/pag e/year: 5,311,1952	MVCRB3 MVC-Report. (Stockholm, Sweden) No.1-2, 1972-73. Discontinued. Volu me(issue)/page/year: 2,193,1973	US ARMY

Model Applicability

Unknown features are fingerprint features in the query molecule, but not found in the training set.

1. All properties and OPS components are within expected ranges.

Feature Contribution

Top features for positive contribution				
Fingerprint	Bit/Smiles	Feature Structure	Score	Irritant in training set

FCFP_12	-1539132615	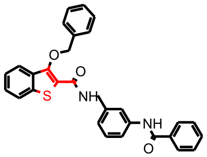 [*]C(=[*])[c]1:s:[*]:[*]:[c]:1[*]	0.079	9 out of 9
FCFP_12	907096426	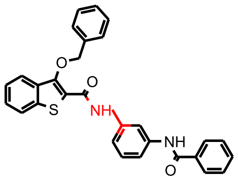 [*]NC[c](:[*]):[*]	0.077	7 out of 7
FCFP_12	427906732	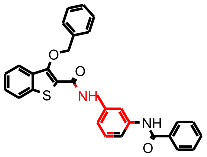 [*]NC[c]1:[cH]:[cH]:[*]:[c]([*]):[cH]:1	0.076	6 out of 6

Top Features for negative contribution

Fingerprint	Bit/Smiles	Feature Structure	Score	Irritant in training set
FCFP_12	-1838187238	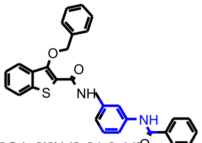 [*]C(=[*])N[c]1:[cH]:[cH]:[*]:[c]([*]):[cH]:1	-0.692	5 out of 12
FCFP_12	-792685140	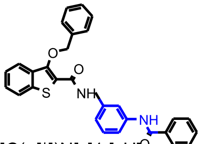 [*]C(=[*])N[c]1:[cH]:[cH]:[cH]:[c]([*]):[cH]:1	-0.650	0 out of 1

FCFP_12	1294255210	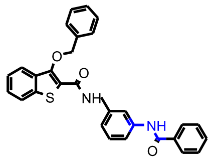<br>[*]C(=[*])N[c](:[*]):[*]	-0.486	12 out of 22

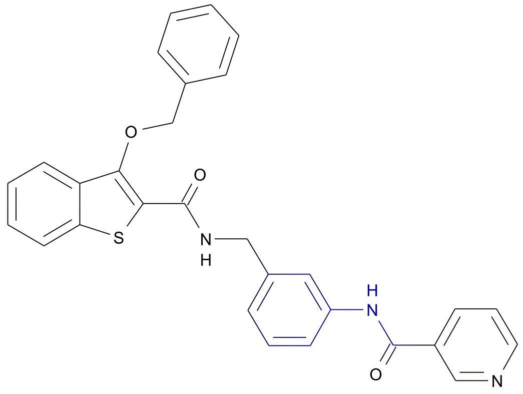

$C_{29}H_{23}N_3O_3S$
Molecular Weight: 493.57622
ALogP: 4.976
Rotatable Bonds: 8
Acceptors: 4
Donors: 2

Model Prediction

Prediction: Non-Irritant
Probability: 0.781
Enrichment: 0.848
Bayesian Score: -3.605
Mahalanobis Distance: 8.280
Mahalanobis Distance p-value: 0.787

Prediction: Positive if the Bayesian score is above the estimated best cutoff value from minimizing the false positive and false negative rate.
Probability: The esimated probability that the sample is in the positive category. This assumes that the Bayesian score follows a normal distribution and is different from the prediction using a cutoff.
Enrichment: An estimate of enrichment, that is, the increased likelihood (versus random) of this sample being in the category.
Bayesian Score: The standard Laplacian-modified Bayesian score.
Mahalanobis Distance: The Mahalanobis distance (MD) is the distance to the center of the training data. The larger the MD, the less trustworthy the prediction.
Mahalanobis Distance p-value: The p-value gives the fraction of training data with an MD greater than or equal to the one for the given sample, assuming normally distributed data. The smaller the p-value, the less trustworthy the prediciton. For highly non-normal X properties (e.g., fingerprints), the MD p-value is wildly inaccurate.

Structural Similar Compounds

Name	Benzenesulfonic acid, 2,2'-(4,4'-biphenylylenedivinylene)di-, disod ium salt	Anthraquinone, 1,1'-iminodi-	Sulfide, bis(4-t-butyl-m-cresyl)-
Structure	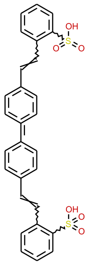	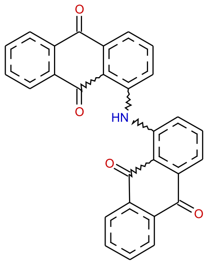	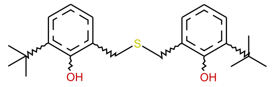
Actual Endpoint	Irritant	Irritant	Irritant
Predicted Endpoint	Non-Irritant	Non-Irritant	Irritant
Distance	0.717	0.862	0.887
Reference	MVCRB3 MVC-Report. (Stockholm, Sweden) No.1-2, 1972-73. Discontinued. Volu me(issue)/page/year: 2,193,1973	85JCAE "Prehled Prumyslove Toxikologie; Organicke Latky," Marhold, J., Prague , Czechoslovakia, Avicenum, 1986 Volume(issue)/page/year: -,735,1986	AMIHBC AMA Archives of Industrial Hygiene and Occupational Medicine. (Chicago , IL) V.2-10, 1950-54. For publisher information, see AEHLAU. Volume(issue)/pag e/year: 5,311,1952

Model Applicability

Unknown features are fingerprint features in the query molecule, but not found in the training set.

1. All properties and OPS components are within expected ranges.

Feature Contribution

Top features for positive contribution				
Fingerprint	Bit/Smiles	Feature Structure	Score	Irritant in training set

FCFP_12	-124655670	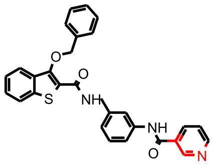<br>[*][c](:[*]):[cH]:n:[<br>*]	0.082	13 out of 13
FCFP_12	-1539132615	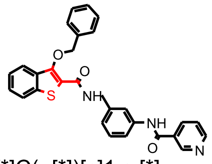<br>[*]C(=[*])[c]1:s:[*]:<br>[*]:[c]:1[*]	0.079	9 out of 9
FCFP_12	-1695756380	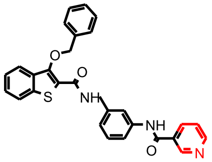<br>[*]1:[cH]:[cH]:[cH]:n<br>:[cH]:1	0.077	7 out of 7

Top Features for negative contribution

Fingerprint	Bit/Smiles	Feature Structure	Score	Irritant in training set
FCFP_12	-1838187238	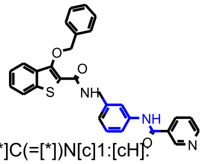<br>[*]C(=[*])N[c]1:[cH]:<br>[cH]:[*]:[c]([*]):[c<br>H]:1	-0.692	5 out of 12
FCFP_12	-792685140	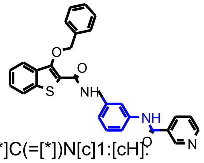<br>[*]C(=[*])N[c]1:[cH]:<br>[cH]:[cH]:[c]([*]):[<br>cH]:1	-0.650	0 out of 1

FCFP_12	1294255210	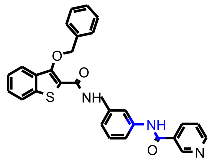 [*]C(=[*])N[c](:[*]):[*]	-0.486	12 out of 22

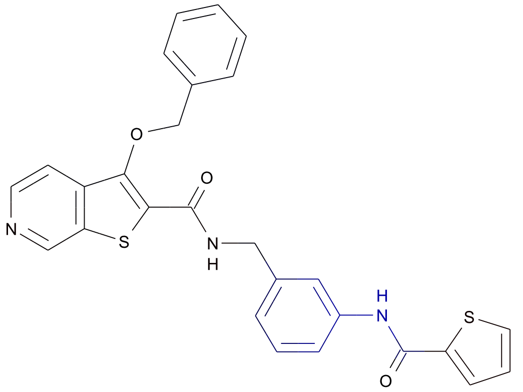

$C_{27}H_{21}N_3O_3S_2$

Molecular Weight: 499.60394

ALogP: 4.929

Rotatable Bonds: 8

Acceptors: 4

Donors: 2

Model Prediction

Prediction: Non-Irritant

Probability: 0.825

Enrichment: 0.896

Bayesian Score: -3.365

Mahalanobis Distance: 9.888

Mahalanobis Distance p-value: 0.0829

Prediction: Positive if the Bayesian score is above the estimated best cutoff value from minimizing the false positive and false negative rate.
Probability: The esimated probability that the sample is in the positive category. This assumes that the Bayesian score follows a normal distribution and is different from the prediction using a cutoff.
Enrichment: An estimate of enrichment, that is, the increased likelihood (versus random) of this sample being in the category.
Bayesian Score: The standard Laplacian-modified Bayesian score.
Mahalanobis Distance: The Mahalanobis distance (MD) is the distance to the center of the training data. The larger the MD, the less trustworthy the prediction.
Mahalanobis Distance p-value: The p-value gives the fraction of training data with an MD greater than or equal to the one for the given sample, assuming normally distributed data. The smaller the p-value, the less trustworthy the prediciton. For highly non-normal X properties (e.g., fingerprints), the MD p-value is wildly inaccurate.

Structural Similar Compounds

Name	Benzenesulfonic acid, 2,2'-(4,4'-biphenylylenedivinylene)di-, disod ium salt	Urea, 1,3-bis(2-benzothiazolylthiomethyl)-	2-Anthracenesulfonic acid, 1-amino-9,10-dihydro-9,10-dioxo-4-(2,4,6 -trimethylanilino)-, monosodium salt
Structure	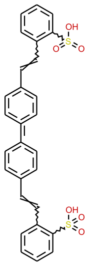	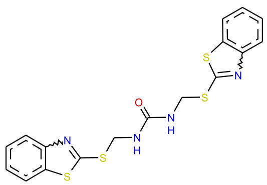	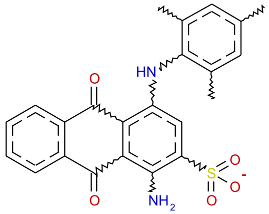
Actual Endpoint	Irritant	Irritant	Irritant
Predicted Endpoint	Non-Irritant	Irritant	Non-Irritant
Distance	0.705	0.821	0.937
Reference	MVCRB3 MVC-Report. (Stockholm, Sweden) No.1-2, 1972-73. Discontinued. Volu me(issue)/page/year: 2,193,1973	AMIHBC AMA Archives of Industrial Hygiene and Occupational Medicine. (Chicago , IL) V.2-10, 1950-54. For publisher information, see AEHLAU. Volume(issue)/pag e/year: 5,311,1952	85JCAE "Prehled Prumyslove Toxikologie; Organicke Latky," Marhold, J., Prague , Czechoslovakia, Avicenum, 1986 Volume(issue)/page/year: -,1327,1986

Model Applicability

Unknown features are fingerprint features in the query molecule, but not found in the training set.

1. All properties and OPS components are within expected ranges.

Feature Contribution

Top features for positive contribution

Fingerprint	Bit/Smiles	Feature Structure	Score	Irritant in training set

FCFP_12	-124655670	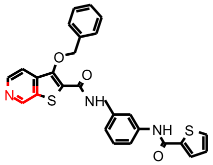 [*][c](:[*]):[cH]:n:[*]	0.082	13 out of 13
FCFP_12	-1539132615	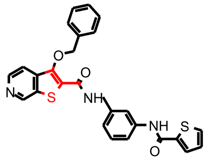 [*]C(=[*])[c]1:s:[*]: [*]:[c]:1[*]	0.079	9 out of 9
FCFP_12	907096426	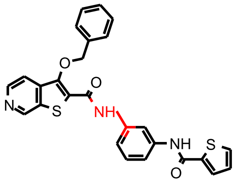 [*]NC[c](:[*]):[*]	0.077	7 out of 7

Top Features for negative contribution

Fingerprint	Bit/Smiles	Feature Structure	Score	Irritant in training set
FCFP_12	-1838187238	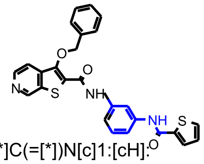 [*]C(=[*])N[c]1:[cH]: [cH]:[*]:[c]([*]):[c H]:1	-0.692	5 out of 12
FCFP_12	-792685140	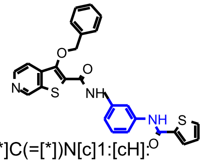 [*]C(=[*])N[c]1:[cH]: [cH]:[cH]:[c]([*]):[cH]:1	-0.650	0 out of 1

FCFP_12	1294255210	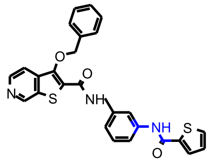 [*]C(=[*])N[c](:[*]):[*]	-0.486	12 out of 22

Co-crystallized ligand

TOPKAT_Skin_Irritancy_None_vs_Irritant

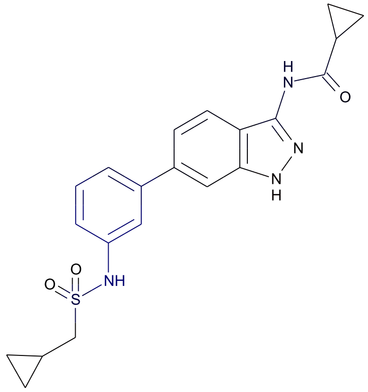

$C_{21}H_{22}N_4O_3S$
Molecular Weight: 410.48938
ALogP: 3.14
Rotatable Bonds: 7
Acceptors: 4
Donors: 3

Model Prediction

Prediction: Non-Irritant
Probability: 0.710
Enrichment: 0.771
Bayesian Score: -3.902
Mahalanobis Distance: 8.407
Mahalanobis Distance p-value: 0.732

Prediction: Positive if the Bayesian score is above the estimated best cutoff value from minimizing the false positive and false negative rate.
Probability: The esimated probability that the sample is in the positive category. This assumes that the Bayesian score follows a normal distribution and is different from the prediction using a cutoff.
Enrichment: An estimate of enrichment, that is, the increased likelihood (versus random) of this sample being in the category.
Bayesian Score: The standard Laplacian-modified Bayesian score.
Mahalanobis Distance: The Mahalanobis distance (MD) is the distance to the center of the training data. The larger the MD, the less trustworthy the prediction.
Mahalanobis Distance p-value: The p-value gives the fraction of training data with an MD greater than or equal to the one for the given sample, assuming normally distributed data. The smaller the p-value, the less trustworthy the prediciton. For highly non-normal X properties (e.g., fingerprints), the MD p-value is wildly inaccurate.

Structural Similar Compounds

Name	5-Norbornene-2,3-dicarboxylic acid, 1,4,5,6,7,7-hexachloro-	p-Acetophenetidide, 3'-(bis(2-hydroxyethyl)amino)-	Benzenesulfonic acid, 2-anilino-5-nitro-
Structure	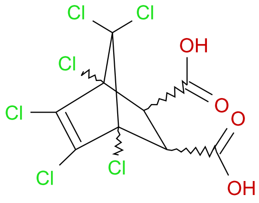	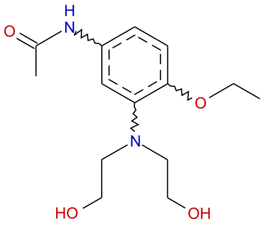	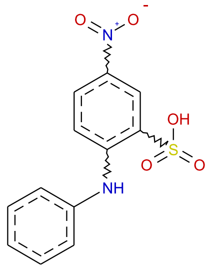
Actual Endpoint	Irritant	Non-Irritant	Irritant
Predicted Endpoint	Irritant	Non-Irritant	Non-Irritant
Distance	0.858	0.883	0.909
Reference	85JCAE "Prehled Prumyslove Toxikologie; Organicke Latky," Marhold, J., Prague , Czechoslovakia, Avicenum, 1986 Volume(issue)/page/year: -,581,1986	28ZPAK -,100,72	85JCAE "Prehled Prumyslove Toxikologie; Organicke Latky," Marhold, J., Prague , Czechoslovakia, Avicenum, 1986 Volume(issue)/page/year: -,1061,1986

Model Applicability

Unknown features are fingerprint features in the query molecule, but not found in the training set.

1. All properties and OPS components are within expected ranges.
2. Unknown FCFP_2 feature: 262592487: [*]:[c]1:[*]:[*]:n:[nH]:1
3. Unknown FCFP_2 feature: 1747267175: [*][c]1:[*]:[*]:[nH]:n:1

Feature Contribution

Top features for positive contribution				
Fingerprint	Bit/Smiles	Feature Structure	Score	Irritant in training set

FCFP_12	1940464803	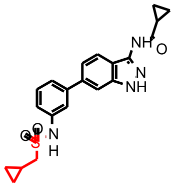 [*]S(=[*])(=[*])CC1CC 1	0.077	7 out of 7
FCFP_12	751214629	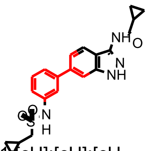 [*][c]1:[cH]:[cH]:[cH]:[c](:[cH]:1)[c](:[cH]:[*]):[cH]:[*]	0.073	5 out of 5
FCFP_12	19	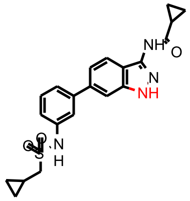 [*]:[nH]:[*]	0.066	3 out of 3

Top Features for negative contribution

Fingerprint	Bit/Smiles	Feature Structure	Score	Irritant in training set
FCFP_12	-1838187238	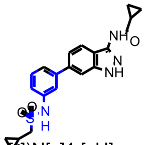 [*]C(=[*])N[c]1:[cH]: [cH]:[*]:[c]([*]):[c H]:1	-0.692	5 out of 12
FCFP_12	-792685140	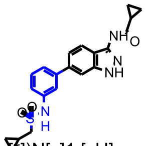 [*]C(=[*])N[c]1:[cH]: [cH]:[cH]:[c]([*]):[cH]:1	-0.650	0 out of 1

FCFP_12	1294255210	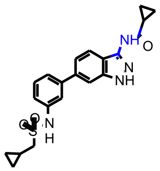 [*]C(=[*])N[c](:[*]):[*]	-0.486	12 out of 22

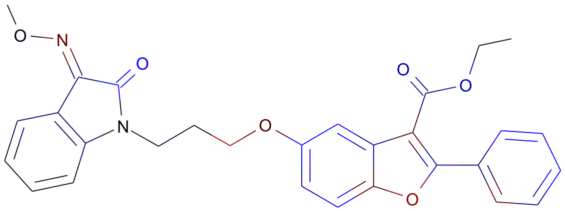

$C_{29}H_{26}N_2O_6$

Molecular Weight: 498.52653

ALogP: 4.994

Rotatable Bonds: 10

Acceptors: 6

Donors: 0

Model Prediction

Prediction: 44.302

Unit: mg/kg_body_weight/day

Mahalanobis Distance: 17.715

Mahalanobis Distance p-value: 5.46e-022

Mahalanobis Distance: The Mahalanobis distance (MD) is a generalization of the Euclidean distance that accounts for correlations among the X properties. It is calculated as the distance to the center of the training data. The larger the MD, the less trustworthy the prediction.
Mahalanobis Distance p-value: The p-value gives the fraction of training data with an MD greater than or equal to the one for the given sample, assuming normally distributed data. The smaller the p-value, the less trustworthy the prediciton. For highly non-normal X properties (e.g., fingerprints), the MD p-value is wildly inaccurate.

Structural Similar Compounds

Name	223	Estradiol mustard	Tamoxifen citrate
Structure	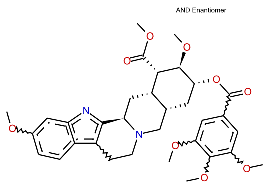	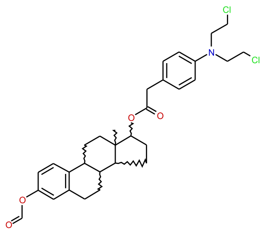	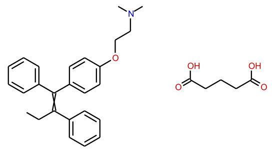
Actual Endpoint (-log C)	5.08368	5.58568	5.05965
Predicted Endpoint (-log C)	5.08273	5.97715	4.24168
Distance	0.849	0.860	0.904
Reference	CPDB	CPDB	CPDB

Model Applicability

Unknown features are fingerprint features in the query molecule, but not found in the training set.

1. OPS PC22 out of range. Value: 4.6468. Training min, max, SD, explained variance: -3.1587, 3.8589, 1.086, 0.0147.
2. OPS PC23 out of range. Value: 3.5777. Training min, max, SD, explained variance: -2.6901, 3.3252, 1.05, 0.0138.
3. Unknown ECFP_2 feature: -813643813: [*][c]1:[*]:[*]:o:[c]:1[c](:[*]):[*]
4. Unknown ECFP_2 feature: -1658273810: [*]C(=[*])[c]1:[c]([*]):[*]:[*]:[c]:1:[*]
5. Unknown ECFP_2 feature: -661097313: [*]CN1C(=[*])[*][*]:[c]1:[*]
6. Unknown ECFP_2 feature: -1236953626: [*]N1[*][*][c](:[*]):[c]1:[cH]:[*]
7. Unknown ECFP_2 feature: -820505146: [*]ON=C([*])[*]
8. Unknown ECFP_2 feature: -408704017: [*]=NOC

Feature Contribution

Top features for positive contribution

Fingerprint	Bit/Smiles	Feature Structure	Score

ECFP_6	1559650422	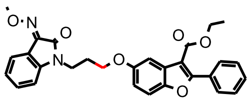 [*]C[*]	0.203
ECFP_6	683445015	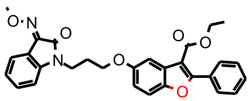 [*]:o:[*]	0.136
ECFP_6	-1087070950	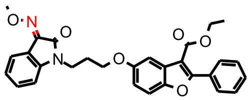 [*]N=[*]	0.104

Top Features for negative contribution

**Fingerprint**	**Bit/Smiles**	**Feature Structure**	**Score**
ECFP_6	2106656448	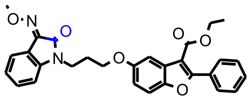 [*]C(=O)[*]	-0.275
ECFP_6	1996767644	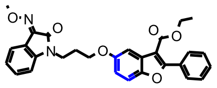 [*][c](:[*]):[cH]:[cH]:[*]	-0.251

ECFP_6	642810091	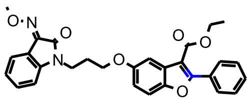 [*][c](:[*]):[*]	-0.247

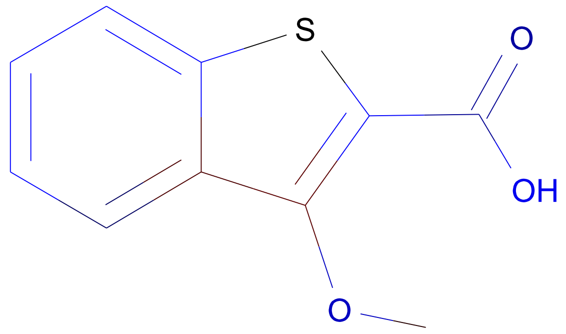

$C_{10}H_8O_3S$
Molecular Weight: 208.23372
ALogP: 2.596
Rotatable Bonds: 2
Acceptors: 3
Donors: 1

Model Prediction

Prediction: 431.948
Unit: mg/kg_body_weight/day
Mahalanobis Distance: 11.399
Mahalanobis Distance p-value: 0.0003

Mahalanobis Distance: The Mahalanobis distance (MD) is a generalization of the Euclidean distance that accounts for correlations among the X properties. It is calculated as the distance to the center of the training data. The larger the MD, the less trustworthy the prediction.
Mahalanobis Distance p-value: The p-value gives the fraction of training data with an MD greater than or equal to the one for the given sample, assuming normally distributed data. The smaller the p-value, the less trustworthy the prediciton. For highly non-normal X properties (e.g., fingerprints), the MD p-value is wildly inaccurate.

Structural Similar Compounds

Name	3-Methoxy-4-aminoazobenzene	6-Nitrobenzimidazole	4,4´-Oxydianiline
Structure	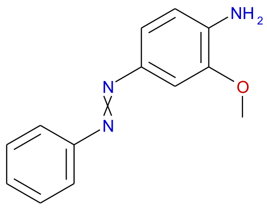	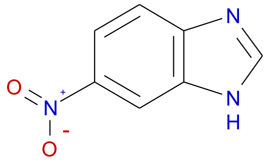	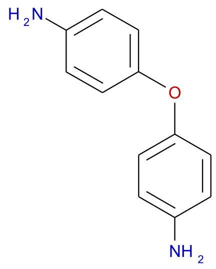
Actual Endpoint (-log C)	3.57693	2.642	3.7752
Predicted Endpoint (-log C)	2.89883	3.39256	3.57646
Distance	0.571	0.591	0.601
Reference	CPDB	CPDB	CPDB

Model Applicability

Unknown features are fingerprint features in the query molecule, but not found in the training set.

1. All properties and OPS components are within expected ranges.
2. Unknown ECFP_2 feature: -1670580914: [*]C(=[*])[c]1:s:[*]:[*]:[c]:1[*]
3. Unknown ECFP_2 feature: 1895035276: [*]:[cH]:[c]1:s:[*]:[*]:[c]:1:[*]

Feature Contribution

Top features for positive contribution			
**Fingerprint**	**Bit/Smiles**	**Feature Structure**	**Score**
ECFP_6	1333660716	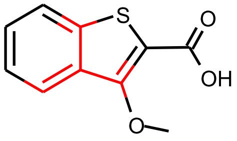 [*][c]1:[*]:[*]:[c](:[*]):[c]:1:[cH]:[*]	0.075

ECFP_6	734603939	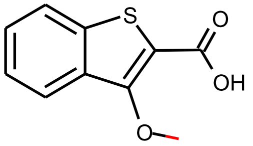 [*]C	0.042
ECFP_6	1307307440	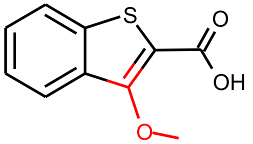 [*]:[c](:[*])OC	0.016

Top Features for negative contribution

Fingerprint	Bit/Smiles	Feature Structure	Score
ECFP_6	1996767644	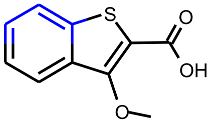 [*][c](:[*]):[cH]:[cH<br>]:[*]	-0.251
ECFP_6	642810091	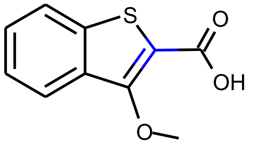 [*][c](:[*]):[*]	-0.247
ECFP_6	-182236392	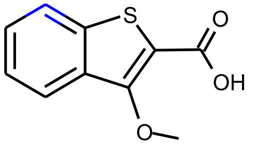 [*]:[cH]:[*]	-0.232

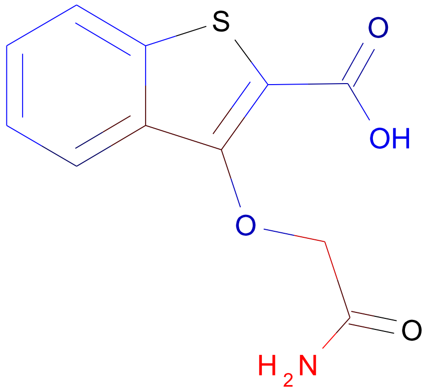

$C_{11}H_9NO_4S$
Molecular Weight: 251.25846
ALogP: 1.45
Rotatable Bonds: 4
Acceptors: 4
Donors: 2

Model Prediction

Prediction: 215.730
Unit: mg/kg_body_weight/day
Mahalanobis Distance: 11.329
Mahalanobis Distance p-value: 0.000399

Mahalanobis Distance: The Mahalanobis distance (MD) is a generalization of the Euclidean distance that accounts for correlations among the X properties. It is calculated as the distance to the center of the training data. The larger the MD, the less trustworthy the prediction.
Mahalanobis Distance p-value: The p-value gives the fraction of training data with an MD greater than or equal to the one for the given sample, assuming normally distributed data. The smaller the p-value, the less trustworthy the prediciton. For highly non-normal X properties (e.g., fingerprints), the MD p-value is wildly inaccurate.

Structural Similar Compounds

Name	2-Hydrazino-4-(p-nitrophenyl) thiazole	4-Ethylsulphonylnaphthalene-1-sulfonamide	Formic acid 2-[4-(5-nitro-2-furyl)-2-thiazolyl]hydrazide s
Structure	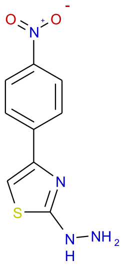	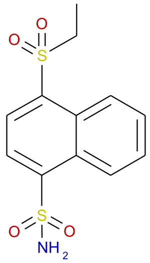	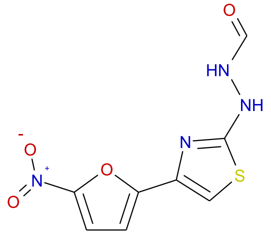
Actual Endpoint (-log C)	4.34807	4.15192	4.37179
Predicted Endpoint (-log C)	4.37858	3.54657	4.32851
Distance	0.557	0.566	0.578
Reference	CPDB	CPDB	CPDB

Model Applicability

Unknown features are fingerprint features in the query molecule, but not found in the training set.

1. OPS PC23 out of range. Value: 3.3455. Training min, max, SD, explained variance: -2.6901, 3.3252, 1.05, 0.0138.
2. Unknown ECFP_2 feature: -1670580914: [*]C(=[*])[c]1:s:[*]:[*]:[c]:1[*]
3. Unknown ECFP_2 feature: 1895035276: [*]:[cH]:[c]1:s:[*]:[*]:[c]:1:[*]
4. Unknown ECFP_2 feature: -1686813061: [*]OCC(=[*])[*]

Feature Contribution

Top features for positive contribution

Fingerprint	Bit/Smiles	Feature Structure	Score
ECFP_6	1572579716	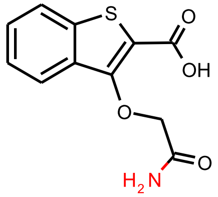 [*]N	0.225

ECFP_6	1559650422	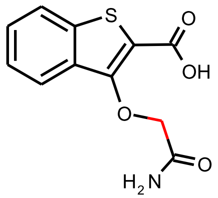<br>[*]C[*]	0.203
ECFP_6	-932108170	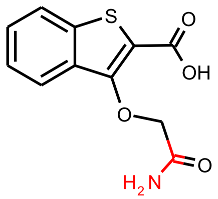<br>[*]C(=[*])N	0.078

Top Features for negative contribution

Fingerprint	Bit/Smiles	Feature Structure	Score
ECFP_6	1996767644	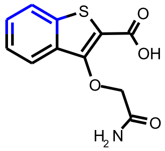<br>[*][c](:[*]):[cH]:[cH<br>]:[*]	-0.251
ECFP_6	642810091	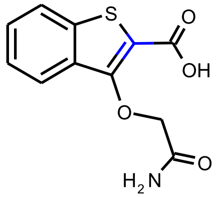<br>[*][c](:[*]):[*]	-0.247
ECFP_6	-182236392	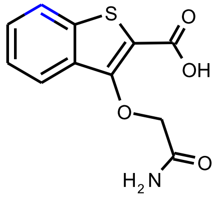<br>[*]:[cH]:[*]	-0.232

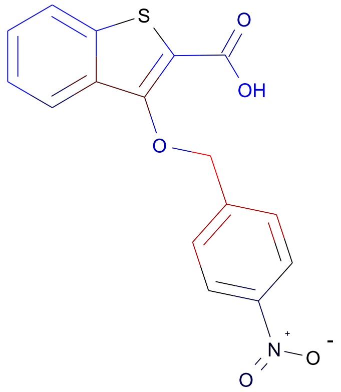

$C_{16}H_{11}NO_5S$
Molecular Weight: 329.32724
ALogP: 4.074
Rotatable Bonds: 5
Acceptors: 5
Donors: 1

Model Prediction

Prediction: 99.555
Unit: mg/kg_body_weight/day
Mahalanobis Distance: 11.596
Mahalanobis Distance p-value: 0.00013

Mahalanobis Distance: The Mahalanobis distance (MD) is a generalization of the Euclidean distance that accounts for correlations among the X properties. It is calculated as the distance to the center of the training data. The larger the MD, the less trustworthy the prediction.
Mahalanobis Distance p-value: The p-value gives the fraction of training data with an MD greater than or equal to the one for the given sample, assuming normally distributed data. The smaller the p-value, the less trustworthy the prediciton. For highly non-normal X properties (e.g., fingerprints), the MD p-value is wildly inaccurate.

Structural Similar Compounds

Name	646	Acifluorfen	470
Structure	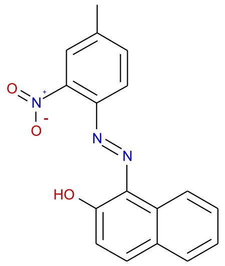	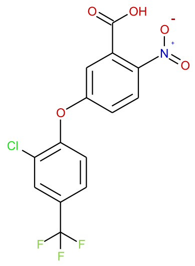	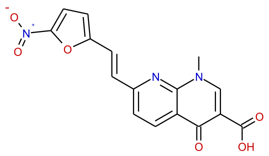
Actual Endpoint (-log C)	0.937339	3.40908	4.62839
Predicted Endpoint (-log C)	3.26294	3.10974	3.93264
Distance	0.628	0.663	0.682
Reference	CPDB	CPDB	CPDB

Model Applicability

Unknown features are fingerprint features in the query molecule, but not found in the training set.

1. All properties and OPS components are within expected ranges.
2. Unknown ECFP_2 feature: -1670580914: [*]C(=[*])[c]1:s:[*]:[*]:[c]:1[*]
3. Unknown ECFP_2 feature: 1895035276: [*]:[cH]:[c]1:s:[*]:[*]:[c]:1:[*]

Feature Contribution

Top features for positive contribution			
Fingerprint	Bit/Smiles	Feature Structure	Score
ECFP_6	1559650422	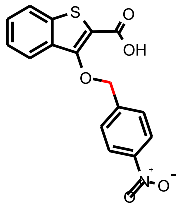 [*]C[*]	0.203

ECFP_6	-2024255407	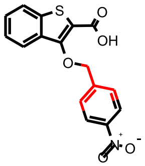 [*]C[c](:[cH]:[*]):[cH]:[*]	0.172
ECFP_6	781519895	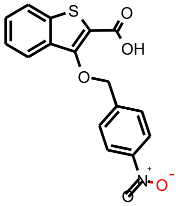 [*][O-]	0.080
**Top Features for negative contribution**			
**Fingerprint**	**Bit/Smiles**	**Feature Structure**	**Score**
ECFP_6	1996767644	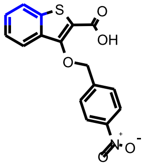 [*][c](:[*]):[cH]:[cH]:[*]	-0.251
ECFP_6	642810091	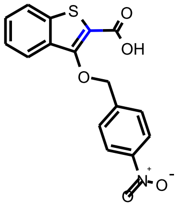 [*][c](:[*]):[*]	-0.247
ECFP_6	-182236392	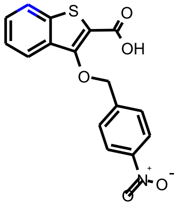 [*]:[cH]:[*]	-0.232

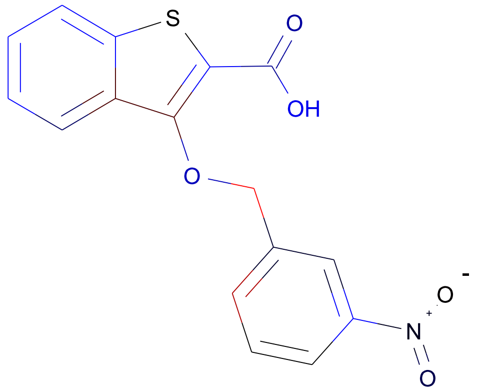

$C_{16}H_{11}NO_5S$

Molecular Weight: 329.32724

ALogP: 4.074

Rotatable Bonds: 5

Acceptors: 5

Donors: 1

Model Prediction

Prediction: 164.040

Unit: mg/kg_body_weight/day

Mahalanobis Distance: 11.806

Mahalanobis Distance p-value: 5.12e-005

Mahalanobis Distance: The Mahalanobis distance (MD) is a generalization of the Euclidean distance that accounts for correlations among the X properties. It is calculated as the distance to the center of the training data. The larger the MD, the less trustworthy the prediction.
Mahalanobis Distance p-value: The p-value gives the fraction of training data with an MD greater than or equal to the one for the given sample, assuming normally distributed data. The smaller the p-value, the less trustworthy the prediciton. For highly non-normal X properties (e.g., fingerprints), the MD p-value is wildly inaccurate.

Structural Similar Compounds

Name	646	Acifluorfen	470
Structure	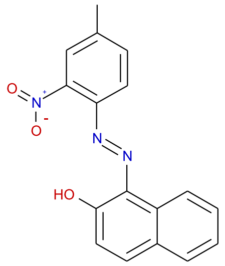	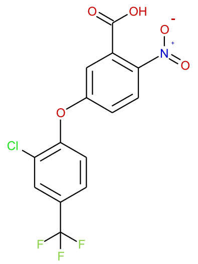	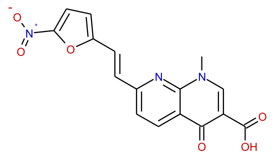
Actual Endpoint (-log C)	0.937339	3.40908	4.62839
Predicted Endpoint (-log C)	3.26294	3.10974	3.93264
Distance	0.626	0.661	0.685
Reference	CPDB	CPDB	CPDB

Model Applicability

Unknown features are fingerprint features in the query molecule, but not found in the training set.

1. All properties and OPS components are within expected ranges.
2. Unknown ECFP_2 feature: -1670580914: [*]C(=[*])[c]1:s:[*]:[*]:[c]:1[*]
3. Unknown ECFP_2 feature: 1895035276: [*]:[cH]:[c]1:s:[*]:[*]:[c]:1:[*]

Feature Contribution

Top features for positive contribution

Fingerprint	Bit/Smiles	Feature Structure	Score
ECFP_6	1559650422	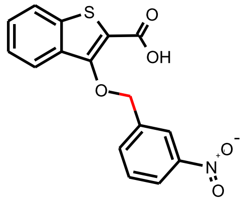 [*]C[*]	0.203

ECFP_6	-2024255407	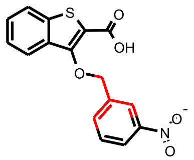<br>[*]C[c](:[cH]:[*]):[c<br>H]:[*]	0.172
ECFP_6	781519895	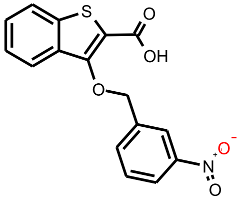<br>[*][O-]	0.080

Top Features for negative contribution

Fingerprint	Bit/Smiles	Feature Structure	Score
ECFP_6	1996767644	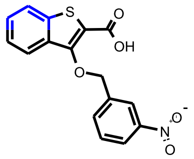<br>[*][c](:[*]):[cH]:[cH<br>]:[*]	-0.251
ECFP_6	642810091	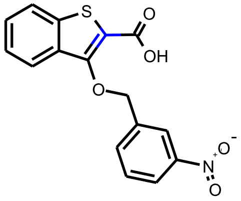<br>[*][c](:[*]):[*]	-0.247
ECFP_6	-182236392	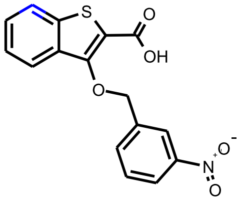<br>[*]:[cH]:[*]	-0.232

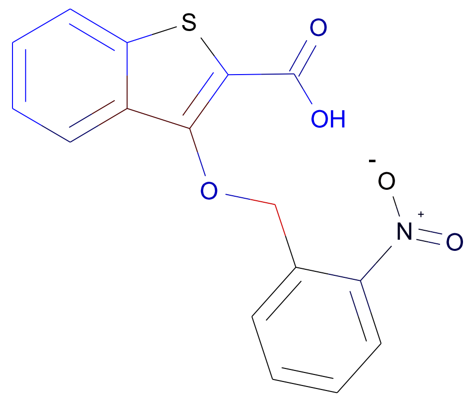

$C_{16}H_{11}NO_5S$
Molecular Weight: 329.32724
ALogP: 4.074
Rotatable Bonds: 5
Acceptors: 5
Donors: 1

Model Prediction

Prediction: 148.064
Unit: mg/kg_body_weight/day
Mahalanobis Distance: 11.015
Mahalanobis Distance p-value: 0.00137

Mahalanobis Distance: The Mahalanobis distance (MD) is a generalization of the Euclidean distance that accounts for correlations among the X properties. It is calculated as the distance to the center of the training data. The larger the MD, the less trustworthy the prediction.
Mahalanobis Distance p-value: The p-value gives the fraction of training data with an MD greater than or equal to the one for the given sample, assuming normally distributed data. The smaller the p-value, the less trustworthy the prediciton. For highly non-normal X properties (e.g., fingerprints), the MD p-value is wildly inaccurate.

Structural Similar Compounds

Name	646	Acifluorfen	470
Structure	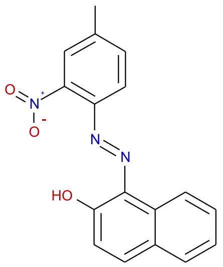	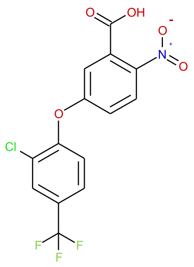	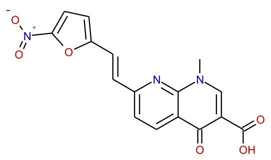
Actual Endpoint (-log C)	0.937339	3.40908	4.62839
Predicted Endpoint (-log C)	3.26294	3.10974	3.93264
Distance	0.619	0.653	0.684
Reference	CPDB	CPDB	CPDB

Model Applicability

Unknown features are fingerprint features in the query molecule, but not found in the training set.

1. All properties and OPS components are within expected ranges.
2. Unknown ECFP_2 feature: -1670580914: [*]C(=[*])[c]1:s:[*]:[*]:[c]:1[*]
3. Unknown ECFP_2 feature: 1895035276: [*]:[cH]:[c]1:s:[*]:[*]:[c]:1:[*]

Feature Contribution

Top features for positive contribution			
**Fingerprint**	**Bit/Smiles**	**Feature Structure**	**Score**
ECFP_6	1559650422	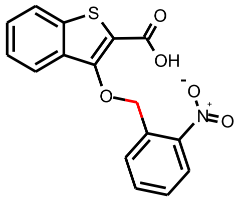 [*]C[*]	0.203

ECFP_6	781519895	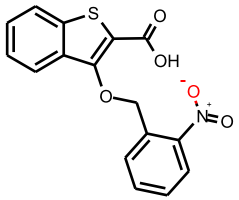 [*][O-]	0.080
ECFP_6	1333660716	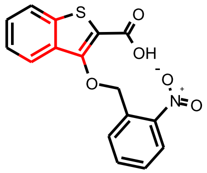 [*][c]1:[*]:[*]:[c](:[*]):[c]:1:[cH]:[*]	0.075

Top Features for negative contribution

Fingerprint	Bit/Smiles	Feature Structure	Score
ECFP_6	1996767644	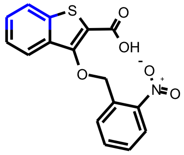 [*][c](:[*]):[cH]:[cH]:[*]	-0.251
ECFP_6	642810091	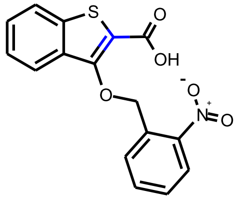 [*][c](:[*]):[*]	-0.247
ECFP_6	-182236392	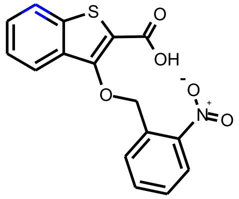 [*]:[cH]:[*]	-0.232

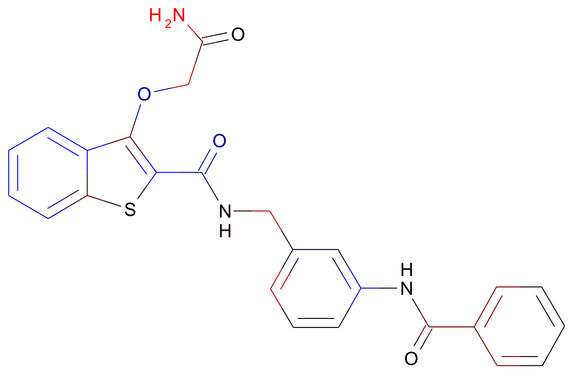

$C_{25}H_{21}N_3O_4S$
Molecular Weight: 459.51694
ALogP: 3.396
Rotatable Bonds: 8
Acceptors: 4
Donors: 3

Structural Similar Compounds

Name	Ochratoxin A	542	4-Chloro-6-(2,3-xylidino)-2-pyri-mi-dinylthio(N-b-hydroxy-ethyl) acetamide
Structure	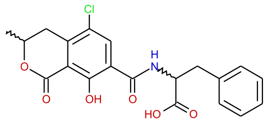	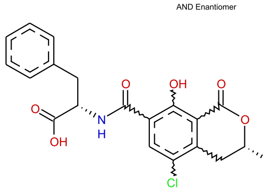	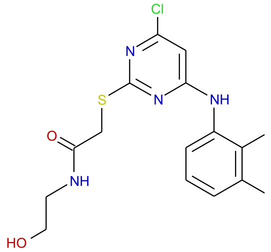
Actual Endpoint (-log C)	4.79932	4.79932	3.91517
Predicted Endpoint (-log C)	3.6353	3.6353	3.92186
Distance	0.853	0.853	0.912
Reference	CPDB	CPDB	CPDB

Model Prediction

Prediction: 14.134
Unit: mg/kg_body_weight/day
Mahalanobis Distance: 14.110
Mahalanobis Distance p-value: 1.63e-010

Mahalanobis Distance: The Mahalanobis distance (MD) is a generalization of the Euclidean distance that accounts for correlations among the X properties. It is calculated as the distance to the center of the training data. The larger the MD, the less trustworthy the prediction.
Mahalanobis Distance p-value: The p-value gives the fraction of training data with an MD greater than or equal to the one for the given sample, assuming normally distributed data. The smaller the p-value, the less trustworthy the prediciton. For highly non-normal X properties (e.g., fingerprints), the MD p-value is wildly inaccurate.

Model Applicability

Unknown features are fingerprint features in the query molecule, but not found in the training set.

1. All properties and OPS components are within expected ranges.
2. Unknown ECFP_2 feature: -1670580914: [*]C(=[*])[c]1:s:[*]:[*]:[c]:1[*]
3. Unknown ECFP_2 feature: 1895035276: [*]:[cH]:[c]1:s:[*]:[*]:[c]:1:[*]
4. Unknown ECFP_2 feature: -1686813061: [*]OCC(=[*])[*]

Feature Contribution

Top features for positive contribution

Fingerprint	Bit/Smiles	Feature Structure	Score
ECFP_6	1572579716	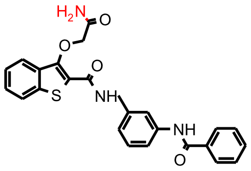 [*]N	0.225

ECFP_6	1559650422	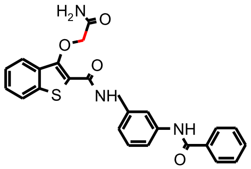 [*]C[*]	0.203
ECFP_6	-2024255407	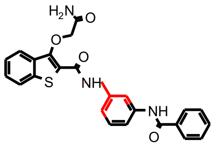 [*]C[c](:[cH]:[*]):[cH]:[*]	0.172
**Top Features for negative contribution**			
**Fingerprint**	**Bit/Smiles**	**Feature Structure**	**Score**
ECFP_6	1996767644	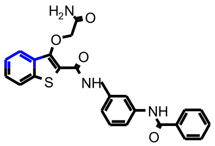 [*][c](:[*]):[cH]:[cH]:[*]	-0.251
ECFP_6	642810091	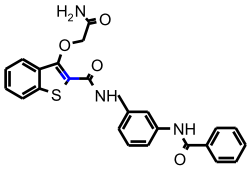 [*][c](:[*]):[*]	-0.247
ECFP_6	-182236392	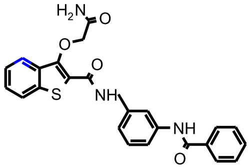 [*]:[cH]:[*]	-0.232

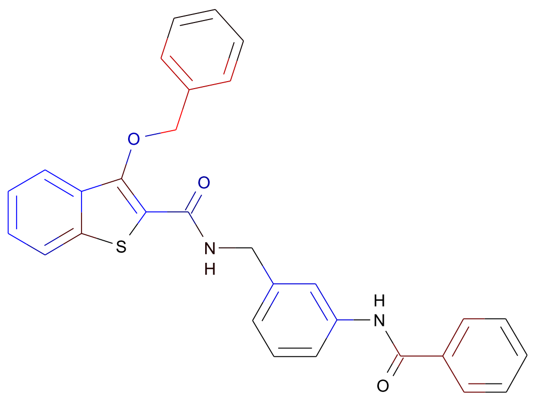

$C_{30}H_{24}N_2O_3S$
Molecular Weight: 492.58816
ALogP: 6.126
Rotatable Bonds: 8
Acceptors: 3
Donors: 2

Model Prediction

Prediction: 8.080
Unit: mg/kg_body_weight/day
Mahalanobis Distance: 13.675
Mahalanobis Distance p-value: 2.42e-009

Mahalanobis Distance: The Mahalanobis distance (MD) is a generalization of the Euclidean distance that accounts for correlations among the X properties. It is calculated as the distance to the center of the training data. The larger the MD, the less trustworthy the prediction.
Mahalanobis Distance p-value: The p-value gives the fraction of training data with an MD greater than or equal to the one for the given sample, assuming normally distributed data. The smaller the p-value, the less trustworthy the prediciton. For highly non-normal X properties (e.g., fingerprints), the MD p-value is wildly inaccurate.

Structural Similar Compounds

Name	Phenolphthalein	646	Tamoxifen citrate
Structure	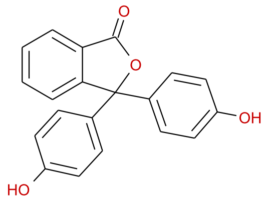	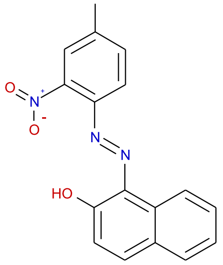	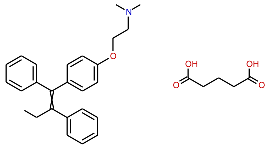
Actual Endpoint (-log C)	2.43468	0.937339	5.05965
Predicted Endpoint (-log C)	3.66084	3.26294	4.24168
Distance	0.998	1.001	1.010
Reference	CPDB	CPDB	CPDB

Model Applicability

Unknown features are fingerprint features in the query molecule, but not found in the training set.

1. All properties and OPS components are within expected ranges.
2. Unknown ECFP_2 feature: -1670580914: [*]C(=[*])[c]1:s:[*]:[*]:[c]:1[*]
3. Unknown ECFP_2 feature: 1895035276: [*]:[cH]:[c]1:s:[*]:[*]:[c]:1:[*]

Feature Contribution

Top features for positive contribution

Fingerprint	Bit/Smiles	Feature Structure	Score
ECFP_6	1559650422	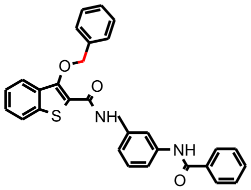 [*]C[*]	0.203

ECFP_6	-2024255407	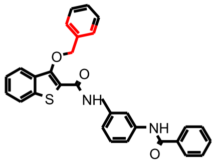 [*]C[c](:[cH]:[*]):[cH]:[*]	0.172
ECFP_6	-175146122	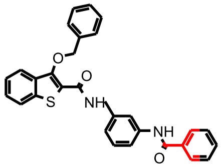 [*]C(=[*])[c](:[cH]:[*]):[cH]:[*]	0.107

Top Features for negative contribution

Fingerprint	Bit/Smiles	Feature Structure	Score
ECFP_6	1996767644	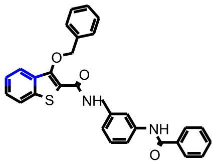 [*][c](:[*]):[cH]:[cH]:[*]	-0.251
ECFP_6	642810091	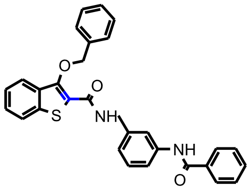 [*][c](:[*]):[*]	-0.247
ECFP_6	-182236392	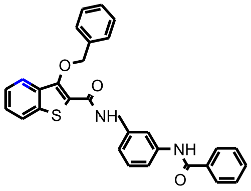 [*]:[cH]:[*]	-0.232

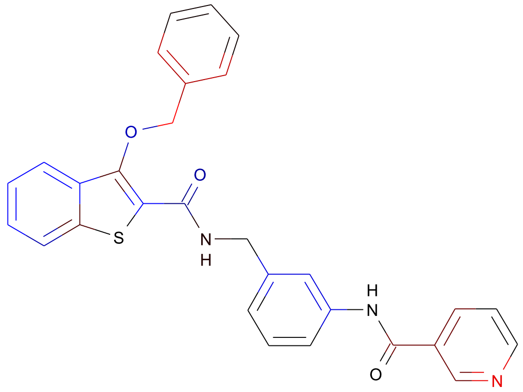

$C_{29}H_{23}N_3O_3S$
Molecular Weight: 493.57622
ALogP: 4.976
Rotatable Bonds: 8
Acceptors: 4
Donors: 2

Model Prediction

Prediction: 6.036
Unit: mg/kg_body_weight/day
Mahalanobis Distance: 13.658
Mahalanobis Distance p-value: 2.69e-009

Mahalanobis Distance: The Mahalanobis distance (MD) is a generalization of the Euclidean distance that accounts for correlations among the X properties. It is calculated as the distance to the center of the training data. The larger the MD, the less trustworthy the prediction.
Mahalanobis Distance p-value: The p-value gives the fraction of training data with an MD greater than or equal to the one for the given sample, assuming normally distributed data. The smaller the p-value, the less trustworthy the prediciton. For highly non-normal X properties (e.g., fingerprints), the MD p-value is wildly inaccurate.

Structural Similar Compounds

Name	Tamoxifen citrate	646	Phenolphthalein
Structure	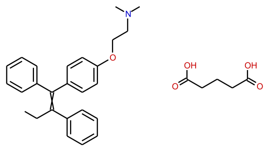	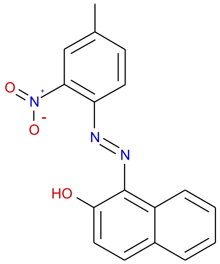	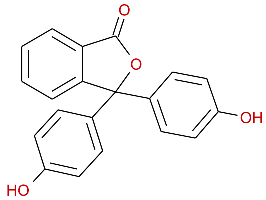
Actual Endpoint (-log C)	5.05965	0.937339	2.43468
Predicted Endpoint (-log C)	4.24168	3.26294	3.66084
Distance	0.982	0.993	0.993
Reference	CPDB	CPDB	CPDB

Model Applicability

Unknown features are fingerprint features in the query molecule, but not found in the training set.

1. All properties and OPS components are within expected ranges.
2. Unknown ECFP_2 feature: -1670580914: [*]C(=[*])[c]1:s:[*]:[*]:[c]:1[*]
3. Unknown ECFP_2 feature: 1895035276: [*]:[cH]:[c]1:s:[*]:[*]:[c]:1:[*]

Feature Contribution

Top features for positive contribution

Fingerprint	Bit/Smiles	Feature Structure	Score
ECFP_6	655739385	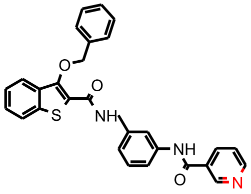 [*]:n:[*]	0.229

ECFP_6	1559650422	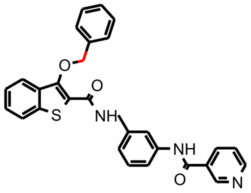 [*]C[*]	0.203
ECFP_6	-2024255407	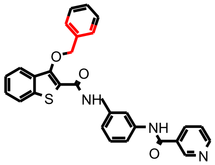 [*]C[c](:[cH]:[*]):[cH]:[*]	0.172
**Top Features for negative contribution**			
**Fingerprint**	**Bit/Smiles**	**Feature Structure**	**Score**
ECFP_6	1996767644	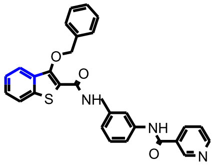 [*][c](:[*]):[cH]:[cH]:[*]	-0.251
ECFP_6	642810091	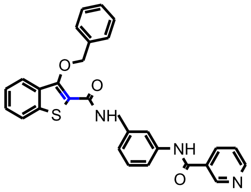 [*][c](:[*]):[*]	-0.247
ECFP_6	-182236392	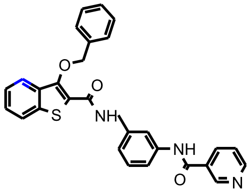 [*]:[cH]:[*]	-0.232

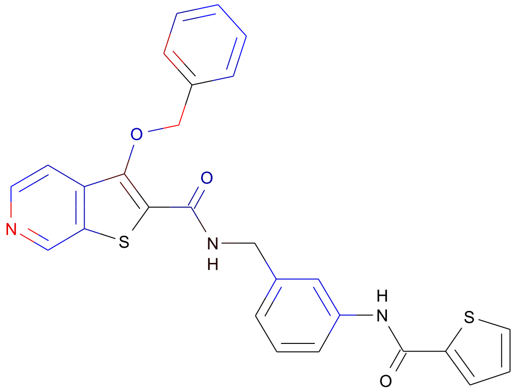

$C_{27}H_{21}N_3O_3S_2$
Molecular Weight: 499.60394
ALogP: 4.929
Rotatable Bonds: 8
Acceptors: 4
Donors: 2

Model Prediction

Prediction: 9.075
Unit: mg/kg_body_weight/day
Mahalanobis Distance: 13.530
Mahalanobis Distance p-value: 5.77e-009

Mahalanobis Distance: The Mahalanobis distance (MD) is a generalization of the Euclidean distance that accounts for correlations among the X properties. It is calculated as the distance to the center of the training data. The larger the MD, the less trustworthy the prediction.
Mahalanobis Distance p-value: The p-value gives the fraction of training data with an MD greater than or equal to the one for the given sample, assuming normally distributed data. The smaller the p-value, the less trustworthy the prediciton. For highly non-normal X properties (e.g., fingerprints), the MD p-value is wildly inaccurate.

Structural Similar Compounds

Name	Tamoxifen citrate	646	Ochratoxin A
Structure	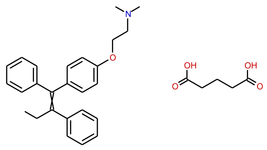	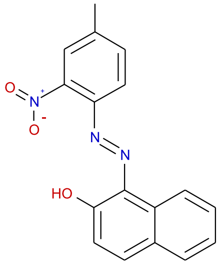	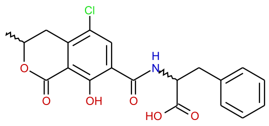
Actual Endpoint (-log C)	5.05965	0.937339	4.79932
Predicted Endpoint (-log C)	4.24168	3.26294	3.6353
Distance	1.024	1.035	1.043
Reference	CPDB	CPDB	CPDB

Model Applicability

Unknown features are fingerprint features in the query molecule, but not found in the training set.

1. All properties and OPS components are within expected ranges.
2. Unknown ECFP_2 feature: 1895035276: [*]:[cH]:[c]1:s:[*]:[*]:[c]:1:[*]
3. Unknown ECFP_2 feature: -1670580914: [*]C(=[*])[c]1:s:[*]:[*]:[c]:1[*]
4. Unknown ECFP_2 feature: 1898414610: [*]C(=[*])[c]1:[cH]:[*]:[*]:s:1
5. Unknown ECFP_2 feature: 1996740348: [*]1:[*]:s:[cH]:[cH]:1

Feature Contribution

Top features for positive contribution

Fingerprint	Bit/Smiles	Feature Structure	Score
ECFP_6	655739385	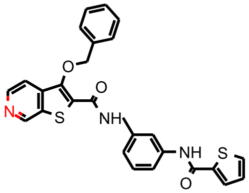 [*]:n:[*]	0.229

ECFP_6	1559650422	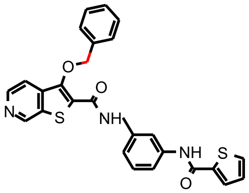 [*]C[*]	0.203
ECFP_6	-2024255407	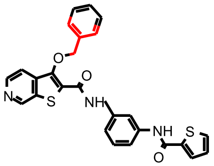 [*]C[c](:[cH]:[*]):[cH]:[*]	0.172
**Top Features for negative contribution**			
**Fingerprint**	**Bit/Smiles**	**Feature Structure**	**Score**
ECFP_6	1996767644	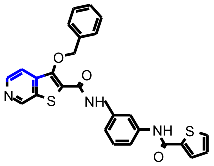 [*][c](:[*]):[cH]:[cH]:[*]	-0.251
ECFP_6	642810091	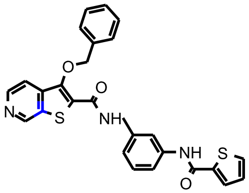 [*][c](:[*]):[*]	-0.247
ECFP_6	-182236392	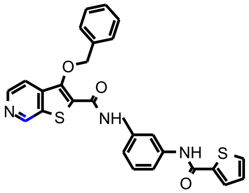 [*]:[cH]:[*]	-0.232

Co-crystallized ligand

TOPKAT_Carcinogenic_Potency_TD50_Mouse

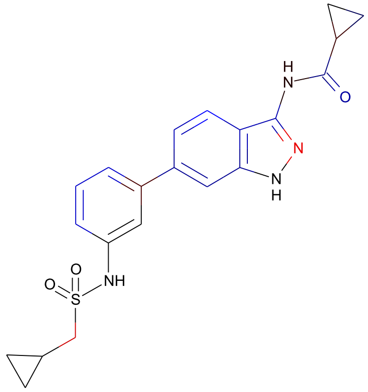

$C_{21}H_{22}N_4O_3S$
Molecular Weight: 410.48938
ALogP: 3.14
Rotatable Bonds: 7
Acceptors: 4
Donors: 3

Model Prediction

Prediction: 5.812
Unit: mg/kg_body_weight/day
Mahalanobis Distance: 13.011
Mahalanobis Distance p-value: 1.15e-007

Mahalanobis Distance: The Mahalanobis distance (MD) is a generalization of the Euclidean distance that accounts for correlations among the X properties. It is calculated as the distance to the center of the training data. The larger the MD, the less trustworthy the prediction.
Mahalanobis Distance p-value: The p-value gives the fraction of training data with an MD greater than or equal to the one for the given sample, assuming normally distributed data. The smaller the p-value, the less trustworthy the prediciton. For highly non-normal X properties (e.g., fingerprints), the MD p-value is wildly inaccurate.

Structural Similar Compounds

Name	Ochratoxin A	542	Phenolphthalein
Structure	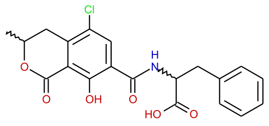	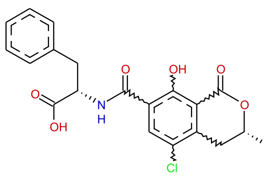	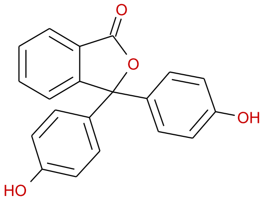
Actual Endpoint (-log C)	4.79932	4.79932	2.43468
Predicted Endpoint (-log C)	3.6353	3.6353	3.66084
Distance	0.778	0.778	0.844
Reference	CPDB	CPDB	CPDB

Model Applicability

Unknown features are fingerprint features in the query molecule, but not found in the training set.

1. All properties and OPS components are within expected ranges.
2. Unknown ECFP_2 feature: -857146788: [*]C(=[*])C1CC1
3. Unknown ECFP_2 feature: 268744321: [*]CS(=O)(=O)N[*]
4. Unknown ECFP_2 feature: -1341194584: [*]S(=[*])(=[*])CC1[*][*]1
5. Unknown ECFP_2 feature: -1795620553: [*]CC1CC1

Feature Contribution

Top features for positive contribution

Fingerprint	Bit/Smiles	Feature Structure	Score
ECFP_6	655739385	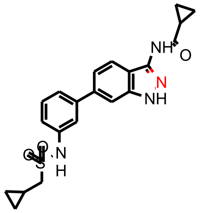 [*]:n:[*]	0.229

ECFP_6	1559650422	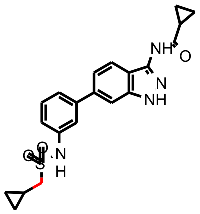 [*]C[*]	0.203
ECFP_6	1333660716	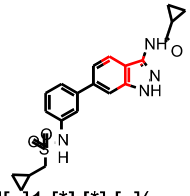 [*][c]1:[*]:[*]:[c](:[*]):[c]:1:[cH]:[*]	0.075
**Top Features for negative contribution**			
**Fingerprint**	**Bit/Smiles**	**Feature Structure**	**Score**
ECFP_6	1996767644	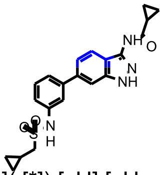 [*][c](:[*]):[cH]:[cH]:[*]	-0.251
ECFP_6	642810091	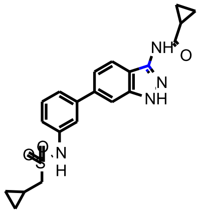 [*][c](:[*]):[*]	-0.247
ECFP_6	-182236392	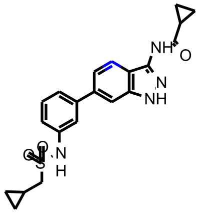 [*]:[cH]:[*]	-0.232

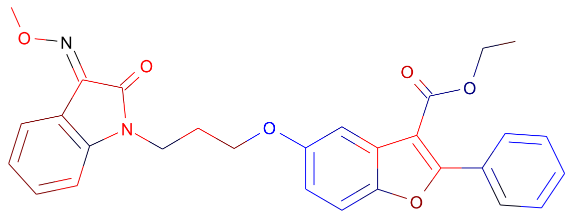

$C_{29}H_{26}N_2O_6$
Molecular Weight: 498.52653
ALogP: 4.994
Rotatable Bonds: 10
Acceptors: 6
Donors: 0

Model Prediction

Prediction: 0.495
Unit: mg/kg_body_weight/day
Mahalanobis Distance: 20.358
Mahalanobis Distance p-value: 1.58e-030

Mahalanobis Distance: The Mahalanobis distance (MD) is a generalization of the Euclidean distance that accounts for correlations among the X properties. It is calculated as the distance to the center of the training data. The larger the MD, the less trustworthy the prediction.
Mahalanobis Distance p-value: The p-value gives the fraction of training data with an MD greater than or equal to the one for the given sample, assuming normally distributed data. The smaller the p-value, the less trustworthy the prediciton. For highly non-normal X properties (e.g., fingerprints), the MD p-value is wildly inaccurate.

Structural Similar Compounds

Name	223	426	C.I. direct brown 95
Structure	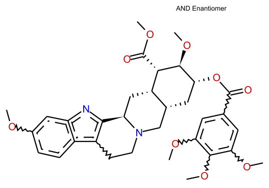	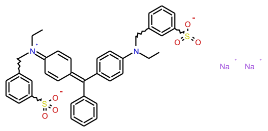	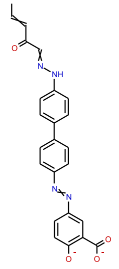
Actual Endpoint (-log C)	6.29867	2.04218	5.31387
Predicted Endpoint (-log C)	7.5657	3.24288	4.30266
Distance	0.807	0.817	0.820
Reference	CPDB	CPDB	CPDB

Model Applicability

Unknown features are fingerprint features in the query molecule, but not found in the training set.

1. All properties and OPS components are within expected ranges.

Feature Contribution

Top features for positive contribution

Fingerprint	Bit/Smiles	Feature Structure	Score
FCFP_6	136627117	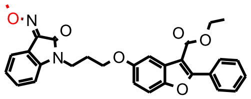 [*]OC	0.690

FCFP_6	-1861645784	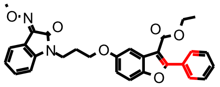 [*]:[cH]:[c](:[cH]:[*])[c](:[*]):[*]	0.359
FCFP_6	565998553	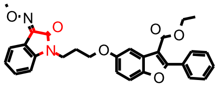 [*]N1[*]:[*]C(=[*])C1 =O	0.357

Top Features for negative contribution

Fingerprint	Bit/Smiles	Feature Structure	Score
FCFP_6	991735244	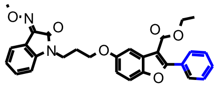 [*][c]1:[*]:[cH]:[cH] :[cH]:[cH]:1	-0.422
FCFP_6	-2093839777	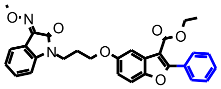 [*][c]1:[cH]:[cH]:[cH]:[cH]:[cH]:1	-0.378
FCFP_6	16	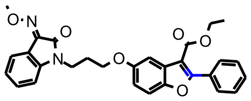 [*][c](:[*]):[*]	-0.354

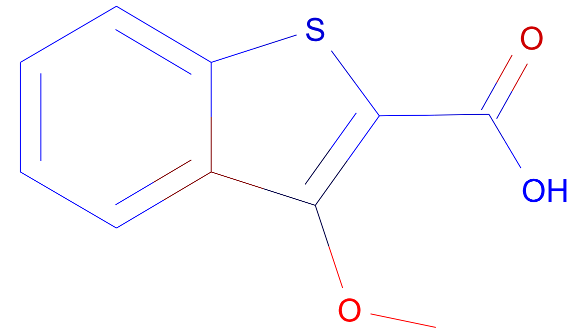

$C_{10}H_8O_3S$
Molecular Weight: 208.23372
ALogP: 2.596
Rotatable Bonds: 2
Acceptors: 3
Donors: 1

Model Prediction

Prediction: 57.225
Unit: mg/kg_body_weight/day
Mahalanobis Distance: 14.492
Mahalanobis Distance p-value: 9.82e-010

Mahalanobis Distance: The Mahalanobis distance (MD) is a generalization of the Euclidean distance that accounts for correlations among the X properties. It is calculated as the distance to the center of the training data. The larger the MD, the less trustworthy the prediction.
Mahalanobis Distance p-value: The p-value gives the fraction of training data with an MD greater than or equal to the one for the given sample, assuming normally distributed data. The smaller the p-value, the less trustworthy the prediciton. For highly non-normal X properties (e.g., fingerprints), the MD p-value is wildly inaccurate.

Structural Similar Compounds

Name	2-Methoxy-3-aminodibenzofuran	Carbaryl	269
Structure	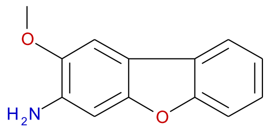	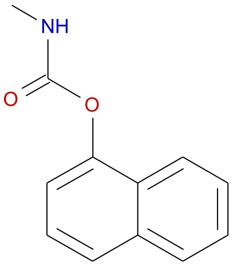	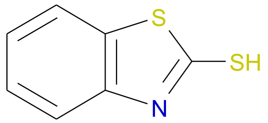
Actual Endpoint (-log C)	3.86645	4.15445	2.68681
Predicted Endpoint (-log C)	4.3205	3.66654	3.37119
Distance	0.524	0.553	0.554
Reference	CPDB	CPDB	CPDB

Model Applicability

Unknown features are fingerprint features in the query molecule, but not found in the training set.

1. All properties and OPS components are within expected ranges.

Feature Contribution

Top features for positive contribution

Fingerprint	Bit/Smiles	Feature Structure	Score
FCFP_6	136627117	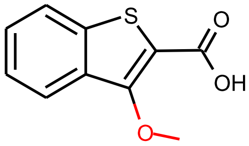 [*]OC	0.690

FCFP_6	1	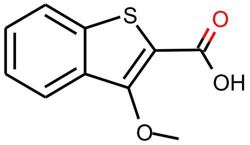 [*]=O	0.234
FCFP_6	-1977641857	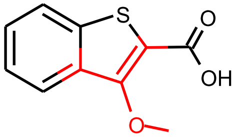 [*][c]1:[*]:[*]:[c](:[*]):[c]:1OC	0.123
**Top Features for negative contribution**			
**Fingerprint**	**Bit/Smiles**	**Feature Structure**	**Score**
FCFP_6	991735244	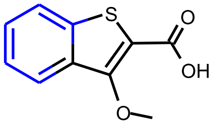 [*][c]1:[*]:[cH]:[cH]:[cH]:[cH]:1	-0.422
FCFP_6	7	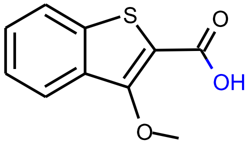 [*]O	-0.372
FCFP_6	16	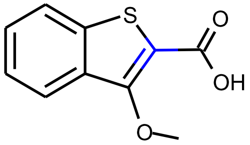 [*][c](:[*]):[*]	-0.354

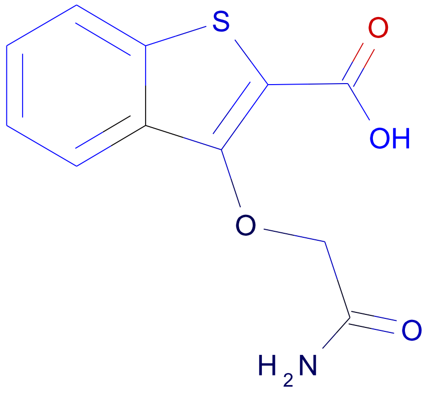

$C_{11}H_9NO_4S$
Molecular Weight: 251.25846
ALogP: 1.45
Rotatable Bonds: 4
Acceptors: 4
Donors: 2

Model Prediction

Prediction: 735.305
Unit: mg/kg_body_weight/day
Mahalanobis Distance: 16.647
Mahalanobis Distance p-value: 1.96e-016

Mahalanobis Distance: The Mahalanobis distance (MD) is a generalization of the Euclidean distance that accounts for correlations among the X properties. It is calculated as the distance to the center of the training data. The larger the MD, the less trustworthy the prediction.
Mahalanobis Distance p-value: The p-value gives the fraction of training data with an MD greater than or equal to the one for the given sample, assuming normally distributed data. The smaller the p-value, the less trustworthy the prediciton. For highly non-normal X properties (e.g., fingerprints), the MD p-value is wildly inaccurate.

Structural Similar Compounds

Name	2-Hydrazino-4-(p-nitrophenyl) thiazole	Dapsone	AF-2 s
Structure	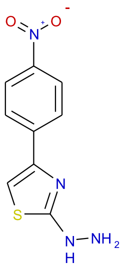	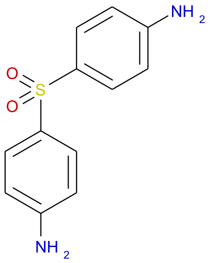	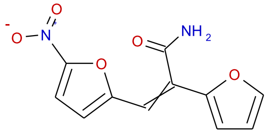
Actual Endpoint (-log C)	4.86687	4.04473	3.92644
Predicted Endpoint (-log C)	5.14723	4.05717	3.4842
Distance	0.551	0.564	0.583
Reference	CPDB	CPDB	CPDB

Model Applicability

Unknown features are fingerprint features in the query molecule, but not found in the training set.

1. All properties and OPS components are within expected ranges.

Feature Contribution

Top features for positive contribution			
**Fingerprint**	**Bit/Smiles**	**Feature Structure**	**Score**
FCFP_6	1	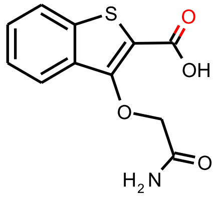 [*]=O	0.234

FCFP_6	-1272768868	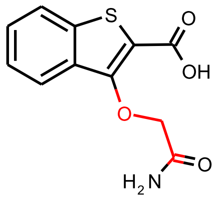 [*]CCO[*]	0.127
FCFP_6	307419094	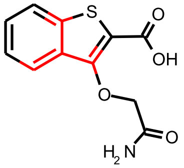 [*][c]1:[*]:[*]:[c](:[*]):[c]:1:[cH]:[*]	0.121

Top Features for negative contribution

Fingerprint	Bit/Smiles	Feature Structure	Score
FCFP_6	991735244	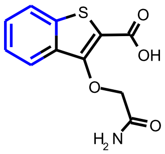 [*][c]1:[*]:[cH]:[cH]:[cH]:[cH]:1	-0.422
FCFP_6	7	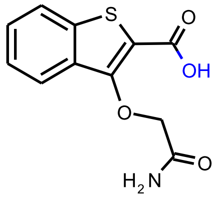 [*]O	-0.372
FCFP_6	16	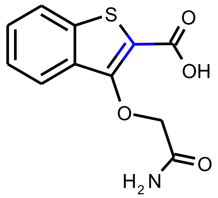 [*][c](:[*]):[*]	-0.354

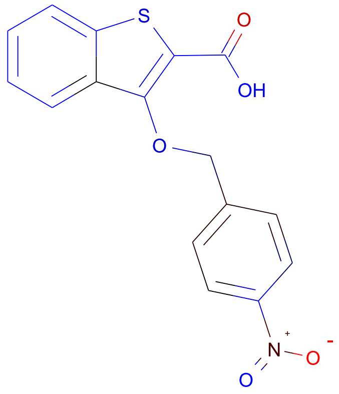

$C_{16}H_{11}NO_5S$
Molecular Weight: 329.32724
ALogP: 4.074
Rotatable Bonds: 5
Acceptors: 5
Donors: 1

Model Prediction

Prediction: 79.533
Unit: mg/kg_body_weight/day
Mahalanobis Distance: 17.822
Mahalanobis Distance p-value: 1.33e-020

Mahalanobis Distance: The Mahalanobis distance (MD) is a generalization of the Euclidean distance that accounts for correlations among the X properties. It is calculated as the distance to the center of the training data. The larger the MD, the less trustworthy the prediction.
Mahalanobis Distance p-value: The p-value gives the fraction of training data with an MD greater than or equal to the one for the given sample, assuming normally distributed data. The smaller the p-value, the less trustworthy the prediciton. For highly non-normal X properties (e.g., fingerprints), the MD p-value is wildly inaccurate.

Structural Similar Compounds

Name	4-(2-Hydroxyethylamino)-2-(5-nitro-2-thienyl)quinazoline	646	Omeprazole
Structure	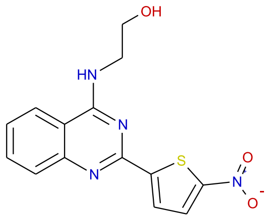	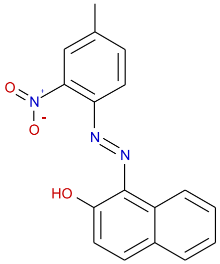	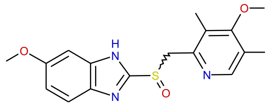
Actual Endpoint (-log C)	5.22831	2.41938	3.4628
Predicted Endpoint (-log C)	4.31976	3.77987	4.7324
Distance	0.553	0.558	0.581
Reference	CPDB	CPDB	CPDB

Model Applicability

Unknown features are fingerprint features in the query molecule, but not found in the training set.

1. All properties and OPS components are within expected ranges.

Feature Contribution

Top features for positive contribution

Fingerprint	Bit/Smiles	Feature Structure	Score
FCFP_6	5	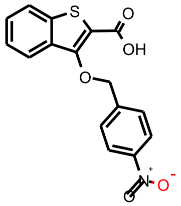 [*][O-]	0.431

FCFP_6	8	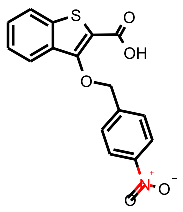 [*][N+](=[*])[*]	0.336
FCFP_6	1	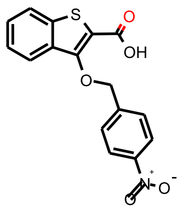 [*]=O	0.234

Top Features for negative contribution

Fingerprint	Bit/Smiles	Feature Structure	Score
FCFP_6	991735244	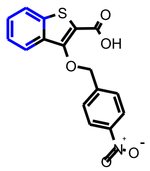 [*][c]1:[*]:[cH]:[cH]:[cH]:[cH]:1	-0.422
FCFP_6	7	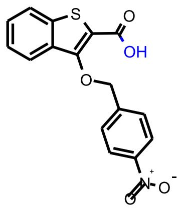 [*]O	-0.372
FCFP_6	16	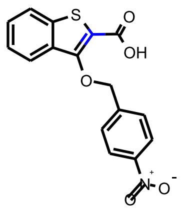 [*][c](:[*]):[*]	-0.354

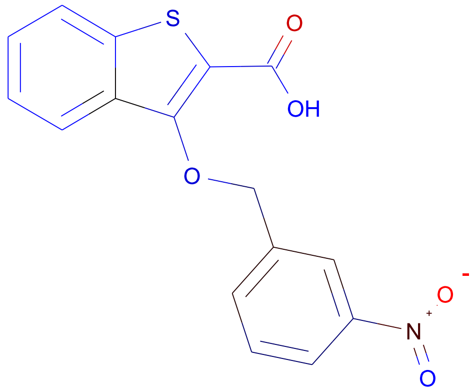

$C_{16}H_{11}NO_5S$
Molecular Weight: 329.32724
ALogP: 4.074
Rotatable Bonds: 5
Acceptors: 5
Donors: 1

Model Prediction

Prediction: 79.533
Unit: mg/kg_body_weight/day
Mahalanobis Distance: 17.822
Mahalanobis Distance p-value: 1.33e-020

Mahalanobis Distance: The Mahalanobis distance (MD) is a generalization of the Euclidean distance that accounts for correlations among the X properties. It is calculated as the distance to the center of the training data. The larger the MD, the less trustworthy the prediction.
Mahalanobis Distance p-value: The p-value gives the fraction of training data with an MD greater than or equal to the one for the given sample, assuming normally distributed data. The smaller the p-value, the less trustworthy the prediciton. For highly non-normal X properties (e.g., fingerprints), the MD p-value is wildly inaccurate.

Structural Similar Compounds

Name	646	4-(2-Hydroxyethylamino)-2-(5-nitro-2-thienyl)quinazoline	Omeprazole
Structure	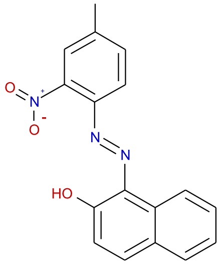	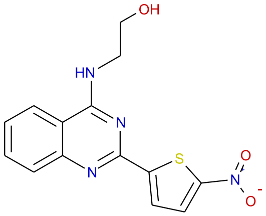	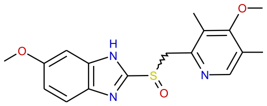
Actual Endpoint (-log C)	2.41938	5.22831	3.4628
Predicted Endpoint (-log C)	3.77987	4.31976	4.7324
Distance	0.555	0.556	0.583
Reference	CPDB	CPDB	CPDB

Model Applicability

Unknown features are fingerprint features in the query molecule, but not found in the training set.

1. All properties and OPS components are within expected ranges.

Feature Contribution

Top features for positive contribution			
**Fingerprint**	**Bit/Smiles**	**Feature Structure**	**Score**
FCFP_6	5	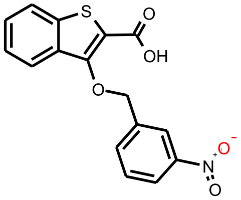 [*][O-]	0.431

FCFP_6	8	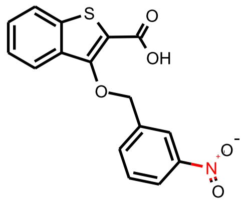 [*][N+](=[*])[*]	0.336
FCFP_6	1	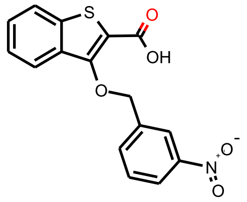 [*]=O	0.234

Top Features for negative contribution

Fingerprint	Bit/Smiles	Feature Structure	Score
FCFP_6	991735244	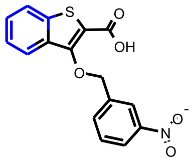 [*][c]1:[*]:[cH]:[cH]:[cH]:[cH]:1	-0.422
FCFP_6	7	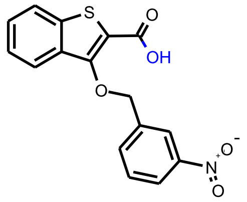 [*]O	-0.372
FCFP_6	16	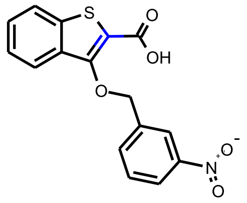 [*][c](:[*]):[*]	-0.354

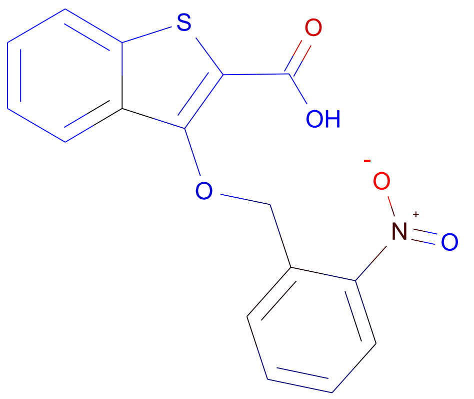

$C_{16}H_{11}NO_5S$
Molecular Weight: 329.32724
ALogP: 4.074
Rotatable Bonds: 5
Acceptors: 5
Donors: 1

Model Prediction

Prediction: 82.668
Unit: mg/kg_body_weight/day
Mahalanobis Distance: 18.091
Mahalanobis Distance p-value: 1.33e-021

Mahalanobis Distance: The Mahalanobis distance (MD) is a generalization of the Euclidean distance that accounts for correlations among the X properties. It is calculated as the distance to the center of the training data. The larger the MD, the less trustworthy the prediction.
Mahalanobis Distance p-value: The p-value gives the fraction of training data with an MD greater than or equal to the one for the given sample, assuming normally distributed data. The smaller the p-value, the less trustworthy the prediciton. For highly non-normal X properties (e.g., fingerprints), the MD p-value is wildly inaccurate.

Structural Similar Compounds

Name	4-(2-Hydroxyethylamino)-2-(5-nitro-2-thienyl)quinazoline	646	Omeprazole
Structure	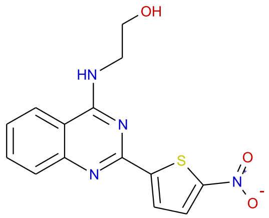	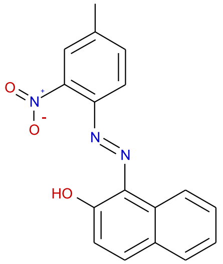	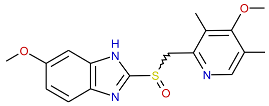
Actual Endpoint (-log C)	5.22831	2.41938	3.4628
Predicted Endpoint (-log C)	4.31976	3.77987	4.7324
Distance	0.555	0.561	0.582
Reference	CPDB	CPDB	CPDB

Model Applicability

Unknown features are fingerprint features in the query molecule, but not found in the training set.

1. All properties and OPS components are within expected ranges.

Feature Contribution

Top features for positive contribution

Fingerprint	Bit/Smiles	Feature Structure	Score
FCFP_6	5	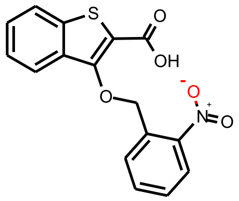 [*][O-]	0.431

FCFP_6	8	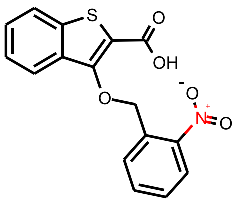 [*][N+](=[*])[*]	0.336
FCFP_6	1	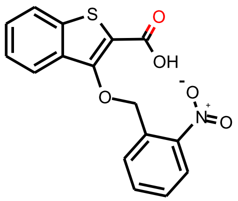 [*]=O	0.234
**Top Features for negative contribution**			
**Fingerprint**	**Bit/Smiles**	**Feature Structure**	**Score**
FCFP_6	991735244	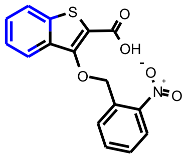 [*][c]1:[*]:[cH]:[cH]:[cH]:[cH]:1	-0.422
FCFP_6	7	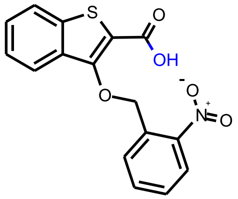 [*]O	-0.372
FCFP_6	16	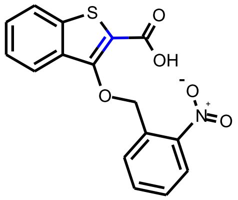 [*][c](:[*]):[*]	-0.354

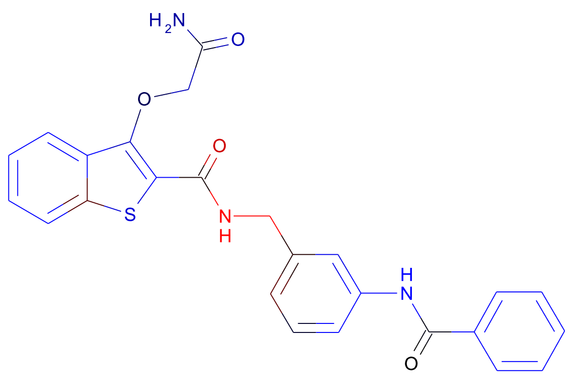

$C_{25}H_{21}N_3O_4S$
Molecular Weight: 459.51694
ALogP: 3.396
Rotatable Bonds: 8
Acceptors: 4
Donors: 3

Model Prediction

Prediction: 151.999
Unit: mg/kg_body_weight/day
Mahalanobis Distance: 20.452
Mahalanobis Distance p-value: 6.46e-031

Mahalanobis Distance: The Mahalanobis distance (MD) is a generalization of the Euclidean distance that accounts for correlations among the X properties. It is calculated as the distance to the center of the training data. The larger the MD, the less trustworthy the prediction.
Mahalanobis Distance p-value: The p-value gives the fraction of training data with an MD greater than or equal to the one for the given sample, assuming normally distributed data. The smaller the p-value, the less trustworthy the prediciton. For highly non-normal X properties (e.g., fingerprints), the MD p-value is wildly inaccurate.

Structural Similar Compounds

Name	Fluvastatin	913	4-Bis(2-hydroxyethyl)amino-2-(5-nitro-2-thienyl)quinazoline
Structure	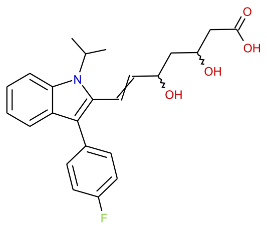	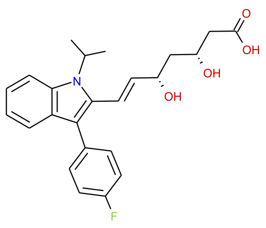	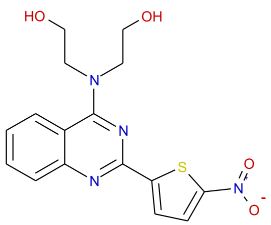
Actual Endpoint (-log C)	3.51742	3.51742	5.05984
Predicted Endpoint (-log C)	5.41573	5.41573	4.23808
Distance	0.704	0.704	0.726
Reference	CPDB	CPDB	CPDB

Model Applicability

Unknown features are fingerprint features in the query molecule, but not found in the training set.

1. All properties and OPS components are within expected ranges.

Feature Contribution

Top features for positive contribution

Fingerprint	Bit/Smiles	Feature Structure	Score
FCFP_6	1	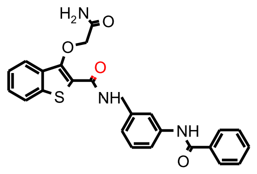 [*]=O	0.234

FCFP_6	-885550502	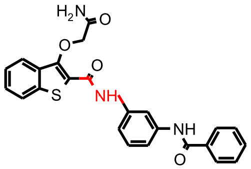<br>[*]CNC(=[*])[*]	0.229
FCFP_6	203677720	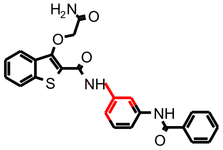<br>[*]C(=[*])[c]1:[c]([*]):[*]:[*]:[c]:1:[*]	0.137

Top Features for negative contribution

Fingerprint	Bit/Smiles	Feature Structure	Score
FCFP_6	991735244	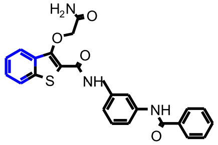<br>[*][c]1:[*]:[cH]:[cH]:[cH]:[cH]:1	-0.422
FCFP_6	-2093839777	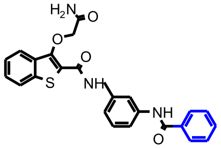<br>[*][c]1:[cH]:[cH]:[cH]:[cH]:[cH]:1	-0.378
FCFP_6	16	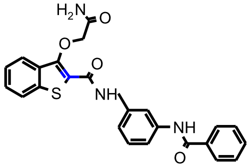<br>[*][c](:[*]):[*]	-0.354

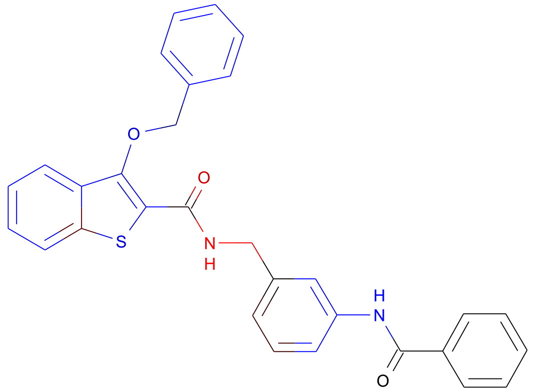

$C_{30}H_{24}N_2O_3S$
Molecular Weight: 492.58816
ALogP: 6.126
Rotatable Bonds: 8
Acceptors: 3
Donors: 2

Model Prediction

Prediction: 27.572
Unit: mg/kg_body_weight/day
Mahalanobis Distance: 19.733
Mahalanobis Distance p-value: 5.53e-028

Mahalanobis Distance: The Mahalanobis distance (MD) is a generalization of the Euclidean distance that accounts for correlations among the X properties. It is calculated as the distance to the center of the training data. The larger the MD, the less trustworthy the prediction.
Mahalanobis Distance p-value: The p-value gives the fraction of training data with an MD greater than or equal to the one for the given sample, assuming normally distributed data. The smaller the p-value, the less trustworthy the prediciton. For highly non-normal X properties (e.g., fingerprints), the MD p-value is wildly inaccurate.

Structural Similar Compounds

Name	913	Fluvastatin	Indomethacin
Structure	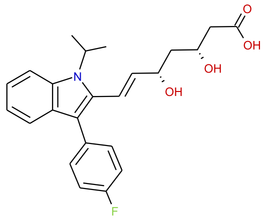	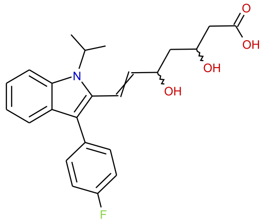	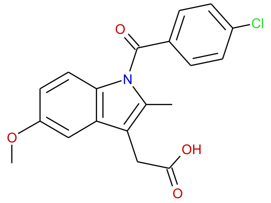
Actual Endpoint (-log C)	3.51742	3.51742	5.49293
Predicted Endpoint (-log C)	5.41573	5.41573	4.9569
Distance	0.829	0.829	0.905
Reference	CPDB	CPDB	CPDB

Model Applicability

Unknown features are fingerprint features in the query molecule, but not found in the training set.

1. All properties and OPS components are within expected ranges.

Feature Contribution

Top features for positive contribution			
**Fingerprint**	**Bit/Smiles**	**Feature Structure**	**Score**
FCFP_6	1	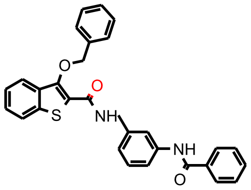 [*]=O	0.234

FCFP_6	-885550502	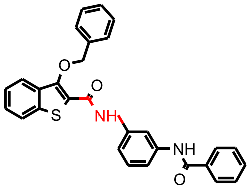 [*]CNC(=[*])[*]	0.229
FCFP_6	203677720	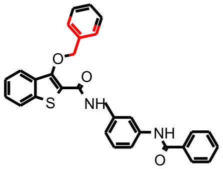 [*]C(=[*])[c]1:[c]([*]):[*]:[*]:[c]:1:[*]	0.137

Top Features for negative contribution

Fingerprint	Bit/Smiles	Feature Structure	Score
FCFP_6	991735244	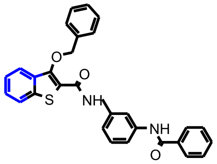 [*][c]1:[*]:[cH]:[cH] :[cH]:[cH]:1	-0.422
FCFP_6	-2093839777	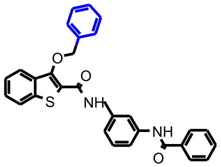 [*][c]1:[cH]:[cH]:[cH]:[cH]:[cH]:1	-0.378
FCFP_6	16	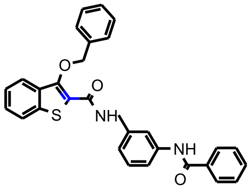 [*][c](:[*]):[*]	-0.354

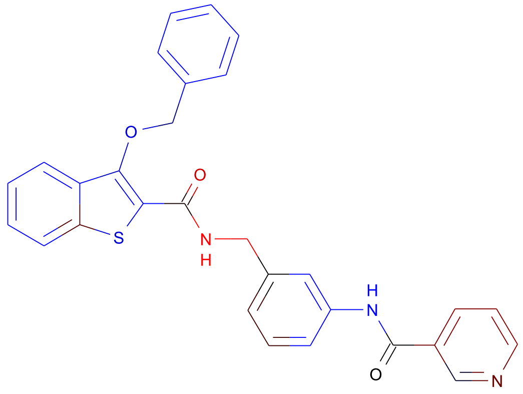

$C_{29}H_{23}N_3O_3S$
Molecular Weight: 493.57622
ALogP: 4.976
Rotatable Bonds: 8
Acceptors: 4
Donors: 2

Model Prediction

Prediction: 25.767
Unit: mg/kg_body_weight/day
Mahalanobis Distance: 21.213
Mahalanobis Distance p-value: 4.23e-034

Mahalanobis Distance: The Mahalanobis distance (MD) is a generalization of the Euclidean distance that accounts for correlations among the X properties. It is calculated as the distance to the center of the training data. The larger the MD, the less trustworthy the prediction.
Mahalanobis Distance p-value: The p-value gives the fraction of training data with an MD greater than or equal to the one for the given sample, assuming normally distributed data. The smaller the p-value, the less trustworthy the prediciton. For highly non-normal X properties (e.g., fingerprints), the MD p-value is wildly inaccurate.

Structural Similar Compounds

Name	913	Fluvastatin	C.I. direct brown 95
Structure	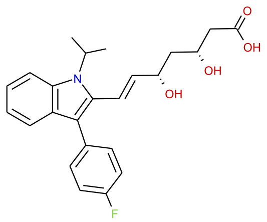	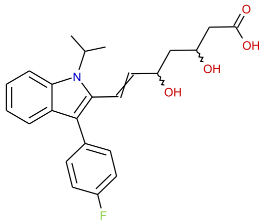	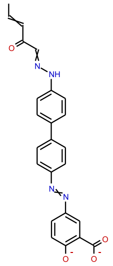
Actual Endpoint (-log C)	3.51742	3.51742	5.31387
Predicted Endpoint (-log C)	5.41573	5.41573	4.30266
Distance	0.818	0.818	0.893
Reference	CPDB	CPDB	CPDB

Model Applicability

Unknown features are fingerprint features in the query molecule, but not found in the training set.

1. OPS PC6 out of range. Value: 7.0182. Training min, max, SD, explained variance: -5.5832, 6.4847, 1.973, 0.0374.

Feature Contribution

Top features for positive contribution			
**Fingerprint**	**Bit/Smiles**	**Feature Structure**	**Score**
FCFP_6	1	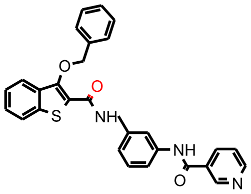 [*]=O	0.234

FCFP_6	-885550502	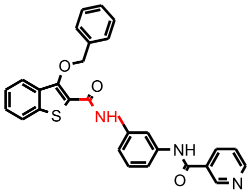 [*]CNC(=[*])[*]	0.229
FCFP_6	730557100	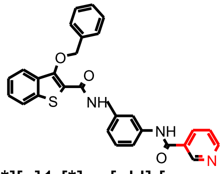 [*][c]1:[*]:n:[cH]:[cH]:[cH]:1	0.141

Top Features for negative contribution

**Fingerprint**	**Bit/Smiles**	**Feature Structure**	**Score**
FCFP_6	991735244	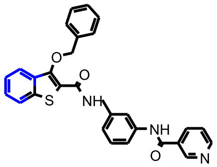 [*][c]1:[*]:[cH]:[cH]:[cH]:[cH]:1	-0.422
FCFP_6	-2093839777	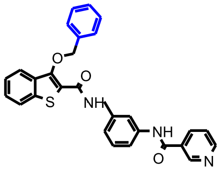 [*][c]1:[cH]:[cH]:[cH]:[cH]:[cH]:1	-0.378
FCFP_6	16	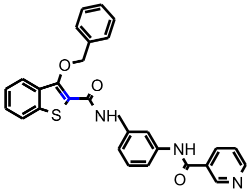 [*][c](:[*]):[*]	-0.354

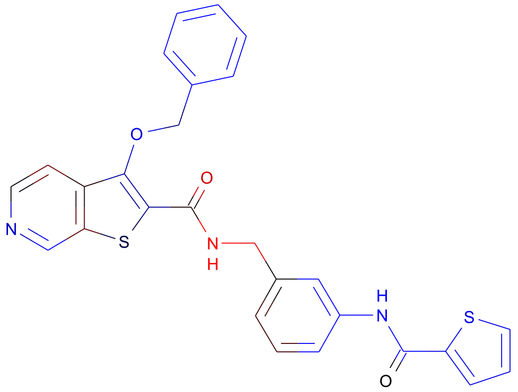

$C_{27}H_{21}N_3O_3S_2$
Molecular Weight: 499.60394
ALogP: 4.929
Rotatable Bonds: 8
Acceptors: 4
Donors: 2

Model Prediction

Prediction: 84.954
Unit: mg/kg_body_weight/day
Mahalanobis Distance: 21.005
Mahalanobis Distance p-value: 3.18e-033

Mahalanobis Distance: The Mahalanobis distance (MD) is a generalization of the Euclidean distance that accounts for correlations among the X properties. It is calculated as the distance to the center of the training data. The larger the MD, the less trustworthy the prediction.
Mahalanobis Distance p-value: The p-value gives the fraction of training data with an MD greater than or equal to the one for the given sample, assuming normally distributed data. The smaller the p-value, the less trustworthy the prediciton. For highly non-normal X properties (e.g., fingerprints), the MD p-value is wildly inaccurate.

Structural Similar Compounds

Name	Fluvastatin	913	C.I. direct brown 95
Structure	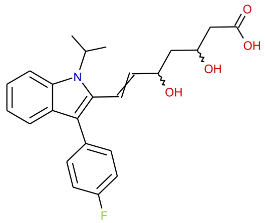	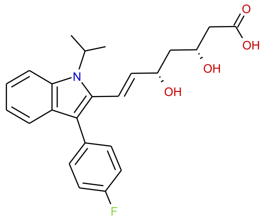	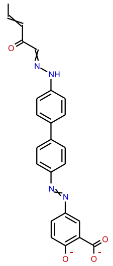
Actual Endpoint (-log C)	3.51742	3.51742	5.31387
Predicted Endpoint (-log C)	5.41573	5.41573	4.30266
Distance	0.858	0.858	0.890
Reference	CPDB	CPDB	CPDB

Model Applicability

Unknown features are fingerprint features in the query molecule, but not found in the training set.

1. OPS PC6 out of range. Value: 6.8529. Training min, max, SD, explained variance: -5.5832, 6.4847, 1.973, 0.0374.

Feature Contribution

Top features for positive contribution			
**Fingerprint**	**Bit/Smiles**	**Feature Structure**	**Score**
FCFP_6	1	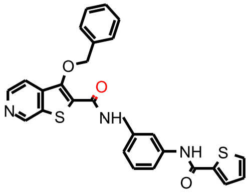 [*]=O	0.234

FCFP_6	-885550502	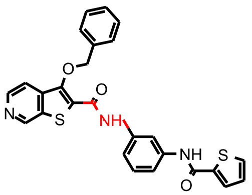<br>[*]CNC(=[*])[*]	0.229
FCFP_6	730557100	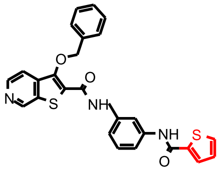<br>[*][c]1:[*]:n:[cH]:[c<br>H]:[cH]:1	0.141
**Top Features for negative contribution**			
**Fingerprint**	**Bit/Smiles**	**Feature Structure**	**Score**
FCFP_6	991735244	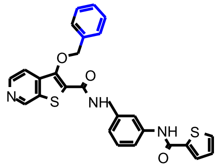<br>[*][c]1:[*]:[cH]:[cH]<br>:[cH]:[cH]:1	-0.422
FCFP_6	-2093839777	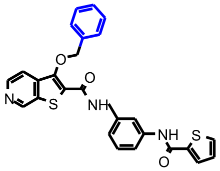<br>[*][c]1:[cH]:[cH]:[cH<br>]:[cH]:[cH]:1	-0.378
FCFP_6	-1280036918	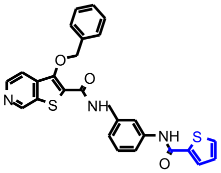<br>[*]C(=[*])[c]1:[cH]:[<br>cH]:[cH]:s:1	-0.363

Co-crystallized ligand

TOPKAT_Carcinogenic_Potency_TD50_Rat

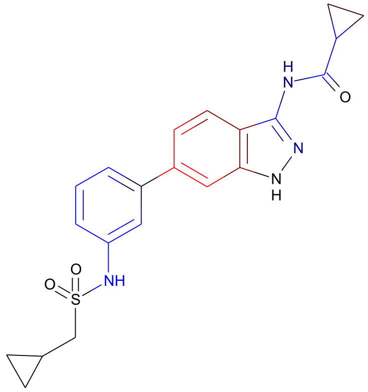

$C_{21}H_{22}N_{4}O_{3}S$

Molecular Weight: 410.48938

ALogP: 3.14

Rotatable Bonds: 7

Acceptors: 4

Donors: 3

Model Prediction

Prediction: 7.863

Unit: mg/kg_body_weight/day

Mahalanobis Distance: 15.505

Mahalanobis Distance p-value: 1.03e-012

Mahalanobis Distance: The Mahalanobis distance (MD) is a generalization of the Euclidean distance that accounts for correlations among the X properties. It is calculated as the distance to the center of the training data. The larger the MD, the less trustworthy the prediction.
Mahalanobis Distance p-value: The p-value gives the fraction of training data with an MD greater than or equal to the one for the given sample, assuming normally distributed data. The smaller the p-value, the less trustworthy the prediciton. For highly non-normal X properties (e.g., fingerprints), the MD p-value is wildly inaccurate.

Structural Similar Compounds

Name	Fluvastatin	913	Ochratoxin A
Structure	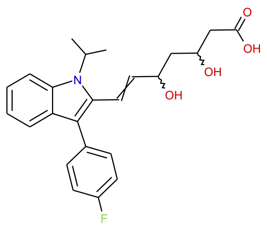	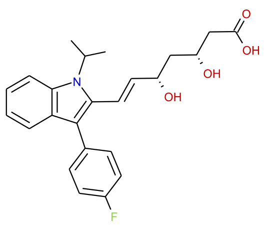	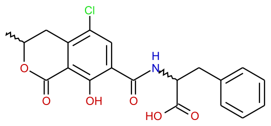
Actual Endpoint (-log C)	3.51742	3.51742	6.47264
Predicted Endpoint (-log C)	5.41573	5.41573	5.06501
Distance	0.685	0.685	0.743
Reference	CPDB	CPDB	CPDB

Model Applicability

Unknown features are fingerprint features in the query molecule, but not found in the training set.

1. OPS PC17 out of range. Value: 4.9376. Training min, max, SD, explained variance: -4.122, 4.7538, 1.355, 0.0176.

Feature Contribution

Top features for positive contribution

Fingerprint	Bit/Smiles	Feature Structure	Score
FCFP_6	-1861645784	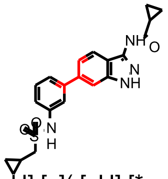 [*]:[cH]:[c](:[cH]:[*])[c](:[*]):[*]	0.359

FCFP_6	1	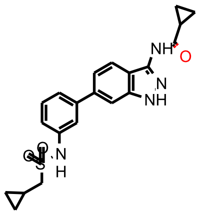 [*]=O	0.234
FCFP_6	307419094	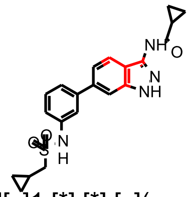 [*][c]1:[*]:[*]:[c](:[*]):[c]:1:[cH]:[*]	0.121
**Top Features for negative contribution**			
**Fingerprint**	**Bit/Smiles**	**Feature Structure**	**Score**
FCFP_6	991735244	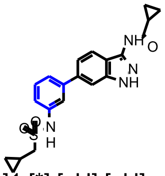 [*][c]1:[*]:[cH]:[cH]:[cH]:[cH]:1	-0.422
FCFP_6	16	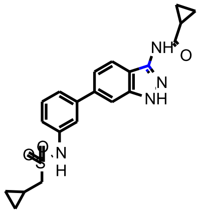 [*][c](:[*]):[*]	-0.354
FCFP_6	590925877	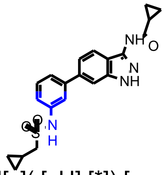 [*]N[c](:[cH]:[*]):[cH]:[*]	-0.323

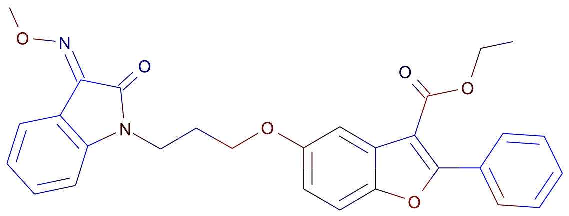

$C_{29}H_{26}N_2O_6$

Molecular Weight: 498.52653

ALogP: 4.994

Rotatable Bonds: 10

Acceptors: 6

Donors: 0

Model Prediction

Prediction: 0.041

Unit: g/kg_body_weight

Mahalanobis Distance: 34.193

Mahalanobis Distance p-value: 3.29e-032

Mahalanobis Distance: The Mahalanobis distance (MD) is a generalization of the Euclidean distance that accounts for correlations among the X properties. It is calculated as the distance to the center of the training data. The larger the MD, the less trustworthy the prediction.
Mahalanobis Distance p-value: The p-value gives the fraction of training data with an MD greater than or equal to the one for the given sample, assuming normally distributed data. The smaller the p-value, the less trustworthy the prediciton. For highly non-normal X properties (e.g., fingerprints), the MD p-value is wildly inaccurate.

Structural Similar Compounds

Name	FLUVALINATE	ASSURE	RESERPINE
Structure	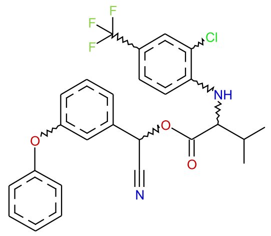	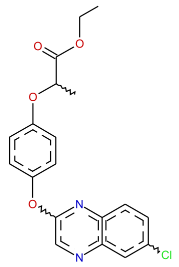	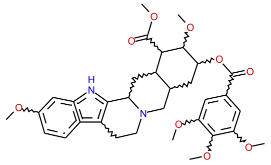
Actual Endpoint (-log C)	5.30356	5.00328	6.38645
Predicted Endpoint (-log C)	4.89944	4.27671	5.548
Distance	0.706	0.751	0.788
Reference	EPA COVER SHEET 0281;880630;(1)	EPA COVER SHEET 0335;891001;(1)	NTP 193 22

Model Applicability

Unknown features are fingerprint features in the query molecule, but not found in the training set.

1. All properties and OPS components are within expected ranges.
2. Unknown ECFP_6 feature: 1203316083: [*][c]1:[*]:[*]:[c](:[*]):o:1
3. Unknown ECFP_6 feature: -813643813: [*][c]1:[*]:[*]:o:[c]:1[c](:[*]):[*]
4. Unknown ECFP_6 feature: -1658273810: [*]C(=[*])[c]1:[c]([*]):[*]:[*]:[c]:1:[*]
5. Unknown ECFP_6 feature: 1333660716: [*][c]1:[*]:[*]:[c](:[*]):[c]:1:[cH]:[*]
6. Unknown ECFP_6 feature: 1334014211: [*]:[cH]:[c]1:o:[*]:[*]:[c]:1:[*]
7. Unknown ECFP_6 feature: 1430791942: [*]OC(=O)[c](:[*]):[*]
8. Unknown ECFP_6 feature: -181568884: [*]:[cH]:[c](:[cH]:[*])[c](:[*]):[*]
9. Unknown ECFP_6 feature: 1997021792: [*]:[cH]:[cH]:[cH]:[*]
10. Unknown ECFP_6 feature: -1255706725: [*]CO[c](:[*]):[*]
11. Unknown ECFP_6 feature: -1790412586: [*]CCO[*]
12. Unknown ECFP_6 feature: -1788978402: [*]CCN([*])[*]
13. Unknown ECFP_6 feature: -661097313: [*]CN1C(=[*])[*][*]:[c]1:[*]
14. Unknown ECFP_6 feature: 1945129186: [*]N1[*]:[*]C(=[*])C1=O
15. Unknown ECFP_6 feature: 1716966732: [*]\N=C/1\C(=[*])[*][*]:[c]1:[*]
16. Unknown ECFP_6 feature: -1236953626: [*]N1[*][*][c](:[*]):[c]1:[cH]:[*]
17. Unknown ECFP_6 feature: -820505146: [*]ON=C([*])[*]
18. Unknown ECFP_6 feature: -408704017: [*]=NOC
19. Unknown ECFP_6 feature: -949601813: [*]OCC

Feature Contribution

Top features for positive contribution			
**Fingerprint**	**Bit/Smiles**	**Feature Structure**	**Score**
FCFP_6	-1143715940	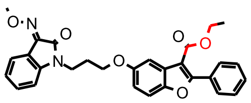<br>[*]COC(=[*])[*]	0.130
ECFP_6	1559650422	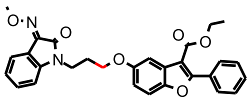<br>[*]C[*]	0.129
ECFP_6	-176455838	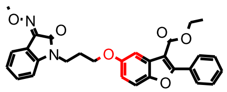<br>[*]O[c](:[cH]:[*]):[cH]:[*]	0.106
**Top Features for negative contribution**			
**Fingerprint**	**Bit/Smiles**	**Feature Structure**	**Score**
FCFP_6	991735244	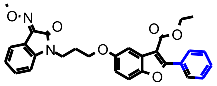<br>[*][c]1:[*]:[cH]:[cH]:[cH]:[cH]:1	-0.134

ECFP_6	1564392544	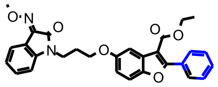 [*][c]1:[*]:[cH]:[cH]:[cH]:[cH]:1	-0.133
ECFP_6	2106656448	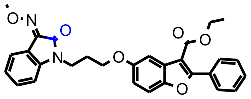 [*]C(=O)[*]	-0.110

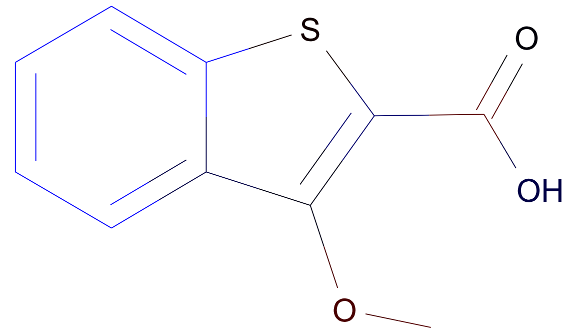

$C_{10}H_8O_3S$
Molecular Weight: 208.23372
ALogP: 2.596
Rotatable Bonds: 2
Acceptors: 3
Donors: 1

Model Prediction

Prediction: 0.088
Unit: g/kg_body_weight
Mahalanobis Distance: 28.898
Mahalanobis Distance p-value: 1.18e-022

Mahalanobis Distance: The Mahalanobis distance (MD) is a generalization of the Euclidean distance that accounts for correlations among the X properties. It is calculated as the distance to the center of the training data. The larger the MD, the less trustworthy the prediction.
Mahalanobis Distance p-value: The p-value gives the fraction of training data with an MD greater than or equal to the one for the given sample, assuming normally distributed data. The smaller the p-value, the less trustworthy the prediciton. For highly non-normal X properties (e.g., fingerprints), the MD p-value is wildly inaccurate.

Structural Similar Compounds

Name	NABUMETONE	KETOROLAC.TROMETHAMINE	8-METHOXYPSORALEN
Structure	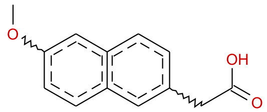	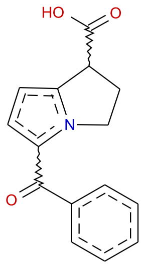	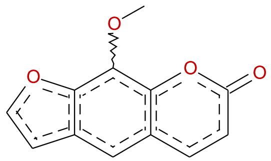
Actual Endpoint (-log C)	3.7609	5.10597	3.9067
Predicted Endpoint (-log C)	3.98782	4.00475	4.12187
Distance	0.488	0.530	0.541
Reference	NDA-19583	NDA-19698	NTP REPORT # 359

Model Applicability

Unknown features are fingerprint features in the query molecule, but not found in the training set.

1. All properties and OPS components are within expected ranges.
2. Unknown ECFP_6 feature: 914325265: [*]:s:[*]
3. Unknown ECFP_6 feature: -1670580914: [*]C(=[*])[c]1:s:[*]:[*]:[c]:1[*]
4. Unknown ECFP_6 feature: -1531301414: [*]O[c]1:[c]([*]):[*]:[*]:[c]:1:[*]
5. Unknown ECFP_6 feature: 1333660716: [*][c]1:[*]:[*]:[c](:[*]):[c]:1:[cH]:[*]
6. Unknown ECFP_6 feature: 1895035276: [*]:[cH]:[c]1:s:[*]:[*]:[c]:1:[*]
7. Unknown ECFP_6 feature: 85262808: [*][c]1:[*]:[*]:[c](:[*]):s:1
8. Unknown ECFP_6 feature: 1997021792: [*]:[cH]:[cH]:[cH]:[*]
9. Unknown ECFP_6 feature: 1429461619: [*]:[c](:[*])C(=O)O
10. Unknown ECFP_6 feature: 1307307440: [*]:[c](:[*])OC

Feature Contribution

Top features for positive contribution

Fingerprint	Bit/Smiles	Feature Structure	Score

ECFP_6	2099970318	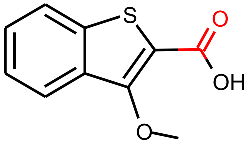<br>[*]C(=O)[*]	0.077
FCFP_6	1036089772	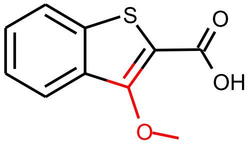<br>[*]CO[c](:[*]):[*]	0.073
FCFP_6	136627117	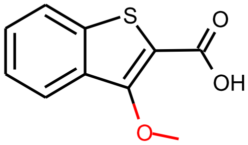<br>[*]OC	0.054

Top Features for negative contribution

Fingerprint	Bit/Smiles	Feature Structure	Score
FCFP_6	991735244	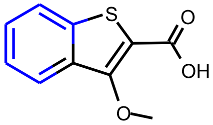<br>[*][c]1:[*]:[cH]:[cH]<br>:[cH]:[cH]:1	-0.134
ECFP_6	1564392544	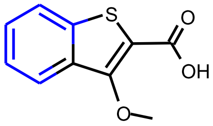<br>[*][c]1:[*]:[cH]:[cH]<br>:[cH]:[cH]:1	-0.133

FCFP_6	1	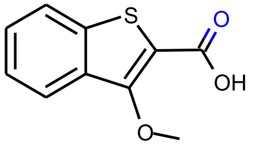<br><br>[*]=O	-0.102

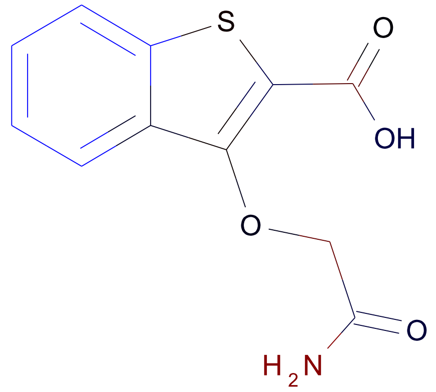

$C_{11}H_9NO_4S$
Molecular Weight: 251.25846
ALogP: 1.45
Rotatable Bonds: 4
Acceptors: 4
Donors: 2

Model Prediction

Prediction: 0.076
Unit: g/kg_body_weight
Mahalanobis Distance: 33.247
Mahalanobis Distance p-value: 1.57e-030

Mahalanobis Distance: The Mahalanobis distance (MD) is a generalization of the Euclidean distance that accounts for correlations among the X properties. It is calculated as the distance to the center of the training data. The larger the MD, the less trustworthy the prediction.
Mahalanobis Distance p-value: The p-value gives the fraction of training data with an MD greater than or equal to the one for the given sample, assuming normally distributed data. The smaller the p-value, the less trustworthy the prediciton. For highly non-normal X properties (e.g., fingerprints), the MD p-value is wildly inaccurate.

Structural Similar Compounds

Name	DAPSONE	FUROSEMIDE	DANTROLENE.NA
Structure	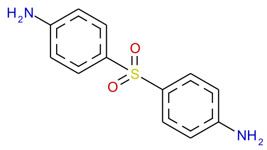	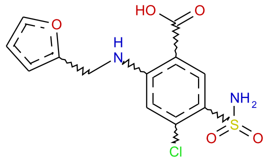	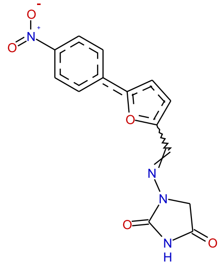
Actual Endpoint (-log C)	3.6168	4.27645	4.19625
Predicted Endpoint (-log C)	3.43657	4.40005	4.62637
Distance	0.562	0.591	0.620
Reference	NTP 20 47	NTP REPORT # 356	NDA-17443

Model Applicability

Unknown features are fingerprint features in the query molecule, but not found in the training set.

1. OPS PC30 out of range. Value: -4.1254. Training min, max, SD, explained variance: -3.8594, 4.2863, 1.27, 0.0071.
2. Unknown ECFP_6 feature: 914325265: [*]:s:[*]
3. Unknown ECFP_6 feature: -1670580914: [*]C(=[*])[c]1:s:[*]:[*]:[c]:1[*]
4. Unknown ECFP_6 feature: -1531301414: [*]O[c]1:[c]([*]):[*]:[*]:[c]:1:[*]
5. Unknown ECFP_6 feature: 1333660716: [*][c]1:[*]:[*]:[c](:[*]):[c]:1:[cH]:[*]
6. Unknown ECFP_6 feature: 1895035276: [*]:[cH]:[c]1:s:[*]:[*]:[c]:1:[*]
7. Unknown ECFP_6 feature: 85262808: [*][c]1:[*]:[*]:[c](:[*]):s:1
8. Unknown ECFP_6 feature: 1997021792: [*]:[cH]:[cH]:[cH]:[*]
9. Unknown ECFP_6 feature: 1429461619: [*]:[c](:[*])C(=O)O
10. Unknown ECFP_6 feature: -1255706725: [*]CO[c](:[*]):[*]
11. Unknown ECFP_6 feature: -1686813061: [*]OCC(=[*])[*]
12. Unknown ECFP_6 feature: -1708545601: [*]CC(=O)N
13. Unknown ECFP_6 feature: -932108170: [*]C(=[*])N

Feature Contribution

Top features for positive contribution			
Fingerprint	Bit/Smiles	Feature Structure	Score

ECFP_6	1559650422	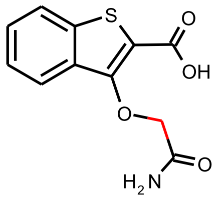 [*]C[*]	0.129
FCFP_6	3	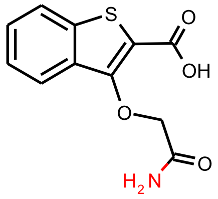 [*]N	0.092
ECFP_6	2099970318	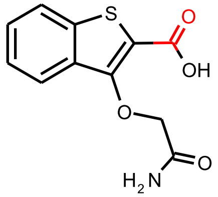 [*]C(=O)[*]	0.077
**Top Features for negative contribution**			
**Fingerprint**	**Bit/Smiles**	**Feature Structure**	**Score**
FCFP_6	991735244	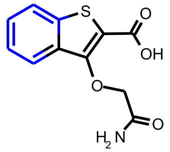 [*][c]1:[*]:[cH]:[cH]:[cH]:[cH]:1	-0.134
ECFP_6	1564392544	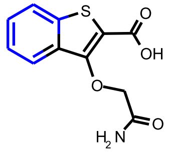 [*][c]1:[*]:[cH]:[cH]:[cH]:[cH]:1	-0.133

FCFP_6	1	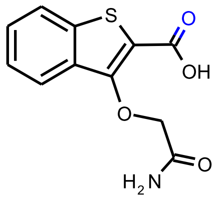 [*]=O	-0.102

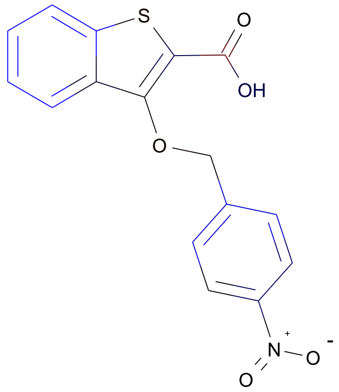

$C_{16}H_{11}NO_5S$
Molecular Weight: 329.32724
ALogP: 4.074
Rotatable Bonds: 5
Acceptors: 5
Donors: 1

Model Prediction

Prediction: 0.035
Unit: g/kg_body_weight
Mahalanobis Distance: 37.876
Mahalanobis Distance p-value: 1.33e-038

Mahalanobis Distance: The Mahalanobis distance (MD) is a generalization of the Euclidean distance that accounts for correlations among the X properties. It is calculated as the distance to the center of the training data. The larger the MD, the less trustworthy the prediction.
Mahalanobis Distance p-value: The p-value gives the fraction of training data with an MD greater than or equal to the one for the given sample, assuming normally distributed data. The smaller the p-value, the less trustworthy the prediciton. For highly non-normal X properties (e.g., fingerprints), the MD p-value is wildly inaccurate.

Structural Similar Compounds

Name	C.I. PIGMENT RED 3	SODIUM ACIFLUORFEN	D & C RED 9
Structure	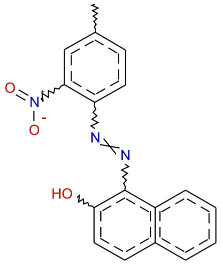	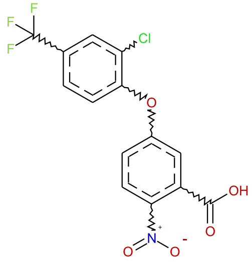	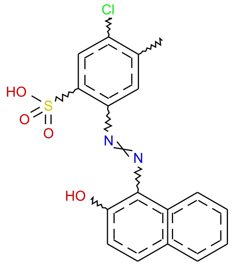
Actual Endpoint (-log C)	3.0252	4.16036	3.87715
Predicted Endpoint (-log C)	3.34768	4.65915	3.6546
Distance	0.573	0.582	0.622
Reference	NTP REPORT # 407	EPA COVER SHEET 0192;891101;(1)	NTP REPORT # 225

Model Applicability

Unknown features are fingerprint features in the query molecule, but not found in the training set.

1. All properties and OPS components are within expected ranges.
2. Unknown FCFP_2 feature: 5: [*][O-]
3. Unknown FCFP_2 feature: -828984032: [*][N+](=[*])[c](:[cH]:[*]):[cH]:[*]
4. Unknown FCFP_2 feature: -1338588315: [*]:[c](:[*])[N+](=O)[O-]
5. Unknown FCFP_2 feature: 1872392852: [*][N+](=O)[*]
6. Unknown FCFP_2 feature: 260476081: [*][N+](=[*])[O-]
7. Unknown ECFP_6 feature: 914325265: [*]:s:[*]
8. Unknown ECFP_6 feature: 1043790491: [*][N+](=[*])[*]
9. Unknown ECFP_6 feature: 781519895: [*][O-]
10. Unknown ECFP_6 feature: -1670580914: [*]C(=[*])[c]1:s:[*]:[*]:[c]:1[*]
11. Unknown ECFP_6 feature: -1531301414: [*]O[c]1:[c]([*]):[*]:[*]:[c]:1:[*]
12. Unknown ECFP_6 feature: 1333660716: [*][c]1:[*]:[*]:[c](:[*]):[c]:1:[cH]:[*]
13. Unknown ECFP_6 feature: 1895035276: [*]:[cH]:[c]1:s:[*]:[*]:[c]:1:[*]
14. Unknown ECFP_6 feature: 85262808: [*][c]1:[*]:[*]:[c](:[*]):s:1
15. Unknown ECFP_6 feature: 1997021792: [*]:[cH]:[cH]:[cH]:[*]
16. Unknown ECFP_6 feature: 1429461619: [*]:[c](:[*])C(=O)O
17. Unknown ECFP_6 feature: -1255706725: [*]CO[c](:[*]):[*]
18. Unknown ECFP_6 feature: 770547857: [*]OC[c](:[*]):[*]
19. Unknown ECFP_6 feature: -179073144: [*][N+](=[*])[c](:[cH]:[*]):[cH]:[*]
20. Unknown ECFP_6 feature: -215026467: [*]:[c](:[*])[N+](=O)[O-]

21. Unknown ECFP_6 feature: 2104376220: [*][N+](=O)[*]
22. Unknown ECFP_6 feature: -659271057: [*][N+](=[*])[O-]

Feature Contribution

Top features for positive contribution

Fingerprint	Bit/Smiles	Feature Structure	Score
ECFP_6	1559650422	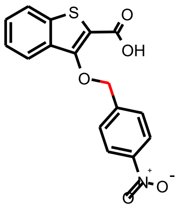 [*]C[*]	0.129
ECFP_6	2099970318	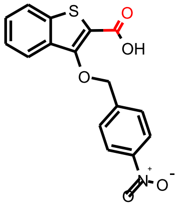 [*]C(=O)[*]	0.077
FCFP_6	1036089772	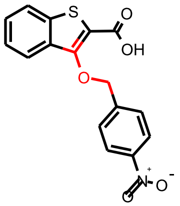 [*]CO[c](:[*]):[*]	0.073

Top Features for negative contribution

Fingerprint	Bit/Smiles	Feature Structure	Score
FCFP_6	991735244	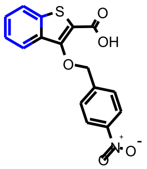 [*][c]1:[*]:[cH]:[cH]:[cH]:[cH]:1	-0.134

ECFP_6	1564392544	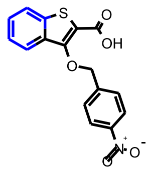<br>[*][c]1:[*]:[cH]:[cH]:[cH]:[cH]:1	-0.133
FCFP_6	1	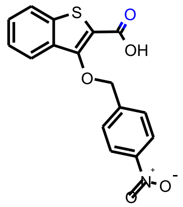<br>[*]=O	-0.102

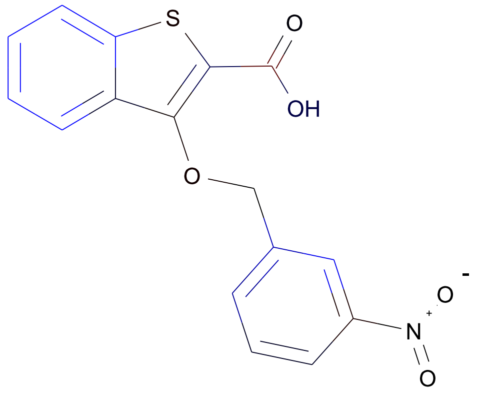

$C_{16}H_{11}NO_5S$
Molecular Weight: 329.32724
ALogP: 4.074
Rotatable Bonds: 5
Acceptors: 5
Donors: 1

Model Prediction

Prediction: 0.034
Unit: g/kg_body_weight
Mahalanobis Distance: 37.876
Mahalanobis Distance p-value: 1.33e-038

Mahalanobis Distance: The Mahalanobis distance (MD) is a generalization of the Euclidean distance that accounts for correlations among the X properties. It is calculated as the distance to the center of the training data. The larger the MD, the less trustworthy the prediction.
Mahalanobis Distance p-value: The p-value gives the fraction of training data with an MD greater than or equal to the one for the given sample, assuming normally distributed data. The smaller the p-value, the less trustworthy the prediciton. For highly non-normal X properties (e.g., fingerprints), the MD p-value is wildly inaccurate.

Structural Similar Compounds

Name	C.I. PIGMENT RED 3	SODIUM ACIFLUORFEN	D & C RED 9
Structure	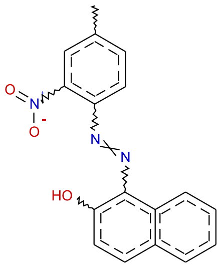	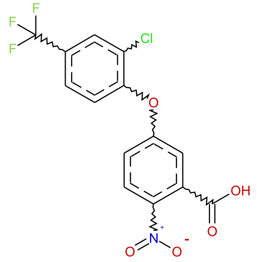	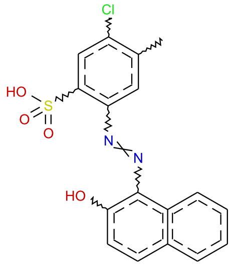
Actual Endpoint (-log C)	3.0252	4.16036	3.87715
Predicted Endpoint (-log C)	3.34768	4.65915	3.6546
Distance	0.573	0.581	0.622
Reference	NTP REPORT # 407	EPA COVER SHEET 0192;891101;(1)	NTP REPORT # 225

Model Applicability

Unknown features are fingerprint features in the query molecule, but not found in the training set.

1. All properties and OPS components are within expected ranges.
2. Unknown FCFP_2 feature: 5: [*][O-]
3. Unknown FCFP_2 feature: -828984032: [*][N+](=[*])[c](:[cH]:[*]):[cH]:[*]
4. Unknown FCFP_2 feature: -1338588315: [*]:[c](:[*])[N+](=O)[O-]
5. Unknown FCFP_2 feature: 1872392852: [*][N+](=O)[*]
6. Unknown FCFP_2 feature: 260476081: [*][N+](=[*])[O-]
7. Unknown ECFP_6 feature: 914325265: [*]:s:[*]
8. Unknown ECFP_6 feature: 1043790491: [*][N+](=[*])[*]
9. Unknown ECFP_6 feature: 781519895: [*][O-]
10. Unknown ECFP_6 feature: -1670580914: [*]C(=[*])[c]1:s:[*]:[*]:[c]:1[*]
11. Unknown ECFP_6 feature: -1531301414: [*]O[c]1:[c]([*]):[*]:[*]:[c]:1:[*]
12. Unknown ECFP_6 feature: 1333660716: [*][c]1:[*]:[*]:[c](:[*]):[c]:1:[cH]:[*]
13. Unknown ECFP_6 feature: 1895035276: [*]:[cH]:[c]1:s:[*]:[*]:[c]:1:[*]
14. Unknown ECFP_6 feature: 85262808: [*][c]1:[*]:[*]:[c](:[*]):s:1
15. Unknown ECFP_6 feature: 1997021792: [*]:[cH]:[cH]:[cH]:[*]
16. Unknown ECFP_6 feature: 1429461619: [*]:[c](:[*])C(=O)O
17. Unknown ECFP_6 feature: -1255706725: [*]CO[c](:[*]):[*]
18. Unknown ECFP_6 feature: 770547857: [*]OC[c](:[*]):[*]
19. Unknown ECFP_6 feature: -179073144: [*][N+](=[*])[c](:[cH]:[*]):[cH]:[*]
20. Unknown ECFP_6 feature: -215026467: [*]:[c](:[*])[N+](=O)[O-]

21. Unknown ECFP_6 feature: 2104376220: [*][N+](=O)[*]
22. Unknown ECFP_6 feature: -659271057: [*][N+](=[*])[O-]

Feature Contribution

Top features for positive contribution			
**Fingerprint**	**Bit/Smiles**	**Feature Structure**	**Score**
ECFP_6	1559650422	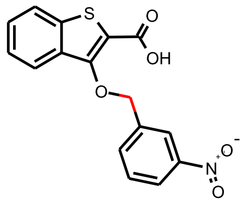 [*]C[*]	0.129
ECFP_6	2099970318	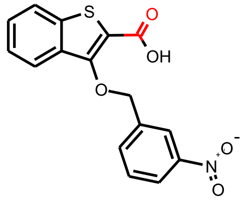 [*]C(=O)[*]	0.077
FCFP_6	1036089772	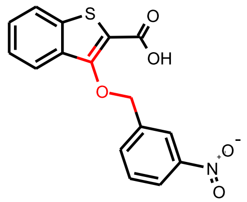 [*]CO[c](:[*]):[*]	0.073

Top Features for negative contribution			
**Fingerprint**	**Bit/Smiles**	**Feature Structure**	**Score**
FCFP_6	991735244	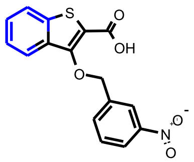 [*][c]1:[*]:[cH]:[cH]:[cH]:[cH]:1	-0.134

ECFP_6	1564392544	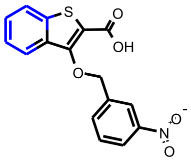 [*][c]1:[*]:[cH]:[cH]:[cH]:[cH]:1	-0.133
FCFP_6	1	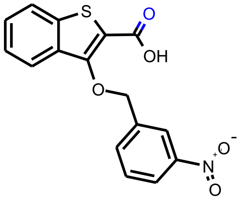 [*]=O	-0.102

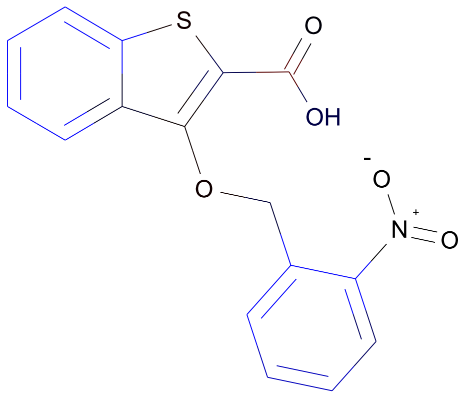

$C_{16}H_{11}NO_5S$
Molecular Weight: 329.32724
ALogP: 4.074
Rotatable Bonds: 5
Acceptors: 5
Donors: 1

Structural Similar Compounds

Name	C.I. PIGMENT RED 3	SODIUM ACIFLUORFEN	D & C RED 9
Structure	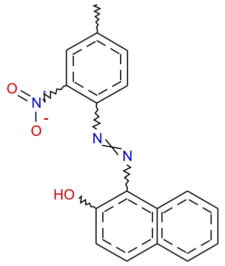	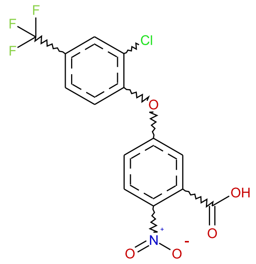	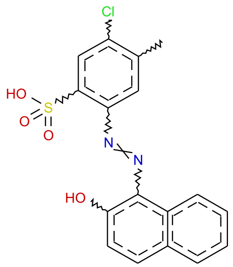
Actual Endpoint (-log C)	3.0252	4.16036	3.87715
Predicted Endpoint (-log C)	3.34768	4.65915	3.6546
Distance	0.571	0.580	0.621
Reference	NTP REPORT # 407	EPA COVER SHEET 0192;891101;(1)	NTP REPORT # 225

Model Prediction

Prediction: 0.033
Unit: g/kg_body_weight
Mahalanobis Distance: 36.280
Mahalanobis Distance p-value: 7.27e-036

Mahalanobis Distance: The Mahalanobis distance (MD) is a generalization of the Euclidean distance that accounts for correlations among the X properties. It is calculated as the distance to the center of the training data. The larger the MD, the less trustworthy the prediction.
Mahalanobis Distance p-value: The p-value gives the fraction of training data with an MD greater than or equal to the one for the given sample, assuming normally distributed data. The smaller the p-value, the less trustworthy the prediciton. For highly non-normal X properties (e.g., fingerprints), the MD p-value is wildly inaccurate.

Model Applicability

Unknown features are fingerprint features in the query molecule, but not found in the training set.

1. All properties and OPS components are within expected ranges.
2. Unknown FCFP_2 feature: 5: [*][O-]
3. Unknown FCFP_2 feature: -828984032: [*][N+](=[*])[c](:[cH]:[*]):[cH]:[*]
4. Unknown FCFP_2 feature: -1338588315: [*]:[c](:[*])[N+](=O)[O-]
5. Unknown FCFP_2 feature: 1872392852: [*][N+](=O)[*]
6. Unknown FCFP_2 feature: 260476081: [*][N+](=[*])[O-]
7. Unknown ECFP_6 feature: 914325265: [*]:s:[*]
8. Unknown ECFP_6 feature: 1043790491: [*][N+](=[*])[*]
9. Unknown ECFP_6 feature: 781519895: [*][O-]
10. Unknown ECFP_6 feature: -1670580914: [*]C(=[*])[c]1:s:[*]:[*]:[c]:1[*]
11. Unknown ECFP_6 feature: -1531301414: [*]O[c]1:[c]([*]):[*]:[*]:[c]:1:[*]
12. Unknown ECFP_6 feature: 1333660716: [*][c]1:[*]:[*]:[c](:[*]):[c]:1:[cH]:[*]
13. Unknown ECFP_6 feature: 1895035276: [*]:[cH]:[c]1:s:[*]:[*]:[c]:1:[*]
14. Unknown ECFP_6 feature: 85262808: [*][c]1:[*]:[*]:[c](:[*]):s:1
15. Unknown ECFP_6 feature: 1997021792: [*]:[cH]:[cH]:[cH]:[*]
16. Unknown ECFP_6 feature: 1429461619: [*]:[c](:[*])C(=O)O
17. Unknown ECFP_6 feature: -1255706725: [*]CO[c](:[*]):[*]
18. Unknown ECFP_6 feature: 770547857: [*]OC[c](:[*]):[*]
19. Unknown ECFP_6 feature: -2024509555: [*]C[c](:[cH]:[*]):[c]([*]):[*]
20. Unknown ECFP_6 feature: -1956535100: [*][c](:[*]):[c](:[cH]:[*])[N+](=[*])[*]

21. Unknown ECFP_6 feature: -215026467: [*]:[c](:[*])[N+](=O)[O-]
22. Unknown ECFP_6 feature: 2104376220: [*][N+](=O)[*]
23. Unknown ECFP_6 feature: -659271057: [*][N+](=[*])[O-]

Feature Contribution

Top features for positive contribution			
**Fingerprint**	**Bit/Smiles**	**Feature Structure**	**Score**
ECFP_6	1559650422	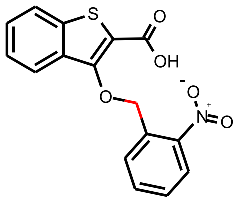 [*]C[*]	0.129
ECFP_6	2099970318	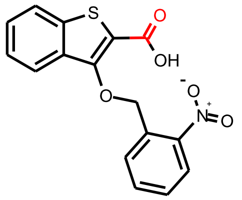 [*]C(=O)[*]	0.077
FCFP_6	1036089772	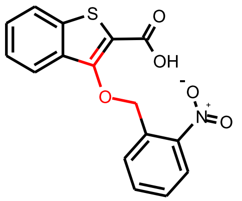 [*]CO[c](:[*]):[*]	0.073

Top Features for negative contribution			
**Fingerprint**	**Bit/Smiles**	**Feature Structure**	**Score**

FCFP_6	991735244	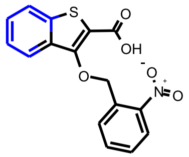<br>[*][c]1:[*]:[cH]:[cH]:[cH]:[cH]:1	-0.134
ECFP_6	1564392544	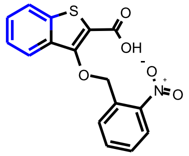<br>[*][c]1:[*]:[cH]:[cH]:[cH]:[cH]:1	-0.133
FCFP_6	1	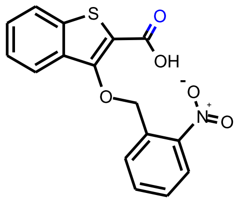<br>[*]=O	-0.102

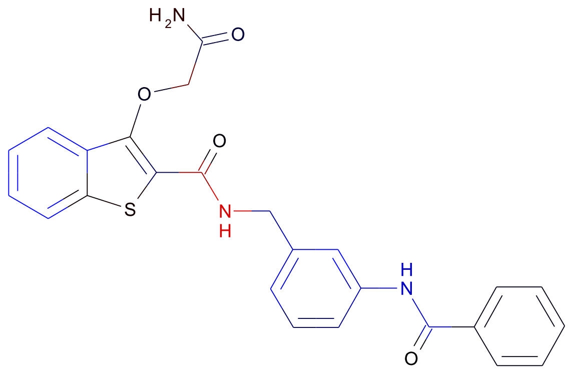

$C_{25}H_{21}N_3O_4S$
Molecular Weight: 459.51694
ALogP: 3.396
Rotatable Bonds: 8
Acceptors: 4
Donors: 3

Model Prediction

Prediction: 0.059
Unit: g/kg_body_weight
Mahalanobis Distance: 40.235
Mahalanobis Distance p-value: 1.49e-042

Mahalanobis Distance: The Mahalanobis distance (MD) is a generalization of the Euclidean distance that accounts for correlations among the X properties. It is calculated as the distance to the center of the training data. The larger the MD, the less trustworthy the prediction.
Mahalanobis Distance p-value: The p-value gives the fraction of training data with an MD greater than or equal to the one for the given sample, assuming normally distributed data. The smaller the p-value, the less trustworthy the prediciton. For highly non-normal X properties (e.g., fingerprints), the MD p-value is wildly inaccurate.

Structural Similar Compounds

Name	GLYBURIDE	C.I.PIGMENT RED 23	GLIPIZIDE
Structure	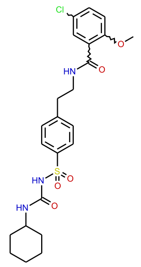	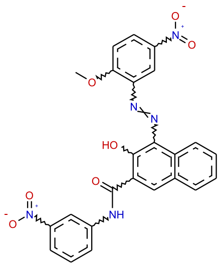	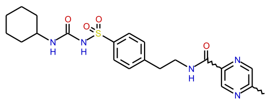
Actual Endpoint (-log C)	4.21661	2.28997	3.94991
Predicted Endpoint (-log C)	4.21035	3.52921	3.95594
Distance	0.760	0.785	0.790
Reference	UPJ-26452	NTP 411 146	NDA-17583

Model Applicability

Unknown features are fingerprint features in the query molecule, but not found in the training set.

1. OPS PC22 out of range. Value: -4.7641. Training min, max, SD, explained variance: -4.3287, 5.3383, 1.588, 0.0110.
2. Unknown ECFP_6 feature: 914325265: [*]:s:[*]
3. Unknown ECFP_6 feature: -1670580914: [*]C(=[*])[c]1:s:[*]:[*]:[c]:1[*]
4. Unknown ECFP_6 feature: -1531301414: [*]O[c]1:[c]([*]):[*]:[*]:[c]:1:[*]
5. Unknown ECFP_6 feature: 1333660716: [*][c]1:[*]:[*]:[c](:[*]):[c]:1:[cH]:[*]
6. Unknown ECFP_6 feature: 1895035276: [*]:[cH]:[c]1:s:[*]:[*]:[c]:1:[*]
7. Unknown ECFP_6 feature: 85262808: [*][c]1:[*]:[*]:[c](:[*]):s:1
8. Unknown ECFP_6 feature: 1997021792: [*]:[cH]:[cH]:[cH]:[*]
9. Unknown ECFP_6 feature: 1430169877: [*]NC(=O)[c](:[*]):[*]
10. Unknown ECFP_6 feature: 497523368: [*]CNC(=[*])[*]
11. Unknown ECFP_6 feature: -1255706725: [*]CO[c](:[*]):[*]
12. Unknown ECFP_6 feature: -1686813061: [*]OCC(=[*])[*]
13. Unknown ECFP_6 feature: -1708545601: [*]CC(=O)N
14. Unknown ECFP_6 feature: -932108170: [*]C(=[*])N
15. Unknown ECFP_6 feature: 769925792: [*]NC[c](:[*]):[*]
16. Unknown ECFP_6 feature: -177077903: [*]N[c](:[cH]:[*]):[cH]:[*]
17. Unknown ECFP_6 feature: -175146122: [*]C(=[*])[c](:[cH]:[*]):[cH]:[*]

Feature Contribution

Top features for positive contribution

Fingerprint	Bit/Smiles	Feature Structure	Score
ECFP_6	1559650422	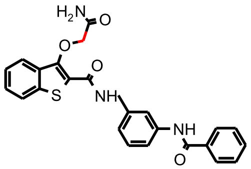 [*]C[*]	0.129
FCFP_6	3	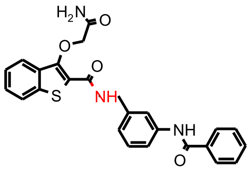 [*]N	0.092
FCFP_6	-2093839777	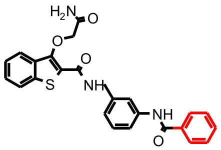 [*][c]1:[cH]:[cH]:[cH]:[cH]:[cH]:1	0.078

Top Features for negative contribution

Fingerprint	Bit/Smiles	Feature Structure	Score
FCFP_6	991735244	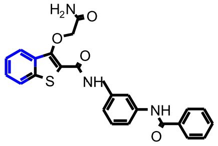 [*][c]1:[*]:[cH]:[cH]:[cH]:[cH]:1	-0.134

ECFP_6	1564392544	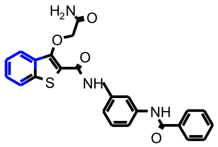 [*][c]1:[*]:[cH]:[cH]:[cH]:[cH]:1	-0.133
FCFP_6	1	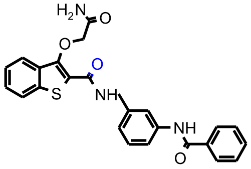 [*]=O	-0.102

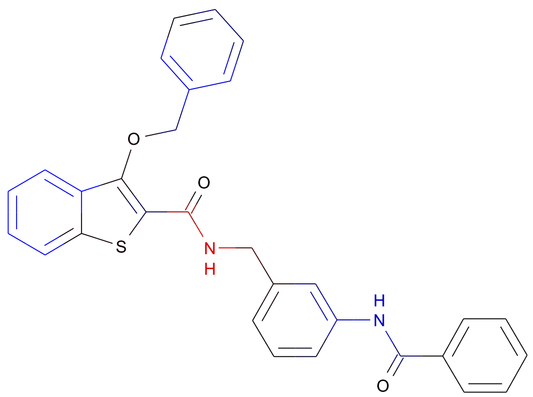

$C_{30}H_{24}N_{2}O_{3}S$
Molecular Weight: 492.58816
ALogP: 6.126
Rotatable Bonds: 8
Acceptors: 3
Donors: 2

Model Prediction

Prediction: 0.037
Unit: g/kg_body_weight
Mahalanobis Distance: 41.298
Mahalanobis Distance p-value: 2.7e-044

Mahalanobis Distance: The Mahalanobis distance (MD) is a generalization of the Euclidean distance that accounts for correlations among the X properties. It is calculated as the distance to the center of the training data. The larger the MD, the less trustworthy the prediction.
Mahalanobis Distance p-value: The p-value gives the fraction of training data with an MD greater than or equal to the one for the given sample, assuming normally distributed data. The smaller the p-value, the less trustworthy the prediciton. For highly non-normal X properties (e.g., fingerprints), the MD p-value is wildly inaccurate.

Structural Similar Compounds

Name	FLUVALINATE	DIARYLANILIDE YELLOW	C.I.PIGMENT RED 23
Structure	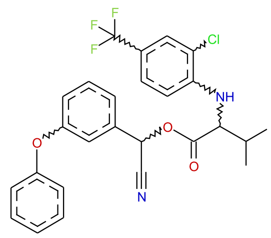	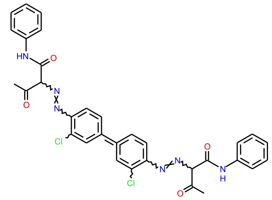	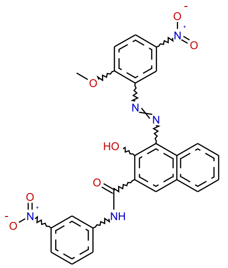
Actual Endpoint (-log C)	5.30356	2.70208	2.28997
Predicted Endpoint (-log C)	4.89944	3.76154	3.52921
Distance	0.824	0.897	0.931
Reference	EPA COVER SHEET 0281;880630;(1)	NTP 30 C-4	NTP 411 146

Model Applicability

Unknown features are fingerprint features in the query molecule, but not found in the training set.

1. OPS PC22 out of range. Value: -4.8636. Training min, max, SD, explained variance: -4.3287, 5.3383, 1.588, 0.0110.
2. Unknown ECFP_6 feature: 914325265: [*]:s:[*]
3. Unknown ECFP_6 feature: -1670580914: [*]C(=[*])[c]1:s:[*]:[*]:[c]:1[*]
4. Unknown ECFP_6 feature: -1531301414: [*]O[c]1:[c]([*]):[*]:[*]:[c]:1:[*]
5. Unknown ECFP_6 feature: 1333660716: [*][c]1:[*]:[*]:[c](:[*]):[c]:1:[cH]:[*]
6. Unknown ECFP_6 feature: 1895035276: [*]:[cH]:[c]1:s:[*]:[*]:[c]:1:[*]
7. Unknown ECFP_6 feature: 85262808: [*][c]1:[*]:[*]:[c](:[*]):s:1
8. Unknown ECFP_6 feature: 1997021792: [*]:[cH]:[cH]:[cH]:[*]
9. Unknown ECFP_6 feature: 1430169877: [*]NC(=O)[c](:[*]):[*]
10. Unknown ECFP_6 feature: 497523368: [*]CNC(=[*])[*]
11. Unknown ECFP_6 feature: -1255706725: [*]CO[c](:[*]):[*]
12. Unknown ECFP_6 feature: 770547857: [*]OC[c](:[*]):[*]
13. Unknown ECFP_6 feature: 769925792: [*]NC[c](:[*]):[*]
14. Unknown ECFP_6 feature: -177077903: [*]N[c](:[cH]:[*]):[cH]:[*]
15. Unknown ECFP_6 feature: -175146122: [*]C(=[*])[c](:[cH]:[*]):[cH]:[*]

Feature Contribution

Top features for positive contribution

Fingerprint	Bit/Smiles	Feature Structure	Score
ECFP_6	1559650422	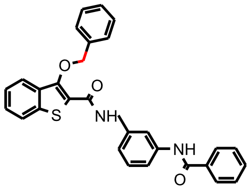 [*]C[*]	0.129
FCFP_6	3	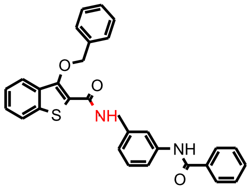 [*]N	0.092
FCFP_6	-2093839777	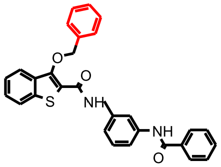 [*][c]1:[cH]:[cH]:[cH]:[cH]:[cH]:1	0.078
**Top Features for negative contribution**			
**Fingerprint**	**Bit/Smiles**	**Feature Structure**	**Score**
FCFP_6	991735244	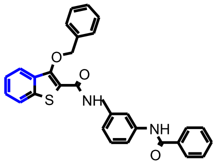 [*][c]1:[*]:[cH]:[cH] :[cH]:[cH]:1	-0.134

ECFP_6	1564392544	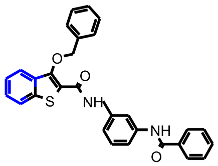<br>[*][c]1:[*]:[cH]:[cH]<br>:[cH]:[cH]:1	-0.133
FCFP_6	1	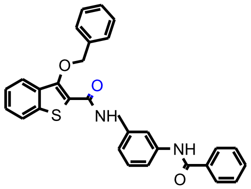<br>[*]=O	-0.102

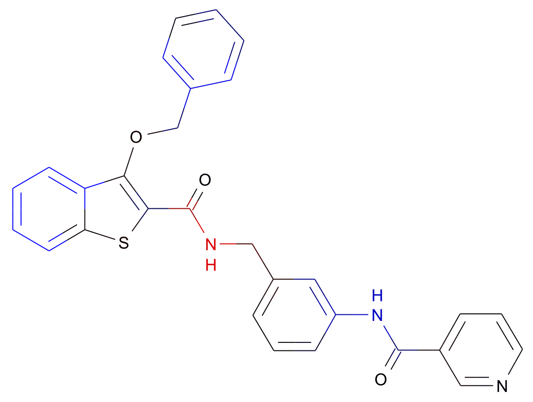

$C_{29}H_{23}N_3O_3S$
Molecular Weight: 493.57622
ALogP: 4.976
Rotatable Bonds: 8
Acceptors: 4
Donors: 2

Model Prediction

Prediction: 0.039
Unit: g/kg_body_weight
Mahalanobis Distance: 39.362
Mahalanobis Distance p-value: 4.18e-041

Mahalanobis Distance: The Mahalanobis distance (MD) is a generalization of the Euclidean distance that accounts for correlations among the X properties. It is calculated as the distance to the center of the training data. The larger the MD, the less trustworthy the prediction.
Mahalanobis Distance p-value: The p-value gives the fraction of training data with an MD greater than or equal to the one for the given sample, assuming normally distributed data. The smaller the p-value, the less trustworthy the prediciton. For highly non-normal X properties (e.g., fingerprints), the MD p-value is wildly inaccurate.

Structural Similar Compounds

Name	C.I.PIGMENT RED 23	FLUVALINATE	DIARYLANILIDE YELLOW
Structure	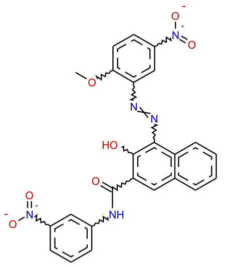	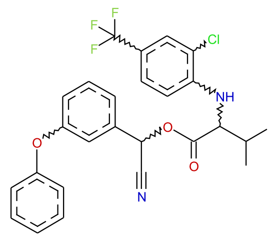	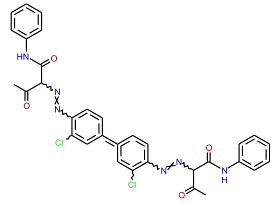
Actual Endpoint (-log C)	2.28997	5.30356	2.70208
Predicted Endpoint (-log C)	3.52921	4.89944	3.76154
Distance	0.852	0.865	0.870
Reference	NTP 411 146	EPA COVER SHEET 0281;880630;(1)	NTP 30 C-4

Model Applicability

Unknown features are fingerprint features in the query molecule, but not found in the training set.

1. OPS PC22 out of range. Value: -5.1794. Training min, max, SD, explained variance: -4.3287, 5.3383, 1.588, 0.0110.
2. Unknown ECFP_6 feature: 914325265: [*]:s:[*]
3. Unknown ECFP_6 feature: -1670580914: [*]C(=[*])[c]1:s:[*]:[*]:[c]:1[*]
4. Unknown ECFP_6 feature: -1531301414: [*]O[c]1:[c]([*]):[*]:[*]:[c]:1:[*]
5. Unknown ECFP_6 feature: 1333660716: [*][c]1:[*]:[*]:[c](:[*]):[c]:1:[cH]:[*]
6. Unknown ECFP_6 feature: 1895035276: [*]:[cH]:[c]1:s:[*]:[*]:[c]:1:[*]
7. Unknown ECFP_6 feature: 85262808: [*][c]1:[*]:[*]:[c](:[*]):s:1
8. Unknown ECFP_6 feature: 1997021792: [*]:[cH]:[cH]:[cH]:[*]
9. Unknown ECFP_6 feature: 1430169877: [*]NC(=O)[c](:[*]):[*]
10. Unknown ECFP_6 feature: 497523368: [*]CNC(=[*])[*]
11. Unknown ECFP_6 feature: -1255706725: [*]CO[c](:[*]):[*]
12. Unknown ECFP_6 feature: 770547857: [*]OC[c](:[*]):[*]
13. Unknown ECFP_6 feature: 769925792: [*]NC[c](:[*]):[*]
14. Unknown ECFP_6 feature: -177077903: [*]N[c](:[cH]:[*]):[cH]:[*]
15. Unknown ECFP_6 feature: -709633021: [*][c](:[*]):[cH]:n:[*]
16. Unknown ECFP_6 feature: -175146122: [*]C(=[*])[c](:[cH]:[*]):[cH]:[*]
17. Unknown ECFP_6 feature: 1996163143: [*]:[cH]:[cH]:n:[*]
18. Unknown ECFP_6 feature: -677055651: [*]:[cH]:n:[cH]:[*]

Feature Contribution

Top features for positive contribution			
**Fingerprint**	**Bit/Smiles**	**Feature Structure**	**Score**
ECFP_6	1559650422	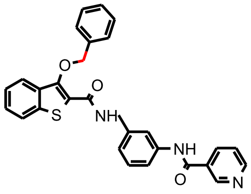 [*]C[*]	0.129
FCFP_6	3	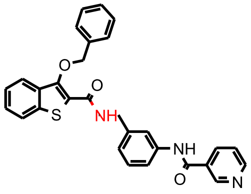 [*]N	0.092
FCFP_6	-2093839777	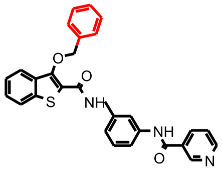 [*][c]1:[cH]:[cH]:[cH]:[cH]:[cH]:1	0.078

Top Features for negative contribution			
**Fingerprint**	**Bit/Smiles**	**Feature Structure**	**Score**
FCFP_6	991735244	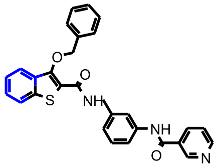 [*][c]1:[*]:[cH]:[cH] :[cH]:[cH]:1	-0.134

ECFP_6	1564392544	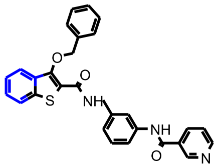 [*][c]1:[*]:[cH]:[cH]:[cH]:[cH]:1	-0.133
FCFP_6	1	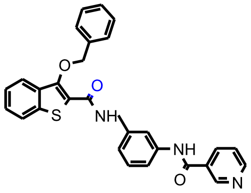 [*]=O	-0.102

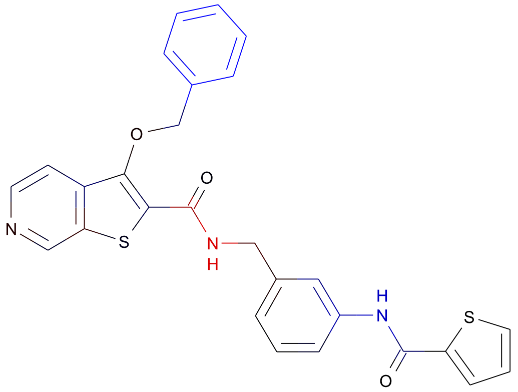

$C_{27}H_{21}N_3O_3S_2$
Molecular Weight: 499.60394
ALogP: 4.929
Rotatable Bonds: 8
Acceptors: 4
Donors: 2

Model Prediction

Prediction: 0.053
Unit: g/kg_body_weight
Mahalanobis Distance: 38.434
Mahalanobis Distance p-value: 1.51e-039

Mahalanobis Distance: The Mahalanobis distance (MD) is a generalization of the Euclidean distance that accounts for correlations among the X properties. It is calculated as the distance to the center of the training data. The larger the MD, the less trustworthy the prediction.
Mahalanobis Distance p-value: The p-value gives the fraction of training data with an MD greater than or equal to the one for the given sample, assuming normally distributed data. The smaller the p-value, the less trustworthy the prediciton. For highly non-normal X properties (e.g., fingerprints), the MD p-value is wildly inaccurate.

Structural Similar Compounds

Name	C.I.PIGMENT RED 23	DIARYLANILIDE YELLOW	C.I. ACID RED 14
Structure	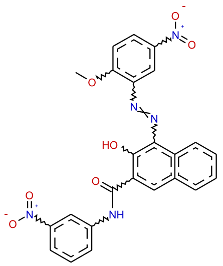	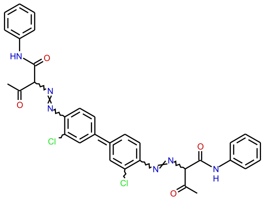	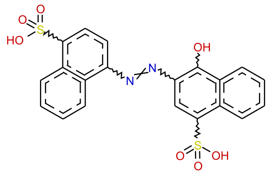
Actual Endpoint (-log C)	2.28997	2.70208	2.8654
Predicted Endpoint (-log C)	3.52921	3.76154	3.29295
Distance	0.793	0.865	0.907
Reference	NTP 411 146	NTP 30 C-4	NTP REPORT # 220

Model Applicability

Unknown features are fingerprint features in the query molecule, but not found in the training set.

1. OPS PC22 out of range. Value: -4.8994. Training min, max, SD, explained variance: -4.3287, 5.3383, 1.588, 0.0110.
2. Unknown ECFP_6 feature: 914325265: [*]:s:[*]
3. Unknown ECFP_6 feature: -709633021: [*][c](:[*]):[cH]:n:[*]
4. Unknown ECFP_6 feature: 1895035276: [*]:[cH]:[c]1:s:[*]:[*]:[c]:1:[*]
5. Unknown ECFP_6 feature: 1333660716: [*][c]1:[*]:[*]:[c](:[*]):[c]:1:[cH]:[*]
6. Unknown ECFP_6 feature: 1996163143: [*]:[cH]:[cH]:n:[*]
7. Unknown ECFP_6 feature: -677055651: [*]:[cH]:n:[cH]:[*]
8. Unknown ECFP_6 feature: -1531301414: [*]O[c]1:[c]([*]):[*]:[*]:[c]:1:[*]
9. Unknown ECFP_6 feature: -1670580914: [*]C(=[*])[c]1:s:[*]:[*]:[c]:1[*]
10. Unknown ECFP_6 feature: 85262808: [*][c]1:[*]:[*]:[c](:[*]):s:1
11. Unknown ECFP_6 feature: 1430169877: [*]NC(=O)[c](:[*]):[*]
12. Unknown ECFP_6 feature: 497523368: [*]CNC(=[*])[*]
13. Unknown ECFP_6 feature: -1255706725: [*]CO[c](:[*]):[*]
14. Unknown ECFP_6 feature: 770547857: [*]OC[c](:[*]):[*]
15. Unknown ECFP_6 feature: 1997021792: [*]:[cH]:[cH]:[cH]:[*]
16. Unknown ECFP_6 feature: 769925792: [*]NC[c](:[*]):[*]
17. Unknown ECFP_6 feature: -177077903: [*]N[c](:[cH]:[*]):[cH]:[*]
18. Unknown ECFP_6 feature: 1898414610: [*]C(=[*])[c]1:[cH]:[*]:[*]:s:1
19. Unknown ECFP_6 feature: 1996740348: [*]1:[*]:s:[cH]:[cH]:1
20. Unknown ECFP_6 feature: -1426923364: [*][c]1:[*]:[*]:[cH]:s:1

Feature Contribution

Top features for positive contribution

Fingerprint	Bit/Smiles	Feature Structure	Score
ECFP_6	1559650422	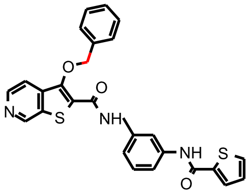 [*]C[*]	0.129
FCFP_6	3	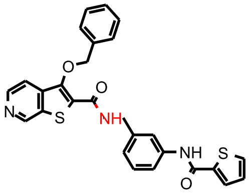 [*]N	0.092
FCFP_6	-2093839777	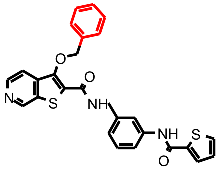 [*][c]1:[cH]:[cH]:[cH]:[cH]:[cH]:1	0.078

Top Features for negative contribution

Fingerprint	Bit/Smiles	Feature Structure	Score
FCFP_6	991735244	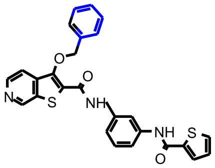 [*][c]1:[*]:[cH]:[cH]:[cH]:[cH]:1	-0.134

ECFP_6	1564392544	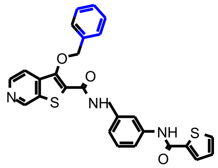 [*][c]1:[*]:[cH]:[cH]:[cH]:[cH]:1	-0.133
FCFP_6	1	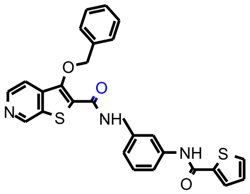 [*]=O	-0.102

Co-crystallized ligand

TOPKAT_Chronic_LOAEL

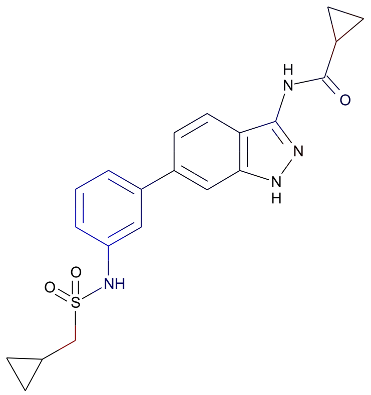

$C_{21}H_{22}N_4O_3S$

Molecular Weight: 410.48938

ALogP: 3.14

Rotatable Bonds: 7

Acceptors: 4

Donors: 3

Structural Similar Compounds

Name	GLIPIZIDE	GLYBURIDE	FUROSEMIDE
Structure	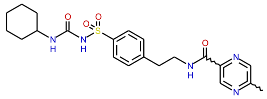	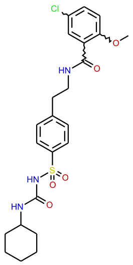	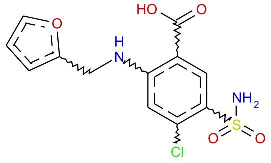
Actual Endpoint (-log C)	3.94991	4.21661	4.27645
Predicted Endpoint (-log C)	3.95594	4.21035	4.40005
Distance	0.627	0.629	0.725
Reference	NDA-17583	UPJ-26452	NTP REPORT # 356

Model Prediction

Prediction: 0.049

Unit: g/kg_body_weight

Mahalanobis Distance: 41.460

Mahalanobis Distance p-value: 1.48e-044

Mahalanobis Distance: The Mahalanobis distance (MD) is a generalization of the Euclidean distance that accounts for correlations among the X properties. It is calculated as the distance to the center of the training data. The larger the MD, the less trustworthy the prediction.

Mahalanobis Distance p-value: The p-value gives the fraction of training data with an MD greater than or equal to the one for the given sample, assuming normally distributed data. The smaller the p-value, the less trustworthy the prediciton. For highly non-normal X properties (e.g., fingerprints), the MD p-value is wildly inaccurate.

Model Applicability

Unknown features are fingerprint features in the query molecule, but not found in the training set.

1. All properties and OPS components are within expected ranges.
2. Unknown FCFP_2 feature: 262592487: [*]:[c]1:[*]:[*]:n:[nH]:1
3. Unknown FCFP_2 feature: 1018942292: [*]CS(=O)(=O)N[*]
4. Unknown ECFP_6 feature: -152683720: [*]:[nH]:[*]
5. Unknown ECFP_6 feature: -797085356: [*]S(=[*])(=[*])[*]
6. Unknown ECFP_6 feature: -857146788: [*]C(=[*])C1CC1
7. Unknown ECFP_6 feature: -81134287: [*]NC(=O)C1[*][*]1
8. Unknown ECFP_6 feature: 634582385: [*]:[c]1:[*]:[*]:n:[nH]:1
9. Unknown ECFP_6 feature: 600440273: [*][c]1:[*]:[*]:[nH]:n:1
10. Unknown ECFP_6 feature: 1049768340: [*]N[c]1:n:[*]:[*]:[c]:1:[*]
11. Unknown ECFP_6 feature: 1333660716: [*][c]1:[*]:[*]:[c](:[*]):[c]:1:[cH]:[*]
12. Unknown ECFP_6 feature: -181568884: [*]:[cH]:[c](:[cH]:[*])[c](:[*]):[*]
13. Unknown ECFP_6 feature: 1099224616: [*]:[cH]:[c]1:[nH]:[*]:[*]:[c]:1:[*]
14. Unknown ECFP_6 feature: -177077903: [*]N[c](:[cH]:[*]):[cH]:[*]
15. Unknown ECFP_6 feature: 1997021792: [*]:[cH]:[cH]:[cH]:[*]
16. Unknown ECFP_6 feature: -1238602038: [*]S(=[*])(=[*])N[c](:[*]):[*]
17. Unknown ECFP_6 feature: 268744321: [*]CS(=O)(=O)N[*]
18. Unknown ECFP_6 feature: 2102150379: [*]S(=[*])(=O)[*]
19. Unknown ECFP_6 feature: -1341194584: [*]S(=[*])(=[*])CC1[*][*]1
20. Unknown ECFP_6 feature: -1795620553: [*]CC1CC1

Feature Contribution

Top features for positive contribution			
**Fingerprint**	**Bit/Smiles**	**Feature Structure**	**Score**
ECFP_6	-167460056	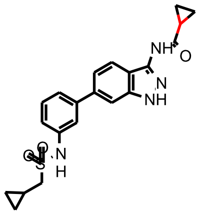 [*]C1[*][*]1	0.136
ECFP_6	1559650422	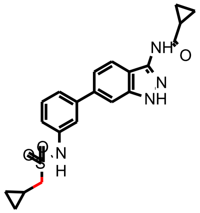 [*]C[*]	0.129
FCFP_6	3	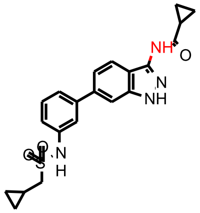 [*]N	0.092

Top Features for negative contribution			
**Fingerprint**	**Bit/Smiles**	**Feature Structure**	**Score**
FCFP_6	991735244	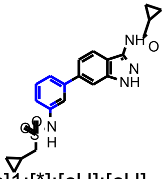 [*][c]1:[*]:[cH]:[cH]:[cH]:[cH]:1	-0.134

FCFP_6	1	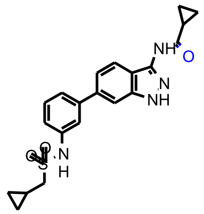<br>[*]=O	-0.102
ECFP_6	-1236483485	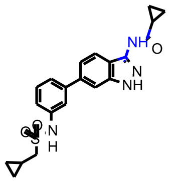<br>[*]C(=[*])N[c](:[*]):<br>[*]	-0.075

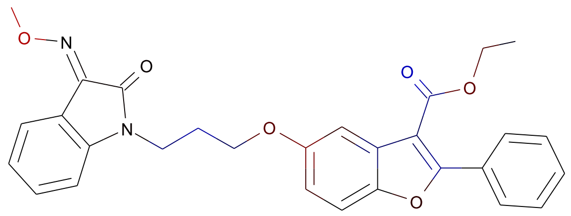

$C_{29}H_{26}N_{2}O_{6}$
Molecular Weight: 498.52653
ALogP: 4.994
Rotatable Bonds: 10
Acceptors: 6
Donors: 0

Model Prediction

Prediction: 0.035
Unit: g/kg_body_weight
Mahalanobis Distance: 10.599
Mahalanobis Distance p-value: 4.44e-006

Mahalanobis Distance: The Mahalanobis distance (MD) is a generalization of the Euclidean distance that accounts for correlations among the X properties. It is calculated as the distance to the center of the training data. The larger the MD, the less trustworthy the prediction.
Mahalanobis Distance p-value: The p-value gives the fraction of training data with an MD greater than or equal to the one for the given sample, assuming normally distributed data. The smaller the p-value, the less trustworthy the prediciton. For highly non-normal X properties (e.g., fingerprints), the MD p-value is wildly inaccurate.

Structural Similar Compounds

Name	RESERPINE	BUTYL BENZYL PHTHALATE	ROTENONE
Structure	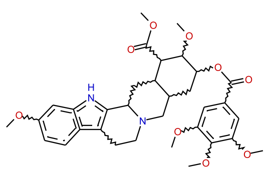	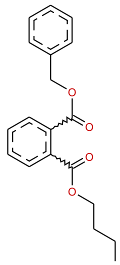	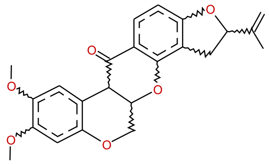
Actual Endpoint (-log C)	6.13118	2.79569	5.06769
Predicted Endpoint (-log C)	4.38304	3.18498	4.11907
Distance	0.779	0.868	0.923
Reference	NCI/NTP TR-193	NCI/NTP TR-458	NCI/NTP TR-320

Model Applicability

Unknown features are fingerprint features in the query molecule, but not found in the training set.

1. All properties and OPS components are within expected ranges.

Feature Contribution

Top features for positive contribution			
**Fingerprint**	**Bit/Smiles**	**Feature Structure**	**Score**
FCFP_2	136627117	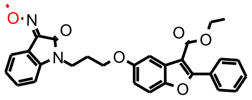 [*]OC	0.173

FCFP_2	-1143715940	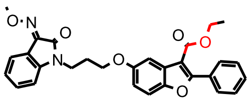 [*]COC(=[*])[*]	0.095
FCFP_2	1036089772	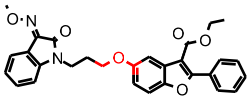 [*]CO[c](:[*]):[*]	0.075
**Top Features for negative contribution**			
**Fingerprint**	**Bit/Smiles**	**Feature Structure**	**Score**
FCFP_2	-1272798659	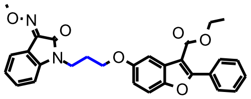 [*]CCC[*]	-0.111
FCFP_2	1872154524	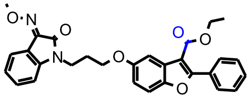 [*]C(=O)[*]	-0.105
FCFP_2	203677720	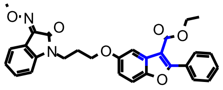 [*]C(=[*])[c]1:[c]([*]):[*]:[*]:[c]:1:[*]	-0.083

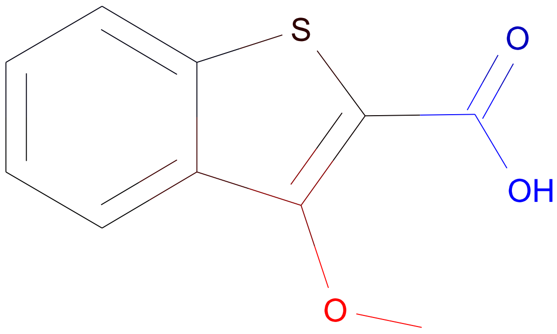

$C_{10}H_8O_3S$
Molecular Weight: 208.23372
ALogP: 2.596
Rotatable Bonds: 2
Acceptors: 3
Donors: 1

Model Prediction

Prediction: 0.115
Unit: g/kg_body_weight
Mahalanobis Distance: 8.833
Mahalanobis Distance p-value: 0.00238

Mahalanobis Distance: The Mahalanobis distance (MD) is a generalization of the Euclidean distance that accounts for correlations among the X properties. It is calculated as the distance to the center of the training data. The larger the MD, the less trustworthy the prediction.
Mahalanobis Distance p-value: The p-value gives the fraction of training data with an MD greater than or equal to the one for the given sample, assuming normally distributed data. The smaller the p-value, the less trustworthy the prediciton. For highly non-normal X properties (e.g., fingerprints), the MD p-value is wildly inaccurate.

Structural Similar Compounds

Name	6-NITROBENZIMIDAZOLE	2-AMINOANTHRAQUINONE	1-AMINO-2-METHYLANTHRAQUINONE
Structure	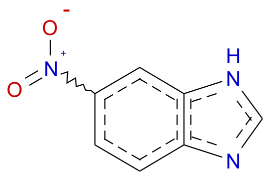	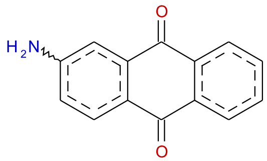	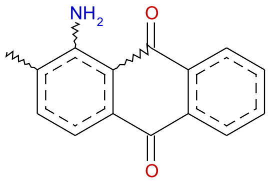
Actual Endpoint (-log C)	2.86037	2.85669	3.42098
Predicted Endpoint (-log C)	3.14119	3.39913	3.49868
Distance	0.472	0.520	0.524
Reference	NCI/NTP TR-117	NCI/NTP TR-144	NCI/NTP TR-111

Model Applicability

Unknown features are fingerprint features in the query molecule, but not found in the training set.

1. All properties and OPS components are within expected ranges.

Feature Contribution

Top features for positive contribution

Fingerprint	Bit/Smiles	Feature Structure	Score
FCFP_2	136627117	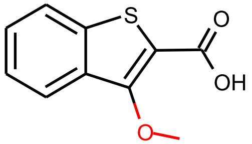 [*]OC	0.173

FCFP_2	1036089772	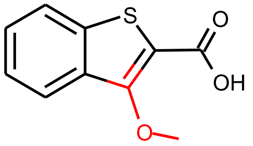<br>[*]CO[c](:[*]):[*]	0.075
FCFP_2	332760439	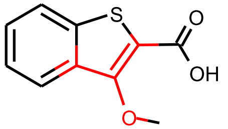<br>[*]O[c](:[cH]:[*]):[cH]:[*]	0.061

Top Features for negative contribution

**Fingerprint**	**Bit/Smiles**	**Feature Structure**	**Score**
FCFP_2	7	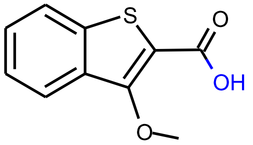<br>[*]O	-0.214
FCFP_2	-548632217	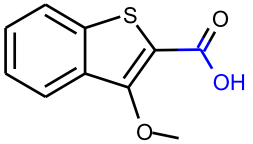<br>[*]C(=[*])O	-0.119
FCFP_2	1872154524	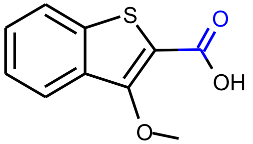<br>[*]C(=O)[*]	-0.105

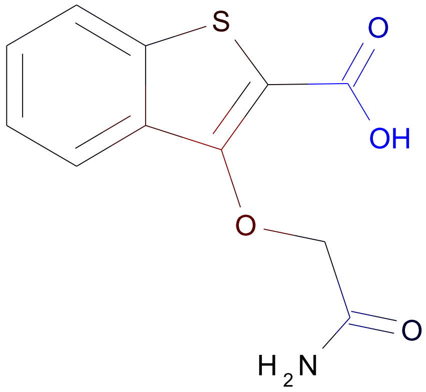

$C_{11}H_9NO_4S$

Molecular Weight: 251.25846

ALogP: 1.45

Rotatable Bonds: 4

Acceptors: 4

Donors: 2

Model Prediction

Prediction: 0.210

Unit: g/kg_body_weight

Mahalanobis Distance: 11.340

Mahalanobis Distance p-value: 2.14e-007

Mahalanobis Distance: The Mahalanobis distance (MD) is a generalization of the Euclidean distance that accounts for correlations among the X properties. It is calculated as the distance to the center of the training data. The larger the MD, the less trustworthy the prediction.

Mahalanobis Distance p-value: The p-value gives the fraction of training data with an MD greater than or equal to the one for the given sample, assuming normally distributed data. The smaller the p-value, the less trustworthy the prediciton. For highly non-normal X properties (e.g., fingerprints), the MD p-value is wildly inaccurate.

Structural Similar Compounds

Name	FUROSEMIDE	DAPSONE	NITROFURAZONE
Structure	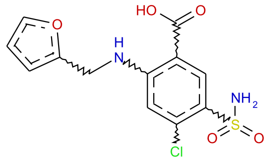	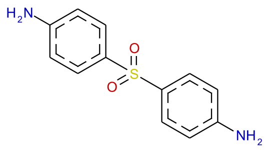	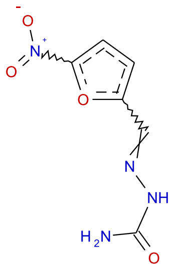
Actual Endpoint (-log C)	4.04236	3.66258	4.21779
Predicted Endpoint (-log C)	2.8614	3.26993	3.40885
Distance	0.503	0.529	0.553
Reference	NCI/NTP TR-356	NCI/NTP TR-20	NCI/NTP TR-337

Model Applicability

Unknown features are fingerprint features in the query molecule, but not found in the training set.

1. OPS PC12 out of range. Value: 2.954. Training min, max, SD, explained variance: -2.364, 2.9228, 1.079, 0.0263.

Feature Contribution

Top features for positive contribution

Fingerprint	Bit/Smiles	Feature Structure	Score
FCFP_2	1036089772	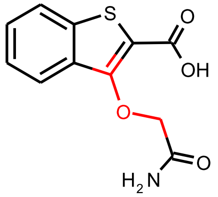 [*]CO[c](:[*]):[*]	0.075

FCFP_2	3	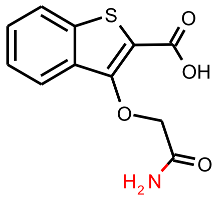 [*]N	0.074
FCFP_2	332760439	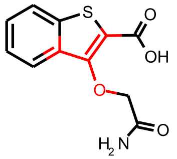 [*]O[c](:[cH]:[*]):[cH]:[*]	0.061

Top Features for negative contribution

Fingerprint	Bit/Smiles	Feature Structure	Score
FCFP_2	7	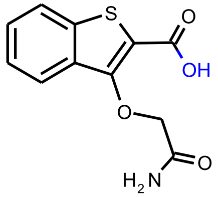 [*]O	-0.214
FCFP_2	-548632217	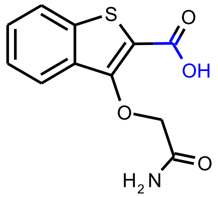 [*]C(=[*])O	-0.119
FCFP_2	1872154524	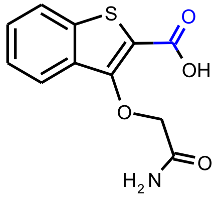 [*]C(=O)[*]	-0.105

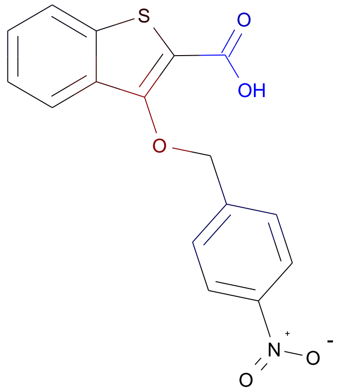

$C_{16}H_{11}NO_5S$
Molecular Weight: 329.32724
ALogP: 4.074
Rotatable Bonds: 5
Acceptors: 5
Donors: 1

Model Prediction

Prediction: 0.190
Unit: g/kg_body_weight
Mahalanobis Distance: 8.839
Mahalanobis Distance p-value: 0.00234

Mahalanobis Distance: The Mahalanobis distance (MD) is a generalization of the Euclidean distance that accounts for correlations among the X properties. It is calculated as the distance to the center of the training data. The larger the MD, the less trustworthy the prediction.
Mahalanobis Distance p-value: The p-value gives the fraction of training data with an MD greater than or equal to the one for the given sample, assuming normally distributed data. The smaller the p-value, the less trustworthy the prediciton. For highly non-normal X properties (e.g., fingerprints), the MD p-value is wildly inaccurate.

Structural Similar Compounds

Name	C.I.PIGMENT RED 3	SALICYLAZOSULFAPYRIDINE	FUROSEMIDE
Structure	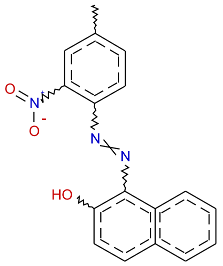	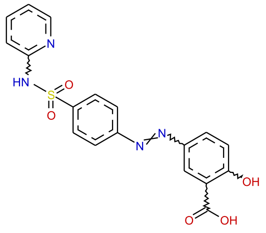	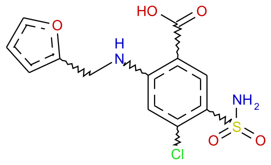
Actual Endpoint (-log C)	2.65635	3.375	4.04236
Predicted Endpoint (-log C)	2.97957	2.80292	2.8614
Distance	0.595	0.736	0.759
Reference	NCI/NTP TR-407	NCI/NTP TR-457	NCI/NTP TR-356

Model Applicability

Unknown features are fingerprint features in the query molecule, but not found in the training set.

1. All properties and OPS components are within expected ranges.
2. Unknown FCFP_2 feature: 8: [*][N+](=[*])[*]
3. Unknown FCFP_2 feature: 5: [*][O-]
4. Unknown FCFP_2 feature: -828984032: [*][N+](=[*])[c](:[cH]:[*]):[cH]:[*]
5. Unknown FCFP_2 feature: -1338588315: [*]:[c](:[*])[N+](=O)[O-]
6. Unknown FCFP_2 feature: 1872392852: [*][N+](=O)[*]
7. Unknown FCFP_2 feature: 260476081: [*][N+](=[*])[O-]

Feature Contribution

Top features for positive contribution

Fingerprint	Bit/Smiles	Feature Structure	Score

FCFP_2	1036089772	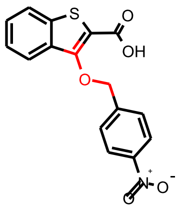 [*]CO[c](:[*]):[*]	0.075
FCFP_2	332760439	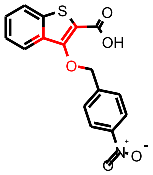 [*]O[c](:[cH]:[*]):[cH]:[*]	0.061
FCFP_2	17	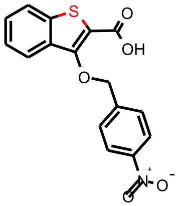 [*]:o:[*]	0.044

Top Features for negative contribution

Fingerprint	Bit/Smiles	Feature Structure	Score
FCFP_2	7	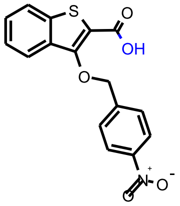 [*]O	-0.214
FCFP_2	-548632217	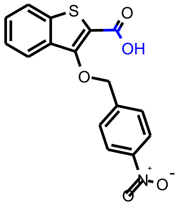 [*]C(=[*])O	-0.119

FCFP_2	1872154524	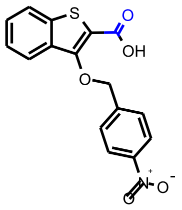 [*]C(=O)[*]	-0.105

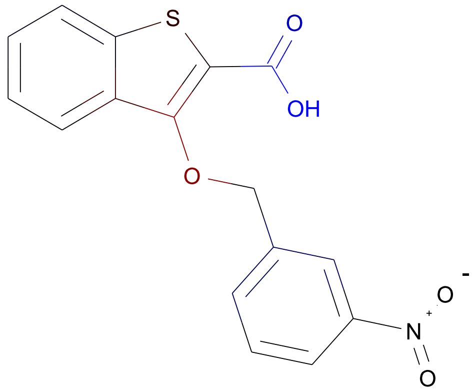

$C_{16}H_{11}NO_5S$

Molecular Weight: 329.32724

ALogP: 4.074

Rotatable Bonds: 5

Acceptors: 5

Donors: 1

Structural Similar Compounds

Name	C.I.PIGMENT RED 3	SALICYLAZOSULFAPYRIDINE	FUROSEMIDE
Structure	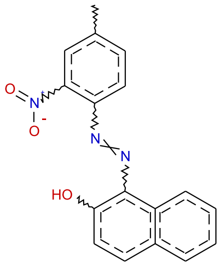	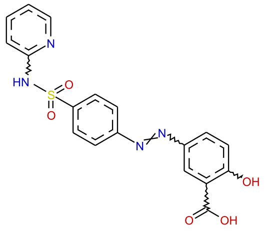	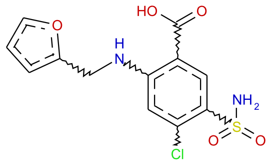
Actual Endpoint (-log C)	2.65635	3.375	4.04236
Predicted Endpoint (-log C)	2.97957	2.80292	2.8614
Distance	0.595	0.736	0.759
Reference	NCI/NTP TR-407	NCI/NTP TR-457	NCI/NTP TR-356

Model Prediction

Prediction: 0.190

Unit: g/kg_body_weight

Mahalanobis Distance: 8.839

Mahalanobis Distance p-value: 0.00234

Mahalanobis Distance: The Mahalanobis distance (MD) is a generalization of the Euclidean distance that accounts for correlations among the X properties. It is calculated as the distance to the center of the training data. The larger the MD, the less trustworthy the prediction.
Mahalanobis Distance p-value: The p-value gives the fraction of training data with an MD greater than or equal to the one for the given sample, assuming normally distributed data. The smaller the p-value, the less trustworthy the prediciton. For highly non-normal X properties (e.g., fingerprints), the MD p-value is wildly inaccurate.

Model Applicability

Unknown features are fingerprint features in the query molecule, but not found in the training set.

1. All properties and OPS components are within expected ranges.
2. Unknown FCFP_2 feature: 8: [*][N+](=[*])[*]
3. Unknown FCFP_2 feature: 5: [*][O-]
4. Unknown FCFP_2 feature: -828984032: [*][N+](=[*])[c](:[cH]:[*]):[cH]:[*]
5. Unknown FCFP_2 feature: -1338588315: [*]:[c](:[*])[N+](=O)[O-]
6. Unknown FCFP_2 feature: 1872392852: [*][N+](=O)[*]
7. Unknown FCFP_2 feature: 260476081: [*][N+](=[*])[O-]

Feature Contribution

Top features for positive contribution			
Fingerprint	Bit/Smiles	Feature Structure	Score

FCFP_2	1036089772	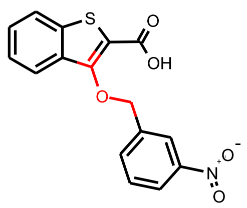 [*]CO[c](:[*]):[*]	0.075
FCFP_2	332760439	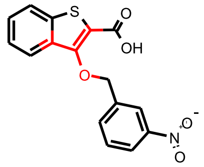 [*]O[c](:[cH]:[*]):[cH]:[*]	0.061
FCFP_2	17	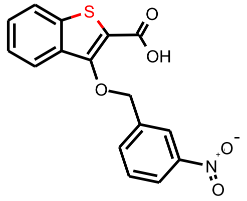 [*]:o:[*]	0.044
**Top Features for negative contribution**			
**Fingerprint**	**Bit/Smiles**	**Feature Structure**	**Score**
FCFP_2	7	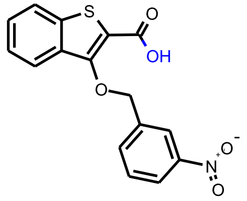 [*]O	-0.214
FCFP_2	-548632217	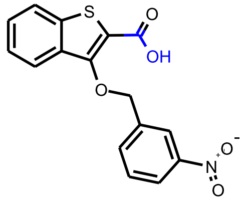 [*]C(=[*])O	-0.119

FCFP_2	1872154524	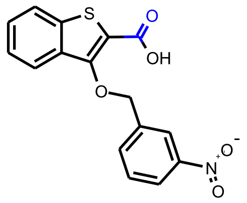 [*]C(=O)[*]	-0.105

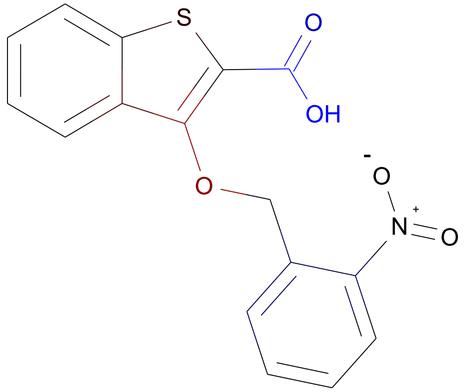

$C_{16}H_{11}NO_5S$
Molecular Weight: 329.32724
ALogP: 4.074
Rotatable Bonds: 5
Acceptors: 5
Donors: 1

Model Prediction

Prediction: 0.190
Unit: g/kg_body_weight
Mahalanobis Distance: 8.839
Mahalanobis Distance p-value: 0.00234

Mahalanobis Distance: The Mahalanobis distance (MD) is a generalization of the Euclidean distance that accounts for correlations among the X properties. It is calculated as the distance to the center of the training data. The larger the MD, the less trustworthy the prediction.
Mahalanobis Distance p-value: The p-value gives the fraction of training data with an MD greater than or equal to the one for the given sample, assuming normally distributed data. The smaller the p-value, the less trustworthy the prediciton. For highly non-normal X properties (e.g., fingerprints), the MD p-value is wildly inaccurate.

Structural Similar Compounds

Name	C.I.PIGMENT RED 3	SALICYLAZOSULFAPYRIDINE	FUROSEMIDE
Structure	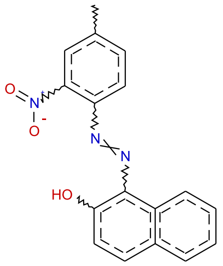	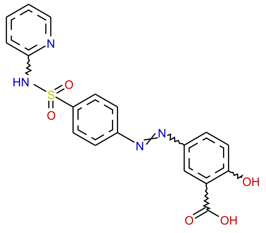	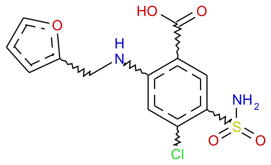
Actual Endpoint (-log C)	2.65635	3.375	4.04236
Predicted Endpoint (-log C)	2.97957	2.80292	2.8614
Distance	0.595	0.736	0.759
Reference	NCI/NTP TR-407	NCI/NTP TR-457	NCI/NTP TR-356

Model Applicability

Unknown features are fingerprint features in the query molecule, but not found in the training set.

1. All properties and OPS components are within expected ranges.
2. Unknown FCFP_2 feature: 8: [*][N+](=[*])[*]
3. Unknown FCFP_2 feature: 5: [*][O-]
4. Unknown FCFP_2 feature: -828984032: [*][N+](=[*])[c](:[cH]:[*]):[cH]:[*]
5. Unknown FCFP_2 feature: -1338588315: [*]:[c](:[*])[N+](=O)[O-]
6. Unknown FCFP_2 feature: 1872392852: [*][N+](=O)[*]
7. Unknown FCFP_2 feature: 260476081: [*][N+](=[*])[O-]

Feature Contribution

Top features for positive contribution			
Fingerprint	Bit/Smiles	Feature Structure	Score

FCFP_2	1036089772	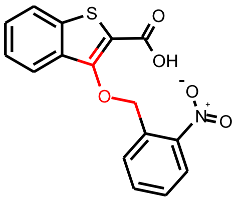 [*]CO[c](:[*]):[*]	0.075
FCFP_2	332760439	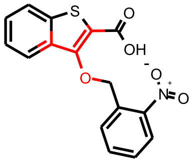 [*]O[c](:[cH]:[*]):[c H]:[*]	0.061
FCFP_2	17	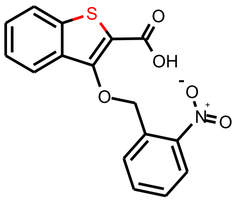 [*]:o:[*]	0.044

Top Features for negative contribution

Fingerprint	Bit/Smiles	Feature Structure	Score
FCFP_2	7	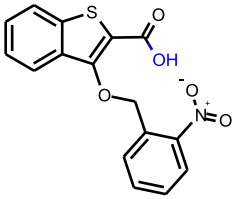 [*]O	-0.214
FCFP_2	-548632217	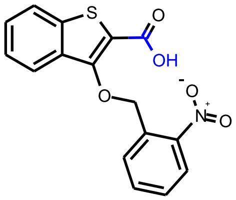 [*]C(=[*])O	-0.119

FCFP_2	1872154524	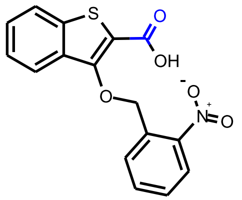 [*]C(=O)[*]	-0.105

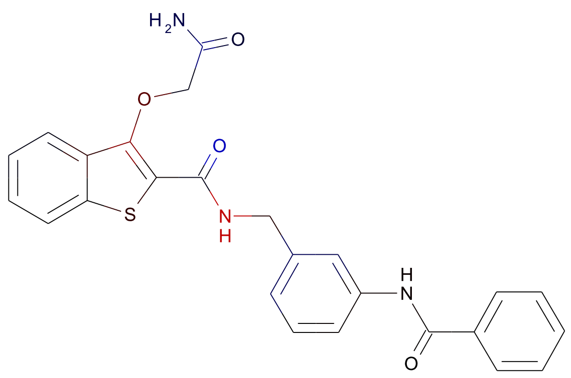

$C_{25}H_{21}N_3O_4S$

Molecular Weight: 459.51694

ALogP: 3.396

Rotatable Bonds: 8

Acceptors: 4

Donors: 3

Model Prediction

Prediction: 0.073

Unit: g/kg_body_weight

Mahalanobis Distance: 13.829

Mahalanobis Distance p-value: 2.94e-012

Mahalanobis Distance: The Mahalanobis distance (MD) is a generalization of the Euclidean distance that accounts for correlations among the X properties. It is calculated as the distance to the center of the training data. The larger the MD, the less trustworthy the prediction.
Mahalanobis Distance p-value: The p-value gives the fraction of training data with an MD greater than or equal to the one for the given sample, assuming normally distributed data. The smaller the p-value, the less trustworthy the prediciton. For highly non-normal X properties (e.g., fingerprints), the MD p-value is wildly inaccurate.

Structural Similar Compounds

Name	SALICYLAZOSULFAPYRIDINE	C.I.PIGMENT RED 23	FUROSEMIDE
Structure	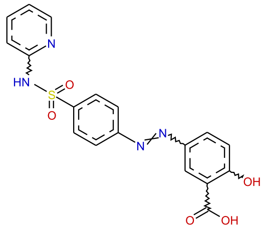	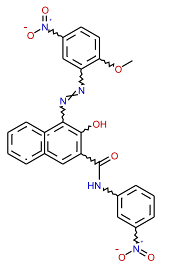	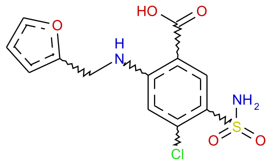
Actual Endpoint (-log C)	3.375	2.30052	4.04236
Predicted Endpoint (-log C)	2.80292	3.55333	2.8614
Distance	0.738	0.774	0.804
Reference	NCI/NTP TR-457	NCI/NTP TR-411	NCI/NTP TR-356

Model Applicability

Unknown features are fingerprint features in the query molecule, but not found in the training set.

1. OPS PC9 out of range. Value: 5.2287. Training min, max, SD, explained variance: -2.8548, 3.3954, 1.263, 0.0360.

Feature Contribution

Top features for positive contribution			
**Fingerprint**	**Bit/Smiles**	**Feature Structure**	**Score**
FCFP_2	-885550502	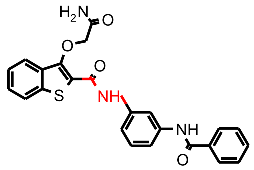 [*]CNC(=[*])[*]	0.115

FCFP_2	1036089772	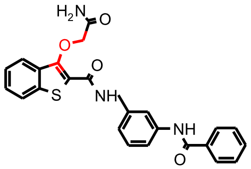<br>[*]CO[c](:[*]):[*]	0.075
FCFP_2	3	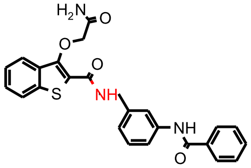<br>[*]N	0.074
**Top Features for negative contribution**			
**Fingerprint**	**Bit/Smiles**	**Feature Structure**	**Score**
FCFP_2	1872154524	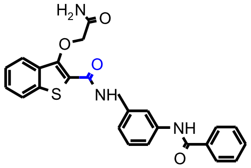<br>[*]C(=O)[*]	-0.105
FCFP_2	203677720	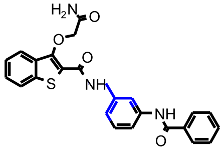<br>[*]C(=[*])[c]1:[c]([*]):[*]:[*]:[c]:1:[*]	-0.083
FCFP_2	1	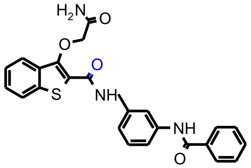<br>[*]=O	-0.080

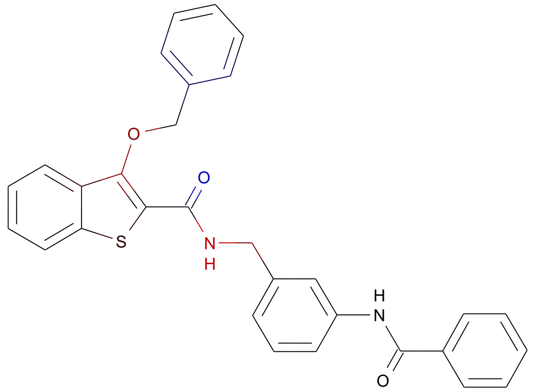

$C_{30}H_{24}N_2O_3S$
Molecular Weight: 492.58816
ALogP: 6.126
Rotatable Bonds: 8
Acceptors: 3
Donors: 2

Structural Similar Compounds

Name	C.I.PIGMENT RED 23	C.I.PIGMENT RED 3	PHENOLPHTHALEIN
Structure	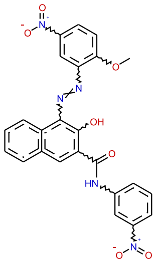	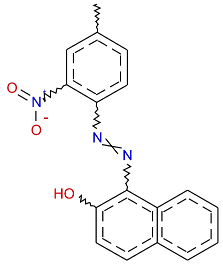	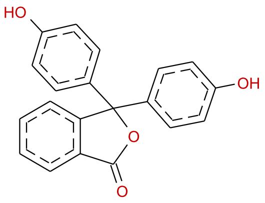
Actual Endpoint (-log C)	2.30052	2.65635	2.20184
Predicted Endpoint (-log C)	3.55333	2.97957	2.8857
Distance	0.958	0.980	1.044
Reference	NCI/NTP TR-411	NCI/NTP TR-407	NCI/NTP TR-465

Model Prediction

Prediction: 0.057
Unit: g/kg_body_weight
Mahalanobis Distance: 11.900
Mahalanobis Distance p-value: 1.93e-008

Mahalanobis Distance: The Mahalanobis distance (MD) is a generalization of the Euclidean distance that accounts for correlations among the X properties. It is calculated as the distance to the center of the training data. The larger the MD, the less trustworthy the prediction.
Mahalanobis Distance p-value: The p-value gives the fraction of training data with an MD greater than or equal to the one for the given sample, assuming normally distributed data. The smaller the p-value, the less trustworthy the prediciton. For highly non-normal X properties (e.g., fingerprints), the MD p-value is wildly inaccurate.

Model Applicability

Unknown features are fingerprint features in the query molecule, but not found in the training set.

1. Num_AromaticRings out of range. Value: 5. Training min, max, mean, SD: 0, 4, 1.1685, 0.8469.
2. OPS PC9 out of range. Value: 4.4697. Training min, max, SD, explained variance: -2.8548, 3.3954, 1.263, 0.0360.

Feature Contribution

Top features for positive contribution

Fingerprint	Bit/Smiles	Feature Structure	Score
FCFP_2	-885550502	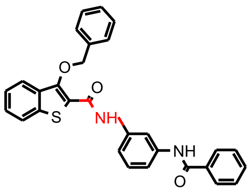 [*]CNC(=[*])[*]	0.115

FCFP_2	1036089772	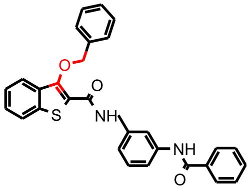 [*]CO[c](:[*]):[*]	0.075
FCFP_2	3	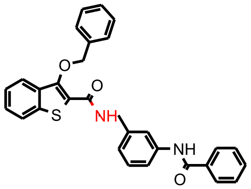 [*]N	0.074

Top Features for negative contribution

Fingerprint	Bit/Smiles	Feature Structure	Score
FCFP_2	1872154524	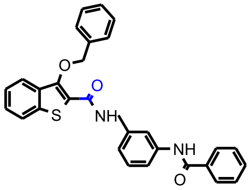 [*]C(=O)[*]	-0.105
FCFP_2	203677720	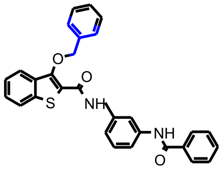 [*]C(=[*])[c]1:[c]([*]):[*]:[*]:[c]:1:[*]	-0.083
FCFP_2	1	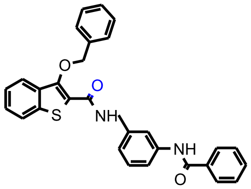 [*]=O	-0.080

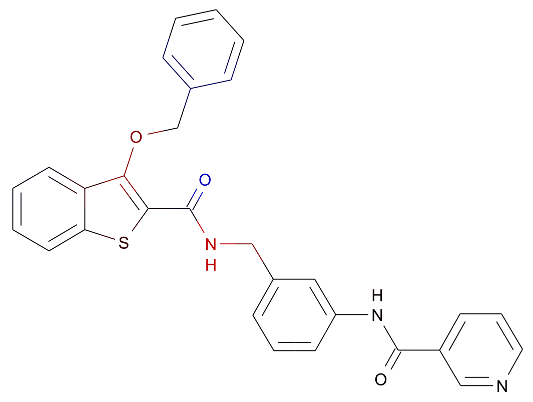

$C_{29}H_{23}N_3O_3S$

Molecular Weight: 493.57622

ALogP: 4.976

Rotatable Bonds: 8

Acceptors: 4

Donors: 2

Model Prediction

Prediction: 0.051

Unit: g/kg_body_weight

Mahalanobis Distance: 11.789

Mahalanobis Distance p-value: 3.12e-008

Mahalanobis Distance: The Mahalanobis distance (MD) is a generalization of the Euclidean distance that accounts for correlations among the X properties. It is calculated as the distance to the center of the training data. The larger the MD, the less trustworthy the prediction.
Mahalanobis Distance p-value: The p-value gives the fraction of training data with an MD greater than or equal to the one for the given sample, assuming normally distributed data. The smaller the p-value, the less trustworthy the prediciton. For highly non-normal X properties (e.g., fingerprints), the MD p-value is wildly inaccurate.

Structural Similar Compounds

Name	C.I.PIGMENT RED 23	SALICYLAZOSULFAPYRIDINE	C.I.PIGMENT RED 3
Structure	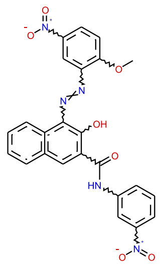	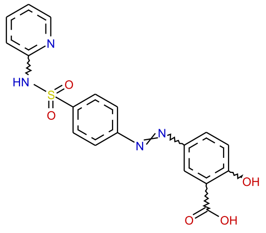	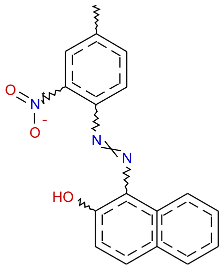
Actual Endpoint (-log C)	2.30052	3.375	2.65635
Predicted Endpoint (-log C)	3.55333	2.80292	2.97957
Distance	0.867	0.941	0.973
Reference	NCI/NTP TR-411	NCI/NTP TR-457	NCI/NTP TR-407

Model Applicability

Unknown features are fingerprint features in the query molecule, but not found in the training set.

1. Num_AromaticRings out of range. Value: 5. Training min, max, mean, SD: 0, 4, 1.1685, 0.8469.
2. OPS PC9 out of range. Value: 4.4518. Training min, max, SD, explained variance: -2.8548, 3.3954, 1.263, 0.0360.

Feature Contribution

Top features for positive contribution

Fingerprint	Bit/Smiles	Feature Structure	Score
FCFP_2	-885550502	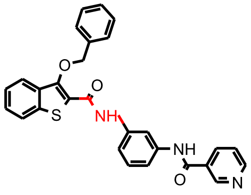 [*]CNC(=[*])[*]	0.115

FCFP_2	1036089772	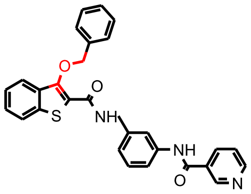 [*]CO[c](:[*]):[*]	0.075
FCFP_2	3	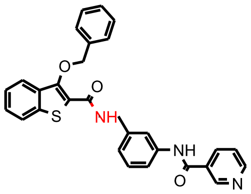 [*]N	0.074

Top Features for negative contribution

Fingerprint	Bit/Smiles	Feature Structure	Score
FCFP_2	1872154524	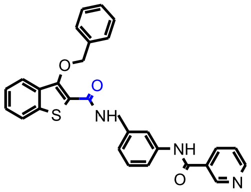 [*]C(=O)[*]	-0.105
FCFP_2	203677720	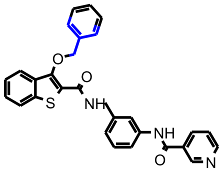 [*]C(=[*])[c]1:[c]([*]):[*]:[*]:[c]:1:[*]	-0.083
FCFP_2	1	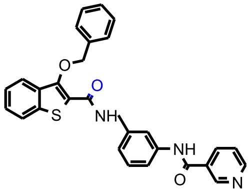 [*]=O	-0.080

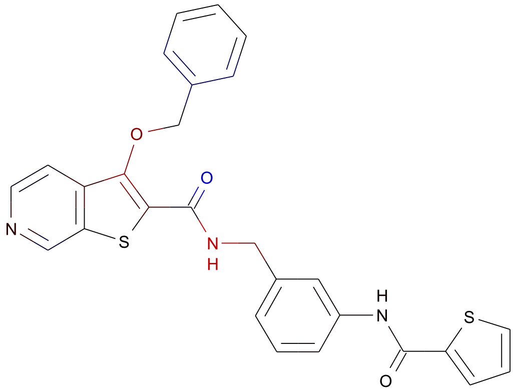

$C_{27}H_{21}N_3O_3S_2$
Molecular Weight: 499.60394
ALogP: 4.929
Rotatable Bonds: 8
Acceptors: 4
Donors: 2

Model Prediction

Prediction: 0.037
Unit: g/kg_body_weight
Mahalanobis Distance: 12.774
Mahalanobis Distance p-value: 3.89e-010

Mahalanobis Distance: The Mahalanobis distance (MD) is a generalization of the Euclidean distance that accounts for correlations among the X properties. It is calculated as the distance to the center of the training data. The larger the MD, the less trustworthy the prediction.
Mahalanobis Distance p-value: The p-value gives the fraction of training data with an MD greater than or equal to the one for the given sample, assuming normally distributed data. The smaller the p-value, the less trustworthy the prediciton. For highly non-normal X properties (e.g., fingerprints), the MD p-value is wildly inaccurate.

Structural Similar Compounds

Name	C.I.PIGMENT RED 23	SALICYLAZOSULFAPYRIDINE	C.I.PIGMENT RED 3
Structure	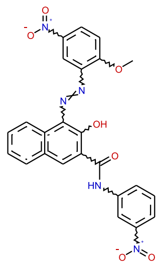	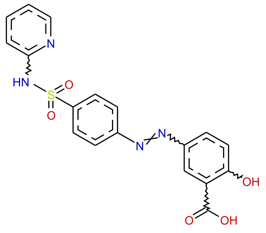	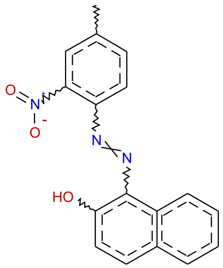
Actual Endpoint (-log C)	2.30052	3.375	2.65635
Predicted Endpoint (-log C)	3.55333	2.80292	2.97957
Distance	0.773	0.897	1.024
Reference	NCI/NTP TR-411	NCI/NTP TR-457	NCI/NTP TR-407

Model Applicability

Unknown features are fingerprint features in the query molecule, but not found in the training set.

1. Num_AromaticRings out of range. Value: 5. Training min, max, mean, SD: 0, 4, 1.1685, 0.8469.
2. OPS PC9 out of range. Value: 4.4167. Training min, max, SD, explained variance: -2.8548, 3.3954, 1.263, 0.0360.

Feature Contribution

Top features for positive contribution

Fingerprint	Bit/Smiles	Feature Structure	Score
FCFP_2	-885550502	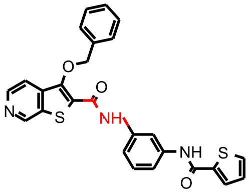 [*]CNC(=[*])[*]	0.115

FCFP_2	1036089772	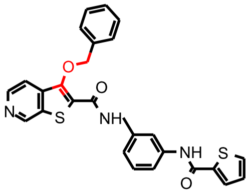 [*]CO[c](:[*]):[*]	0.075
FCFP_2	3	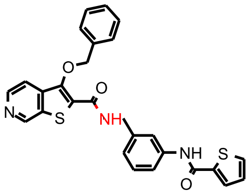 [*]N	0.074
**Top Features for negative contribution**			
**Fingerprint**	**Bit/Smiles**	**Feature Structure**	**Score**
FCFP_2	1872154524	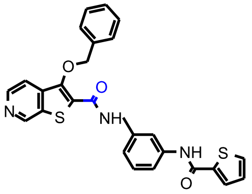 [*]C(=O)[*]	-0.105
FCFP_2	203677720	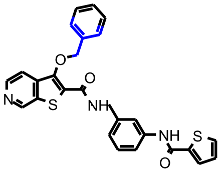 [*]C(=[*])[c]1:[c]([*]):[*]:[*]:[c]:1:[*]	-0.083
FCFP_2	1	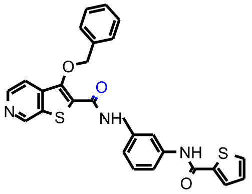 [*]=O	-0.080

Co-crystallized ligand

TOPKAT_Rat_Maximum_Tolerated_Dose_Feed

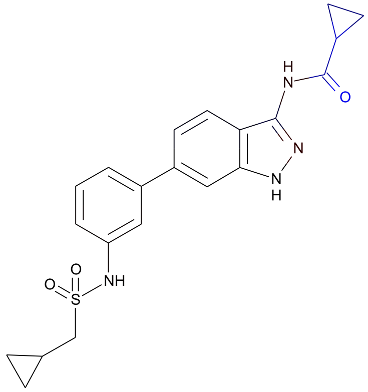

$C_{21}H_{22}N_4O_3S$

Molecular Weight: 410.48938

ALogP: 3.14

Rotatable Bonds: 7

Acceptors: 4

Donors: 3

Model Prediction

Prediction: 0.161

Unit: g/kg_body_weight

Mahalanobis Distance: 9.901

Mahalanobis Distance p-value: 6.41e-005

Mahalanobis Distance: The Mahalanobis distance (MD) is a generalization of the Euclidean distance that accounts for correlations among the X properties. It is calculated as the distance to the center of the training data. The larger the MD, the less trustworthy the prediction.

Mahalanobis Distance p-value: The p-value gives the fraction of training data with an MD greater than or equal to the one for the given sample, assuming normally distributed data. The smaller the p-value, the less trustworthy the prediciton. For highly non-normal X properties (e.g., fingerprints), the MD p-value is wildly inaccurate.

Structural Similar Compounds

Name	FUROSEMIDE	SALICYLAZOSULFAPYRIDINE	DISPERSE YELLOW 3
Structure	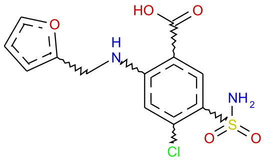	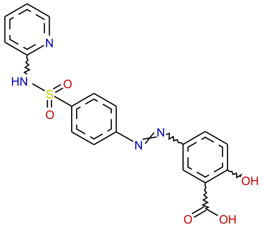	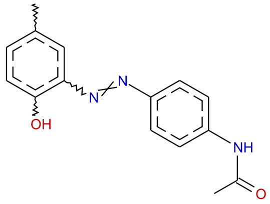
Actual Endpoint (-log C)	4.04236	3.375	2.77703
Predicted Endpoint (-log C)	2.8614	2.80292	2.80195
Distance	0.728	0.749	0.817
Reference	NCI/NTP TR-356	NCI/NTP TR-457	NCI/NTP TR-222

Model Applicability

Unknown features are fingerprint features in the query molecule, but not found in the training set.

1. OPS PC9 out of range. Value: 4.3482. Training min, max, SD, explained variance: -2.8548, 3.3954, 1.263, 0.0360.
2. Unknown FCFP_2 feature: 1747267175: [*][c]1:[*]:[*]:[nH]:n:1
3. Unknown FCFP_2 feature: 1018942292: [*]CS(=O)(=O)N[*]

Feature Contribution

Top features for positive contribution

Fingerprint	Bit/Smiles	Feature Structure	Score
FCFP_2	3	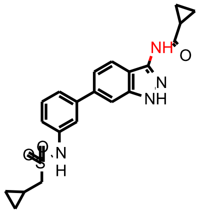 [*]N	0.074

FCFP_2	17	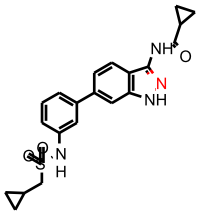 [*]:o:[*]	0.044
FCFP_2	590925877	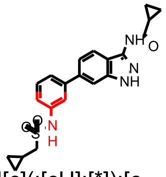 [*]N[c](:[cH]:[*]):[cH]:[*]	0.008

Top Features for negative contribution

Fingerprint	Bit/Smiles	Feature Structure	Score
FCFP_2	-1272798659	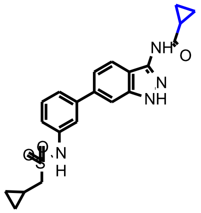 [*]CCC[*]	-0.111
FCFP_2	1872154524	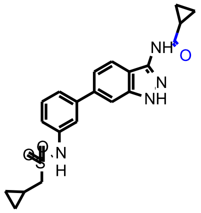 [*]C(=O)[*]	-0.105
FCFP_2	1	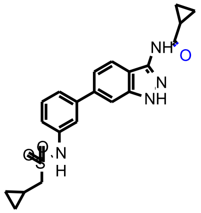 [*]=O	-0.080

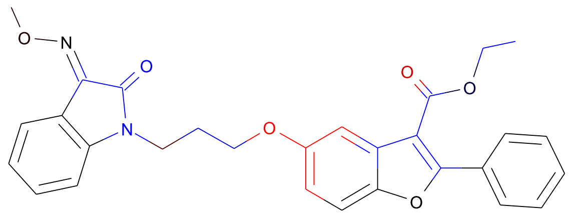

$C_{29}H_{26}N_2O_6$
Molecular Weight: 498.52653
ALogP: 4.994
Rotatable Bonds: 10
Acceptors: 6
Donors: 0

Model Prediction

Prediction: 0.027
Unit: g/kg_body_weight
Mahalanobis Distance: 11.662
Mahalanobis Distance p-value: 2.67e-008

Mahalanobis Distance: The Mahalanobis distance (MD) is a generalization of the Euclidean distance that accounts for correlations among the X properties. It is calculated as the distance to the center of the training data. The larger the MD, the less trustworthy the prediction.
Mahalanobis Distance p-value: The p-value gives the fraction of training data with an MD greater than or equal to the one for the given sample, assuming normally distributed data. The smaller the p-value, the less trustworthy the prediciton. For highly non-normal X properties (e.g., fingerprints), the MD p-value is wildly inaccurate.

Structural Similar Compounds

Name	PHENYLBUTAZONE	8-METHOXYPSORALEN	OCHRATOXIN
Structure	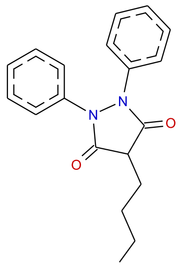	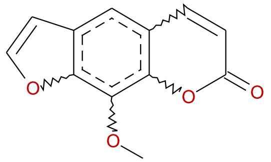	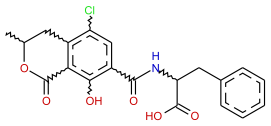
Actual Endpoint (-log C)	3.48909	3.45978	6.28396
Predicted Endpoint (-log C)	3.17333	4.14745	5.12358
Distance	1.241	1.348	1.349
Reference	NCI/NTP TR-367	NCI/NTP TR-359	NCI/NTP TR-358

Model Applicability

Unknown features are fingerprint features in the query molecule, but not found in the training set.

1. Molecular_Weight out of range. Value: 498.53. Training min, max, mean, SD: 68.074, 434.63, 171.13, 85.06.
2. Num_AromaticRings out of range. Value: 4. Training min, max, mean, SD: 0, 2, 0.5625, 0.693.
3. OPS PC3 out of range. Value: 5.5461. Training min, max, SD, explained variance: -4.6235, 5.1158, 1.773, 0.0972.
4. OPS PC6 out of range. Value: -3.31. Training min, max, SD, explained variance: -2.4321, 2.9885, 1.256, 0.0488.
5. Unknown FCFP_2 feature: 690511177: [*][c]1:[*]:[*]:o:[c]:1[c](:[*]):[*]
6. Unknown FCFP_2 feature: -1861645784: [*]:[cH]:[c](:[cH]:[*])[c](:[*]):[*]
7. Unknown FCFP_2 feature: -1549192822: [*]\N=C/1\C(=[*])[*][*]:[c]1:[*]
8. Unknown FCFP_2 feature: 580960234: [*]ON=C([*])[*]
9. Unknown FCFP_2 feature: -1143686149: [*]=NOC

Feature Contribution

Top features for positive contribution			
Fingerprint	Bit/Smiles	Feature Structure	Score

FCFP_2	332760439	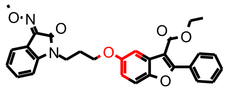 [*]O[c](:[cH]:[*]):[cH]:[*]	0.672
FCFP_2	1	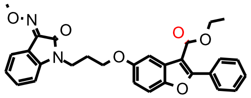 [*]=O	0.511
FCFP_2	-1272798659	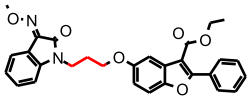 [*]CCC[*]	0.070

Top Features for negative contribution

**Fingerprint**	**Bit/Smiles**	**Feature Structure**	**Score**
FCFP_2	136597326	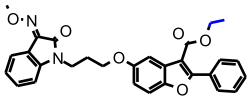 [*]CC	-0.489
FCFP_2	203677720	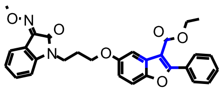 [*]C(=[*])[c]1:[c]([*]):[*]:[*]:[c]:1:[*]	-0.406

FCFP_2	565998553	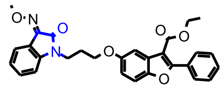<br>[*]N1[*]:[*]C(=[*])C1<br>=O	-0.348

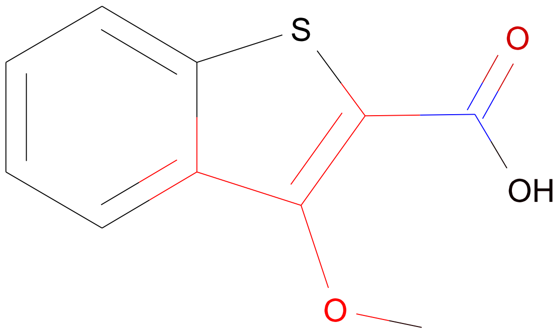

$C_{10}H_8O_3S$
Molecular Weight: 208.23372
ALogP: 2.596
Rotatable Bonds: 2
Acceptors: 3
Donors: 1

Structural Similar Compounds

Name	2-MERCAPTOBENZOTHIAZOLE	8-METHOXYPSORALEN	M-CRESIDINE
Structure	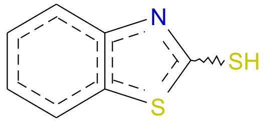	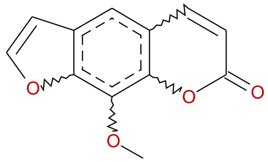	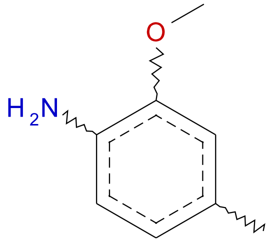
Actual Endpoint (-log C)	2.34829	3.45978	5.93318
Predicted Endpoint (-log C)	3.82125	4.14745	3.87056
Distance	0.530	0.538	0.773
Reference	NCI/NTP TR-332	NCI/NTP TR-359	NCI/NTP TR-105

Model Prediction

Prediction: 0.010
Unit: g/kg_body_weight
Mahalanobis Distance: 10.181
Mahalanobis Distance p-value: 3.2e-006

Mahalanobis Distance: The Mahalanobis distance (MD) is a generalization of the Euclidean distance that accounts for correlations among the X properties. It is calculated as the distance to the center of the training data. The larger the MD, the less trustworthy the prediction.
Mahalanobis Distance p-value: The p-value gives the fraction of training data with an MD greater than or equal to the one for the given sample, assuming normally distributed data. The smaller the p-value, the less trustworthy the prediciton. For highly non-normal X properties (e.g., fingerprints), the MD p-value is wildly inaccurate.

Model Applicability

Unknown features are fingerprint features in the query molecule, but not found in the training set.

1. All properties and OPS components are within expected ranges.

Feature Contribution

Top features for positive contribution

Fingerprint	Bit/Smiles	Feature Structure	Score
FCFP_2	332760439	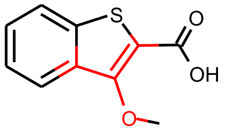 [*]O[c](:[cH]:[*]):[cH]:[*]	0.672

FCFP_2	1	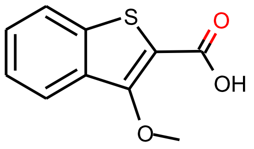 [*]=O	0.511
FCFP_2	136627117	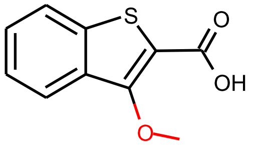 [*]OC	0.030

Top Features for negative contribution

Fingerprint	Bit/Smiles	Feature Structure	Score
FCFP_2	1872154524	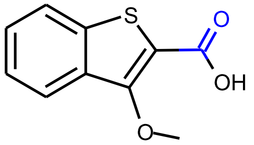 [*]C(=O)[*]	-0.307
FCFP_2	0	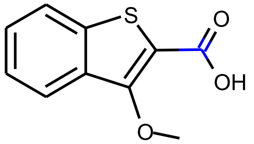 [*]C(=[*])[*]	-0.290

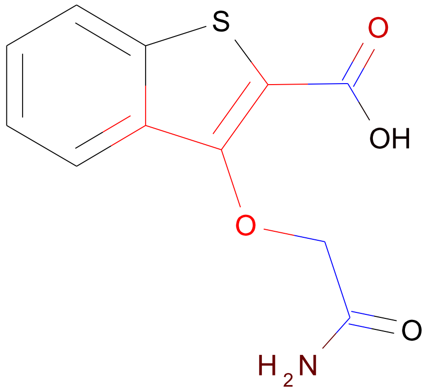

$C_{11}H_9NO_4S$

Molecular Weight: 251.25846

ALogP: 1.45

Rotatable Bonds: 4

Acceptors: 4

Donors: 2

Model Prediction

Prediction: 0.055

Unit: g/kg_body_weight

Mahalanobis Distance: 11.482

Mahalanobis Distance p-value: 4.81e-008

Mahalanobis Distance: The Mahalanobis distance (MD) is a generalization of the Euclidean distance that accounts for correlations among the X properties. It is calculated as the distance to the center of the training data. The larger the MD, the less trustworthy the prediction.
Mahalanobis Distance p-value: The p-value gives the fraction of training data with an MD greater than or equal to the one for the given sample, assuming normally distributed data. The smaller the p-value, the less trustworthy the prediciton. For highly non-normal X properties (e.g., fingerprints), the MD p-value is wildly inaccurate.

Structural Similar Compounds

Name	SULFISOOXAZOLE	PENICILLIN VK	HC RED 3
Structure	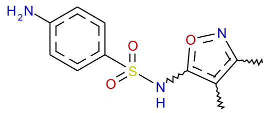	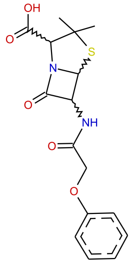	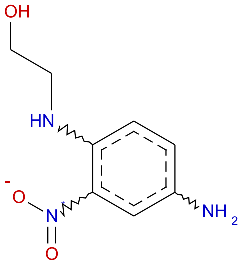
Actual Endpoint (-log C)	2.82494	2.54455	2.59592
Predicted Endpoint (-log C)	3.0705	3.9702	3.285
Distance	0.491	0.708	0.761
Reference	NCI/NTP TR-138	NCI/NTP TR-336	NCI/NTP TR-281

Model Applicability

Unknown features are fingerprint features in the query molecule, but not found in the training set.

1. All properties and OPS components are within expected ranges.

Feature Contribution

Top features for positive contribution

Fingerprint	Bit/Smiles	Feature Structure	Score
FCFP_2	332760439	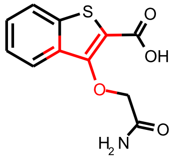 [*]O[c](:[cH]:[*]):[cH]:[*]	0.672

FCFP_2	1	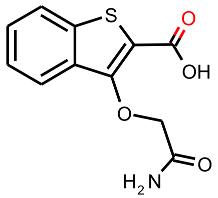<br>[*]=O	0.511
FCFP_2	3	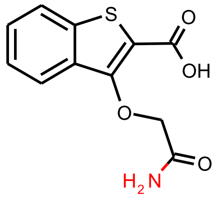<br>[*]N	0.104
**Top Features for negative contribution**			
**Fingerprint**	**Bit/Smiles**	**Feature Structure**	**Score**
FCFP_2	1872154524	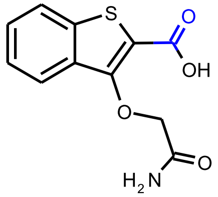<br>[*]C(=O)[*]	-0.307
FCFP_2	0	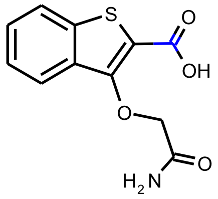<br>[*]C(=[*])[*]	-0.290
FCFP_2	-1272768868	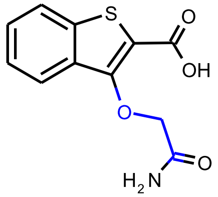<br>[*]CCO[*]	-0.271

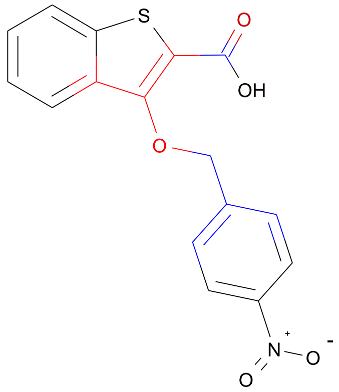

$C_{16}H_{11}NO_5S$
Molecular Weight: 329.32724
ALogP: 4.074
Rotatable Bonds: 5
Acceptors: 5
Donors: 1

Model Prediction

Prediction: 0.009
Unit: g/kg_body_weight
Mahalanobis Distance: 10.586
Mahalanobis Distance p-value: 8.78e-007

Mahalanobis Distance: The Mahalanobis distance (MD) is a generalization of the Euclidean distance that accounts for correlations among the X properties. It is calculated as the distance to the center of the training data. The larger the MD, the less trustworthy the prediction.
Mahalanobis Distance p-value: The p-value gives the fraction of training data with an MD greater than or equal to the one for the given sample, assuming normally distributed data. The smaller the p-value, the less trustworthy the prediciton. For highly non-normal X properties (e.g., fingerprints), the MD p-value is wildly inaccurate.

Structural Similar Compounds

Name	SULFISOOXAZOLE	OCHRATOXIN	PROBENECID
Structure	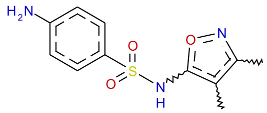	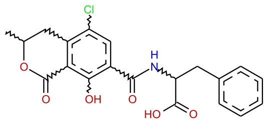	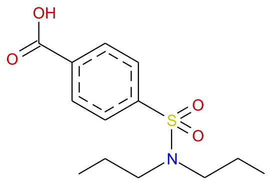
Actual Endpoint (-log C)	2.82494	6.28396	2.85333
Predicted Endpoint (-log C)	3.0705	5.12358	2.4258
Distance	0.903	0.938	0.990
Reference	NCI/NTP TR-138	NCI/NTP TR-358	NCI/NTP TR-395

Model Applicability

Unknown features are fingerprint features in the query molecule, but not found in the training set.

1. Num_AromaticRings out of range. Value: 3. Training min, max, mean, SD: 0, 2, 0.5625, 0.693.
2. OPS PC6 out of range. Value: -3.1049. Training min, max, SD, explained variance: -2.4321, 2.9885, 1.256, 0.0488.
3. Unknown FCFP_2 feature: 8: [*][N+](=[*])[*]
4. Unknown FCFP_2 feature: 5: [*][O-]
5. Unknown FCFP_2 feature: 907036844: [*]OC[c](:[*]):[*]
6. Unknown FCFP_2 feature: -828984032: [*][N+](=[*])[c](:[cH]:[*]):[cH]:[*]
7. Unknown FCFP_2 feature: -1338588315: [*]:[c](:[*])[N+](=O)[O-]
8. Unknown FCFP_2 feature: 1872392852: [*][N+](=O)[*]
9. Unknown FCFP_2 feature: 260476081: [*][N+](=[*])[O-]

Feature Contribution

Top features for positive contribution

Fingerprint	Bit/Smiles	Feature Structure	Score

FCFP_2	332760439	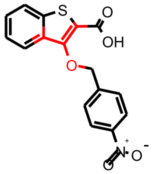 [*]O[c](:[cH]:[*]):[cH]:[*]	0.672
FCFP_2	1	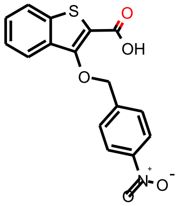 [*]=O	0.511
FCFP_2	7	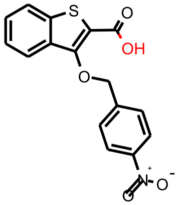 [*]O	0.014

Top Features for negative contribution

Fingerprint	Bit/Smiles	Feature Structure	Score
FCFP_2	203677720	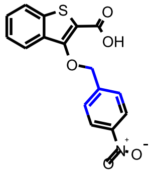 [*]C(=[*])[c]1:[c]([*]):[*]:[*]:[c]:1:[*]	-0.406
FCFP_2	1872154524	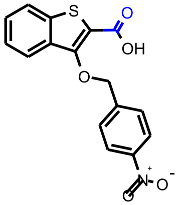 [*]C(=O)[*]	-0.307

FCFP_2	0	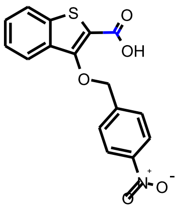 [*]C(=[*])[*]	-0.290

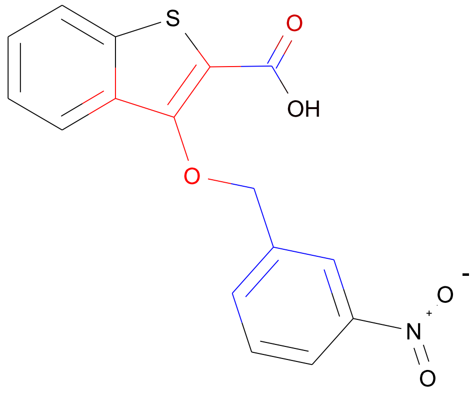

$C_{16}H_{11}NO_5S$
Molecular Weight: 329.32724
ALogP: 4.074
Rotatable Bonds: 5
Acceptors: 5
Donors: 1

Model Prediction

Prediction: 0.009
Unit: g/kg_body_weight
Mahalanobis Distance: 10.586
Mahalanobis Distance p-value: 8.78e-007

Mahalanobis Distance: The Mahalanobis distance (MD) is a generalization of the Euclidean distance that accounts for correlations among the X properties. It is calculated as the distance to the center of the training data. The larger the MD, the less trustworthy the prediction.
Mahalanobis Distance p-value: The p-value gives the fraction of training data with an MD greater than or equal to the one for the given sample, assuming normally distributed data. The smaller the p-value, the less trustworthy the prediciton. For highly non-normal X properties (e.g., fingerprints), the MD p-value is wildly inaccurate.

Structural Similar Compounds

Name	SULFISOOXAZOLE	OCHRATOXIN	PROBENECID
Structure	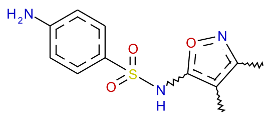	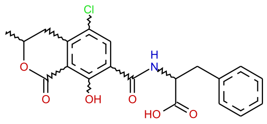	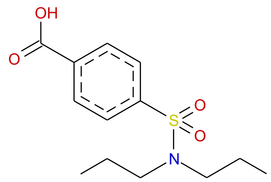
Actual Endpoint (-log C)	2.82494	6.28396	2.85333
Predicted Endpoint (-log C)	3.0705	5.12358	2.4258
Distance	0.903	0.938	0.990
Reference	NCI/NTP TR-138	NCI/NTP TR-358	NCI/NTP TR-395

Model Applicability

Unknown features are fingerprint features in the query molecule, but not found in the training set.

1. Num_AromaticRings out of range. Value: 3. Training min, max, mean, SD: 0, 2, 0.5625, 0.693.
2. OPS PC6 out of range. Value: -3.1049. Training min, max, SD, explained variance: -2.4321, 2.9885, 1.256, 0.0488.
3. Unknown FCFP_2 feature: 8: [*][N+](=[*])[*]
4. Unknown FCFP_2 feature: 5: [*][O-]
5. Unknown FCFP_2 feature: 907036844: [*]OC[c](:[*]):[*]
6. Unknown FCFP_2 feature: -828984032: [*][N+](=[*])[c](:[cH]:[*]):[cH]:[*]
7. Unknown FCFP_2 feature: -1338588315: [*]:[c](:[*])[N+](=O)[O-]
8. Unknown FCFP_2 feature: 1872392852: [*][N+](=O)[*]
9. Unknown FCFP_2 feature: 260476081: [*][N+](=[*])[O-]

Feature Contribution

Top features for positive contribution

Fingerprint	Bit/Smiles	Feature Structure	Score

FCFP_2	332760439	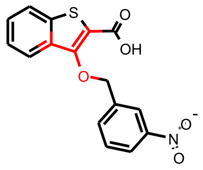 [*]O[c](:[cH]:[*]):[cH]:[*]	0.672
FCFP_2	1	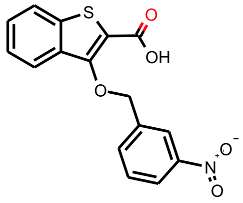 [*]=O	0.511
FCFP_2	7	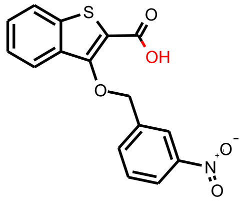 [*]O	0.014
**Top Features for negative contribution**			
**Fingerprint**	**Bit/Smiles**	**Feature Structure**	**Score**
FCFP_2	203677720	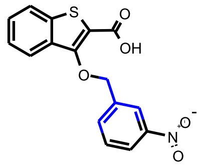 [*]C(=[*])[c]1:[c]([*]):[*]:[*]:[c]:1:[*]	-0.406
FCFP_2	1872154524	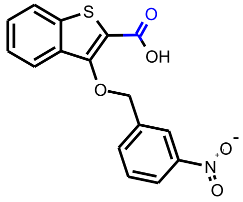 [*]C(=O)[*]	-0.307

FCFP_2	0	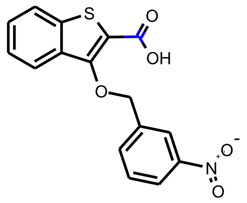 [*]C(=[*])[*]	-0.290

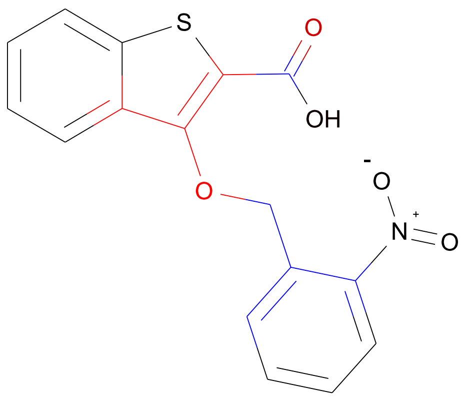

$C_{16}H_{11}NO_5S$
Molecular Weight: 329.32724
ALogP: 4.074
Rotatable Bonds: 5
Acceptors: 5
Donors: 1

Model Prediction

Prediction: 0.009
Unit: g/kg_body_weight
Mahalanobis Distance: 10.586
Mahalanobis Distance p-value: 8.78e-007

Mahalanobis Distance: The Mahalanobis distance (MD) is a generalization of the Euclidean distance that accounts for correlations among the X properties. It is calculated as the distance to the center of the training data. The larger the MD, the less trustworthy the prediction.
Mahalanobis Distance p-value: The p-value gives the fraction of training data with an MD greater than or equal to the one for the given sample, assuming normally distributed data. The smaller the p-value, the less trustworthy the prediciton. For highly non-normal X properties (e.g., fingerprints), the MD p-value is wildly inaccurate.

Structural Similar Compounds

Name	SULFISOOXAZOLE	OCHRATOXIN	PROBENECID
Structure	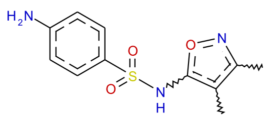	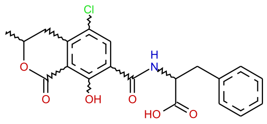	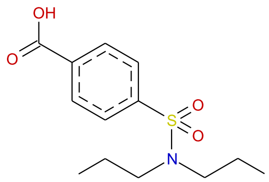
Actual Endpoint (-log C)	2.82494	6.28396	2.85333
Predicted Endpoint (-log C)	3.0705	5.12358	2.4258
Distance	0.903	0.938	0.990
Reference	NCI/NTP TR-138	NCI/NTP TR-358	NCI/NTP TR-395

Model Applicability

Unknown features are fingerprint features in the query molecule, but not found in the training set.

1. Num_AromaticRings out of range. Value: 3. Training min, max, mean, SD: 0, 2, 0.5625, 0.693.
2. OPS PC6 out of range. Value: -3.1049. Training min, max, SD, explained variance: -2.4321, 2.9885, 1.256, 0.0488.
3. Unknown FCFP_2 feature: 8: [*][N+](=[*])[*]
4. Unknown FCFP_2 feature: 5: [*][O-]
5. Unknown FCFP_2 feature: 907036844: [*]OC[c](:[*]):[*]
6. Unknown FCFP_2 feature: -828984032: [*][N+](=[*])[c](:[cH]:[*]):[cH]:[*]
7. Unknown FCFP_2 feature: -1338588315: [*]:[c](:[*])[N+](=O)[O-]
8. Unknown FCFP_2 feature: 1872392852: [*][N+](=O)[*]
9. Unknown FCFP_2 feature: 260476081: [*][N+](=[*])[O-]

Feature Contribution

Top features for positive contribution

Fingerprint	Bit/Smiles	Feature Structure	Score

FCFP_2	332760439	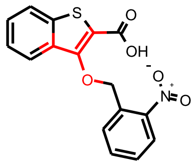<br>[*]O[c](:[cH]:[*]):[c H]:[*]	0.672
FCFP_2	1	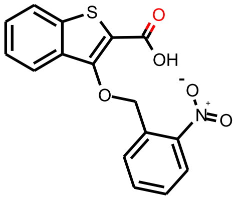<br>[*]=O	0.511
FCFP_2	7	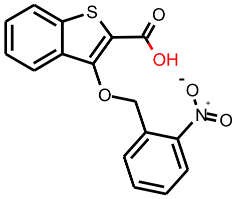<br>[*]O	0.014

Top Features for negative contribution

Fingerprint	Bit/Smiles	Feature Structure	Score
FCFP_2	203677720	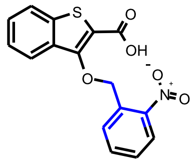<br>[*]C(=[*])[c]1:[c]([*]):[*]:[*]:[c]:1:[*]	-0.406
FCFP_2	1872154524	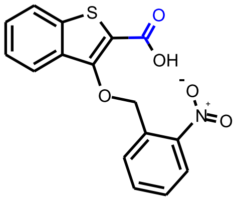<br>[*]C(=O)[*]	-0.307

FCFP_2	0	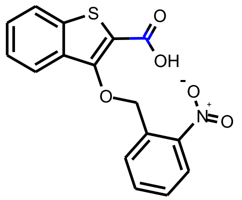 [*]C(=[*])[*]	-0.290

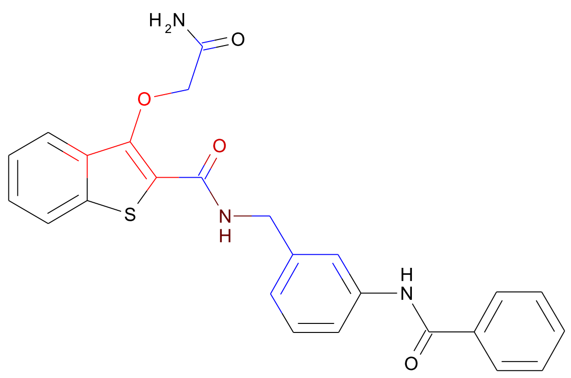

$C_{25}H_{21}N_3O_4S$
Molecular Weight: 459.51694
ALogP: 3.396
Rotatable Bonds: 8
Acceptors: 4
Donors: 3

Model Prediction

Prediction: 0.280
Unit: g/kg_body_weight
Mahalanobis Distance: 15.549
Mahalanobis Distance p-value: 9.19e-014

Mahalanobis Distance: The Mahalanobis distance (MD) is a generalization of the Euclidean distance that accounts for correlations among the X properties. It is calculated as the distance to the center of the training data. The larger the MD, the less trustworthy the prediction.
Mahalanobis Distance p-value: The p-value gives the fraction of training data with an MD greater than or equal to the one for the given sample, assuming normally distributed data. The smaller the p-value, the less trustworthy the prediciton. For highly non-normal X properties (e.g., fingerprints), the MD p-value is wildly inaccurate.

Structural Similar Compounds

Name	OCHRATOXIN	SULFISOOXAZOLE	PENICILLIN VK
Structure	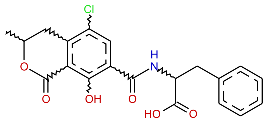	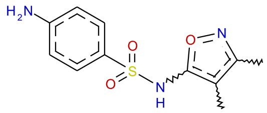	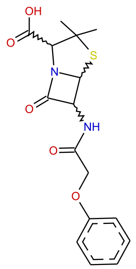
Actual Endpoint (-log C)	6.28396	2.82494	2.54455
Predicted Endpoint (-log C)	5.12358	3.0705	3.9702
Distance	1.005	1.132	1.272
Reference	NCI/NTP TR-358	NCI/NTP TR-138	NCI/NTP TR-336

Model Applicability

Unknown features are fingerprint features in the query molecule, but not found in the training set.

1. Molecular_Weight out of range. Value: 459.52. Training min, max, mean, SD: 68.074, 434.63, 171.13, 85.06.
2. Num_AromaticRings out of range. Value: 4. Training min, max, mean, SD: 0, 2, 0.5625, 0.693.
3. Molecular_PolarSASA out of range. Value: 228.72. Training min, max, mean, SD: 0, 223.97, 50.816, 55.15.
4. Molecular_PolarSurfaceArea out of range. Value: 138.76. Training min, max, mean, SD: 0, 138.03, 28.978, 32.1.
5. OPS PC6 out of range. Value: -3.4423. Training min, max, SD, explained variance: -2.4321, 2.9885, 1.256, 0.0488.

Feature Contribution

Top features for positive contribution			
Fingerprint	Bit/Smiles	Feature Structure	Score

FCFP_2	332760439	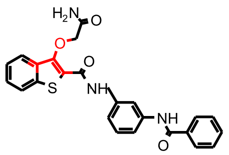 [*]O[c](:[cH]:[*]):[cH]:[*]	0.672
FCFP_2	1	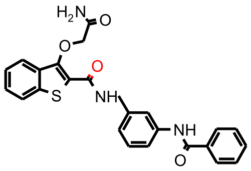 [*]=O	0.511
FCFP_2	3	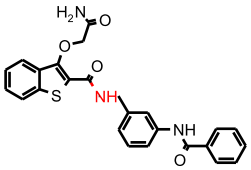 [*]N	0.104
**Top Features for negative contribution**			
**Fingerprint**	**Bit/Smiles**	**Feature Structure**	**Score**
FCFP_2	203677720	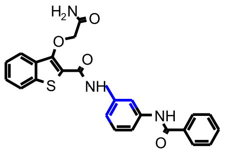 [*]C(=[*])[c]1:[c]([*]):[*]:[*]:[c]:1:[*]	-0.406
FCFP_2	1872154524	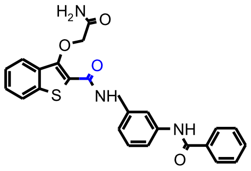 [*]C(=O)[*]	-0.307

FCFP_2	0	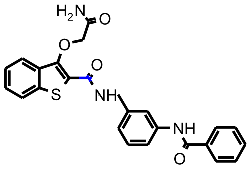<br>[*]C(=[*])[*]	-0.290

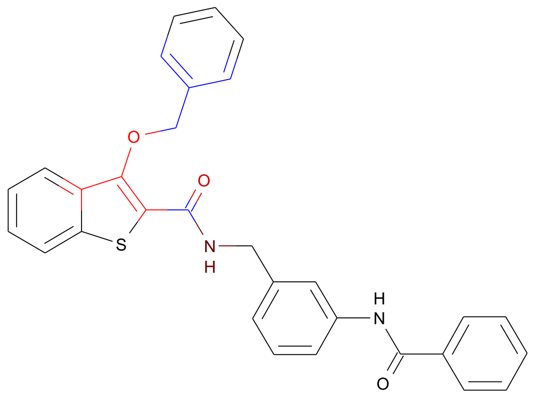

$C_{30}H_{24}N_2O_3S$
Molecular Weight: 492.58816
ALogP: 6.126
Rotatable Bonds: 8
Acceptors: 3
Donors: 2

Structural Similar Compounds

Name	OCHRATOXIN	SULFISOOXAZOLE	PHENYLBUTAZONE
Structure	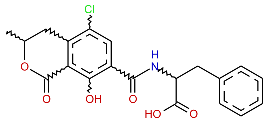	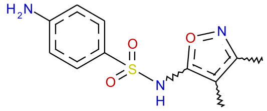	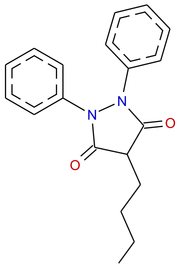
Actual Endpoint (-log C)	6.28396	2.82494	3.48909
Predicted Endpoint (-log C)	5.12358	3.0705	3.17333
Distance	1.334	1.403	1.476
Reference	NCI/NTP TR-358	NCI/NTP TR-138	NCI/NTP TR-367

Model Prediction

Prediction: 0.040
Unit: g/kg_body_weight
Mahalanobis Distance: 14.628
Mahalanobis Distance p-value: 1.71e-012

Mahalanobis Distance: The Mahalanobis distance (MD) is a generalization of the Euclidean distance that accounts for correlations among the X properties. It is calculated as the distance to the center of the training data. The larger the MD, the less trustworthy the prediction.
Mahalanobis Distance p-value: The p-value gives the fraction of training data with an MD greater than or equal to the one for the given sample, assuming normally distributed data. The smaller the p-value, the less trustworthy the prediciton. For highly non-normal X properties (e.g., fingerprints), the MD p-value is wildly inaccurate.

Model Applicability

Unknown features are fingerprint features in the query molecule, but not found in the training set.

1. Molecular_Weight out of range. Value: 492.59. Training min, max, mean, SD: 68.074, 434.63, 171.13, 85.06.
2. Num_AromaticRings out of range. Value: 5. Training min, max, mean, SD: 0, 2, 0.5625, 0.693.
3. OPS PC6 out of range. Value: -4.1688. Training min, max, SD, explained variance: -2.4321, 2.9885, 1.256, 0.0488.
4. Unknown FCFP_2 feature: 907036844: [*]OC[c](:[*]):[*]

Feature Contribution

Top features for positive contribution			
**Fingerprint**	**Bit/Smiles**	**Feature Structure**	**Score**
FCFP_2	332760439	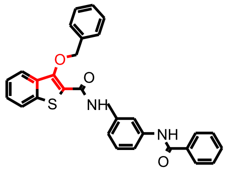 [*]O[c](:[cH]:[*]):[cH]:[*]	0.672

FCFP_2	1	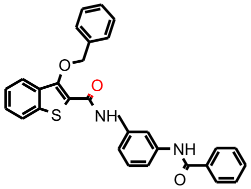 [*]=O	0.511
FCFP_2	3	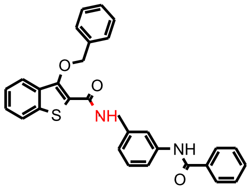 [*]N	0.104

Top Features for negative contribution

Fingerprint	Bit/Smiles	Feature Structure	Score
FCFP_2	203677720	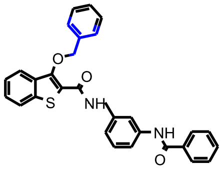 [*]C(=[*])[c]1:[c]([*]):[*]:[*]:[c]:1:[*]	-0.406
FCFP_2	1872154524	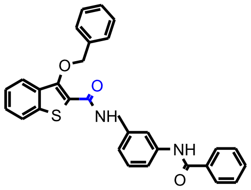 [*]C(=O)[*]	-0.307
FCFP_2	0	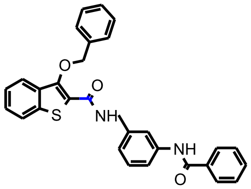 [*]C(=[*])[*]	-0.290

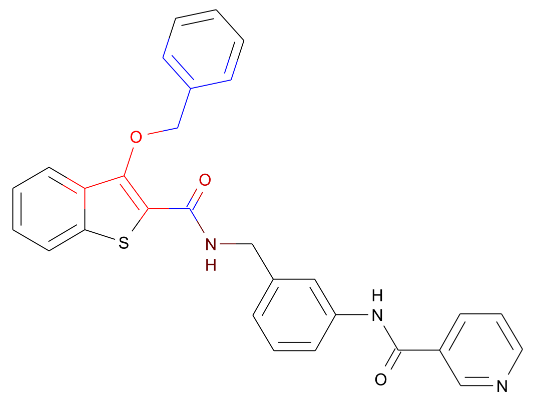

$C_{29}H_{23}N_3O_3S$
Molecular Weight: 493.57622
ALogP: 4.976
Rotatable Bonds: 8
Acceptors: 4
Donors: 2

Model Prediction

Prediction: 0.023
Unit: g/kg_body_weight
Mahalanobis Distance: 13.575
Mahalanobis Distance p-value: 5.13e-011

Mahalanobis Distance: The Mahalanobis distance (MD) is a generalization of the Euclidean distance that accounts for correlations among the X properties. It is calculated as the distance to the center of the training data. The larger the MD, the less trustworthy the prediction.
Mahalanobis Distance p-value: The p-value gives the fraction of training data with an MD greater than or equal to the one for the given sample, assuming normally distributed data. The smaller the p-value, the less trustworthy the prediciton. For highly non-normal X properties (e.g., fingerprints), the MD p-value is wildly inaccurate.

Structural Similar Compounds

Name	OCHRATOXIN	SULFISOOXAZOLE	8-METHOXYPSORALEN
Structure	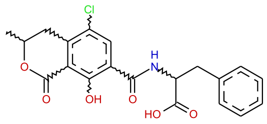	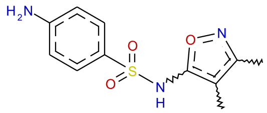	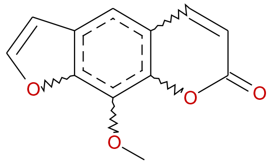
Actual Endpoint (-log C)	6.28396	2.82494	3.45978
Predicted Endpoint (-log C)	5.12358	3.0705	4.14745
Distance	1.264	1.344	1.508
Reference	NCI/NTP TR-358	NCI/NTP TR-138	NCI/NTP TR-359

Model Applicability

Unknown features are fingerprint features in the query molecule, but not found in the training set.

1. Molecular_Weight out of range. Value: 493.58. Training min, max, mean, SD: 68.074, 434.63, 171.13, 85.06.
2. Num_AromaticRings out of range. Value: 5. Training min, max, mean, SD: 0, 2, 0.5625, 0.693.
3. OPS PC6 out of range. Value: -4.0783. Training min, max, SD, explained variance: -2.4321, 2.9885, 1.256, 0.0488.
4. Unknown FCFP_2 feature: 907036844: [*]OC[c](:[*]):[*]

Feature Contribution

Top features for positive contribution			
Fingerprint	Bit/Smiles	Feature Structure	Score
FCFP_2	332760439	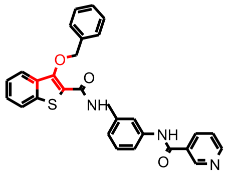 [*]O[c](:[cH]:[*]):[cH]:[*]	0.672

FCFP_2	1	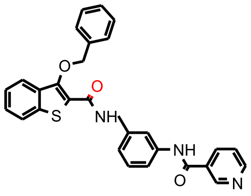<br>[*]=O	0.511
FCFP_2	3	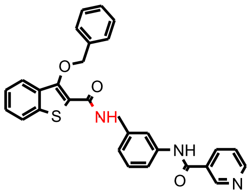<br>[*]N	0.104
**Top Features for negative contribution**			
**Fingerprint**	**Bit/Smiles**	**Feature Structure**	**Score**
FCFP_2	203677720	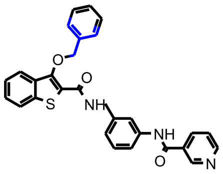<br>[*]C(=[*])[c]1:[c]([*<br>]):[*]:[*]:[c]:1:[*]	-0.406
FCFP_2	1872154524	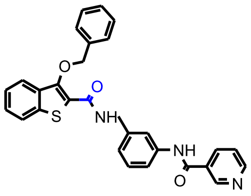<br>[*]C(=O)[*]	-0.307
FCFP_2	0	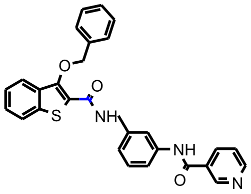<br>[*]C(=[*])[*]	-0.290

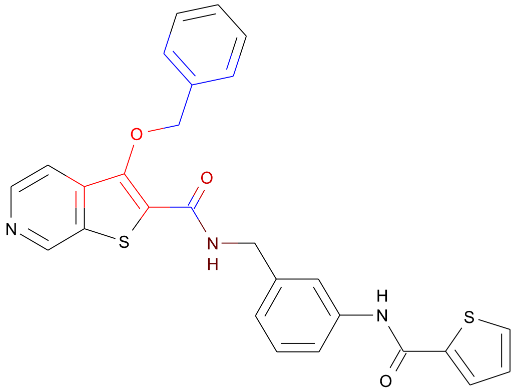

$C_{27}H_{21}N_3O_3S_2$
Molecular Weight: 499.60394
ALogP: 4.929
Rotatable Bonds: 8
Acceptors: 4
Donors: 2

Model Prediction

Prediction: 0.049
Unit: g/kg_body_weight
Mahalanobis Distance: 14.054
Mahalanobis Distance p-value: 1.08e-011

Mahalanobis Distance: The Mahalanobis distance (MD) is a generalization of the Euclidean distance that accounts for correlations among the X properties. It is calculated as the distance to the center of the training data. The larger the MD, the less trustworthy the prediction.
Mahalanobis Distance p-value: The p-value gives the fraction of training data with an MD greater than or equal to the one for the given sample, assuming normally distributed data. The smaller the p-value, the less trustworthy the prediciton. For highly non-normal X properties (e.g., fingerprints), the MD p-value is wildly inaccurate.

Structural Similar Compounds

Name	OCHRATOXIN	SULFISOOXAZOLE	PENICILLIN VK
Structure	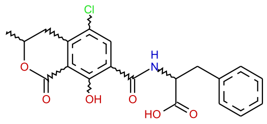	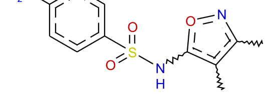	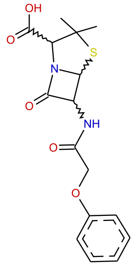
Actual Endpoint (-log C)	6.28396	2.82494	2.54455
Predicted Endpoint (-log C)	5.12358	3.0705	3.9702
Distance	1.273	1.362	1.567
Reference	NCI/NTP TR-358	NCI/NTP TR-138	NCI/NTP TR-336

Model Applicability

Unknown features are fingerprint features in the query molecule, but not found in the training set.

1. Molecular_Weight out of range. Value: 499.6. Training min, max, mean, SD: 68.074, 434.63, 171.13, 85.06.
2. Num_AromaticRings out of range. Value: 5. Training min, max, mean, SD: 0, 2, 0.5625, 0.693.
3. OPS PC6 out of range. Value: -4.0599. Training min, max, SD, explained variance: -2.4321, 2.9885, 1.256, 0.0488.
4. Unknown FCFP_2 feature: 907036844: [*]OC[c](:[*]):[*]

Feature Contribution

Top features for positive contribution

Fingerprint	Bit/Smiles	Feature Structure	Score
FCFP_2	332760439	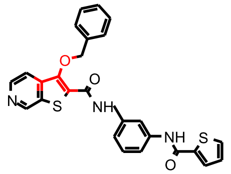 [*]O[c](:[cH]:[*]):[cH]:[*]	0.672

FCFP_2	1	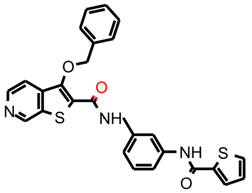 [*]=O	0.511
FCFP_2	3	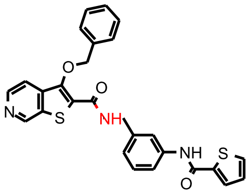 [*]N	0.104

Top Features for negative contribution

Fingerprint	Bit/Smiles	Feature Structure	Score
FCFP_2	203677720	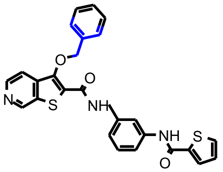 [*]C(=[*])[c]1:[c]([*]):[*]:[*]:[c]:1:[*]	-0.406
FCFP_2	1872154524	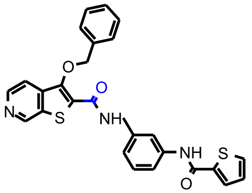 [*]C(=O)[*]	-0.307
FCFP_2	0	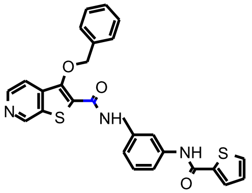 [*]C(=[*])[*]	-0.290

Co-crystallized ligand

TOPKAT_Rat_Maximum_Tolerated_Dose_Gavage

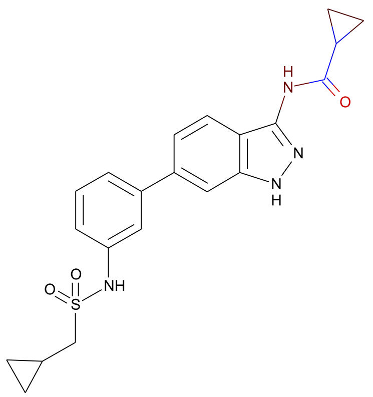

$C_{21}H_{22}N_4O_3S$
Molecular Weight: 410.48938
ALogP: 3.14
Rotatable Bonds: 7
Acceptors: 4
Donors: 3

Model Prediction

Prediction: 0.081
Unit: g/kg_body_weight
Mahalanobis Distance: 11.836
Mahalanobis Distance p-value: 1.51e-008

Mahalanobis Distance: The Mahalanobis distance (MD) is a generalization of the Euclidean distance that accounts for correlations among the X properties. It is calculated as the distance to the center of the training data. The larger the MD, the less trustworthy the prediction.
Mahalanobis Distance p-value: The p-value gives the fraction of training data with an MD greater than or equal to the one for the given sample, assuming normally distributed data. The smaller the p-value, the less trustworthy the prediciton. For highly non-normal X properties (e.g., fingerprints), the MD p-value is wildly inaccurate.

Structural Similar Compounds

Name	OCHRATOXIN	SULFISOOXAZOLE	PENICILLIN VK
Structure	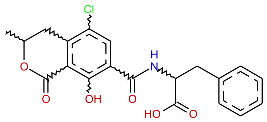	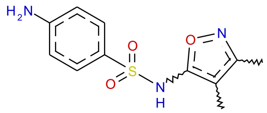	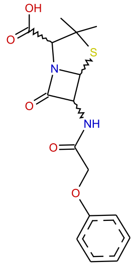
Actual Endpoint (-log C)	6.28396	2.82494	2.54455
Predicted Endpoint (-log C)	5.12358	3.0705	3.9702
Distance	0.781	0.878	1.069
Reference	NCI/NTP TR-358	NCI/NTP TR-138	NCI/NTP TR-336

Model Applicability

Unknown features are fingerprint features in the query molecule, but not found in the training set.

1. Num_AromaticRings out of range. Value: 3. Training min, max, mean, SD: 0, 2, 0.5625, 0.693.
2. Unknown FCFP_2 feature: 19: [*]:[nH]:[*]
3. Unknown FCFP_2 feature: 262592487: [*]:[c]1:[*]:[*]:n:[nH]:1
4. Unknown FCFP_2 feature: 1747267175: [*][c]1:[*]:[*]:[nH]:n:1
5. Unknown FCFP_2 feature: -1861645784: [*]:[cH]:[c](:[cH]:[*])[c](:[*]):[*]
6. Unknown FCFP_2 feature: 307448885: [*]:[c]1:[*]:[*]:[nH]:[c]:1:c:[*]
7. Unknown FCFP_2 feature: 1018942292: [*]CS(=O)(=O)N[*]

Feature Contribution

Top features for positive contribution			
Fingerprint	Bit/Smiles	Feature Structure	Score
FCFP_2	1	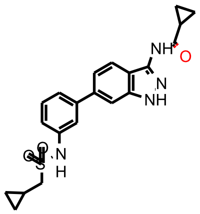 [*]=O	0.511

FCFP_2	3	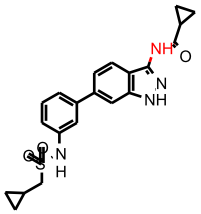 [*]N	0.104
FCFP_2	-1272798659	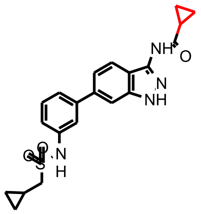 [*]CCC[*]	0.070
**Top Features for negative contribution**			
**Fingerprint**	**Bit/Smiles**	**Feature Structure**	**Score**
FCFP_2	1872154524	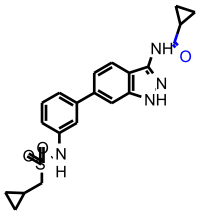 [*]C(=O)[*]	-0.307
FCFP_2	0	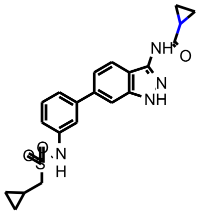 [*]C(=[*])[*]	-0.290

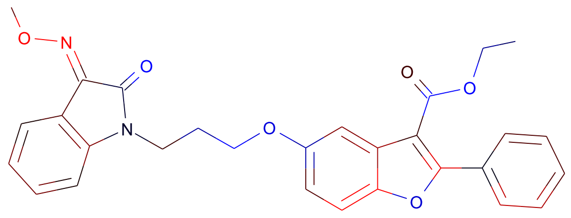

$C_{29}H_{26}N_2O_6$

Molecular Weight: 498.52653

ALogP: 4.994

Rotatable Bonds: 10

Acceptors: 6

Donors: 0

Model Prediction

Prediction: 2.223

Unit: g/kg_body_weight

Mahalanobis Distance: 28.272

Mahalanobis Distance p-value: 1.03e-051

Mahalanobis Distance: The Mahalanobis distance (MD) is a generalization of the Euclidean distance that accounts for correlations among the X properties. It is calculated as the distance to the center of the training data. The larger the MD, the less trustworthy the prediction.
Mahalanobis Distance p-value: The p-value gives the fraction of training data with an MD greater than or equal to the one for the given sample, assuming normally distributed data. The smaller the p-value, the less trustworthy the prediciton. For highly non-normal X properties (e.g., fingerprints), the MD p-value is wildly inaccurate.

Structural Similar Compounds

Name	2-BENZIMIDAZOLINONE; 1-[1-(3-CYANO-3;3-DIPHENYLPROPYL)-4-PIPERIDYL]-3-PROPIONYL-	KETOCONAZOLE	1-ACETYL-3;3-bis-[4-(ACETYLOXY)PHENYL]-1;3-DIHYDRO-2H-INDOL-2-ONE
Structure	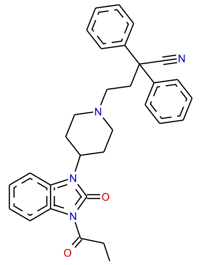	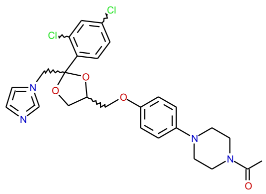	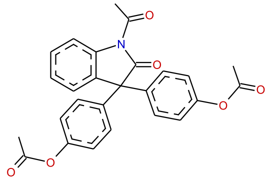
Actual Endpoint (-log C)	3.543	3.505	2.948
Predicted Endpoint (-log C)	3.00532	2.65464	2.6866
Distance	0.688	0.688	0.711
Reference	ARZNAD 21;862;71	MDACAP 17;373;81	JAPMA8 42;468;53

Model Applicability

Unknown features are fingerprint features in the query molecule, but not found in the training set.

1. All properties and OPS components are within expected ranges.
2. Unknown ECFP_2 feature: 1716966732: [*]\N=C/1\C(=[*])[*][*]:[c]1:[*]
3. Unknown ECFP_2 feature: -408704017: [*]=NOC
4. Unknown FCFP_6 feature: 16: [*][c](:[*]):[*]
5. Unknown FCFP_6 feature: 1747237384: [*][c]1:[*]:[*]:[c](:[*]):o:1
6. Unknown FCFP_6 feature: 690511177: [*][c]1:[*]:[*]:o:[c]:1[c](:[*]):[*]
7. Unknown FCFP_6 feature: 1618154665: [*][c](:[*]):[cH]:[c](:[*]):[*]
8. Unknown FCFP_6 feature: -1861645784: [*]:[cH]:[c](:[cH]:[*])[c](:[*]):[*]
9. Unknown FCFP_6 feature: 580960234: [*]ON=C([*])[*]

Feature Contribution

Top features for positive contribution			
Fingerprint	Bit/Smiles	Feature Structure	Score

ECFP_6	642810091	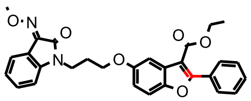 [*][c](:[*]):[*]	0.281
FCFP_6	-1143686149	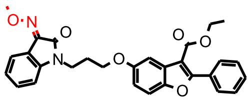 [*]=NOC	0.243
ECFP_6	1571214559	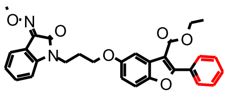 [*]1:[cH]:[cH]:[cH]:[cH]:[cH]:1	0.190

Top Features for negative contribution

Fingerprint	Bit/Smiles	Feature Structure	Score
ECFP_6	2106656448	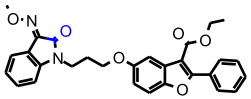 [*]C(=O)[*]	-0.352
ECFP_6	1887306650	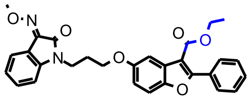 [*]C(=[*])OCC	-0.271

ECFP_6	683445015	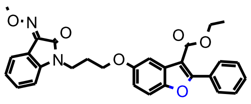<br>[*]:o:[*]	-0.266

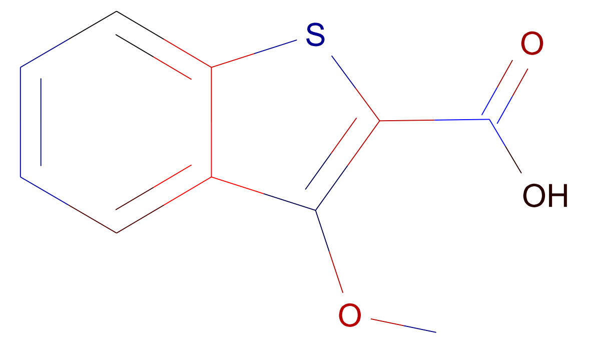

$C_{10}H_8O_3S$

Molecular Weight: 208.23372

ALogP: 2.596

Rotatable Bonds: 2

Acceptors: 3

Donors: 1

Model Prediction

Prediction: 0.240

Unit: g/kg_body_weight

Mahalanobis Distance: 16.045

Mahalanobis Distance p-value: 0.273

Mahalanobis Distance: The Mahalanobis distance (MD) is a generalization of the Euclidean distance that accounts for correlations among the X properties. It is calculated as the distance to the center of the training data. The larger the MD, the less trustworthy the prediction.
Mahalanobis Distance p-value: The p-value gives the fraction of training data with an MD greater than or equal to the one for the given sample, assuming normally distributed data. The smaller the p-value, the less trustworthy the prediciton. For highly non-normal X properties (e.g., fingerprints), the MD p-value is wildly inaccurate.

Structural Similar Compounds

Name	N-METHYLCARBAMIC ACID; 4-BENZOTHIENYL ESTER	BENZIMIDAZOLE; 2-(TRIFLUORO)METHYL-4-NITRO-	BENZIMIDAZOLE; 2-(TRIFLUORO)METHYL-5-NITRO-
Structure	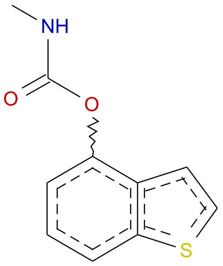	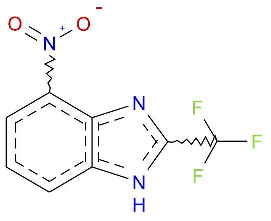	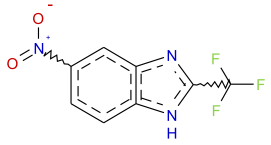
Actual Endpoint (-log C)	3.471	4.34	4.8
Predicted Endpoint (-log C)	4.24261	3.69685	3.84207
Distance	0.438	0.472	0.480
Reference	TXAPA9 11;546;67	PSSCBG 15;31;84	PSSCBG 15;31;84

Model Applicability

Unknown features are fingerprint features in the query molecule, but not found in the training set.

1. All properties and OPS components are within expected ranges.
2. Unknown ECFP_2 feature: -1670580914: [*]C(=[*])[c]1:s:[*]:[*]:[c]:1[*]
3. Unknown FCFP_6 feature: 16: [*][c](:[*]):[*]
4. Unknown FCFP_6 feature: 1747237384: [*][c]1:[*]:[*]:[c](:[*]):o:1
5. Unknown FCFP_6 feature: 1618154665: [*][c](:[*]):[cH]:[c](:[*]):[*]
6. Unknown FCFP_6 feature: -1549222613: [*]:[c](:[*])C(=O)O

Feature Contribution

Top features for positive contribution			
Fingerprint	Bit/Smiles	Feature Structure	Score
ECFP_6	642810091	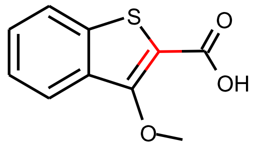 [*][c](:[*]):[*]	0.281

FCFP_6	136627117	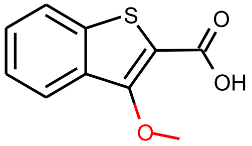 [*]OC	0.170
ECFP_6	-1074141656	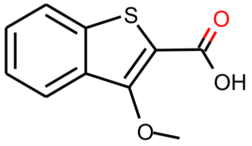 [*]=O	0.142
**Top Features for negative contribution**			
**Fingerprint**	**Bit/Smiles**	**Feature Structure**	**Score**
ECFP_6	734603939	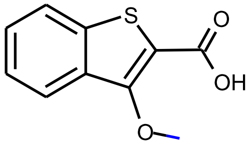 [*]C	-0.201
FCFP_6	-1539132615	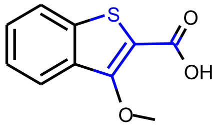 [*]C(=[*])[c]1:s:[*]:[*]:[c]:1[*]	-0.200
FCFP_6	1036089772	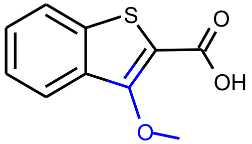 [*]CO[c](:[*]):[*]	-0.136

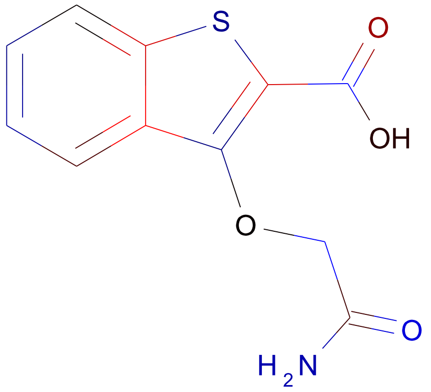

$C_{11}H_9NO_4S$
Molecular Weight: 251.25846
ALogP: 1.45
Rotatable Bonds: 4
Acceptors: 4
Donors: 2

Structural Similar Compounds

Name	SULFAFURAZOLE	2-NAPHTHALENESULFONIC ACID; 5-AMINO-6-ETHOXY-	SULFAMETHOXAZOLE
Structure	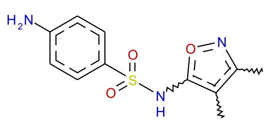	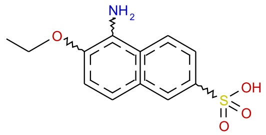	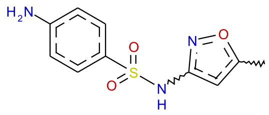
Actual Endpoint (-log C)	1.427	1.348	1.611
Predicted Endpoint (-log C)	1.37011	1.84442	1.72769
Distance	0.537	0.546	0.550
Reference	NIIRDN 6;391;82	85JCAE -;1061;86	TXAPA9 18;185;71

Model Prediction

Prediction: 0.230
Unit: g/kg_body_weight
Mahalanobis Distance: 19.767
Mahalanobis Distance p-value: 3.26e-008

Mahalanobis Distance: The Mahalanobis distance (MD) is a generalization of the Euclidean distance that accounts for correlations among the X properties. It is calculated as the distance to the center of the training data. The larger the MD, the less trustworthy the prediction.
Mahalanobis Distance p-value: The p-value gives the fraction of training data with an MD greater than or equal to the one for the given sample, assuming normally distributed data. The smaller the p-value, the less trustworthy the prediciton. For highly non-normal X properties (e.g., fingerprints), the MD p-value is wildly inaccurate.

Model Applicability

Unknown features are fingerprint features in the query molecule, but not found in the training set.

1. All properties and OPS components are within expected ranges.
2. Unknown ECFP_2 feature: -1670580914: [*]C(=[*])[c]1:s:[*]:[*]:[c]:1[*]
3. Unknown FCFP_6 feature: 16: [*][c](:[*]):[*]
4. Unknown FCFP_6 feature: 1747237384: [*][c]1:[*]:[*]:[c](:[*]):o:1
5. Unknown FCFP_6 feature: 1618154665: [*][c](:[*]):[cH]:[c](:[*]):[*]
6. Unknown FCFP_6 feature: -1549222613: [*]:[c](:[*])C(=O)O

Feature Contribution

Top features for positive contribution			
Fingerprint	Bit/Smiles	Feature Structure	Score

ECFP_6	642810091	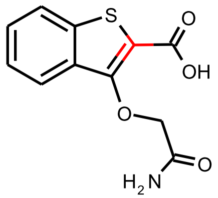 [*][c](:[*]):[*]	0.281
ECFP_6	-1074141656	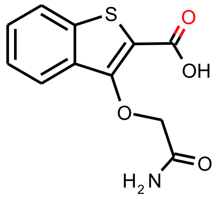 [*]=O	0.142
ECFP_6	-932108170	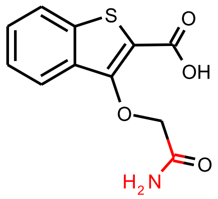 [*]C(=[*])N	0.126

Top Features for negative contribution

Fingerprint	Bit/Smiles	Feature Structure	Score
FCFP_6	566058135	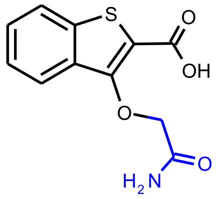 [*]CC(=O)N	-0.216
FCFP_6	-1539132615	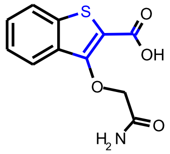 [*]C(=[*])[c]1:s:[*]:[*]:[c]:1[*]	-0.200

FCFP_6	1036089772	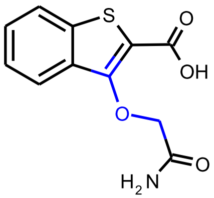 [*]CO[c](:[*]):[*]	-0.136

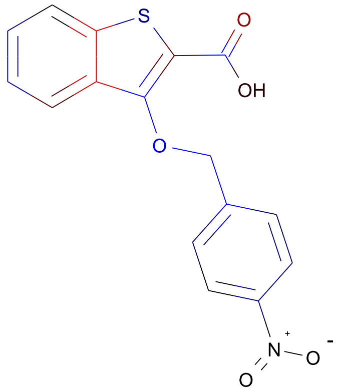

$C_{16}H_{11}NO_5S$
Molecular Weight: 329.32724
ALogP: 4.074
Rotatable Bonds: 5
Acceptors: 5
Donors: 1

Structural Similar Compounds

Name	ACENOCOUMARIN	BENZIMIDAZOLE; 2-(TRIFLUORO)METHYL-4;6-DICHLORO-5;7-DINITRO-	ACEMETACIN
Structure	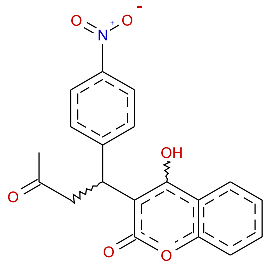	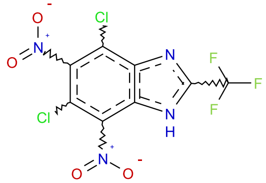	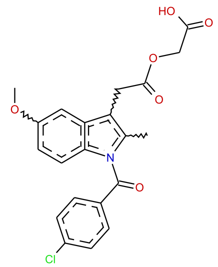
Actual Endpoint (-log C)	2.838	3.47	4.235
Predicted Endpoint (-log C)	3.3152	3.99964	3.39415
Distance	0.634	0.644	0.654
Reference	29ZVAB -;3;69	PSSCBG 15;31;84	ARZNAD 30;1398;80

Model Prediction

Prediction: 1.066
Unit: g/kg_body_weight
Mahalanobis Distance: 18.931
Mahalanobis Distance p-value: 6.47e-006

Mahalanobis Distance: The Mahalanobis distance (MD) is a generalization of the Euclidean distance that accounts for correlations among the X properties. It is calculated as the distance to the center of the training data. The larger the MD, the less trustworthy the prediction.
Mahalanobis Distance p-value: The p-value gives the fraction of training data with an MD greater than or equal to the one for the given sample, assuming normally distributed data. The smaller the p-value, the less trustworthy the prediciton. For highly non-normal X properties (e.g., fingerprints), the MD p-value is wildly inaccurate.

Model Applicability

Unknown features are fingerprint features in the query molecule, but not found in the training set.

1. All properties and OPS components are within expected ranges.
2. Unknown ECFP_2 feature: 1043790491: [*][N+](=[*])[*]
3. Unknown ECFP_2 feature: 781519895: [*][O-]
4. Unknown ECFP_2 feature: -1670580914: [*]C(=[*])[c]1:s:[*]:[*]:[c]:1[*]
5. Unknown ECFP_2 feature: -179073144: [*][N+](=[*])[c](:[cH]:[*]):[cH]:[*]
6. Unknown ECFP_2 feature: -215026467: [*]:[c](:[*])[N+](=O)[O-]
7. Unknown ECFP_2 feature: 2104376220: [*][N+](=O)[*]
8. Unknown ECFP_2 feature: -659271057: [*][N+](=[*])[O-]
9. Unknown FCFP_6 feature: 16: [*][c](:[*]):[*]
10. Unknown FCFP_6 feature: 8: [*][N+](=[*])[*]
11. Unknown FCFP_6 feature: 5: [*][O-]
12. Unknown FCFP_6 feature: 1747237384: [*][c]1:[*]:[*]:[c](:[*]):o:1
13. Unknown FCFP_6 feature: 1618154665: [*][c](:[*]):[cH]:[c](:[*]):[*]
14. Unknown FCFP_6 feature: -1549222613: [*]:[c](:[*])C(=O)O
15. Unknown FCFP_6 feature: -828984032: [*][N+](=[*])[c](:[cH]:[*]):[cH]:[*]
16. Unknown FCFP_6 feature: -1338588315: [*]:[c](:[*])[N+](=O)[O-]
17. Unknown FCFP_6 feature: 1872392852: [*][N+](=O)[*]
18. Unknown FCFP_6 feature: 260476081: [*][N+](=[*])[O-]

Feature Contribution

Top features for positive contribution

Fingerprint	Bit/Smiles	Feature Structure	Score
ECFP_6	642810091	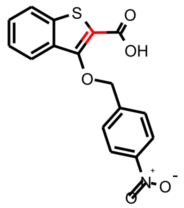 [*][c](:[*]):[*]	0.281
ECFP_6	-1074141656	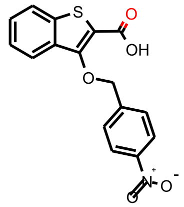 [*]=O	0.142
ECFP_6	1333660716	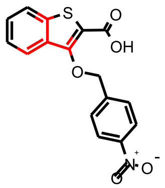 [*][c]1:[*]:[*]:[c](:[*]):[c]:1:[cH]:[*]	0.115

Top Features for negative contribution

Fingerprint	Bit/Smiles	Feature Structure	Score
FCFP_6	907036844	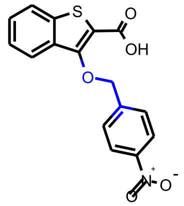 [*]OC[c](:[*]):[*]	-0.222

FCFP_6	-1539132615	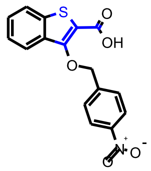<br>[*]C(=[*])[c]1:s:[*]:[*]:[c]:1[*]	-0.200
FCFP_6	1036089772	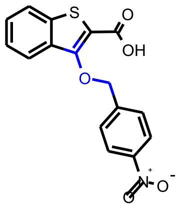<br>[*]CO[c](:[*]):[*]	-0.136

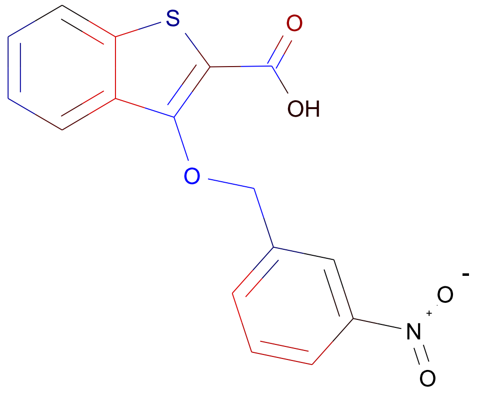

$C_{16}H_{11}NO_5S$
Molecular Weight: 329.32724
ALogP: 4.074
Rotatable Bonds: 5
Acceptors: 5
Donors: 1

Structural Similar Compounds

Name	ACENOCOUMARIN	BENZIMIDAZOLE; 2-(TRIFLUORO)METHYL-4;6-DICHLORO-5;7-DINITRO-	ACEMETACIN
Structure	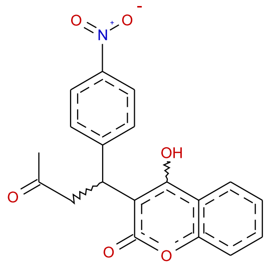	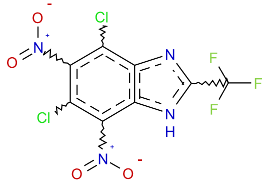	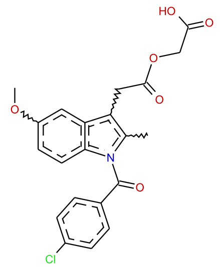
Actual Endpoint (-log C)	2.838	3.47	4.235
Predicted Endpoint (-log C)	3.3152	3.99964	3.39415
Distance	0.635	0.644	0.654
Reference	29ZVAB -;3;69	PSSCBG 15;31;84	ARZNAD 30;1398;80

Model Prediction

Prediction: 0.644
Unit: g/kg_body_weight
Mahalanobis Distance: 18.931
Mahalanobis Distance p-value: 6.47e-006

Mahalanobis Distance: The Mahalanobis distance (MD) is a generalization of the Euclidean distance that accounts for correlations among the X properties. It is calculated as the distance to the center of the training data. The larger the MD, the less trustworthy the prediction.
Mahalanobis Distance p-value: The p-value gives the fraction of training data with an MD greater than or equal to the one for the given sample, assuming normally distributed data. The smaller the p-value, the less trustworthy the prediciton. For highly non-normal X properties (e.g., fingerprints), the MD p-value is wildly inaccurate.

Model Applicability

Unknown features are fingerprint features in the query molecule, but not found in the training set.

1. All properties and OPS components are within expected ranges.
2. Unknown ECFP_2 feature: 1043790491: [*][N+](=[*])[*]
3. Unknown ECFP_2 feature: 781519895: [*][O-]
4. Unknown ECFP_2 feature: -1670580914: [*]C(=[*])[c]1:s:[*]:[*]:[c]:1[*]
5. Unknown ECFP_2 feature: -179073144: [*][N+](=[*])[c](:[cH]:[*]):[cH]:[*]
6. Unknown ECFP_2 feature: -215026467: [*]:[c](:[*])[N+](=O)[O-]
7. Unknown ECFP_2 feature: 2104376220: [*][N+](=O)[*]
8. Unknown ECFP_2 feature: -659271057: [*][N+](=[*])[O-]
9. Unknown FCFP_6 feature: 16: [*][c](:[*]):[*]
10. Unknown FCFP_6 feature: 8: [*][N+](=[*])[*]
11. Unknown FCFP_6 feature: 5: [*][O-]
12. Unknown FCFP_6 feature: 1747237384: [*][c]1:[*]:[*]:[c](:[*]):o:1
13. Unknown FCFP_6 feature: 1618154665: [*][c](:[*]):[cH]:[c](:[*]):[*]
14. Unknown FCFP_6 feature: -1549222613: [*]:[c](:[*])C(=O)O
15. Unknown FCFP_6 feature: -828984032: [*][N+](=[*])[c](:[cH]:[*]):[cH]:[*]
16. Unknown FCFP_6 feature: -1338588315: [*]:[c](:[*])[N+](=O)[O-]
17. Unknown FCFP_6 feature: 1872392852: [*][N+](=O)[*]
18. Unknown FCFP_6 feature: 260476081: [*][N+](=[*])[O-]

Feature Contribution

Top features for positive contribution

Fingerprint	Bit/Smiles	Feature Structure	Score
ECFP_6	642810091	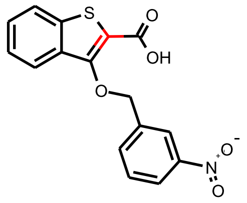 [*][c](:[*]):[*]	0.281
ECFP_6	-1074141656	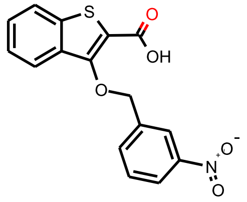 [*]=O	0.142
ECFP_6	2007300961	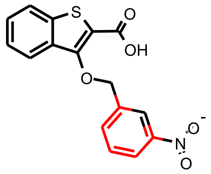 [*][c]1:[*]:[c]([*]):[cH]:[cH]:[cH]:1	0.123

Top Features for negative contribution

Fingerprint	Bit/Smiles	Feature Structure	Score
FCFP_6	907036844	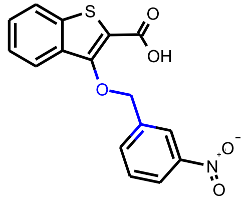 [*]OC[c](:[*]):[*]	-0.222

FCFP_6	-1539132615	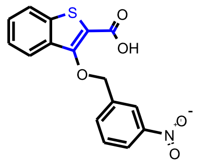 [*]C(=[*])[c]1:s:[*]:[*]:[c]:1[*]	-0.200
FCFP_6	1036089772	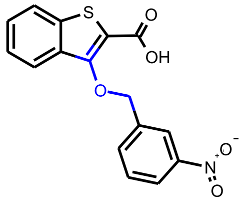 [*]CO[c](:[*]):[*]	-0.136

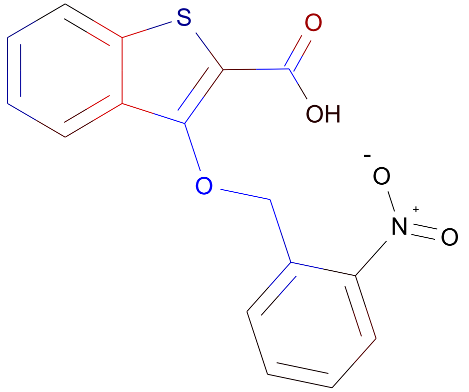

$C_{16}H_{11}NO_5S$
Molecular Weight: 329.32724
ALogP: 4.074
Rotatable Bonds: 5
Acceptors: 5
Donors: 1

Structural Similar Compounds

Name	ACENOCOUMARIN	BENZIMIDAZOLE; 2-(TRIFLUORO)METHYL-4;6-DICHLORO-5;7-DINITRO-	ACEMETACIN
Structure	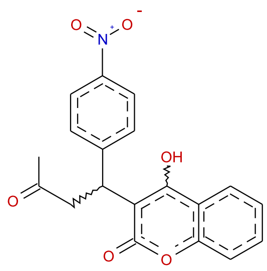	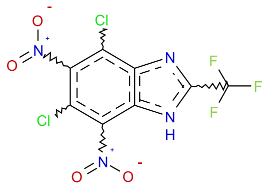	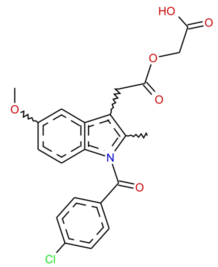
Actual Endpoint (-log C)	2.838	3.47	4.235
Predicted Endpoint (-log C)	3.3152	3.99964	3.39415
Distance	0.634	0.642	0.656
Reference	29ZVAB -;3;69	PSSCBG 15;31;84	ARZNAD 30;1398;80

Model Prediction

Prediction: 1.109
Unit: g/kg_body_weight
Mahalanobis Distance: 19.126
Mahalanobis Distance p-value: 2.05e-006

Mahalanobis Distance: The Mahalanobis distance (MD) is a generalization of the Euclidean distance that accounts for correlations among the X properties. It is calculated as the distance to the center of the training data. The larger the MD, the less trustworthy the prediction.
Mahalanobis Distance p-value: The p-value gives the fraction of training data with an MD greater than or equal to the one for the given sample, assuming normally distributed data. The smaller the p-value, the less trustworthy the prediciton. For highly non-normal X properties (e.g., fingerprints), the MD p-value is wildly inaccurate.

Model Applicability

Unknown features are fingerprint features in the query molecule, but not found in the training set.

1. All properties and OPS components are within expected ranges.
2. Unknown ECFP_2 feature: 1043790491: [*][N+](=[*])[*]
3. Unknown ECFP_2 feature: 781519895: [*][O-]
4. Unknown ECFP_2 feature: -1670580914: [*]C(=[*])[c]1:s:[*]:[*]:[c]:1[*]
5. Unknown ECFP_2 feature: -1956535100: [*][c](:[*]):[c](:[cH]:[*])[N+](=[*])[*]
6. Unknown ECFP_2 feature: -215026467: [*]:[c](:[*])[N+](=O)[O-]
7. Unknown ECFP_2 feature: 2104376220: [*][N+](=O)[*]
8. Unknown ECFP_2 feature: -659271057: [*][N+](=[*])[O-]
9. Unknown FCFP_6 feature: 16: [*][c](:[*]):[*]
10. Unknown FCFP_6 feature: 8: [*][N+](=[*])[*]
11. Unknown FCFP_6 feature: 5: [*][O-]
12. Unknown FCFP_6 feature: 1747237384: [*][c]1:[*]:[*]:[c](:[*]):o:1
13. Unknown FCFP_6 feature: 1618154665: [*][c](:[*]):[cH]:[c](:[*]):[*]
14. Unknown FCFP_6 feature: -1549222613: [*]:[c](:[*])C(=O)O
15. Unknown FCFP_6 feature: -828984032: [*][N+](=[*])[c](:[cH]:[*]):[cH]:[*]
16. Unknown FCFP_6 feature: -1338588315: [*]:[c](:[*])[N+](=O)[O-]
17. Unknown FCFP_6 feature: 1872392852: [*][N+](=O)[*]
18. Unknown FCFP_6 feature: 260476081: [*][N+](=[*])[O-]

Feature Contribution

Top features for positive contribution			
**Fingerprint**	**Bit/Smiles**	**Feature Structure**	**Score**
ECFP_6	642810091	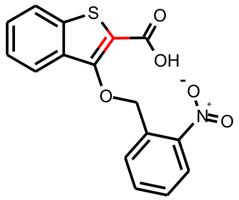 [*][c](:[*]):[*]	0.281
ECFP_6	-1074141656	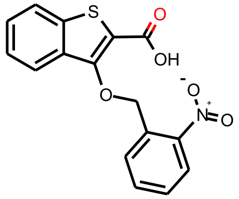 [*]=O	0.142
ECFP_6	1333660716	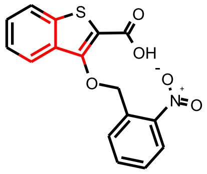 [*][c]1:[*]:[*]:[c](:[*]):[c]:1:[cH]:[*]	0.115

Top Features for negative contribution			
**Fingerprint**	**Bit/Smiles**	**Feature Structure**	**Score**
FCFP_6	907036844	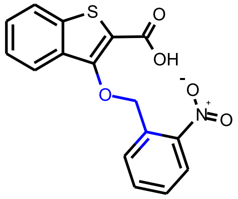 [*]OC[c](:[*]):[*]	-0.222

FCFP_6	-1539132615	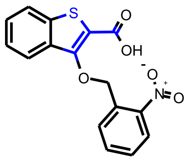 [*]C(=[*])[c]1:s:[*]:[*]:[c]:1[*]	-0.200
FCFP_6	1036089772	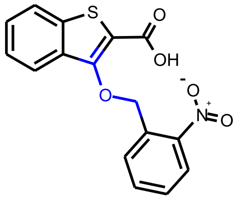 [*]CO[c](:[*]):[*]	-0.136

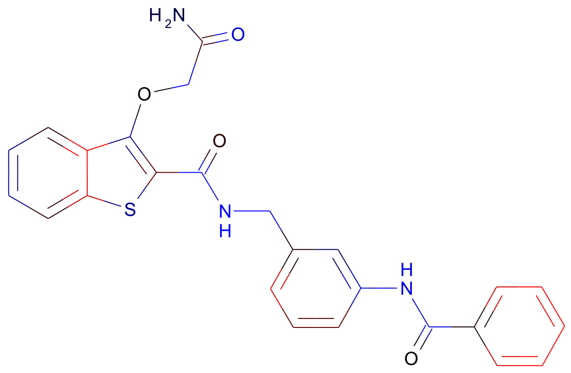

$C_{25}H_{21}N_3O_4S$

Molecular Weight: 459.51694

ALogP: 3.396

Rotatable Bonds: 8

Acceptors: 4

Donors: 3

Structural Similar Compounds

Name	BENZENESULFONIC ACID; 2;2'-(4;4'-BIPHENYLYLENEDIVINYLENE)DI-; DISODIUM SALT (Na STRIPPED)	AZOSEMIDE	NAFAMSTAT; MESYLATE SALT (MESYLATE STRIPPED)
Structure	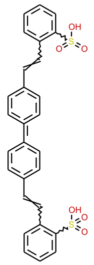	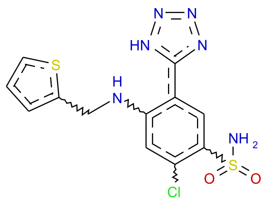	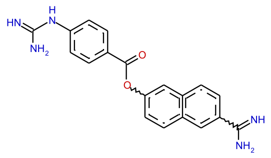
Actual Endpoint (-log C)	1.968	2.163	2.057
Predicted Endpoint (-log C)	1.72109	2.21052	2.61968
Distance	0.820	0.842	0.881
Reference	MVCRB3 2;193;73	IYKEDH 18;666;87	IYKEDH 17;1106;86

Model Prediction

Prediction: 2.364

Unit: g/kg_body_weight

Mahalanobis Distance: 23.783

Mahalanobis Distance p-value: 1.38e-024

Mahalanobis Distance: The Mahalanobis distance (MD) is a generalization of the Euclidean distance that accounts for correlations among the X properties. It is calculated as the distance to the center of the training data. The larger the MD, the less trustworthy the prediction.
Mahalanobis Distance p-value: The p-value gives the fraction of training data with an MD greater than or equal to the one for the given sample, assuming normally distributed data. The smaller the p-value, the less trustworthy the prediciton. For highly non-normal X properties (e.g., fingerprints), the MD p-value is wildly inaccurate.

Model Applicability

Unknown features are fingerprint features in the query molecule, but not found in the training set.

1. All properties and OPS components are within expected ranges.
2. Unknown ECFP_2 feature: -1670580914: [*]C(=[*])[c]1:s:[*]:[*]:[c]:1[*]
3. Unknown FCFP_6 feature: 16: [*][c](:[*]):[*]
4. Unknown FCFP_6 feature: 1747237384: [*][c]1:[*]:[*]:[c](:[*]):o:1
5. Unknown FCFP_6 feature: 1618154665: [*][c](:[*]):[cH]:[c](:[*]):[*]
6. Unknown FCFP_6 feature: 907096426: [*]NC[c](:[*]):[*]

Feature Contribution

Top features for positive contribution			
**Fingerprint**	**Bit/Smiles**	**Feature Structure**	**Score**

ECFP_6	642810091	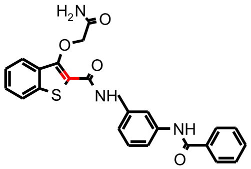 [*][c](:[*]):[*]	0.281
ECFP_6	-1897341097	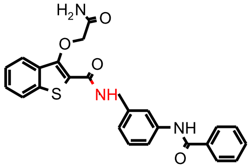 [*]N[*]	0.216
ECFP_6	1571214559	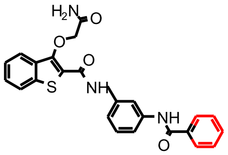 [*]1:[cH]:[cH]:[cH]:[cH]:[cH]:1	0.190
**Top Features for negative contribution**			
**Fingerprint**	**Bit/Smiles**	**Feature Structure**	**Score**
ECFP_6	497523368	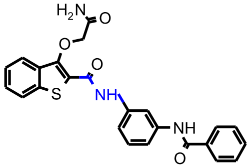 [*]CNC(=[*])[*]	-0.301
FCFP_6	566058135	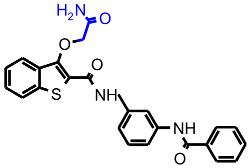 [*]CC(=O)N	-0.216

FCFP_6	-1539132615	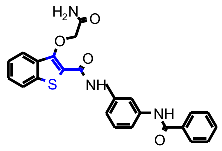 [*]C(=[*])[c]1:s:[*]:[*]:[c]:1[*]	-0.200

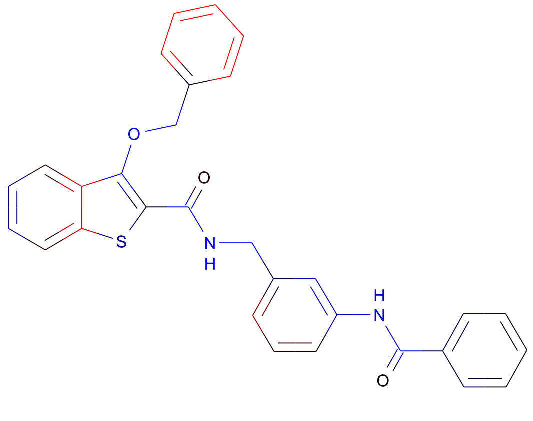

$C_{30}H_{24}N_2O_3S$
Molecular Weight: 492.58816
ALogP: 6.126
Rotatable Bonds: 8
Acceptors: 3
Donors: 2

Model Prediction

Prediction: 5.598
Unit: g/kg_body_weight
Mahalanobis Distance: 25.252
Mahalanobis Distance p-value: 1.53e-032

Mahalanobis Distance: The Mahalanobis distance (MD) is a generalization of the Euclidean distance that accounts for correlations among the X properties. It is calculated as the distance to the center of the training data. The larger the MD, the less trustworthy the prediction.
Mahalanobis Distance p-value: The p-value gives the fraction of training data with an MD greater than or equal to the one for the given sample, assuming normally distributed data. The smaller the p-value, the less trustworthy the prediciton. For highly non-normal X properties (e.g., fingerprints), the MD p-value is wildly inaccurate.

Structural Similar Compounds

Name	ANTHRAQUINONE; 1;4-bis-(p-TOLYLAMINO)-	FENDOSAL	BENZENESULFONIC ACID; 2;2'-(4;4'-BIPHENYLYLENEDIVINYLENE)DI-; DISODIUM SALT (Na STRIPPED)
Structure	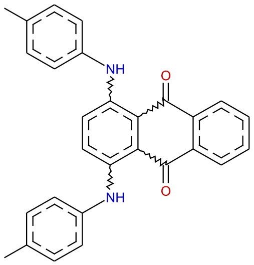	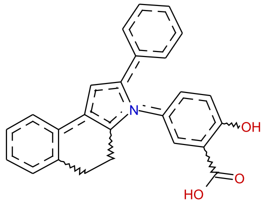	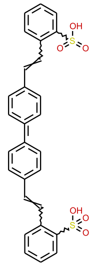
Actual Endpoint (-log C)	2.058	2.928	1.968
Predicted Endpoint (-log C)	1.57464	2.59	1.72109
Distance	0.801	0.817	0.874
Reference	85JCAE -;1330;86	AGACBH 8;209;78	MVCRB3 2;193;73

Model Applicability

Unknown features are fingerprint features in the query molecule, but not found in the training set.

1. All properties and OPS components are within expected ranges.
2. Unknown ECFP_2 feature: -1670580914: [*]C(=[*])[c]1:s:[*]:[*]:[c]:1[*]
3. Unknown FCFP_6 feature: 16: [*][c](:[*]):[*]
4. Unknown FCFP_6 feature: 1747237384: [*][c]1:[*]:[*]:[c](:[*]):o:1
5. Unknown FCFP_6 feature: 1618154665: [*][c](:[*]):[cH]:[c](:[*]):[*]
6. Unknown FCFP_6 feature: 907096426: [*]NC[c](:[*]):[*]

Feature Contribution

Top features for positive contribution			
**Fingerprint**	**Bit/Smiles**	**Feature Structure**	**Score**

ECFP_6	642810091	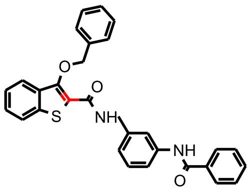 [*][c](:[*]):[*]	0.281
ECFP_6	-1897341097	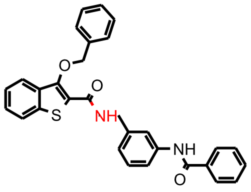 [*]N[*]	0.216
ECFP_6	1571214559	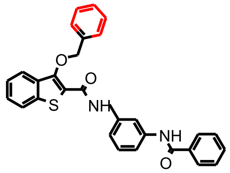 [*]1:[cH]:[cH]:[cH]:[cH]:[cH]:1	0.190
**Top Features for negative contribution**			
**Fingerprint**	**Bit/Smiles**	**Feature Structure**	**Score**
ECFP_6	497523368	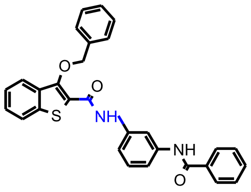 [*]CNC(=[*])[*]	-0.301
FCFP_6	907036844	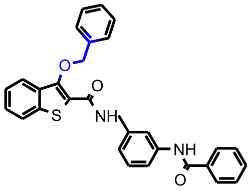 [*]OC[c](:[*]):[*]	-0.222

FCFP_6	-1539132615	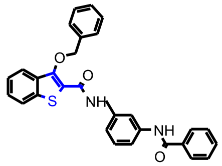 [*]C(=[*])[c]1:s:[*]:[*]:[c]:1[*]	-0.200

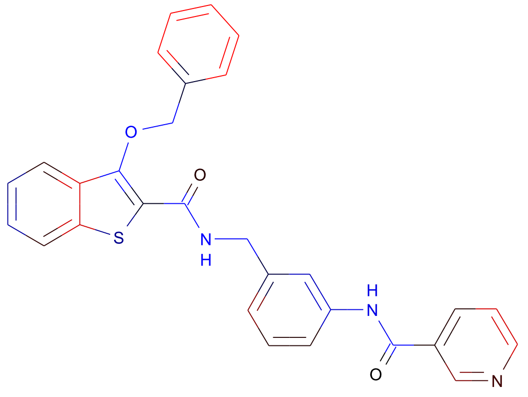

$C_{29}H_{23}N_{3}O_{3}S$

Molecular Weight: 493.57622

ALogP: 4.976

Rotatable Bonds: 8

Acceptors: 4

Donors: 2

Structural Similar Compounds

Name	BENZENESULFONIC ACID; 2;2'-(4;4'-BIPHENYLYLENEDIVINYLENE)DI-; DISODIUM SALT (Na STRIPPED)	FENDOSAL	ANTHRAQUINONE; 1;4-bis-(p-TOLYLAMINO)-
Structure	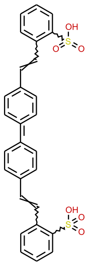	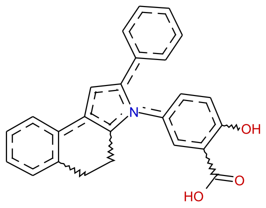	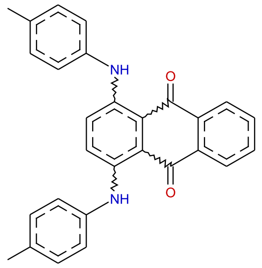
Actual Endpoint (-log C)	1.968	2.928	2.058
Predicted Endpoint (-log C)	1.72109	2.59	1.57464
Distance	0.799	0.871	0.883
Reference	MVCRB3 2;193;73	AGACBH 8;209;78	85JCAE -;1330;86

Model Prediction

Prediction: 5.308

Unit: g/kg_body_weight

Mahalanobis Distance: 25.534

Mahalanobis Distance p-value: 3.51e-034

Mahalanobis Distance: The Mahalanobis distance (MD) is a generalization of the Euclidean distance that accounts for correlations among the X properties. It is calculated as the distance to the center of the training data. The larger the MD, the less trustworthy the prediction.
Mahalanobis Distance p-value: The p-value gives the fraction of training data with an MD greater than or equal to the one for the given sample, assuming normally distributed data. The smaller the p-value, the less trustworthy the prediciton. For highly non-normal X properties (e.g., fingerprints), the MD p-value is wildly inaccurate.

Model Applicability

Unknown features are fingerprint features in the query molecule, but not found in the training set.

1. All properties and OPS components are within expected ranges.
2. Unknown ECFP_2 feature: -1670580914: [*]C(=[*])[c]1:s:[*]:[*]:[c]:1[*]
3. Unknown FCFP_6 feature: 16: [*][c](:[*]):[*]
4. Unknown FCFP_6 feature: 1747237384: [*][c]1:[*]:[*]:[c](:[*]):o:1
5. Unknown FCFP_6 feature: 1618154665: [*][c](:[*]):[cH]:[c](:[*]):[*]
6. Unknown FCFP_6 feature: 907096426: [*]NC[c](:[*]):[*]

Feature Contribution

Top features for positive contribution

Fingerprint	Bit/Smiles	Feature Structure	Score

ECFP_6	642810091	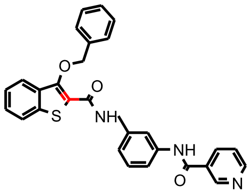 [*][c](:[*]):[*]	0.281
ECFP_6	-1897341097	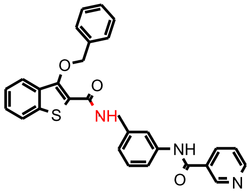 [*]N[*]	0.216
ECFP_6	1571214559	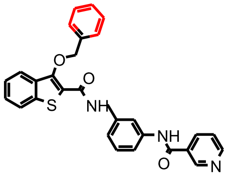 [*]1:[cH]:[cH]:[cH]:[cH]:[cH]:1	0.190

Top Features for negative contribution

Fingerprint	Bit/Smiles	Feature Structure	Score
ECFP_6	497523368	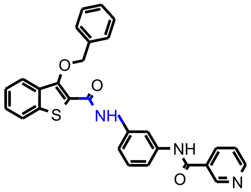 [*]CNC(=[*])[*]	-0.301
ECFP_6	655739385	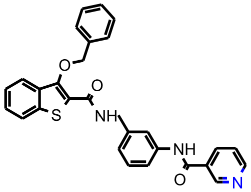 [*]:n:[*]	-0.239

FCFP_6	907036844	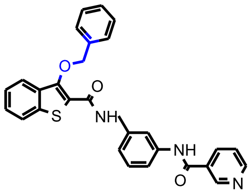<br>[*]OC[c](:[*]):[*]	-0.222

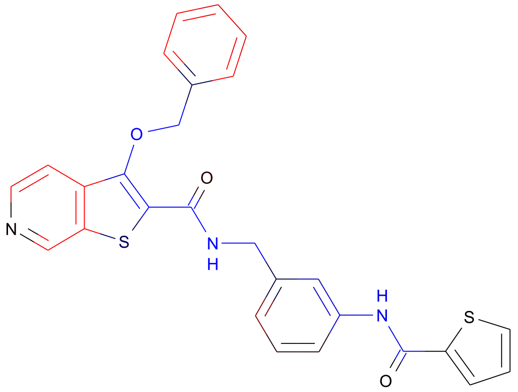

$C_{27}H_{21}N_3O_3S_2$
Molecular Weight: 499.60394
ALogP: 4.929
Rotatable Bonds: 8
Acceptors: 4
Donors: 2

Structural Similar Compounds

Name	BENZENESULFONIC ACID; 2;2'-(4;4'-BIPHENYLYLENEDIVINYLENE)DI-; DISODIUM SALT (Na STRIPPED)	ACEMETACIN	FENDOSAL
Structure	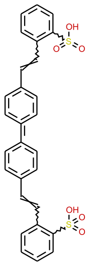	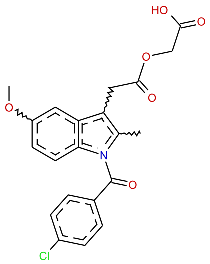	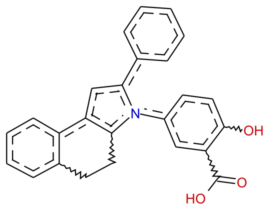
Actual Endpoint (-log C)	1.968	4.235	2.928
Predicted Endpoint (-log C)	1.72109	3.39415	2.59
Distance	0.752	0.977	0.988
Reference	MVCRB3 2;193;73	ARZNAD 30;1398;80	AGACBH 8;209;78

Model Prediction

Prediction: 1.838
Unit: g/kg_body_weight
Mahalanobis Distance: 26.236
Mahalanobis Distance p-value: 2.16e-038

Mahalanobis Distance: The Mahalanobis distance (MD) is a generalization of the Euclidean distance that accounts for correlations among the X properties. It is calculated as the distance to the center of the training data. The larger the MD, the less trustworthy the prediction.
Mahalanobis Distance p-value: The p-value gives the fraction of training data with an MD greater than or equal to the one for the given sample, assuming normally distributed data. The smaller the p-value, the less trustworthy the prediciton. For highly non-normal X properties (e.g., fingerprints), the MD p-value is wildly inaccurate.

Model Applicability

Unknown features are fingerprint features in the query molecule, but not found in the training set.

1. All properties and OPS components are within expected ranges.
2. Unknown ECFP_2 feature: -1670580914: [*]C(=[*])[c]1:s:[*]:[*]:[c]:1[*]
3. Unknown FCFP_6 feature: 16: [*][c](:[*]):[*]
4. Unknown FCFP_6 feature: 1618154665: [*][c](:[*]):[cH]:[c](:[*]):[*]
5. Unknown FCFP_6 feature: 1747237384: [*][c]1:[*]:[*]:[c](:[*]):o:1
6. Unknown FCFP_6 feature: 907096426: [*]NC[c](:[*]):[*]

Feature Contribution

Top features for positive contribution			
Fingerprint	Bit/Smiles	Feature Structure	Score

ECFP_6	642810091	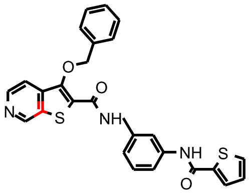 [*][c](:[*]):[*]	0.281
ECFP_6	-1897341097	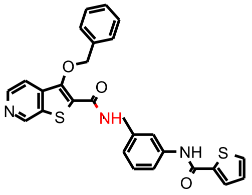 [*]N[*]	0.216
ECFP_6	1571214559	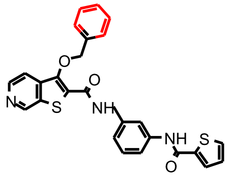 [*]1:[cH]:[cH]:[cH]:[cH]:[cH]:1	0.190

Top Features for negative contribution

**Fingerprint**	**Bit/Smiles**	**Feature Structure**	**Score**
ECFP_6	497523368	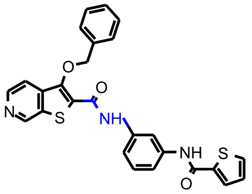 [*]CNC(=[*])[*]	-0.301
ECFP_6	655739385	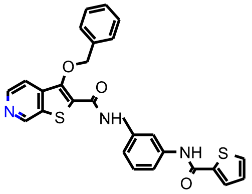 [*]:n:[*]	-0.239

FCFP_6	907036844	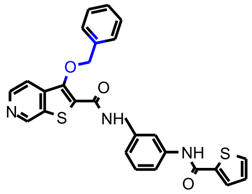 [*]OC[c](:[*]):[*]	-0.222

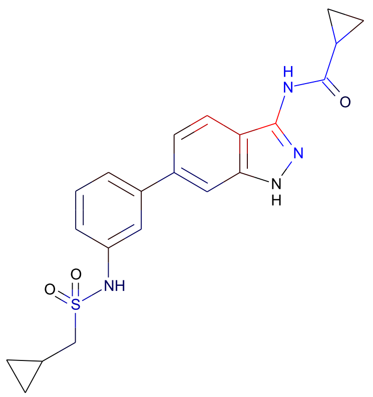

$C_{21}H_{22}N_4O_3S$
Molecular Weight: 410.48938
ALogP: 3.14
Rotatable Bonds: 7
Acceptors: 4
Donors: 3

Structural Similar Compounds

Name	CARBAMIC ACID; [1-[(5-CYANOPENTYL)CARBAMOYL]BENZIMIDAZOL-2-YL]-; METHYL ESTER	OCHRATOXIN A	SULFAQUINOXALINE
Structure	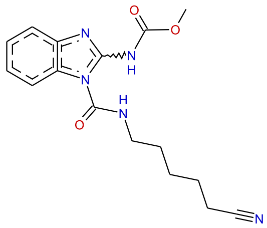	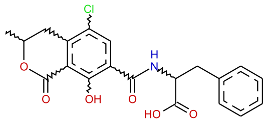	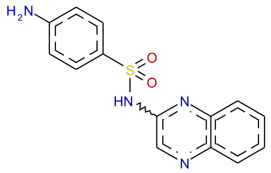
Actual Endpoint (-log C)	2.12	4.305	2.341
Predicted Endpoint (-log C)	1.78415	3.03558	2.42674
Distance	0.713	0.717	0.741
Reference	85ARAE 4;118;76/77	FCTXAV 6;479;68	MahWM# 16NOV82

Model Prediction

Prediction: 2.626
Unit: g/kg_body_weight
Mahalanobis Distance: 27.226
Mahalanobis Distance p-value: 1.13e-044

Mahalanobis Distance: The Mahalanobis distance (MD) is a generalization of the Euclidean distance that accounts for correlations among the X properties. It is calculated as the distance to the center of the training data. The larger the MD, the less trustworthy the prediction.
Mahalanobis Distance p-value: The p-value gives the fraction of training data with an MD greater than or equal to the one for the given sample, assuming normally distributed data. The smaller the p-value, the less trustworthy the prediciton. For highly non-normal X properties (e.g., fingerprints), the MD p-value is wildly inaccurate.

Model Applicability

Unknown features are fingerprint features in the query molecule, but not found in the training set.

1. All properties and OPS components are within expected ranges.
2. Unknown ECFP_2 feature: 268744321: [*]CS(=O)(=O)N[*]
3. Unknown ECFP_2 feature: -1341194584: [*]S(=[*])(=[*])CC1[*][*]1
4. Unknown FCFP_6 feature: 19: [*]:[nH]:[*]
5. Unknown FCFP_6 feature: 16: [*][c](:[*]):[*]
6. Unknown FCFP_6 feature: 262592487: [*]:[c]1:[*]:[*]:n:[nH]:1
7. Unknown FCFP_6 feature: 1747267175: [*][c]1:[*]:[*]:[nH]:n:1
8. Unknown FCFP_6 feature: -1151884458: [*]N[c]1:n:[*]:[*]:[c]:1:[*]
9. Unknown FCFP_6 feature: 1618154665: [*][c](:[*]):[cH]:[c](:[*]):[*]
10. Unknown FCFP_6 feature: -1861645784: [*]:[cH]:[c](:[cH]:[*])[c](:[*]):[*]
11. Unknown FCFP_6 feature: 307448885: [*]:[cH]:[c]1:[nH]:[*]:[*]:[c]:1:[*]
12. Unknown FCFP_6 feature: 1018942292: [*]CS(=O)(=O)N[*]

Feature Contribution

Top features for positive contribution

Fingerprint	Bit/Smiles	Feature Structure	Score

ECFP_6	642810091	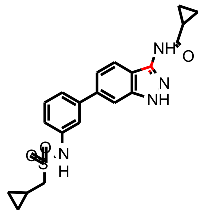 [*][c](:[*]):[*]	0.281
ECFP_6	-1897341097	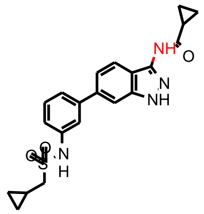 [*]N[*]	0.216
ECFP_6	-1074141656	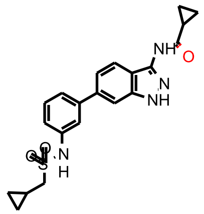 [*]=O	0.142

Top Features for negative contribution

Fingerprint	Bit/Smiles	Feature Structure	Score
ECFP_6	655739385	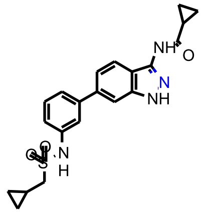 [*]:n:[*]	-0.239
FCFP_6	566058135	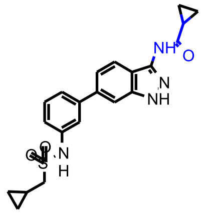 [*]CC(=O)N	-0.216

FCFP_6	3	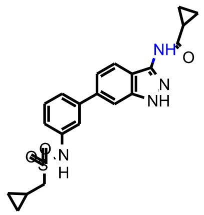 [*]N	-0.107
